# An Adaptive Rhodium Catalyst to Control the Hydrogenation Network of Nitroarenes

**DOI:** 10.1002/anie.202205515

**Published:** 2022-08-01

**Authors:** Vishal Chugh, Basujit Chatterjee, Wei‐Chieh Chang, Hanna H. Cramer, Carsten Hindemith, Helena Randel, Thomas Weyhermüller, Christophe Farès, Christophe Werlé

**Affiliations:** ^1^ Max Planck Institute for Chemical Energy Conversion Stiftstr. 34–36 45470 Mülheim an der Ruhr Germany; ^2^ Ruhr University Bochum Universitätsstr. 150 44801 Bochum Germany; ^3^ Max-Planck-Institut für Kohlenforschung Kaiser-Wilhelm-Platz 1 45470 Mülheim an der Ruhr Germany

**Keywords:** Adaptive Catalysis, Anilines, Hydrogenation, Hydroxylamines, Nitroarenes

## Abstract

An adaptive catalytic system that provides control over the nitroarene hydrogenation network to prepare a wide range of aniline and hydroxylamine derivatives is presented. This system takes advantage of a delicate interplay between a rhodium(III) center and a Lewis acidic borane introduced in the secondary coordination sphere of the metal. The high chemoselectivity of the catalyst in the presence of various potentially vulnerable functional groups and its readiness to be deployed at a preparative scale illustrate its practicality. Mechanistic studies and density functional theory (DFT) methods were used to shed light on the mode of functioning of the catalyst and elucidate the origin of adaptivity. The competition for interaction with boron between a solvent molecule and a substrate was found crucial for adaptivity. When operating in THF, the reduction network stops at the hydroxylamine platform, whereas the reaction can be directed to the aniline platform in toluene.

## Graphical abstract

An adaptive rhodium‐based catalyst for controlled hydrogenation of nitroarenes to hydroxylamines and anilines is presented. Both product platforms could be accessed under mild conditions and excellent yields using molecular hydrogen as the reducing agent. Mechanistic studies and density functional theory (DFT) methods were used to shed light on the mode of functioning of the catalyst and elucidate the origin of adaptivity.

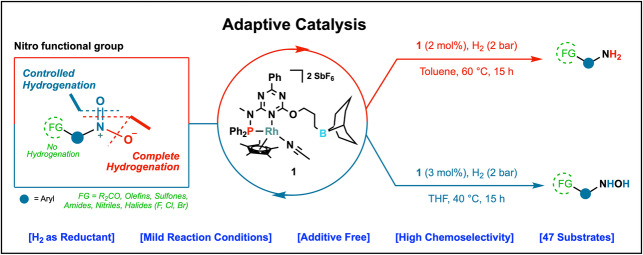

## Introduction

Nature's chemical machinery can perform a myriad of parallel processes with unique levels of efficiency, capable of providing various products in high selectivity.^[1]^ It shows that precise control over the bond activation processes and successive catalytic sequences can be achieved. By contrast, catalytic protocols are traditionally designed to transform substrates exclusively into a single product.^[2]^ Another paradigm might evolve from dynamic catalytic systems that adapt their activity based on subtle changes in reaction conditions in order to create distinct products from a given substrate.^[3]^

**Scheme 1:**
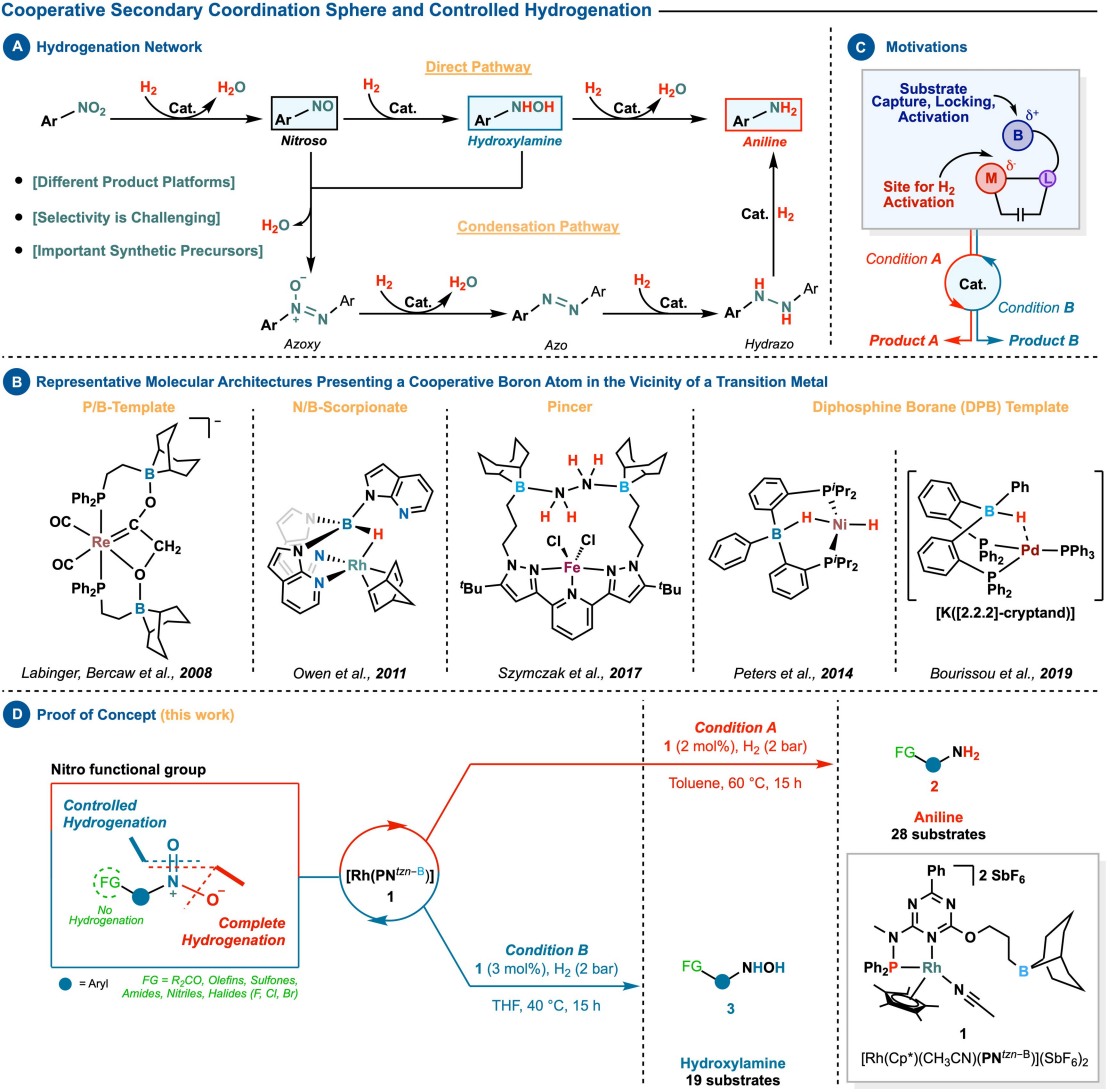
Development of an adaptive rhodium‐based catalyst for controlled hydrogenation.

In this context, the hydrogenation network of nitroarenes on the way to fully reduced aniline comprises several potentially accessible product platforms (Scheme 1, panel A).[^4^, ^5^] Our aim in this study was to examine if an adaptive catalyst could be used to control this hydrogenation network and selectively stop at desired reduction levels. Such an approach could, for instance, provide a direct route to aniline or hydroxylamine motifs which are found in many bioactive molecules, natural products, drugs, and synthetically valuable chemicals.^[6]^

However, in order to make this strategy synthetically viable, certain preconditions must be met: (1) the catalytic system must be capable of navigating the hydrogenation network; (2) selectively stop at the desired reduction level; and (3) tolerate other reactive functional groups embedded within the substrate.

To this end, we decided to use rhodium as a metal center because of its high propensity to drive hydrogenation reactions,^[7]^ provided that the different prerequisites can be met. Additionally, we were inspired by molecular architectures that contain a Lewis acid center (such as boron) in the vicinity of a transition metal (Scheme 1, panel B).^[8]^ Recent attention has been drawn to these structures aligned with the concepts of metal‐ligand cooperation,[^9^, ^10^] because of their ability to activate chemical bonds.

We hypothesized that implementing a polarized cooperative domain within a catalytic system might develop in an adaptive platform. Our approach consisted of using a ligand environment with an attached borane arm to challenge and tame rhodium's reactivity. Because both partners have unique electronic properties, a polarized environment is created, which can be used to capture, lock, activate, and convert substrates (Scheme 1, panel C).[^10e^, ^11^]

The developed system (**1**) proved capable of supplying anilines (complete hydrogenation) and hydroxylamines (controlled hydrogenation) in excellent yields under mild conditions while preserving the integrity of other potentially vulnerable functional groups (Scheme 1, panel D).

## Results and Discussion

**Scheme 2:**
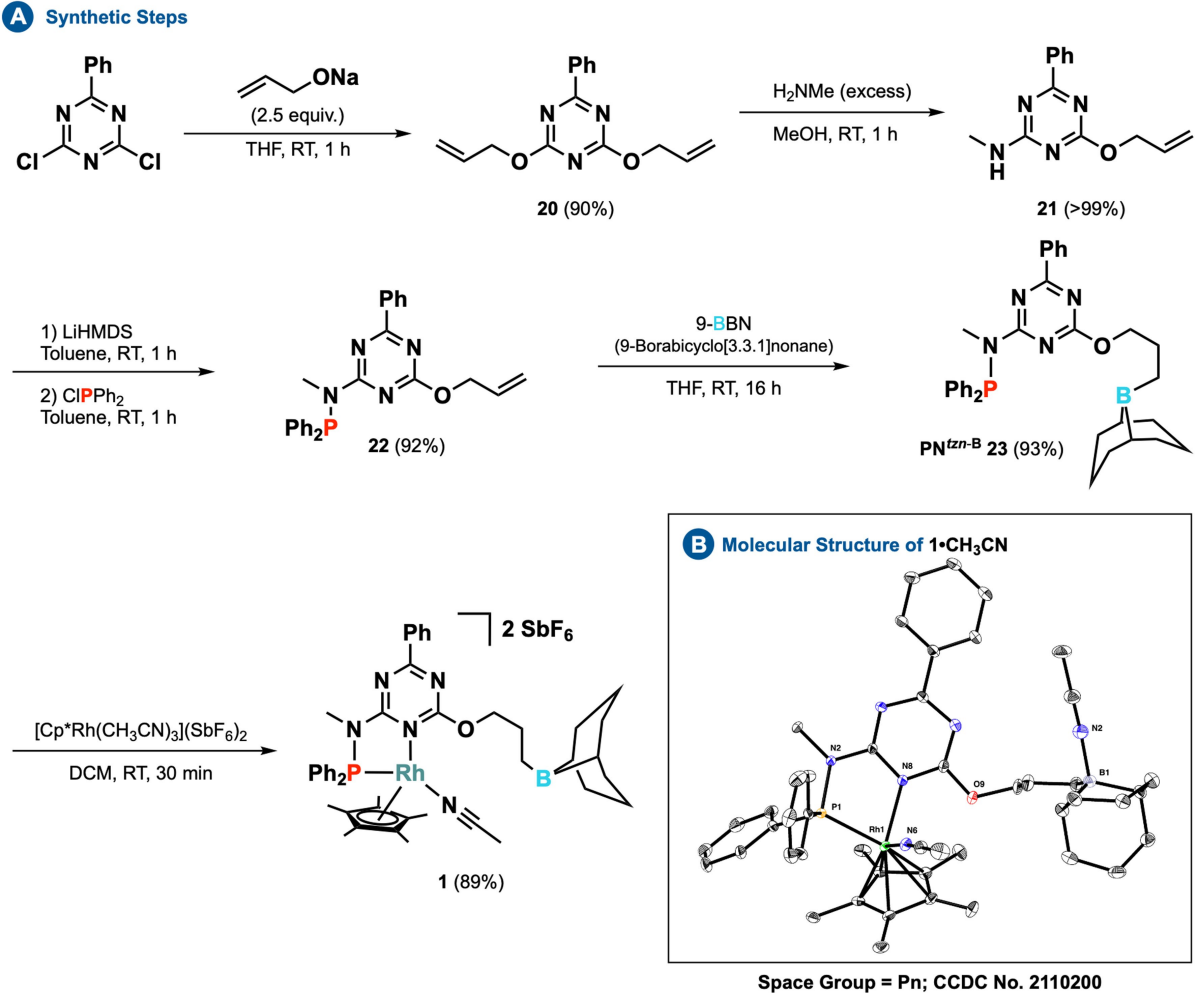
Synthesis of PN^*tzn‐B*^ ligand **23** and the associated rhodium complex (**1**).

To evaluate the feasibility of the concept, we examined a rhodium complex with a triazine **PN** ^*tzn‐B*^ ligand (**23**), which was designed to form a well‐defined structural and electronic environment for the metal center (Scheme 2). As a proxy to control the kinetic and thermodynamic stability of the complex, this ligand architecture combines hard and soft donor centers.^[12]^ The electron‐withdrawing triazine core acts as a π‐acceptor unit, while an electron‐donating phosphine attached through nitrogen to the ring serves as an anchor. Finally, a boron‐based secondary coordination sphere is used to influence the capture and conversion of substrates. Synthetic details and crystallographic information^[13]^ confirming the projected binding scenario of **1** are provided in the Supporting Information.^[14]^

As a way of evaluating the practicality of the concept, we then set up the reaction conditions of the standard protocols derived from a detailed screening with 4′‐nitroacetophenone as a benchmark substrate (Table 1). This substrate was chosen because it contains another potentially vulnerable functional group (i.e., ketone) as an indicator for probing the selectivity of the catalyst towards nitro‐reduction over other functional groups.

**Table 1:**
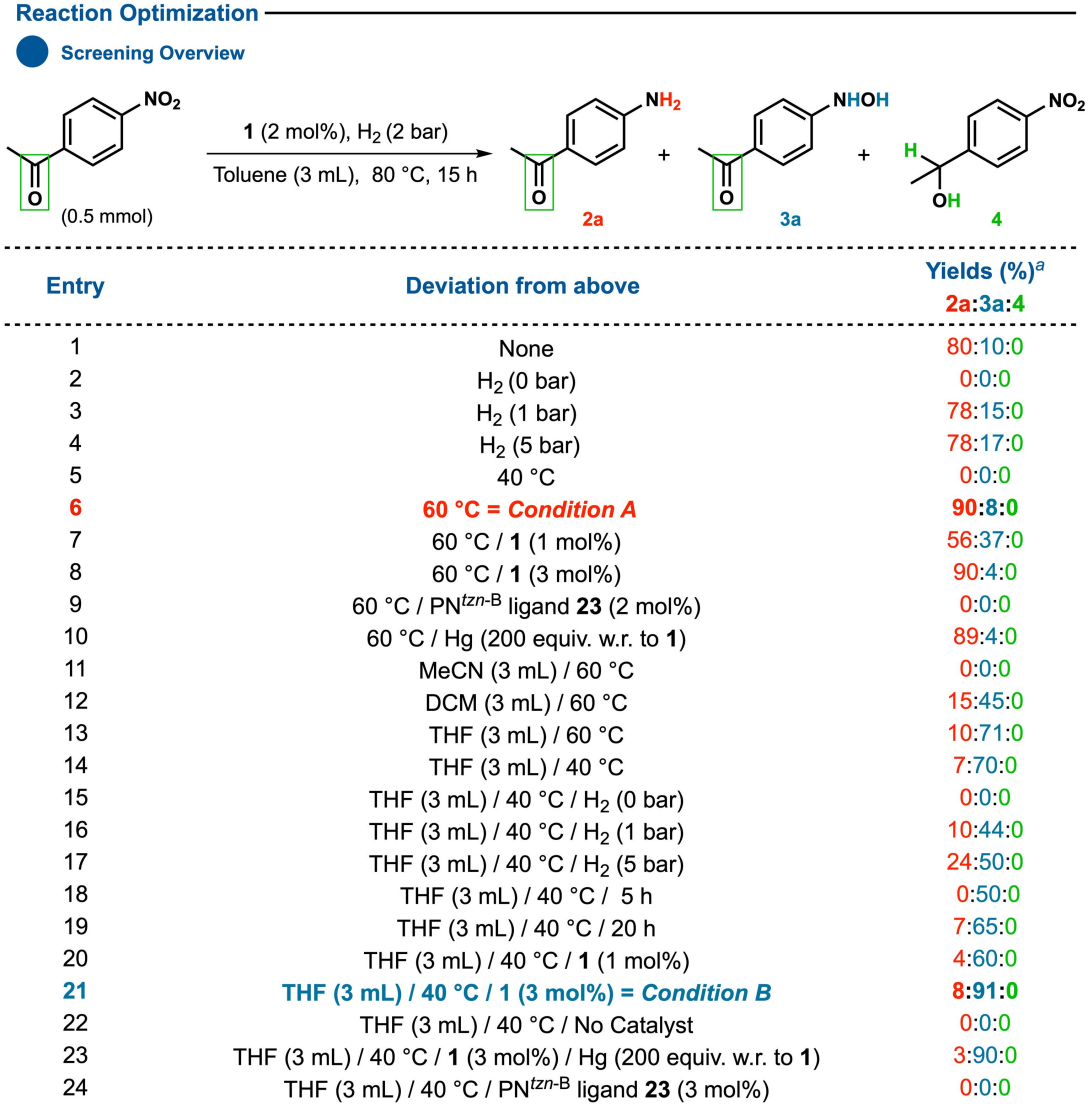
Optimization of the reaction conditions for aniline and hydroxylamine synthesis.

[a] Yields are based on ^1^H NMR relative to mesitylene (0.5 mmol) as an internal standard.

Initial screening, conducted in toluene under H_2_ (2 bars) at 80 °C, led to aniline **2 a** (80 %) and hydroxylamine **3 a** (10 %) as products, with the ketone remaining unaltered (Table 1, entry 1). The next step involved optimizing the reaction conditions for the aniline product platform. Varying the pressure did not improve selectivity toward **2 a** (Table 1, entries 3 and 4), whereas reducing the temperature (60 °C) favorably increased the yield (90 %). A detailed examination of other reaction parameters (i.e., time, solvents, catalyst loading; see the Supporting Information for more information) revealed *condition **A*** (Table 1, entry 6) as optimized for accessing the aniline product platform. While examining alternative solvents, a significant finding was that using tetrahydrofuran caused the catalyst to predominantly produce **3 a** instead of **2 a**. Again, a detailed review of the reaction parameters (i.e., catalyst loading, temperature, time, pressure) assessed *condition **B*** (Table 1, entry 21) involving **1** (3 mol %) in THF at 40 °C under H_2_ (2 bars) for 15 hours as ideal for the formation of **3 a** (91 %).^[15]^ Further control experiments confirmed that both **1** and H_2_ are required for the reaction to proceed (Table 1, entries 22 and 2), and the molecular nature of the catalyst during the reaction (Table 1, entries 10, 23).^[16]^ In addition, there is no indication at that stage that the final reduction step leading to aniline could involve the disproportionation^[17]^ of hydroxylamine under *condition **A***.^[18]^

**Scheme 3:**
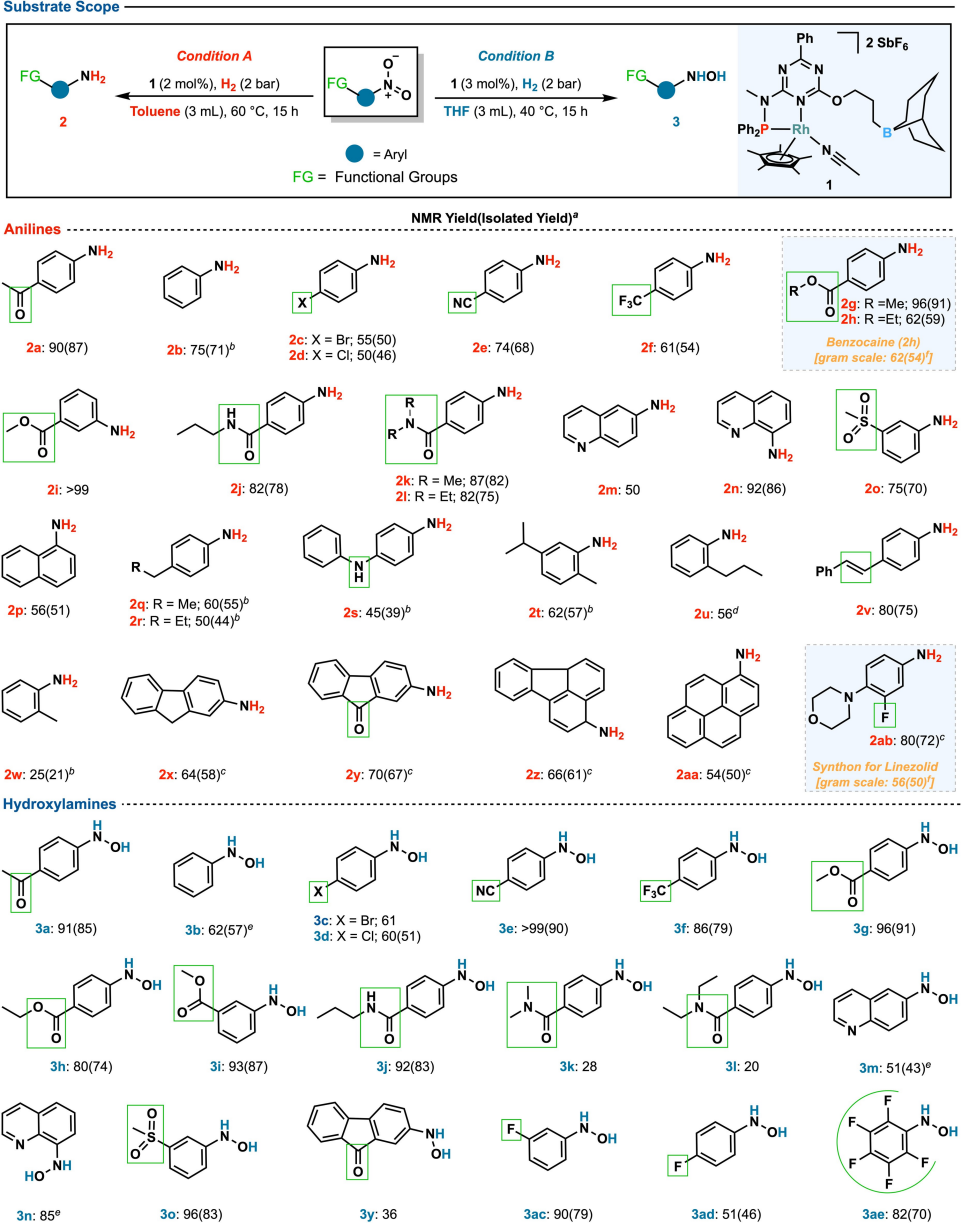
Substrate scope of anilines **2** and hydroxylamines **3** obtained under optimized conditions. [a] Conversions [%] and Yields [%] are based on ^1^H NMR relative to mesitylene (0.5 mmol) as an internal standard. Isolated yields [%] are given in brackets. Deviations from conditions **A** or **B**: [b] 80 °C, 5 bar; [c] 80 °C; [d] 100 °C, 5 bar; [e] 60 °C, 5 bar; [f] 20 h.

The optimized conditions (Table 1, entries 6 and 21) were then used to examine the generality of the protocol (Scheme 3). Hence, substrates with diverse functionalities were evaluated for their ability to withstand the established reaction conditions while maintaining the integrity of other potentially reactive functional groups. Halogen‐containing aromatic rings supported the two optimized conditions well, producing the desired anilines (**2 c**, **d**) and hydroxylamines (**3 c**, **d**, **3 ac–ae**) in good yields. Also, nitroarenes with a *para*‐trifluoromethyl group were tolerated under the reaction conditions, resulting in the targeted products (**2 f**, **3 f**). This confirms that substrates sensitive to hydrodehalogenation reactions are well tolerated under this protocol.^[5j]^ Additionally, the system showed high tolerance to carbonyl‐containing substrates in which ketones (**2 a**, **2 y**, **3 a**), esters (**2 g**–**i**, **3 g**–**i**), and amides (**2 j**–**l**, **3 j**–**l**) functional groups were preserved. Substrates with sulfone, nitrile, and olefin groups also withstood the reaction conditions, supplying the corresponding products (**2 o**, **2 e**, **2 v**, **3 o**, **3 e**) in good yields. The presence of aliphatic chains, especially when located at the *ortho* position of the nitro group, did not impede the reaction, and the corresponding anilines (**2 q**, **r**, **2 t**, **u**, **2 w**) were produced effectively. When the nitro functionality was incorporated onto heteroarenes, good to excellent yields were achieved for the corresponding anilines (**2 m**, **n**) and hydroxylamines (**3 m**, **n**). Then, preparative scale reactions were conducted to verify the method's practicality. A topical analgesic, benzocaine (**2 h**), was synthesized on a gram‐scale with an isolated output of 54 % using *condition **A***. In the same way, **2 ab**, a key synthon for the synthesis of linezolid, an antibiotic used to treat Gram‐positive bacterial infections, was obtained in 50 % yield under *condition **A***.

**Scheme 4:**
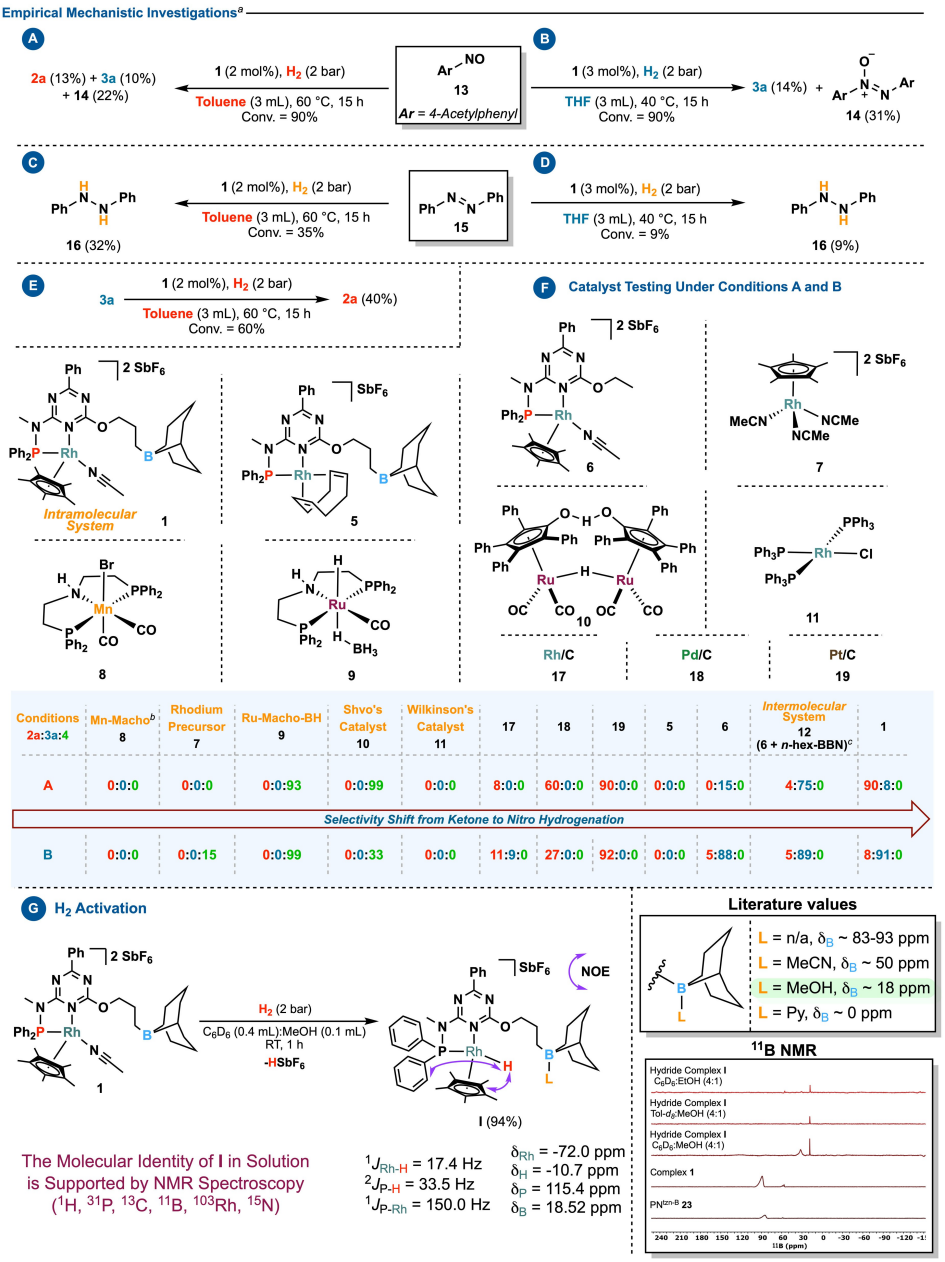
Empirical mechanistic investigations to elucidate the reaction network. [a] Yields are based on ^1^H NMR relative to mesitylene (0.5 mmol) as an internal standard. [b] KO^*t*^Bu (*condition **A***: 2 mol %; *condition **B***: 3 mol %) was used to activate complex **8**. [c] *n*‐hex‐BBN (*condition **A***: 2 mol %; *condition **B***: 3 mol %) was used for intermolecular system **12**. For the literature values of the ^11^B chemical shifts, see Ref. [26] and the Supporting Information for further details.

Several control experiments were conducted to understand better the mode of functioning and adaptivity of the catalyst (Scheme 4).^[19]^ The first step was to investigate the catalytic network, especially the significance of the nitroso platform. After submitting 1‐(4‐nitrosophenyl)‐ethan‐1‐one (**13**) to *condition **A***, a mixture of hydroxylamine **3 a** (10 %), azoxy **14** (22 %), and aniline **2 a** (13 %) was obtained (Scheme 4, panel A).^[20]^ In contrast, *condition **B*** yielded a mixture of **3 a** (14 %) and **14** (31 %) but without aniline (Scheme 4, panel B). The fact that aniline is formed under *condition **A*** but not under *condition **B*** suggests that the nitroso platform (**13**) is part of the hydrogenation network as navigated by **1**. Furthermore, the large amount of azoxybenzene **14** in the reaction medium suggests that the catalyst may have some difficulty passing through the condensation route. When azobenzene **15** was used as a substrate in both sets of optimized conditions (Scheme 4, panels C and D), the corresponding hydrazobenzene **16** was obtained in low yields (*condition **A***: 32 % and *condition **B***: 9 %). Additionally, aniline formation was not observed under *condition **A***. As a result, it is less likely that catalyst **1** passes through a condensation path. By submitting hydroxylamine **3 a** to the standard protocol for aniline formation, **2 a** was obtained in 40 % yield (Scheme 4, panel E).^[21]^ Therefore, hydroxylamine is an integral part of the reaction mechanism, supporting the theory that the catalyst follows a direct pathway.^[22]^

In the next step, we evaluated the importance and role of the ligand system by comparing **1** with well‐established hydrogenation/transfer‐hydrogenation catalysts (Scheme 4, panel F). All the catalysts tested (i.e., **8**–**11**) either showed no reactivity or only hydrogenated the ketone, resulting in **4**. When supported metal catalysts (**17**–**19**) were used, only aniline was produced under conditions **A** and **B**. In contrast to **1**, the rhodium(I) variation (**5**) failed to undergo the targeted transformation.^[23]^ To assess the role of the ligand framework, we then prepared **PN** ^*tzn‐OEt*^ *‐*Rh^III^ complex **6**, in which the triazine ring was retained while an ethyl group was introduced in place of the borane arm.^[24]^ This system enabled the hydrogenation of the nitro group while maintaining the integrity of the ketone. However, as opposed to **1**, only the hydroxylamine platform was produced in low to good yields (*condition **A***: 15 %, *condition **B***: 88 %). There appears to be a dominant role for the triazine ring in selectively reducing the nitro group while leaving other potentially vulnerable functionalities (like ketone) intact.

The role of boron was then assessed using the intermolecular version (**12**) of catalyst **1** involving complex **6** and an external borane additive (i.e., 9‐hexyl‐9‐borabicyclo[3.3.1]nonane). As a result, system **12** produced more hydroxylamine **3 a** (75 %) under *condition **A*** than any of the other tested catalysts, including **1**. However, neither optimized conditions **A** nor **B** enabled **12** to efficiently produce aniline **2 a**, which could only be synthesized at low yield (*condition **A***: 4 %, *condition **B***: 5 %). We then considered possible catalytically relevant metal‐based intermediates. Hence, **1** was exposed to a H_2_ atmosphere (2 bars) at room temperature (RT) in a mixture of C_6_D_6_/MeOH (4 : 1, 0.5 mL) solvents. Under these conditions, the formation of a metal‐centered hydride species (complex **I**, *δ*_Rh‐H_=−10.7 ppm, dd, *J*=33.5, 17.4 Hz; Scheme 4, panel G) was observed.^[25]^

**Figure 1:**
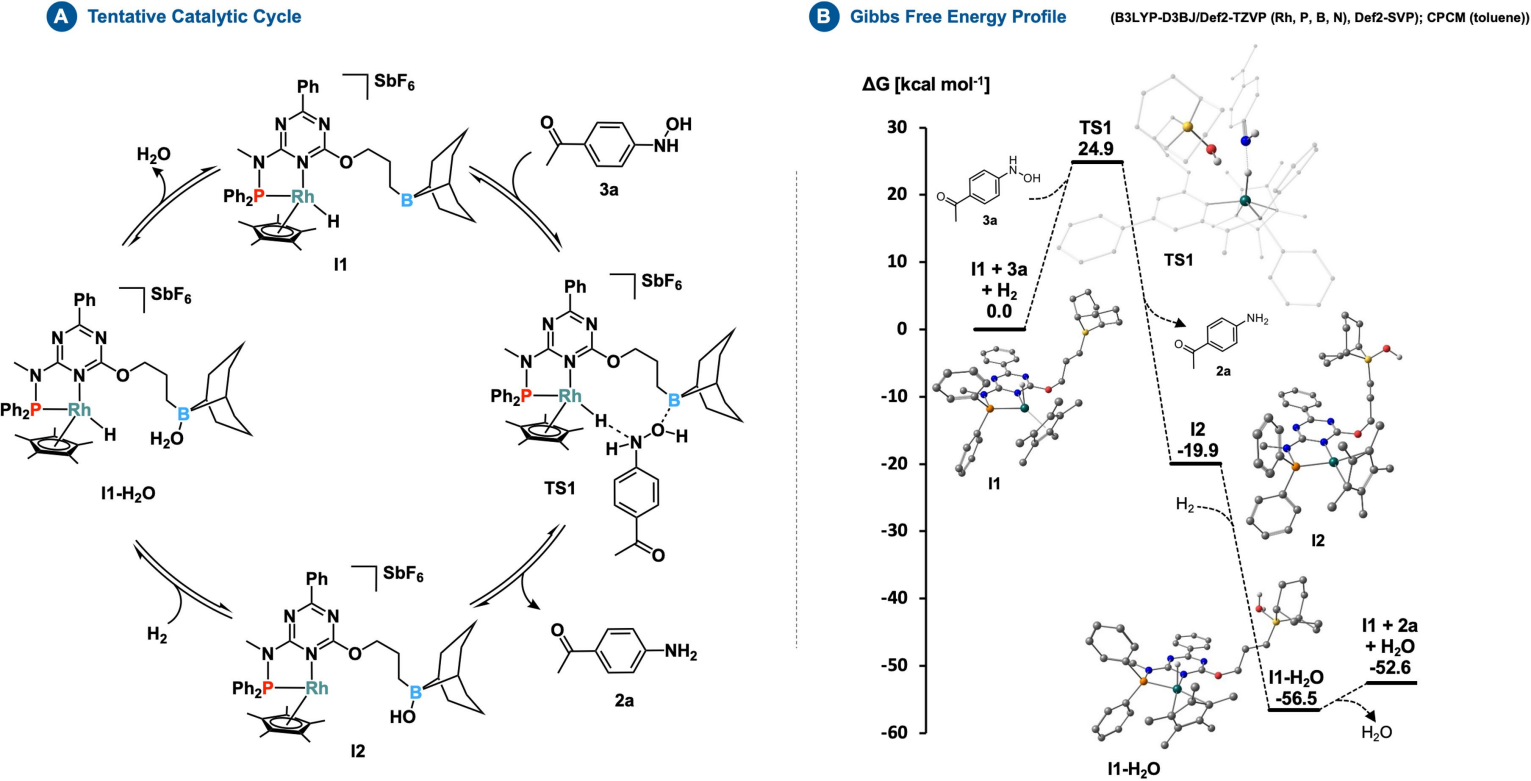
Tentative catalytic cycle (Panel A) and Gibbs free energy profile (Panel B) of the reduction of hydroxylamine **3 a** to aniline **2 a**.

Our next objective was to investigate the source of the adaptivity and selectivity of the catalyst. One important experimental observation was that complex **6** did not carry out the final reduction step that converts **3 a** into **2 a**. This suggests that the borane arm in **1** has a specific effect on aniline formation. To obtain further insights, we have studied the catalytic mechanism of the reduction of hydroxylamine **3 a** to aniline **2 a** with density functional theory (DFT) methods, using the B3LYP functional with D3BJ empirical correction^[27]^ and a mixed basis set combination of Def2‐TZVP (Rh, P, B, N)^[28]^ and Def2‐SVP.^[29]^ Our first observation was that, in the absence of the borane arm, hydroxylamine **3 a** remains insensitive to the nucleophilic attack of the hydride. Based on relaxed surface scans (Figure S209), we determined that, without the borane arm, the interaction between **3 a** and the hydride of **I1** leads to a constant rise in energy without significant elongation of the N−O bond (1.41 to 1.43 Å). To facilitate the cleavage of the N−O bond, we, therefore, hypothesized that the borane might act as a hydroxide acceptor (Figure 1, Figure S210) and, through a concerted process (**TS1**; relative energy of 24.9 kcal mol^−1^), promote the hydride transfer from the rhodium center to the N‐atom (Table S11). Subsequently, after dissociation of aniline **2 a**, **TS1** relaxes to complex **I2** with a relative energy of −19.9 kcal mol^−1^. In the next step, the heterolytic cleavage of H_2_ by **I2** results in the formation of **I1‐H_2_O** containing a rhodium‐bound hydride and a water molecule coordinated to the borane arm. This step proceeds without apparent barriers, as indicated by relaxed surface scans (Figure S211), and is followed by the regeneration of **I1** after the dissociation of water. Moreover, we determined that the reaction between **3 a** and H_2_ resulting in the formation of **2 a** and water was highly exothermic, as indicated by a Gibbs free energy of −52.6 kcal mol^−1^. The overall kinetic barrier of the proposed mechanism is determined by **TS1** and **I1‐H_2_O**, and the associated energy span of 28.9 kcal mol^−1^ is consistent with the fact that the reaction takes several hours at elevated temperatures (60 °C) to proceed.

Next, we wondered whether the formation of solvent‐borane‐adducts could explain the selectivity for hydroxylamine in tetrahydrofuran. This type of adduct might prevent the borane from participating in the cleavage of the N−O bond. We thus calculated the Gibbs free energy of various borane adducts of **I1** (Table 2). The formation of adducts with ethanol, methanol, acetonitrile, and diethyl ether is close to thermoneutral. This suggests that Lewis adduct formation between the borane and the solvent competes with N−O bond cleavage via **TS1**, particularly when the reaction is carried out in these solvents, which are then present in large local concentrations and may thereby inhibit aniline formation. This hypothesis is in agreement with the experimental observation that the reaction stops at the hydroxylamine reduction level in tetrahydrofuran and methanol as solvents, whereas it can reach the aniline reduction level in non‐coordinating solvents such as toluene.

**Table 2:**
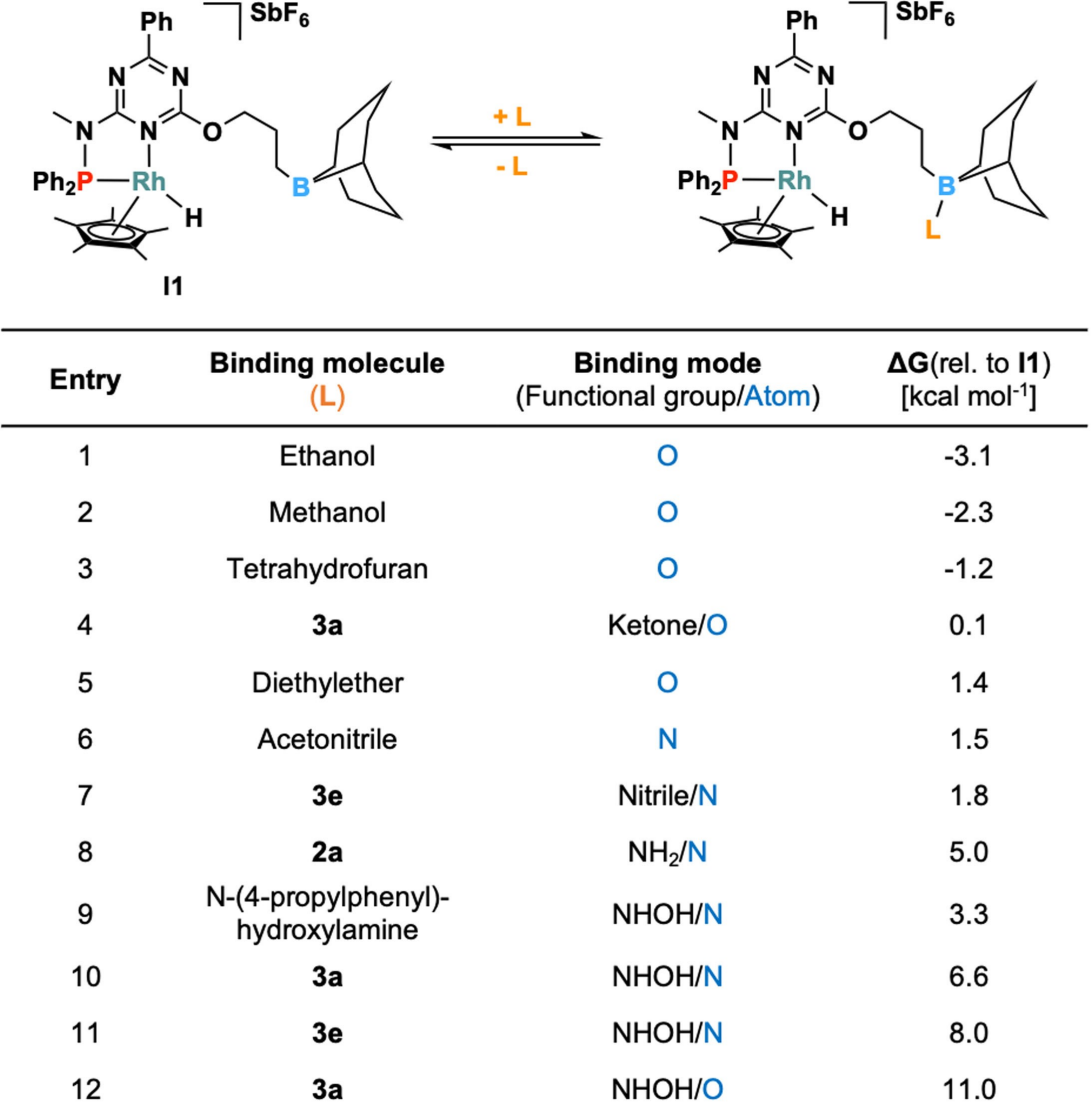
Relative Gibbs free energies [kcal mol^−1^] of selected borane adducts of **I1**.

Additionally, we explored whether specific functional groups embedded in substrates (e.g., nitrile, ketone, and amine groups) might impair hydroxylamine to aniline conversion. The values obtained for the interactions between the nitrile functionality in **3 e** (1.8 kcal mol^−1^) or the acetyl group in **3 a** (0.1 kcal mol^−1^) with the borane are comparable to those measured for acetonitrile or tetrahydrofuran. However, in contrast to coordinating solvents, these functional groups do not inhibit aniline formation, as evidenced by the obtained NMR yields of **2 e** (74 %) and **2 a** (90 %). A possible explanation is that the concentration of substrate molecules, or the amount of water generated during the reaction, is much lower than the concentration of solvent molecules. Finally, it has been determined that borane undergoes relatively weak interactions with either the NH_2_ unit in aniline **2 a** or with the oxygen/nitrogen atoms in hydroxylamine **3 a** (ca. 3.3–11.0 kcal mol^−1^, see Table 2, entries 9–12).

## Conclusion

In conclusion, we have developed an adaptive rhodium‐based system that enables molecular control over the hydrogenation network of nitroarenes by responding to subtle changes in the reaction conditions. As a result, a wide range of aniline and hydroxylamine derivatives could be synthesized under mild conditions in good to excellent yields. The versatility of the catalyst is demonstrated by its tolerance of a large number of functional groups and its capability to synthesize biologically relevant molecules at a preparative scale. Experimental mechanistic studies and DFT calculations revealed: 1) the importance of the designed ligand environment for providing the catalyst with adaptive properties; 2) the role of the solvent medium in controlling the selectivity of the catalyst; 3) the formation of a rhodium‐hydride intermediate capable of 4) transferring the hydride to the boron‐activated site. At present, more studies are being conducted to gain a more comprehensive understanding of how this catalyst system works and extend its scope to other chemical transformations.

## Conflict of interest

The authors declare no conflict of interest.

1

## Supporting information

As a service to our authors and readers, this journal provides supporting information supplied by the authors. Such materials are peer reviewed and may be re‐organized for online delivery, but are not copy‐edited or typeset. Technical support issues arising from supporting information (other than missing files) should be addressed to the authors.

Supporting Information

## Data Availability

The data that support the findings of this study are available in the Supporting Information of this article.
