# Supplementary material for: An Adaptive Rhodium Catalyst to Control the Hydrogenation Network of Nitroarenes
Source: Angew Chem Int Ed Engl. 2022 Aug 1;61(36):e202205515. doi: 10.1002/anie.202205515 (PMC9544374; doi:10.1002/anie.202205515)
Supplement: Supplementary file 1 — Supporting Information [file ANIE-61-0-s001.pdf]

Angewandte Chemie
Eine Zeitschrift der Gesellschaft Deutscher Chemiker

Supporting Information

An Adaptive Rhodium Catalyst to Control the Hydrogenation Network of Nitroarenes

*V. Chugh, B. Chatterjee, W.-C. Chang, H. H. Cramer, C. Hindemith, H. Randel, T. Weyhermüller, C. Farès, C. Werlé**

Supporting Information

An Adaptive Rhodium Catalyst to Control the Hydrogenation Network of Nitroarenes

Vishal Chugh,[1,2] Basujit Chatterjee,[1,2] Wei-Chieh Chang,[1,2] Hanna H. Cramer,[1] Carsten Hindemith,[1,2] Helena Randel,[1,2] Thomas Weyhermüller,[1] Christophe Farès,[3] and Christophe Werlé[1,2,*]

[1] Max Planck Institute for Chemical Energy Conversion, Stiftstr. 34 – 36, 45470 Mülheim an der Ruhr, Germany.

[2] Ruhr University Bochum, Universitätsstr. 150, 44801 Bochum, Germany.

[3] Max-Planck-Institut für Kohlenforschung, Kaiser-Wilhelm-Platz 1, 45470 Mülheim an der Ruhr, Germany

* Email: christophe.werle@cec.mpg.de

Table of Contents

General Methods **S5**

Ligand Synthesis and Characterization **S7**

1. Synthesis of Triazine **20** S7
2. Synthesis of Triazine **21** S9
3. Synthesis of Triazine **22** S12
4. Synthesis of Triazine **23** S15
5. Synthesis of Triazine **24** S18
6. Synthesis of Triazine **25** S20
7. Synthesis of Triazine **26** S23

Complex Synthesis and Characterization **S26**

1. Synthesis of $[Rh(Cp^*)(MeCN)(PN^{tzn-B})](SbF_6)_2$ Complex (**1**) S26
2. Synthesis of $[Rh(Cp^*)(MeCN)(PN^{tzn-OEt})](SbF_6)_2$ Complex (**6**) S31
3. Synthesis of $[Rh(COD)(PN^{tzn-B})](SbF_6)$ Complex (**5**) S36

Optimization of Reaction Parameters for Hydroxylamine Synthesis **S42**

1. Solvent S42
2. Temperature S42
3. Time S43
4. Pressure S43
5. Catalyst Loading S44

Optimization of Reaction Parameters for Anilines Synthesis. **S44**

1. Pressure S44

2. Temperature S45
3. Solvent S45
4. Catalyst Loading S46

General Procedure for Aniline Synthesis........ S47

- Preparation of Anilines S47

General Procedure for Hydroxylamine Synthesis S60

- Preparation of Hydroxylamines S60

Preparative Scale Reactions........ S68

1. Benzocaine (**2h**) S68
2. Synthon for Linezolid (**2ab**) S69

Empirical Mechanistic Studies S70

1. Catalyst Screening Under Optimized Conditions S70
2. Poisoning Experiments S80
3. Reactions Using Water Instead of H_2 as the Reducing Agent S84
   - Using Water (1 equiv.) Instead of H_2........ S84
   - Using Water (2 equiv.) Instead of H_2........ S86
   - Using Water (Excess) Instead of H_2........ S88
4. Reaction with Water as an Additive in the Presence of H_2........ S90
5. Testing the Possibility of a Disproportionation Reaction S91
6. Temporal Conversion Profiles S92
7. Identifying the Reaction Network........ S93
8. Dihydrogen Activation (Metal-Hydride Formation)........ S98
   - Complex **1** in the Presence of H_2 .... S100
   - Complex **1** in the Presence of D_2 .... S108
   - The Reaction of Complex **5** with H_2........ S111
9. Role of Secondary Coordination Sphere........ S115
   - Reaction Optimization Using MeOH as the Reaction Solvent S116
   - Substrate Scope in MeOH as the Reaction Solvent S117
   - NMR Spectra of the Crude Reaction Mixtures Using MeOH as Solvent for the Substrate Scope........ S118

^{1}H and ^{13}C Spectra S127

Quantum Chemical Calculations........ S171

General Procedure........ S171

Key Bond Lengths and Angles of Selected Structures S172

Relaxed Surface Scans........ S173

- Interaction of Hydroxylamine and **I1** Without Borane S173

- Concerted N–O Bond Cleavage and Hydride Transfer *via* **TS1** by the Interaction of Hydroxylamine and **I1** S173
- H_2 Cleavage Facilitated by **I2** Yielding **I1-H$_2$O** S174

1. Gibbs Free Energies with Implicit Solvation of Toluene and THF S174
2. Electronic Energies and Corrections S175
3. XYZ Coordinates of the Optimized Structures | Toluene S178

- [**23**_RhH_B]$^+$ (**I1**) S178
- **TS1** S179
- [**23**_Rh_BOH]$^+$ (**I2**) S180
- [**23**_Rh_ACN_B_ACN]$^{2+}$ S181
- [**23**_RhH_B_ACN]$^+$ S182
- [**23**_RhH_B_THF]$^+$ S183
- [**23**_RhH_B_CH_2Cl_2]$^+$ S185
- [**23**_RhH_B_Et_2O]$^+$ S186
- [**23**_RhH_B_H_2O]$^+$ S187
- [**23**_RhH_B_EtOH]$^+$ S188
- [**23**_RhH_B_MeOH]$^+$ S189
- [**23**_RhH_B_**3a**]$^+$ (Coordination *via* NHOH/N) S190
- [**23**_RhH_B_**3a**]$^+$ (Coordination *via* NHOH/O) S192
- [**23**_RhH_B_**2a**]$^+$ (Coordination *via* NH_2) S193
- [**23**_RhH_B_**3e**]$^+$ (Coordination *via* NHOH) S194
- [**23**_RhH_B_(*N*-(4-propylphenyl)-hydroxylamine)]$^+$ S196
- [**23**_RhH_B_**3e**]$^+$ (Coordination *via* Nitrile) S197
- [**23**_RhH_B_**3a**]$^+$ (Coordination *via* Ketone) S198
- ACN S200
- H_2O S200
- H_2 ...... S200
- **2a** S200
- **3a** S200
- THF S200
- CH_2Cl_2 ...... S200
- Et_2O S201
- EtOH S201
- MeOH S201
- *N*-(4-propylphenyl)-hydroxylamine S201

- **3e** S201

4. XYZ Coordinates of the Optimized Structures | THF S202
- [**23**_RhH_B]$^+$ (**I1**) S202
- [**23**_Rh_ACN_B_ACN]$^{2+}$ S203
- [**23**_RhH_B_ACN]$^+$ S204
- [**23**_RhH_B_THF]$^+$ S205
- [**23**_RhH_B_**3a**]$^+$ (Coordination *via* NHOH/N) S206
- [**23**_RhH_B_**3a**]$^+$ (Coordination *via* NHOH/O) S208
- ACN S209
- H_2O S209
- H_2 .... S209
- **2a** S209
- **3a** S209
- THF S210

X-ray Crystallography and Refinement of Structures S211

1. Molecular Structure of **1·CH_3CN** (CCDC 2110200) S212
2. Molecular Structure of **6** (CCDC 2117074) S243
3. Molecular Structure of **5·THF** (CCDC 2117073) S269

References S286

General Methods

All the reactions were prepared inside an MBraun glovebox under an argon atmosphere. Solvents for air- and moisture-sensitive experiments were purified using a two-column solvent purification system (MBraun-SPS-7), transferred directly to the glovebox, and stored over molecular sieves (3 and 4 Å). Technical grade solvents were used for workup and purification procedures. All reagents were purchased at the highest commercial quality from either abcr, Alfa Aesar or Sigma Aldrich and used without further purification. Conversions and NMR yields were determined spectroscopically by ^{1}H NMR relative to mesitylene as an internal standard. The different products were isolated either by silica gel chromatography using high purity grade silica gel (60 Å, particle size 0.043–0.063 mm) from Sigma Aldrich or by preparative TLC using SIL G-200 UV_{254} (20*20 cm, 2 mm) thick glass plates. All routine solution-state NMR spectra were recorded on a Bruker Ascend 400 spectrometer in the indicated solvents at the given temperatures. The coupling constants (J) are given in Hertz (Hz). The chemical shifts (δ) expressed in ppm are calibrated using residual undeuterated solvent (acetone, $CHCl_3$, CH_2Cl_2, CH_3CN, DMSO at 2.05, 7.26, 5.32, 1.94, 2.50 ppm for ^{1}H NMR, respectively, and 29.8, 77.2, 53.8, 1.3, 39.5 ppm for ^{13}C NMR, respectively). The following abbreviations were used to explain multiplicities: s = singlet, d = doublet, dd = doublet of doublet, t = triplet, q = quartet, tt = triplet of triplet, m = multiplet, bs = broad singlet. Gas chromatography (GC) was performed on a Shimadzu GC2030 equipped with an FID-detector (CP-WAX-52CB column from Agilent). Gas chromatography coupled with a mass spectrometer (GC-MS) was performed on a Shimadzu QP2020. HR-MS spectra were recorded on a Bruker ESQ3000 spectrometer. The melting point was measured on a Stuart^R SMP50 (Automatic Melting point) apparatus at starting temperature of 50 °C with a ramp rate of 2 °C/min. Infrared spectra were recorded on a Thermo Scientific Nicolet™ iS5 Spectrometer with an ID7 ATR accessory. The IR signals were designated as st (strong), m (medium), w (weak).

Caution! *In order to conduct experiments with pressurized hydrogen, appropriate safety procedures should be followed in conjunction with the right equipment. In order to pressurize (or open) Fisher-Porter tubes, protective measures, such as wearing gloves and operating in a fume hood behind a shield, must be used.*

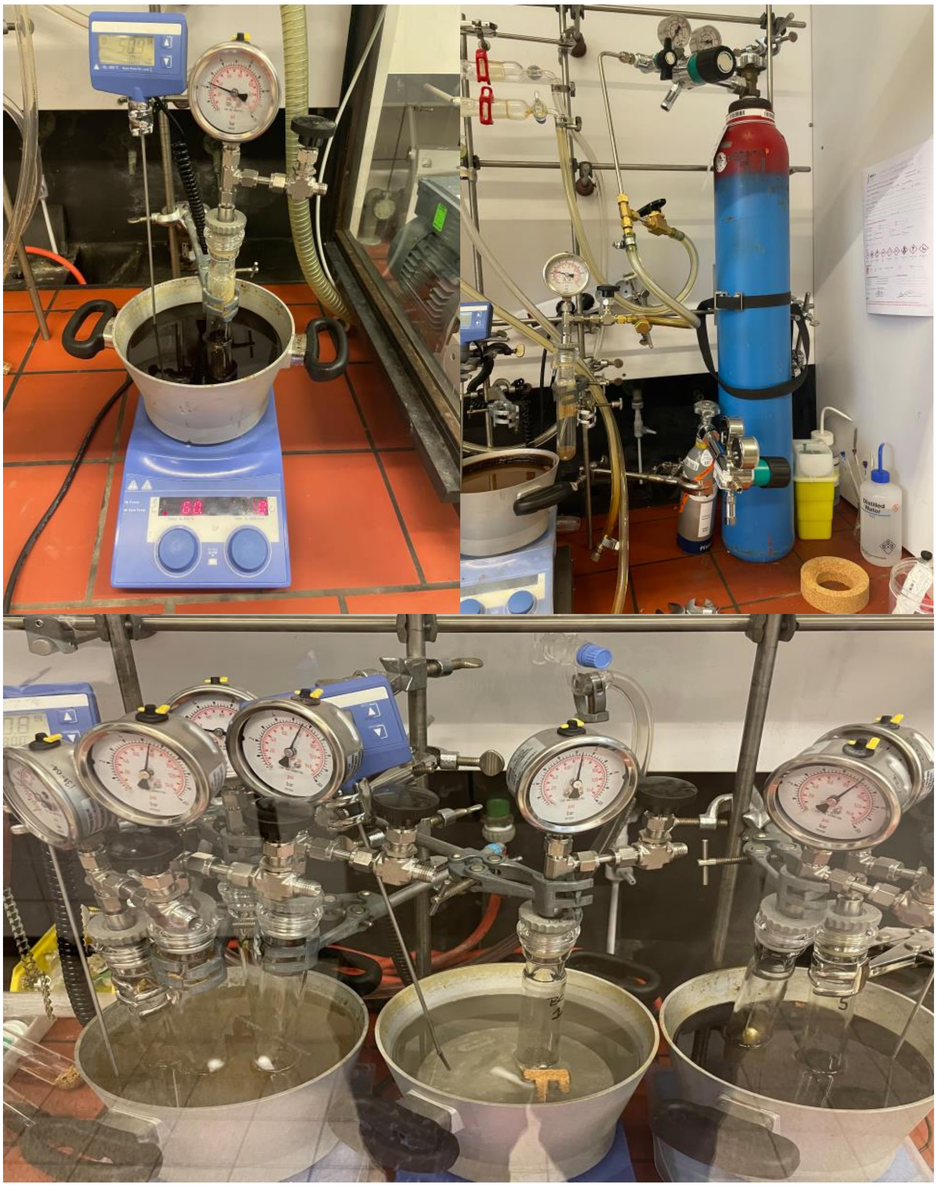

Figure S1 – Reaction setups used for this study: Top left: the reactions were performed in **Fisher-Porter** tubes consisting of a glass vessel equipped with a manometer. Top right: **Hydrogen Cylinder** with a pressure reducer installed in a fume hood. Bottom: The reactions were performed behind a **protective blast shield** inside the fume hood.

Ligand Synthesis and Characterization

1. Synthesis of Triazine 20

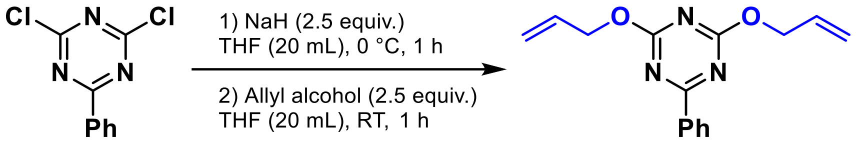

20 (90%)

Procedure:

In a Schlenk flask under an argon atmosphere, sodium hydride (884 mg, 22.1 mmol; 60% dispersion in mineral oil) was washed with pentane (3 × 15 mL) and then dried under vacuum. Dry and degassed THF (20 ml) was added, and the mixture was cooled to 0 °C using an ice bath. Allyl alcohol (1.5 ml, 22.1 mmol) dried over 4 Å molecular sieves before utilization was added dropwise, and the resulting mixture was stirred at 0 °C for 1 hour. 2,4-dichloro-6-phenyl-1,3,5-triazine (2.0 g, 8.9 mmol) was added in 2–3 portions. The mixture was brought to room temperature and stirred for 1 h. The solvent was concentrated *in vacuo*, and water (20 mL) was added to quench the excess NaH. The product was extracted from the water phase with ethyl acetate (2 × 20 mL). The organic layers were combined and dried over $MgSO_4$, and the volatiles were removed *in vacuo* to give **20** as a white solid that was used without further purification (2.2 g, 8.0 mmol, 90%).

^{1}H NMR (500 MHz, $CDCl_3$, 296 K): δ 8.53 – 8.45 (m, 2H), 7.61 – 7.53 (m, 1H), 7.48 (ddd, *J* = 8.1, 6.6, 1.2 Hz, 2H), 6.12 (ddt, *J* = 17.3, 10.4, 5.8 Hz, 2H), 5.47 (dt, *J* = 17.2, 1.5 Hz, 2H), 5.32 (dq, *J* = 10.4, 1.3 Hz, 2H), 5.02 (dt, *J* = 5.8, 1.4 Hz, 4H).

^{13}C{^{1}H} NMR (126.0 MHz, $CDCl_3$, 296 K): δ 175.1, 172.4, 135.2, 133.0, 132.0, 129.2, 128.6, 119.2, 68.9.

HRMS (ESI$^+$) Calcd. (%) for ($C_{15}H_{15}N_3O_2$): 270.12370; Found: 270.12381.

Anal. Calcd. (%) for ($C_{15}H_{15}N_3O_2$): C 66.90, H 5.61, N 15.60; Found: C 67.11, H 5.77, N 15.64.

IR (Diamond-ATR, neat), υ (cm^{-1}): 3070.04 (m), 3036.56 (w), 3018.98 (m), 2992.62 (m), 2926.71 (w), 2878.80 (w), 1589.16 (m), 1548.51 (st), 1540.86 (st), 1475.71 (m), 1459.38 (m), 1445.55 (w), 1389.29 (st), 1347.54 (st), 1327.39 (st), 1306.53 (st), 1291.00 (m), 1223.52 (m), 1126.33 (w), 1070.36 (w), 996.19 (m), 931.61 (w), 909.14 (st), 850.02 (w), 788.61 (st), 707.35 (st), 684.77 (st), 646.90 (m), 557.20 (m).

Melting Point: 67.2 °C.

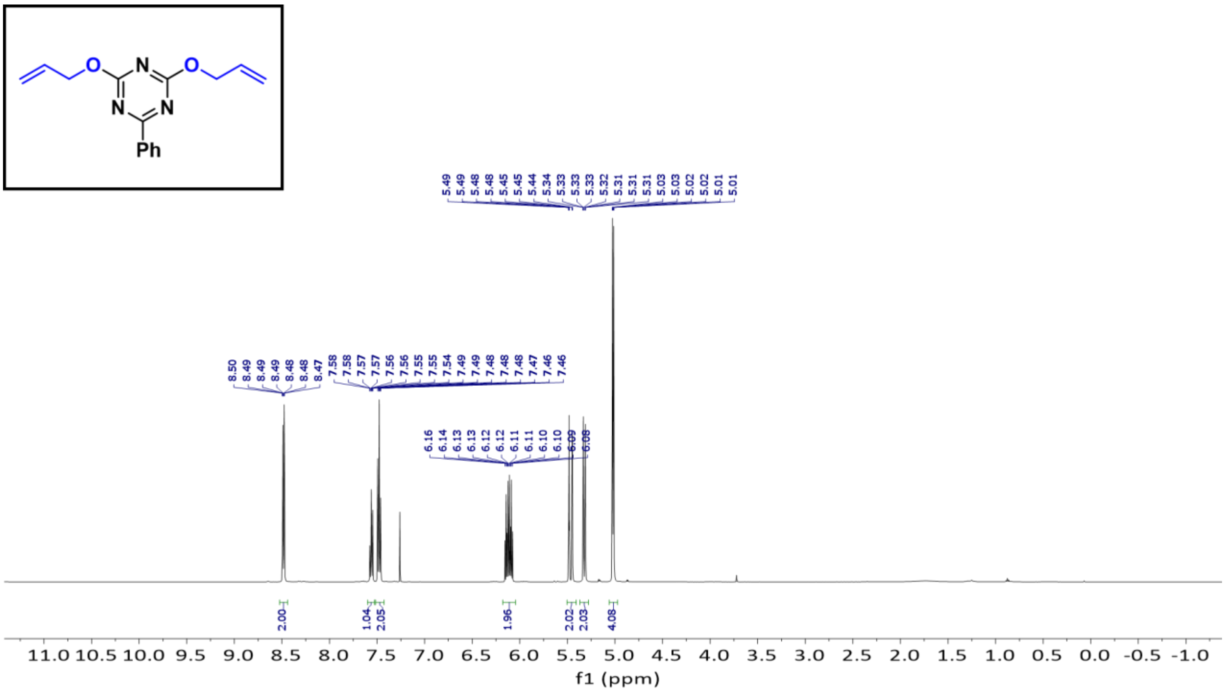

Figure S2 – ^{1}H NMR (500 MHz, $CDCl_3$, 296 K) spectrum of compound **20**.

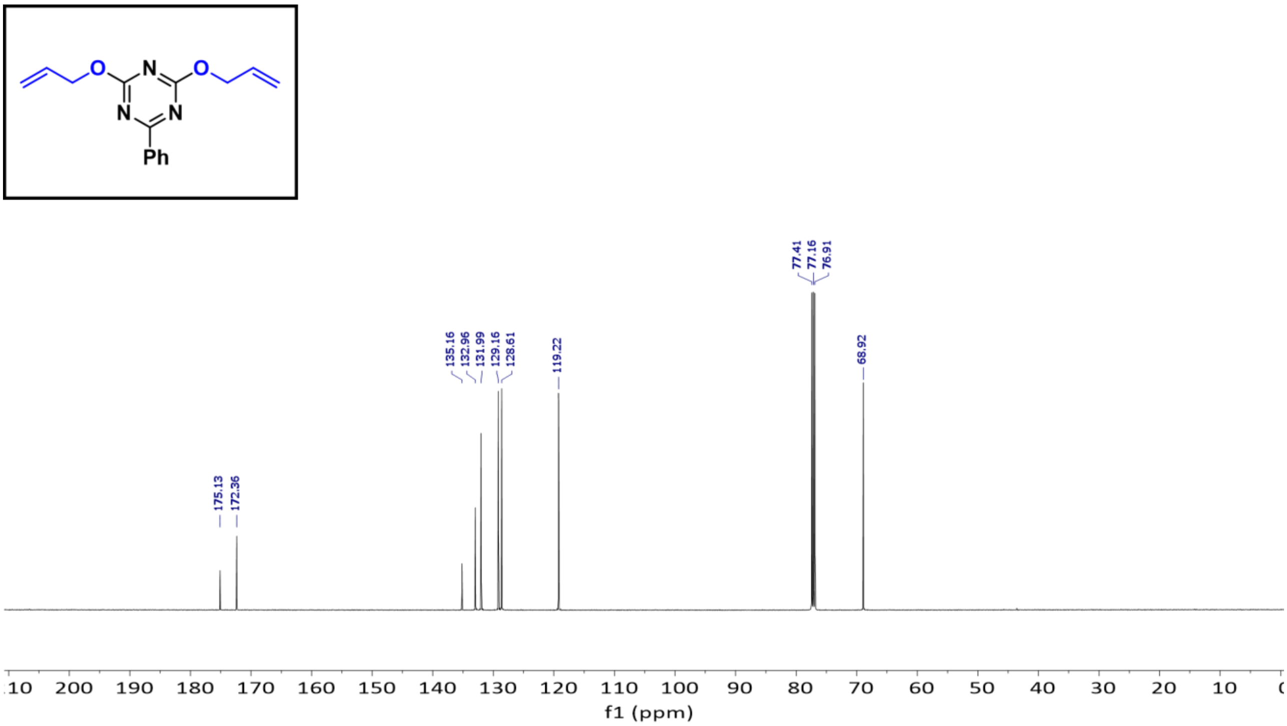

Figure S3 – $^{13}C\{^{1}H\}$ NMR (126 MHz, $CDCl_3$, 296 K) spectrum of compound **20**.

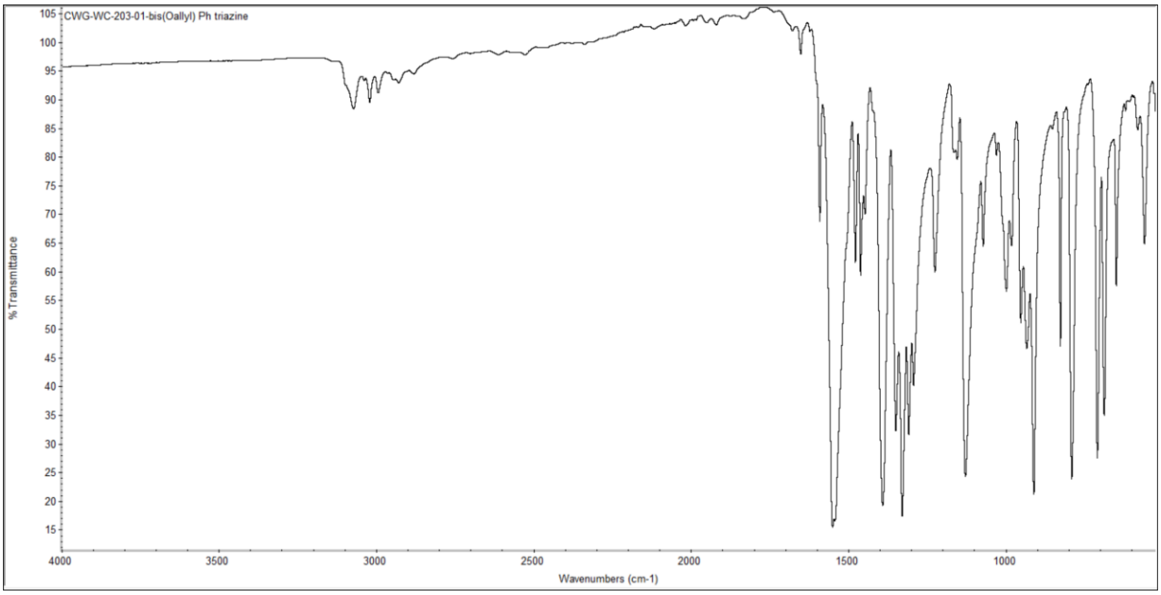

Figure S4 – IR-spectrum of compound **20**.

2. Synthesis of Triazine 21

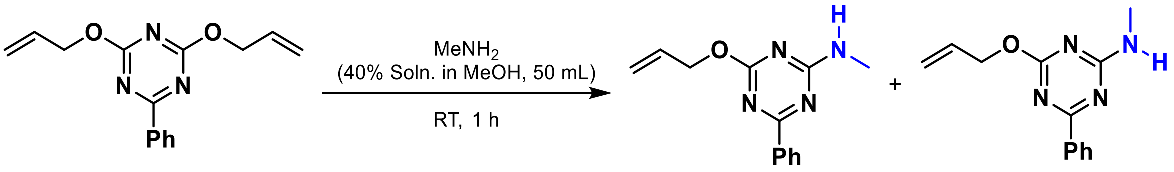

Procedure: In a round bottom Schlenk flask under an argon atmosphere, methylamine (40% solution in methanol) was added in excess (~50 ml) at room temperature to compound **20** (2.2 g, 8.0 mmol). The reaction mixture was stirred until completion (ca. 1 hour; monitored by TLC). Methanol and methylamine were removed *in vacuo* to give **21** as a fluffy white solid that was used without further purification (2.1 g, 8.0 mmol, >99%).

^{1}H NMR (400 MHz, $CDCl_3$, 296 K): δ 8.51 – 8.44 (m, 1H), 8.40 – 8.33 (m, 2H), 7.51 (ddd, *J* = 7.2, 4.7, 2.1 Hz, 2H), 7.46 (dt, *J* = 8.7, 5.9 Hz, 4H), 6.12 (dddd, *J* = 21.7, 15.9, 10.3, 5.5 Hz, 2H), 5.84 (d, *J* = 6.1 Hz, 1H), 5.72 – 5.67 (m, 1H), 5.50 – 5.38 (m, 2H), 5.33 – 5.24 (m, 2H), 5.03 – 4.91 (m, 4H), 3.12 (d, *J* = 5.0 Hz, 2H), 3.03 (d, *J* = 5.0 Hz, 3H).

$^{13}C\{^1H\}$ NMR (101 MHz, $CDCl_3$, 296 K): δ 173.2, 172.6, 171.2, 170.7, 168.4, 168.1, 136.4, 136.2, 132.8, 132.1, 132.0, 128.9, 128.6, 128.4, 128.4, 118.7, 118.3, 77.5, 67.9, 28.1, 27.9.

HRMS (ESI$^+$) Calcd. (%) for ($C_{13}H_{14}N_4O$): 243.12403; Found: 243.12418.

Anal. Calcd. (%) for ($C_{13}H_{14}N_4O$): C 64.45, H 5.82, N 23.13; Found: C 64.64, H 5.90, N 23.13.

IR (Diamond-ATR, neat), υ (cm^{-1}): 3260.26 (st), 3133.26 (st), 3020.80 (m), 2966.54 (m), 2903.00 (w), 1625.51 (st), 1588.70 (w), 1533.26 (st), 1432.12 (m), 1395.40 (m), 1338.66 (st), 1326.04 (st), 1302.87 (m), 1239.37 (st), 1163.57 (st), 1113.94 (m), 1045.45 (m), 1024.36 (w), 993.06 (m), 952.73 (w), 938.74 (m), 920.04 (m), 900.01 (m), 823.31 (st), 778.99 (st), 702.84 (st), 681.20 (m), 650.87 (m), 622.48 (m), 584.24 (w), 548.69 (w).

Melting Point: 131.1 °C.

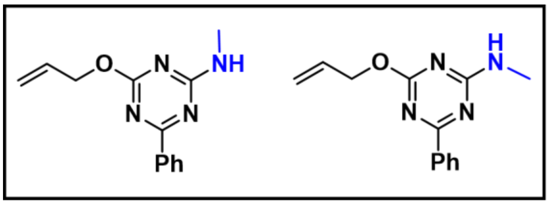

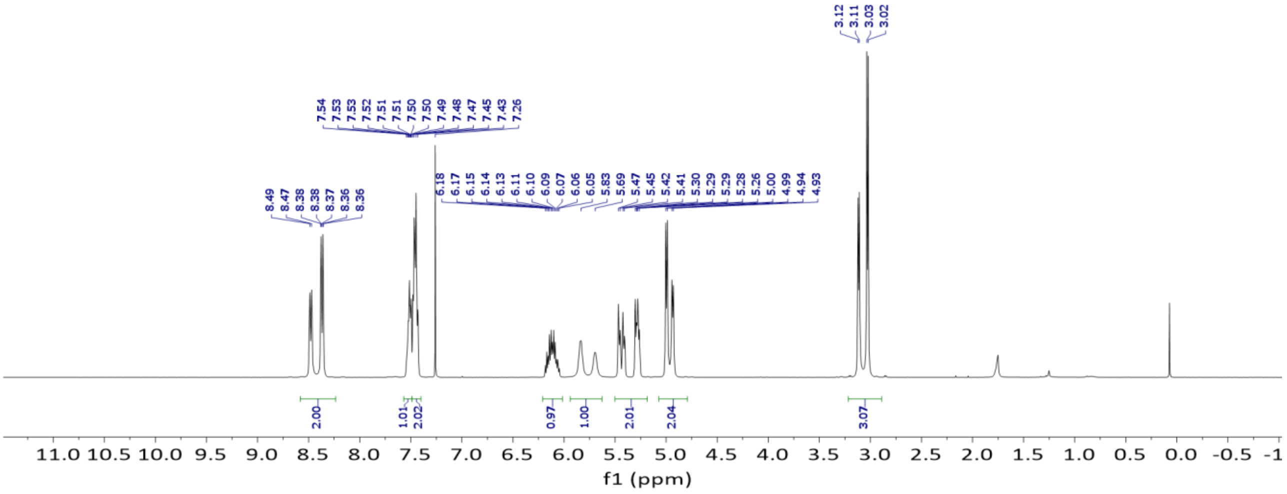

Figure S5 – ^{1}H NMR (400 MHz, $CDCl_3$, 296 K) spectrum of compound **21**.

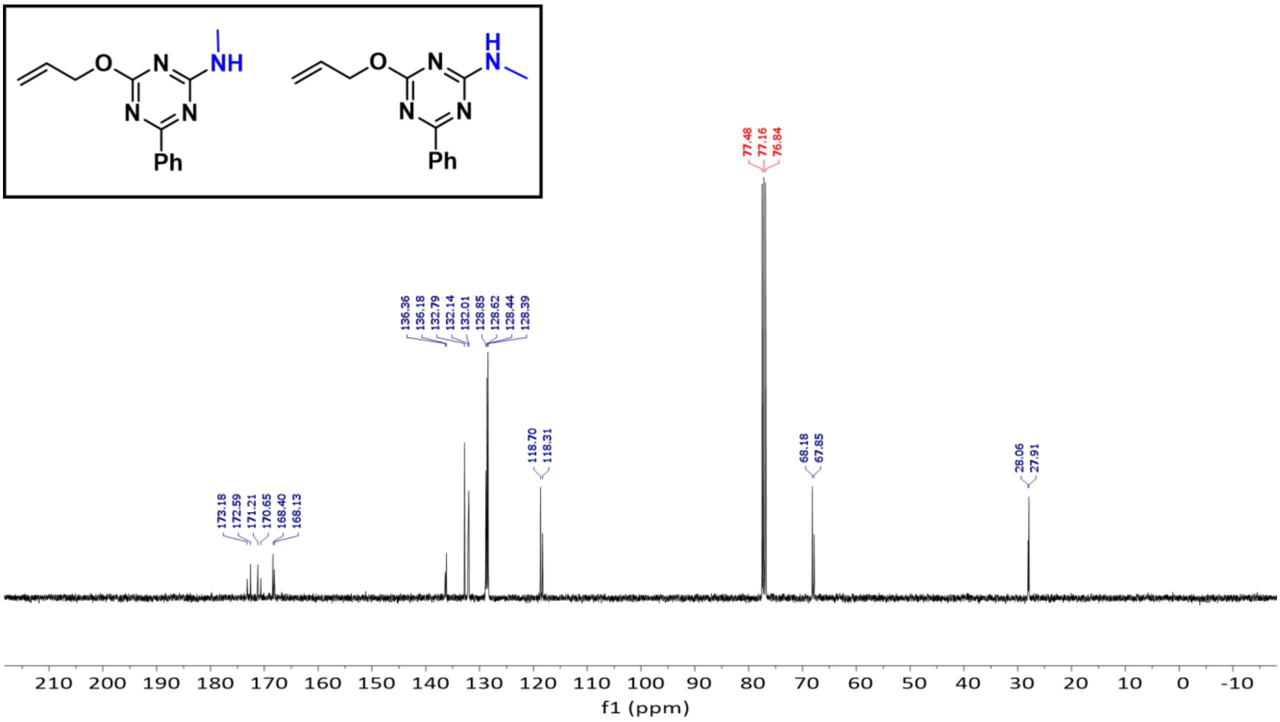

Figure S6 – $^{13}C\{^1H\}$ NMR (101 MHz, $CDCl_3$, 296 K) spectrum of compound **21**.

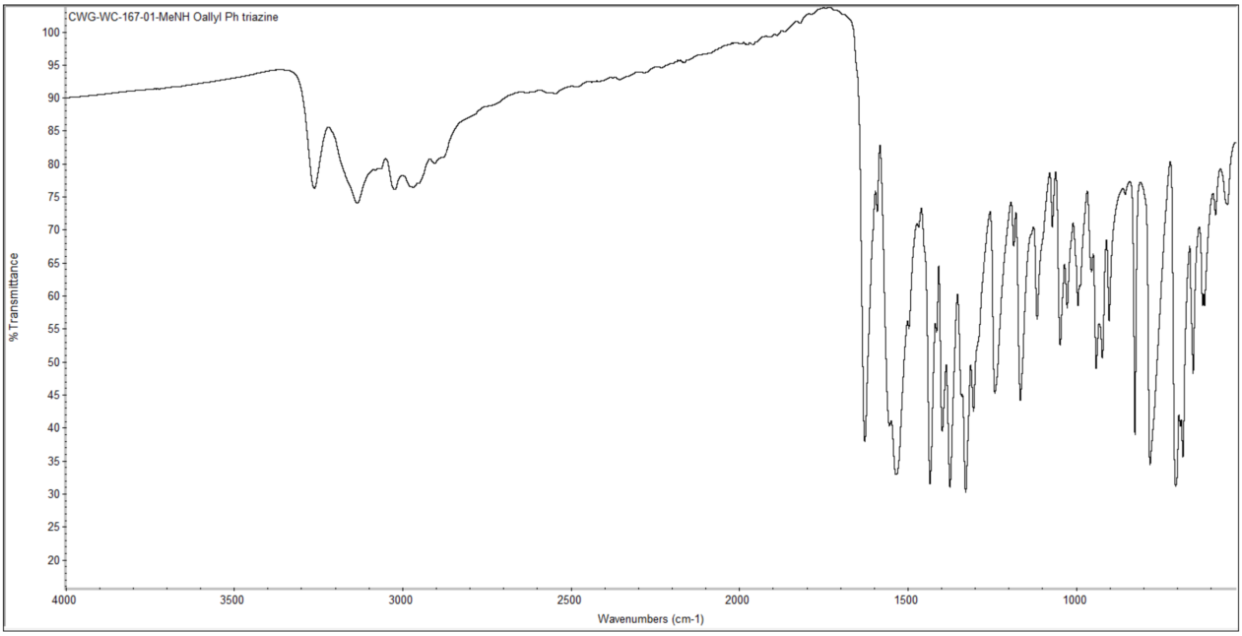

Figure S7 – IR spectrum of compound **21**.

3. Synthesis of Triazine 22

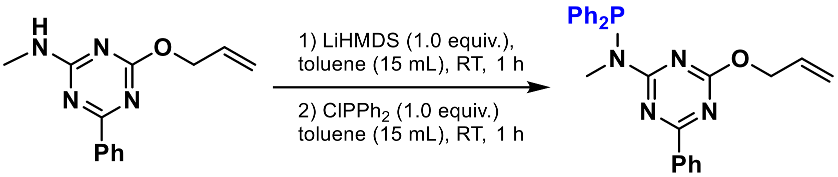

21 **22** (92%)

Procedure: In a Schlenk flask, compound **21** (1.0 g, 3.9 mmol) was solubilized in toluene (15 mL), and LiHMDS (652.5 mg, 3.9 mmol) was added at room temperature. The reaction mixture was stirred for 1 h, which resulted in the formation of a viscous liquid. Chlorodiphenylphosphine (0.7 ml, 3.9 mmol) was added dropwise over the course of 10 min at room temperature. The resulting mixture was stirred for an additional 1 hour at room temperature. After completion, the reaction mixture was filtered through a small pad of celite into another Schlenk flask under an argon atmosphere. The filtrate was dried under reduced pressure to give **22** as an off-white viscous material that was used without further purification (1.5 g, 3.6 mmol, 92%).

^{1}H NMR (500 MHz, C_6D_6, 296 K): δ 8.94 – 8.71 (m, 2H), 7.39 (td, *J* = 6.9, 6.3, 2.3 Hz, 4H), 7.21 (d, *J* = 5.7 Hz, 3H), 7.13 – 7.04 (m, 6H), 5.90 (tt, *J* = 14.5, 6.2 Hz, 1H), 5.27 (d, *J* = 17.2 Hz, 1H), 5.00 (d, *J* = 10.5 Hz, 1H), 4.78 (dt, *J* = 5.6, 1.5 Hz, 2H), 2.99 (s, 3H).

^{13}C{^{1}H} NMR (126 MHz, C_6D_6, 296 K): δ 171.5, 171.3, 137.3, 137.2, 136.9, 133.1, 132.7, 132.5, 132.3, 129.4, 128.9, 128.9, 128.6, 128.4, 117.9, 68.0, 33.6, 33.5.

^{31}P{^{1}H} NMR (202 MHz, C_6D_6, 296 K): δ 51.18 (s).

HRMS (ESI^{+}) Calcd. (%) for ($C_{25}H_{23}N_4OP$): 427.16822; Found: 427.16837.

Anal. Calcd. (%) for ($C_{25}H_{23}N_4OP$): C 70.41, H 5.44, N 13.14; Found: C 70.38, H 5.40, N 12.97.

IR (Diamond-ATR, neat), υ (cm^{-1}): 3068.31 (st), 3054.15 (st), 3030.72 (st), 3013.49 (st), 3001.80 (st), 2984.29 (st), 2937.55 (st), 1588.52 (m), 1543.31 (w), 1518.05 (w), 1504.97 (w), 1485.05 (w), 1448.24 (m), 1433.45 (m), 1412.33 (m), 1371.11 (w), 1326.18 (w), 1305.03 (m), 1288.03 (m), 1225.93 (m), 1199.72 (st), 1160.79 (m), 1092.02 (m), 1049.69 (m), 1025.56 (m), 981.56 (m), 925.08 (m), 883.60 (st), 847.77 (st), 827.36 (m), 782.84 (m), 763.44 (w), 694.45 (w), 654.24 (m), 617.49 (st), 540.98 (st).

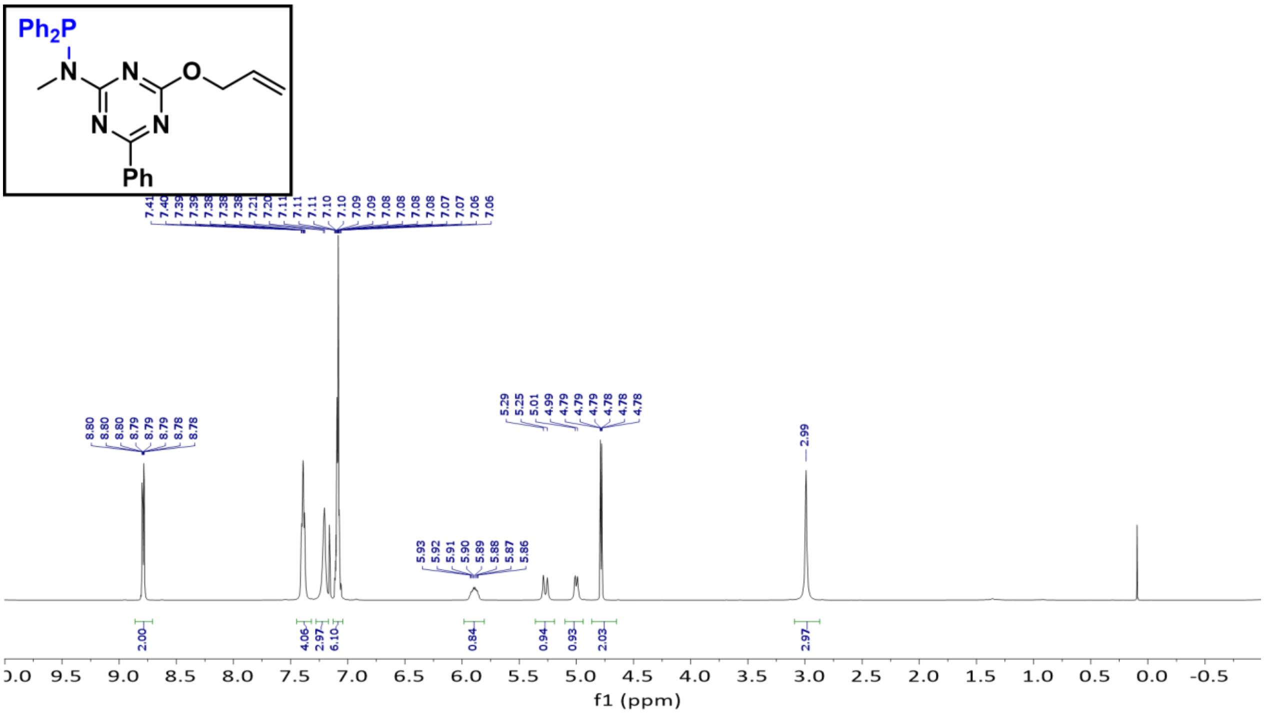

Figure S8 – ¹H NMR (500 MHz, C_6D_6, 296 K) spectrum of compound **22**.

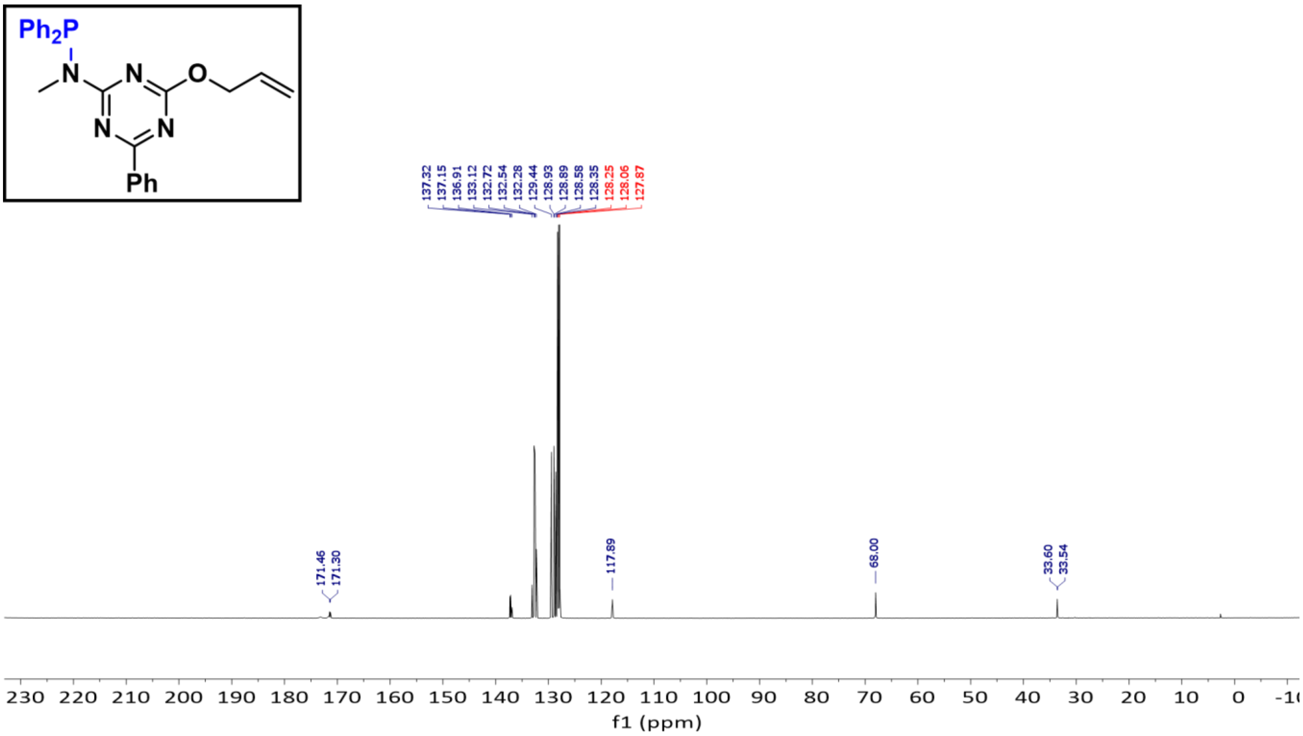

Figure S9 – $^{13}C\{^1H\}$ NMR (126 MHz, C_6D_6, 296 K) spectrum of compound **22**.

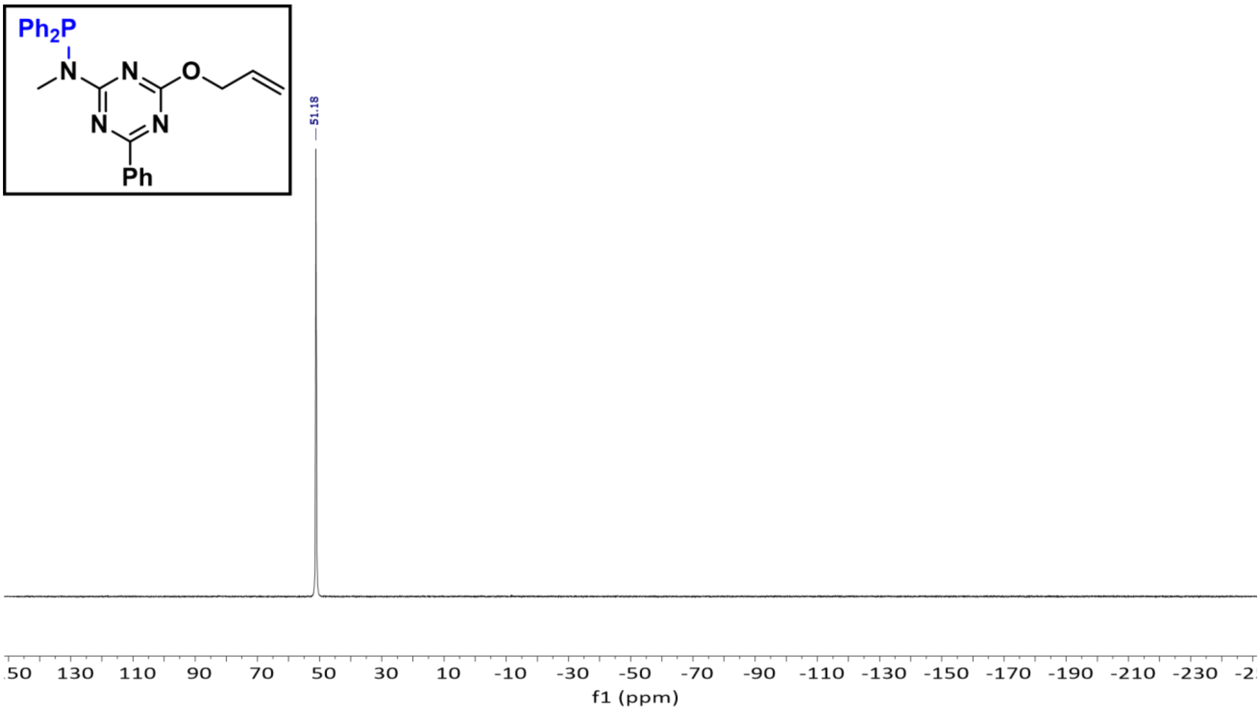

Figure S10 – $^{31}P\{^1H\}$ NMR (202 MHz, C_6D_6, 296 K) spectrum of compound **22**.

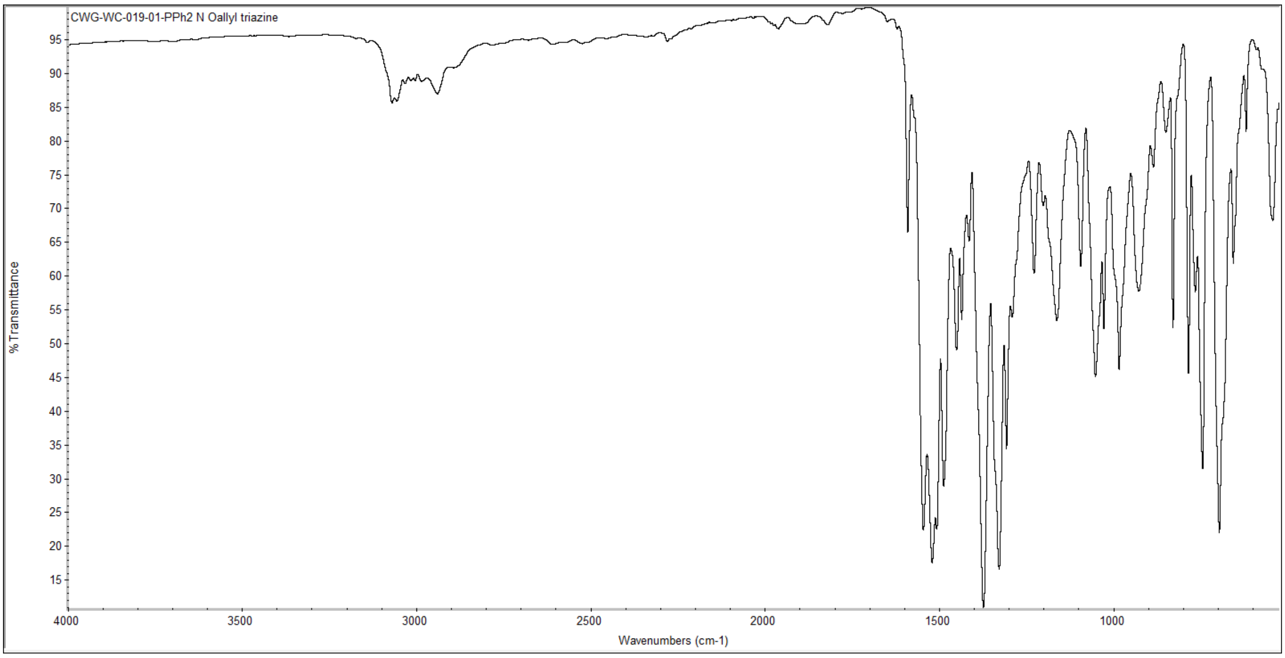

Figure S11 – IR-spectrum of compound **22**.

4. Synthesis of Triazine 23

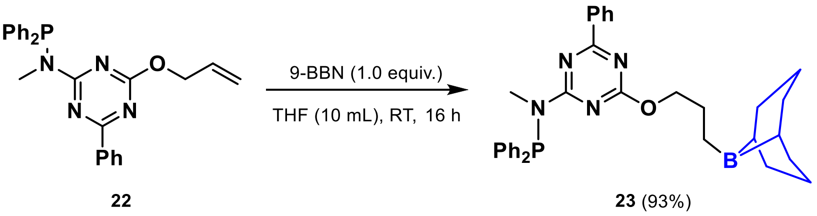

Procedure: 9-Borabicyclo[3.3.1]nonane solution (0.5 M in THF; 0.7 ml, 3.6 mmol) was added at room temperature to a THF (10 mL) solution of **22** (1.5 g, 3.6 mmol). After completion of the reaction (ca. 16 h; monitored by ^{1}H NMR spectroscopy), the volatiles were removed *in vacuo* to give **23** as an off-white viscous substance that was used directly without further purification (2.0 g, 3.3 mmol, 93%).

^{1}H NMR (500 MHz, C_6D_6, 296 K): δ 8.84 (dd, *J* = 7.8, 2.0 Hz, 2H), 7.44 – 7.34 (m, 4H), 7.21 (d, *J* = 6.9 Hz, 3H), 7.12 – 7.03 (m, 6H), 4.40 (t, *J* = 6.6 Hz, 2H), 3.03 (d, *J* = 1.2 Hz, 3H), 2.00 – 1.78 (m, 8H), 1.76 – 1.59 (m, 6H), 1.39 (q, *J* = 7.8, 7.3 Hz, 2H), 1.22 (dtd, *J* = 17.3, 8.6, 7.7, 4.5 Hz, 2H).

^{13}C{^{1}H} NMR (126 MHz, C_6D_6, 296 K): δ 173.2, 172.1, 171.6, 171.4, 137.5, 137.3, 137.1, 132.8, 132.5, 132.2, 129.4, 129.4, 69.8, 33.6, 33.5, 33.5, 31.4, 24.5, 24.3, 23.7.

^{31}P{^{1}H} NMR (202 MHz, C_6D_6, 296 K): δ 50.94 (s).

HRMS (ESI^{+}) Calcd. (%) for ($C_{33}H_{38}BN_4OP$): 549.29490; Found: 549.29503.

Anal. Calcd. (%) for ($C_{33}H_{38}BN_4OP$ + 0.3 CH_2Cl_2): C 69.69, H 6.78, N 9.76; Found: C 69.41, H 6.82, N 9.68.

IR (Diamond-ATR, neat), υ (cm^{-1}): 3053.27 (st), 2884.58 (st), 2836.98 (st), 1588.56 (st), 1544.50 (m), 1521.62 (m), 1486.89 (m), 1446.90 (m), 1433.74 (m), 1419.29 (m), 1375.11 (w), 1346.34 (m), 1330.26 (m), 1305.50 (m), 1263.85 (m), 1227.15 (st), 1160.85 (st), 1093.04 (st), 1050.01 (m), 1025.75 (m), 982.36 (m), 918.60 (st), 828.33 (st), 784.16 (m), 737.17 (m), 696.67 (m), 654.95 (st), 544.60 (st).

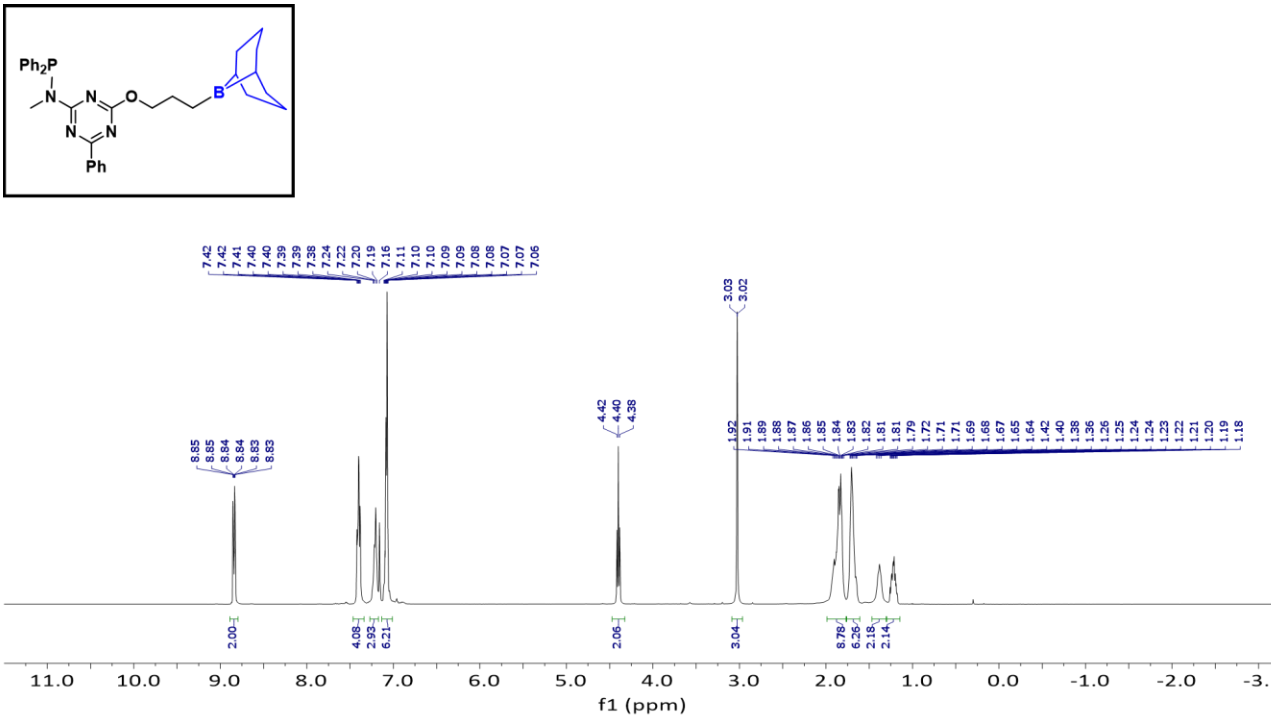

Figure S12 – ^{1}H NMR (500 MHz, C_6D_6, 296 K) spectrum of compound **23**.

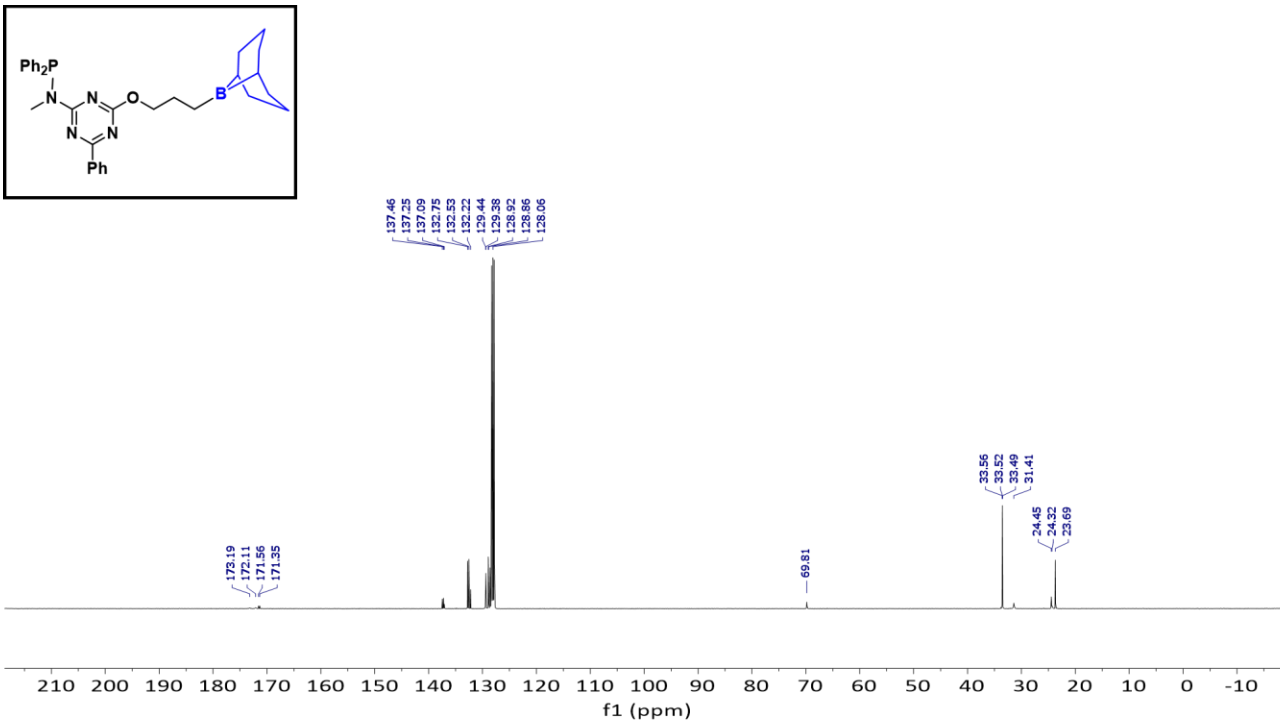

Figure S13 – $^{13}C\{^{1}H\}$ NMR (126 MHz, C_6D_6, 296 K) spectrum of compound **23**.

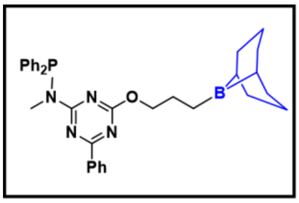

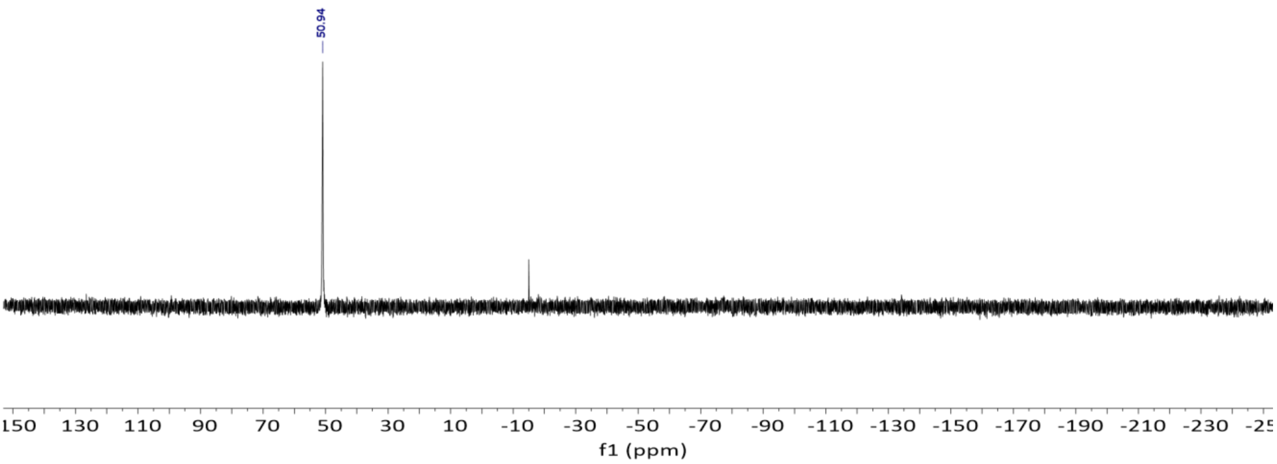

Figure S14 – $^{31}P\{^{1}H\}$ NMR (202 MHz, C_6D_6, 296 K) spectrum of compound **23**.

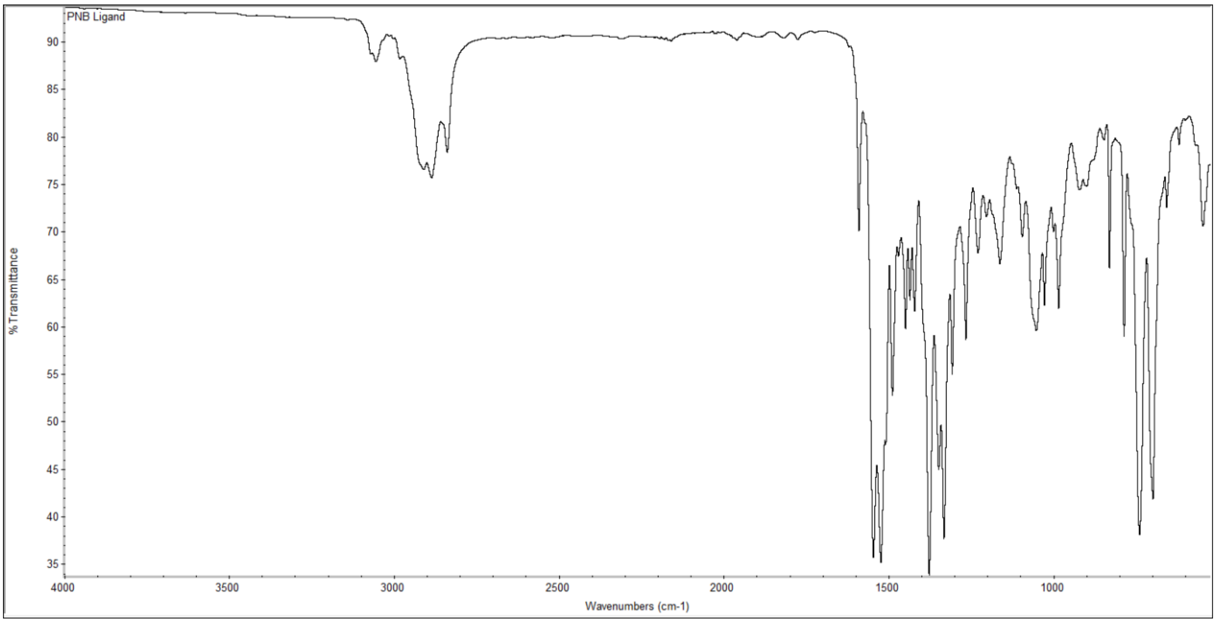

Figure S15 – IR-spectrum of compound **23**.

5. Synthesis of Triazine 24

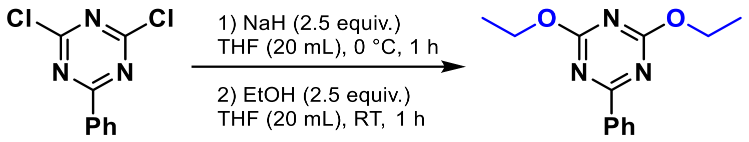

24 (94%)

Procedure: In a Schlenk flask under argon atmosphere, sodium hydride (884 mg, 22.1 mmol; 60% dispersion in mineral oil) was washed with pentane (3 × 15 mL) and then dried under vacuum. Dry and degassed THF (20 ml) was added, and the flask was cooled to 0 °C using an ice bath. Dry ethanol (1.3 mL, 22.1 mmol) was added dropwise to the solution, and the resulting mixture was stirred for 1 hour at 0 °C. 2,4-dichloro-6-phenyl-1,3,5-triazine (2.0 g, 8.9 mmol) was added in 2-3 portions. The resulting mixture was warmed to room temperature and stirred for 1 h. The solvent was concentrated *in vacuo*, and water (20 mL) was added to quench the excess sodium hydride. Extraction of the product from the water phase was performed using ethyl acetate (2*~20 mL). The organic layers were combined and dried over $MgSO_4$, and the volatiles were removed *in vacuo* to provide **24** as a white solid that was used without further purification (2.1 g, 8.4 mmol, 94%).

^{1}H NMR (500 MHz, $CDCl_3$, 296 K): δ 8.50 – 8.45 (m, 2H), 7.57 – 7.52 (m, 1H), 7.49 – 7.43 (m, 2H), 4.56 (q, *J* = 7.1 Hz, 4H), 1.47 (t, *J* = 7.1 Hz, 6H).

^{13}C{^{1}H} NMR (126 MHz, $CDCl_3$, 296 K): δ 174.9, 172.5, 135.3, 132.8, 129.1, 128.6, 64.3, 14.5.

HRMS (ESI$^+$) Calcd. (%) for ($C_{13}H_{15}N_3O_2$): 246.12370; Found: 246.12388.

Anal. Calcd. (%) for ($C_{13}H_{15}N_3O_2$): C 63.66, H 6.16, N 17.13; Found: C 63.46, H 6.21, N 17.01.

IR (Diamond-ATR, neat), υ (cm^{-1}): 3058.92 (st), 2984.64 (st), 2936.45 (st), 2873.78 (st), 1589.13 (st), 1533.27 (w), 1488.48 (m), 1478.58 (m), 1461.98 (m), 1439.26 (st), 1413.47 (m), 1390.71 (m), 1374.75 (w), 1343.48 (w), 1334.90 (w), 1325.35 (w), 1305.66 (w), 1223.13 (m), 1185.79 (st), 1151.73 (m), 1133.35 (w), 1097.24 (m), 1068.74 (m), 1039.04 (m), 1024.06 (m), 1024.06 (m), 1012.86 (m), 1001.12 (m), 986.09 (m), 945.23 (m), 878.27 (st), 860.94 (st), 828.99 (m), 819.03 (st), 787.17 (w), 751.97 (st), 726.62 (st), 710.51 (w), 685.69 (m), 653.19 (m), 638.88 (st), 613.40 (st), 590.81 (st).

Melting Point: 74.9 °C.

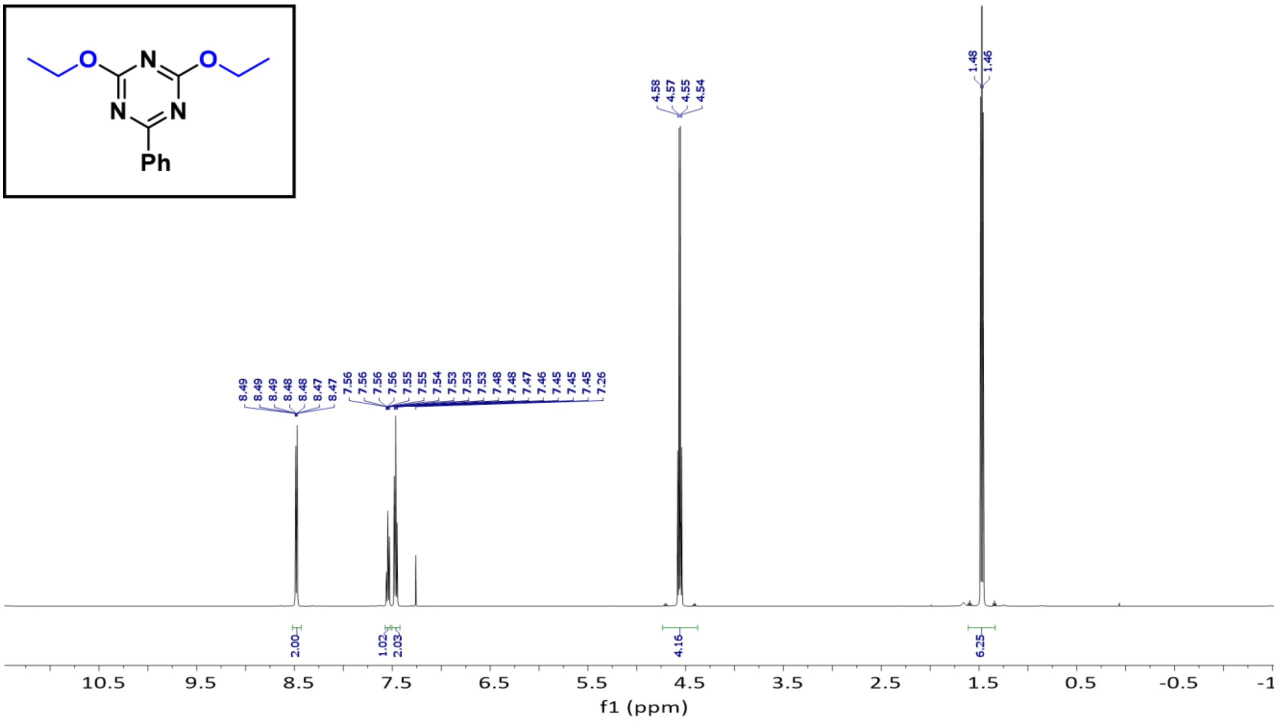

Figure S16 – ^{1}H NMR (500 MHz, $CDCl_3$, 296 K) spectrum of compound **24**.

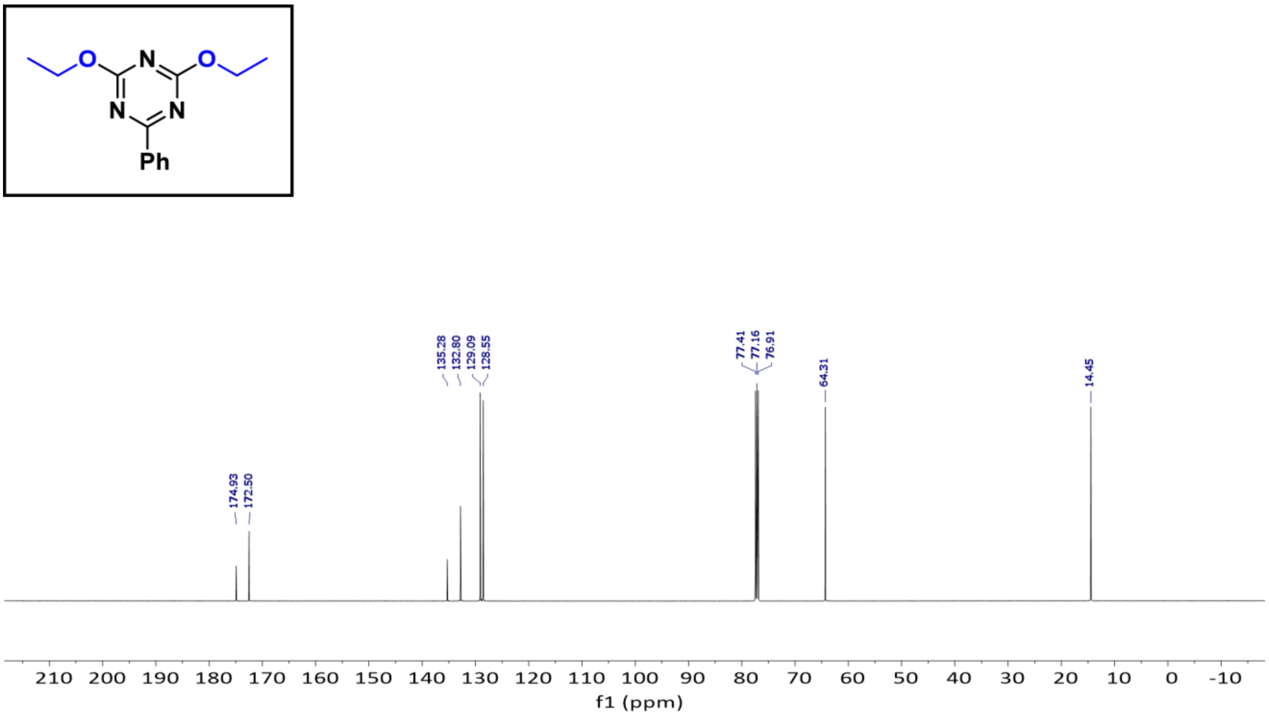

Figure S17 – $^{13}C\{^{1}H\}$ NMR (126 MHz, $CDCl_3$, 296 K) spectrum of compound **24**.

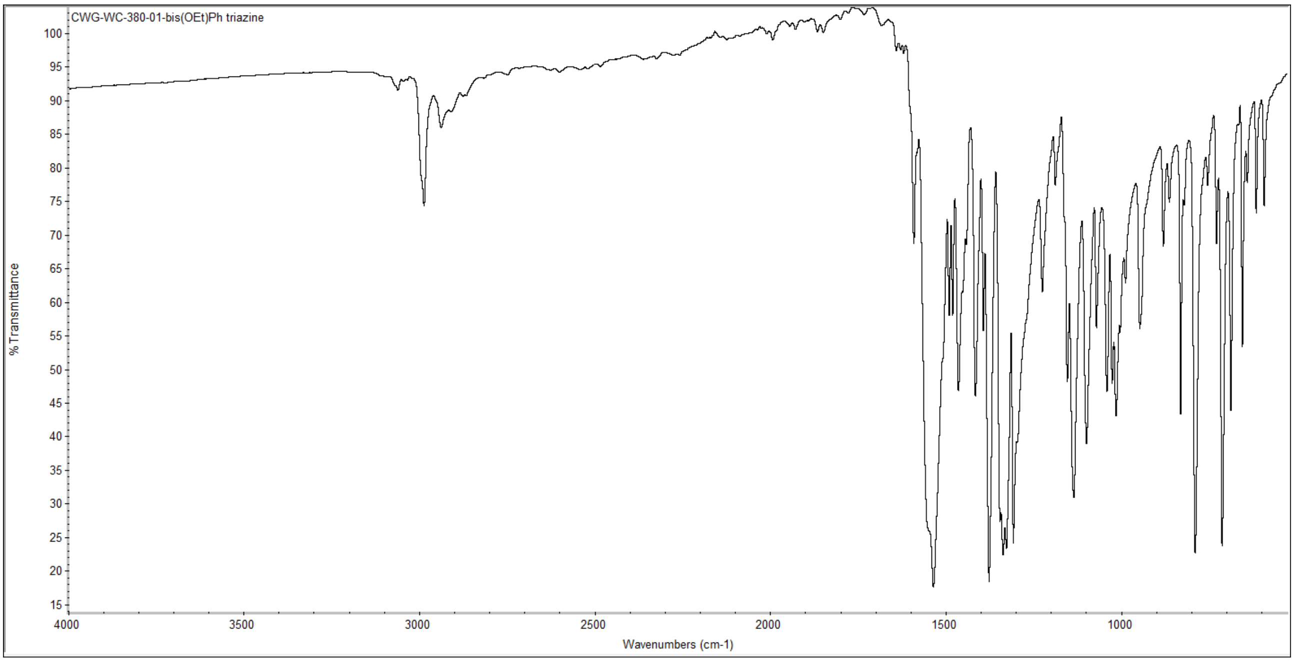

Figure S18 – IR-spectrum of compound **24**.

6. Synthesis of Triazine 25

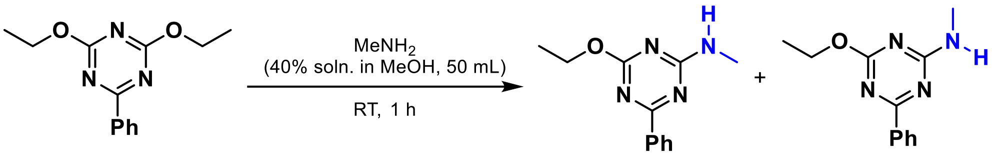

24 **25a** **25b**

25 = **25a** + **25b** (>99%)

Procedure: In a round bottom Schlenk flask under an argon atmosphere, methylamine (40% solution in methanol) was added in excess (~50 ml) at room temperature to compound **24** (2.1 g, 8.4 mmol). The reaction mixture was stirred until completion (ca. 1 h; monitored by TLC). Methanol and methylamine were removed *in vacuo* to give **25** as a fluffy white solid that was used without further purification (1.9 g, 8.4 mmol, >99%).

^{1}H NMR (400 MHz, $CDCl_3$, 296 K): δ 8.50 – 8.45 (m, 2H), 8.39 – 8.34 (m, 3H), 7.54 – 7.48 (m, 3H), 7.48 – 7.41 (m, 6H), 6.14 (s, 1H), 5.94 – 5.90 (m, 1H), 4.53 (q, *J* = 7.1 Hz, 3H), 4.46 (q, *J* = 7.1 Hz, 2H), 3.08 (d, *J* = 5.0 Hz, 3H), 3.02 – 2.97 (m, 4H), 1.44 (dt, *J* = 15.2, 7.1 Hz, 8H).

$^{13}C\{^{1}H\}$. NMR (101 MHz, CDCl$_3$, 296 K): δ 173.1, 172.4, 171.3, 170.7, 168.2, 168.1, 136.4, 136.1, 132.0, 131.9, 128.8, 128.6, 128.4, 128.3, 77.4, 77.2, 76.9, 63.4, 63.1, 28.0, 27.8, 14.6.

HRMS (ESI$^+$) Calcd. (%) for ($C_{12}H_{14}N_4O$): 231.12404; Found: 231.12430.

Anal. Calcd. (%) for ($C_{12}H_{14}N_4O$): C 62.59, H 6.13, N 24.33; Found: C 62.48, H 6.19, N 24.25.

IR (Diamond-ATR, neat), υ (cm^{-1}): 3259.69 (st), 3131.43 (st), 3131.43 (st), 3066.94 (st), 3056.65 (st), 3024.11 (st), 2974.70 (st), 2939.42 (st), 2927.40 (st), 2873.05 (m), 1588.84 (m), 1558.82 (w), 1530.48 (m), 1497.58 (m), 1476.57 (m), 1451.54 (m), 1433.47 (m), 1401.18 (m), 1417.80 (m), 1377.41 (w), 1332.71 (w), 1305.76 (m), 1237.91 (m), 1185.37 (m), 1166.67 (m), 1157.84 (m), 1119.63 (m), 1107.89 (m), 1055.99 (m), 1027.50 (m), 1002.06 (st), 986.33 (st), 878.15 (m), 854.36 (m), 824.04 (st), 783.10 (w), 706.17 (w), 692.72 (m), 683.32 (m), 644.61 (m), 617.17 (m), 559.57 (m).

Melting Point: 142.0 °C.

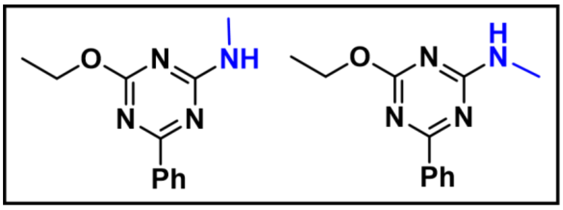

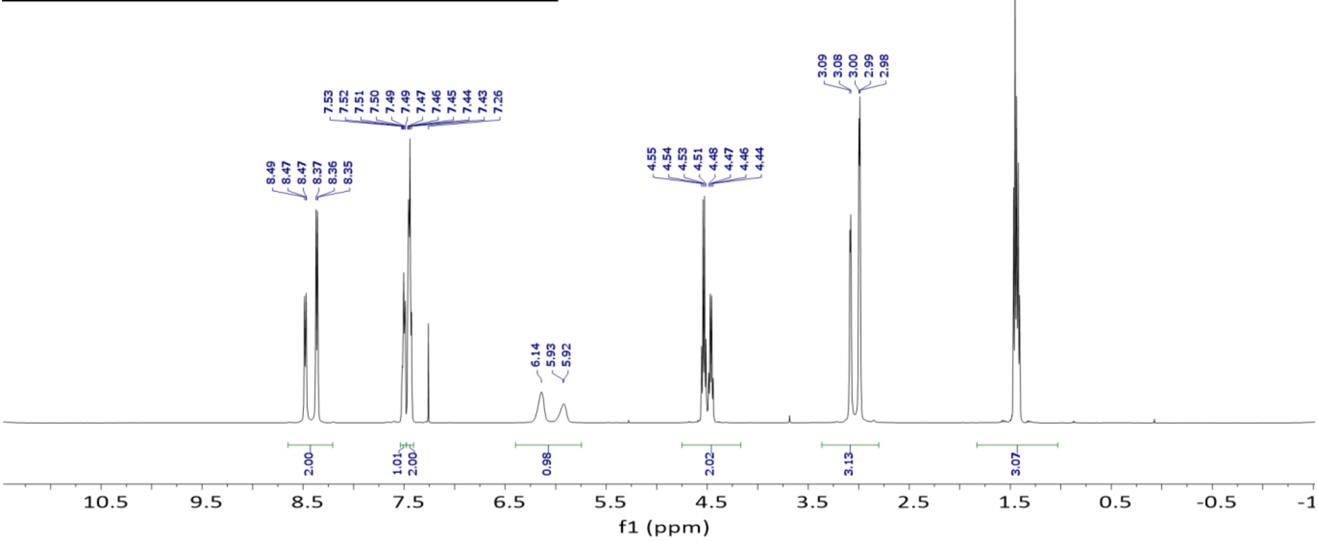

Figure S19 – ^{1}H NMR (400 MHz, CDCl$_3$, 296 K) spectrum of compound **25**.

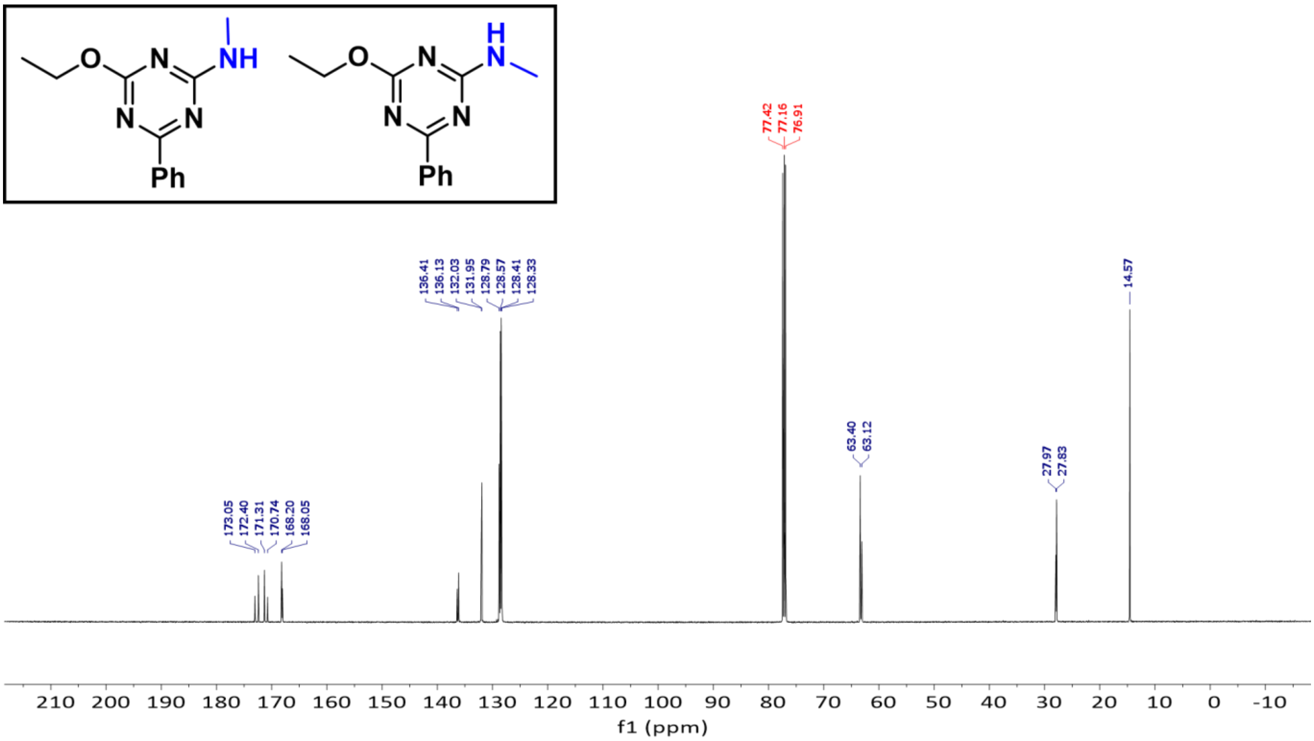

Figure S20 – $^{13}C\{^{1}H\}$ NMR (101 MHz, $CDCl_3$, 296 K) spectrum of compound **25**.

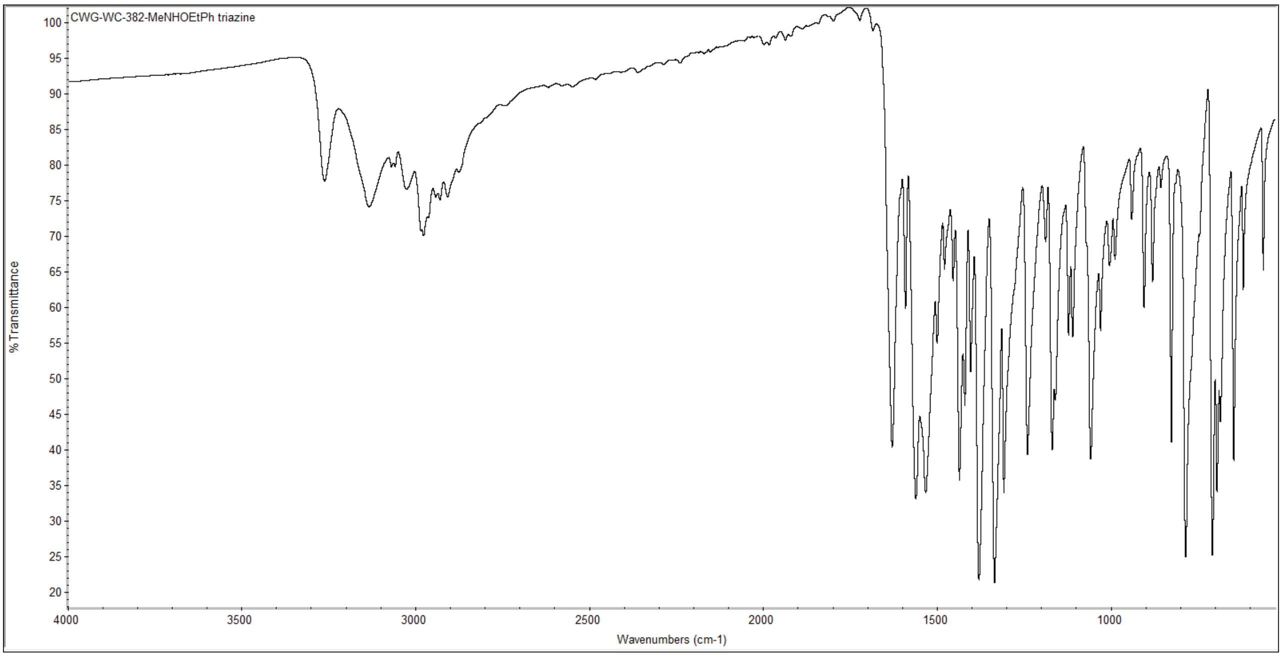

Figure S21 – IR-spectrum of compound **25**.

7. Synthesis of Triazine 26

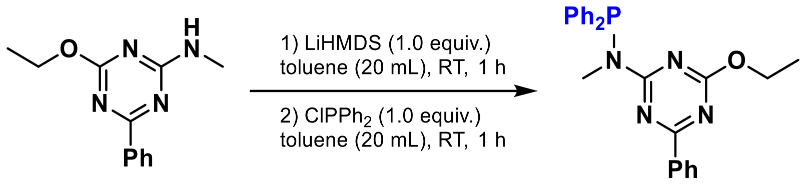

25 **26** (89%)

Procedure: In a Schlenk flask, compound **25** (1.9 g, 8.4 mmol) was solubilized in toluene, and LiHMDS (1.4 g, 8.4 mmol) was added at room temperature. The reaction mixture was stirred for 1 hour, which resulted in the formation of a viscous liquid forming. Chlorodiphenylphosphine (1.5 mL, 8.4 mmol) was added dropwise over the course of 10 min at room temperature. The resulting mixture was stirred for an additional 1 hour at room temperature. After completion, the reaction mixture was filtered through a small pad of celite into another Schlenk flask under an argon atmosphere. The resulting filtrate was dried under reduced pressure to give **26** as an off-white viscous material that was used without further purification (3.1 g, 7.5 mmol, 89%).

^{1}H NMR (400 MHz, CD_2Cl_2, 296 K): δ 8.45 (d, J = 7.6 Hz, 2H), 7.76 – 6.94 (m, 13H), 4.50 (q, J = 7.3 Hz, 2H), 3.03 (s, 3H), 1.42 (t, J = 7.1 Hz, 3H).

^{13}C{^{1}H} NMR (101 MHz, CD_2Cl_2, 296 K): δ 171.3, 171.1, 137.2, 137.0, 136.8, 132.9, 132.7, 132.6, 129.9, 129.2, 129.1, 128.8, 64.0, 34.0, 33.9, 14.8.

^{31}P{^{1}H} NMR (162 MHz, CD_2Cl_2, 296 K): δ 51.22.

HRMS (ESI$^+$) Calcd. (%) for ($C_{24}H_{23}N_4OP$): 415.16822; Found: 415.16830.

Anal. Calcd. (%) for ($C_{24}H_{23}N_4OP$ + 0.1 Toluene): C 70.03, H 5.66, N 13.22; Found: C 70.21, H 5.57, N 13.42.

IR (Diamond-ATR, neat), υ (cm^{-1}): 3061.09 (st), 3030.65 (st), 2999.92 (st), 2976.53 (st), 2935.31 (st), 2897.59 (st), 1588.50 (st), 1572.13 (st), 1520.77 (st), 1505.31 (w), 1488.57 (w), 1463.11 (w), 1442.79 (m), 1433.67 (m), 1417.10 (m), 1394.71 (m), 1374.43 (m), 1331.84 (w), 1303.65 (w), 1274.34 (m), 1225.34 (m), 1198.59 (m), 1185.15 (st), 1169.47 (m), 1157.51 (m), 1093.16 (m), 1068.73 (m), 1048.61 (st), 1025.03 (m), 1007.33 (m), 980.50 (m), 937.76 (m), 926.24 (st), 848.32 (m), 827.66 (st), 806.66 (m), 780.64 (st), 761.58 (m), 750.35 (m), 742.57 (m), 696.48 (w), 682.77 (w), 646.04 (m), 618.36 (st), 574.48 (st), 551.11 (st).

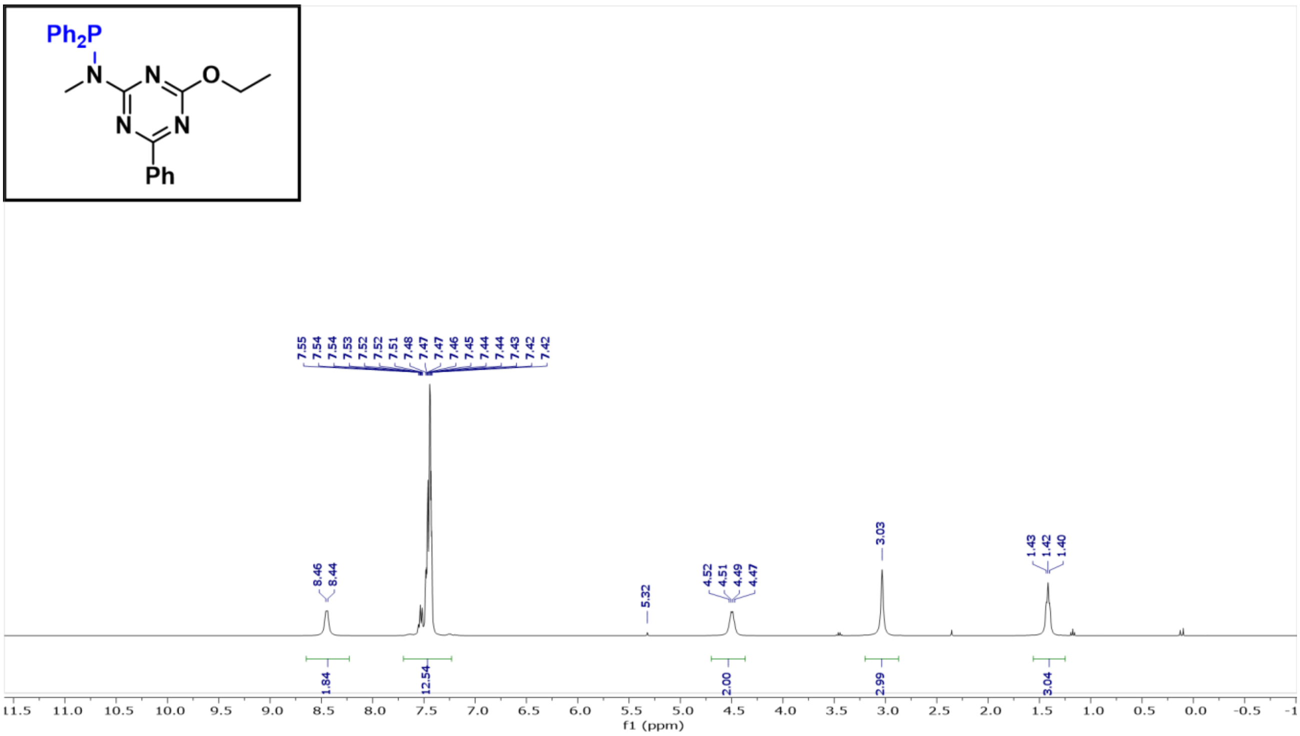

Figure S22 – ^{1}H NMR (400 MHz, $CDCl_3$, 296 K) spectrum of compound **26**.

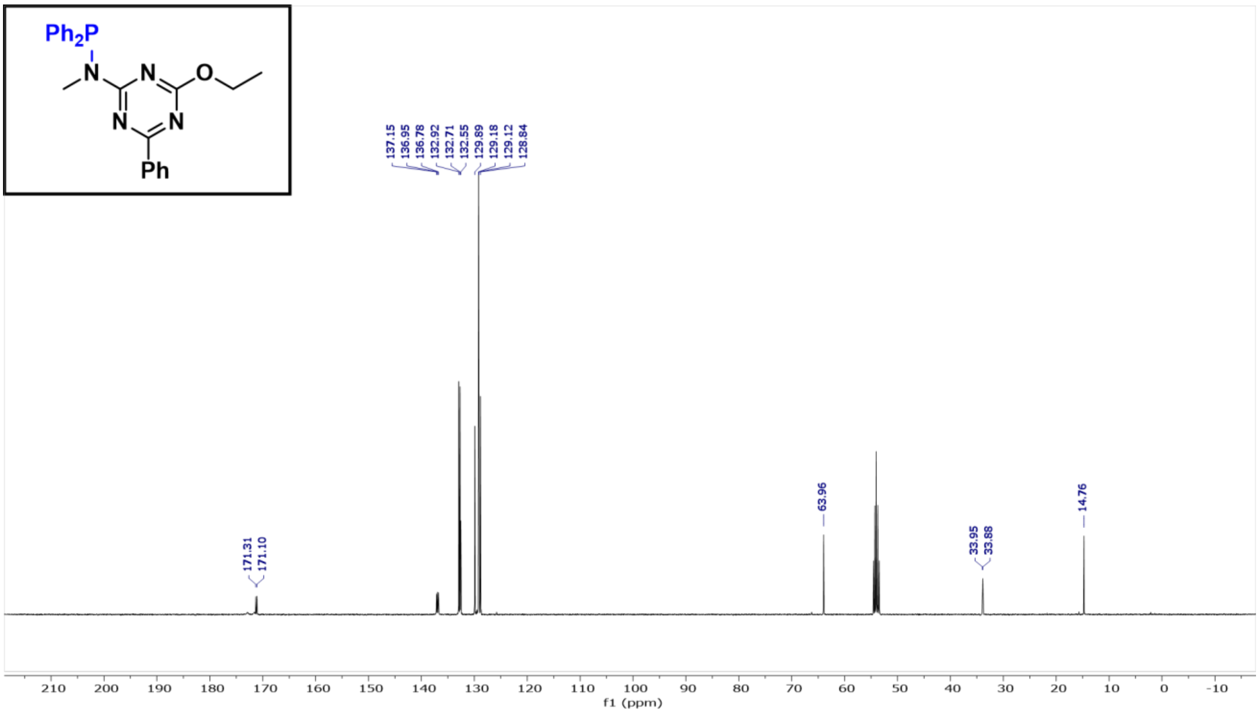

Figure S23 – ^{13}C{^{1}H} NMR (101 MHz, $CDCl_3$, 296 K) spectrum of compound **26**.

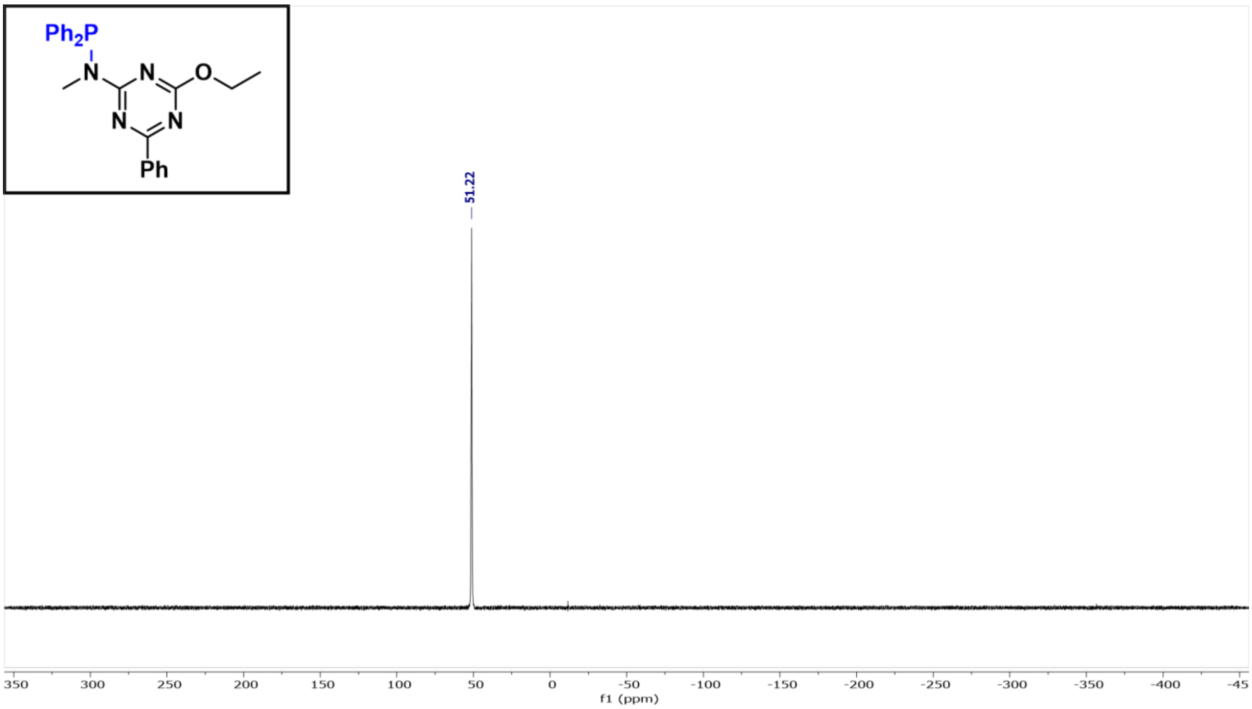

Figure S24 – $^{31}P\{^{1}H\}$ NMR (162 MHz, C_6D_6, 296 K) spectrum of compound **26**.

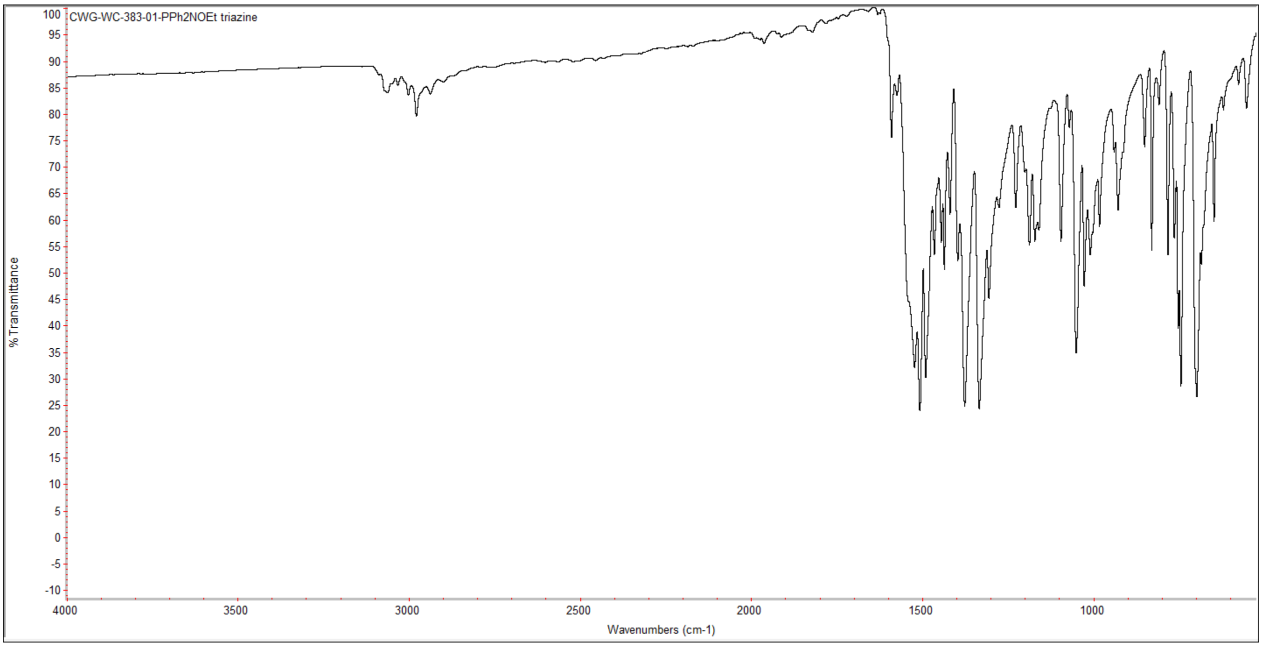

Figure S25 – IR-spectrum of compound **26**.

Complex Synthesis and Characterization

1. Synthesis of [Rh(Cp*)(MeCN)(PN^{tzn-B})]$(SbF_6)_2$ Complex (1)

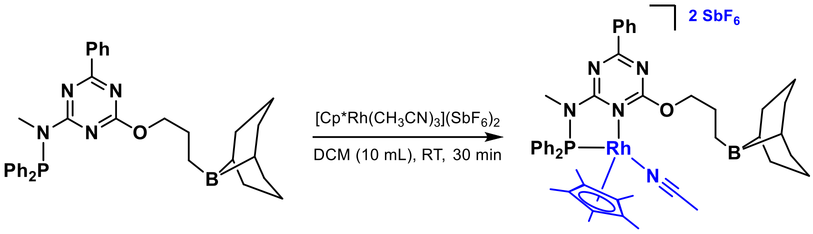

23 **1** (89%)

Procedure: A mixture of ligand **23** (230 mg, 0.42 mmol), [Cp*Rh(CH_3CN)$_3$]$(SbF_6)_2$ (350 mg, 0.42 mmol) in DCM (10 ml) was stirred for 30 min at room temperature. The resulting yellow solution was concentrated to ca. 3 mL *in vacuo*. Pentane (10 mL) was added dropwise, leading to the precipitation of a yellow solid. The precipitate was washed with pentane (3 × 5 mL) and dried under vacuum to give complex **1** as a yellow solid (501 mg, 0.37 mmol, 89%). Single crystals suitable for X-ray diffraction analysis were obtained by layering a concentrated solution of **1** in DCM (0.5 mL) with pentane (5 mL) at –30 °C.

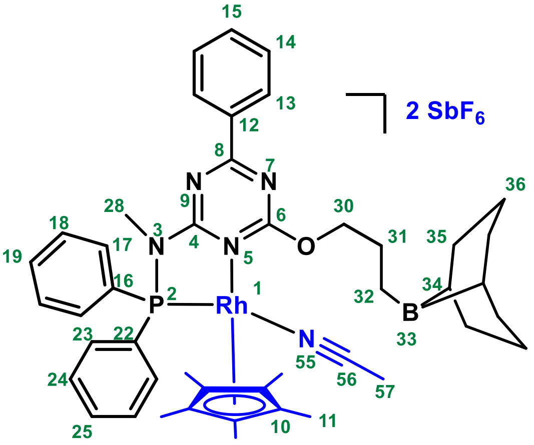

^{1}H NMR (400 MHz, CD_2Cl_2, 296 K): δ 8.59 (dt, *J* = 8.5, 1.6 Hz, 2H, 13), 7.91 – 7.44 (m, 16H, 14, 15, 17, 18, 19, 23, 24, 25), 5.21 (dt, *J* = 10.6, 7.6 Hz, 1H, 30), 4.56 (dt, *J* = 10.4, 7.8 Hz, 1H, 30), 3.43 (d, *J* = 5.5 Hz, 3H, 28), 2.08 (p, *J* = 7.8 Hz, 2H, 31), 1.91 – 1.79 (m, 6H, 36, 35), 1.77 (s, 3H, 57), 1.74 – 1.67 (m, 2H, 34), 1.61 (d, *J* = 4.1 Hz, 13H, 11), 1.56 – 1.47 (m, 2H, 35), 1.35 – 1.29 (m, 3H, 32), 1.30 – 1.22 (m, 1H, 36).

^{13}C{^{1}H} NMR (101 MHz, CD_2Cl_2, 296 K): δ 175.0 (8), 170.7 (d, *J* = 21.9 Hz, 4), 168.7 (d, *J* = 2.7 Hz, 6), 135.4 – 135.3 (m, 16), 135.2 (15), 134.1 (12), 133.8 (22), 133.4 (d, *J* = 11.4 Hz, 23), 130.6 (13), 129.4 (14), 127.1 (d, *J* = 51.4 Hz, 56), 106.1 (dd, *J* = 6.6, 2.5 Hz, 10), 74.9 (30), 37.6 (d, *J* = 4.5 Hz, 28), 33.4 (35), 31.4 – 29.3 (m, 34), 24.6 (31), 24.0 (36), 22.0 (32), 10.1 (d, *J* = 1.3 Hz, 11), 2.9 (57).

^{31}P NMR (162 MHz, CD_2Cl_2, 296 K): δ 101.8 (d, *J* = 135.8 Hz, 2).

^{11}B NMR (128 MHz, CD_2Cl_2, 296 K): δ 80.3 (33).

HRMS (ESI$^+$) Calcd. (%) for ($C_{47}H_{59}BN_6ORh$): 413.66798; Found: 413.66824.

Anal. Calcd. (%) for ($C_{45}H_{56}BF_{12}N_5OPRhSb_2^{2-}$ + 0.3 CH_2Cl_2): C 41.07, H 4.31, N 5.29; Found: C 40.85, H 4.23, N 5.38.

IR (Diamond-ATR, neat), υ (cm^{-1}): 3064.10 (st), 2931.92 (st), 1690.74 (st), 1566.93 (m), 1519.38 (m), 1474.39 (m), 1436.88 (m), 1382.57 (m), 1337.63 (m), 1266.19 (st), 1217.90 (st), 1166.75 (st), 1100.18 (st), 1019.01 (st), 998.44 (st), 936.07 (st), 821.48 (st), 782.55 (m), 733.12 (w), 698.84 (m), 652.27 (w), 557.02 (m).

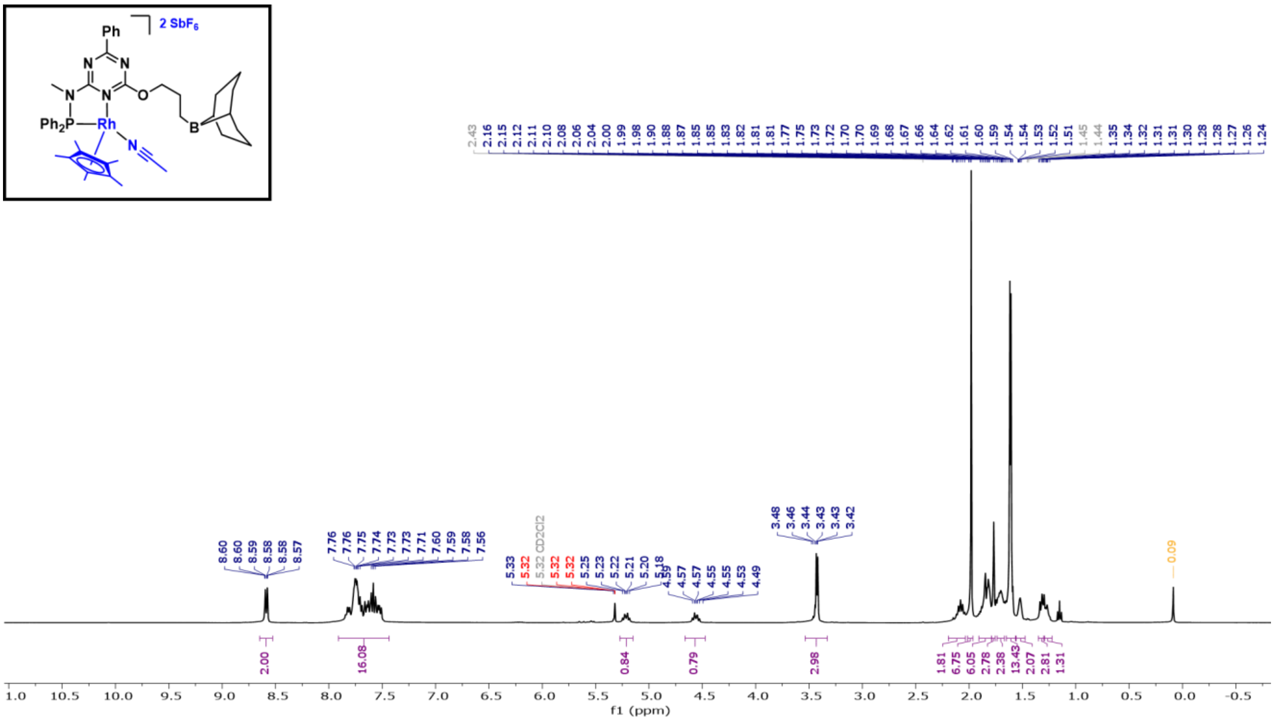

Figure S26 – ^{1}H NMR (400 MHz, CD_2Cl_2, 296 K) spectrum of compound **1**.

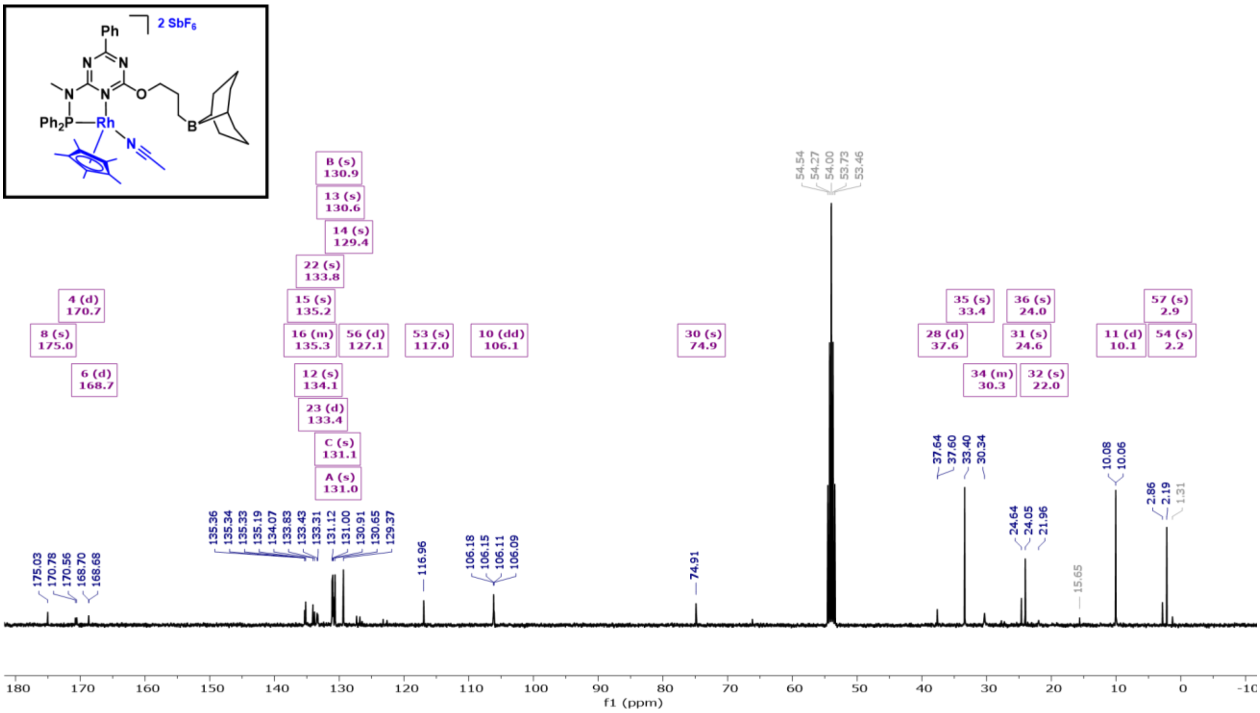

Figure S27 – $^{13}C\{^{1}H\}$ NMR (101 MHz, CD_2Cl_2, 296 K) spectrum of compound **1**.

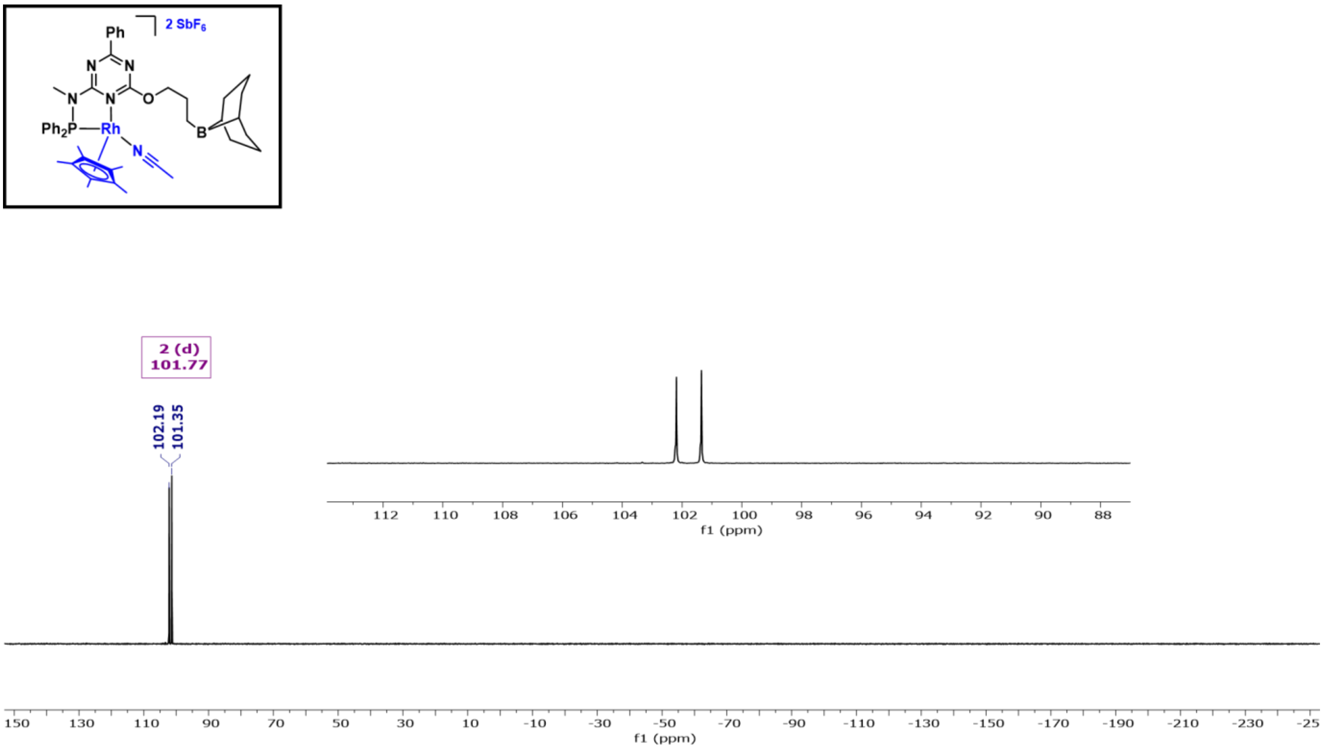

Figure S28 – $^{31}P\{^{1}H\}$ NMR (162 MHz, CD_2Cl_2, 296 K) spectrum of compound **1**.

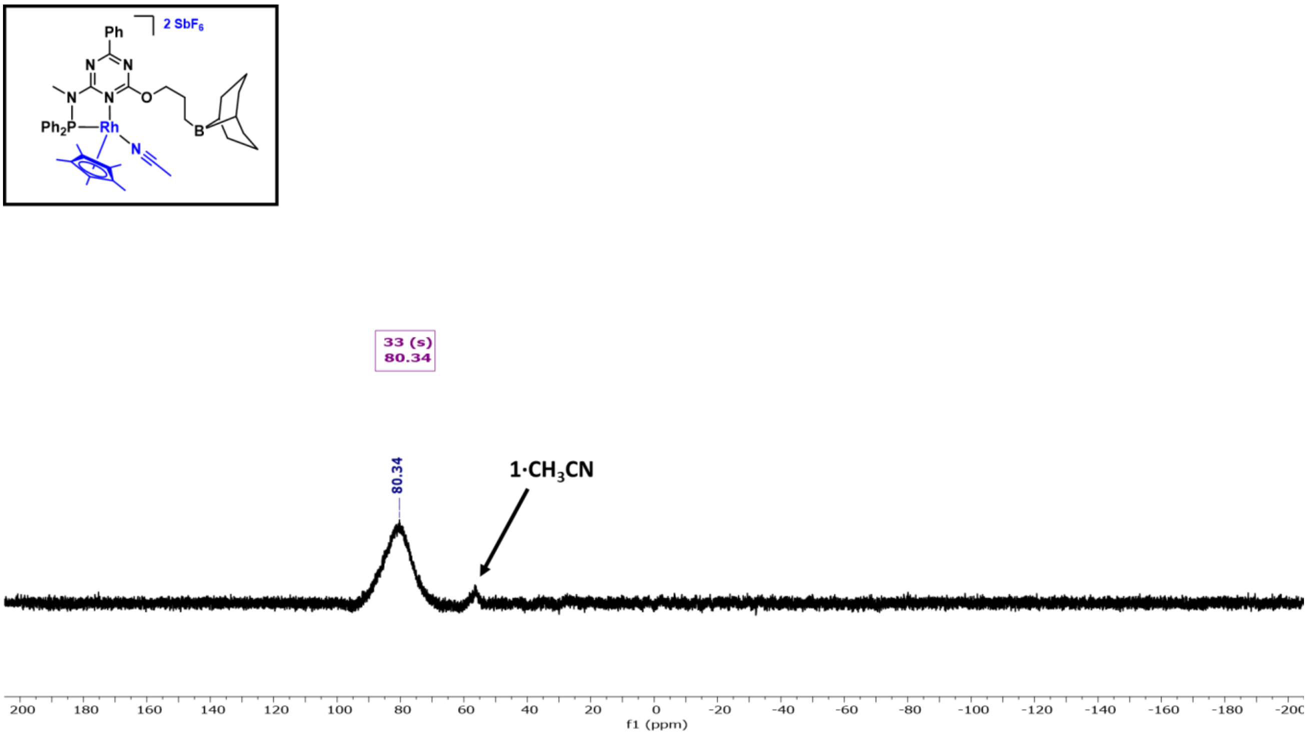

Figure S29 – ^{11}B NMR (128 MHz, CD_2Cl_2, 296 K) spectrum of compound **1**.

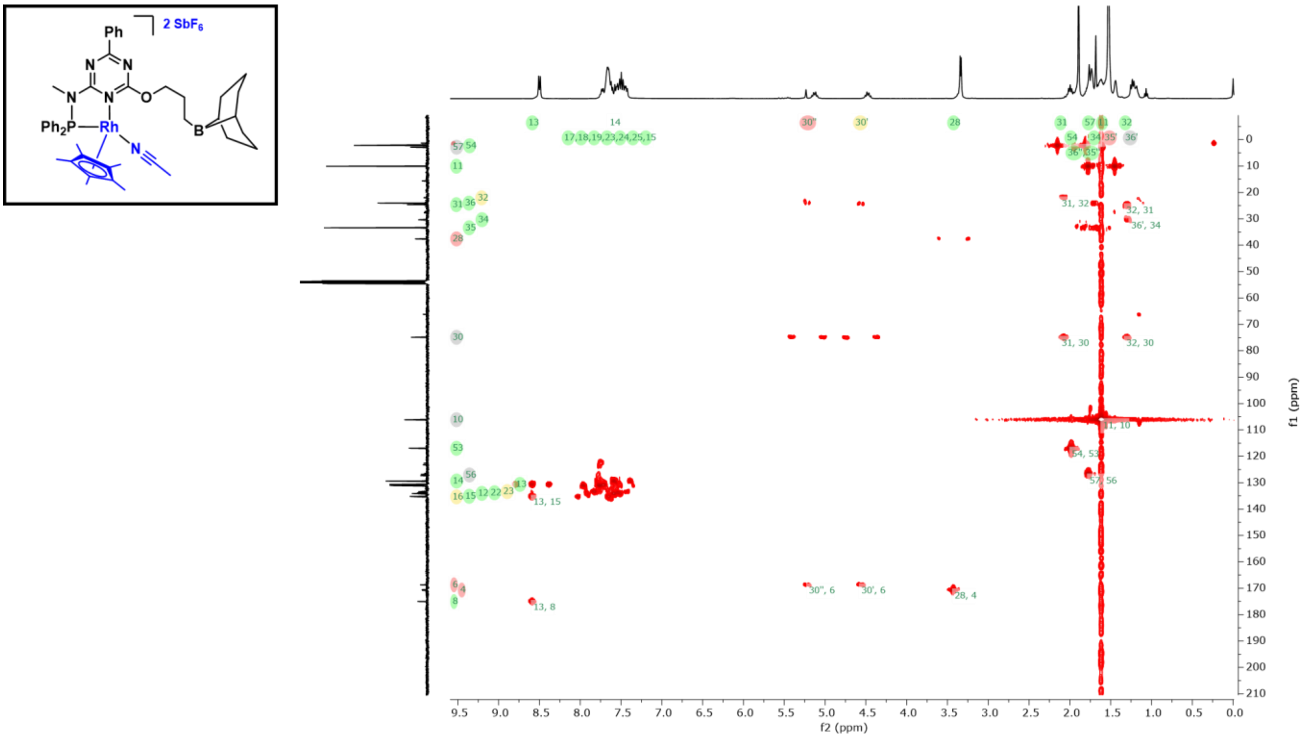

Figure S30 – ^{1}H-^{13}C-HMBC NMR (400, 101 MHz, CD_2Cl_2, 296 K) spectrum of compound **1**.

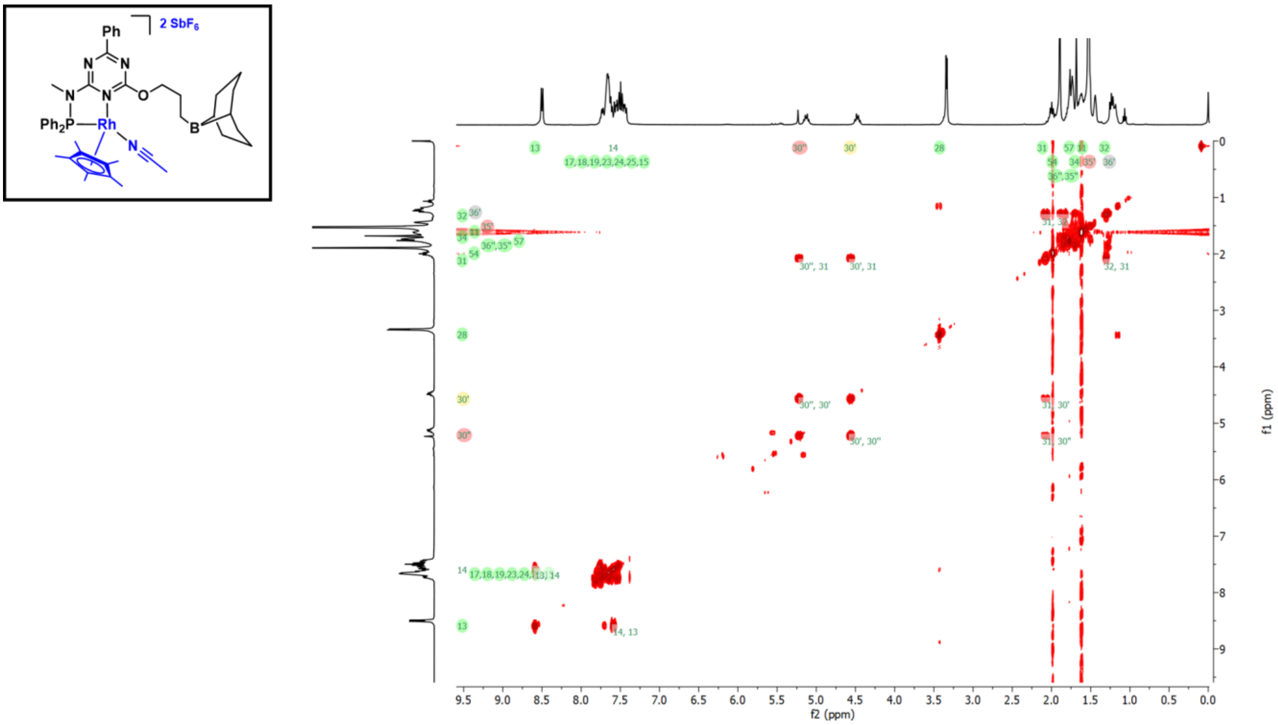

Figure S31 – ^{1}H-^{1}H-COSY NMR (400 MHz, CD_2Cl_2, 296 K) spectrum of compound **1**.

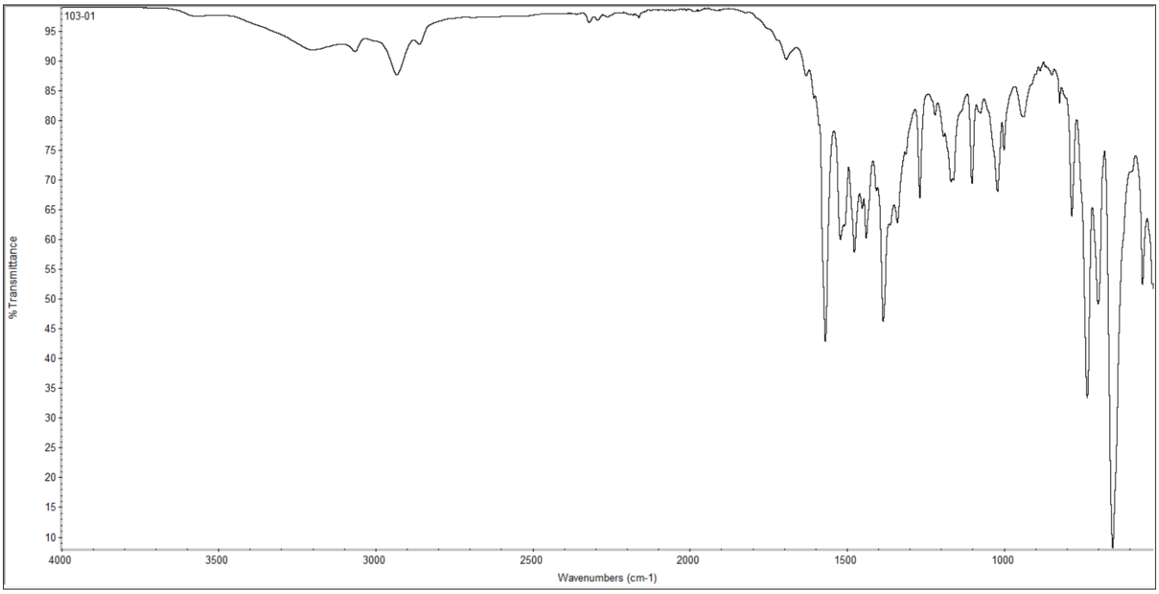

Figure S32 – IR spectrum of compound **1**.

2. Synthesis of [Rh(Cp*)(MeCN)(PN$^{tzn-OEt}$)](SbF_6)$_2$ Complex (6)

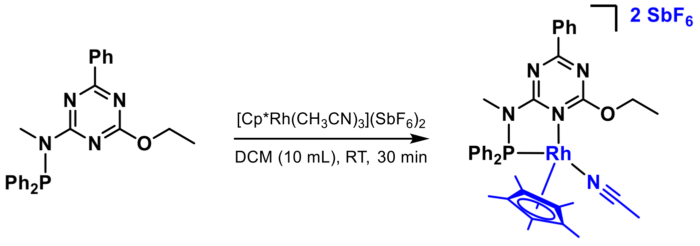

26

6 (92%)

Procedure: A mixture of ligand **26** (100 mg, 0.24 mmol), [Cp*Rh(CH_3CN)$_3$](SbF_6)$_2$ (201.2 mg, 0.24 mmol) in DCM (10 ml) was stirred 30 min at room temperature. The resulting yellow solution was concentrated to ca. 3 mL *in vacuo*. Pentane (10 mL) was added dropwise, leading to the precipitation of a yellow solid. The precipitate was washed with pentane (3 × 5 mL) and dried under vacuum to give **6** as a yellow solid (257 mg, 0.22 mmol, 92%). Single crystals suitable for X-ray diffraction analysis were obtained by layering a concentrated solution of **6** in DCM (0.5 mL) with pentane (5 mL) at –30 °C.

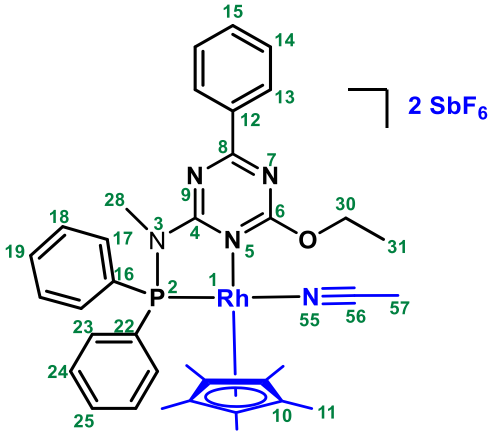

^{1}H NMR (500 MHz, CD_2Cl_2, 296 K): δ 8.7 – 8.5 (m, 2H, 13), 7.9 – 7.4 (m, 14H, 14, 15, 17, 18, 19, 23, 24, 25), 5.2 (dq, *J* = 11.1, 7.0 Hz, 1H), 4.7 (dq, *J* = 11.4, 7.1 Hz, 1H, 30), 3.4 (d, *J* = 5.5 Hz, 3H, 28), 1.8 (s, 3H, 57), 1.6 (d, *J* = 4.1 Hz, 16H, 11), 1.6 (t, *J* = 7.0 Hz, 3H, 31).

^{13}C{^{1}H} NMR (126 MHz, CD_2Cl_2, 296 K): 175.0 (8), 170.6 (d, *J* = 21.9 Hz, 4), 168.5 (d, *J* = 2.6 Hz, 6), 135.3 (d, *J* = 3.1 Hz, 16), 135.1 (15), 134.1 (12), 133.7 (d, *J* = 3.0 Hz, 22), 133.4 (d, *J* = 11.9 Hz, 23), 131.2 – 130.8 (m, 18, 19), 130.7 (13), 129.3 (14), 127.1 (d, *J* = 51.1 Hz, 56), 106.1 (dd, *J* = 6.6, 2.4 Hz, 10), 68.7 (30), 37.5 (d, *J* = 4.6 Hz, 28), 14.8 (31), 10.0 (11), 2.7 (57).

^{31}P NMR (202 MHz, CD_2Cl_2, 296 K): δ 101.8 (d, *J* = 136.4 Hz, 2).

HRMS (ESI^{+}) Calcd. (%) for ($C_{36}H_{41}N_5OPRh$): 346.60464; Found: 346.60513.

Anal. Calcd. (%) for ($C_{36}H_{41}F_{12}N_5OPRhSb_2^{2-}$): C 37.11, H 3.55, N 6.01; Found: C 37.20, H 3.74, N 5.72.

IR (Diamond-ATR, neat), υ (cm^{-1}): 3062.98 (st), 2987.87 (st), 2936.83 (st), 2318.47 (st), 2291.55 (st), 1567.03 (m), 1519.82 (m), 1477.66 (m), 1449.22 (m), 1433.87 (m), 1405.13 (st), 1382.64 (m), 1342.31 (m), 1310.66 (st), 1266.66 (m), 1218.49 (st), 1175.42 (st), 1157.61 (st), 1099.80 (m), 1036.47 (m), 1020.44 (st), 999.11 (st), 934.76 (m), 867.14 (st), 822.21 (st), 783.27 (m), 734.68 (w), 700.10 (m), 655.12 (w), 589.47 (st), 557.20 (m), 526.38 (m).

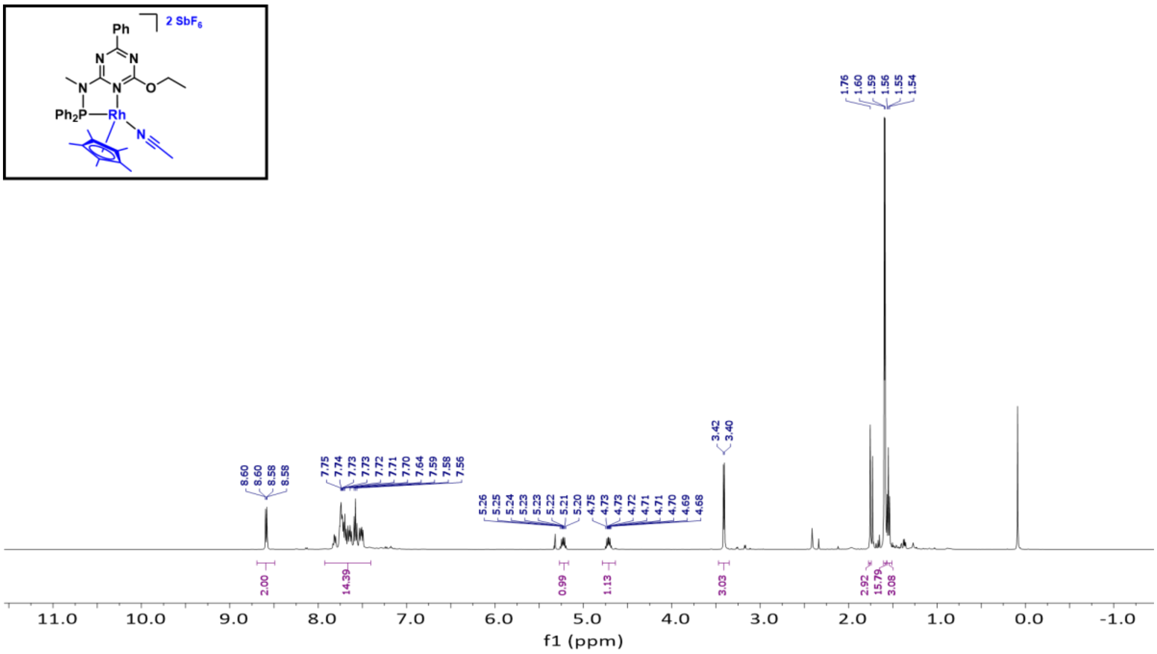

Figure S33 – ^{1}H NMR (500 MHz, CD_2Cl_2, 296 K) spectrum of compound **6**.

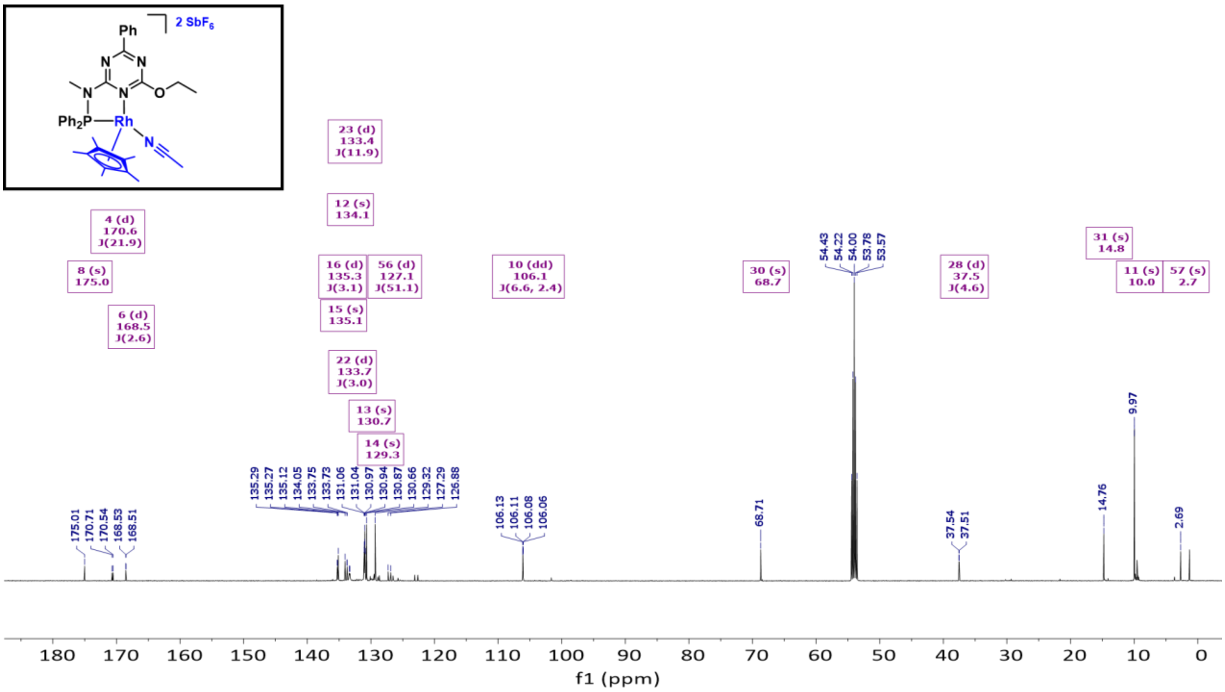

Figure S34 – $^{13}C\{^{1}H\}$ NMR (126 MHz, CD_2Cl_2, 296 K) spectrum of compound **6**.

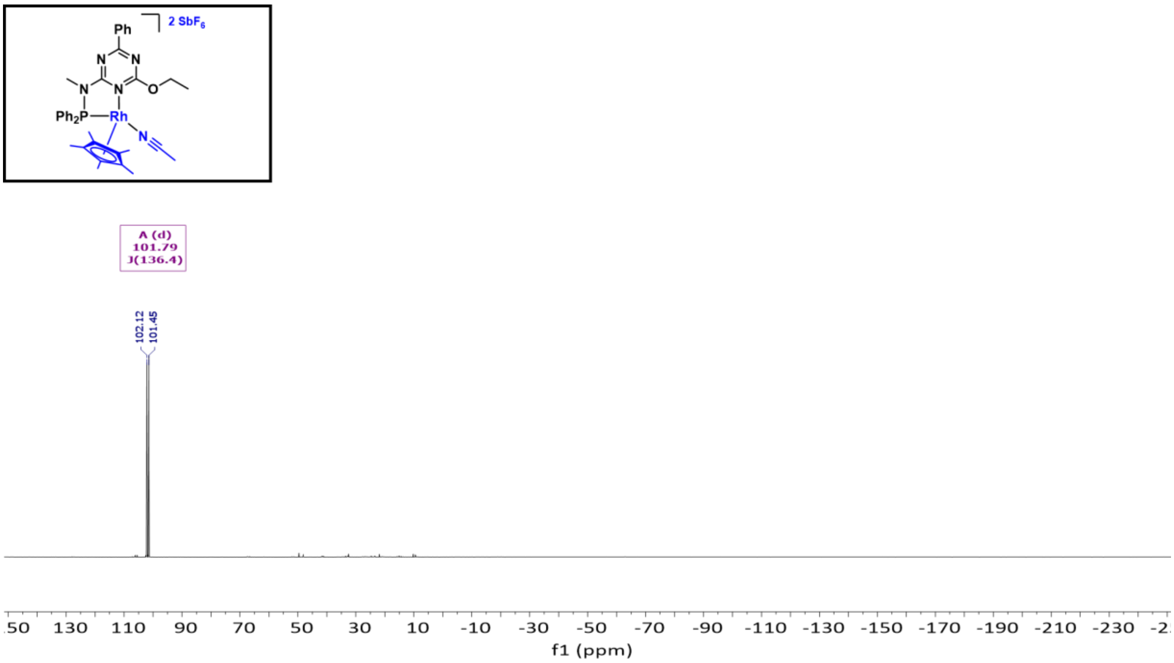

Figure S35 – $^{31}P\{^{1}H\}$ NMR (202 MHz, CD_2Cl_2, 296 K) spectrum of compound **6**.

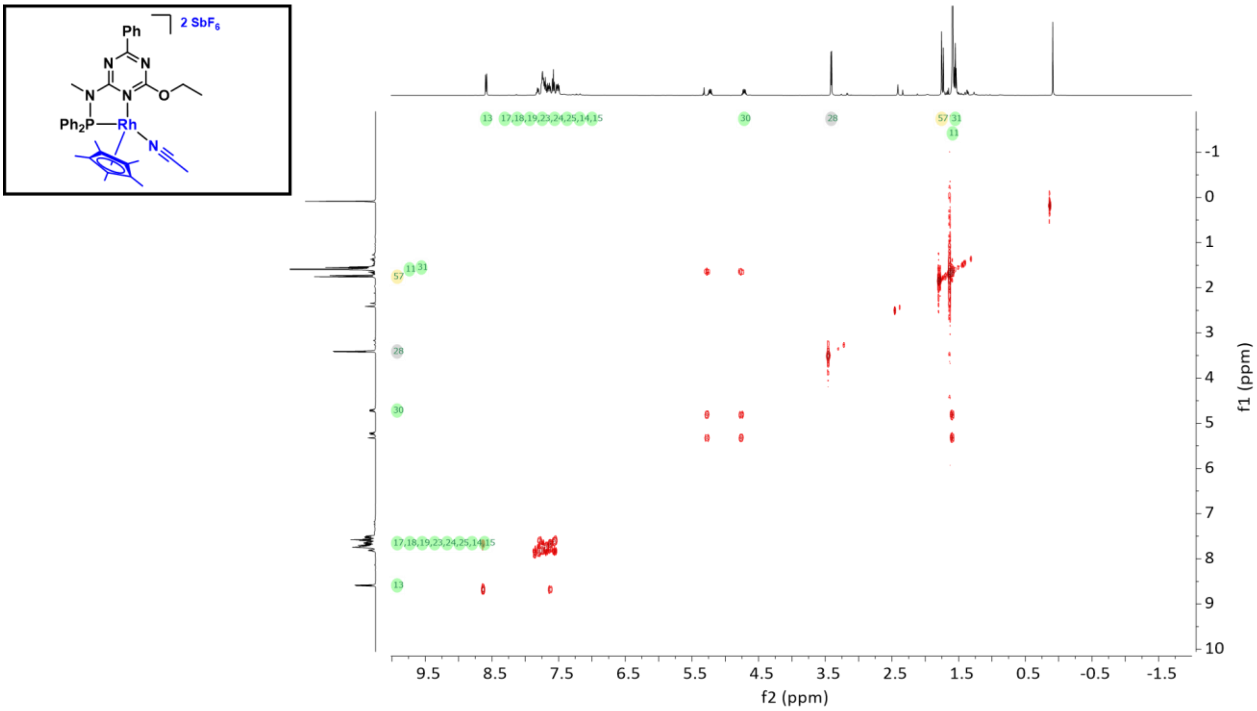

Figure S36 – ^{1}H-^{1}H-COSY NMR (500 MHz, CD_2Cl_2, 296 K) spectrum of compound **6**.

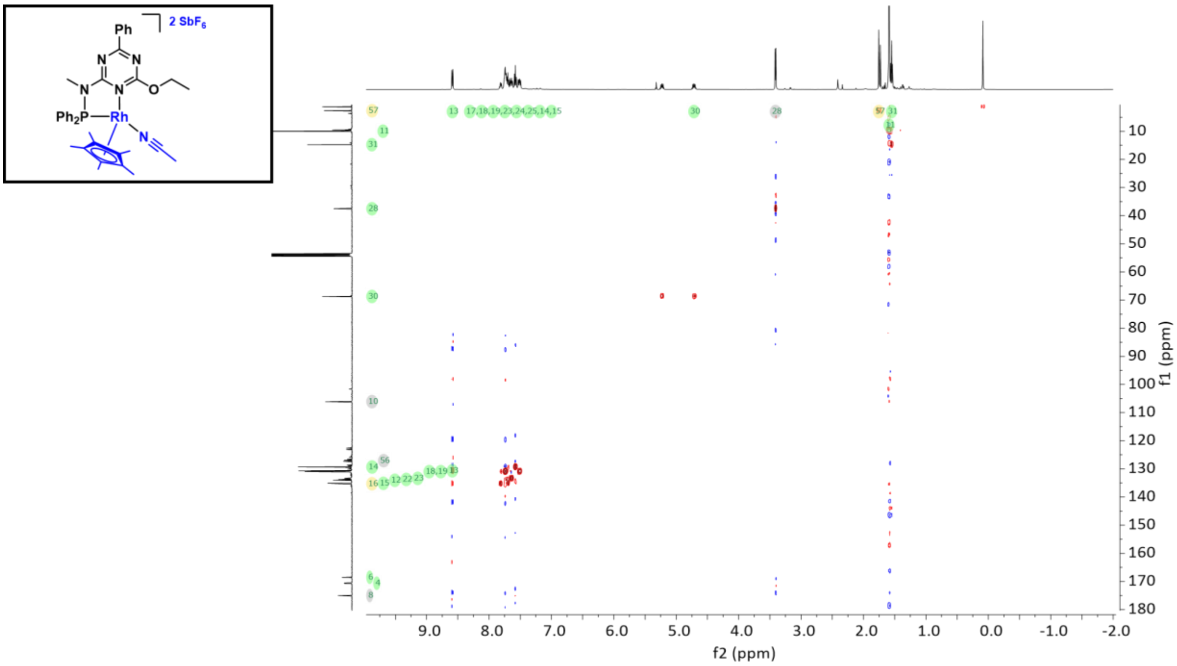

Figure S37 – ^{1}H-^{13}C-HSQC NMR (500, 126 MHz, CD_2Cl_2, 296 K) spectrum of compound **6**.

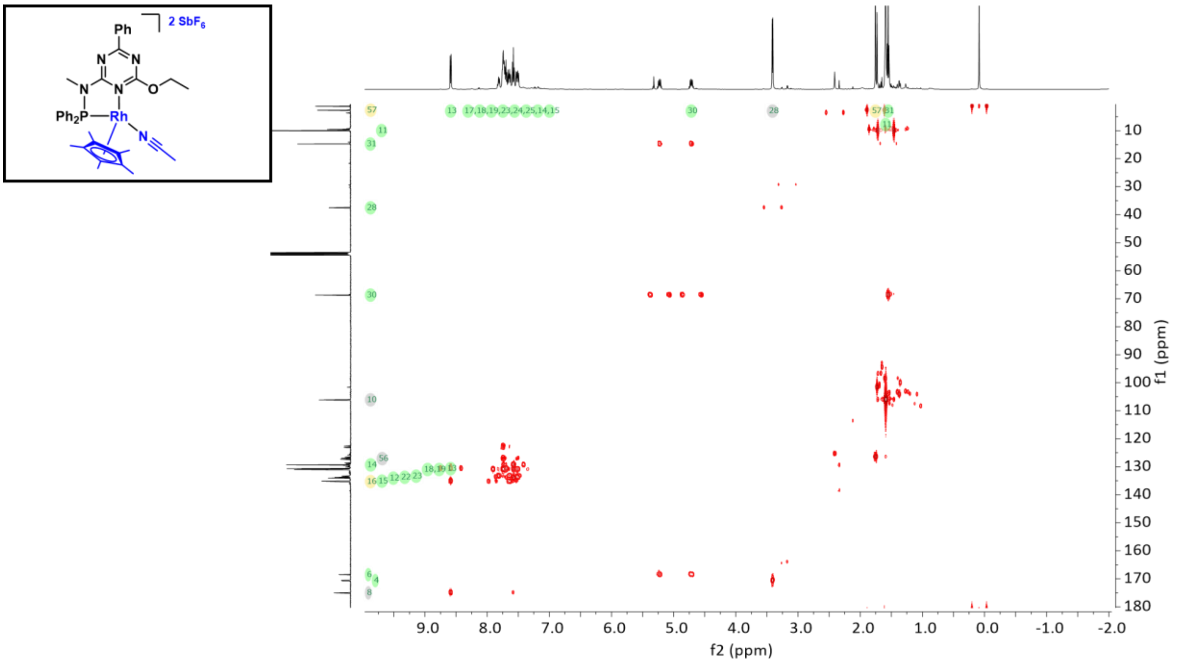

Figure S38 – ^{1}H-^{13}C-HMBC NMR (500, 126 MHz, CD_2Cl_2, 296 K) spectrum of compound **6.**

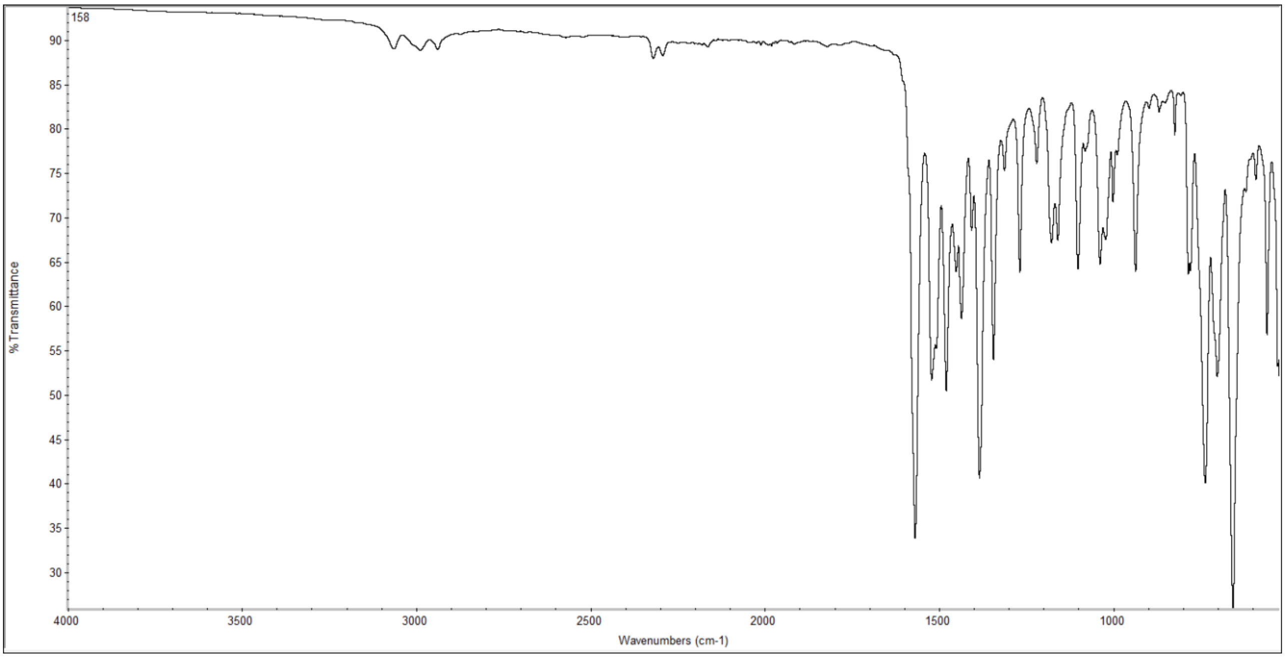

Figure S39 – IR-spectrum of compound **6**.

3. Synthesis of [Rh(COD)(PN^{tzn-B})](SbF$_6$) Complex (5)

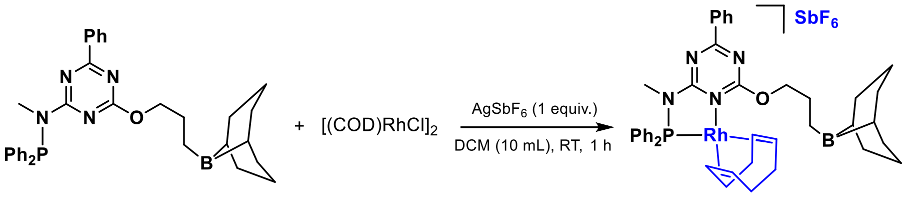

23 (1 equiv.) (0.5 equiv.) **5** (98%)

Procedure: A mixture of ligand **23** (230 mg, 0.42 mmol), Chloro(1,5-cyclooctadiene)rhodium(I) dimer (103.5 mg, 0.21 mmol) in DCM (10 ml) was stirred overnight (16 h) at room temperature. The resulting yellow solution was concentrated to ca. 3 mL *in vacuo*. Pentane (10 mL) was added dropwise, leading to the precipitation of a yellow solid. The precipitate was washed with pentane (3 × 5 mL) and dried *in vacuo* to give **5** as a yellow solid (408.1 mg, 0.41 mmol, 98%). Single crystals suitable for X-ray diffraction analysis were obtained by layering a concentrated solution of **5** in THF (0.5 mL) with an Et_2O/pentane (1:2, 3 mL) mixture of solvents at –30 °C.

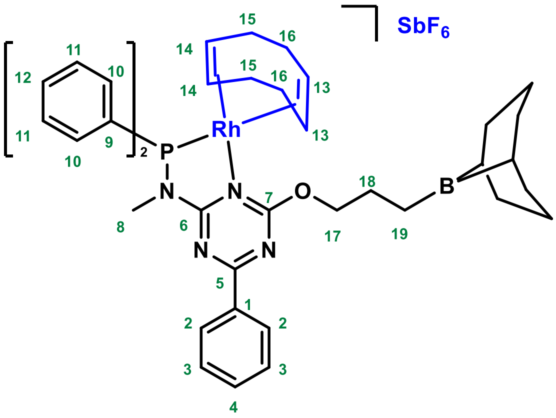

^{1}H NMR (400 MHz, CD_2Cl_2, 296 K): δ 8.50 (d, J = 7.2 Hz, 2H, 2), 7.79 – 7.60 (m, 11H, 1H from 4, 10H from 9-12), 7.54 (t, J = 7.8 Hz, 2H, 3), 6.37 (brs, 2H, 13), 4.71 (t, J = 7.3 Hz, 2H, 17), 3.57 (brs, 2H, 14), 3.15 (d, J = 5.0 Hz, 3H, 8), 2.51 – 2.17 (m, 8H, 15-16), 2.11 (p, J = 7.5 Hz, 2H, 18), 1.98 – 1.62 (m, 12H, BBN), (t, J = 7.5 Hz, 2H, 19), 1.31 – 1.17 (m, 2H, BBN).

^{13}C{^{1}H} NMR (101 MHz, CD_2Cl_2, 296 K): δ 173.6 (s, 5), 171.6 (d, J = 22.5 Hz, 6), 170.3 (s, 7), 135.0 (s, 4), 134.3 (s, 1), 133.8 (d, J = 2.3 Hz, 12), 133.4 (d, J = 13.3 Hz, 10), 130.4 (d, J = 11.2 Hz, 11), 130.2 (s, 2), 129.4 (s, 3), 127.0 (d, J = 53.5 Hz, 9), 113.9 (dd, J = 10.0, 5.6 Hz, 13), 78.7 (d, J = 12.5 Hz, 14), 73.9 (s, 17), 34.5 (d, J = 4.7 Hz, 8), 33.7 (s, C_{BBN}), 31.8 (brs, C_{BBN}), 31.7 (d, J = 2.7 Hz, 16), 29.6 (s, 15), 24.0 (s, 18), 24.0 (brs, 19), 23.7 (s, C_{BBN}).

^{31}P NMR (162 MHz, CD_2Cl_2, 296 K): δ 100.07 (d, J = 162.7 Hz).

^{11}B NMR (193 MHz, CD_2Cl_2, 296 K): δ 88.74.

HRMS (ESI$^+$) Calcd. (%) for ($C_{41}H_{50}BN_4OPRh$): 777.29705; Found: 777.29733.

Anal. Calcd. (%) for ($C_{41}H_{50}BF_6N_4OPRhSb^-$): C 49.48, H 5.06, N 5.63; Found: C 49.27, H 4.89, N 5.66%.

IR (Diamond-ATR, neat), υ (cm^{-1}): 3059.82 (st), 2884.12 (st), 2836.56 (st), 1587.22 (st), 1553.31 (w), 1503.87 (m), 1470.05 (m), 1446.22 (m), 1433.89 (m), 1401.74 (st), 1377.86 (m), 1351.81 (m), 1332.14 (m), 1308.37 (st), 1265.29 (m), 1224.81 (st), 1162.75 (m), 1097.48 (m), 1071.44 (st), 1027.07 (st), 997.38 (st), 970.45 (st), 933.84 (st), 869.61 (st), 822.59 (st), 782.44 (m), 732.66 (w), 693.64 (m), 652.75 (w), 558.59 (m).

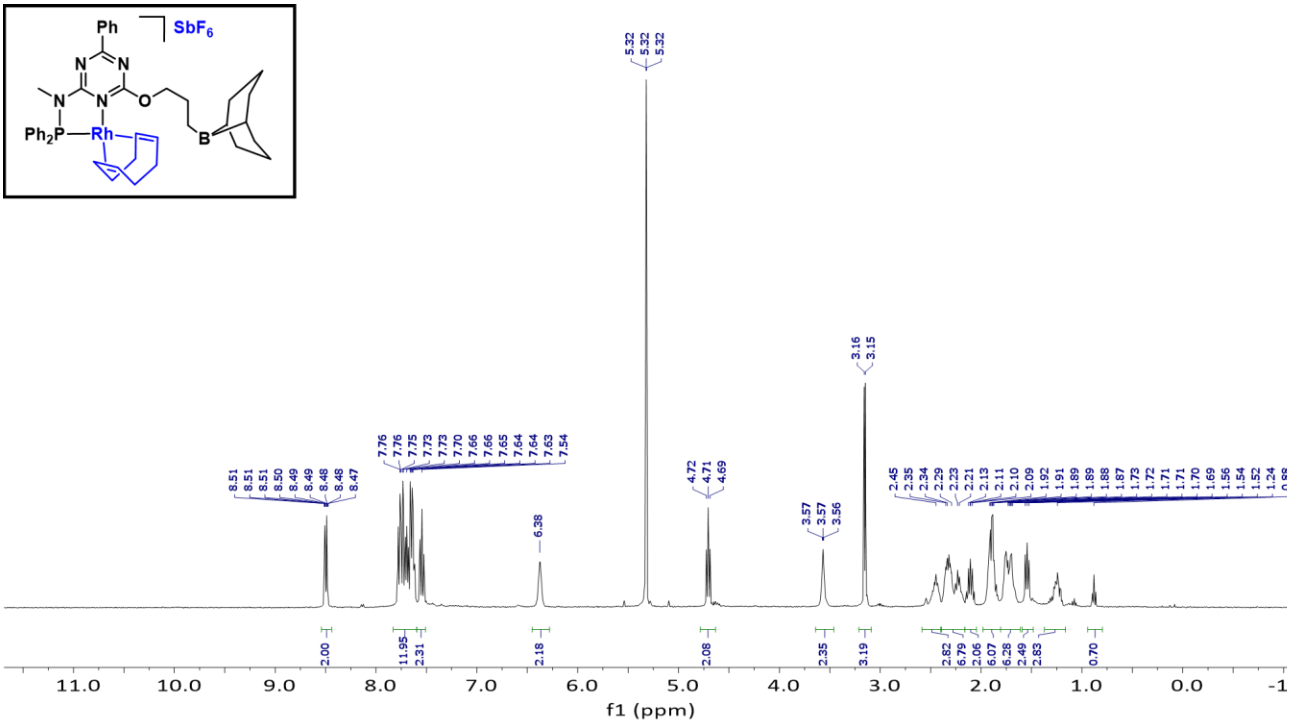

Figure S40 – ^{1}H NMR (400 MHz, CD_2Cl_2, 296 K) spectrum of compound **5**.

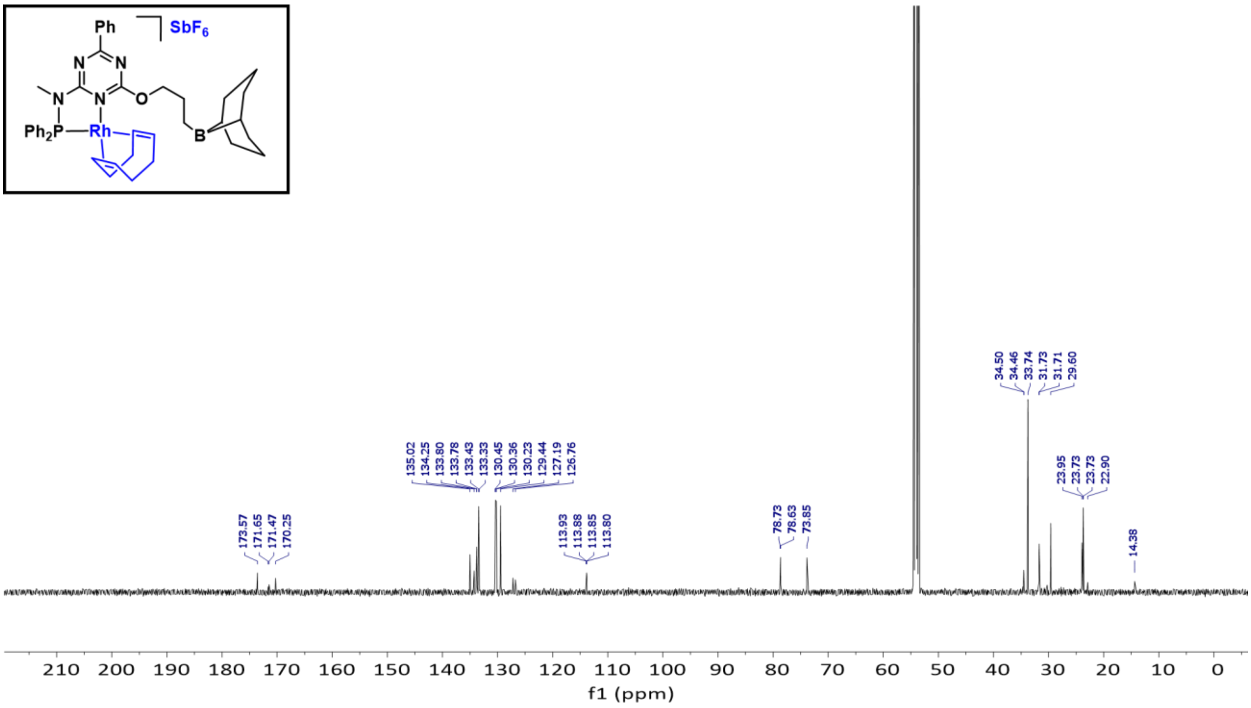

Figure S41 – $^{13}C\{^1H\}$ NMR (101 MHz, CD_2Cl_2, 296 K) spectrum of compound **5**.

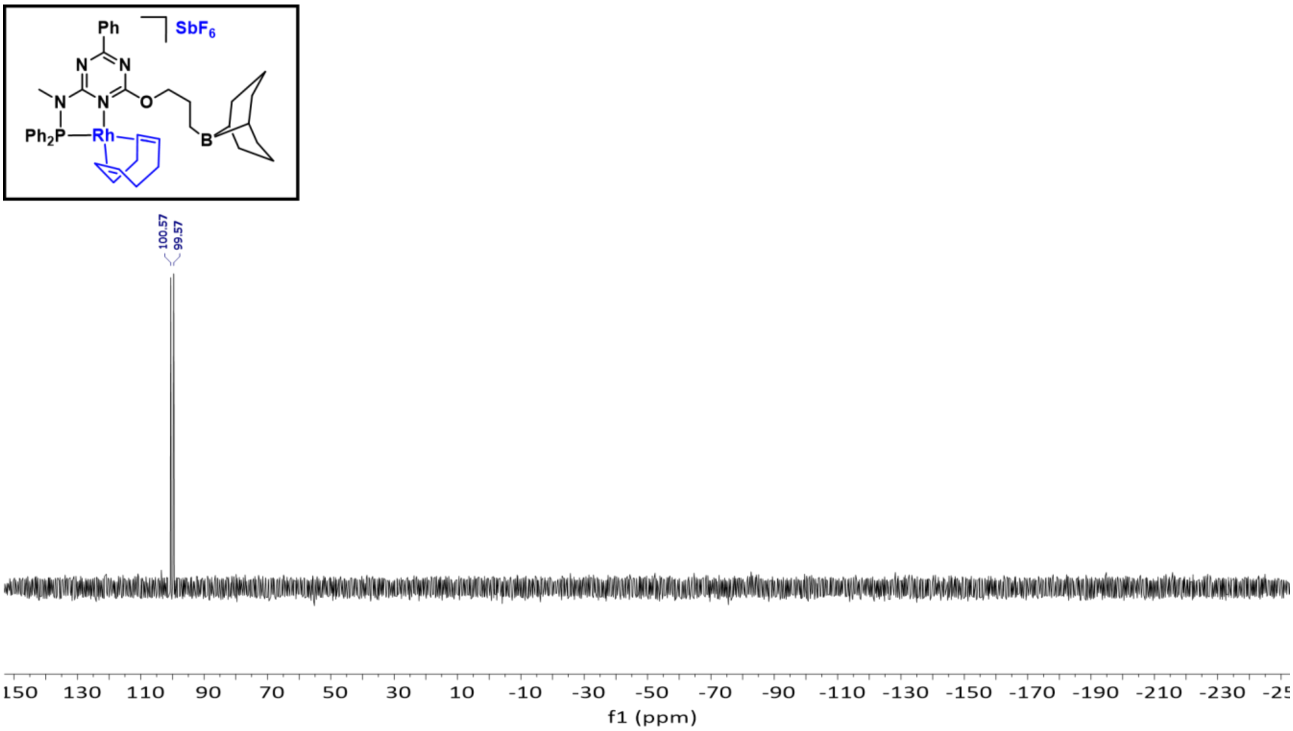

Figure S42 – $^{31}P\{^1H\}$ NMR (162 MHz, CD_2Cl_2, 296 K) spectrum of compound **5**.

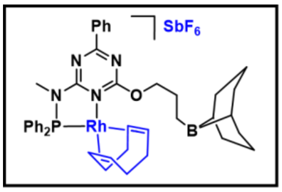

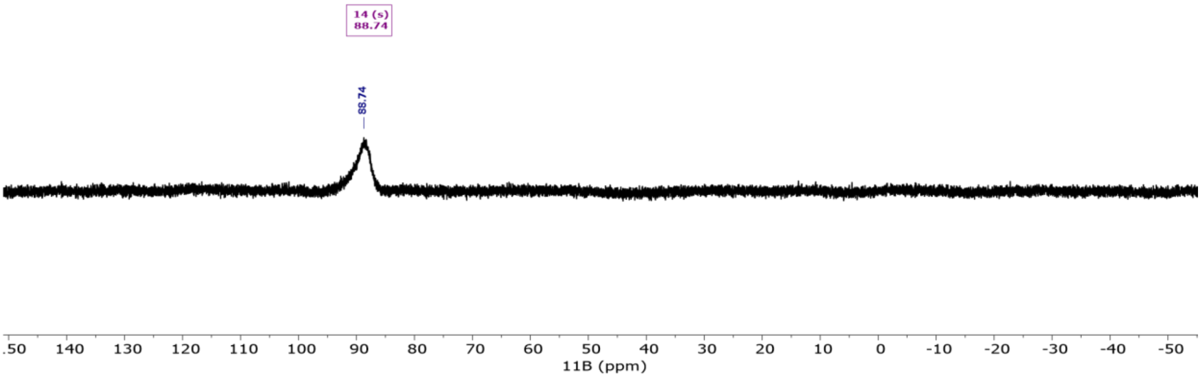

Figure S43 – ^{11}B NMR (193 MHz, CD_2Cl_2, 296 K) spectrum of compound **5**.

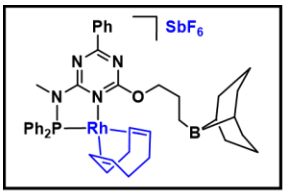

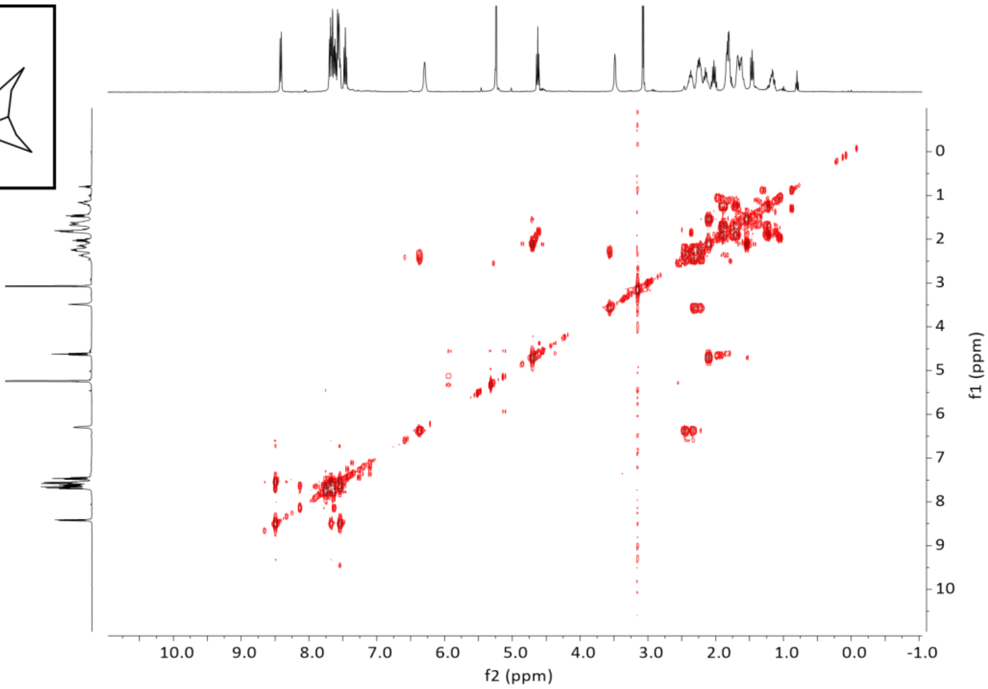

Figure S44 – ^{1}H-^{1}H-COSY NMR (400 MHz, CD_2Cl_2, 296 K) spectrum of compound **5**.

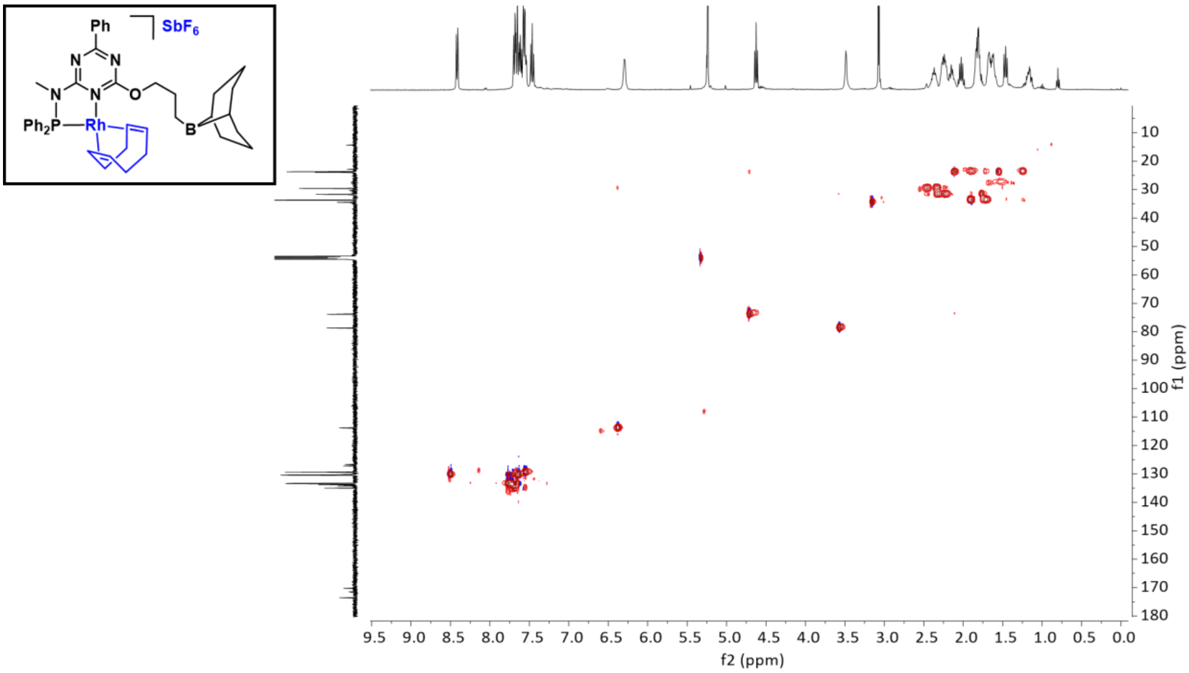

Figure S45 – ^{1}H-^{13}C-HSQC NMR (400, 101 MHz, CD_2Cl_2, 296 K) spectrum of compound **5**.

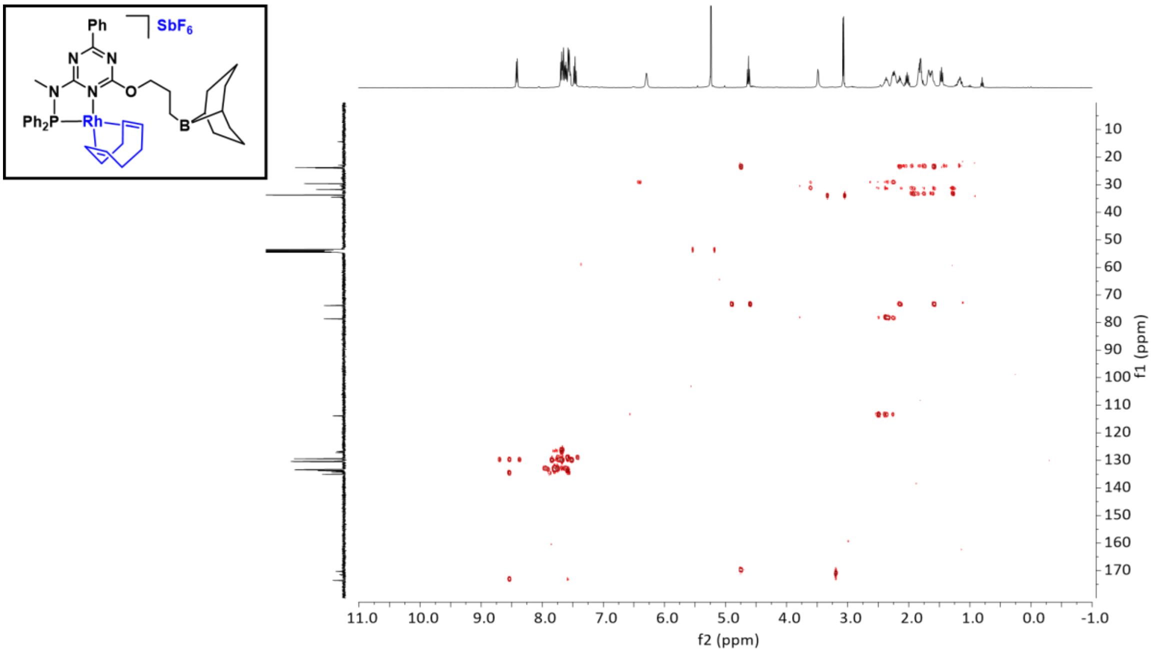

Figure S46 – ^{1}H-^{13}C-HMBC NMR (400, 101 MHz, CD_2Cl_2, 296 K) spectrum of compound **5.**

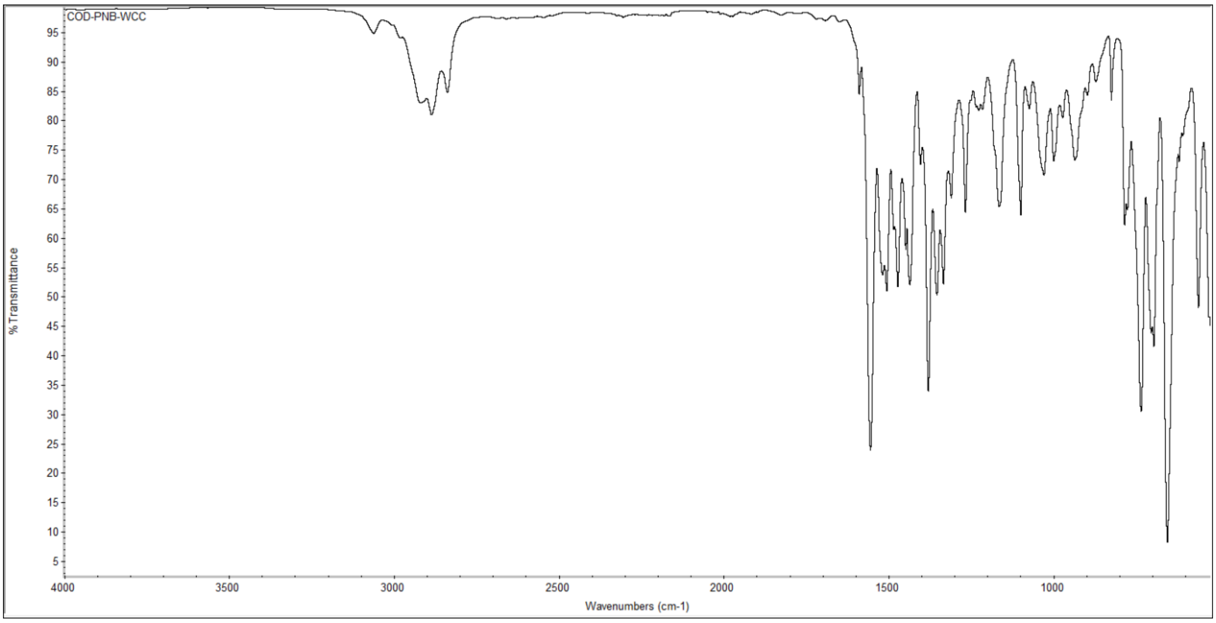

Figure S47 – **IR**-spectrum of compound **5**.

Optimization of Reaction Parameters for Hydroxylamine Synthesis

1. Solvent

Table S1 – Optimization of the reaction conditions for hydroxylamines: **Solvent**.

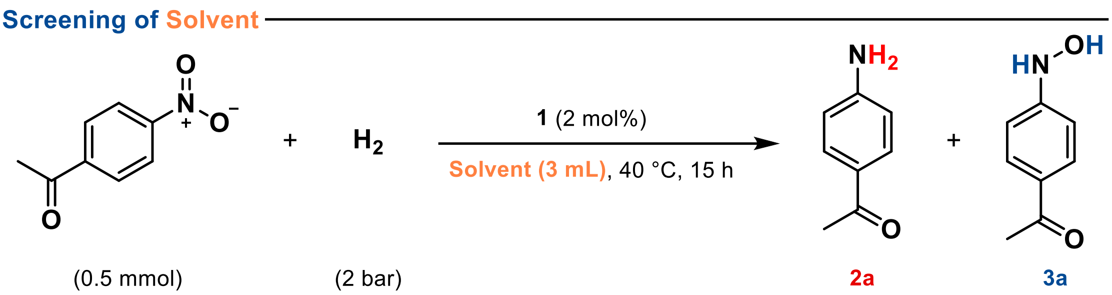

Entry	Deviation from Above	Yield (%)[a] 2a:3a
**1**	**THF**	**7:70**
2	Toluene	0:0
3	MeCN	0:0
4	Ph-Cl	0:0
5	Et_2O	0:0
6	DCM	7:8

[a] *Yields are based on ^{1}H NMR relative to mesitylene (0.5 mmol) as an internal standard.*

2. Temperature

Table S2 – Optimization of the reaction conditions for hydroxylamines: **Temperature**.

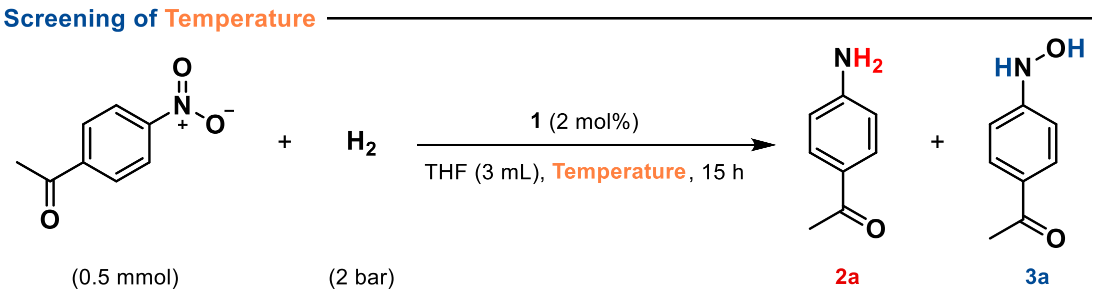

Entry	Deviation from Above	Yield (%)[a] 2a:3a
1	RT	5:26
**2**	**40 °C**	**7:70**
3	60 °C	10:71
4	80 °C	15:68

[a] *Yields are based on ^{1}H NMR relative to mesitylene (0.5 mmol) as an internal standard.*

3. Time

Table S3 – Optimization of the reaction conditions for hydroxylamines: **Time**.

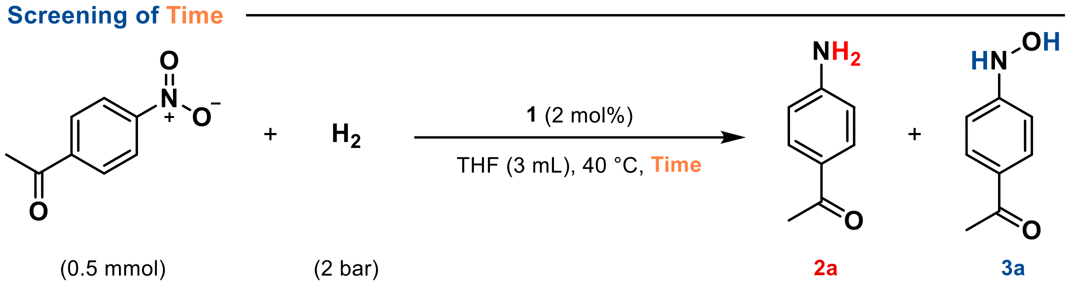

Entry	Deviation from Above	Yield (%)[a] 2a:3a
1	5 h	0:50
**2**	**15 h**	**7:70**
3	20 h	7:65
4	40 h	17:49

[a]*Yields are based on ^{1}H NMR relative to mesitylene (0.5 mmol) as an internal standard.*

As reaction times increase beyond 15 hours, we observe larger amounts of condensation product **14**.

4. Pressure

Table S4 – Optimization of the reaction conditions for hydroxylamines: **Pressure**.

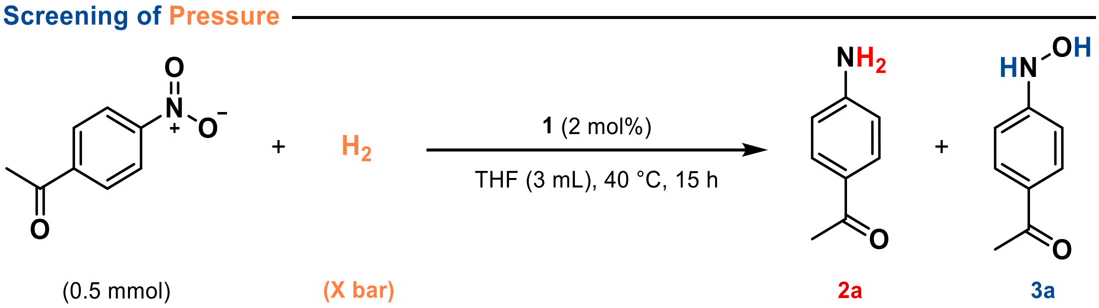

Entry	Deviation from Above	Yield (%)[a] 2a:3a
1	0 bar	0:0
2	1 bar	10:44
**3**	**2 bar**	**7:70**
4	3 bar	10:48
5	5 bar	24:50

[a]*Yields are based on ^{1}H NMR relative to mesitylene (0.5 mmol) as an internal standard.*

5. Catalyst Loading

Table S5 – Optimization of the reaction conditions for hydroxylamines: **Catalyst Loading**.

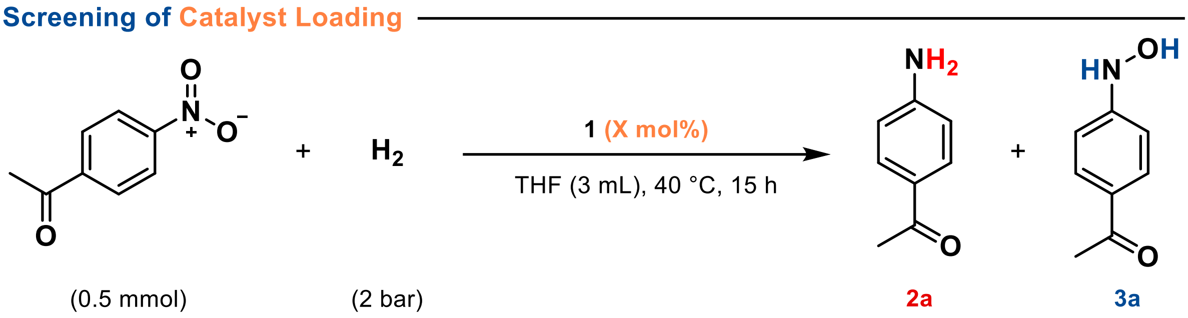

Entry	Deviation from Above	Yield (%)[a] 2a:3a
1	0.5 mol%	2:30
2	1.0 mol%	4:60
3	2.0 mol%	7:70
**4**	**3.0 mol%**	**8:91**

[a]*Yields are based on* [1]*H NMR relative to mesitylene (0.5 mmol) as an internal standard.*

Optimization of Reaction Parameters for Anilines Synthesis.

1. Pressure

Table S6 – Optimization of the reaction conditions for the aniline platform: **Pressure**.

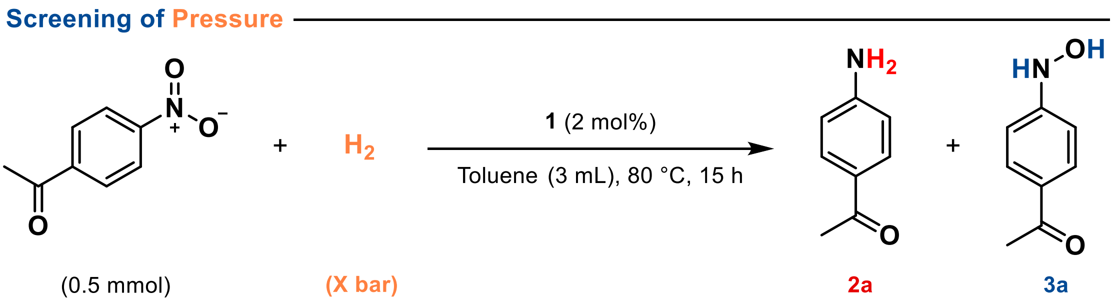

Entry	Deviation from Above	Yield (%)[a] 2a:3a
1	0 bar	0:0
2	1 bar	78:15
**3**	**2 bar**	**80:10**
4	3 bar	75:14
5	5 bar	78:17

[a]*Yields are based on* [1]*H NMR relative to mesitylene (0.5 mmol) as an internal standard.*

2. Temperature

Table S7 – Optimization of the reaction conditions for anilines: **Temperature**.

Screening of Temperature

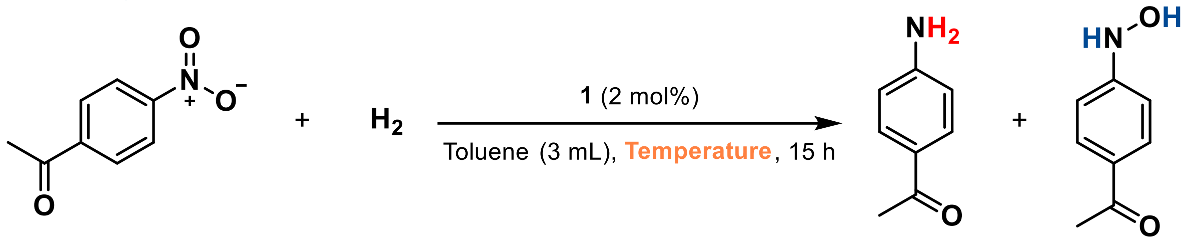

(0.5 mmol) (2 bar) 2a 3a

Entry	Deviation from Above	Yield (%)[a] 2a:3a
1	RT	0:0
2	40 °C	0:0
**3**	**60 °C**	**90:8**
4	80 °C	80:10
5	100 °C[b]	70:25

[a]*Yields are based on ^{1}H NMR relative to mesitylene (0.5 mmol) as an internal standard.*
[b]*Pressure = 5 bar*

3. Solvent

Table S8 – Optimization of the reaction conditions for anilines: **Solvent.**

Screening of Solvent

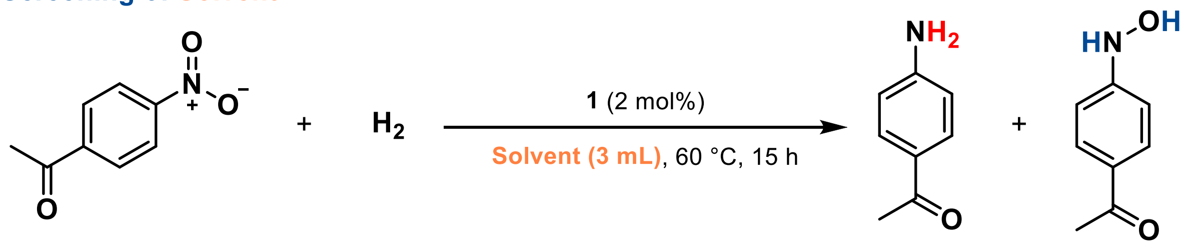

(0.5 mmol) (2 bar) 2a 3a

Entry	Deviation from Above	Yield (%)[a] 2a:3a
1	MeCN	0:0
**2**	**Toluene**	**90:8**
3	DCM	15:45
4	Et_2O	0:10

[a] *Yields are based on ^{1}H NMR relative to mesitylene (0.5 mmol) as an internal standard.*

4. Catalyst Loading

Table S9 – Optimization of the reaction conditions for anilines: **Catalyst Loading**.

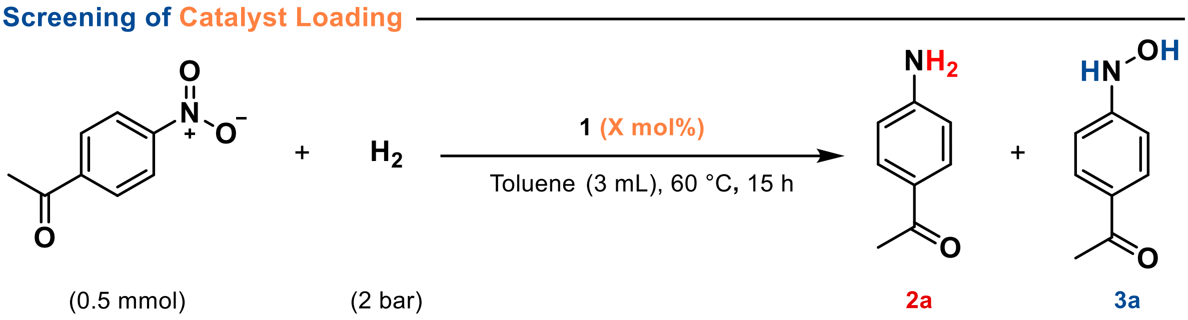

Entry	Deviation from Above	Yield (%)[a] 2a:3a
1	0.5 mol%	13:23
2	1.0 mol%	56:37
**3**	**2.0 mol%**	**90:8**
4	3.0 mol%	90:4

[a] *Yields are based on ^{1}H NMR relative to mesitylene (0.5 mmol) as an internal standard.*

General Procedure for Aniline Synthesis

In an oven-dried Fisher-Porter tube under argon, nitroarene (0.5 mmol), complex **1** (2 mol%), and toluene (3 mL) were charged. The reaction mixture was frozen using liquid nitrogen, evacuated, and then pressurized with H_2 (2 bar). The resulting mixture was stirred for 15 hours at 60 °C. The reaction vessel was cooled to ambient temperature, the pressure was released, and the solvent was removed *in vacuo*. The residue was analyzed by ^{1}H NMR spectroscopy relative to mesitylene (0.5 mmol) as an internal standard to access the conversion and yield of the reaction. The product was purified by either preparative TLC or silica gel chromatography using EtOAc/pentane mixtures as eluting solvents.

- **Preparation of Anilines**

1-(4-aminophenyl)ethan-1-one (**2a**)[1]

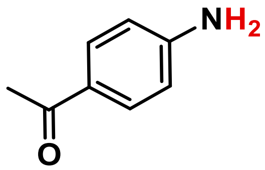

Compound **2a** was prepared by following the general procedure for aniline synthesis using 1-(4-nitrophenyl)ethan-1-one (82.6 mg, 0.5 mmol) as substrate.

Formula: C_8H_9NO.

Molecular weight: 135.17.

Isolated yield: 87% (58.8 mg, 0.43 mmol). The crude product was purified by preparative TLC.

R_f: 0.4 (EtOAc/pentane, 20:80).

^{1}H NMR (400 MHz, $CDCl_3$, 296 K): δ 7.80 (d, J = 8.7 Hz, 2H, Ar-H), 6.64 (d, J = 8.7 Hz, 2H, Ar-H), 4.18 (bs, 2H, NH_2), 2.50 (s, 3H, $COCH_3$).

^{13}C{^{1}H} NMR (101 MHz, $CDCl_3$, 296 K): δ 195.5, 150.2, 129.8, 126.8, 112.7, 25.1.

Aniline (**2b**)[2]

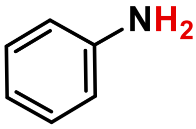

Compound **2b** was prepared by following a modified version of the general procedure for aniline synthesis using nitrobenzene (51.3 µL, 0.5 mmol) as substrate. Modifications: the temperature was increased to 80 °C and the pressure to 5 bar.

Formula: C_6H_7N.

Molecular weight: 93.13.

Isolated yield: 71% (33.1 mg, 0.35 mmol). The crude product was purified by preparative TLC.

R_f: 0.2 (EtOAc/pentane, 10:90).

^{1}H NMR (400 MHz, $CDCl_3$, 296 K): δ 7.12 – 7.06 (m, 2H, Ar-H), 6.70 (tt, *J* = 7.3, 1.1 Hz, 1H, Ar-H), 6.67 – 6.60 (m, 2H, Ar-H), 3.66 (s, 2H, NH_2).

^{13}C{^{1}H} NMR (101 MHz, $CDCl_3$, 296 K): δ 146.2, 129.3, 118.7, 115.2.

4-Bromoaniline (**2c**)[3]

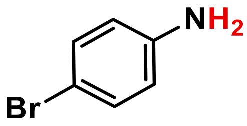

Compound **2c** was prepared by following the general procedure for aniline synthesis using 1-bromo-4-nitrobenzene (101.0 mg, 0.5 mmol) as substrate.

Formula: C_6H_6BrN.

Molecular weight: 172.03.

Isolated yield: 50% (43.0 mg, 0.25 mmol). The crude product was purified by preparative TLC.

R_f: 0.32 (EtOAc/pentane, 20:80).

^{1}H NMR (400 MHz, $CDCl_3$, 296 K): δ 7.23 (d, *J* = 8.7 Hz, 2H, Ar-H), 6.56 (d, *J* = 8.7 Hz, 2H, Ar-H), 3.66 (bs, 2H, NH_2).

^{13}C{^{1}H} NMR (101 MHz, $CDCl_3$, 296 K): δ 145.4, 132.0, 116.7, 110.2.

4-chloroaniline (**2d**)[3]

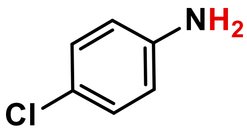

Compound **2d** was prepared by following the general procedure for aniline synthesis using 1-chloro-4-nitrobenzene (78.8 mg, 0.5 mmol) as substrate.

Formula: C_6H_6ClN.

Molecular weight: 127.57.

Isolated yield: 46% (29.3 mg, 0.23 mmol). The crude product was purified by preparative TLC.

R_f: 0.35 (EtOAc/pentane, 20:80).

^{1}H NMR (400 MHz, $CDCl_3$, 296 K): δ 7.10 (d, *J* = 8.6 Hz, 2H, Ar-H), 6.61 (d, *J* = 8.7 Hz, 2H, Ar-H), 3.67 (bs, 2H, NH_2).

^{13}C{^{1}H} NMR (101 MHz, $CDCl_3$, 296 K): δ 144.9, 129.1, 123.2, 116.3.

4-aminobenzonitrile (**2e**)[4]

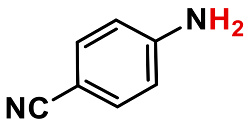

Compound **2e** was prepared by following the general procedure for aniline synthesis using 4-nitrobenzonitrile (74.1 mg, 0.5 mmol) as substrate.

Formula: $C_7H_6N_2$.

Molecular weight: 118.14.

Isolated yield: 68% (40.2 mg, 0.34 mmol). The crude product was purified by preparative TLC.

R$_f$: 0.22 (EtOAc/pentane, 20:80).

^{1}H NMR (400 MHz, CDCl$_3$, 296 K): δ 7.41 (d, *J* = 8.7 Hz, 2H, Ar-H), 6.64 (d, *J* = 8.7 Hz, 2H, Ar-H), 4.17 (bs, 2H, NH_2).

^{13}C{^{1}H} NMR (101 MHz, CDCl$_3$, 296 K): δ 150.5, 133.9, 120.3, 114.6, 100.3.

4-(trifluoromethyl)aniline (**2f**)[5]

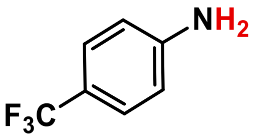

Compound **2f** was prepared by following the general procedure for aniline synthesis using 1-nitro-4-(trifluoromethyl)benzene (95.5 mg, 0.5 mmol) as substrate.

Formula: $C_7H_6F_3N$.

Molecular weight: 161.13.

Isolated yield: 54% (43.5 mg, 0.27 mmol). The crude product was purified by preparative TLC.

R$_f$: 0.22 (EtOAc/pentane, 15:85).

^{1}H NMR (400 MHz, CDCl$_3$, 296 K): δ 7.31 (d, *J* = 8.3 Hz, 2H, Ar-H), 6.61 (d, *J* = 8.3 Hz, 2H, Ar-H), 3.87 (s, 2H, NH_2).

^{13}C{^{1}H} NMR (101 MHz, CDCl$_3$, 296 K): δ 149.3, 126.7 (q, *J* = 3.9 Hz), 124.8 (d, *J* = 270.4 Hz), 120.2 (d, *J* = 32.6 Hz), 114.2.

^{19}F NMR (376 MHz, CDCl$_3$, 296 K): δ -61.22.

Methyl 4-aminobenzoate (**2g**)[6]

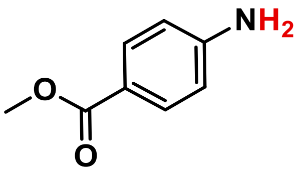

Compound **2g** was prepared by following the general procedure for aniline synthesis using methyl 4-nitrobenzoate (90.6 mg, 0.5 mmol) as substrate.

Formula: $C_8H_9NO_2$.

Molecular weight: 151.17.

Isolated yield: 91% (68.8 mg, 0.45 mmol). The crude product was purified by preparative TLC.

R$_f$: 0.46 (EtOAc/pentane, 30:70).

^{1}H NMR (400 MHz, $CDCl_3$, 296 K): δ 7.85 (d, J = 8.6 Hz, 2H, Ar-H), 6.63 (d, J = 8.6 Hz, 2H, Ar-H), 3.70-4.40 (bs, 2H, NH_2), 3.85 (s, 3H, CO_2CH_3).

^{13}C{^{1}H} NMR (101 MHz, $CDCl_3$, 296 K): δ 166.2, 149.8, 130.6, 118.7, 112.8, 50.6.

Ethyl 4-aminobenzoate (**2h**)[3]

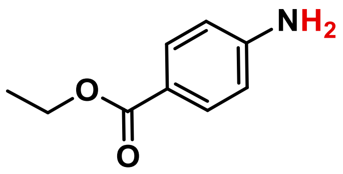

Compound **2h** was prepared by following the general procedure for aniline synthesis using ethyl 4-nitrobenzoate (97.6 mg, 0.5 mmol) as substrate.

Formula: $C_9H_{11}NO_2$.

Molecular weight: 165.19.

Isolated yield: 59% (48.7 mg, 0.29 mmol). The crude product was purified by preparative TLC.

R_f: 0.41 (EtOAc/pentane, 30:70).

^{1}H NMR (400 MHz, $CDCl_3$, 296 K): δ 7.86 (d, *J* = 8.6 Hz, 2H, Ar-H), 6.64 (d, *J* = 8.7 Hz, 2H, Ar-H), 4.31 (q, *J* = 7.1 Hz, 2H, CO_2CH_2), 4.22 – 4.07 (bs, 2H, NH_2), 1.36 (t, *J* = 7.1 Hz, 3H, $CO_2CH_2CH_3$).

^{13}C{^{1}H} NMR (101 MHz, $CDCl_3$, 296 K): δ 166.9, 150.8, 131.7, 120.3, 113.9, 60.5, 14.6.

Methyl 3-aminobenzoate (**2i**)[7]

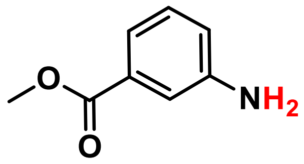

Compound **2i** was prepared by following the general procedure for aniline synthesis using methyl 3-nitrobenzoate (90.6 mg, 0.5 mmol) as substrate.

Formula: $C_9H_{11}NO_2$.

Molecular weight: 165.19.

Conversion: >99%.

NMR yield: >99%.

4-amino-N-propylbenzamide (**2j**)

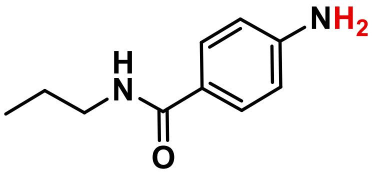

Compound **2j** was prepared by following the general procedure for aniline synthesis using 4-nitro-*N*-propylbenzamide (104.1 mg, 0.5 mmol) as substrate.

Formula: $C_{10}H_{14}N_2O$.

Molecular weight: 178.24.

Isolated yield: 78% (69.5 mg, 0.39 mmol). The crude product was purified by preparative TLC.

R_f: 0.63 (EtOAc/pentane, 40:60).

^{1}H NMR (400 MHz, $CDCl_3$, 296 K): δ 7.59 (d, J = 8.6 Hz, 2H, Ar-H), 6.63 (d, J = 8.6 Hz, 2H, Ar-H), 6.11 (bs, 1H, NH), 3.96 (bs, 2H, NH_2), 3.36 (dd, J = 13.7, 6.4 Hz, 2H, $CONHCH_2$), 1.60 (q, J = 7.3 Hz, 2H $CONHCH_2CH_2$), 0.95 (t, J = 7.4 Hz, 3H $CONHCH_2CH_2CH_3$).

^{13}C{^{1}H} NMR (101 MHz, $CDCl_3$, 296 K): δ 167.4, 149.5, 128.6, 124.3, 114.1, 41.6, 23.1, 11.5.

HRMS (ESI$^+$): Calcd. (%) for ($C_{10}H_{14}N_2O$): 179.11788; Found: 179.11795.

4-amino-N,N-dimethylbenzamide (**2k**)[8]

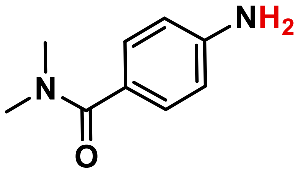

Compound **2k** was prepared by following the general procedure for aniline synthesis using *N*,*N*-dimethyl-4-nitrobenzamide (97.1 mg, 0.5 mmol) as substrate.

Formula: $C_9H_{12}N_2O$.

Molecular weight: 164.21.

Isolated yield: 82% (67.3 mg, 0.41 mmol). The crude product was purified by preparative TLC.

R_f: 0.43 (EtOAc/pentane, 30:70).

^{1}H NMR (400 MHz, $CDCl_3$, 296 K): δ 7.23 (d, *J* = 8.5 Hz, 2H, Ar-H), 6.60 (d, *J* = 8.5 Hz, 2H, Ar-H), 3.84 (s, 2H, NH_2), 3.02 (s, 6H, 2*$CONCH_3$).

^{13}C{^{1}H} NMR (101 MHz, $CDCl_3$, 296 K): δ 172.0, 148.1, 129.3, 125.6, 114.2, 39.9 (br), 35.7 (br).

4-amino-N,N-diethylbenzamide (**2l**)[9]

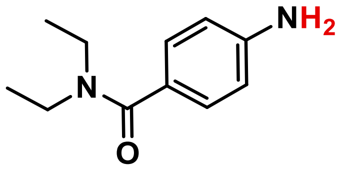

Compound **2l** was prepared by following the general procedure for aniline synthesis using *N,N*-diethyl-4-nitrobenzamide (111.1 mg, 0.5 mmol) as substrate.

Formula: $C_{11}H_{16}N_2O$.

Molecular weight: 192.26.

Isolated yield: 75% (72.1 mg, 0.37 mmol). The crude product was purified by preparative TLC.

R_f: 0.38 (EtOAc/pentane, 30:70).

^{1}H NMR (400 MHz, $CDCl_3$, 296 K): δ 7.18 (d, *J* = 8.5 Hz, 2H, Ar-H), 6.62 (d, *J* = 8.4 Hz, 2H, Ar-H), 3.82 (s, 2H, NH_2), 3.39 (s, 4H, 2*$CONCH_2$), 1.15 (t, *J* = 7.2 Hz, 6H, 2*$CONCH_2CH_3$).

^{13}C{^{1}H} NMR (101 MHz, $CDCl_3$, 296 K): δ 171.7, 147.5, 128.3, 126.9, 114.4, 43.5, 40.2, 13.7.

Quinolin-6-amine (**2m**)[6]

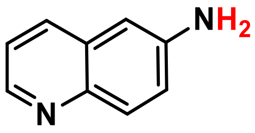

Compound **2m** was prepared by following the general procedure for aniline synthesis using 6-nitroquinoline (87.1 mg, 0.5 mmol) as substrate.

Formula: $C_9H_8N_2$.

Molecular weight: 144.18.

Conversion: 59%.

NMR yield: 50%.

Quinolin-8-amine (**2n**)[10]

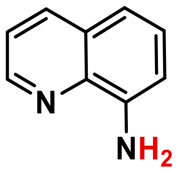

Compound **2n** was prepared by following the general procedure for aniline synthesis using 8-nitroquinoline (87.1 mg, 0.5 mmol) as substrate.

Formula: $C_9H_8N_2$.

Molecular weight: 144.18.

Isolated yield: 86% (61.2 mg, 0.43 mmol). The crude product was purified by preparative TLC.

R_f: 0.47 (EtOAc/pentane, 30:70).

^{1}H NMR (400 MHz, DMSO-*d*$_6$, 296 K): δ 8.72 (dd, *J* = 4.1, 1.7 Hz, 1H, Ar-H), 8.16 (dd, *J* = 8.3, 1.7 Hz, 1H, Ar-H), 7.44 (dd, *J* = 8.3, 4.1 Hz, 1H,

Ar-H), 7.29 (t, J = 7.8 Hz, 1H, Ar-H), 7.05 (dd, J = 8.1, 1.3 Hz, 1H, Ar-H), 6.88 (dd, J = 7.5, 1.3 Hz, 1H, Ar-H), 5.93 (bs, 2H, NH_2).

$^{13}C\{^1H\}$ NMR (101 MHz, DMSO-d_6, 296 K): δ 147.0, 145.2, 137.4, 135.8, 128.6, 127.6, 121.4, 113.7, 108.7.

3-(methylsulfonyl)aniline (**2o**)[3]

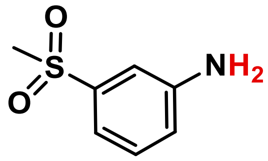

Compound **2o** was prepared by following the general procedure for aniline synthesis using 1-(methylsulfonyl)-3-nitrobenzene (100.6 mg, 0.5 mmol) as substrate.

Formula: $C_7H_9NO_2S$.

Molecular weight: 171.21.

Isolated yield: 70% (59.9 mg, 0.35 mmol). The crude product was purified by preparative TLC.

R_f: 0.58 (EtOAc/pentane, 30:70).

^1H NMR (400 MHz, $CDCl_3$, 296 K): δ 7.31 – 7.13 (m, 3H, Ar-H), 6.86 (ddd, J = 7.9, 2.4, 1.2 Hz, 1H, Ar-H), 4.01 (bs, 2H, NH_2), 3.00 (s, 3H, SO_2CH_3).

$^{13}C\{^1H\}$ NMR (101 MHz, $CDCl_3$, 296 K): δ 147.7, 141.2, 130.3, 119.7, 116.4, 112.7, 44.4.

Naphthalen-1-amine (**2p**)[1]

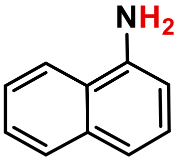

Compound **2p** was prepared by following the general procedure for aniline synthesis using 1-nitronaphthalene (86.6 mg, 0.5 mmol) as substrate.

Formula: $C_{10}H_9N$.

Molecular weight: 143.19.

Isolated yield: 51% (36.5 mg, 0.25 mmol). The crude product was purified by preparative TLC.

R_f: 0.53 (EtOAc/pentane, 30:70).

^1H NMR (400 MHz, $CDCl_3$, 296 K): δ 7.82 (ddt, J = 7.5, 4.7, 2.2 Hz, 2H, Ar-H), 7.51 – 7.43 (m, 2H, Ar-H), 7.36 – 7.27 (m, 2H, Ar-H), 6.79 (dd, J = 6.8, 1.7 Hz, 1H, Ar-H), 4.15 (bs, 2H, NH_2).

$^{13}C\{^1H\}$ NMR (101 MHz, $CDCl_3$, 296 K): δ 141.0, 133.3, 127.5, 125.3, 124.8, 123.8, 122.6, 119.7, 118.0, 108.7.

4-ethylaniline (**2q**)[11]

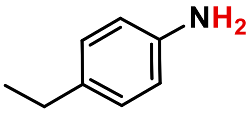

Compound **2q** was prepared by following a modified version of the general procedure for aniline synthesis using 1-ethyl-4-nitrobenzene (68.7 µL, 0.5 mmol) as substrate. Modifications: the temperature was increased to 80 °C and the pressure to 5 bar.

Formula: $C_8H_{11}N$.

Molecular weight: 121.18.

Isolated yield: 55% (33.3 mg, 0.27 mmol). The crude product was purified by preparative TLC.

R_f: 0.25 (EtOAc/pentane, 15:85).

^{1}H NMR (400 MHz, $CDCl_3$, 296 K): δ 7.02 (d, *J* = 8.3 Hz, 2H, Ar-H), 6.73 (d, *J* = 8.3 Hz, 2H, Ar-H), 4.04 (s, 2H, NH_2), 2.55 (q, *J* = 7.6 Hz, 2H, CH_2), 1.19 (t, *J* = 7.6 Hz, 3H, CH_3).

^{13}C{^{1}H} NMR (101 MHz, $CDCl_3$, 296 K): δ 142.2, 135.9, 128.8, 116.3, 28.2, 16.0.

4-Propylaniline (**2r**)[6]

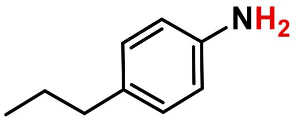

Compound **2r** was prepared by following a modified version of the general procedure for aniline synthesis using 1-nitro-4-propylbenzene (75.1 µL, 0.5 mmol) as substrate. Modifications: the temperature was increased to 80 °C and the pressure to 5 bar.

Formula: $C_9H_{13}N$.

Molecular weight: 135.21.

Isolated yield: 44% (29.7 mg, 0.22 mmol). The crude product was purified by preparative TLC.

R_f: 0.23 (EtOAc/pentane, 15:85).

^{1}H NMR (400 MHz, $CDCl_3$, 296 K): δ 6.99 (d, *J* = 8.3 Hz, 2H, Ar-H), 6.68 (d, *J* = 8.3 Hz, 2H, Ar-H), 3.73 (bs, 2H, NH_2), 2.48 (dd, *J* = 8.5, 6.7 Hz, 2H, CH_2), 1.58 (dq, *J* = 14.8, 7.4 Hz, 2H, CH_2CH_2), 0.92 (t, *J* = 7.3 Hz, 3H, $CH_2CH_2CH_3$).

^{13}C{^{1}H} NMR (101 MHz, $CDCl_3$, 296 K): δ 142.9, 133.7, 129.3, 115.7, 37.2, 24.8, 13.8.

N-phenylbenzene-1,4-diamine (**2s**)[12]

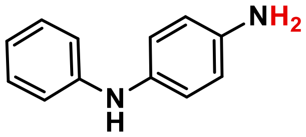

Compound **2s** was prepared by following a modified version of the general procedure for aniline synthesis using 4-nitro-*N*-phenylaniline (107.1 mg, 0.5 mmol) as substrate. Modifications: the temperature was increased to 80 °C and the pressure to 5 bar.

Formula: $C_{12}H_{12}N_{2}$.

Molecular weight: 184.24.

Isolated yield: 39% (35.9 mg, 0.19 mmol). The crude product was purified by preparative TLC.

R_f: 0.39 (EtOAc/pentane, 30:70).

^{1}H NMR (400 MHz, $CDCl_3$, 296 K): δ 7.20 (dd, J = 8.7, 7.3 Hz, 2H, Ar-H), 7.04 – 6.94 (m, 2H, Ar-H), 6.94 – 6.83 (m, 2H, Ar-H), 6.81 (td, J = 7.3, 1.2 Hz, 1H, Ar-H), 6.72 – 6.63 (m, 2H, Ar-H), 5.42 (bs, 1H, NH), 3.51 (bs, 2H, NH_2).

$^{13}C\{^1H\}$ NMR (101 MHz, $CDCl_3$, 296 K): δ 144.8, 141.1, 132.8, 128.2, 122.3, 118.0, 115.1, 114.0.

5-isopropyl-2-methylaniline (**2t**)[6]

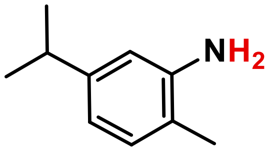

Compound **2t** was prepared by following a modified version of the general procedure for aniline synthesis using 4-isopropyl-1-methyl-2-nitrobenzene (83.7 μL, 0.5 mmol) as substrate. Modifications: the temperature was increased to 80 °C and the pressure to 5 bar.

Formula: $C_{10}H_{15}N$.

Molecular weight: 149.24.

Isolated yield: 57% (42.5 mg, 0.28 mmol). The crude product was purified by preparative TLC.

R_f: 0.42 (EtOAc/pentane, 20:80).

^{1}H NMR (400 MHz, $CDCl_3$, 296 K): δ 7.00 (d, *J* = 7.6 Hz, 1H, Ar-H), 6.62 (dd, *J* = 7.6, 1.8 Hz, 1H, Ar-H), 6.59 (s, 1H, Ar-H), 3.64 (s, 2H, NH_2), 2.82 (pd, *J* = 6.9, 2.6 Hz, 1H, CH), 2.16 (s, 3H, CH_3), 1.24 (dd, *J* = 6.9, 3.4 Hz, 6H, 2*$CHCH_3$).

$^{13}C\{^1H\}$ NMR (101 MHz, $CDCl_3$, 296 K): δ 146.9, 143.3, 129.3, 118.8, 115.7, 112.1, 32.8, 23.0, 15.9.

2-propylaniline (**2u**)[13]

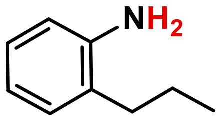

Compound **2u** was prepared by following a modified version of the general procedure for aniline synthesis using 1-nitro-2-propylbenzene (76.3 µL, 0.5 mmol) as substrate. Modifications: the temperature was increased to 100 °C and the pressure to 5 bar.

Formula: $C_9H_{13}N$.

Molecular weight: 135.21.

Conversion: 67%.

NMR yield: 56%.

4-styrylaniline (**2v**)[14]

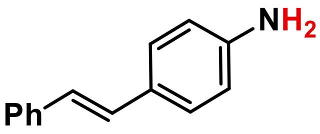

Compound **2v** was prepared by following the general procedure for aniline synthesis using 1-nitro-4-styrylbenzene (112.6 mg, 0.5 mmol) as substrate.

Formula: $C_{14}H_{13}N$.

Molecular weight: 195.27.

Isolated yield: 75% (73.2 mg, 0.37 mmol). The crude product was purified by preparative TLC.

R_f: 0.60 (EtOAc/pentane, 30:70).

^{1}H NMR (400 MHz, $CDCl_3$, 296 K): δ 7.49 (d, *J* = 7.6 Hz, 2H, Ar-H), 7.35 (dd, *J* = 8.1, 6.0 Hz, 4H, Ar-H), 7.23 (t, *J* = 7.5 Hz, 1H, Ar-H), 7.05 (d, *J* = 16.3 Hz, 1H, double bond), 6.94 (d, *J* = 16.3 Hz, 1H, double bond), 6.69 (d, *J* = 8.1 Hz, 2H, Ar-H), 3.81 – 3.62 (bs, 2H, NH_2).

^{13}C{^{1}H} NMR (101 MHz, $CDCl_3$, 296 K): δ 145.1, 136.9, 127.6, 127.6, 127.0, 126.7, 125.9, 125.1, 124.1, 114.2.

o-Toluidine (**2w**)[6]

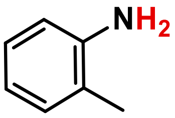

Compound **2w** was prepared by following a modified version of the general procedure for aniline synthesis using 1-methyl-2-nitrobenzene (59.1 µL, 0.5 mmol) as substrate. Modifications: the temperature was increased to 80 °C and the pressure to 5 bar.

Formula: C_7H_9N.

Molecular weight: 107.16.

Isolated yield: 21% (11.2 mg, 0.11 mmol). The crude product was purified by preparative TLC.

R_f: 0.28 (EtOAc/pentane, 15:85).

^{1}H NMR (400 MHz, CDCl$_3$, 296 K): δ 7.04 (t, *J* = 7.6 Hz, 2H, Ar-H), 6.76 – 6.64 (m, 2H, Ar-H), 3.60 (s, 2H, NH_2), 2.18 (s, 3H, CH_3).

^{13}C{^{1}H} NMR (101 MHz, CDCl$_3$, 296 K): δ 144.6, 130.5, 127.0, 122.3, 118.7, 114.9, 17.4.

9H-fluoren-2-amine (**2x**)[15]

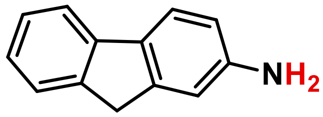

Compound **2x** was prepared by following a modified version of the general procedure for aniline synthesis using 2-nitro-9H-fluorene (105.6 mg, 0.5 mmol) as substrate. Modifications: the temperature was increased to 80 °C.

Formula: $C_{13}H_{11}N$.

Molecular weight: 181.24.

Isolated yield: 58% (52.5 mg, 0.29 mmol). The crude product was purified by preparative TLC.

R$_f$: 0.35 (EtOAc/pentane, 30:70).

^{1}H NMR (400 MHz, CDCl$_3$, 296 K): δ 7.64 (d, *J* = 7.5 Hz, 1H, Ar-H), 7.57 (d, *J* = 8.0 Hz, 1H, Ar-H), 7.47 (d, *J* = 7.5 Hz, 1H, Ar-H), 7.32 (t, *J* = 7.4 Hz, 1H, Ar-H), 7.19 (t, *J* = 7.4 Hz, 1H, Ar-H), 6.88 (d, *J* = 2.2 Hz, 1H, Ar-H), 6.71 (dd, *J* = 7.9, 2.2 Hz, 1H, Ar-H), 3.81 (s, 2H), 3.58-4.01 (bs, 2H, NH_2).

^{13}C{^{1}H} NMR (101 MHz, CDCl$_3$, 296 K): δ 145.9, 145.3, 142.4, 142.3, 133.1, 126.8, 125.2, 124.9, 120.8, 118.7, 114.1, 111.9, 37.0.

2-amino-9H-fluoren-9-one (**2y**)[2]

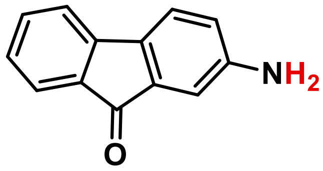

Compound **2y** was prepared by following a modified version of the general procedure for aniline synthesis using 2-nitro-9H-fluoren-9-one (112.6 mg, 0.5 mmol) as substrate. Modifications: the temperature was increased to 80 °C.

Formula: $C_{13}H_9NO$.

Molecular weight: 195.22.

Isolated yield: 67% (65.4 mg, 0.33 mmol). The crude product was purified by preparative TLC.

R$_f$: 0.27 (EtOAc/pentane, 30:70).

^{1}H NMR (400 MHz, CDCl$_3$, 296 K): δ 7.47 (d, *J* = 7.3 Hz, 1H, Ar-H), 7.31 (td, *J* = 7.4, 1.2 Hz, 1H, Ar-H), 7.24 (d, *J* = 7.4 Hz, 1H, Ar-H), 7.18 (d, *J* = 7.9 Hz, 1H, Ar-H), 7.05 (td, *J* = 7.4, 1.0 Hz, 1H, Ar-H), 6.88 (d, *J* = 2.3 Hz, 1H, Ar-H), 6.63 (dd, *J* = 7.9, 2.3 Hz, 1H, Ar-H), 3.83 (s, 2H, NH_2).

$^{13}C\{^1H\}$ NMR (101 MHz, $CDCl_3$, 296 K): δ 193.4, 146.6, 144.5, 134.9, 133.8, 133.5, 133.0, 126.2, 123.1, 120.3, 118.6, 118.0, 110.0.

3,6a-dihydrofluoranthen-3-amine (**2z**)[16]

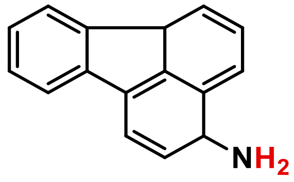

Compound **2z** was prepared by following a modified version of the general procedure for aniline synthesis using 3-nitrofluoranthene (123.6 mg, 0.5 mmol) as substrate. Modifications: the temperature was increased to 80 °C.

Formula: $C_{16}H_{13}N$.

Molecular weight: 219.29.

Isolated yield: 61% (66.9 mg, 0.30 mmol). The crude product was purified by preparative TLC.

R_f: 0.45 (EtOAc/pentane, 40:60).

^1H NMR (400 MHz, $CDCl_3$, 296 K): δ 7.88 (d, *J* = 6.9 Hz, 1H, Ar-H), 7.81 (d, *J* = 7.4 Hz, 1H, Ar-H), 7.72 (dd, *J* = 12.3, 7.9 Hz, 2H, Ar-H), 7.65 (d, *J* = 7.4 Hz, 1H, Ar-H), 7.51 (t, *J* = 7.6 Hz, 1H, Ar-H), 7.26 (t, *J* = 7.5 Hz, 1H, Ar-H), 7.23 – 7.15 (m, 1H, Ar-H), 6.69 (d, *J* = 7.5 Hz, 1H, Ar-H), 4.39 (s, 2H, NH_2).

$^{13}C\{^1H\}$ NMR (101 MHz, $CDCl_3$, 296 K): δ 144.2, 140.0, 138.4, 137.2, 133.5, 127.6, 127.5, 126.4, 125.6, 122.2, 121.5, 120.9, 120.4, 120.3, 110.5.

Pyren-1-amine (**2aa**)[17]

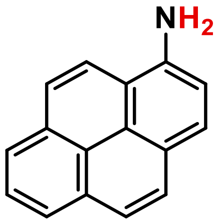

Compound **2aa** was prepared by following a modified version of the general procedure for aniline synthesis using 1-nitropyrene (124.6 mg, 0.5 mmol) as substrate. Modifications: the temperature was increased to 80 °C.

Formula: $C_{16}H_{11}N$.

Molecular weight: 217.27.

Isolated yield: 50% (54.3 mg, 0.25 mmol). The crude product was purified by preparative TLC.

R_f: 0.52 (EtOAc/pentane, 40:60).

^1H NMR (400 MHz, $CDCl_3$, 296 K): δ 8.05 (dd, *J* = 7.7, 4.5 Hz, 2H, Ar-H), 8.01 – 7.95 (m, 2H, Ar-H), 7.95 – 7.90 (m, 2H, Ar-H), 7.82 (d, *J* = 8.9 Hz, 1H, Ar-H), 7.38 (d, *J* = 8.0 Hz, 1H, Ar-H), 4.49 (s, 2H, NH_2).

$^{13}C\{^1H\}$ NMR (101 MHz, $CDCl_3$, 296 K): δ 141.0, 132.3, 131.8, 127.7, 126.2, 126.1, 125.6, 124.4, 124.2, 123.9, 123.7, 120.3, 117.0, 114.1.

3-fluoro-4-morpholinoaniline (**2ab**)[18]

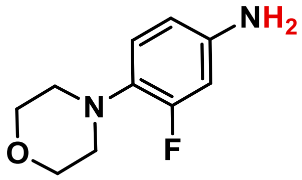

Compound **2ab** was prepared by following a modified version of the general procedure for aniline synthesis using 4-(2-fluoro-4-nitrophenyl)morpholine (113.1 mg, 0.5 mmol) as substrate. Modifications: the temperature was increased to 80 °C.

Formula: $C_{10}H_{13}FN_2O$.

Molecular weight: 196.23.

Isolated yield: 72% (70.6 mg, 0.36 mmol). The crude product was purified by preparative TLC.

R_f: 0.32 (EtOAc/pentane, 20:80).

^{1}H NMR (400 MHz, $CDCl_3$, 296 K): δ 6.78 (td, *J* = 10.0, 9.4, 2.8 Hz, 1H, Ar-H), 6.50 – 6.31 (m, 2H, Ar-H), 3.83 (dq, *J* = 5.4, 2.5 Hz, 4H, 2*Morph-CH_2), 3.58 (s, 2H, NH_2), 2.95 (dq, *J* = 5.8, 2.6 Hz, 4H, 2*Morph-CH_2).

^{13}C{^{1}H} NMR (101 MHz, $CDCl_3$, 296 K): δ 156.8 (d, *J* = 245.2 Hz), 142.9 (d, *J* = 11.3 Hz), 131.7 (d, *J* = 9.8 Hz), 120.3 (d, *J* = 4.4 Hz), 110.7 (d, *J* = 3.1 Hz), 104.0 (d, *J* = 23.4 Hz), 67.2, 51.8 (d, *J* = 2.5 Hz).

General Procedure for Hydroxylamine Synthesis

In an oven-dried Fisher-Porter tube under argon, nitroarene (0.5 mmol), complex **1** (3 mol%), and THF (3 mL) were charged. The reaction mixture was frozen using liquid nitrogen, evacuated, and then pressurized with H_2 (2 bar). The resulting mixture was stirred for 15 hours at 40 °C. The reaction vessel was cooled to ambient temperature, the pressure was released, and the solvent was removed *in vacuo*. The residue was analyzed by ^{1}H NMR spectroscopy relative to mesitylene (0.5 mmol) as an internal standard to access the conversion and yield of the reaction. The product was purified by either preparative TLC or silica gel chromatography using EtOAc/pentane mixtures as eluting solvents.

- **Preparation of Hydroxylamines**

1-(4-(hydroxyamino)phenyl)ethan-1-one (**3a**)[19]

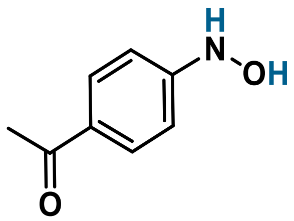

Compound **3a** was prepared by following the general procedure for hydroxylamine synthesis using 1-(4-nitrophenyl)ethan-1-one (82.6 mg, 0.5 mmol) as substrate.

Formula: $C_8H_9NO_2$.

Molecular weight: 151.17.

Isolated yield: 85% (64.2 mg, 0.42 mmol). The crude product was purified by preparative TLC.

R$_f$: 0.33 (EtOAc/pentane, 20:80).

^{1}H NMR (400 MHz, DMSO-*d*$_6$, 296 K): δ 10.64 – 8.15 (m, 2H, NHOH), 7.80 (d, *J* = 8.8 Hz, 2H, Ar-H), 6.83 (d, *J* = 8.8 Hz, 2H, Ar-H), 2.45 (s, 3H, $COCH_3$).

^{13}C{^{1}H} NMR (101 MHz, DMSO-*d*$_6$, 296 K): δ 195.7, 155.9, 129.9, 127.7, 110.9, 26.1.

N-phenylhydroxylamine (**3b**)[19]

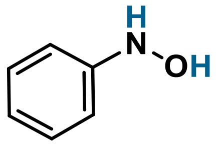

Compound **3b** was prepared by following a modified version of the general procedure for hydroxylamine synthesis using nitrobenzene (51.3 µL, 0.5 mmol) as substrate. Modifications: the temperature was increased to 60 °C and the pressure to 5 bar.

Formula: C_6H_7NO.

Molecular weight: 109.13.

Isolated yield: 57% (31.1 mg, 0.28 mmol). The crude product was purified by preparative TLC.

R$_f$: 0.29 (EtOAc/pentane, 40:60).

^{1}H NMR (400 MHz, DMSO-d_6, 296 K): δ 8.27 (d, J = 2.2 Hz, 1H, OH), 8.22 (d, J = 2.3 Hz, 1H, NH), 7.15 (dd, J = 8.5, 7.3 Hz, 2H, Ar-H), 6.88 – 6.80 (m, 2H, Ar-H), 6.76 – 6.70 (m, 1H, Ar-H).

^{13}C{^{1}H} NMR (101 MHz, DMSO-d_6, 296 K): δ 152.5, 128.9, 119.6, 113.4.

N-(4-bromophenyl)hydroxylamine (**3c**)[19]

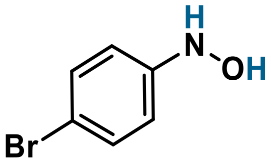

Compound **3c** was prepared by following the general procedure for hydroxylamine synthesis using 1-bromo-4-nitrobenzene (101.0 mg, 0.5 mmol) as substrate.

Formula: C_6H_6BrNO.

Molecular weight: 188.02.

Conversion: 77%.

NMR yield: 61%.

N-(4-chlorophenyl)hydroxylamine (**3d**)[19]

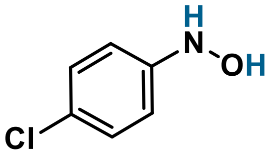

Compound **3d** was prepared by following the general procedure for hydroxylamine synthesis using 1-chloro-4-nitrobenzene (78.8 mg, 0.5 mmol) as substrate.

Formula: C_6H_6ClNO.

Molecular weight: 143.57.

Isolated yield: 51% (36.6 mg, 0.25 mmol). The crude product was purified by preparative TLC.

R_f: 0.34 (EtOAc/pentane, 20:80).

^{1}H NMR (400 MHz, DMSO-d_6, 296 K): δ 8.43 (d, J = 1.7 Hz, 1H, OH), 8.40 (d, J = 2.2 Hz, 1H, NH), 7.19 (d, J = 8.8 Hz, 2H, Ar-H), 6.82 (d, J = 8.9 Hz, 2H, Ar-H).

^{13}C{^{1}H} NMR (101 MHz, DMSO-d_6, 296 K): δ 151.0, 128.2, 122.4, 114.4.

4-(hydroxyamino)benzonitrile (**3e**)[19]

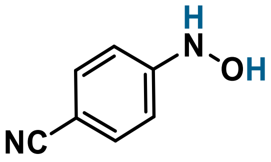

Compound **3e** was prepared by following the general procedure for hydroxylamine synthesis using 4-nitrobenzonitrile (74.1 mg, 0.5 mmol) as substrate.

Formula: $C_7H_6N_2O$.

Molecular weight: 134.14.

Isolated yield: 90% (60.4 mg, 0.45 mmol). The crude product was purified by preparative TLC.

R$_f$: 0.22 (EtOAc/pentane, 10:90).

^{1}H NMR (400 MHz, DMSO-*d*$_6$, 296 K): δ 9.11 (d, *J* = 1.7 Hz, 1H, OH), 8.76 (d, *J* = 1.6 Hz, 1H, NH), 7.55 (d, *J* = 8.7 Hz, 2H, Ar-H), 6.85 (d, *J* = 8.8 Hz, 2H, Ar-H).

^{13}C{^{1}H} NMR (101 MHz, DMSO-*d*$_6$, 296 K): δ 155.4, 133.1, 120.2, 111.8, 99.1.

N-(4-(trifluoromethyl)phenyl)hydroxylamine (**3f**)

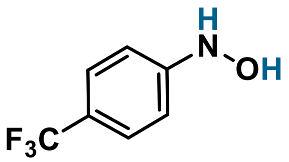

Compound **3f** was prepared by following the general procedure for hydroxylamine synthesis using 1-nitro-4-(trifluoromethyl)benzene (95.5 mg, 0.5 mmol) as substrate.

Formula: $C_7H_6F_3NO$.

Molecular weight: 177.13.

Isolated yield: 79% (70.0 mg, 0.39 mmol). The crude product was purified by preparative TLC.

R$_f$: 0.16 (EtOAc/pentane, 20:80).

^{1}H NMR (400 MHz, CDCl$_3$, 296 K): δ 7.52 (d, *J* = 8.5 Hz, 2H), 7.03 (d, *J* = 8.4 Hz, 2H), 5.98 (bs, 2H).

^{13}C{^{1}H} NMR (101 MHz, CDCl$_3$, 296 K): δ 152.6, 126.4 (q, *J* = 3.9 Hz), 125.9 (d, *J* = 1.7 Hz), 124.1 (q, *J* = 32.4 Hz), 123.2, 113.8.

HRMS (ESI$^+$) Calcd. (%) for ($C_7H_6F_3NO$): 176.03287; Found: 176.03280.

Methyl 4-(hydroxyamino)benzoate (**3g**)[19]

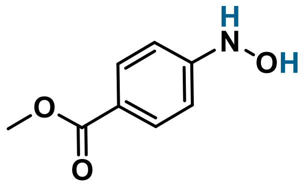

Compound **3g** was prepared by following the general procedure for hydroxylamine synthesis using methyl 4-nitrobenzoate (90.6 mg, 0.5 mmol) as substrate.

Formula: $C_8H_9NO_3$.

Molecular weight: 167.16.

Isolated yield: 91% (76.1 mg, 0.45 mmol). The crude product was purified by preparative TLC.

R$_f$: 0.41 (EtOAc/pentane, 30:70).

^{1}H NMR (400 MHz, DMSO-*d*$_6$, 296 K): δ 8.93 (s, 1H, OH), 8.65 (s, 1H, NH), 7.77 (d, *J* = 8.8 Hz, 2H, Ar-H), 6.83 (d, *J* = 8.8 Hz, 2H, Ar-H), 3.76 (s, 3H, CO_2CH_3).

$^{13}C\{^1H\}$ NMR (101 MHz, DMSO-d_6, 296 K): δ 166.3, 156.0, 130.5, 119.1, 111.2, 51.5.

Ethyl 4-(hydroxyamino)benzoate (**3h**)[20]

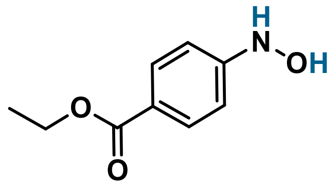

Compound **3h** was prepared by following the general procedure for hydroxylamine using ethyl 4-nitrobenzoate (97.6 mg, 0.5 mmol) as substrate.

Formula: $C_9H_{11}NO_3$.

Molecular weight: 181.19.

Isolated yield: 74% (67.0 mg, 0.37 mmol). The crude product was purified by preparative TLC.

R_f: 0.39 (EtOAc/pentane, 30:70).

^1H NMR (400 MHz, DMSO-d_6, 296 K): δ 8.95 (d, J = 1.6 Hz, 1H, OH), 8.65 (d, J = 1.6 Hz, 1H, NH), 7.77 (d, J = 8.8 Hz, 2H, Ar-H), 6.83 (d, J = 8.8 Hz, 2H, Ar-H), 4.23 (q, J = 7.1 Hz, 2H, CO_2CH_2), 1.28 (t, J = 7.1 Hz, 3H $CO_2CH_2CH_3$).

$^{13}C\{^1H\}$ NMR (101 MHz, DMSO-d_6, 296 K): δ 165.8, 156.0, 130.4, 119.4, 111.2, 59.9, 14.3.

HRMS (ESI$^+$) Calcd. (%) for ($C_9H_{11}NO_3$): 180.06662; Found: 180.06661.

Methyl 3-(hydroxyamino)benzoate (**3i**)[19]

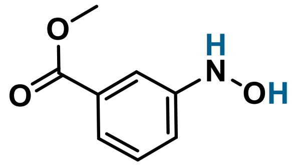

Compound **3i** was prepared by following the general procedure for hydroxylamine using methyl 3-nitrobenzoate (90.6 mg, 0.5 mmol) as substrate.

Formula: $C_8H_9NO_3$.

Molecular weight: 167.16.

Isolated yield: 87% (70.6 mg, 0.43 mmol). The crude product was purified by preparative TLC.

R_f: 0.46 (EtOAc/pentane, 30:70).

^1H NMR (400 MHz, DMSO-d_6, 296 K): δ 8.53 (d, J = 2.2 Hz, 1H, OH), 8.49 (d, J = 2.1 Hz, 1H, NH), 7.45 (t, J = 2.0 Hz, 1H, Ar-H), 7.38 – 7.26 (m, 2H, Ar-H), 7.06 (dt, J = 7.8, 1.9 Hz, 1H, Ar-H), 3.82 (s, 3H, CO_2CH_3).

$^{13}C\{^1H\}$ NMR (101 MHz, DMSO-d_6, 296 K): δ 166.6, 152.4, 129.9, 128.9, 119.9, 117.5, 113.2, 52.0.

4-(hydroxyamino)-N-propylbenzamide (**3j**)[21]

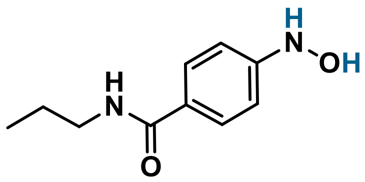

Compound **3j** was prepared by following the general procedure for hydroxylamine synthesis using 4-nitro-*N*-propylbenzamide (104.1 mg, 0.5 mmol) as substrate.

Formula: $C_{10}H_{14}N_2O_2$.

Molecular weight: 194.23

Isolated yield: 83% (80.6 mg, 0.41 mmol). The crude product was purified by preparative TLC.

R_f: 0.63 (EtOAc/pentane, 40:60).

^{1}H NMR (400 MHz, DMSO-d_6, 296 K): δ 8.64 (d, *J* = 1.9 Hz, 1H, OH), 8.49 (d, *J* = 1.9 Hz, 1H, NH), 8.13 (t, *J* = 4.7 Hz, 1H, CONH), 7.69 (d, *J* = 8.6 Hz, 2H, Ar-H), 6.81 (d, *J* = 8.7 Hz, 2H, Ar-H), 3.23 – 3.06 (m, 2H, $CONHCH_2$), 1.50 (q, *J* = 7.3 Hz, 2H $CONHCH_2CH_2$), 0.87 (t, *J* = 7.4 Hz, 3H, CH_3).

^{13}C{^{1}H} NMR (101 MHz, DMSO-d_6, 296 K): δ 166.0, 154.4, 128.0, 125.0, 111.4, 40.9, 22.6, 11.5.

4-(hydroxyamino)-N-propylbenzamide (**3k**)[19]

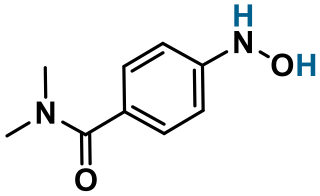

Compound **3k** was prepared by following the general procedure for hydroxylamine synthesis using *N*,*N*-dimethyl-4-nitrobenzamide (97.1 mg, 0.5 mmol) as substrate.

Formula: $C_9H_{12}N_2O_2$.

Molecular weight: 180.21.

Conversion: 34%.

NMR yield: 28%.

4-(hydroxyamino)-N-propylbenzamide (**3l**) [19]

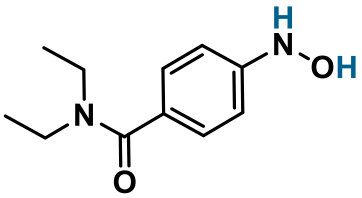

Compound **3l** was prepared by following the general procedure for hydroxylamine synthesis using *N,N*-diethyl-4-nitrobenzamide (111.1 mg, 0.5 mmol) as substrate.

Formula: $C_{11}H_{16}N_2O_2$.

Molecular weight: 208.26.

Conversion: 27%.

NMR yield: 20%.

N-(quinolin-6-yl)hydroxylamine (**3m**)[22]

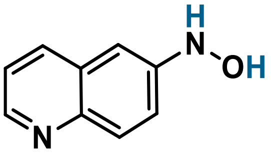

Compound **3m** was prepared by following a modified version of the general procedure for hydroxylamine synthesis using 6-nitroquinoline (87.1 mg, 0.5 mmol) as substrate. Modifications: the temperature was increased to 60 °C and the pressure to 5 bar.

Formula: $C_9H_8N_2O$.

Molecular weight: 160.18.

Isolated yield: 43% (34.4 mg, 0.21 mmol). The crude product was purified by preparative TLC.

R$_f$: 0.62 (EtOAc/pentane, 30:70).

^{1}H NMR (400 MHz, DMSO-*d*$_6$, 296 K): δ 8.72 (t, *J* = 1.4 Hz, 1H, OH), 8.63 – 8.57 (m, 2H, NH and , Ar-H), 8.18 – 8.08 (m, 1H, Ar-H), 7.79 (d, *J* = 8.9 Hz, 1H, Ar-H), 7.37 (dd, *J* = 8.3, 4.2 Hz, 1H, Ar-H), 7.27 – 7.18 (m, 2H, Ar-H).

^{13}C{^{1}H} NMR (101 MHz, DMSO-*d*$_6$, 296 K): δ 149.9, 146.6, 143.7, 134.1, 129.2, 129.1, 121.5, 119.2, 105.3.

N-(quinolin-8-yl)hydroxylamine (**3n**)[19]

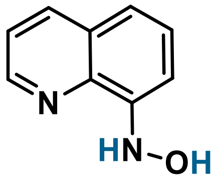

Compound **3n** was prepared by following a modified version of the general procedure for hydroxylamine synthesis using 8-nitroquinoline (87.1 mg, 0.5 mmol) as substrate. Modifications: the temperature was increased to 60 °C and the pressure to 5 bar.

Formula: $C_9H_8N_2O$.

Molecular weight: 160.18.

Conversion: >99%.

NMR yield: 85%.

N-(3-(methylsulfonyl)phenyl)hydroxylamine (**3o**)

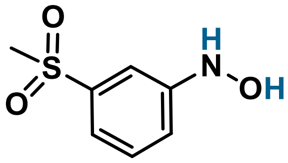

Compound **3o** was prepared by following the general procedure for hydroxylamine synthesis using 1-(methylsulfonyl)-3-nitrobenzene (100.6 mg, 0.5 mmol) as substrate.

Formula: $C_7H_9NO_3S$.

Molecular weight: 187.21.

Isolated yield: 83% (77.7 mg, 0.41 mmol). The crude product was purified by preparative TLC.

R_f: 0.58 (EtOAc/pentane, 30:70).

^{1}H NMR (400 MHz, DMSO-*d*$_6$, 296 K): δ 8.78 (bs, 1H, OH), 8.65 (bs, 1H, NH), 7.42 (t, *J* = 7.9 Hz, 1H, Ar-H), 7.33 (d, *J* = 2.1 Hz, 1H, Ar-H), 7.26 (ddd, *J* = 7.6, 1.8, 1.0 Hz, 1H, Ar-H), 7.09 (ddd, *J* = 8.2, 2.2, 1.0 Hz, 1H, Ar-H), 3.15 (s, 3H, SO_2CH_3).

^{13}C{^{1}H} NMR (101 MHz, DMSO-*d*$_6$, 296 K): δ 153.3, 141.7, 130.1, 117.7, 117.5, 110.6, 44.1.

HRMS (ESI$^+$) Calcd. (%) for ($C_7H_9NO_3S$): 186.02304; Found: 186.02305.

2-(hydroxyamino)-9H-fluoren-9-one (**3y**)

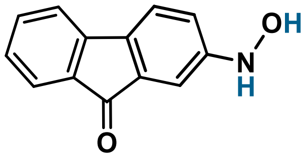

Compound **3y** was prepared by following a modified version of the general procedure for hydroxylamine synthesis using 2-nitro-9H-fluoren-9-one (112.6 mg, 0.5 mmol) as substrate.

Formula: $C_{13}H_9NO_2$.

Molecular weight: 211.22.

Conversion: 65%

NMR yield: 36%.

HRMS (ESI$^+$) Calcd. (%) for ($C_{13}H_8NO_2$): 210.056054; Found: 210.055850.

N-(3-fluorophenyl)hydroxylamine (**3ac**)[19]

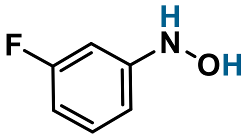

Compound **3ac** was prepared by following the general procedure for hydroxylamine synthesis using methyl 1-floro-3-nitrobenzene (53.2 µL, 0.5 mmol) as substrate.

Formula: C_6H_6FNO.

Molecular weight: 127.12.

Isolated yield: 79% (50.2 mg, 0.39 mmol). The crude product was purified by preparative TLC.

R_f: 0.26 (EtOAc/pentane, 20:80).

^{1}H NMR (400 MHz, DMSO-d_6, 296 K): δ 8.51 (d, J = 1.9 Hz, 1H, OH), 8.47 (d, J = 2.0 Hz, 1H, NH), 7.16 (td, J = 8.3, 6.5 Hz, 1H, Ar-H), 6.65 – 6.56 (m, 2H, Ar-H), 6.50 (td, J = 8.6, 8.2, 2.6 Hz, 1H, Ar-H).

^{13}C{^{1}H} NMR (101 MHz, DMSO-d_6, 296 K): δ 164.2, 161.8, 142.2 (dd, J = 2453.0, 10.0 Hz), 108.7 (d, J = 2.4 Hz), 105.1 (d, J = 21.4 Hz), 99.3 (d, J = 25.4 Hz).

N-(4-fluorophenyl)hydroxylamine (**3ad**)[19]

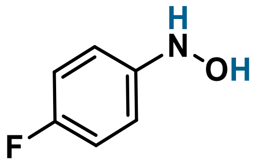

Compound **3ad** was prepared by following the general procedure for hydroxylamine synthesis using methyl 1-floro-4-nitrobenzene (53.1 µL, 0.5 mmol) as substrate.

Formula: C_6H_6FNO.

Molecular weight: 127.12.

Isolated yield: 46% (29.2 mg, 0.23 mmol). The crude product was purified by preparative TLC.

R_f: 0.24 (EtOAc/pentane, 20:80).

^{1}H NMR (400 MHz, DMSO-d_6, 296 K): δ 8.35 (d, J = 2.3 Hz, 1H, OH), 8.20 (d, J = 2.5 Hz, 1H, NH), 7.00 (t, J = 8.9 Hz, 2H, Ar-H), 6.83 (dd, J = 9.0, 4.8 Hz, 2H, Ar-H).

^{13}C{^{1}H} NMR (101 MHz, DMSO-d_6, 296 K): 156.3 (d, J = 234.0 Hz), 148.6 (d, J = 1.9 Hz), 114.9 (d, J = 22.2 Hz), 114.3 (d, J = 7.7 Hz).

N-(perfluorophenyl)hydroxylamine (**3ae**)[23]

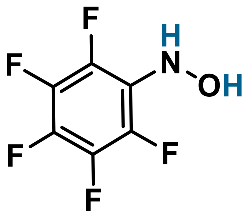

Compound **3ae** was prepared by following the general procedure for hydroxylamine synthesis using 1,2,3,4,5-pentafluoro-6-nitrobenzene (64.3 µL, 0.5 mmol) as substrate.

Formula: $C_6H_2F_5NO$.

Molecular weight: 199.08.

Isolated yield: 70% (69.7 mg, 0.35 mmol). The crude product was purified by preparative TLC.

R_f: 0.28 (EtOAc/pentane, 20:80).

^{1}H NMR (400 MHz, DMSO-d_6, 296 K): δ 9.08 (bs, 1H, OH), 8.46 (bs, 1H, NH).

^{19}F NMR (376 MHz, DMSO-d6, 296 K): δ -153.77 (dt, J = 22.0, 4.2 Hz, 2H), -164.73 (td, J = 22.6, 4.5 Hz, 2H), -166.74 (tt, J = 22.9, 4.1 Hz, 1H).

Preparative Scale Reactions

1. Benzocaine (2h)

Procedure: Compound **2h** was prepared by following the general procedure for aniline synthesis using ethyl 4-nitrobenzoate (976 mg, 5.0 mmol) as a substrate.

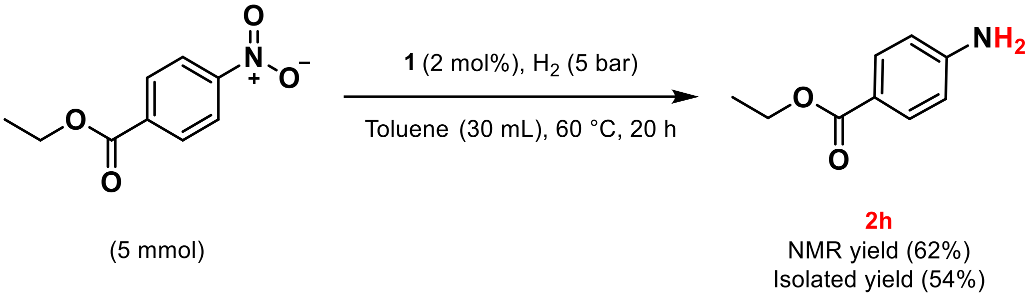

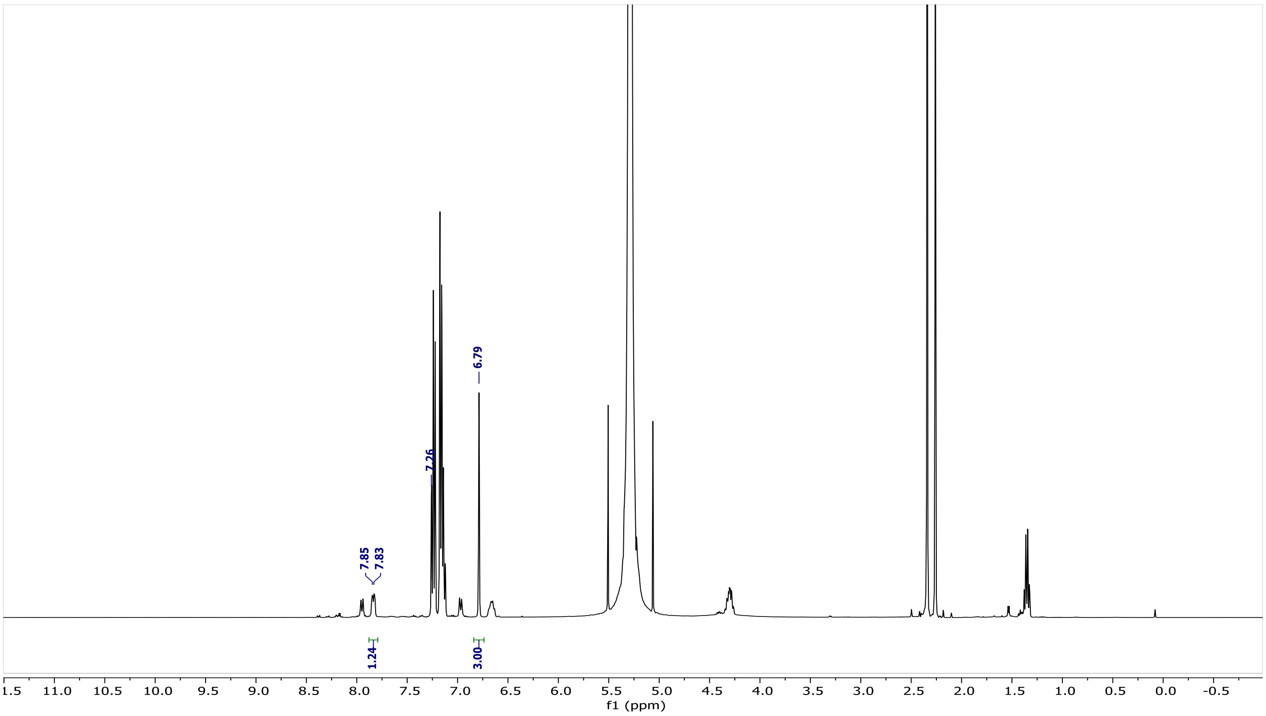

Figure S48 – ¹H NMR (400 MHz, CDCl$_3$, 296 K) crude spectrum of the reaction mixture.

2. Synthon for Linezolid (2ab)

Procedure: Compound **2ab** was prepared by following the general procedure for aniline synthesis using 4-(2-fluoro-4-nitrophenyl)morpholine (1.131 g, 5.0 mmol) as substrate.

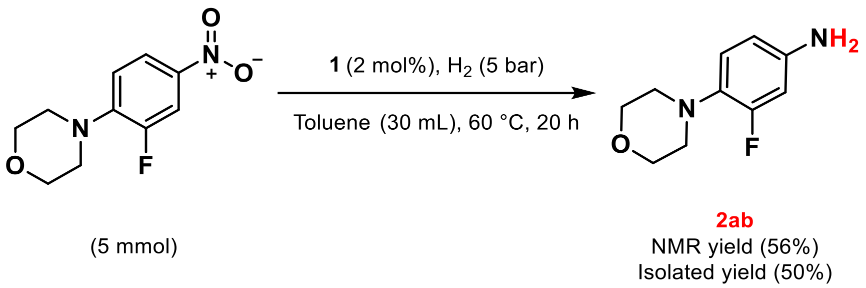

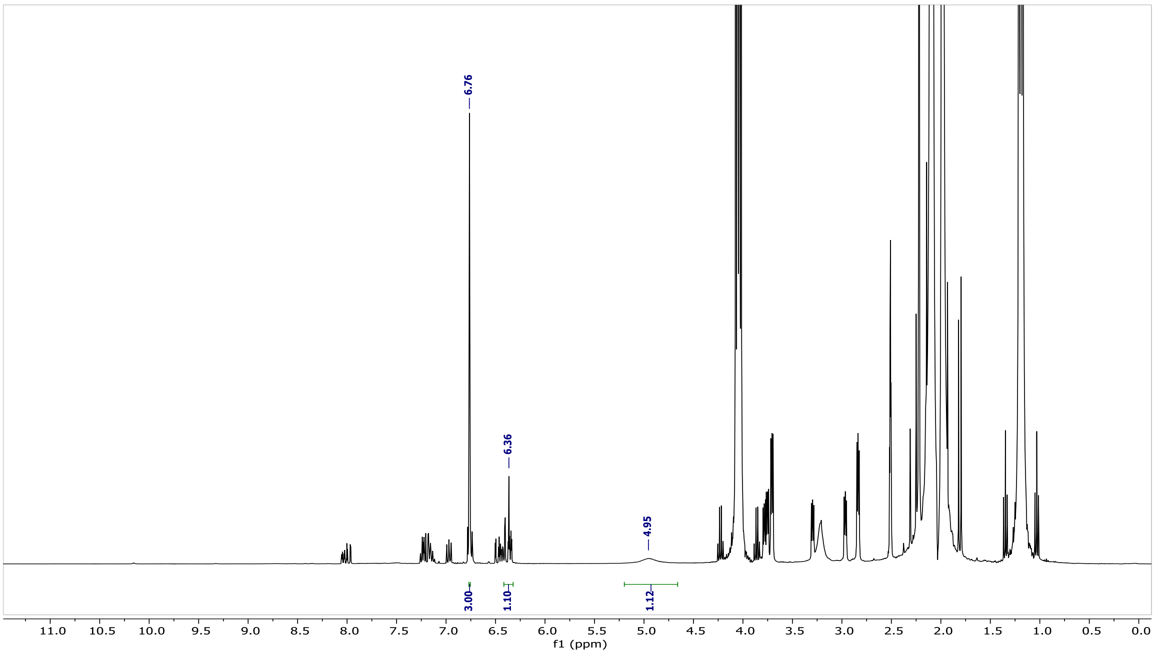

Figure S49 – ^{1}H NMR (400 MHz, DMSO-d_6, 296 K) crude spectrum of the reaction mixture.

Empirical Mechanistic Studies

1. Catalyst Screening Under Optimized Conditions

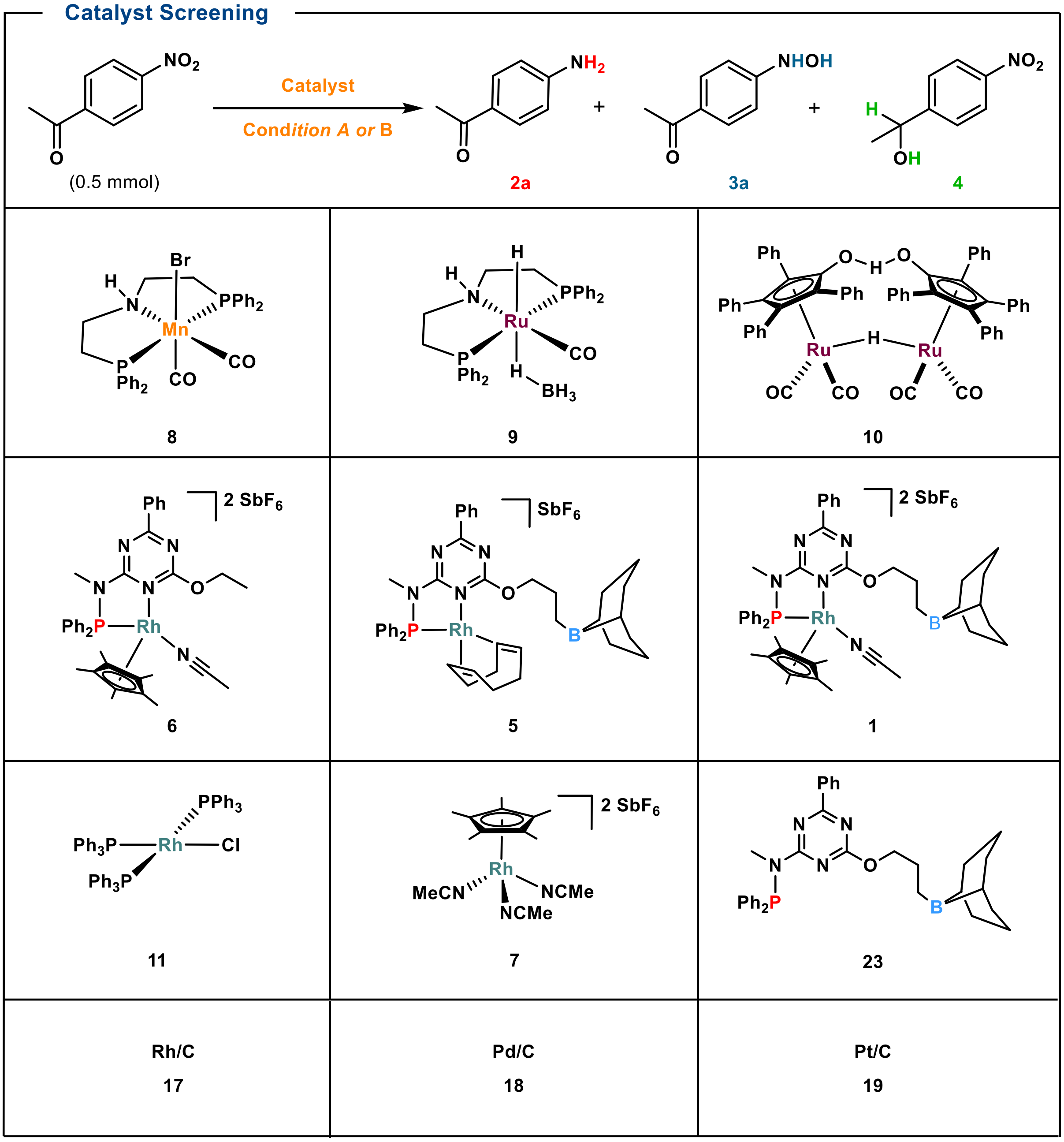

Scheme S1 – Catalysts were screened under both *conditions* ***A*** *and* ***B***. *Condition* ***A****:* catalyst (2 mol%), H_2 (2 bar), Toluene (3 mL), 60 °C, 15 h. *Condition* ***B****:* catalyst (3 mol%), H_2 (2 bar), THF (3 mL), 40 °C, 15 h.

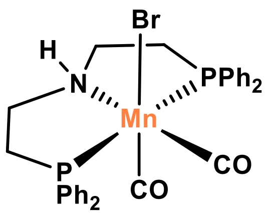

8

Complex **8** was tested by using a modified version of either the general method for aniline synthesis (*condition* ***A***) or hydroxylamine synthesis (*condition* ***B***). Modifications: KO^{t}Bu (*condition* ***A***: 2 mol%; *condition* ***B***: 3 mol%) was used to activate complex **8.**

Results:

NMR yield using *condition A*: 0:0:0.

NMR yield using *condition B*: 0:0:0.

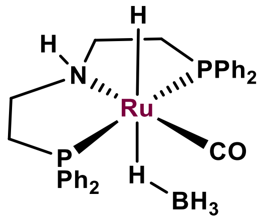

9

Complex **9** was tested by using either the general method for aniline synthesis (*condition* ***A***) or hydroxylamine synthesis (*condition* ***B***).

Results:

NMR yield using *condition* ***A***: 0:0:93.

NMR yield using *condition* ***B***: 0:0:99.

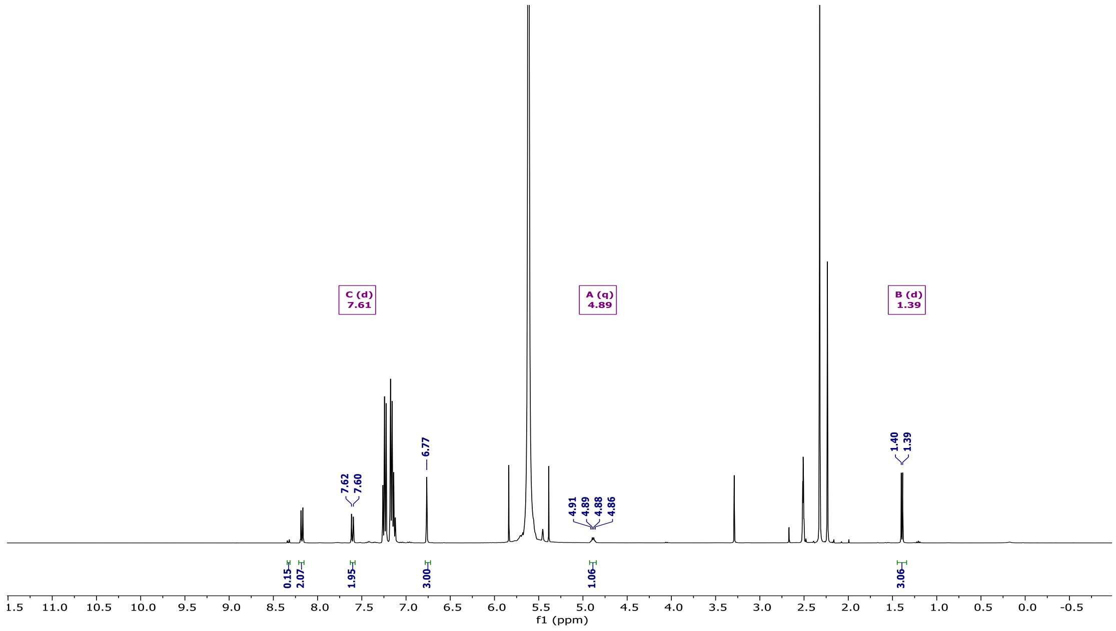

Figure S50 – ^{1}H NMR (400 MHz, DMSO-*d*$_6$, 296 K) spectrum of the reaction mixture using **9** under *condition* ***A***.

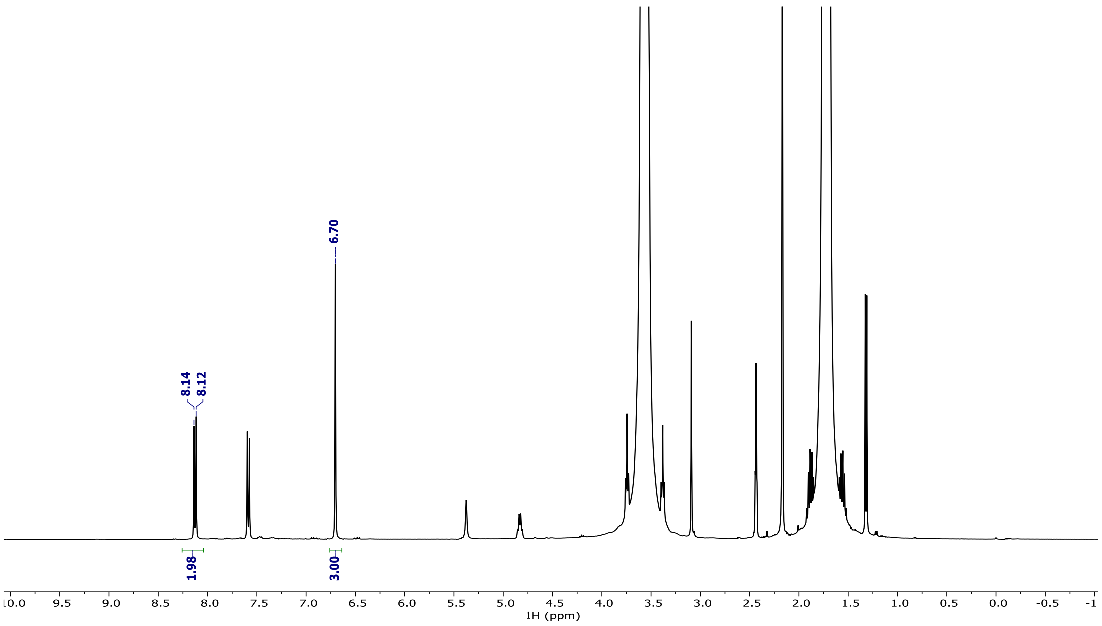

Figure S51 – ^{1}H NMR (400 MHz, DMSO-d_6, 296 K) spectrum of the reaction mixture using **9** under *condition* ***B***.

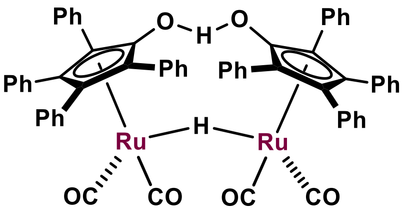

10

Complex **10** was tested by using either the general method for aniline synthesis (*condition* ***A***) or hydroxylamine synthesis (*condition* ***B***).

Results:

NMR yield using *condition* ***A***: 0:0:99.

NMR yield using *condition* ***B***: 0:0:33.

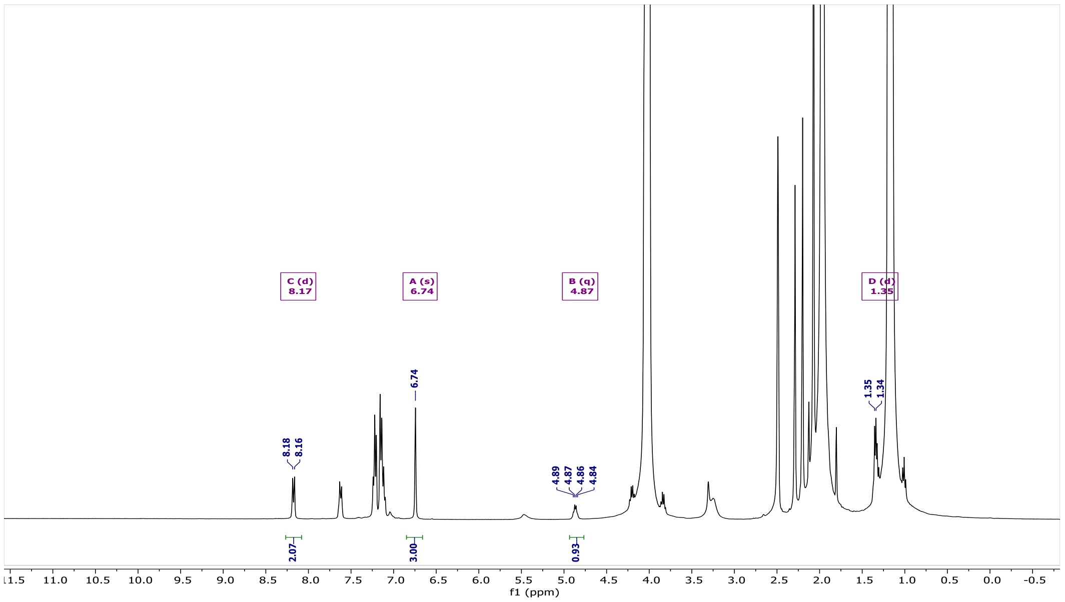

Figure S52 – ^{1}H NMR (400 MHz, DMSO-d_6, 296 K) spectrum of the reaction mixture using **10** under *condition* ***A***.

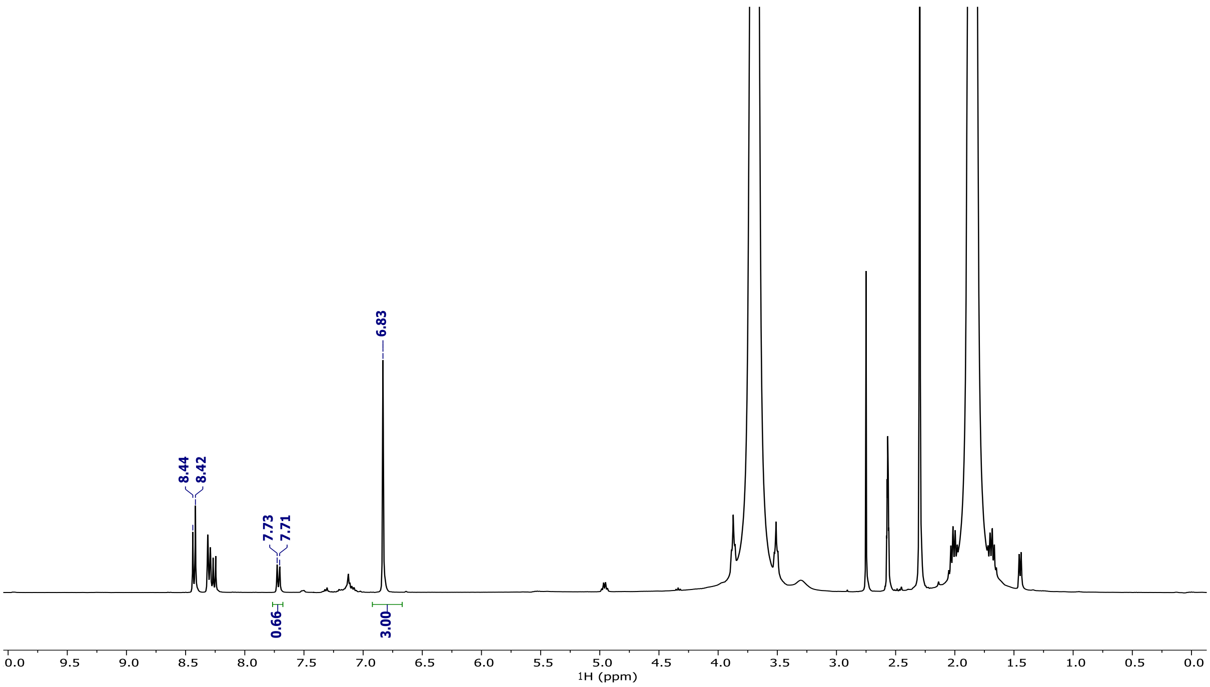

Figure S53 – ¹H NMR (400 MHz, DMSO-*d₆*, 296 K) spectrum of the reaction mixture using **10** under *condition **B***.

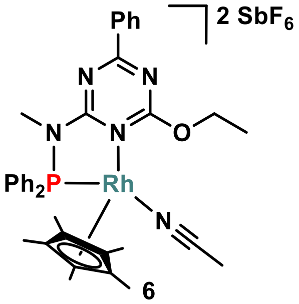

6

Complex **6** was tested by using either the general method for aniline synthesis (*condition **A***) or hydroxylamine synthesis (*condition **B***).

NMR yield using *condition **A***: 0:15:0.

NMR yield using *condition **B***: 5:88:0.

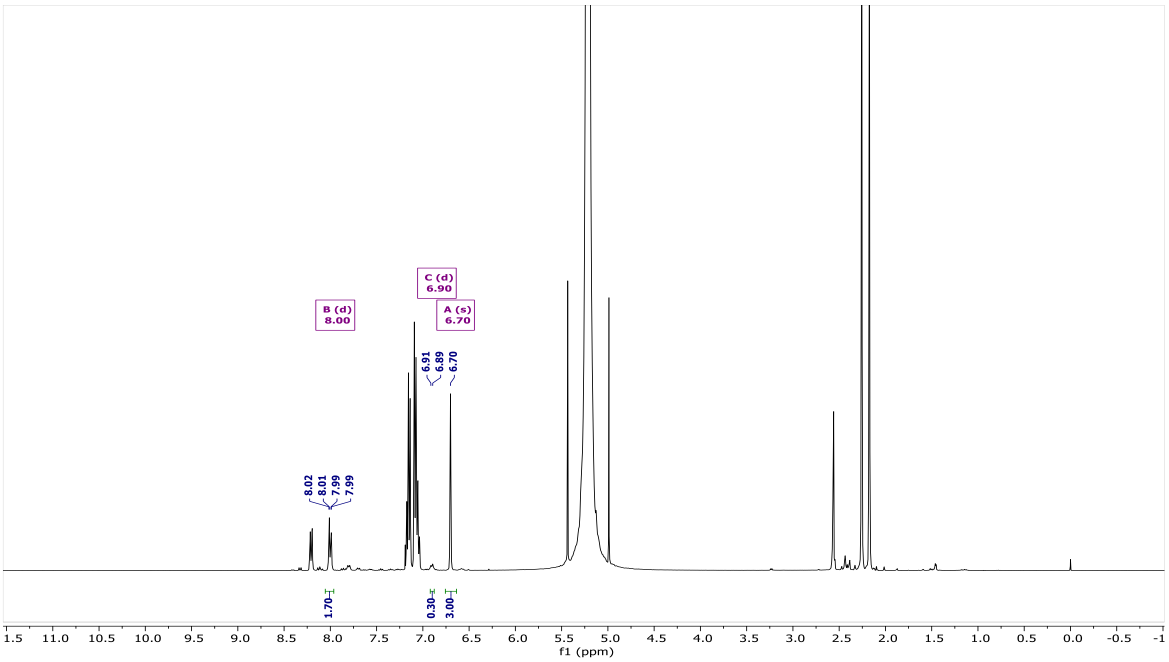

Figure S54 – ¹H NMR (400 MHz, CDCl₃, 296 K) spectrum of the reaction mixture using **6** under *condition **A***.

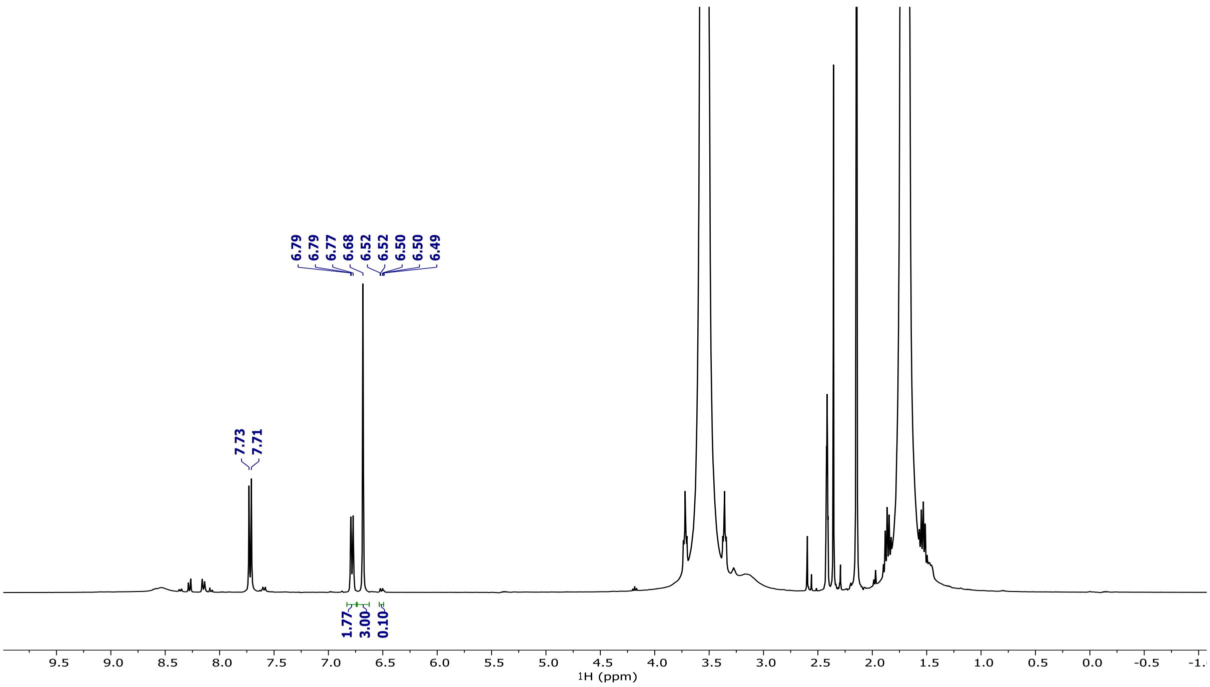

Figure S55 – ^{1}H NMR (400 MHz, DMSO-d_6, 296 K) spectrum of the reaction mixture using **6** under *condition **B***.

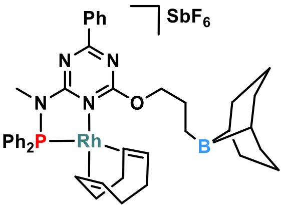

5

Complex **5** was tested by using either the general method for aniline synthesis (*condition **A***) or hydroxylamine synthesis (*condition **B***).

Results:

NMR yield using *condition **A***: 0:0:0.

NMR yield using *condition **B***: 0:0:0.

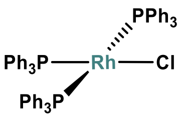

11

Wilkinson's catalyst **11** was tested by using either the general method for aniline synthesis (*condition **A***) or hydroxylamine synthesis (*condition **B***).

Results:

NMR yield using *condition **A***: 0:0:0.

NMR yield using *condition **B***: 0:0:0.

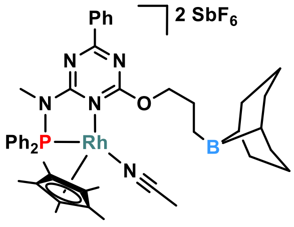

1

Complex **1** was tested by using either the general method for aniline synthesis (*condition **A***) or hydroxylamine synthesis (*condition **B***).

Results:

NMR yield using *condition **A***: 90:8:0.

NMR yield using *condition **B***: 8:91:0.

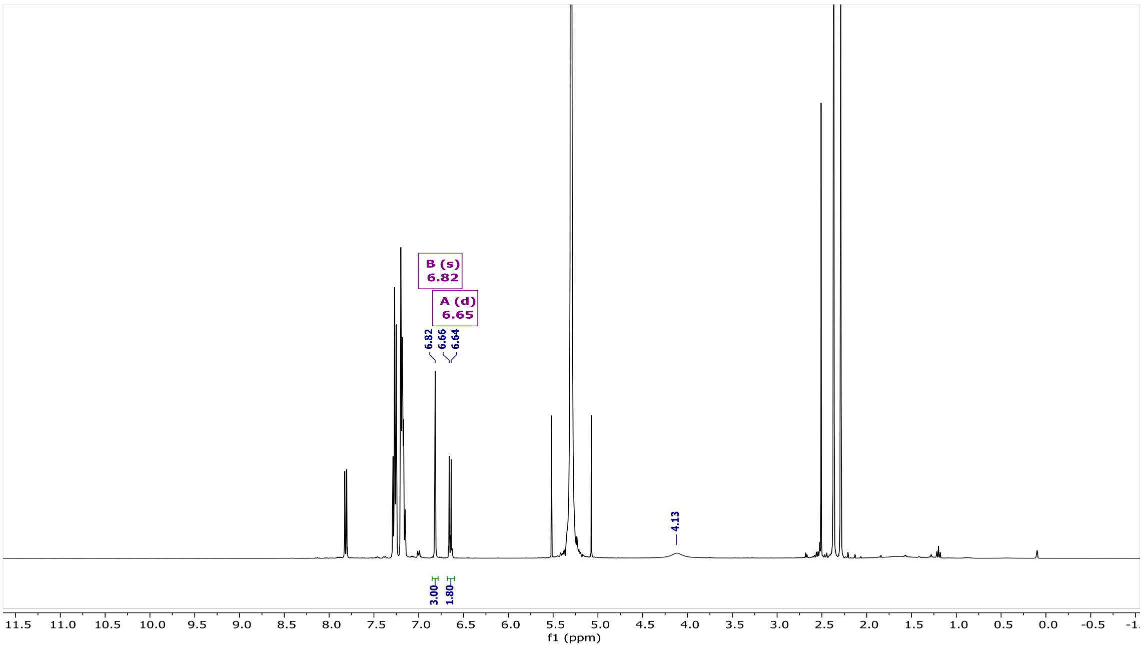

Figure S56 – ^{1}H NMR (400 MHz, $CDCl_3$, 296 K) spectrum of the reaction mixture using **1** under *condition **A***.

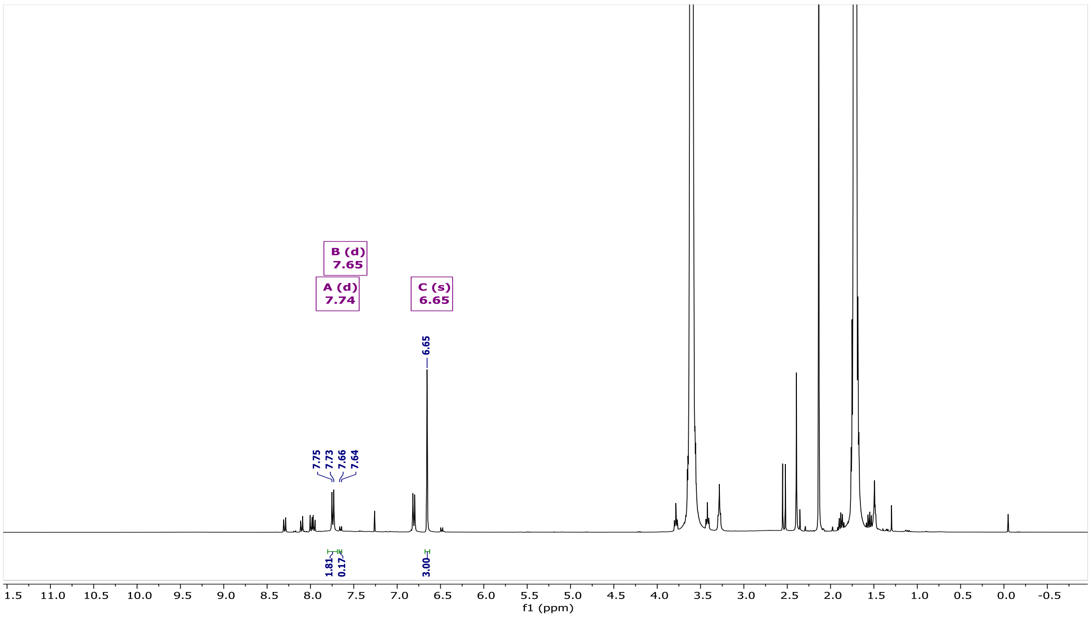

Figure S57 – ^{1}H NMR (400 MHz, $CDCl_3$, 296 K) spectrum of the reaction mixture using **1** under *condition **B***.

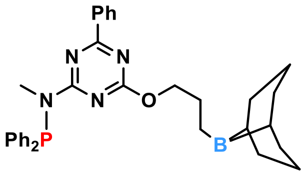

23

Ligand **23** was tested as a catalyst by using either the general method for aniline synthesis (*condition **A***) or hydroxylamine synthesis (*condition **B***).

Results:

NMR yield using *condition **A***: 0:0:0.

NMR yield using *condition **B***: 0:0:0.

Rh/C

17

Supported catalyst **17** was tested either by using the general method for aniline synthesis (*condition **A***) or hydroxylamine synthesis (*condition **B***).

Results:

NMR yield using *condition **A***: 8:0:0.

NMR yield using *condition **B***: 11:9:0.

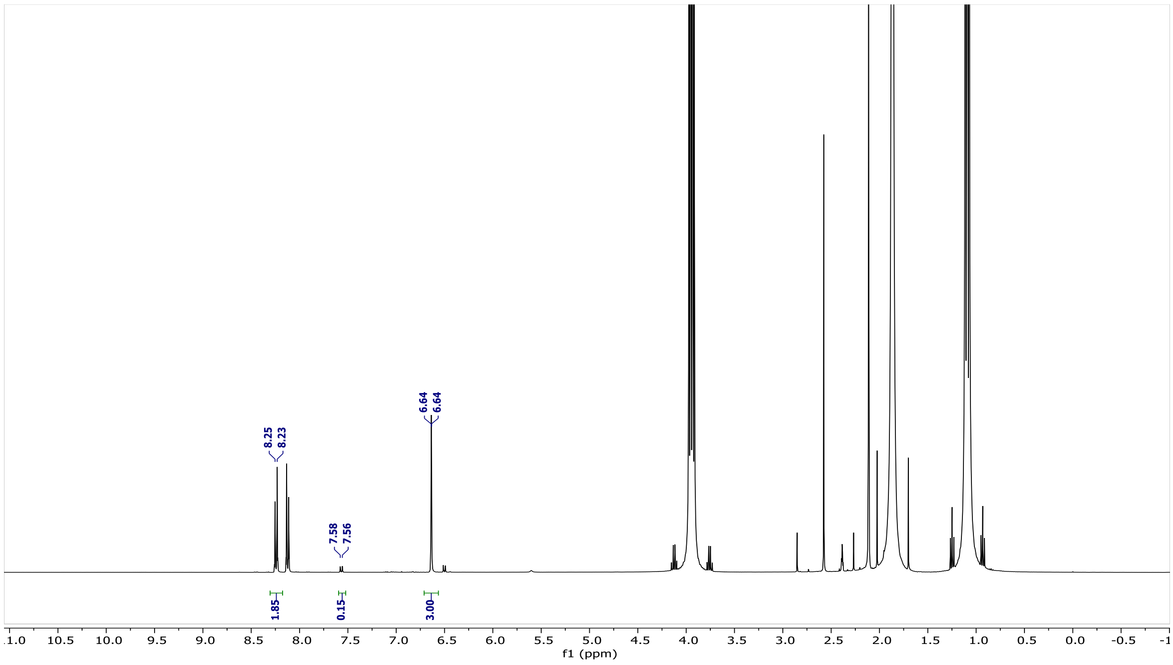

Figure S58 – ^{1}H NMR (400 MHz, DMSO-d_6, 296 K) spectrum of the reaction mixture using **17** under *condition **A***.

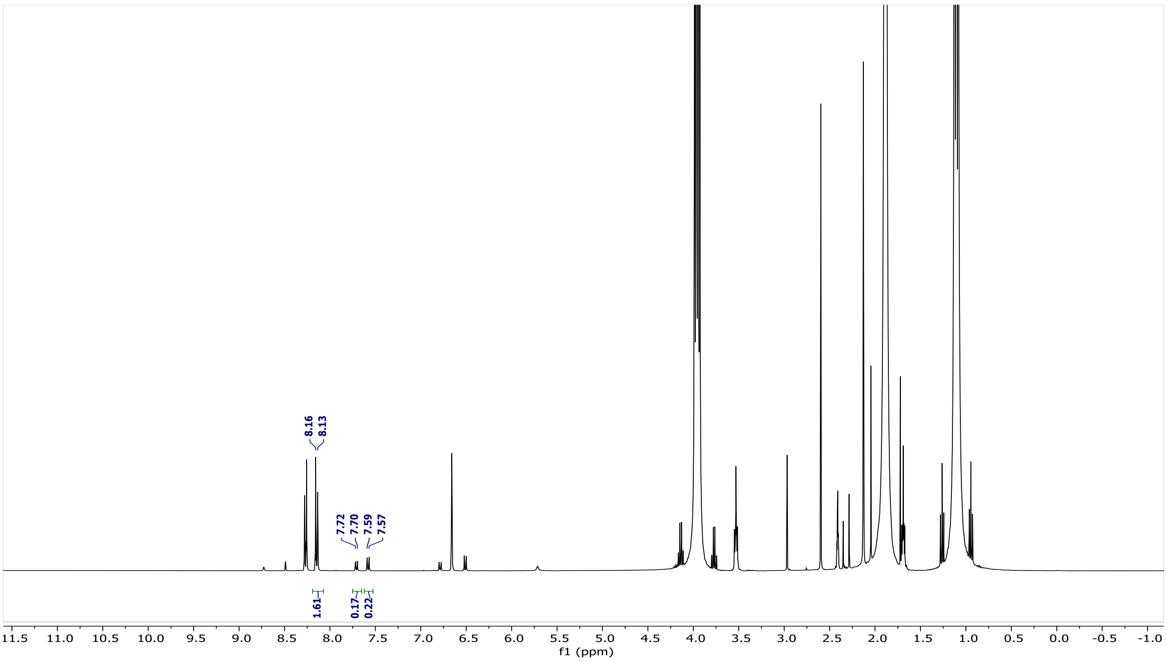

Figure S59 – ^{1}H NMR (400 MHz, DMSO-*d*$_6$, 296 K) spectrum of the reaction mixture using **17** under *condition **B***.

Pd/C

18

Supported catalyst **18** was tested either by using the general method for aniline synthesis (*condition **A***) or hydroxylamine synthesis (*condition **B***).

Results:

NMR yield using *condition **A***: 60:0:0.

NMR yield using *condition **B***: 27:0:0.

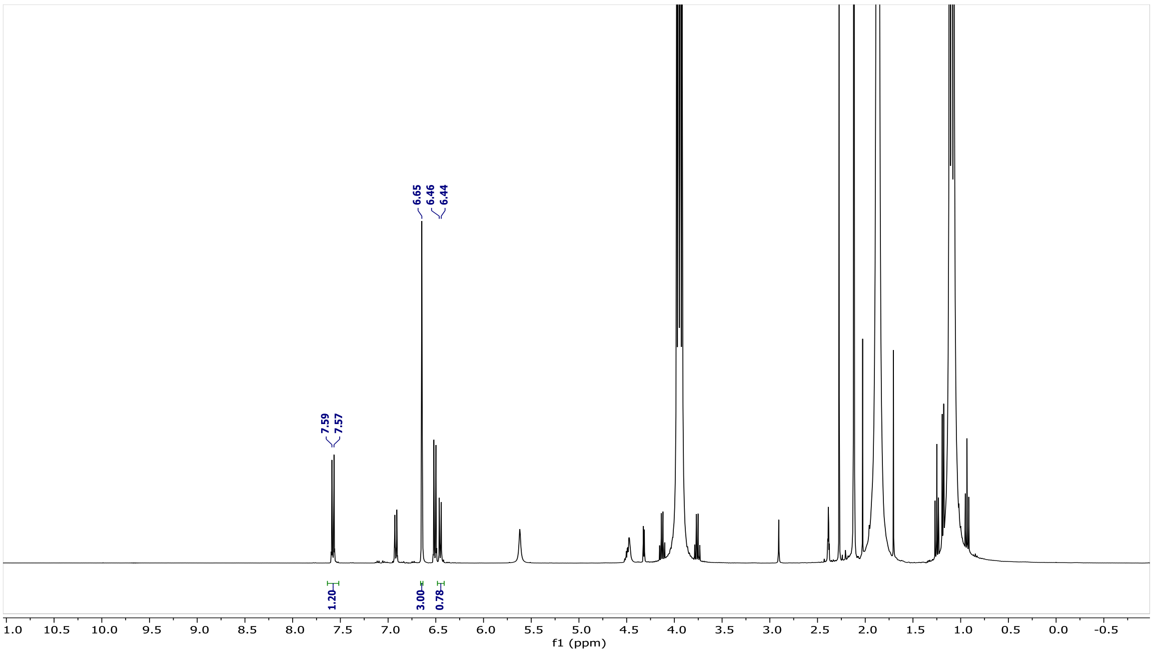

Figure S60 – ^{1}H NMR (400 MHz, DMSO-*d*$_6$, 296 K) spectrum of the reaction mixture using **18** under *condition **A***.

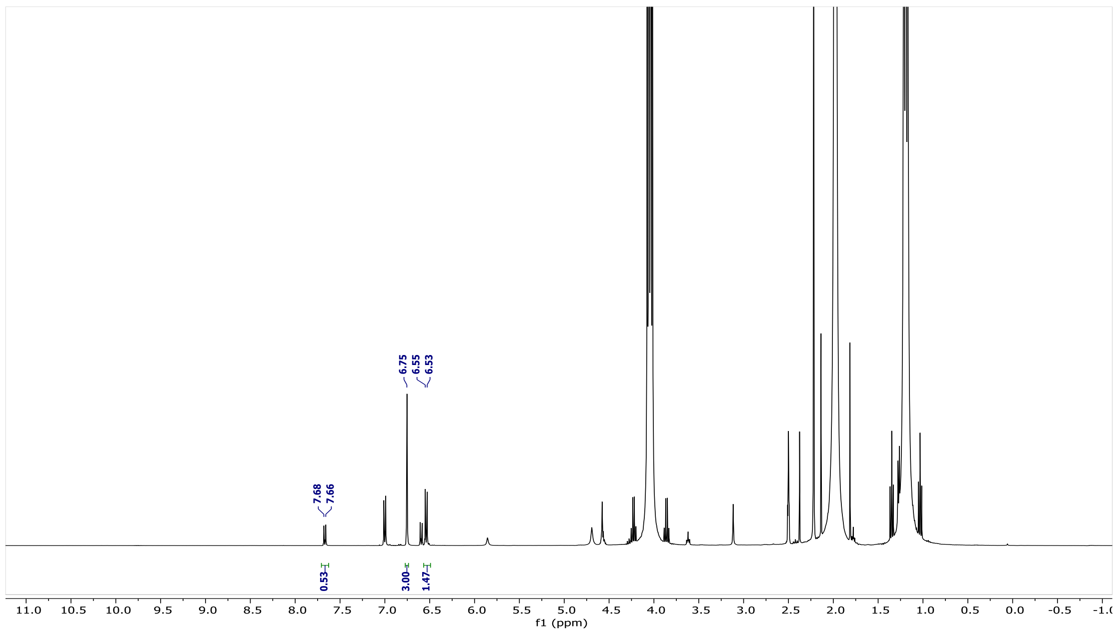

Figure S61 – ^{1}H NMR (400 MHz, DMSO-d_6, 296 K) spectrum of the reaction mixture using **18** under *condition **B***.

Pt/C

19

Supported catalyst **19** was tested either by using the general method for aniline synthesis (*condition **A***) or hydroxylamine synthesis (*condition **B***).

Results:

NMR yield using *condition **A***: 90:0:0.

NMR yield using *condition **B***: 92:0:0.

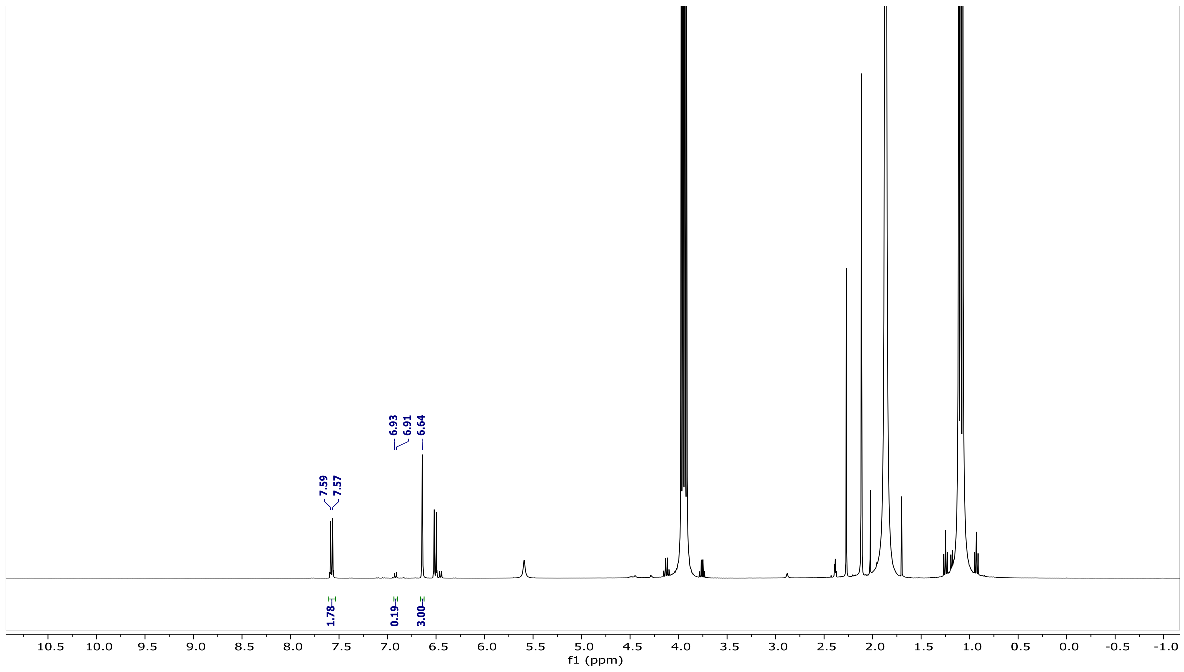

Figure S62 – ^{1}H NMR (400 MHz, DMSO-d_6, 296 K) spectrum of the reaction mixture using **19** under *condition **A***.

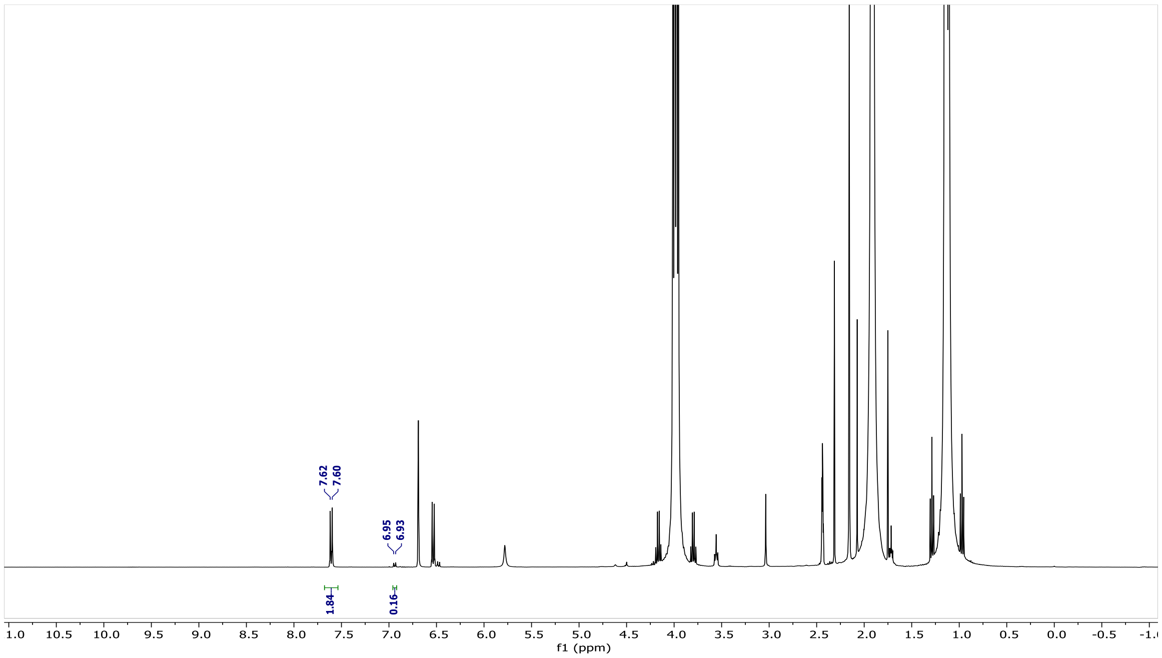

Figure S63 – ^{1}H NMR (400 MHz, DMSO-*d*₆, 296 K) spectrum of the reaction mixture using **19** under *condition **B***.

2. Poisoning Experiments

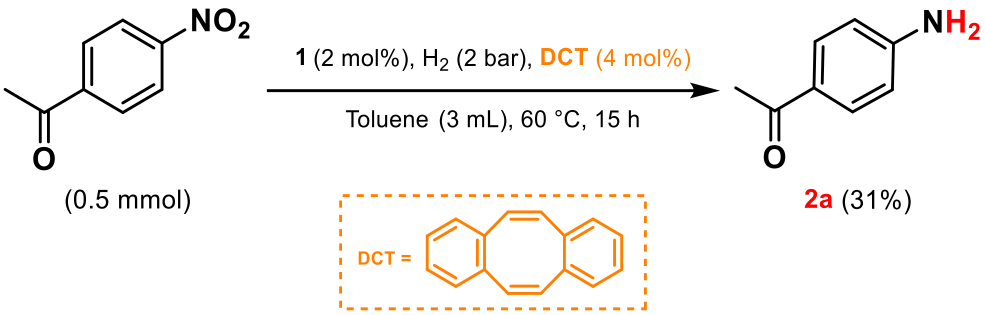

Procedure: The homogeneity of the catalyst was tested under *condition* **A** by performing the reaction in the presence of Dibenzo[a,e]cyclooctatetraene (DCT, 4 mg, 4 mol%), a poison that forms catalytically inert complexes.[24] DCT induces a reduction in yield from 90% to 31% under *condition **A***.

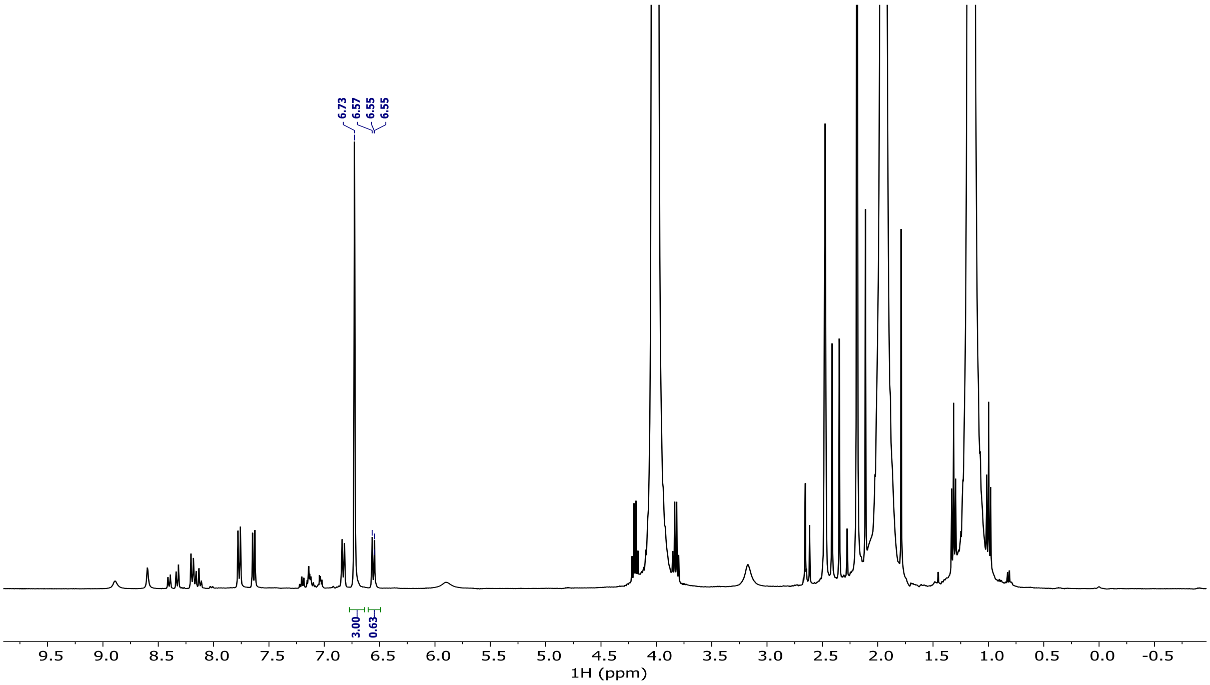

Figure S64 – ^{1}H NMR (400 MHz, DMSO-*d*$_6$, 296 K) spectrum of the reaction mixture using **1** in the presence of DCT under *condition **A***.

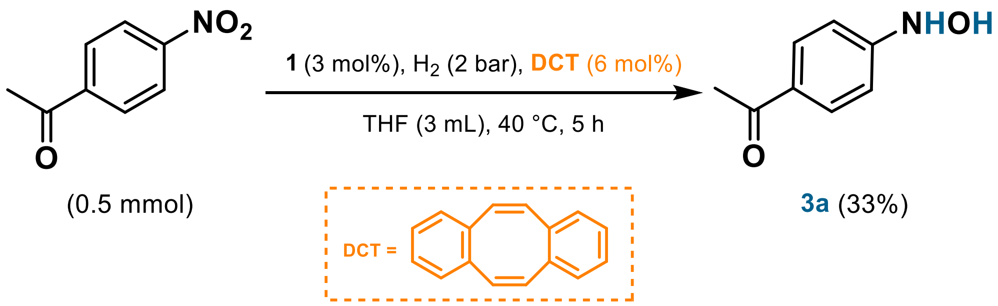

Procedure: The homogeneity of the catalyst was tested under *condition* **B** by performing the reaction in the presence of Dibenzo[a,e]cyclooctatetraene (DCT, 6 mg, 6 mol%), a poison that forms catalytically inert complexes.[24] DCT induces a reduction in yield from 75% to 33% under *condition **B***.

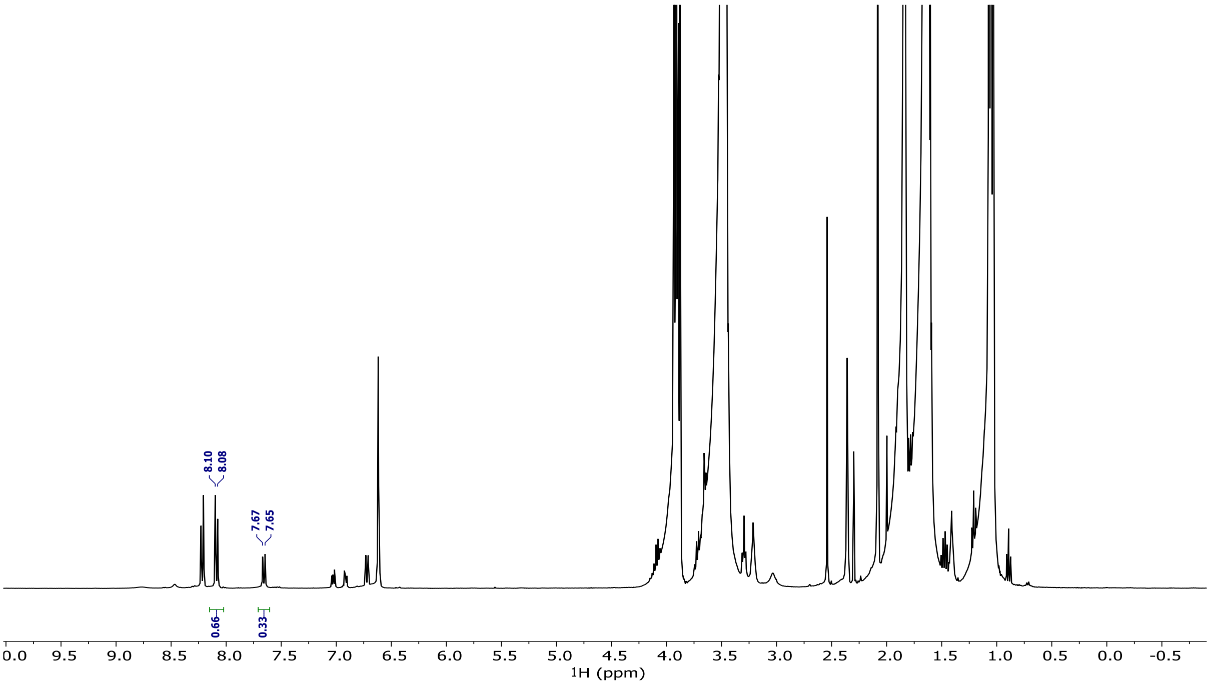

Figure S65 – ^{1}H NMR (400 MHz, DMSO-*d*$_6$, 296 K) spectrum of the reaction mixture using **1** in the presence of DCT under *condition **B***.

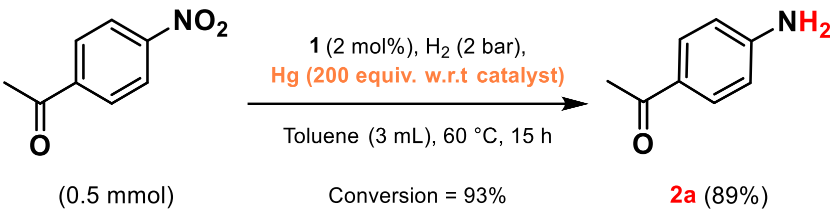

Procedure: In an oven-dried Fisher-Porter tube under argon, 1-(4-nitrophenyl)ethan-1-one (83 mg, 0.5 mmol), complex **1** (13.4 mg, 2 mol%), and toluene (3 mL) were charged. The reaction mixture was heated at 60 °C for 2 hours before adding Hg (401 mg, 2 mmol) under an inert atmosphere and then pressurized with H_2 (2 bar). The resulting reaction mixture was stirred for 13 hours at 60 °C. The reaction vessel was cooled to ambient temperature, the pressure was released, and the solvent was removed *in vacuo*. The residue was analyzed by ^{1}H NMR spectroscopy relative to mesitylene (0.5 mmol) as an internal standard to access the conversion and yield of the reaction.

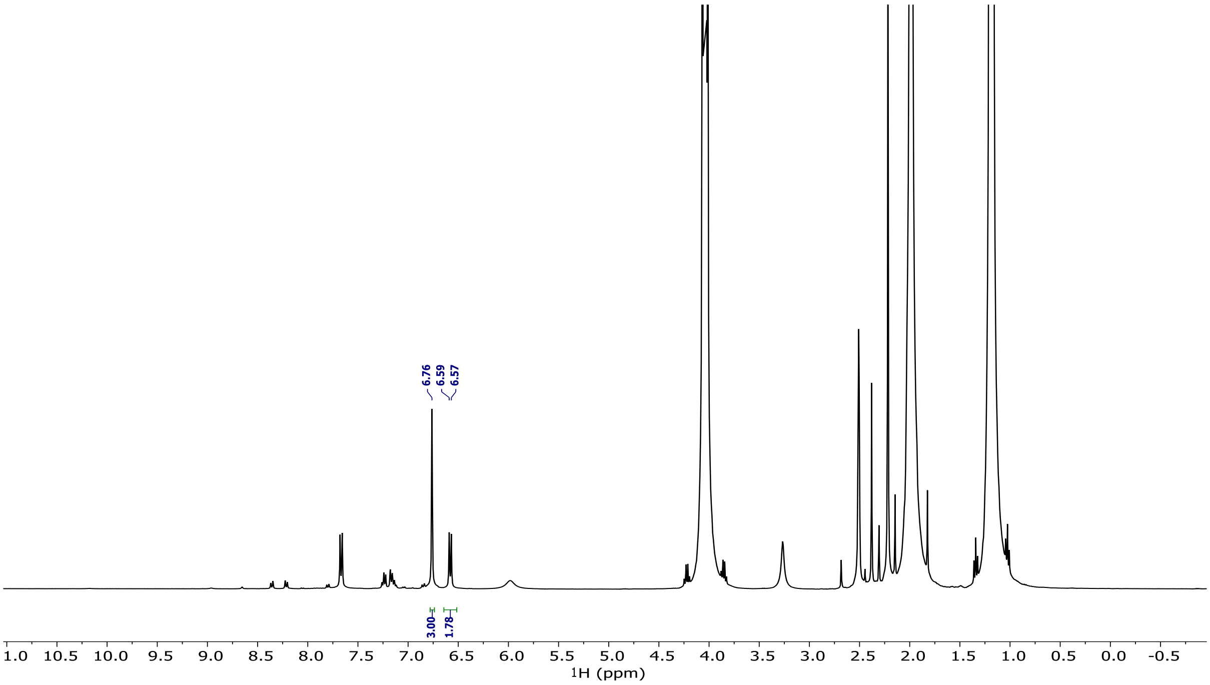

Figure S66 – ^{1}H NMR (400 MHz, DMSO-*d*$_6$, 296 K) crude spectrum of the reaction mixture using **1** in the presence of Hg under *condition* ***A***.

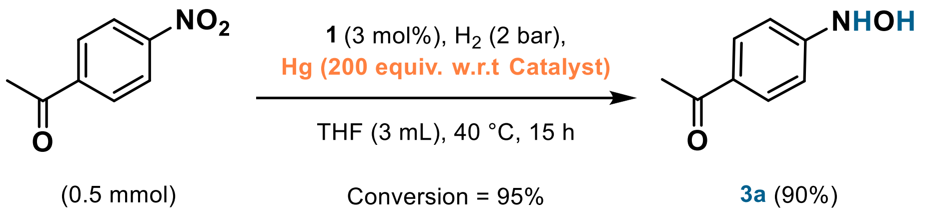

Procedure: In an oven-dried Fisher-Porter tube under argon, 1-(4-nitrophenyl)ethan-1-one (83 mg, 0.5 mmol), complex **1** (20.1 mg, 3 mol%), Hg (602 mg, 3 mmol) and THF (3 mL) were charged. The reaction mixture was frozen using liquid nitrogen, evacuated, and then pressurized with H_2 (2 bar). The resulting reaction mixture was stirred for 15 hours at 40 °C. The reaction vessel was cooled to ambient temperature, the pressure was released, and the solvent was removed *in vacuo*. The residue was analyzed by ^{1}H NMR spectroscopy relative to mesitylene (0.5 mmol) as an internal standard to access the conversion and yield of the reaction.

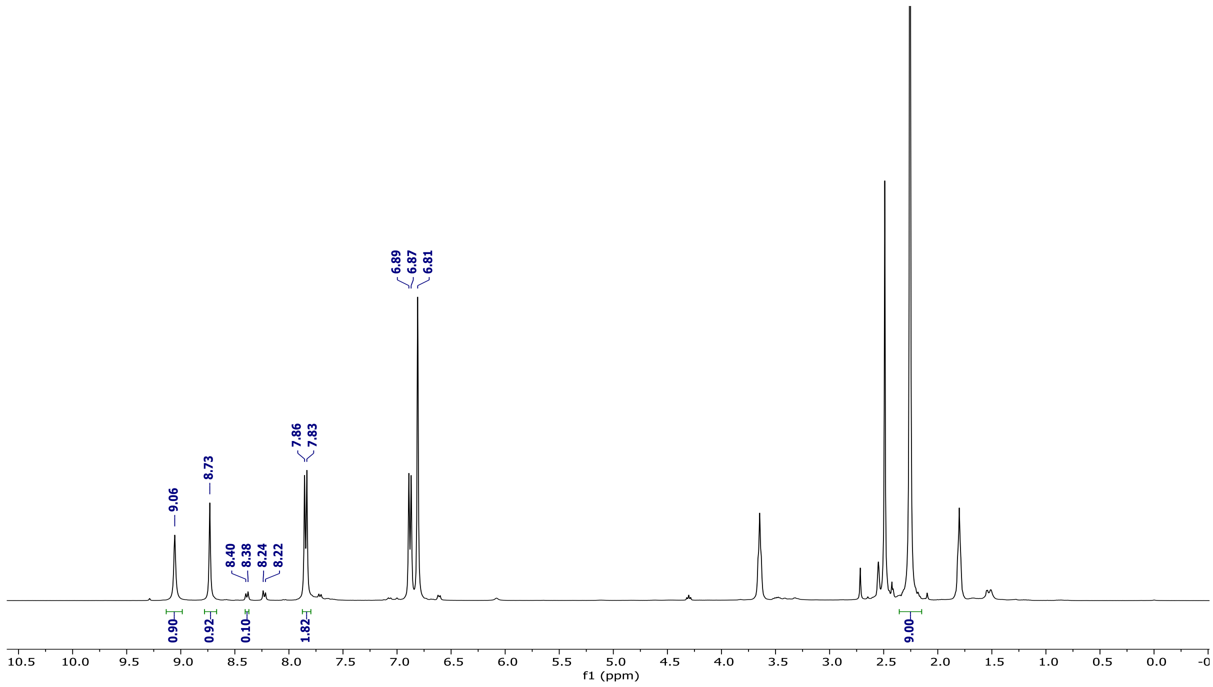

Figure S67 – ^{1}H NMR (400 MHz, DMSO-d_6, 296 K) crude spectrum of the reaction mixture using **1** in the presence of Hg under *condition* ***A***.

3. Reactions Using Water Instead of H_2 as the Reducing Agent

- **Using Water (1 equiv.) Instead of H_2**

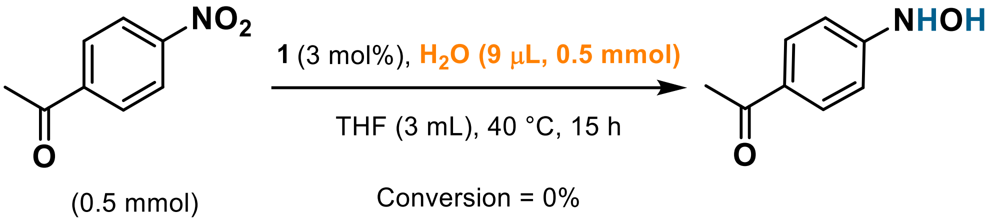

Procedure: To test whether hydrogen is required for the reaction to proceed, we followed the general procedure for hydroxylamine synthesis (*condition **B***) using water (9 µL, 0.5 mmol) instead of H_2. Under these conditions, the substrate was recovered entirely, indicating that hydrogen acts as the reducing agent.

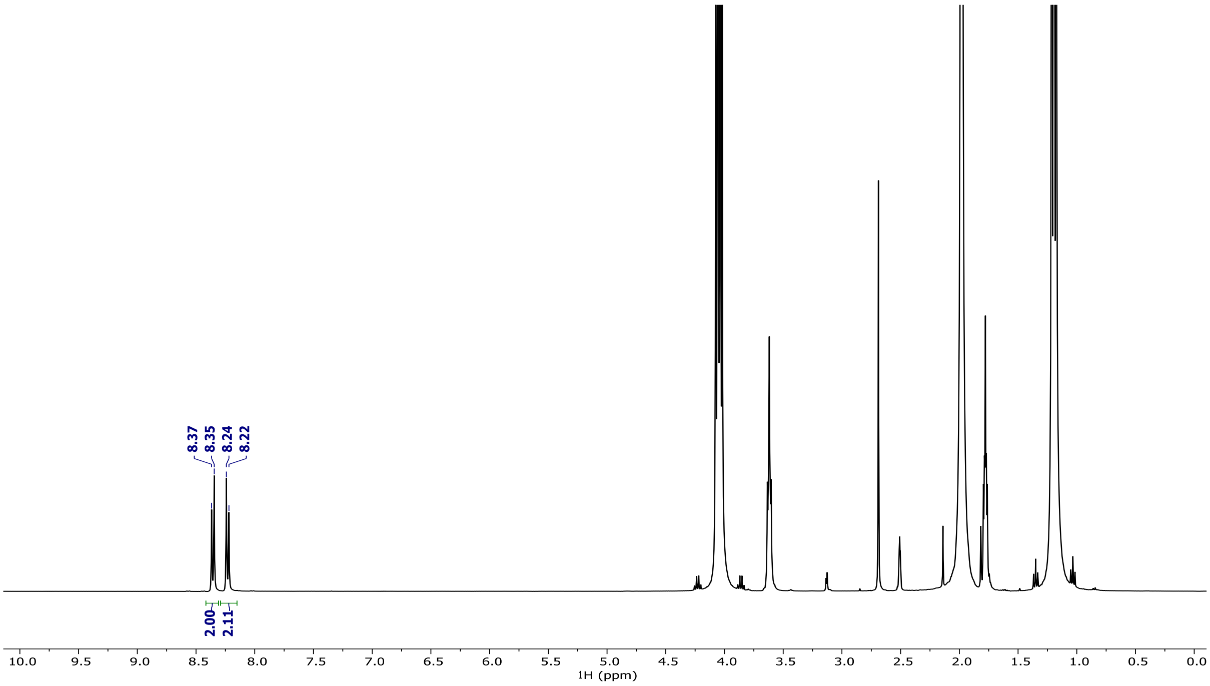

Figure S68 – ^{1}H NMR (400 MHz, DMSO-*d*$_6$, 296 K) crude spectrum of the reaction mixture using **1** in the presence of H_2O (1 equiv.) instead of H_2 under *condition **B***.

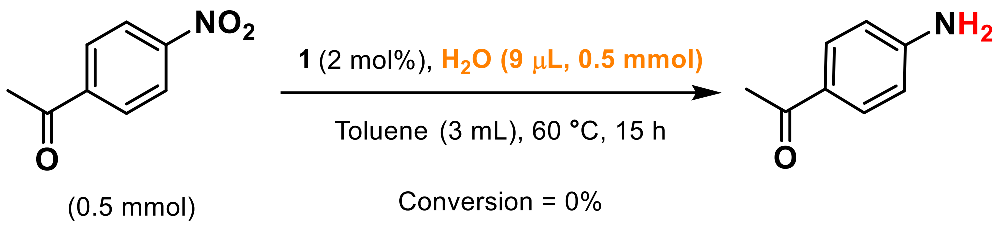

Procedure: To test whether hydrogen is required for the reaction to proceed, we followed the general procedure for aniline synthesis (*condition **A***) using water (9 µL, 0.5 mmol) instead of H_2. Under these conditions, the substrate was recovered entirely, indicating that hydrogen acts as the reducing agent.

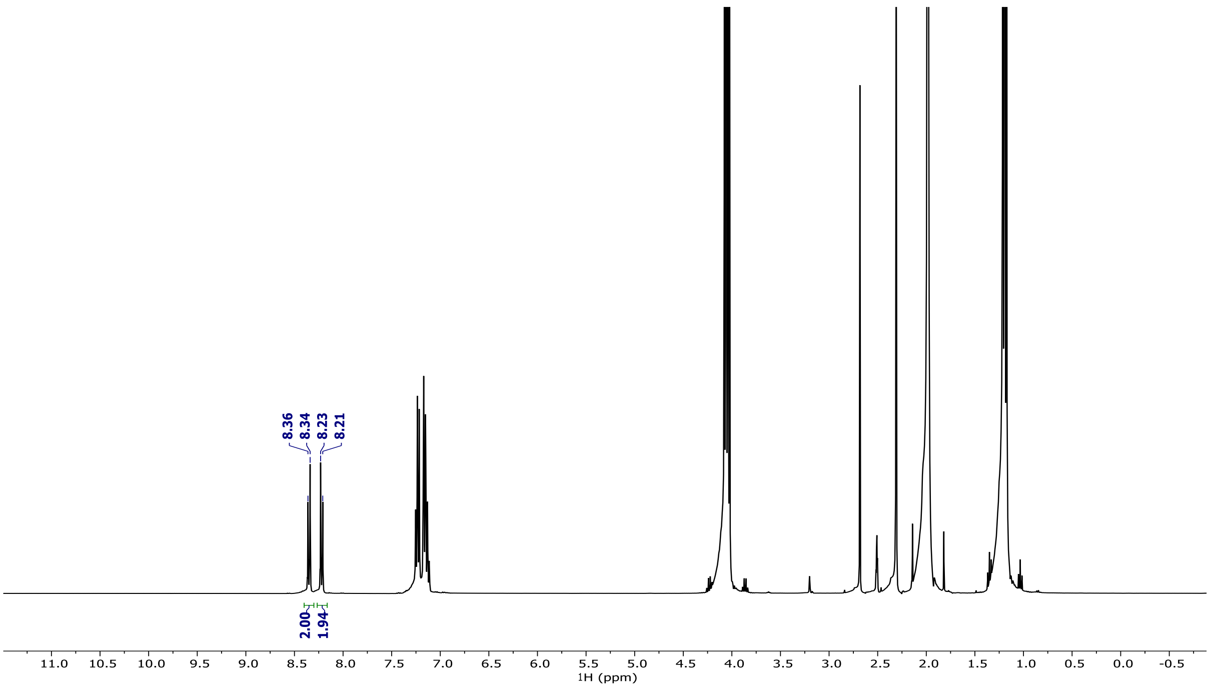

Figure S69 – ^{1}H NMR (400 MHz, DMSO-*d_6*, 296 K) spectrum of the reaction mixture using **1** in the presence of H_2O (1 equiv.) instead of H_2 under *condition **A***.

- **Using Water (2 equiv.) Instead of H_2**

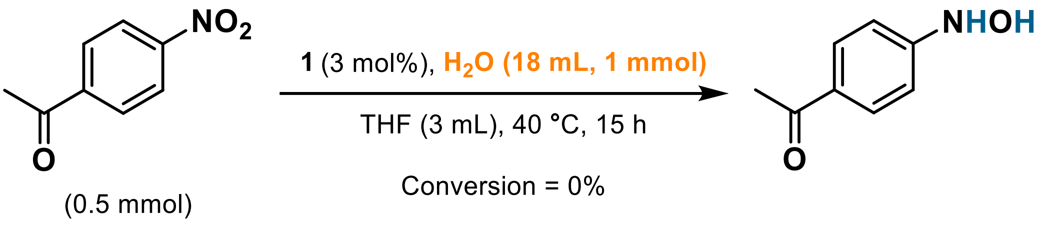

Procedure: To test whether hydrogen is required for the reaction to proceed, we followed the general procedure for hydroxylamine synthesis (*condition **B***) using water (18 µL, 1 mmol) instead of H_2. Under these conditions, the substrate was recovered entirely, indicating that hydrogen acts as the reducing agent.

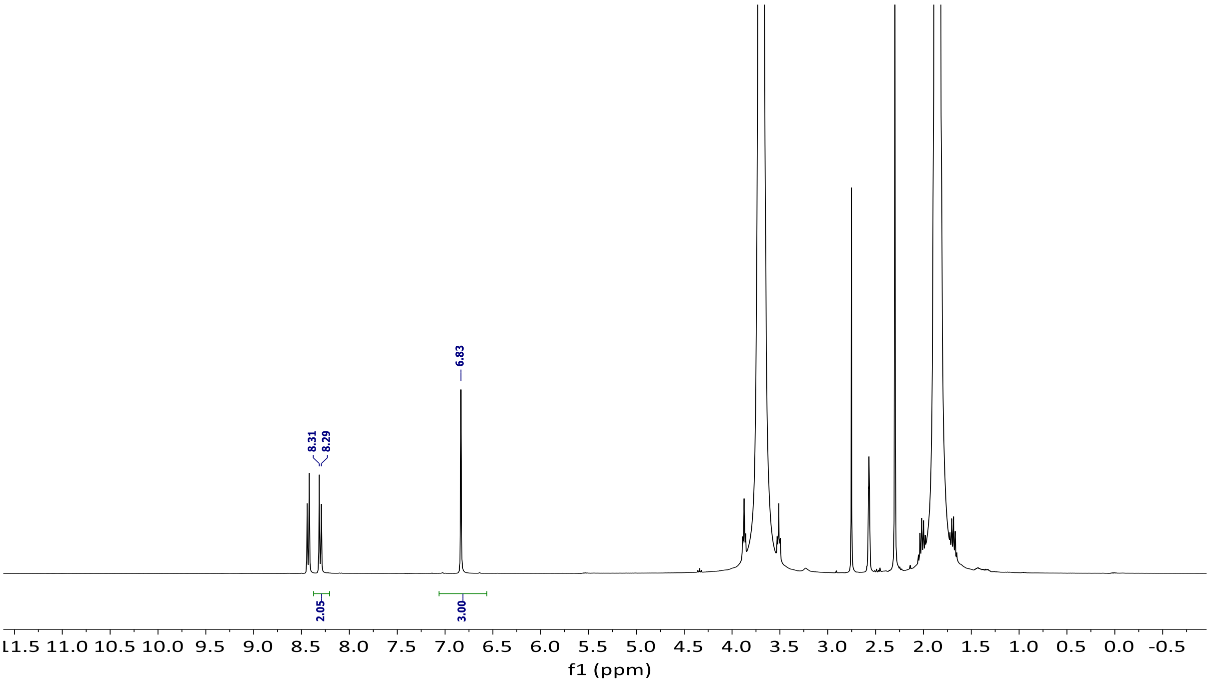

Figure S70 – ^{1}H NMR (400 MHz, DMSO-d_6, 296 K) spectrum of the reaction mixture using **1** in the presence of H_2O (2 equiv.) instead of H_2 under *condition **B***.

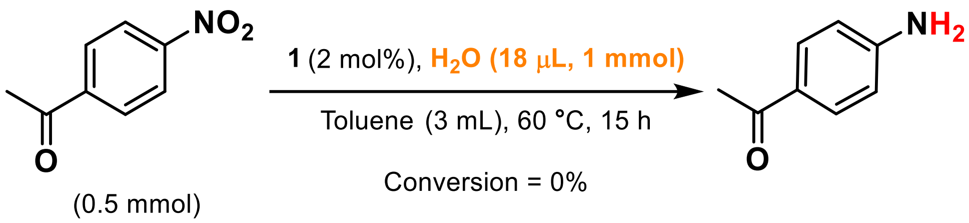

Procedure: To test whether hydrogen is required for the reaction to proceed, we followed the general procedure for aniline synthesis (*condition A*) using water (18 µL, 1 mmol) instead of H_2. Under these conditions, the substrate was recovered entirely, indicating that hydrogen acts as the reducing agent.

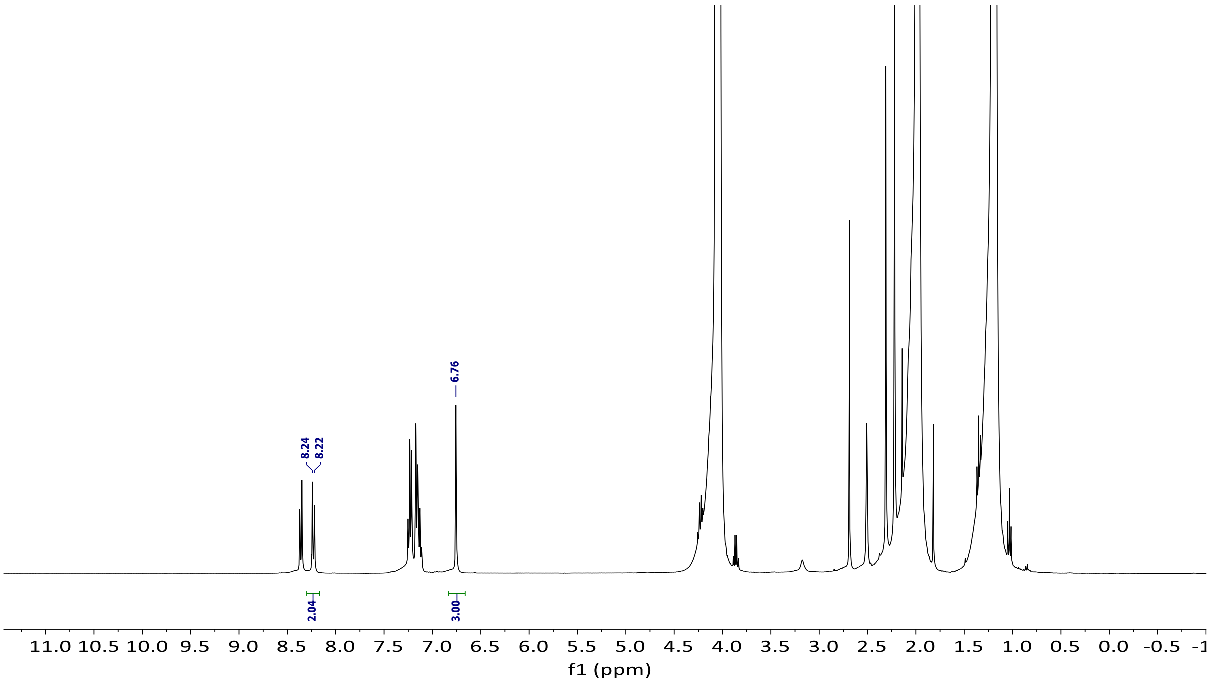

Figure S71 – ^{1}H NMR (400 MHz, DMSO-*d*$_6$, 296 K) spectrum of the reaction mixture using **1** in the presence of H_2O (2 equiv.) instead of H_2 under *condition **A**.*

- **Using Water (Excess) Instead of H_2**

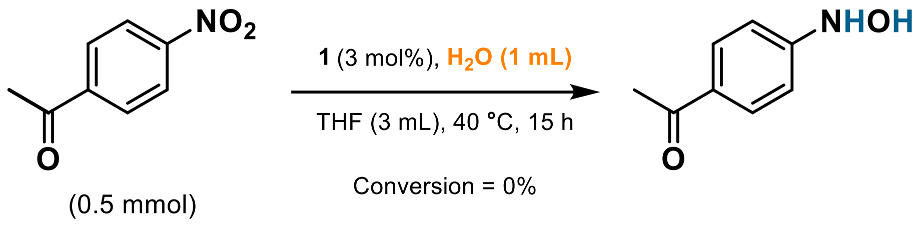

Procedure: To test whether hydrogen is required for the reaction to proceed, we followed the general procedure for hydroxylamine synthesis (*condition **B***) using water (1 mL) instead of H_2. Under these conditions, the substrate was recovered entirely, indicating that hydrogen acts as the reducing agent.

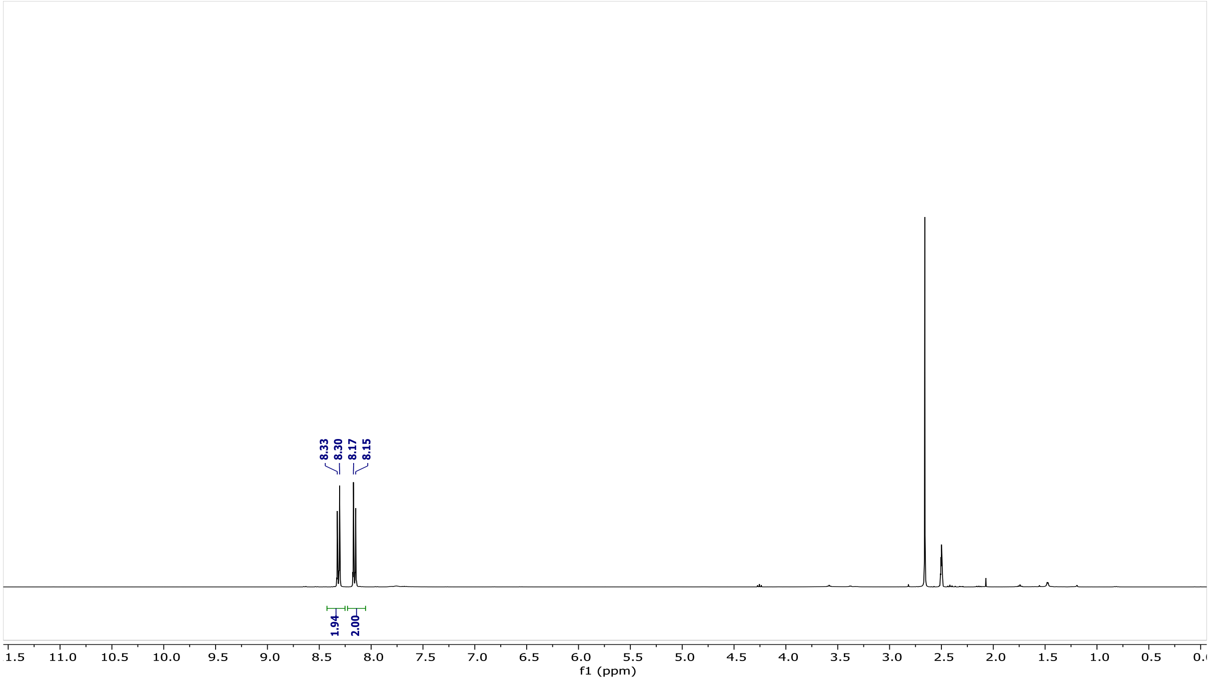

Figure S72 – ^{1}H NMR (400 MHz, DMSO-d_6, 296 K) crude spectrum of the reaction mixture using **1** in the presence of H_2O (excess) instead of H_2 under *condition **B***.

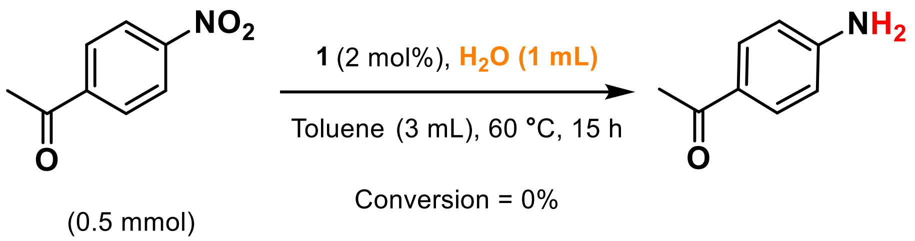

Procedure: To test whether hydrogen is required for the reaction to proceed, we followed the general procedure for aniline synthesis (*condition A*) using water (1 mL) instead of H_2. Under these conditions, the substrate was recovered entirely, indicating that hydrogen acts as the reducing agent.

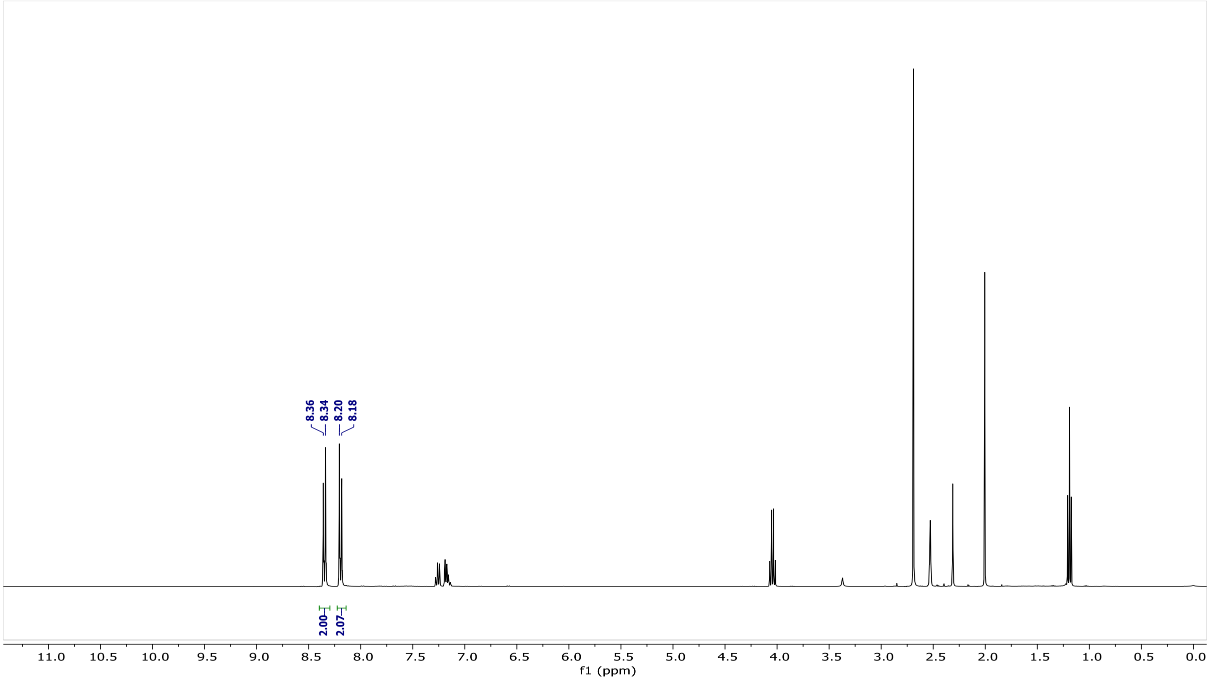

Figure S73 – ^{1}H NMR (400 MHz, DMSO-*d*$_6$, 296 K) crude spectrum of the reaction mixture using **1** in the presence of H_2O (excess) instead of H_2 under *condition A*.

4. Reaction with Water as an Additive in the Presence of H_2

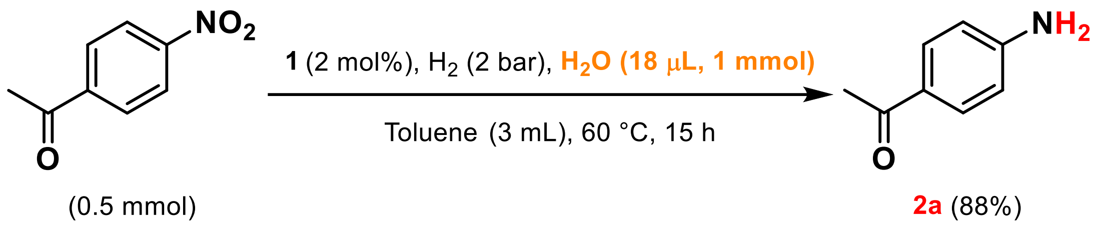

Procedure: The reaction was performed by following the general procedure for aniline synthesis (*condition A*). This reaction was performed to determine whether water can bind to boron and cause the reaction to stop at hydroxylamine level. The formation of **2a** (88%) indicates that higher concentrations of binding solvent are required to stop the reaction at the level of hydroxylamine.

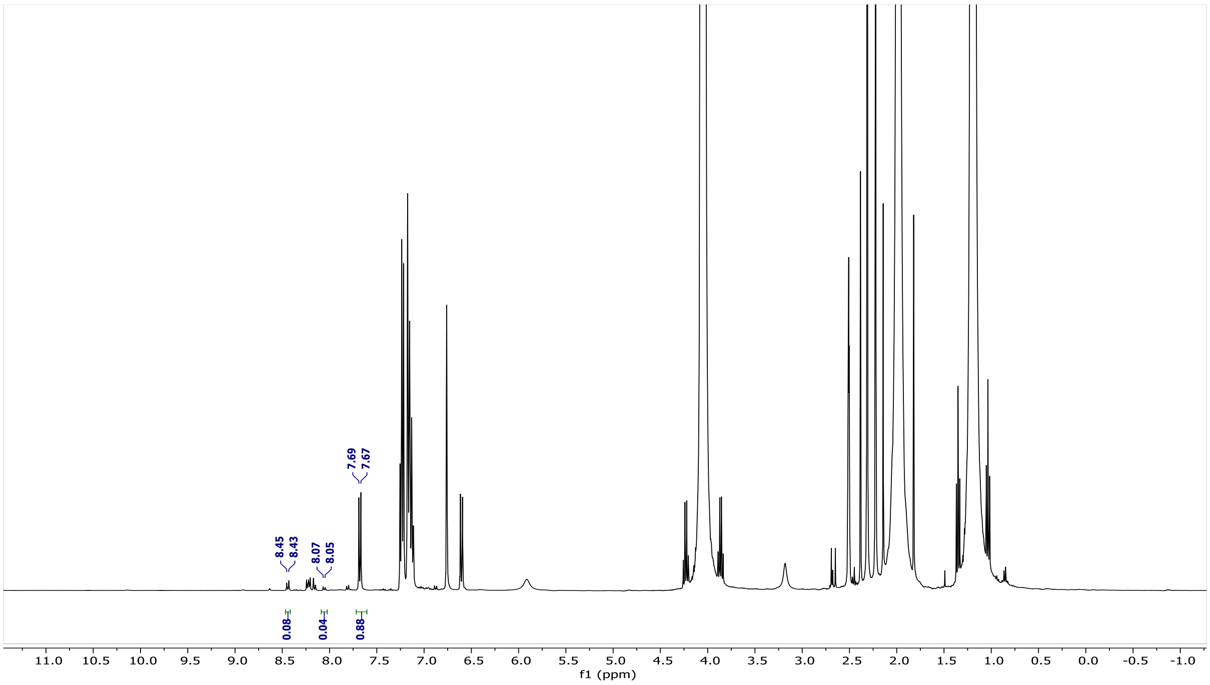

Figure S74 – ^{1}H NMR (400 MHz, $CDCl_3$, 296 K) crude spectrum of the reaction mixture using *condition A*.

5. Testing the Possibility of a Disproportionation Reaction

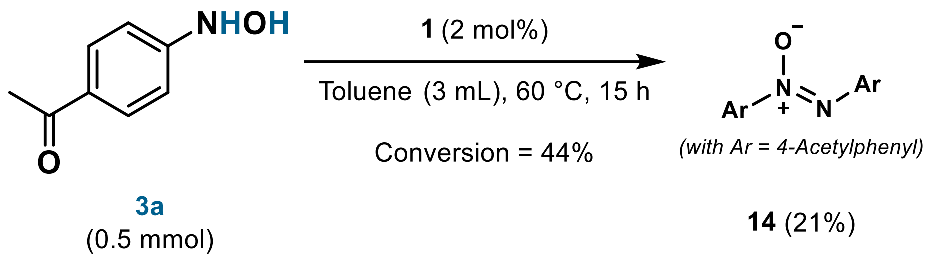

Procedure: Complex **1** in the presence of hydroxylamine under *condition **A*** without H_2 does not show any evidence of hydroxylamine disproportionation to aniline and nitroso. However, we observed the generation of the azoxy derivative **14**. Possibly, it is formed as a result of a dehydrogenation reaction on hydroxylamine that creates the nitroso derivative, which is then condensed with another hydroxylamine molecule.

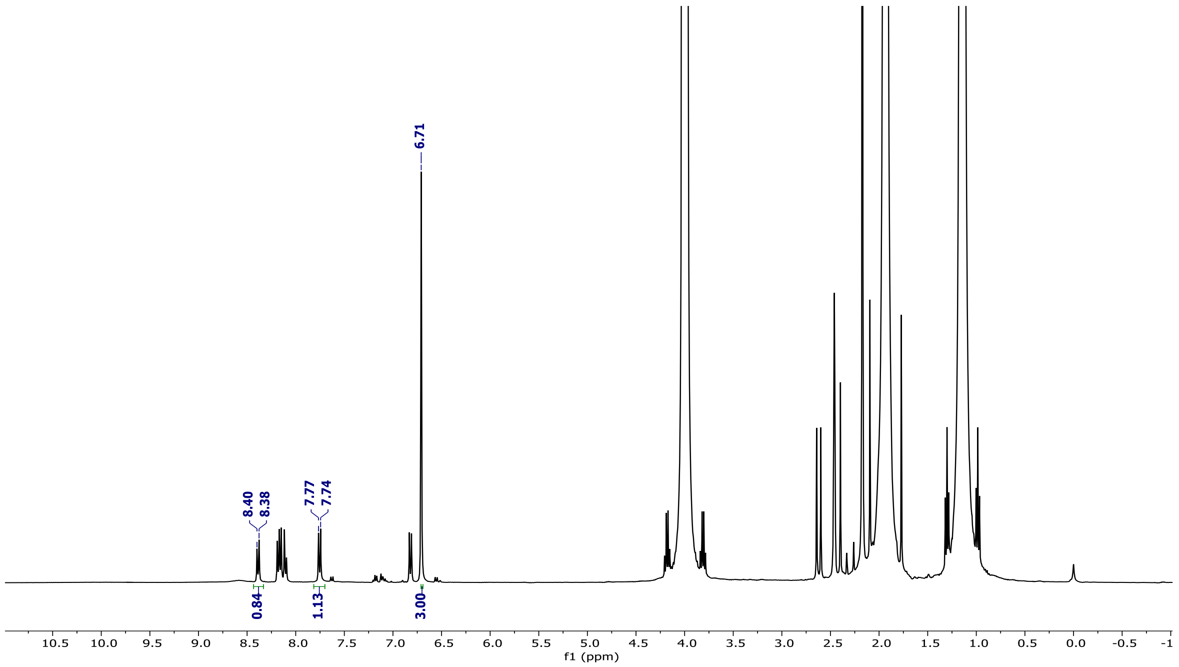

Figure S75 – ^{1}H NMR (400 MHz, DMSO-d_6, 296 K) crude spectrum of the reaction mixture using *condition **A*** without H_2.

6. Temporal Conversion Profiles

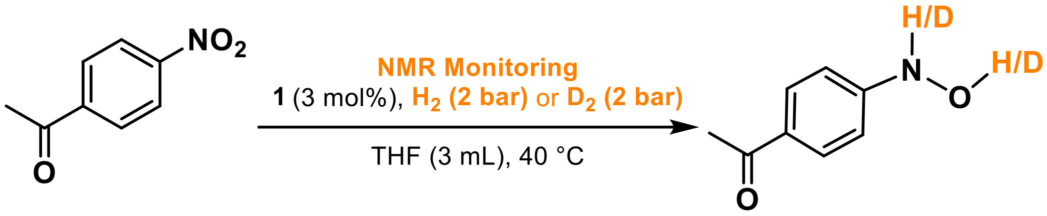

Procedure: The temporal conversion profiles for the conversion of 1-(4-nitrophenyl)ethan-1-one to hydroxylamine **3a** were monitored by ^{1}H NMR spectroscopy. The standard conditions for hydroxylamine synthesis were followed, using either H_2 or D_2 as the reducing agent.

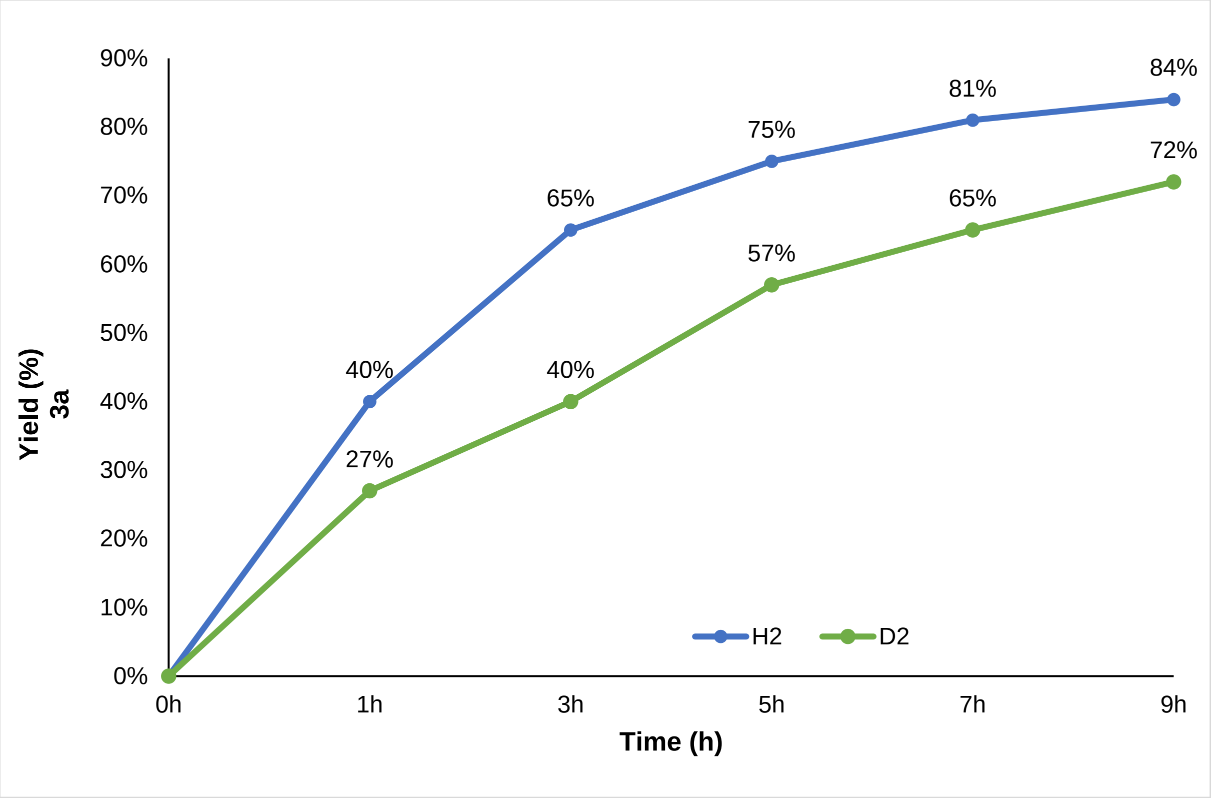

7. Identifying the Reaction Network

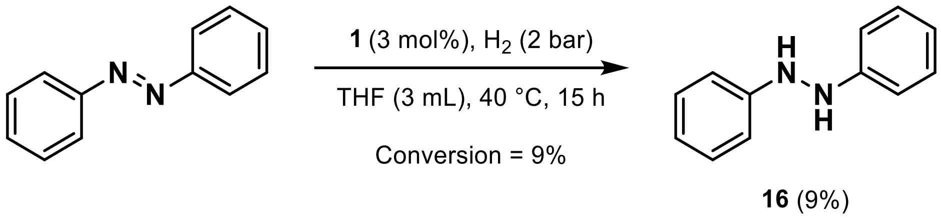

Procedure: The reaction was performed by following the general procedure for hydroxylamine synthesis (*condition* ***B***).

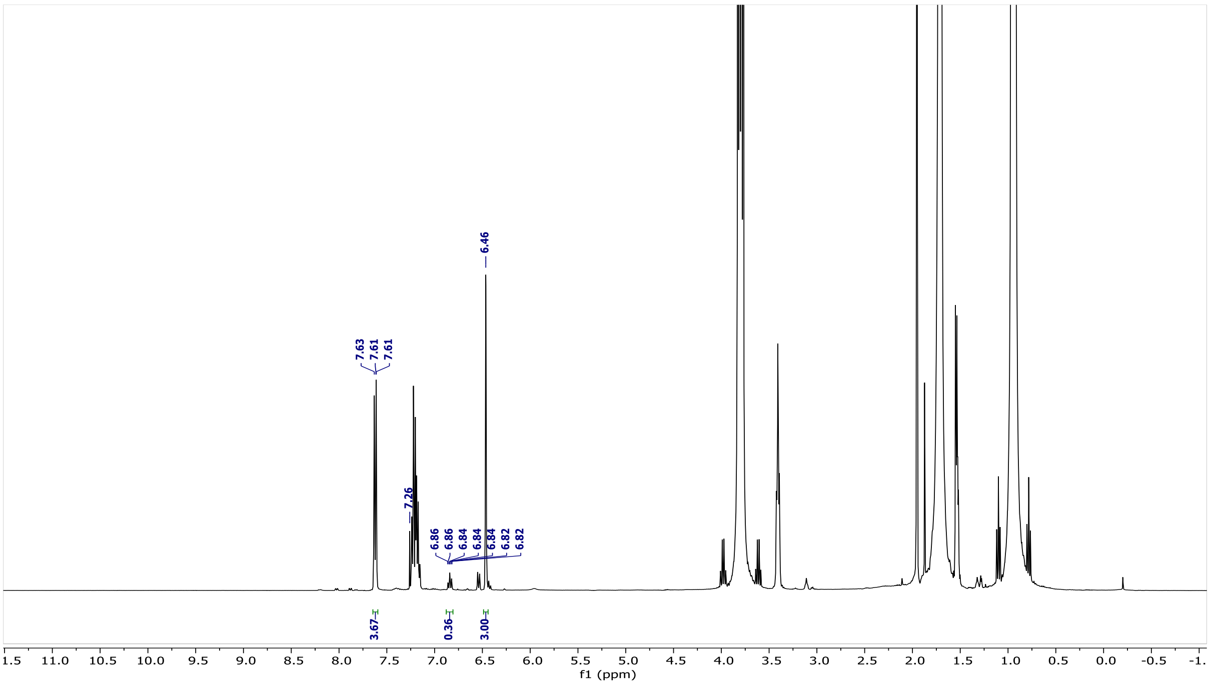

Figure S76 – ^{1}H NMR (400 MHz, $CDCl_3$, 296 K) crude spectrum of the reaction mixture using *condition* ***B***.

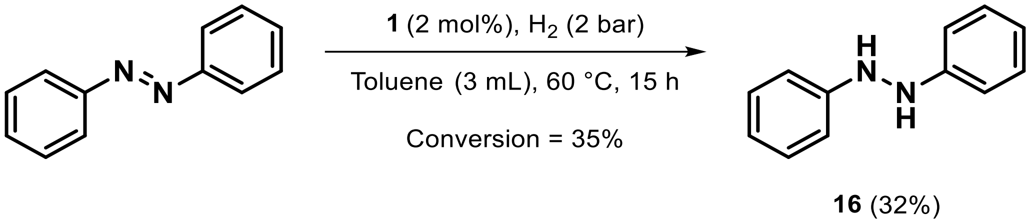

Procedure: The reaction was performed by following the general procedure for aniline synthesis (*condition **A***).

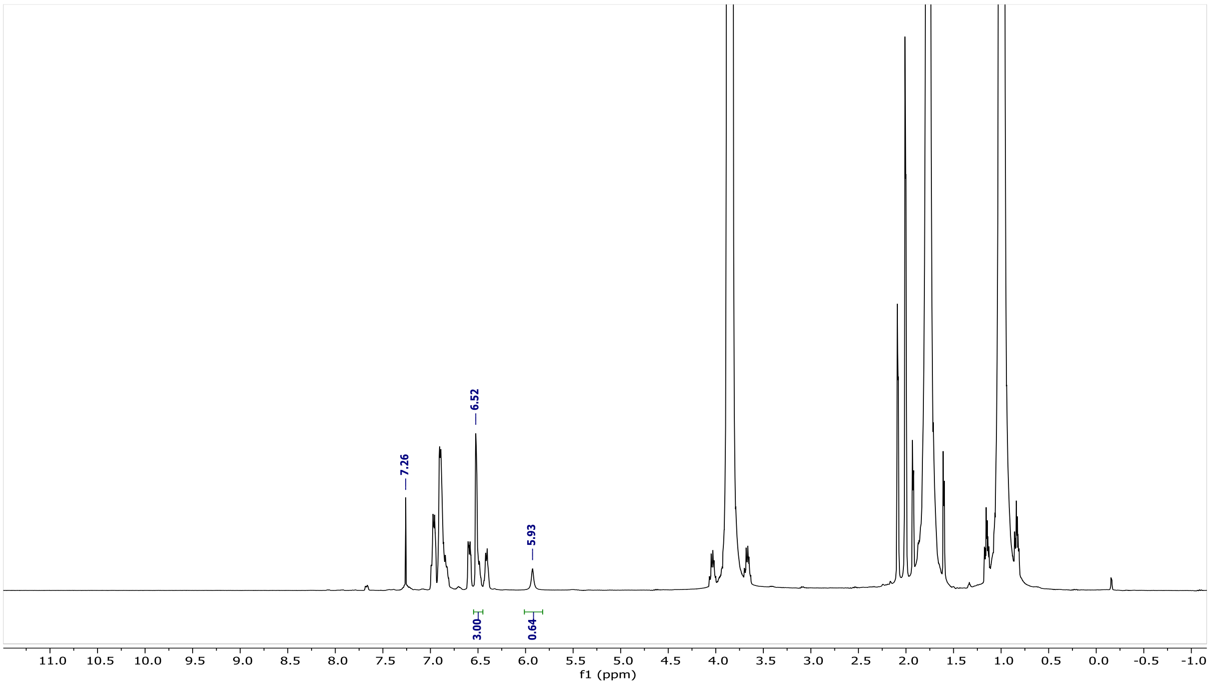

Figure S77 – ^{1}H NMR (400 MHz, CDCl$_3$, 296 K) crude spectrum of the reaction mixture using *condition **A***.

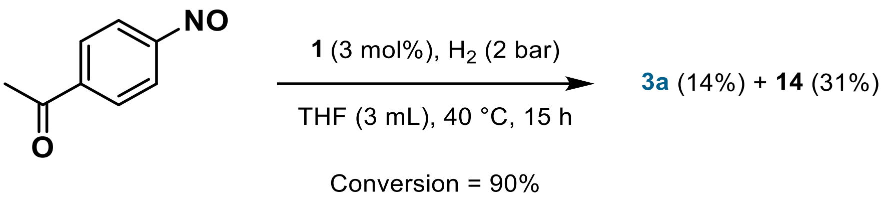

Procedure: The reaction was performed by following the general procedure for hydroxylamine synthesis (*condition **B***). The substrate 1-(4-nitrosophenyl)ethan-1-one was synthesized by following a reported literature procedure.[25]

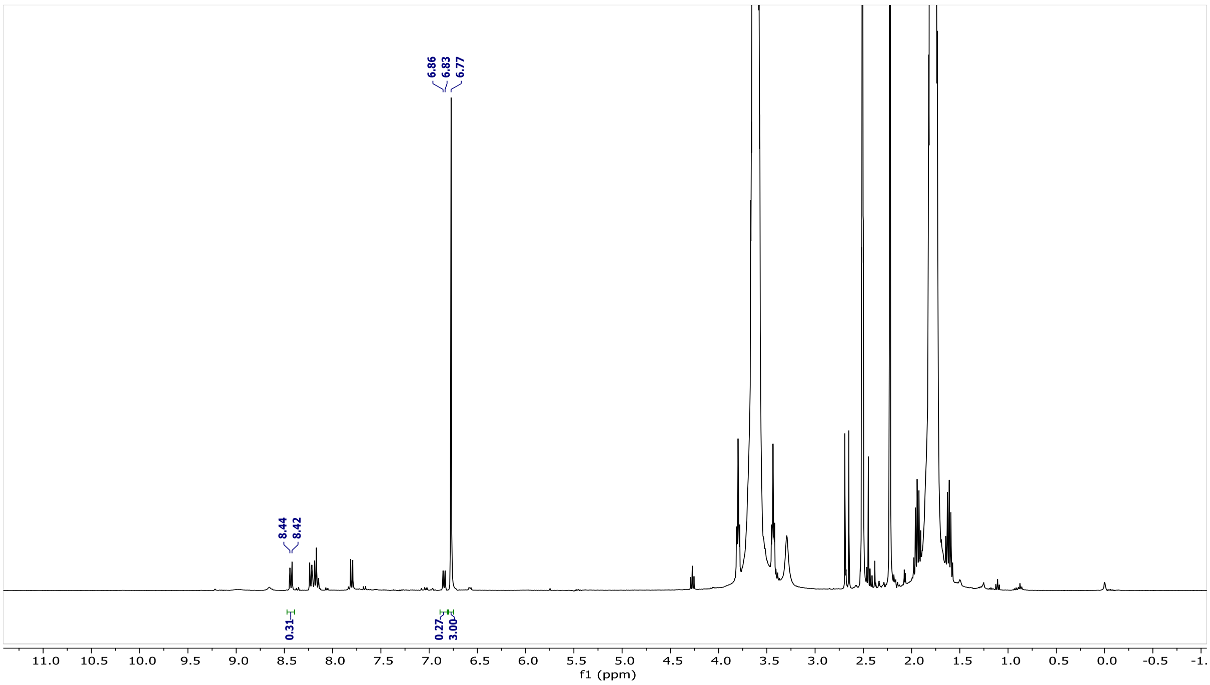

Figure S78 – ^{1}H NMR (400 MHz, DMSO-*d₆*, 296 K) crude spectrum of the reaction mixture using *condition **B***.

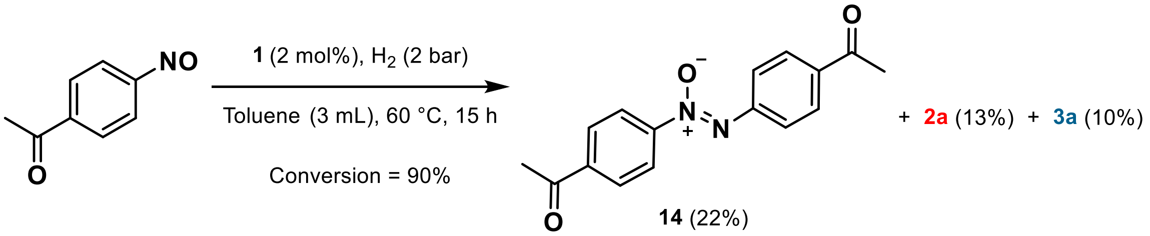

Procedure: The reaction was performed by following the general procedure for aniline synthesis (*condition **A***). The substrate 1-(4-nitrosophenyl)ethan-1-one was synthesized by following a reported literature procedure.[25]

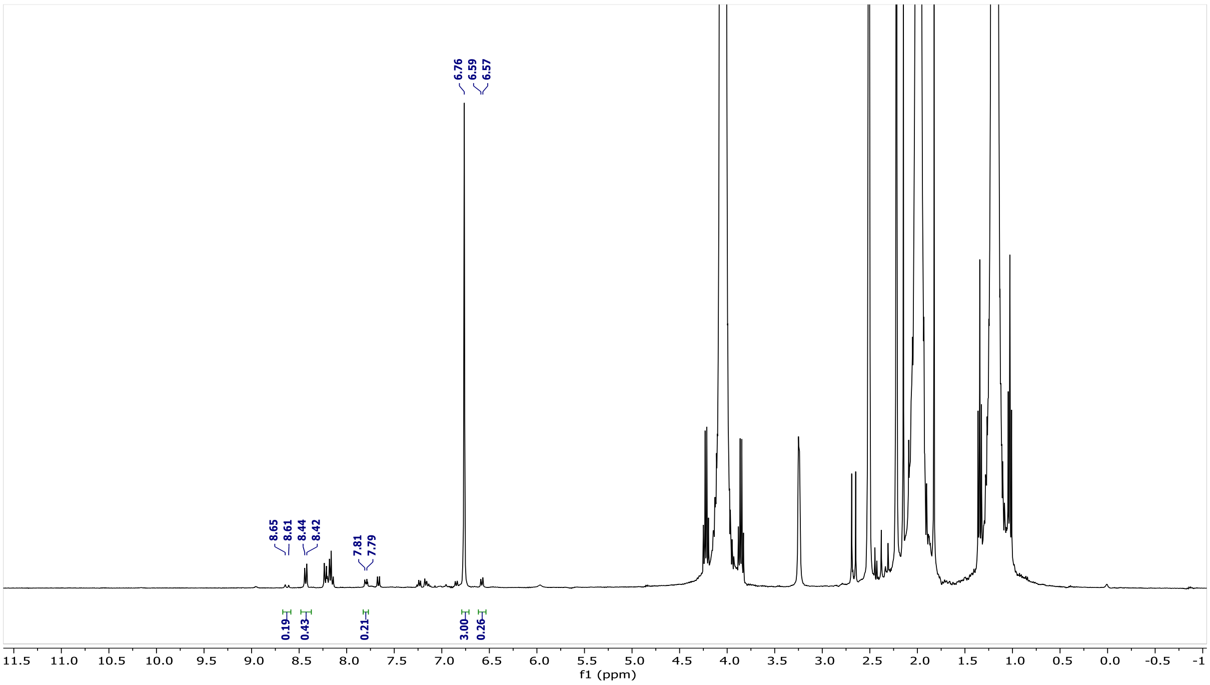

Figure S79 – ^{1}H NMR (400 MHz, DMSO-d_6, 296 K) crude spectrum of the reaction mixture using *condition **A***.

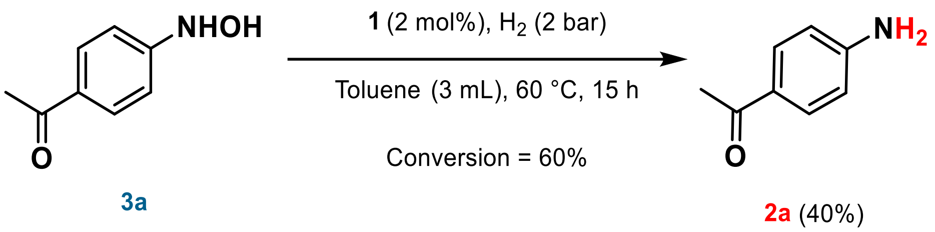

Procedure: The reaction was performed by following the general procedure for aniline synthesis (*condition A*). Substrate **3a** was synthesized by following a reported literature procedure.[19]

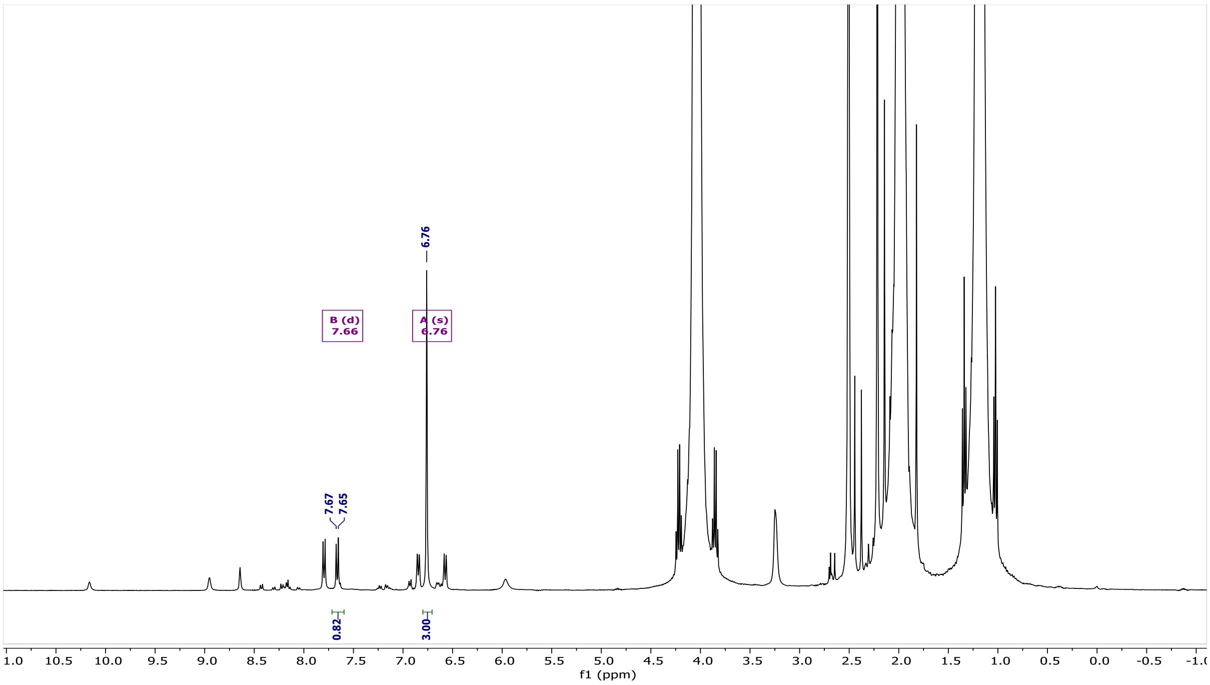

Figure S80 – ^{1}H NMR (400 MHz, DMSO-d_6, 296 K) crude spectrum of the reaction mixture using *condition A*.

8. Dihydrogen Activation (Metal-Hydride Formation)

Experimental evidence regarding the adduct formation at the boron site in the intermediate hydride complex **I** could mainly be obtained indirectly from ^{11}B NMR. Figure S81 compares ^{11}B signals from the 9-BBN of several complexes dissolved in different solvent systems. ^{11}B signals in precursor **PN**$^{tzn\text{-}B}$ **23** in CD_2Cl_2 and of the catalyst complex **1** in C_6D_6 both consist of a broad line around 85-90 ppm, which is consistent with uncoordinated trialkylboranes (83-93 ppm).[26] The hydride complex **I** could only be studied in solvent systems which included methanol or ethanol (THF:MeOH, C_6D_6:MeOH. Tol-d_8:MeOH, C_6D_6:EtOH). In these solvent mixtures, the ^{11}B signal shifts to a higher field (18 ppm), indicating coordination at the boronic site. The candidate ligands that are present in the solution are acetonitrile, alcohol, or intramolecular basic sites. Acetonitrile is present in the solution since it is released from the metal in complex **1** upon formation of the hydride; however, no expected correlations (NOE, coupling) or shifts could be observed, which would suggest its coordination to the 9-BBN site in the hydride complex **I**. Furthermore, ^{11}B signals reported in the literature for CH_3CN associated with trialkylboranes are generally close to 50 ppm.[27] This type of adduct may also be visible in spectra A and B of Figure S81.

Also to be considered is a plausible intramolecular adduct formation with the proximal N-donor site on the triazine ring. However, empirical data from the literature for 9-BBN:pyridine in C_6D_6 suggest ^{11}B shifts around 0 ppm,[28] not to mention that unfavorable steric hindrance between the 9-BBN group and the triazine substitutions makes this adduct formation unlikely. However, it is worth noting that a patent report identifies a alkyl-9-BBN:2-picoline complex with a ^{11}B chemical shift at 30 ppm (no solvent is given)[29] and could give support to the assignment of the broad peak in spectra C of Figure S81.

More plausible is that the 9-BBN site is occupied by the simple alcohol (methanol or ethanol) present in excess in the NMR samples for solubility purposes. Unfortunately, because of the presence of bulk, direct correlations from a coordinating alcohol to the boron-side-arm is difficult to observe due to exchange processes. Even at lower temperatures (–40 °C), no attached alcohol could be distinguished from the bulk. However, examples of Boron-coordinated alcohol resonating near 18 ppm have been reported.[30]

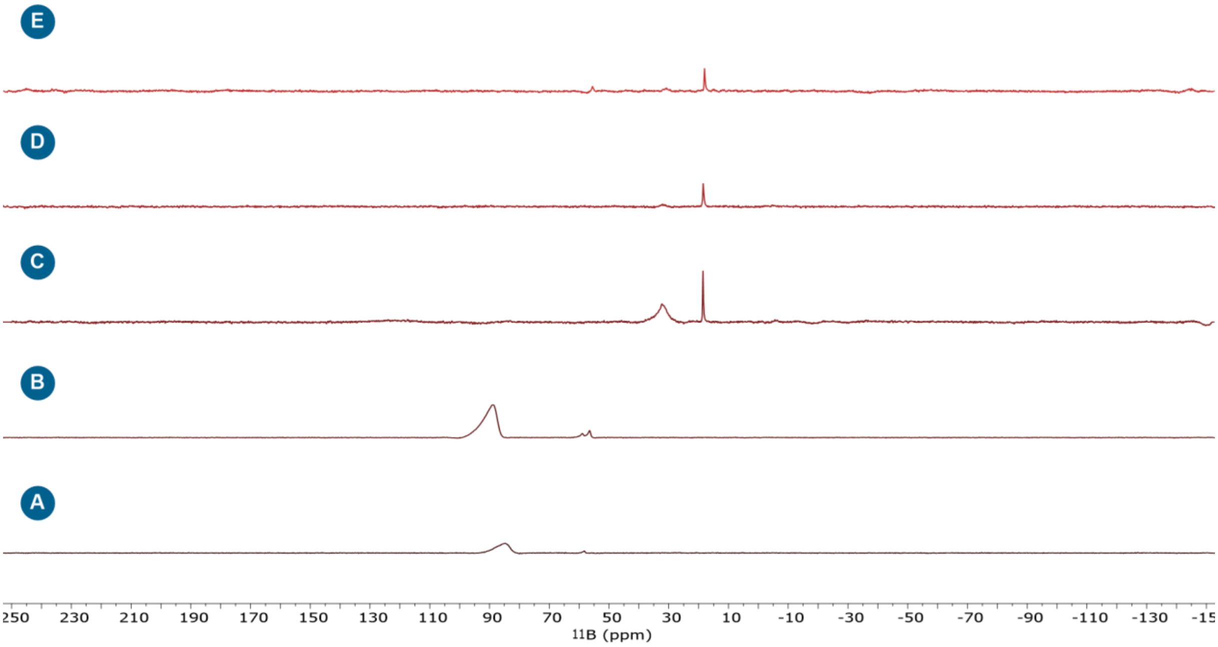

Figure S81 – Comparison of ^{11}B signal from 9-BBN side chain in different solvent systems. **Panel A**: Precursor PN^{tzn-B} **23** in C_6D_6 in the presence of CH_3CN. **Panel B**: Complex **1** in CD_2Cl_2. **Panel C**: Hydride complex **I** in C_6D_6:MeOH (80:20). **Panel D**: Hydride complex **I** in Toluene-d_8:MeOH (80:20). **Panel E**: Hydride complex **I** in C_6D_6:EtOH (80:20).

- **Complex 1 in the Presence of H_2**

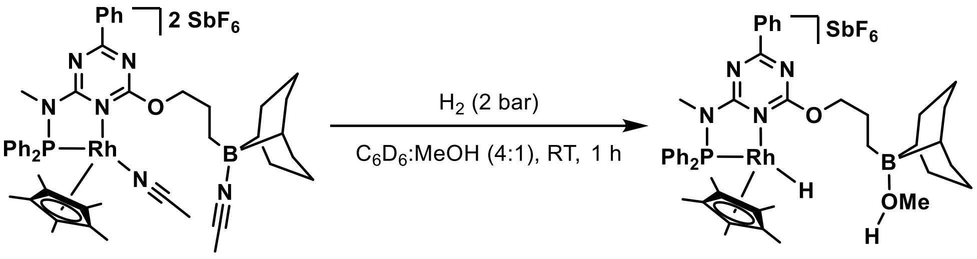

1 **I** (94%)

In an oven-dried NMR tube with a J. Young valve, complex **1** (20 mg, 0.01 mmol) and C_6D_6/MeOH (4:1, 0.5 mL) were charged under argon. The reaction mixture was frozen using liquid nitrogen, evacuated, and then pressurized with either H_2 (2 bar). The resulting solution was analyzed by (^{1}H,^{13}C, ^{31}P, ^{103}Rh, ^{11}B, and ^{15}N) NMR spectroscopy at room temperature. The obtained spectrum shows the appearance of a hydride signal at -10.68 ppm. Under the conditions chosen, there is no indication that a counterion H^+ is associated with this species. There was no unassigned signal that would integrate to 1xH or correlate to ^{13}C or ^{15}N. A possible explanation is that the H^+ is part of the Methanol-OH signal, which is in fast exchange as a labile H^+. The dynamic nature of this process makes it nearly impossible to observe unless one could freeze out the exchange.

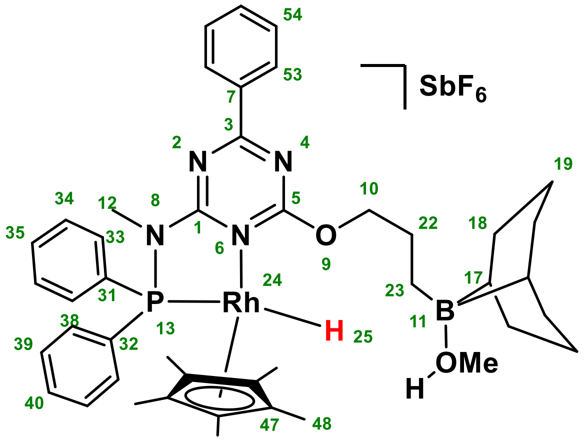

^{1}H NMR (600 MHz, C_6D_6, 273 K): δ = 8.45 (d, *J* =7.5, 2H, 53), 7.72 (dd, *J* =12.8, 7.3, 2H, 33), 6.98 (ddd, *J* =12.0, 8.1, 1.7, 2H, 38), 4.76 (dt, *J* =10.7, 7.6, 7.6, 1H, 10''), 4.50 (dt, *J* =10.7, 7.6, 7.6, 1H, 10'), 2.70 (d, *J* =4.8, 3H, 12), 2.17 - 2.07 (m, 2H, 19''), 2.02 - 1.94 (m, 4H, 18''), 1.92 - 1.83 (m, 6H, 18', 23), 1.79 - 1.66 (m, 2H, 19'), 1.34 (dd, *J* =3.3, 1.4, 15H, 48), 1.24 - 1.18 (m, 6H, 58), 0.97 - 0.89 (m, 2H, 17), 0.75 - 0.66 (m, 2H, 22), -10.68 (dd, *J* =33.5, 17.4, 1H, 25).

^{13}C NMR (151 MHz, C_6D_6, 273 K) δ = 171.9 (3), 170.9 (5), 169.3 (d, *J* =21.4, 1), 136.6 (d, *J* =15.1, 33), 134.8 (7), 134.4 (35, 55), 132.8 (d, *J* =2.3, 40), 132.1 (d, *J* =11.7, 38), 130.1 (d, *J* =11.0, 39), 130.0 (53), 129.9 (d, *J* =11.7, 34), 129.4 (d, *J* =20.5, 32), 127.4 (d, *J* =60.9, 31), 117.5 (57), 101.6 (47), 75.4 (10), 33.7 (d, *J* =4.6, 12), 32.8 (18), 26.2 (19), 24.7 (23), 24.4 (17), 15.0 (22), 10.2 (48), 0.4 (58).

^{31}P NMR (243 MHz, C_6D_6, 273 K) δ = 115.40 (d, *J* =154.5, 13).

^{11}B NMR (193 MHz, C_6D_6, 273 K) δ = 32.23, 18.52 (11).

^{15}N NMR (61 MHz, C_6D_6, 273 K) δ = -138.02 (56).

^{103}Rh NMR (16 MHz, C_6D_6, 273 K) δ = -72.02 (d, *J* =149.8, 24).

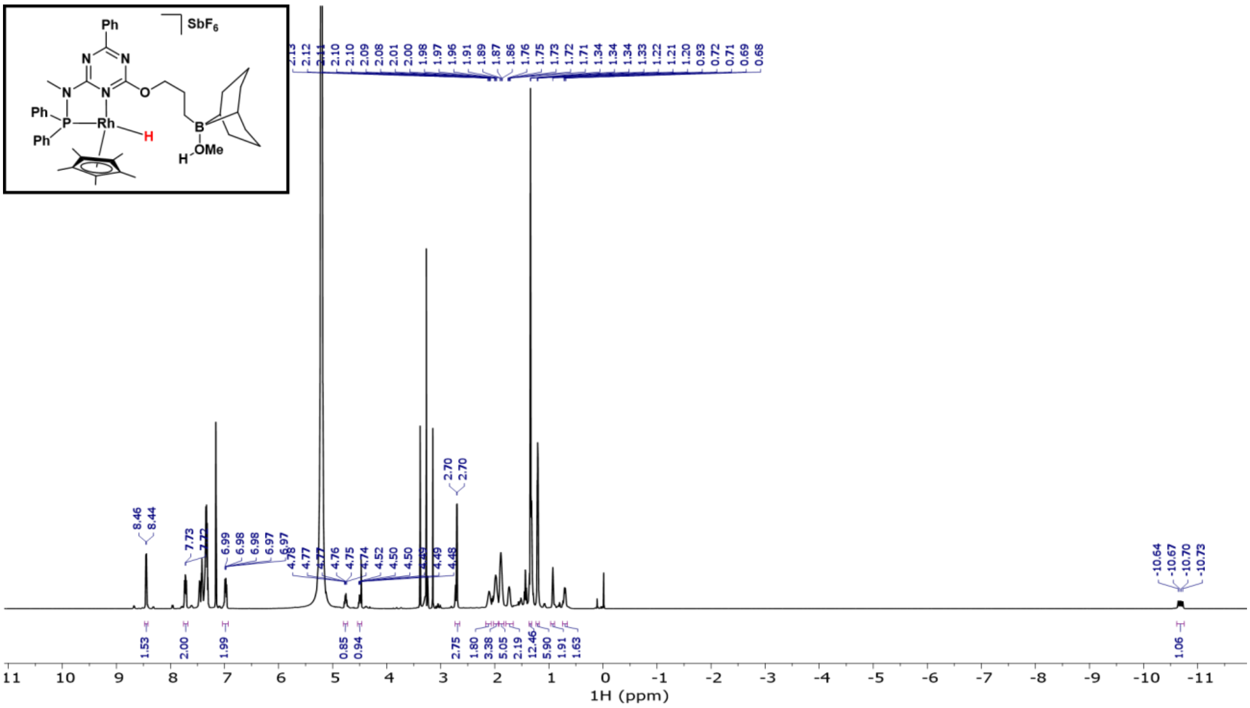

Figure S82 – ^{1}H NMR (600 MHz, C_6D_6, 273 K) spectrum of the crude reaction mixture containing **1** and H_2 (2 bar).

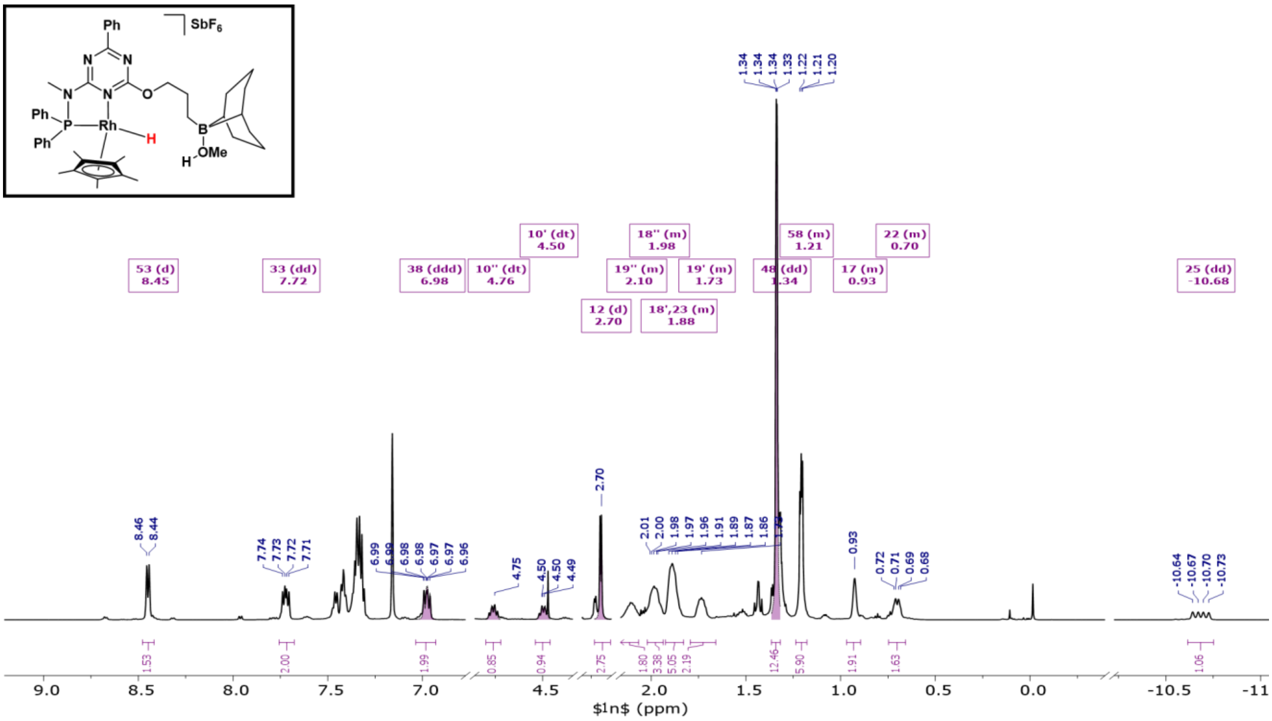

Figure S83 – ^{1}H NMR (600 MHz, C_6D_6, 273 K) cut spectrum of the crude reaction mixture containing **1** and H_2 (2 bar).

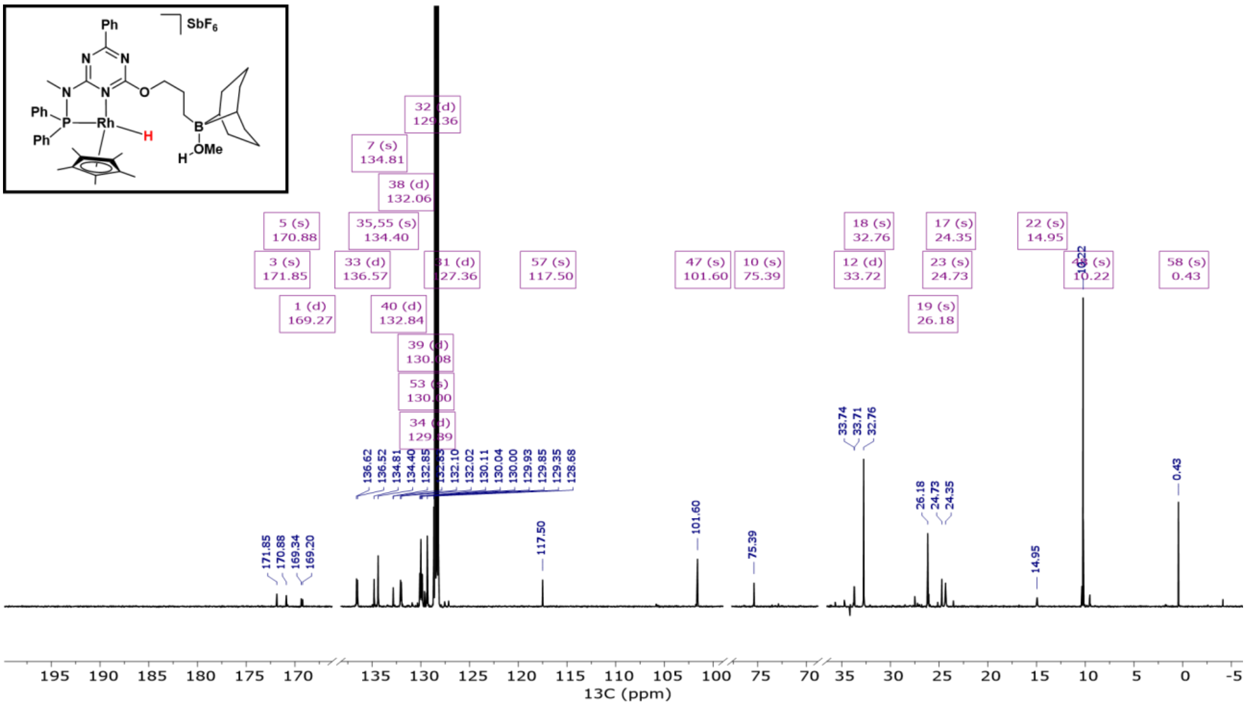

Figure S84 – ^{13}C NMR (151 MHz, C_6D_6, 273 K) spectrum of the crude reaction mixture containing **1** and H_2 (2 bar).

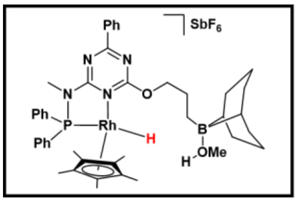

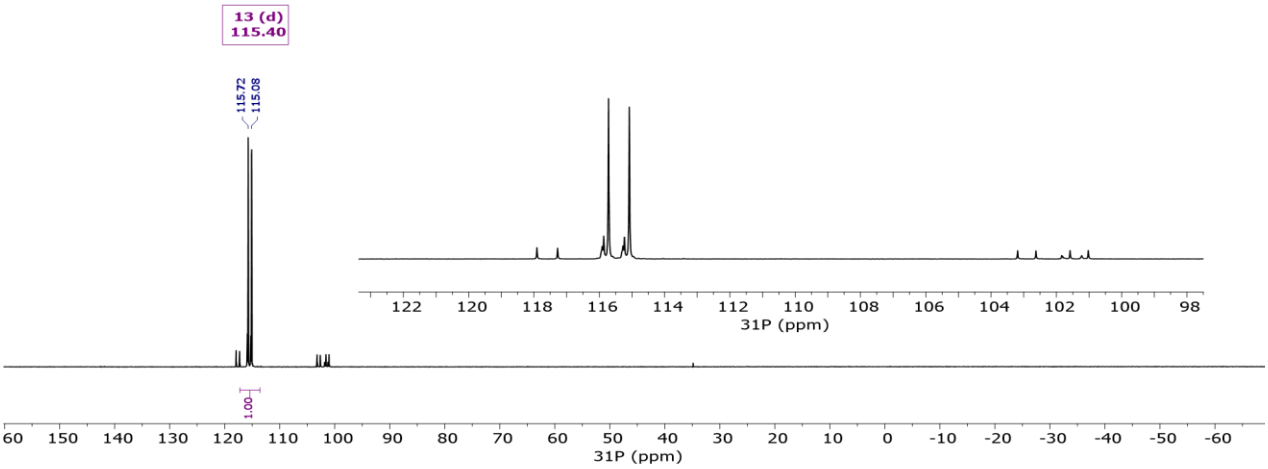

Figure S85 – $^{31}P\{^{1}H\}$NMR (243 MHz, C_6D_6, 273 K) spectrum of the crude reaction mixture containing **1** and H_2 (2 bar).

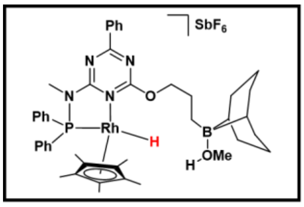

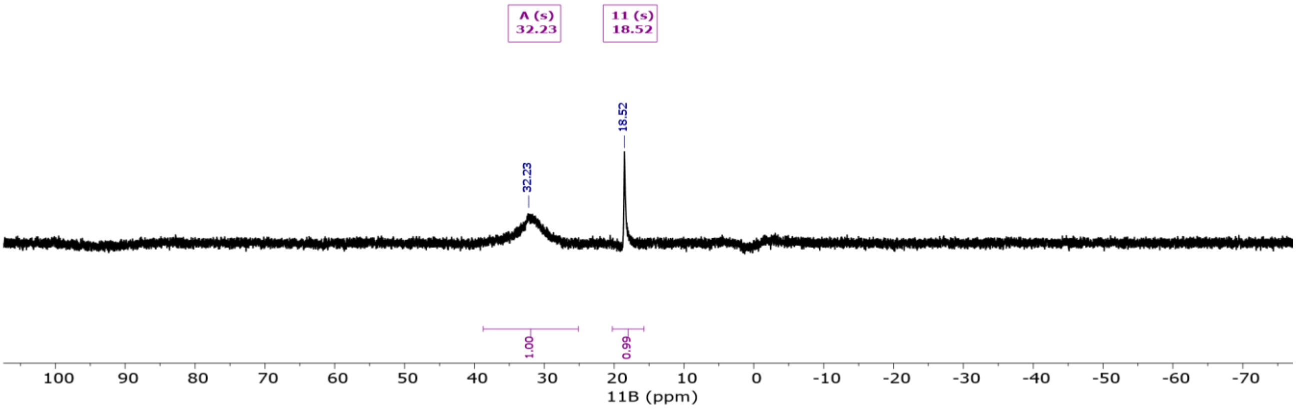

Figure S86 – ^{11}B NMR (193 MHz, C_6D_6, 273 K) spectrum of the crude reaction mixture containing **1** and H_2 (2 bar).

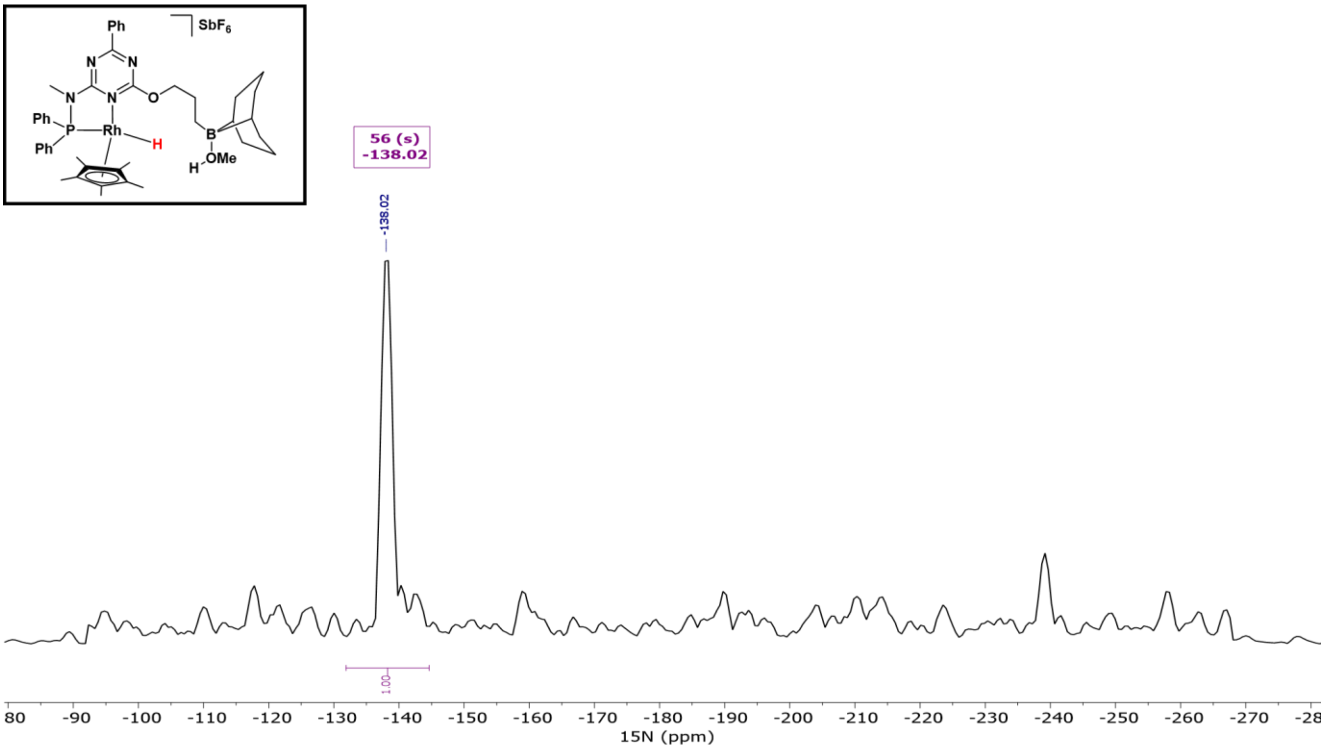

Figure S87 – ^{15}N NMR (61 MHz, C_6D_6, 273 K) spectrum of the crude reaction mixture containing **1** and H_2 (2 bar).

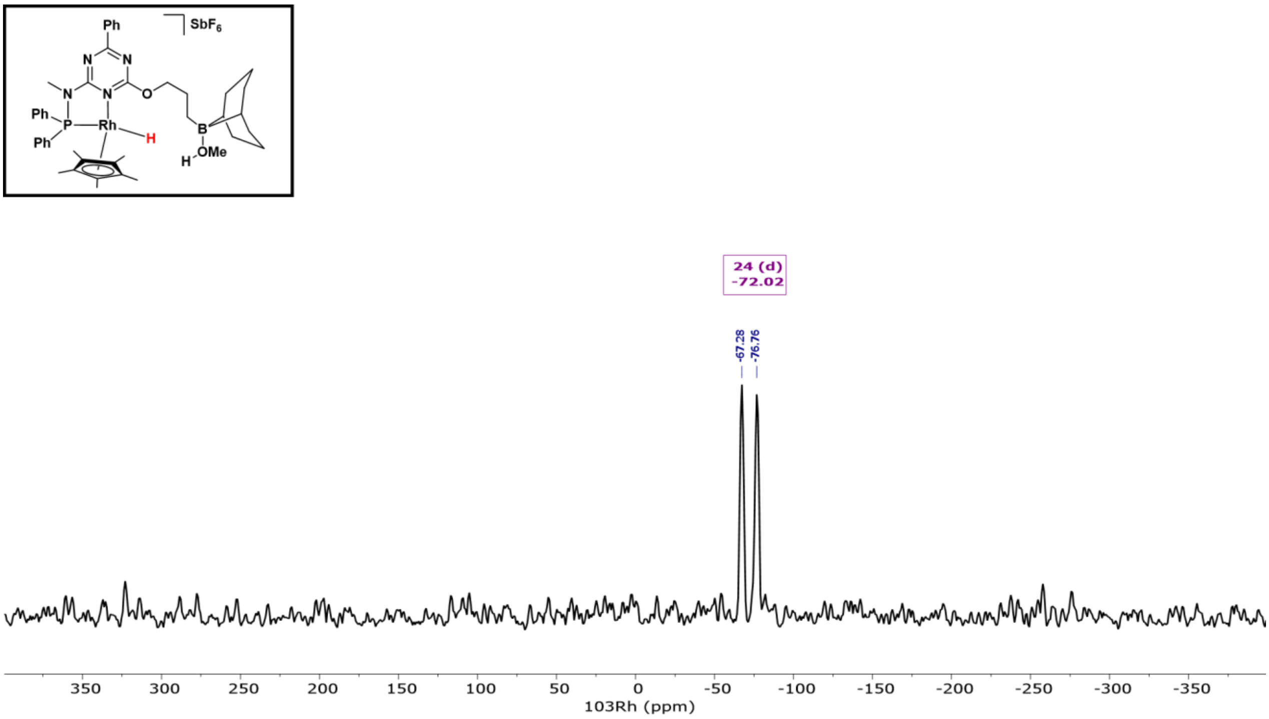

Figure S88 – ^{103}Rh NMR (16 MHz, C_6D_6, 273 K) spectrum of the crude reaction mixture containing **1** and H_2 (2 bar).

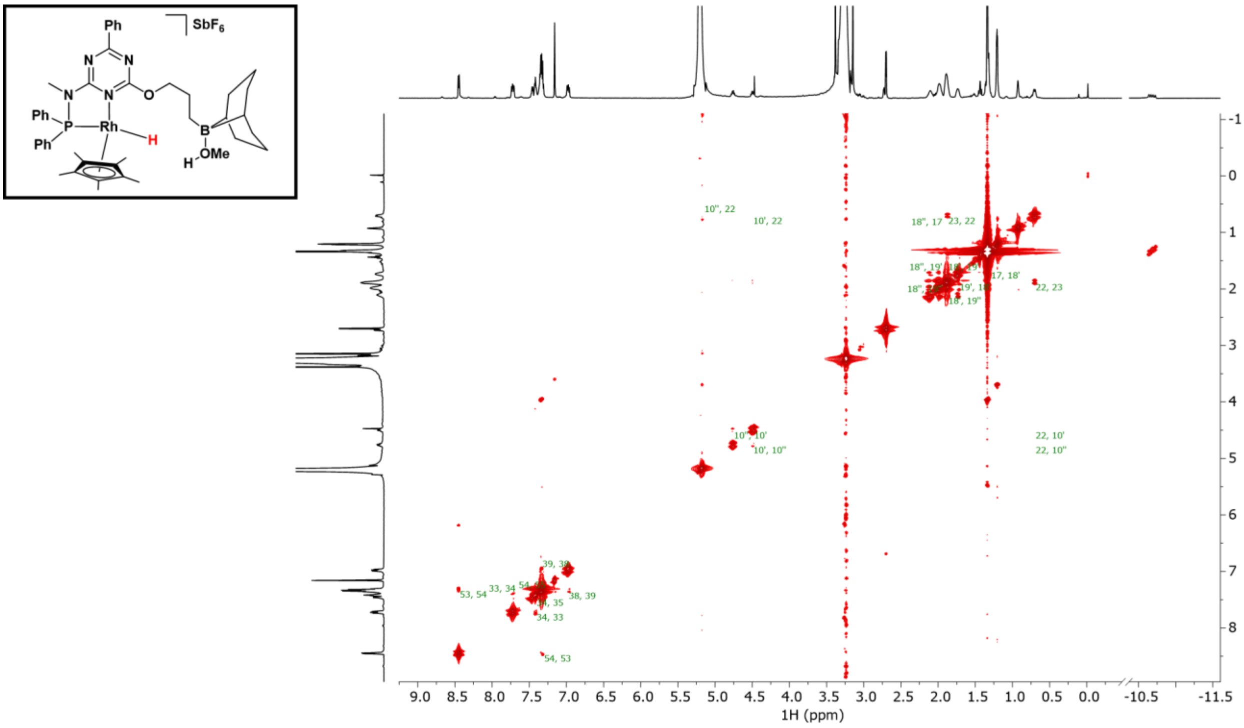

Figure S89 – ¹H-¹H COSY NMR (600 MHz, C_6D_6, 273 K) spectrum of the crude reaction mixture containing **1** and H_2 (2 bar).

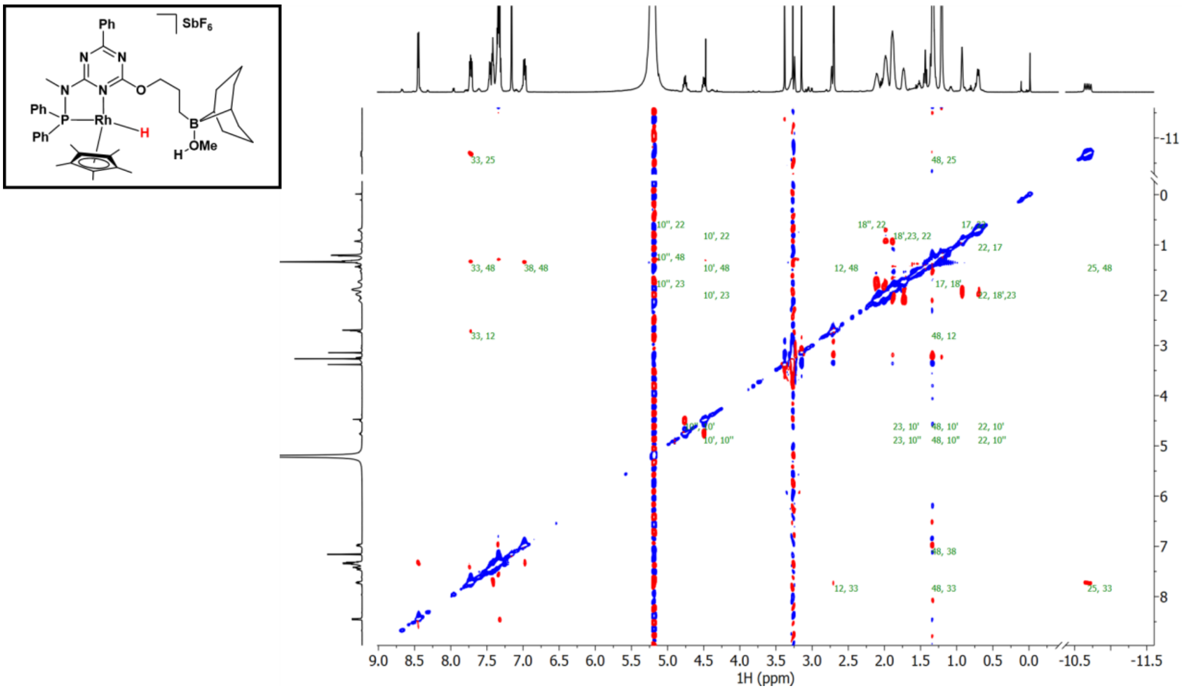

Figure S90 – ¹H-¹H NOESY NMR (600 MHz, C_6D_6, 273 K) spectrum of the crude reaction mixture containing **1** and H_2 (2 bar).

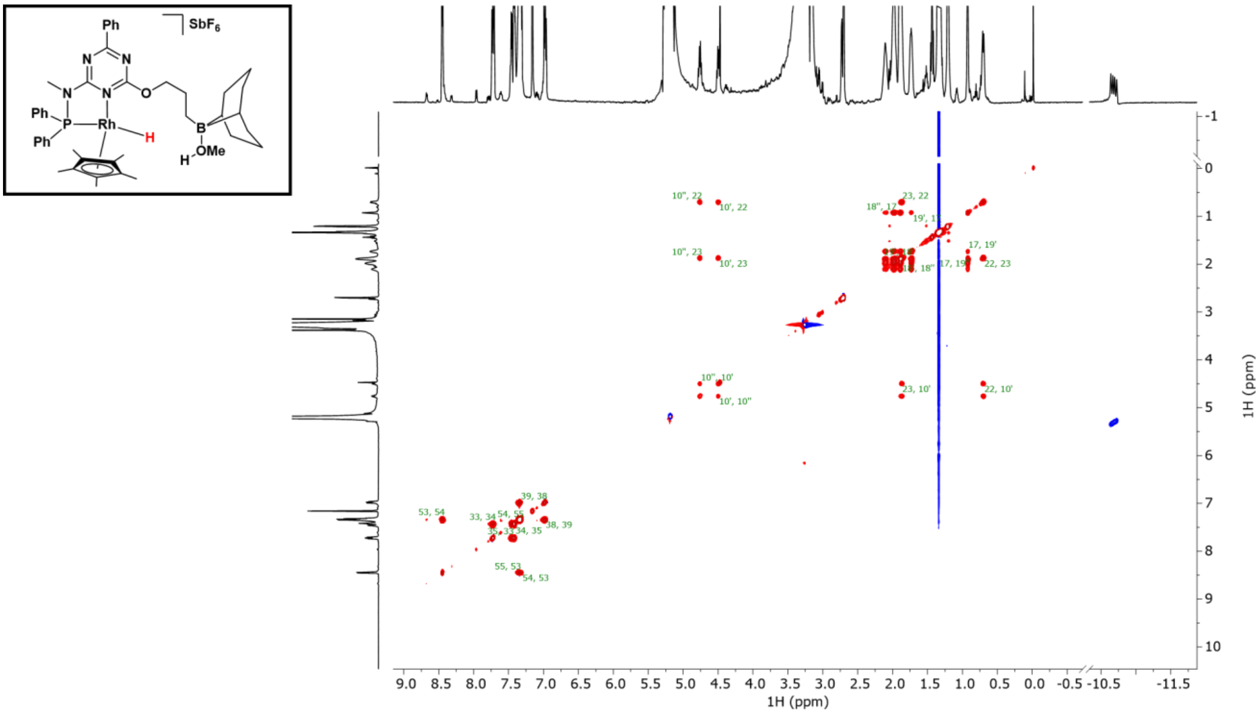

Figure S91 – ^{1}H-^{1}H TOCSY NMR (600 MHz, C_6D_6, 273 K) spectrum of the crude reaction mixture containing **1** and H_2 (2 bar).

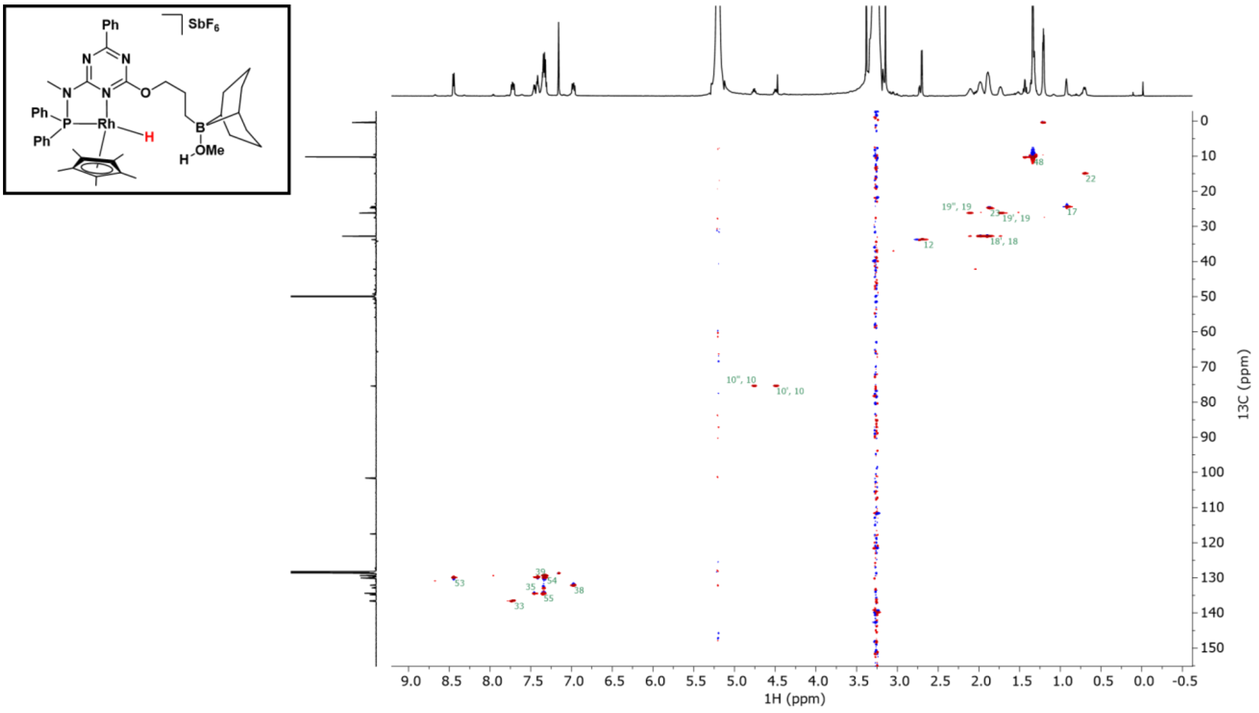

Figure S92 – ^{1}H-^{13}C HSQC NMR (600, 151 MHz, C_6D_6, 296 K) spectrum of the crude reaction mixture containing **1** and H_2 (2 bar).

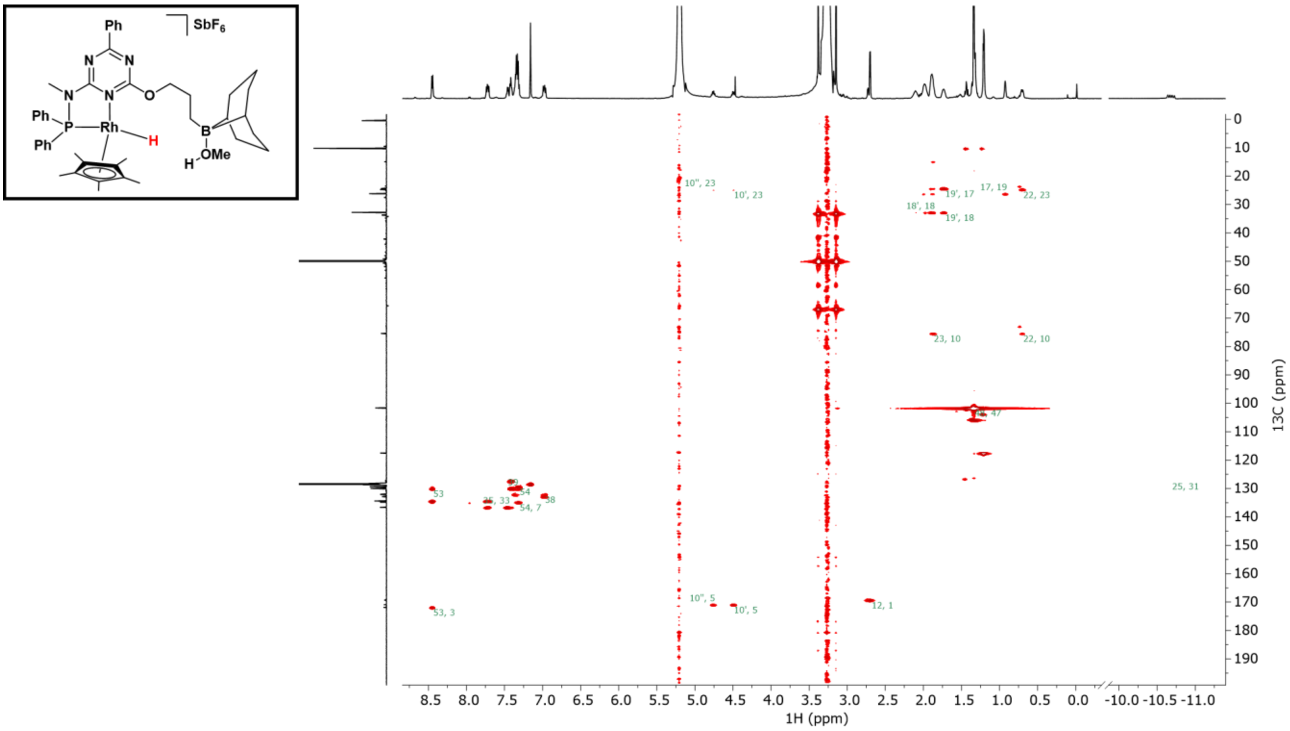

Figure S93 – ^{1}H-^{13}C HMBC NMR (600, 151 MHz, C_6D_6, 273 K) spectrum of the crude reaction mixture containing **1** and H_2 (2 bar).

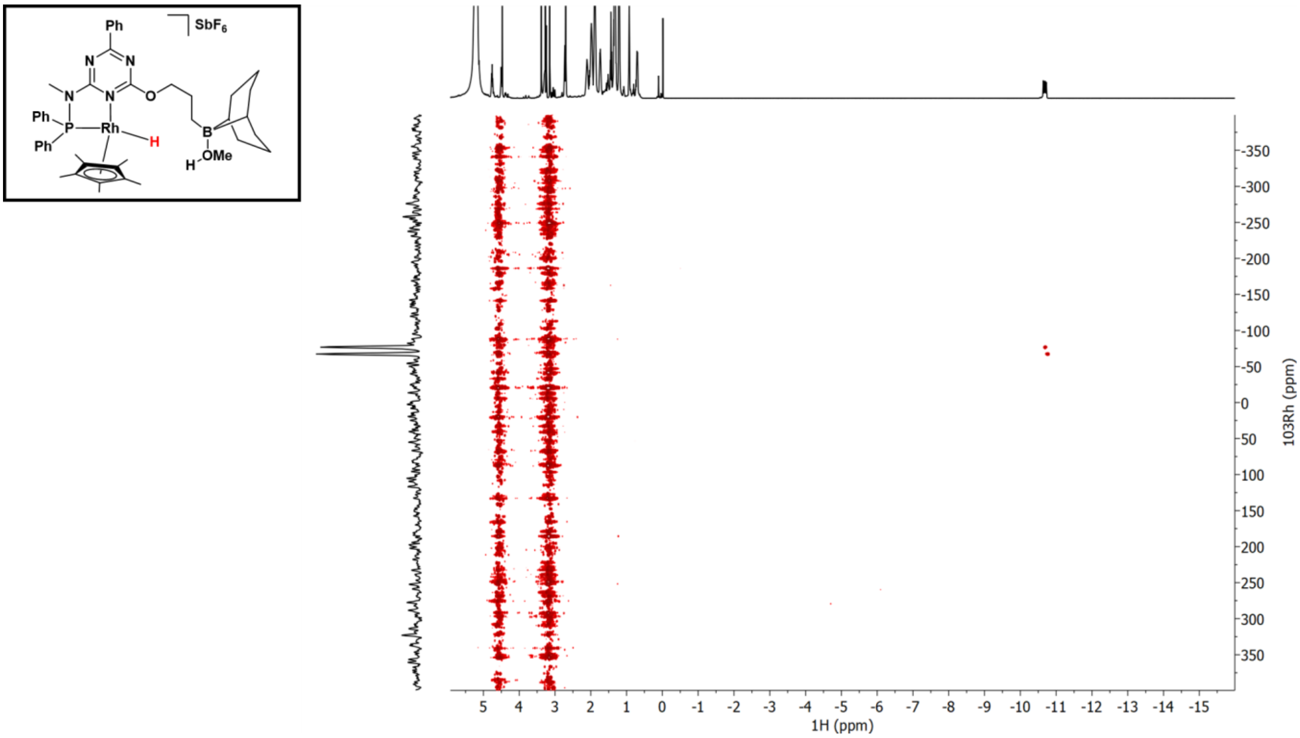

Figure S94 – ^{1}H-^{103}Rh HMBC NMR (600, 16 MHz, C_6D_6, 273 K) spectrum of the crude reaction mixture containing **1** and H_2 (2 bar).

- **Complex 1 in the Presence of D_2**

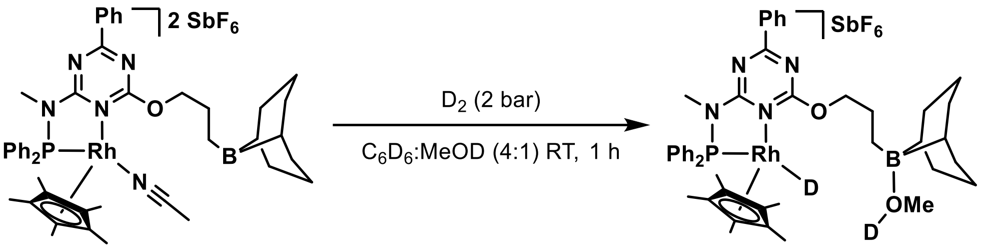

1 (80%)

In an oven-dried NMR tube with J. Young valve, complex **1** (20 mg, 0.01 mmol) and C_6D_6/MeOD (4:1, 0.5 mL) were charged under argon. The reaction mixture was frozen using liquid nitrogen, evacuated, and then pressurized with D_2 (2 bar). After 1 hour, the resulting solution was analyzed by NMR spectroscopy at the indicated temperature. The data shows the formation of the [Rh]–D species in 80%.

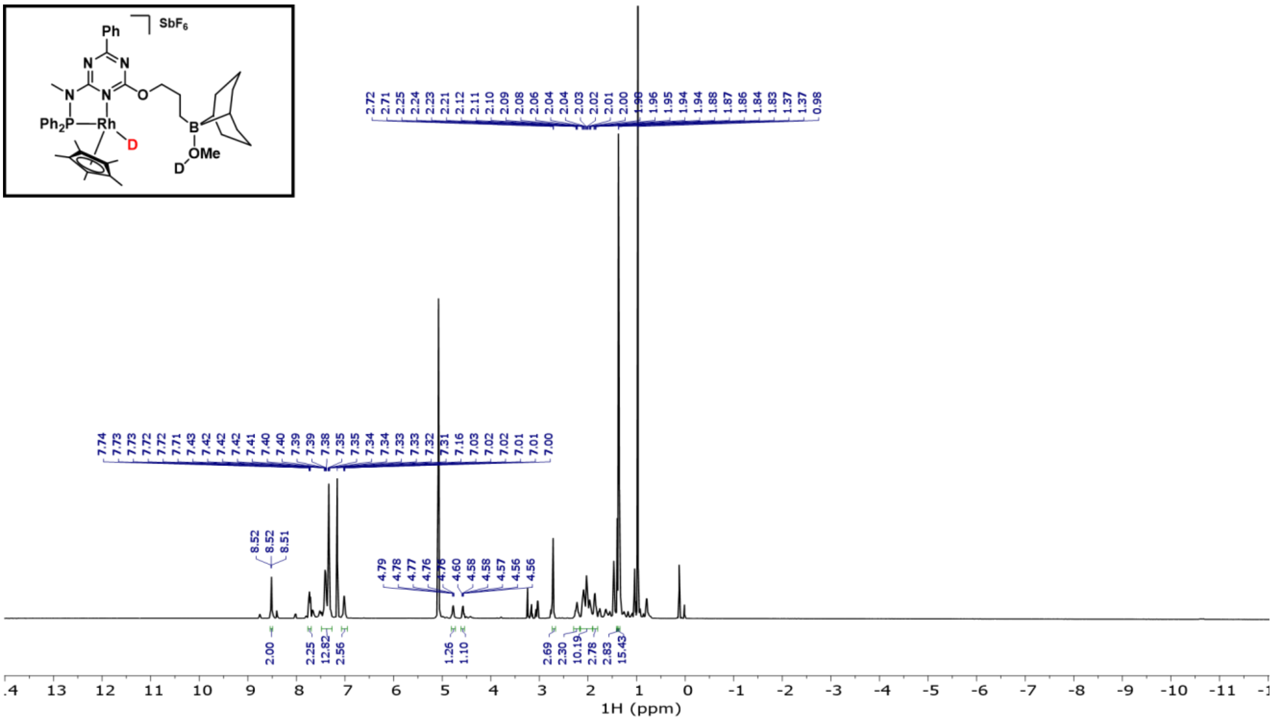

Figure S95 – ^{1}H NMR (600 MHz, C_6D_6, 273 K) spectrum of the crude reaction mixture containing **1** and D_2 (2 bar).

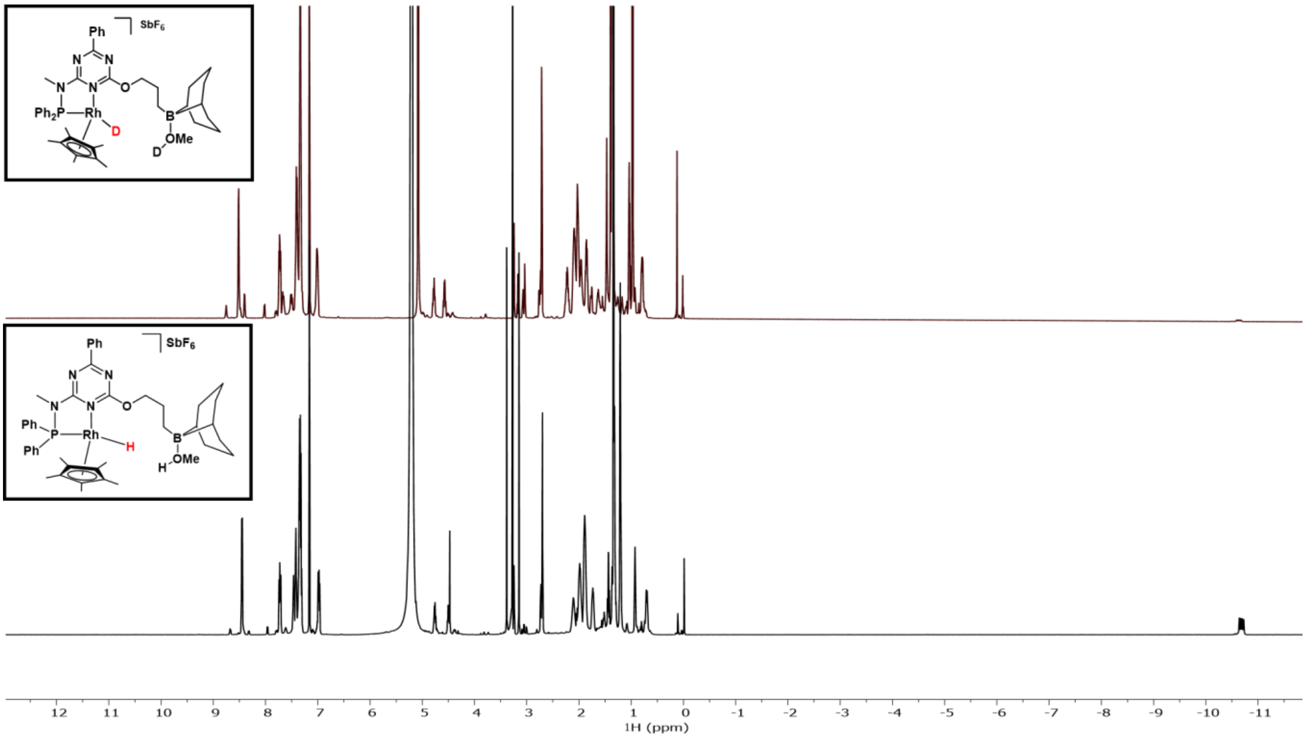

Figure S96 – ^{1}H NMR (600 MHz, C_6D_6, 273 K) stacked spectrum (Top: Rh-D, Bottom: Rh-H) of the crude reaction mixture containing **1** and D_2 (2 bar).

The sample prepared using D_2 instead of H_2 also showed the corresponding ^{1}H-hydride signal (about 9%), indicating that a "scrambling" occurs under these conditions.

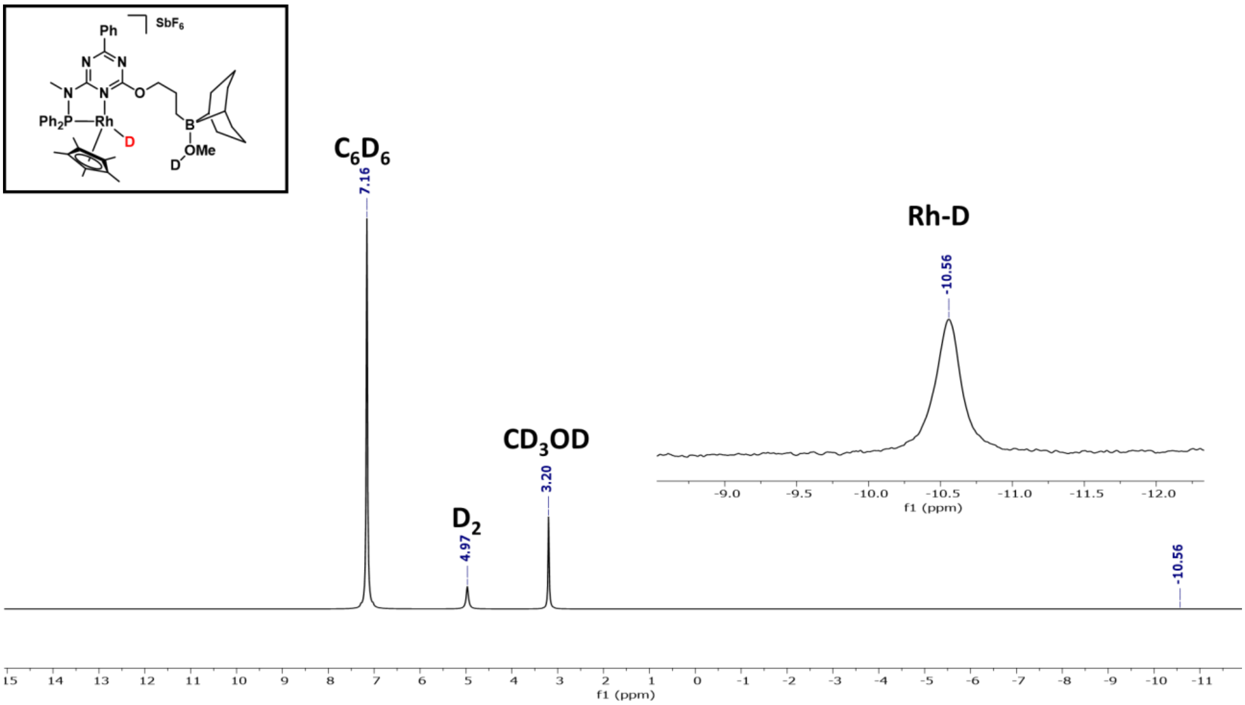

Figure S97 – ^{2}H NMR (92 MHz, C_6D_6, 273 K) cut spectrum of the crude reaction mixture containing **1** and D_2 (2 bar).

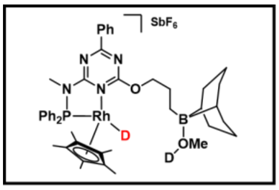

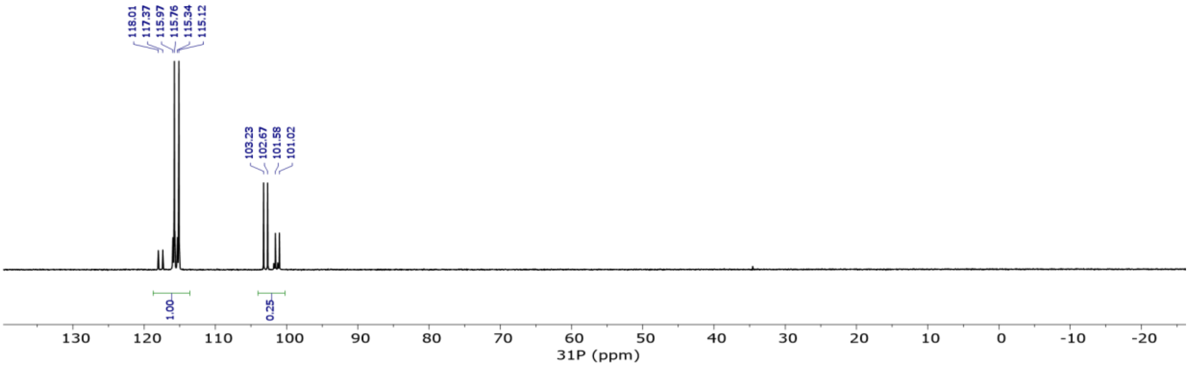

Figure S98 – ^{31}P{^{1}H} NMR (243 MHz, C_6D_6, 273 K) spectrum of the crude reaction mixture containing **1** and D_2 (2 bar).

- **The Reaction of Complex 5 with H_2**

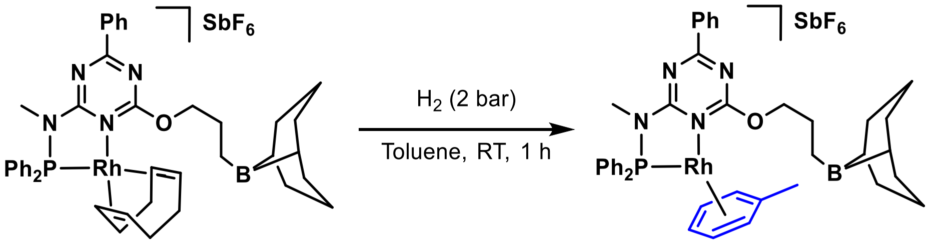

In a Schlenk flask under argon, complex **5** (810.1 mg, 0.814 mmol) in toluene (4 ml) was subjected to freeze-pump-thaw (three cycles). The flask was pressurized with H_2 (3 bar), causing a dark red oil to form and precipitate. The reaction mixture was stirred at room temperature for an additional two hours. Dropwise transfer of the mixture to a pentane solution (10 mL) under vigorous stirring led to precipitation of a dark red solid. The precipitate was washed with pentane (3 × 5 mL) and dried *in vacuo* to give the toluene adduct a dark red solid (778.2 mg, 0.795 mmol, 98%).

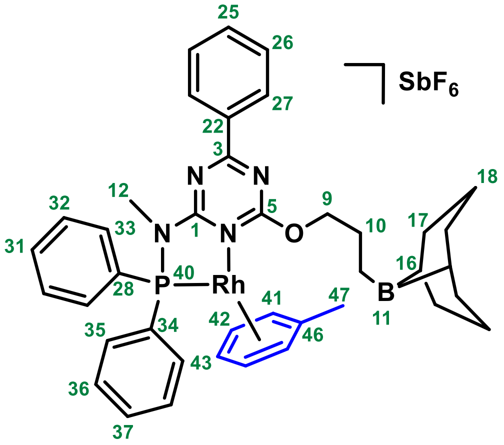

^{1}H NMR (400 MHz, CD_2Cl_2, 296 K): δ = 8.45 (d, *J* =7.1, 2H, 27), 7.81 - 7.71 (m, 4H, 33, 35), 7.71 - 7.58 (m, 7H, 25, 31, 32, 36, 37), 7.56 - 7.47 (m, 2H, 26), 6.84 (t, *J* =6.4, 6.4, 1H, 43), 6.27 (t, *J* =6.5, 6.5, 2H, 42), 5.89 (d, *J* =6.4, 2H, 41), 4.79 - 4.66 (m, 2H, 9), 3.09 (d, *J* =5.6, 3H, 12), 1.97 (s, 3H, 47), 1.97 - 1.68 (m, 12H, 16, 17, 18''), 1.67 - 1.54 (m, 2H, 21), 1.27 (tt, *J* =10.2, 10.2, 3.1, 3.1, 2H, 18').

^{13}C{^{1}H} NMR (101 Hz, CD_2Cl_2): 170.8 (3), 170.5 (5), 168.6 (d, *J* =21.7, 1), 134.6 (22), 134.2 (25), 133.3 (d, *J* =2.5, 28), 132.9, 132.8, 130.7 (dd, *J* =58.8, 1.4, 34), 130.1, 130.0, 129.7 (27), 129.5, 129.3 (26), 128.7, 116.7 (d, *J* =3.4, 46), 103.8 (t, *J* =2.9, 2.4, 43), 99.5 (t, *J* =3.8, 3.8, 42), 97.2 (t, *J* =3.2, 2.6, 41), 73.2 (9), 33.8 (17), 33.1 (d, *J* =3.9, 12), 31.9 (16), 24.4 (10), 23.7 (18), 23.7 (21), 20.2 (47).

^{31}P NMR (162 MHz, CD_2Cl_2, 296 K): δ 119.5 (d, *J* = 208.9 Hz, 40).

^{11}B NMR (128 MHz, CD_2Cl_2, 296 K): δ 87.4 (brs, 1B, 11).

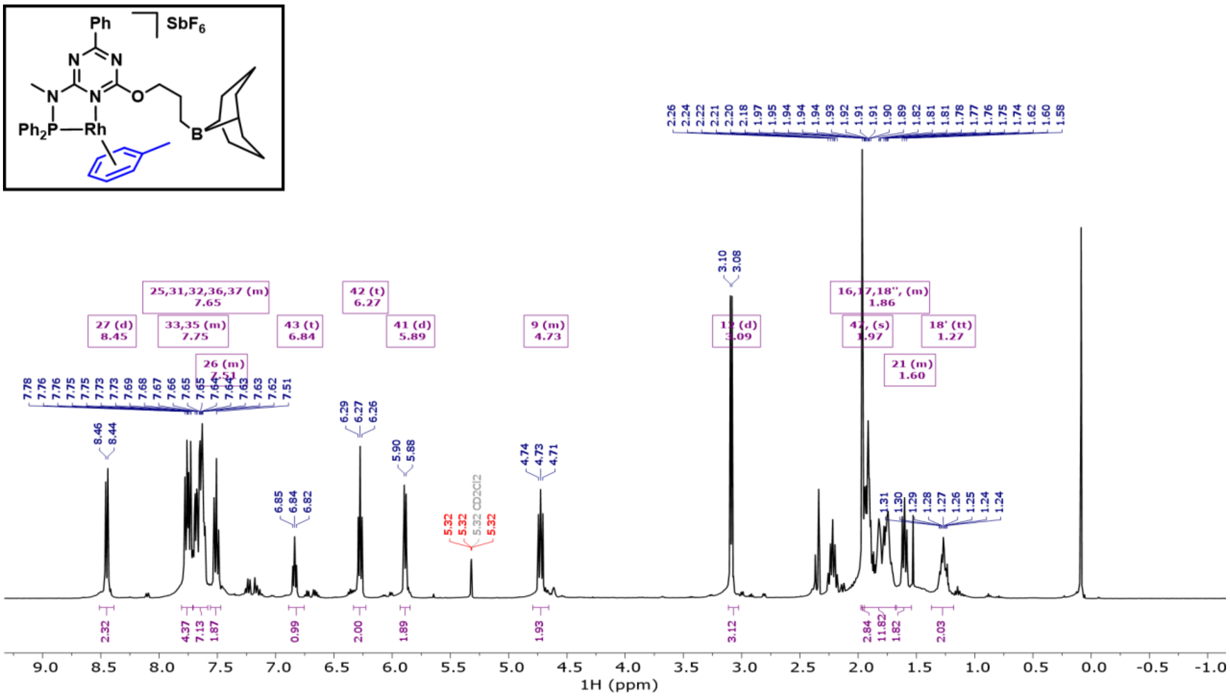

Figure S99 – ¹H NMR (400 MHz, CD_2Cl_2, 296 K) spectrum of the compound.

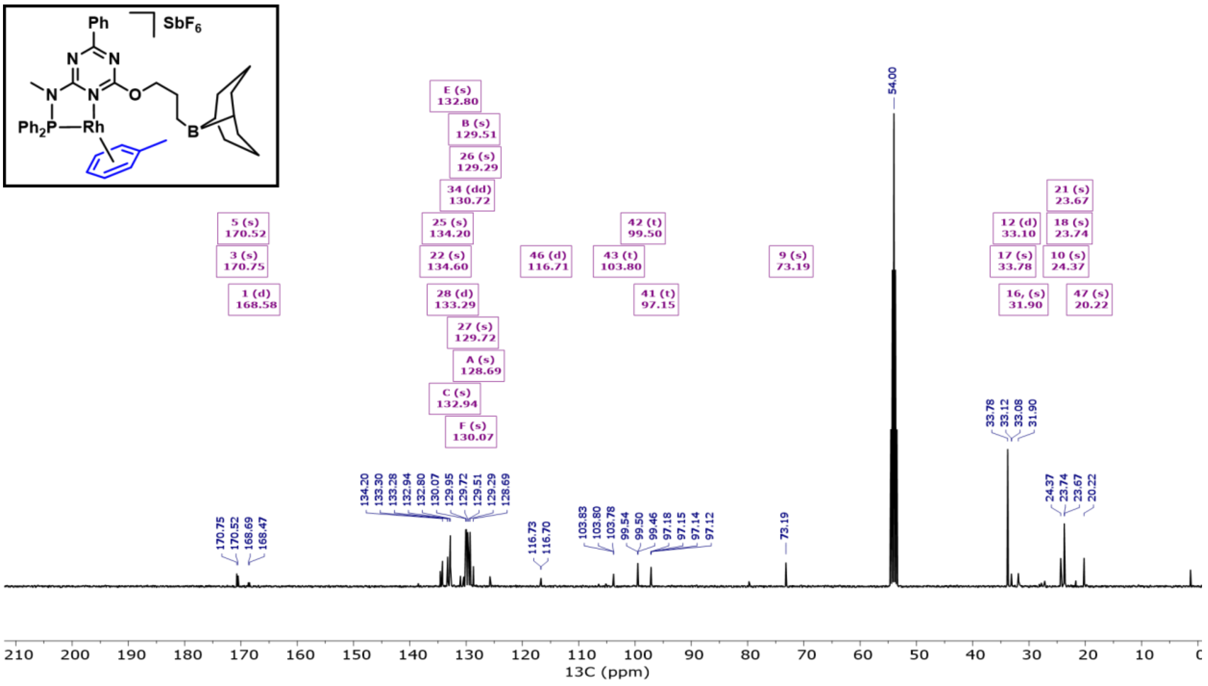

Figure S100 – ¹³C{¹H} NMR (101 MHz, CD_2Cl_2, 296 K) spectrum of compound.

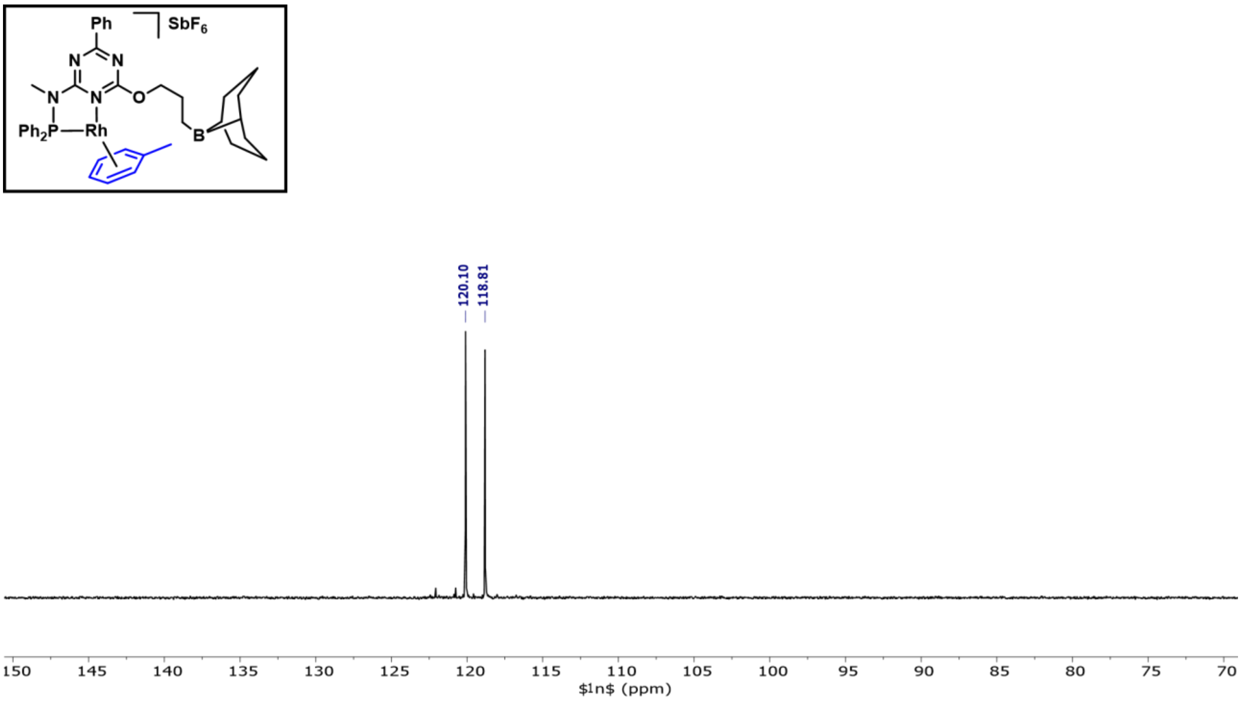

Figure S101 – $^{31}P\{^1H\}$ NMR (162 MHz, CD_2Cl_2, 296 K) spectrum of the compound.

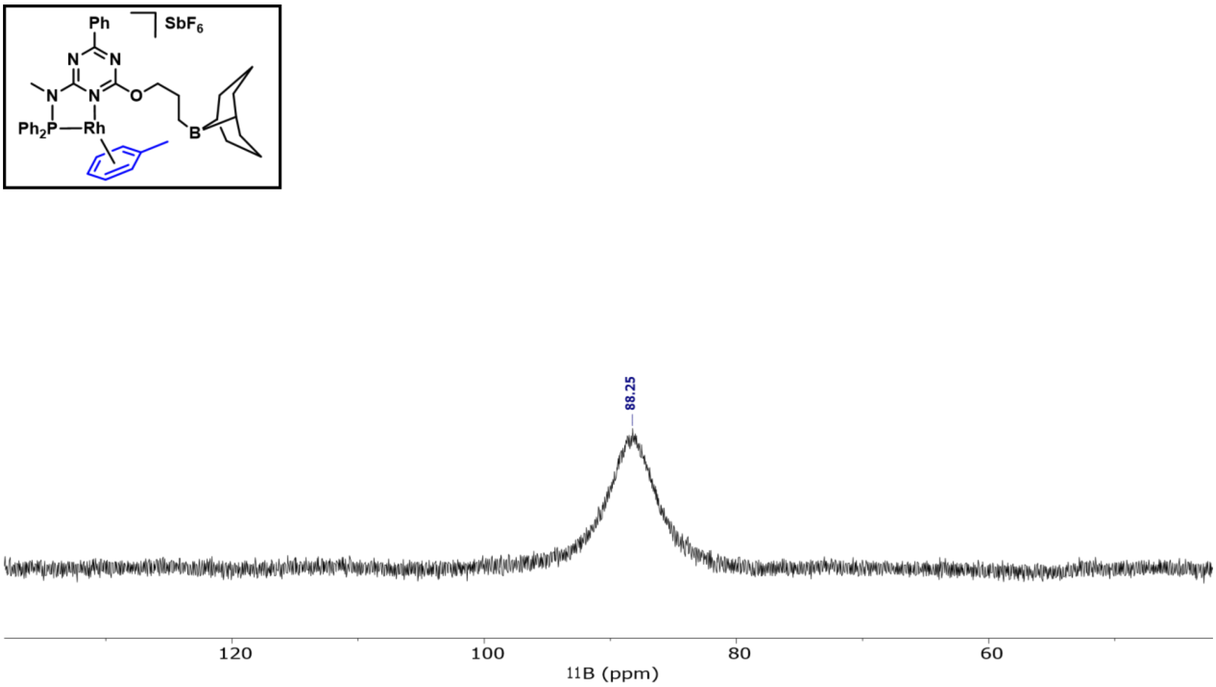

Figure S102 – ^{11}B NMR (128 MHz, CD_2Cl_2, 296 K) spectrum of the compound.

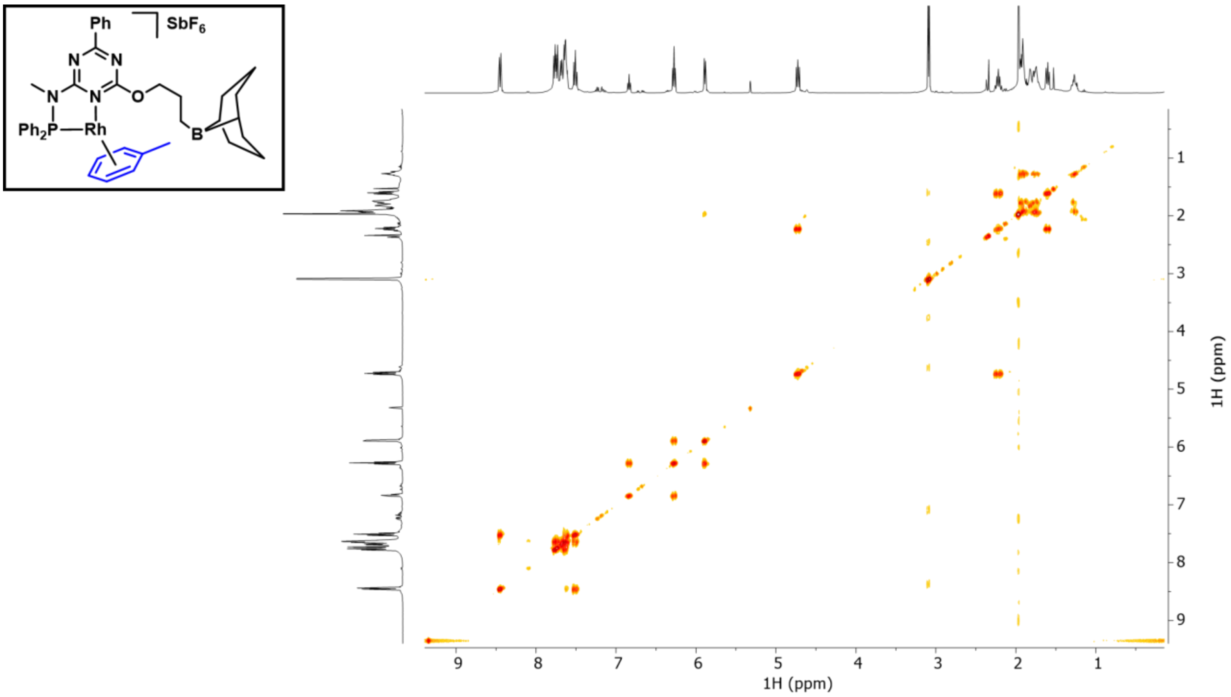

Figure S103 – ^{1}H-^{1}H COSY NMR (400 MHz, CD_2Cl_2, 296 K) spectrum of the compound.

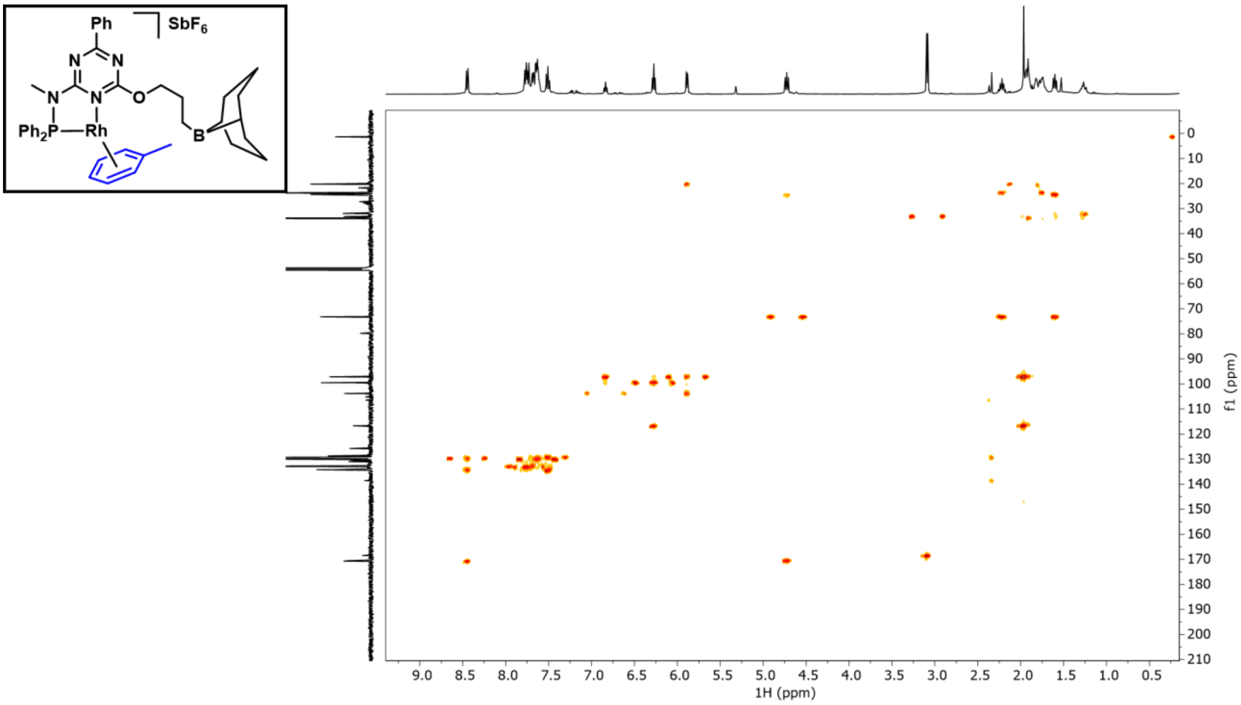

Figure S104 – ^{1}H-^{13}C HMBC NMR (400, 101 MHz, CD_2Cl_2, 296 K) spectrum of the compound.

9. Role of Secondary Coordination Sphere

We also performed the reaction in MeOH to illustrate how the boron arm works. Similar to THF, we observe the formation of hydroxylamine products in methanol. The change in selectivity may also be explained by solvent binding at boron, inhibiting substrate activation and, therefore, its further reduction to the aniline product platform. Contrary to the chemical shift of complex **1** in DCM (80.3 ppm), where the boron does not have any solvent attached, the ^{11}B spectra of complex **1** (18.41 ppm) and [Rh]-H (18.60 ppm) have very similar chemical shifts. In agreement with previously published literature,[31] the results suggest a similar environment around the boron center, more specifically MeOH binding at boron.

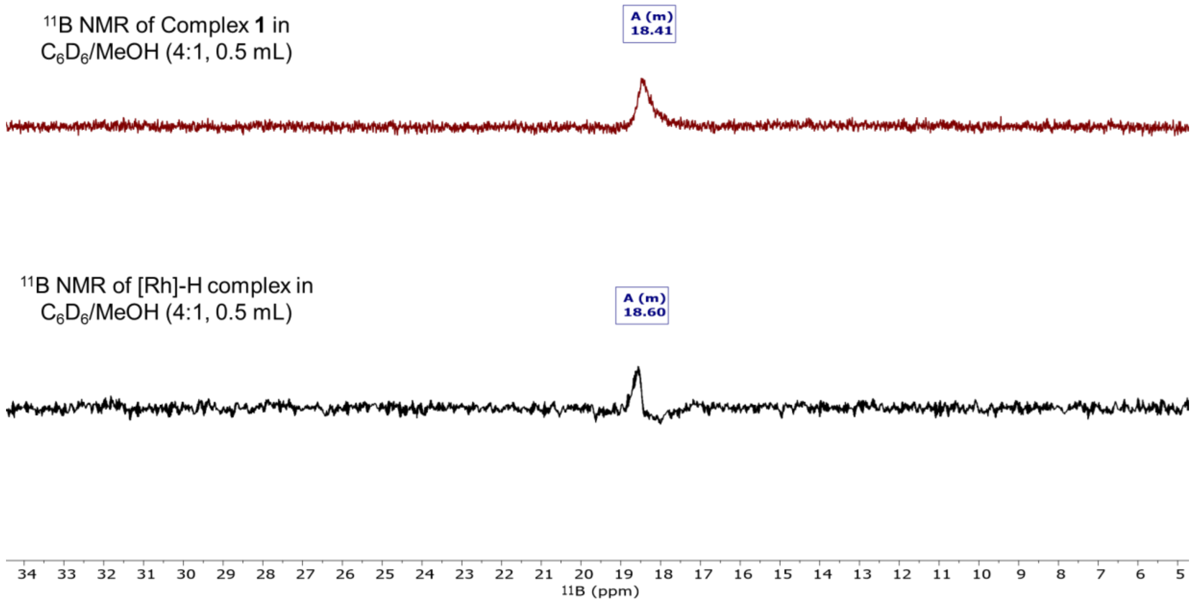

Figure S105 – Stacked ^{11}B NMR (128 MHz, C_6D_6, 296 K) spectrum of the complex **1** (Top), [Rh]–H (Bottom), both showing a similar signal at around 18.5ppm.

In the case of methanol, we have optimized the reaction conditions for hydroxylamine formation as well as investigated the catalytic activity of a wide range of substrates. Due to the better performance of the catalyst in THF, these data have been included in the SI (page S121-131).

- **Reaction Optimization Using MeOH as the Reaction Solvent**

Reaction Optimization

Screening Overview

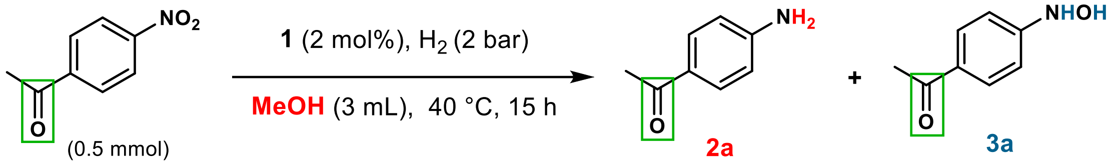

Entry	Deviation from above	Yields (%)[a] 2a:3a
**1**	**None**	**6:91**
2	RT	5:75
3	60 °C	26:74
4	5 h	8:62
5	10 h	5:72
6	20 h	11:83
7	0 bar	0:0
8	1 bar	4:71
9	3 bar	18:68
10	0 mol%	0:0
11	0.5 mol%	3:15
12	1 mol%	6:29
13	3 mol%	15:80
14	Ligand **23** instead of **1**	0:0

[a]Yields are based on ^{1}H NMR relative to mesitylene (0.5 mmol) as an internal standard.

- **Substrate Scope in MeOH as the Reaction Solvent**

Substrate Scope

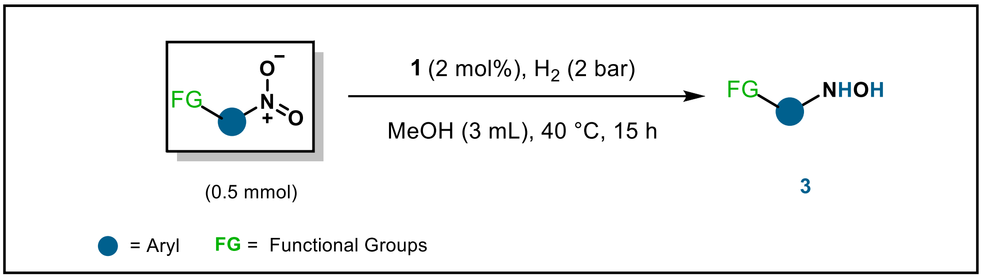

(NMR Yield, %)

Hydroxylamines

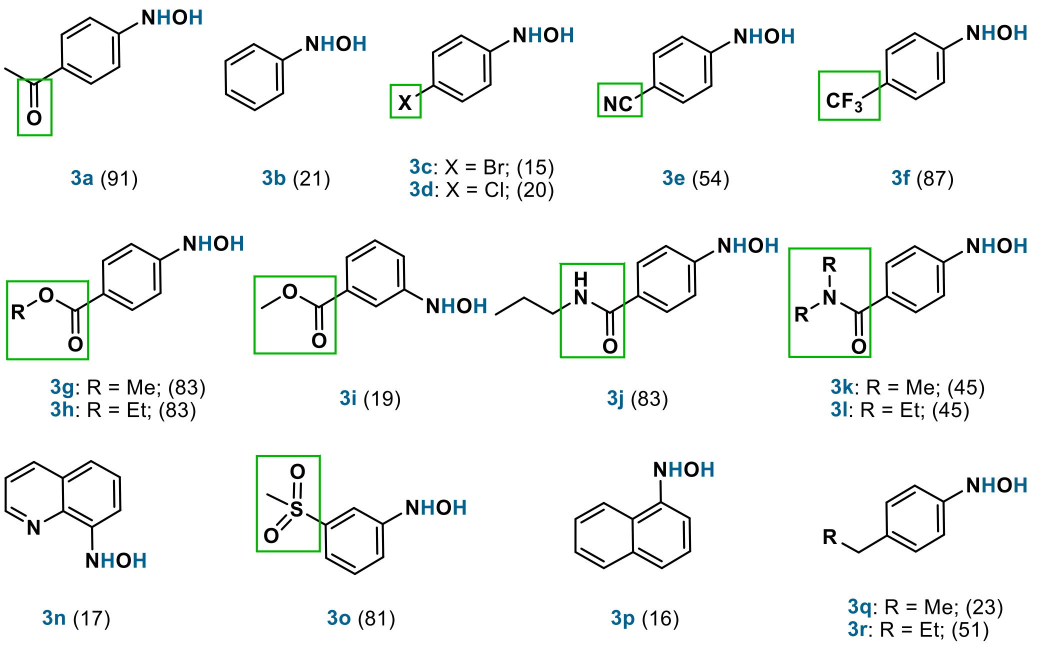

[a]Yields are based on ^{1}H NMR relative to mesitylene (0.5 mmol) as an internal standard.

- **NMR Spectra of the Crude Reaction Mixtures Using MeOH as Solvent for the Substrate Scope**

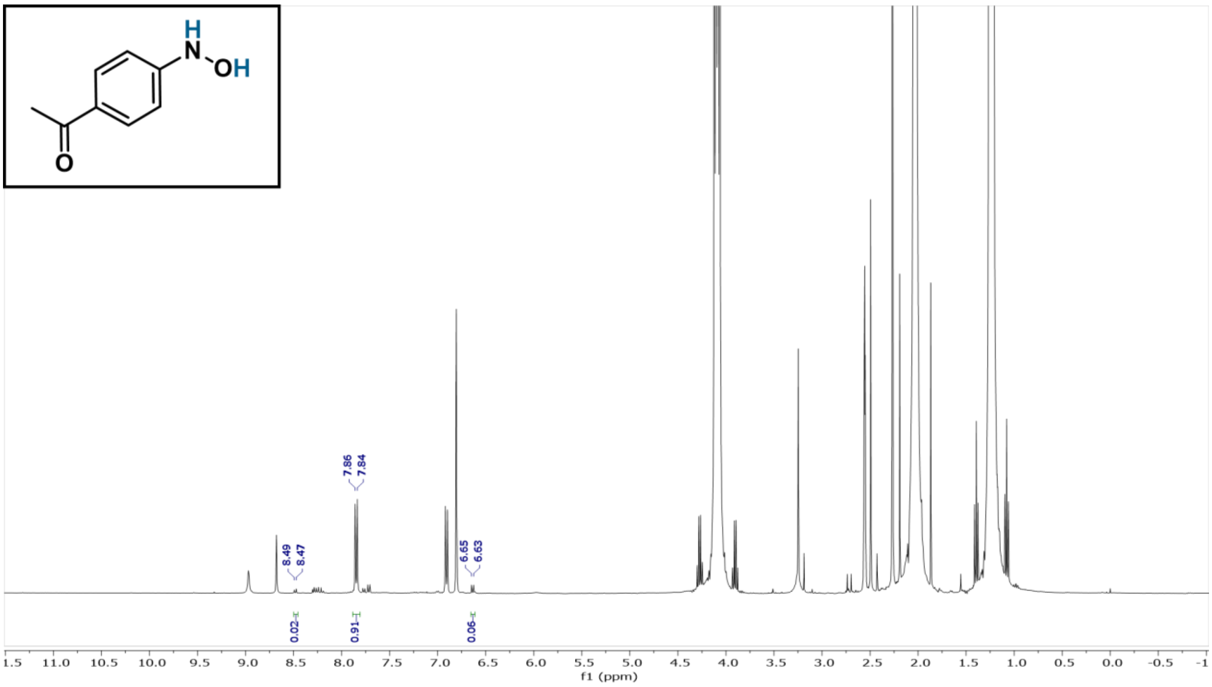

Figure S106 – ^{1}H NMR (400 MHz, DMSO-d_6, 296 K) crude spectrum of compound **3a**.

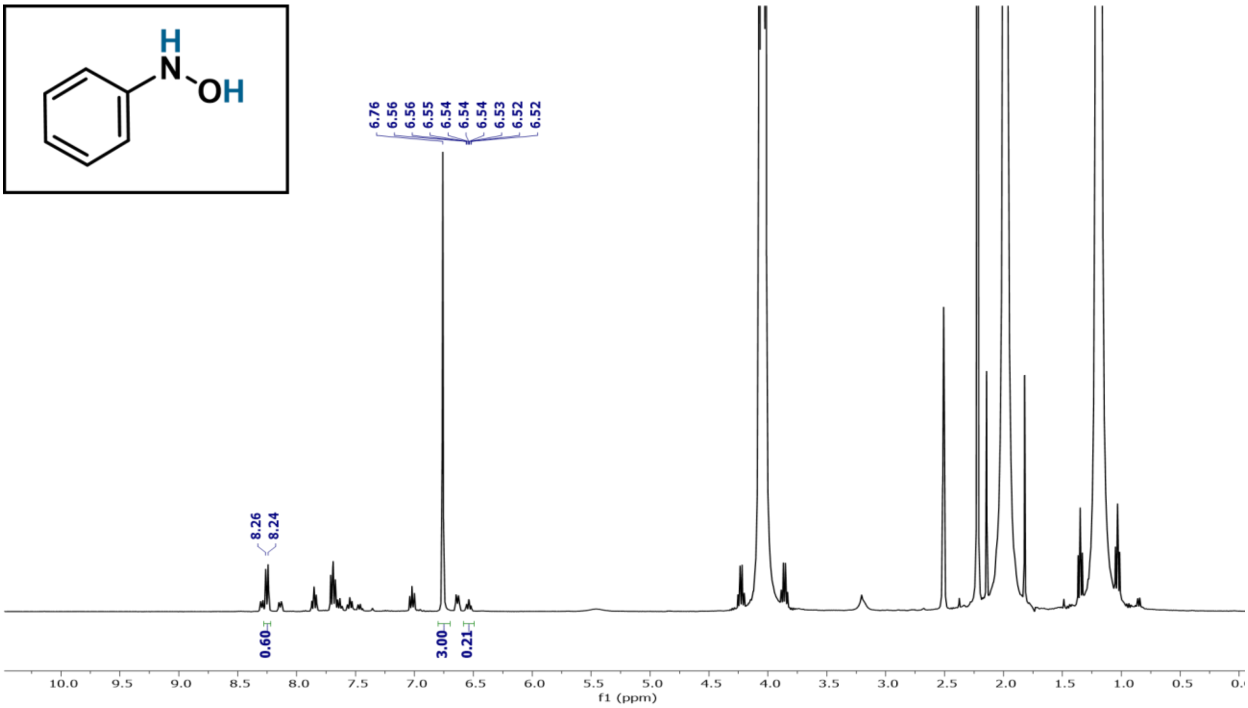

Figure S107 – ^{1}H NMR (400 MHz, DMSO-d_6, 296 K) crude spectrum of compound **3b**.

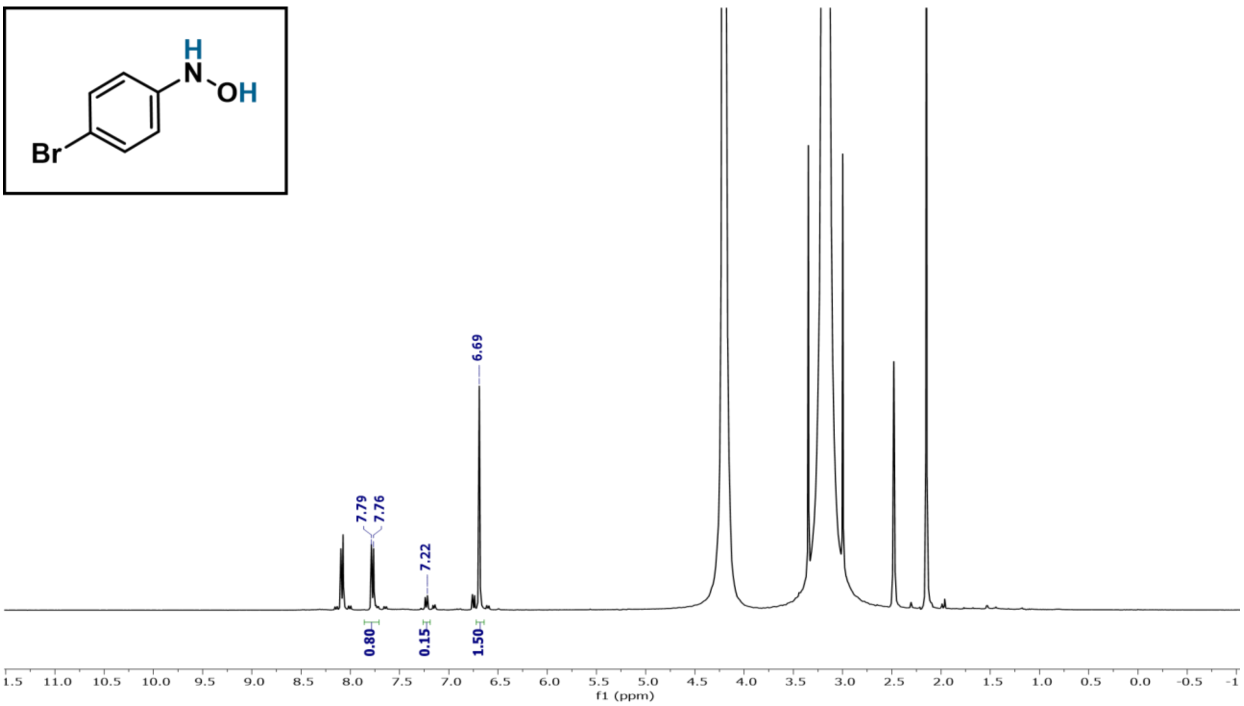

Figure S108 – ^{1}H NMR (400 MHz, DMSO-d_6, 296 K) crude spectrum of compound **3c**.

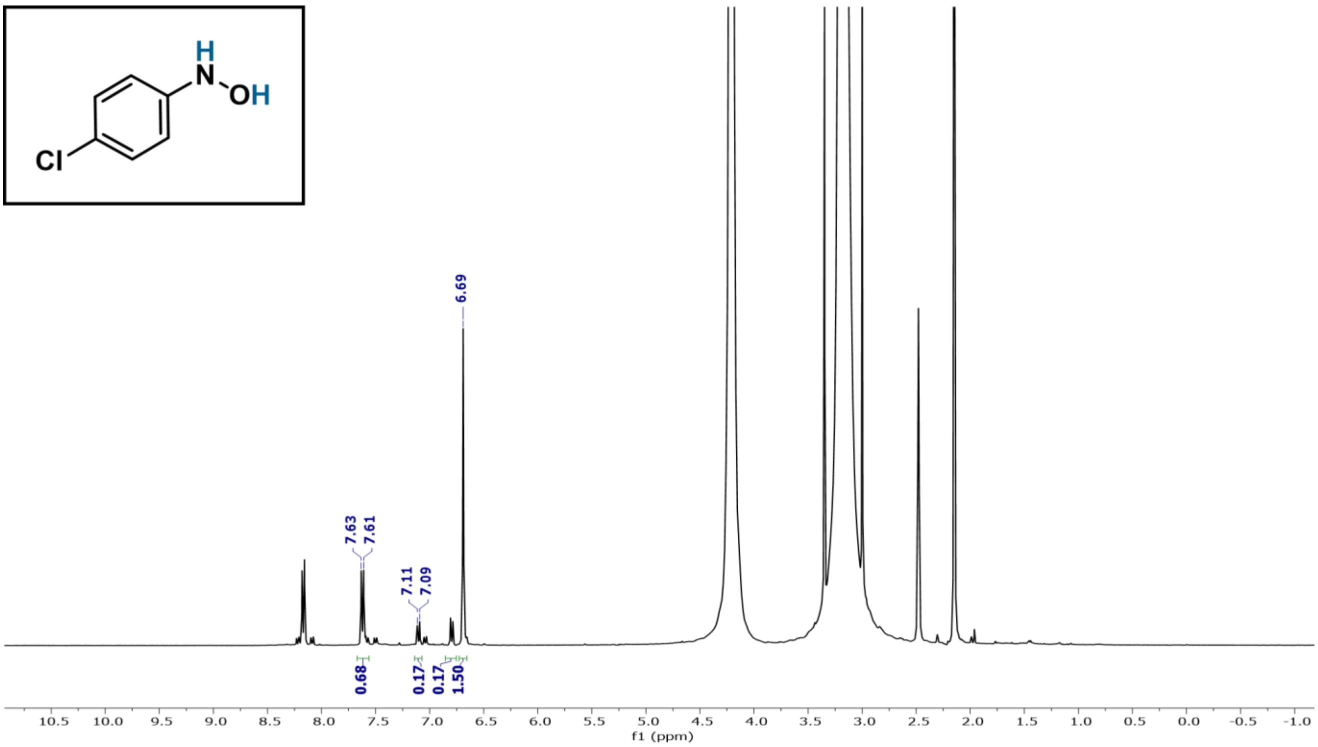

Figure S109 – ^{1}H NMR (400 MHz, DMSO-d_6, 296 K) crude spectrum of compound **3d**.

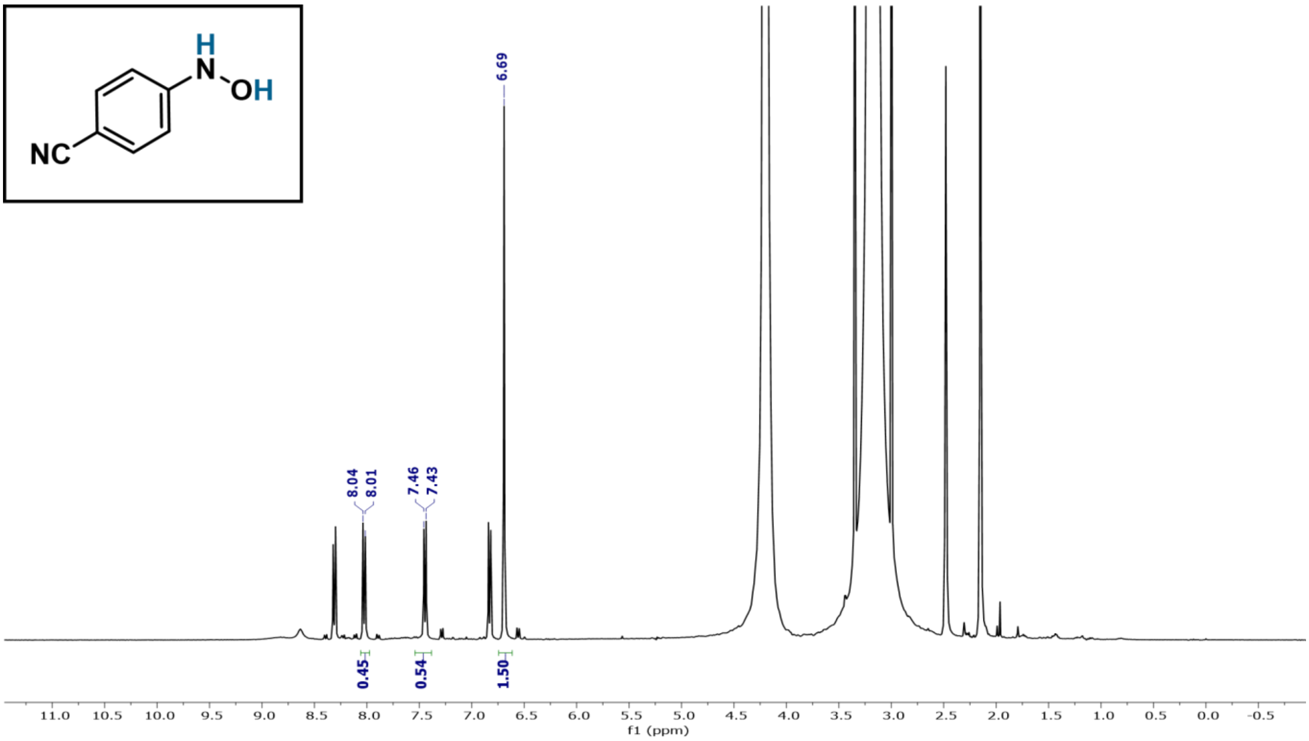

Figure S110 – ^{1}H NMR (400 MHz, DMSO-d_6, 296 K) crude spectrum of compound **3e**.

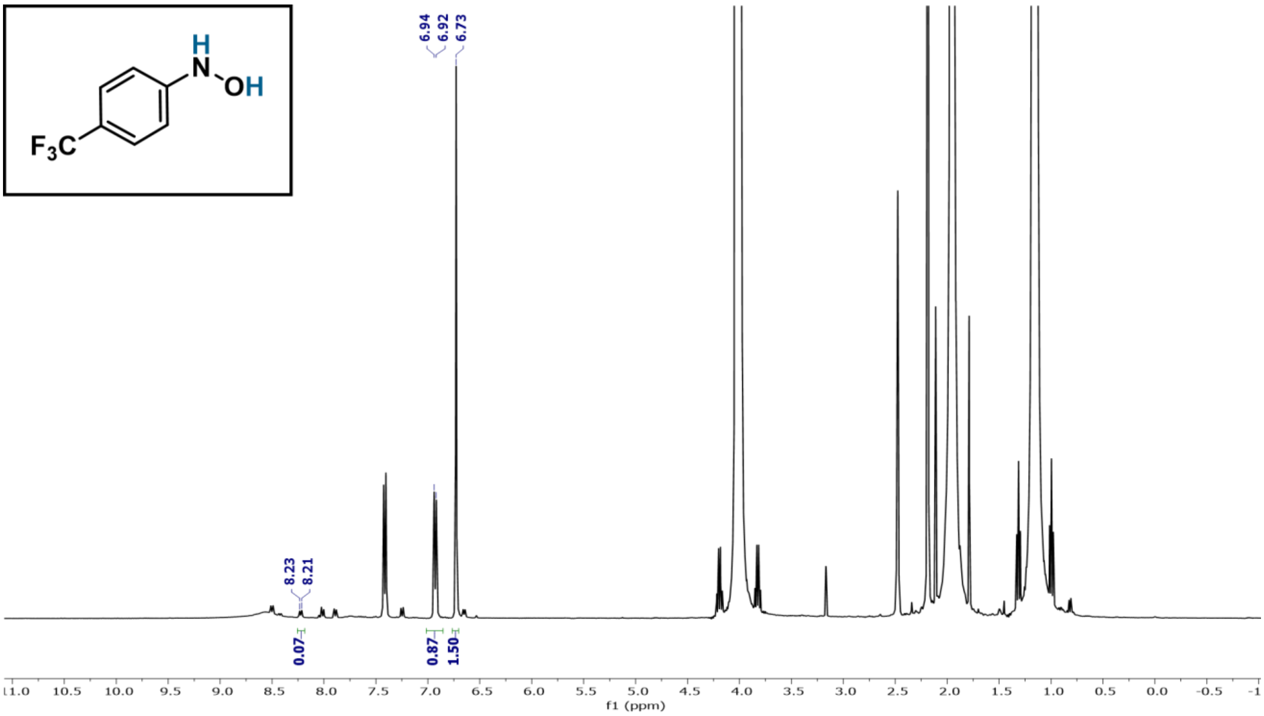

Figure S111 – ^{1}H NMR (400 MHz, DMSO-d_6, 296 K) crude spectrum of compound **3f**.

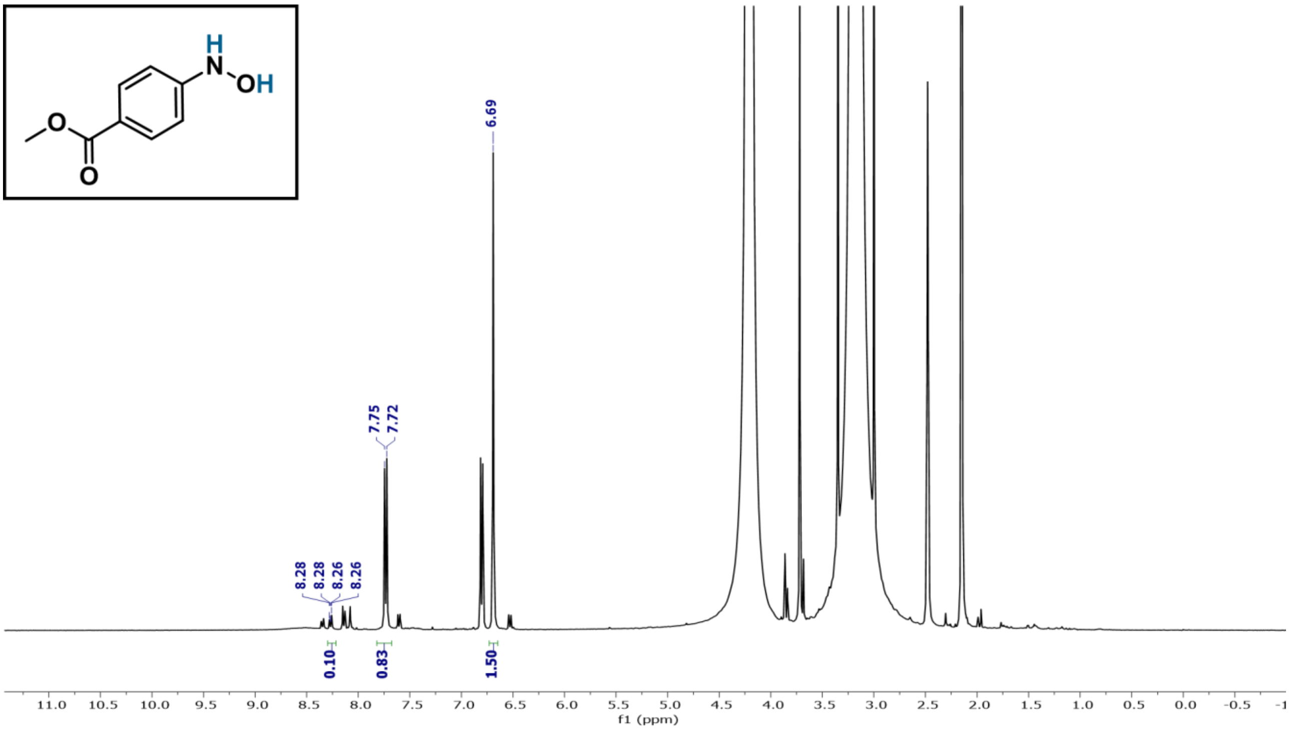

Figure S112 – ^{1}H NMR (400 MHz, DMSO-d_6, 296 K) crude spectrum of compound **3g**.

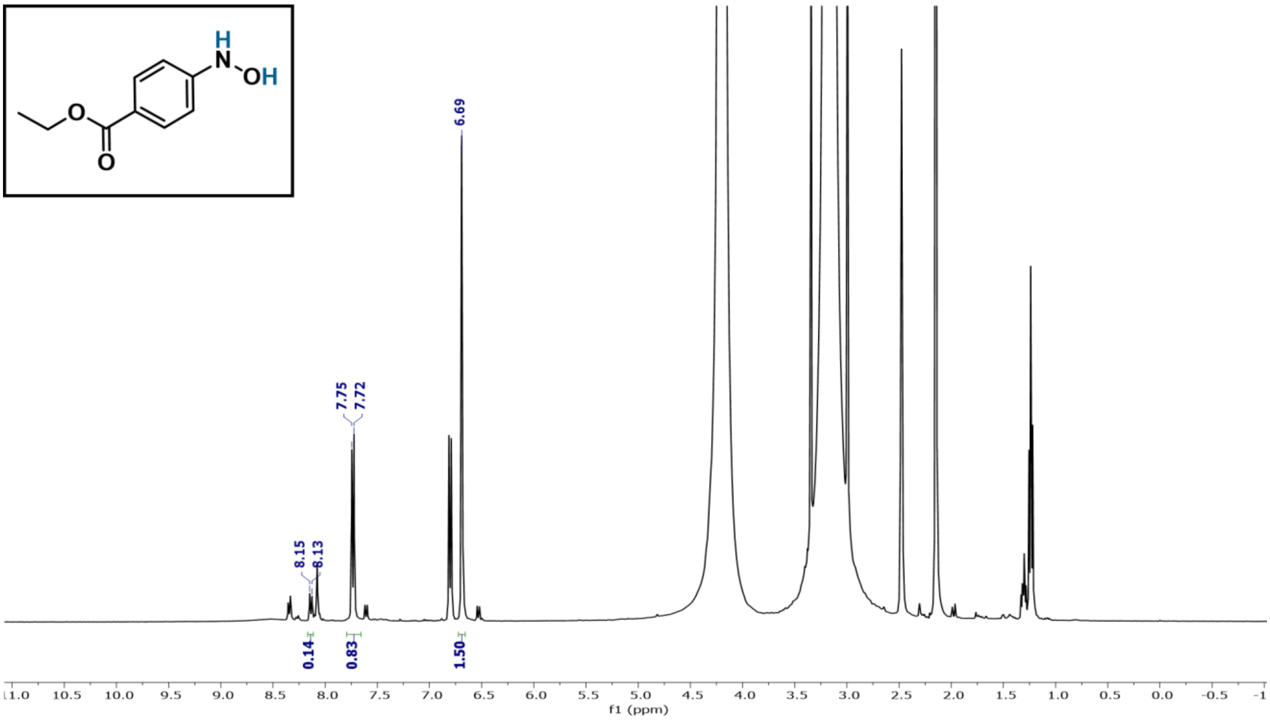

Figure S113 – ^{1}H NMR (400 MHz, DMSO-d_6, 296 K) crude spectrum of compound **3h**.

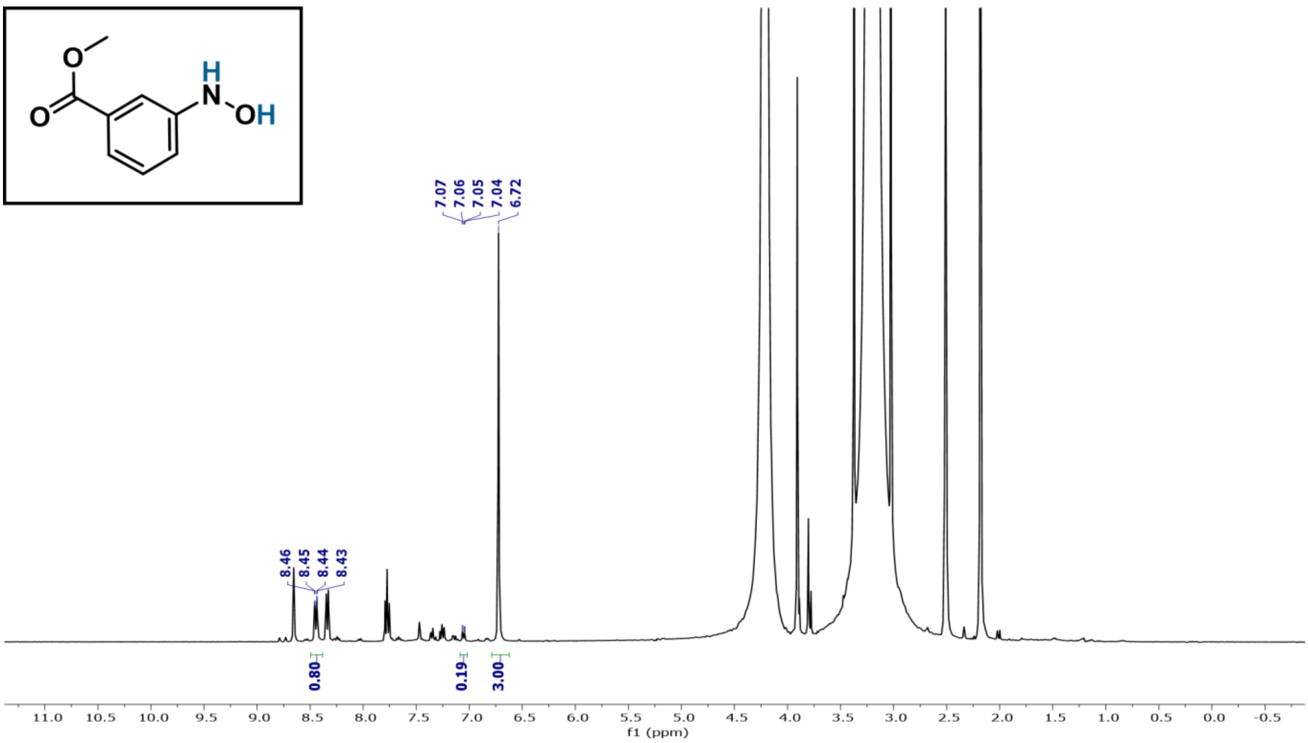

Figure S114 – ^{1}H NMR (400 MHz, DMSO-d_6, 296 K) crude spectrum of compound **3i**.

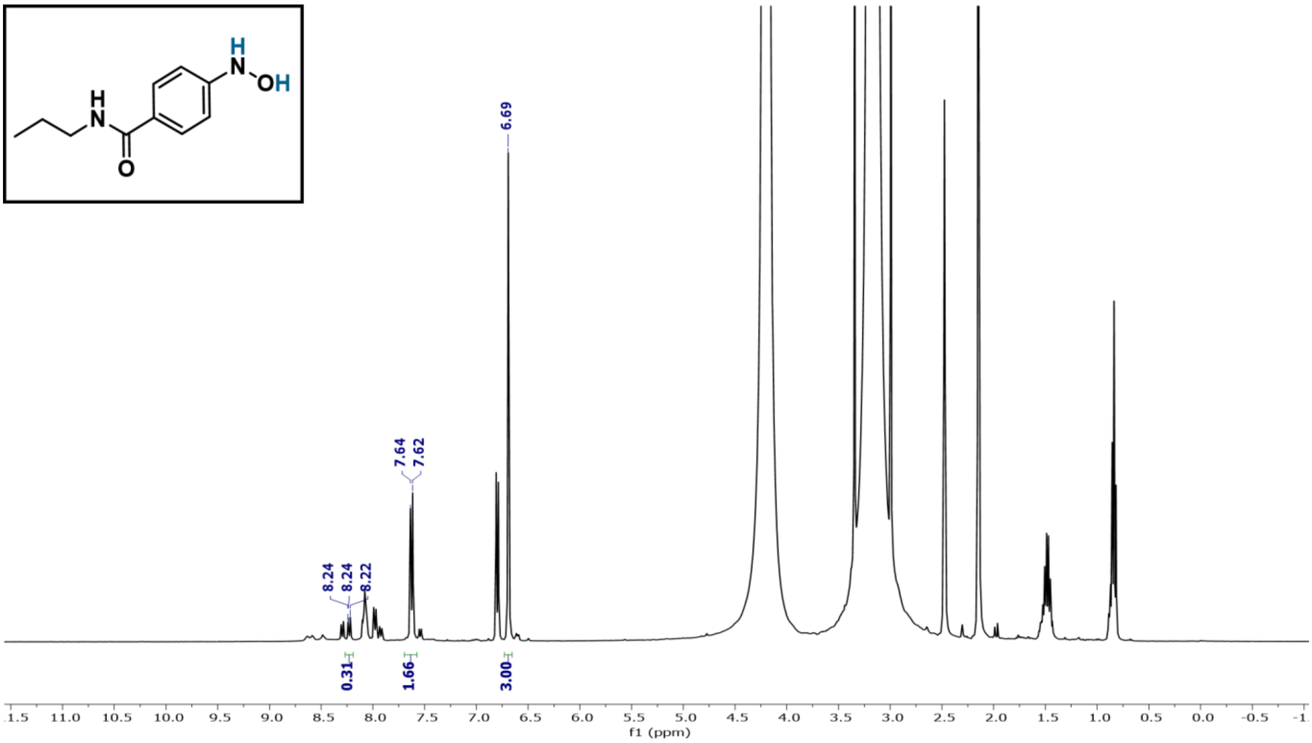

Figure S115 – ^{1}H NMR (400 MHz, DMSO-d_6, 296 K) crude spectrum of compound **3j**.

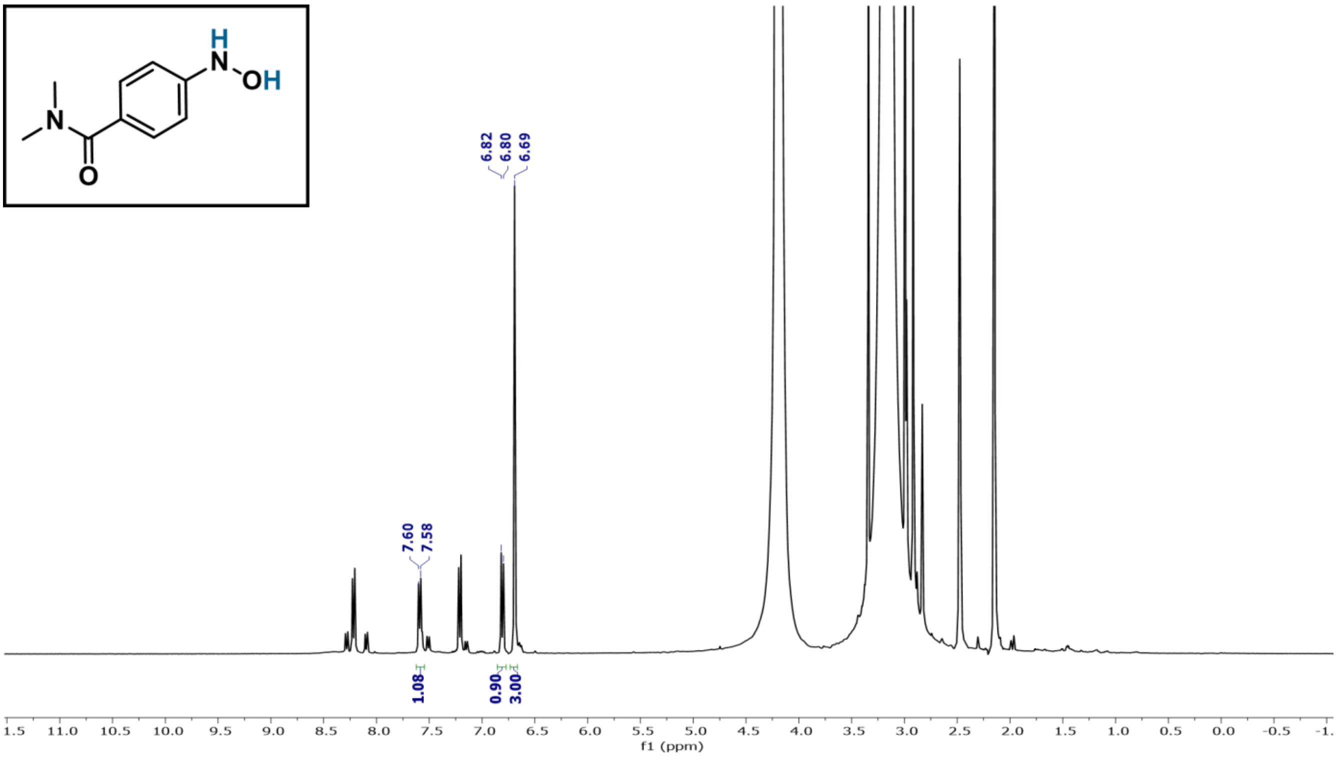

Figure S116 – ^{1}H NMR (400 MHz, DMSO-*d*$_6$, 296 K) crude spectrum of compound **3k**.

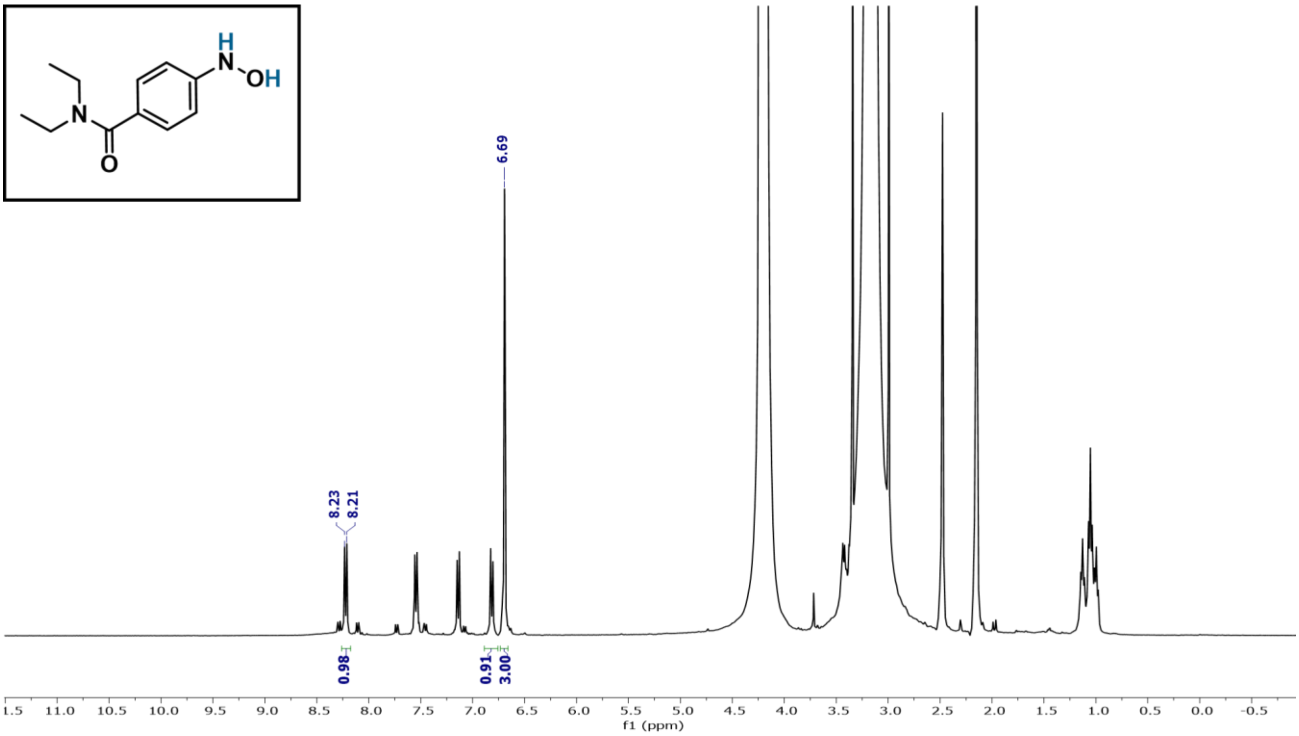

Figure S117 – ^{1}H NMR (400 MHz, DMSO-*d*$_6$, 296 K) crude spectrum of compound **3l**.

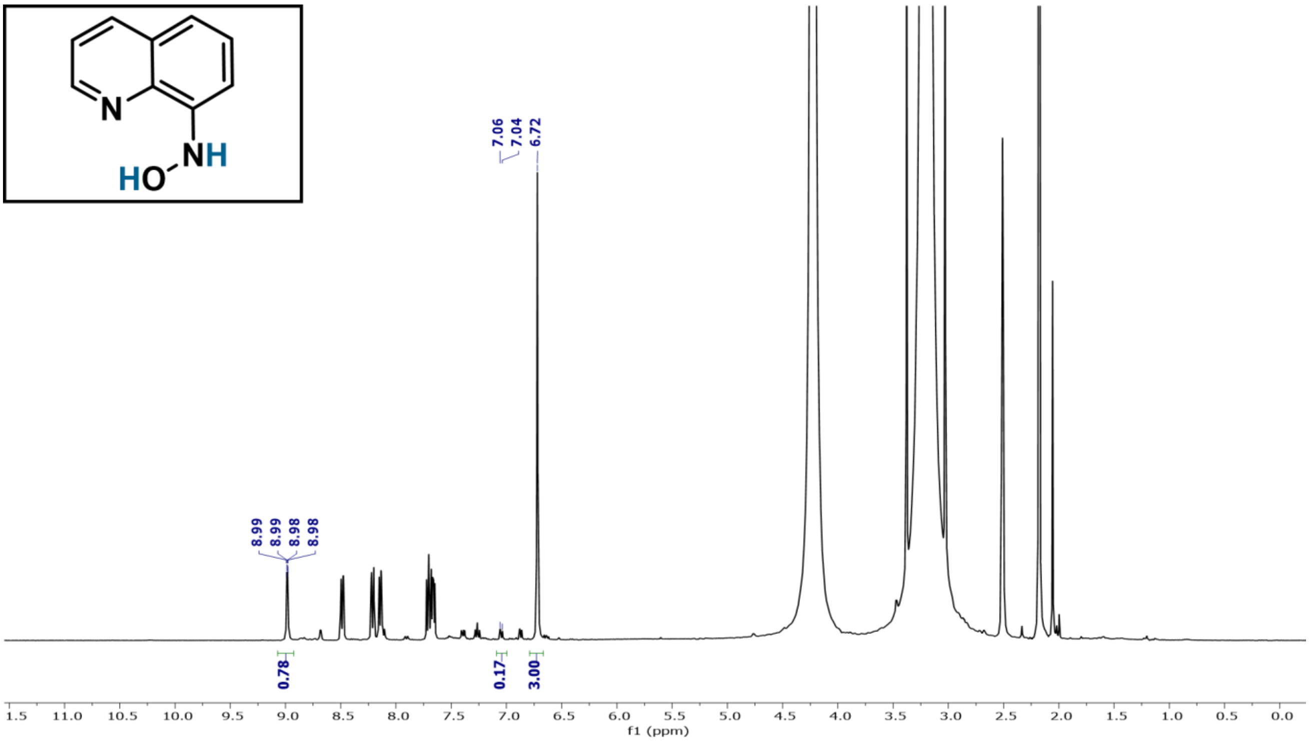

Figure S118 – ^{1}H NMR (400 MHz, DMSO-d_6, 296 K) crude spectrum of compound **3n**.

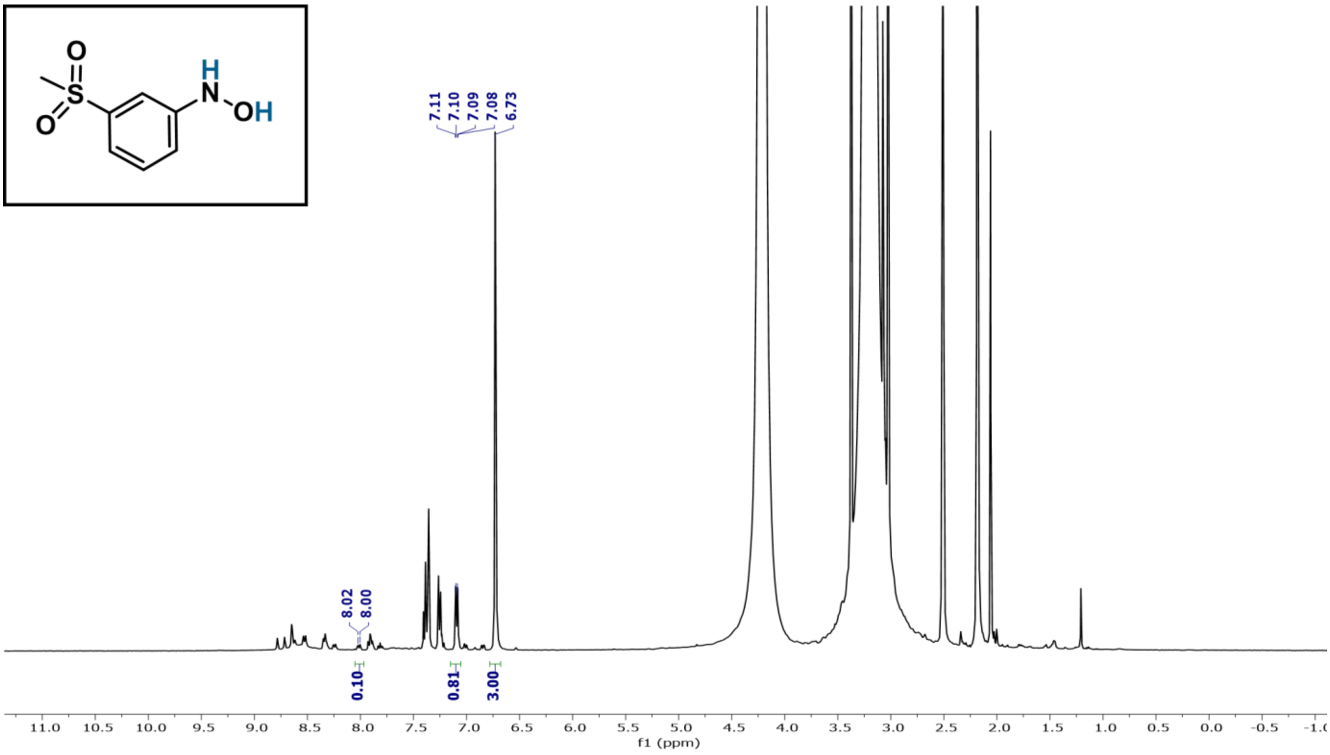

Figure S119 – ^{1}H NMR (400 MHz, DMSO-d_6, 296 K) crude spectrum of compound **3o**.

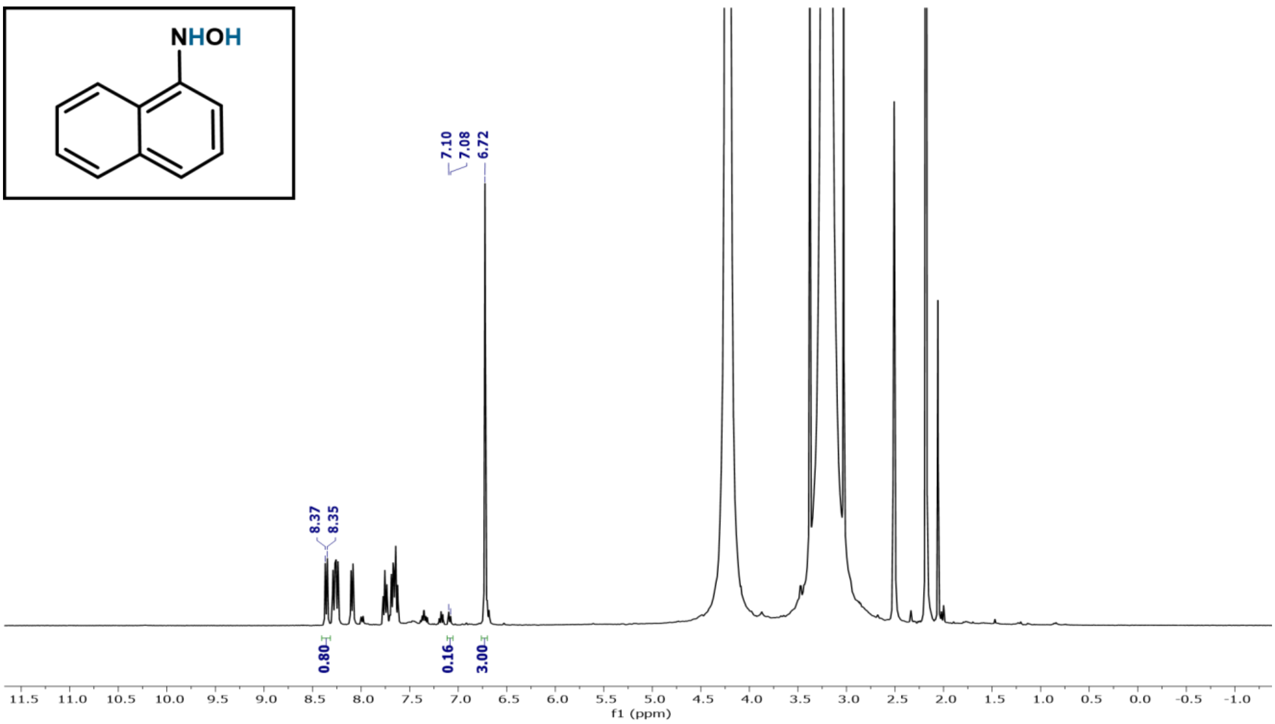

Figure S120 – ^{1}H NMR (400 MHz, DMSO-d_6, 296 K) crude spectrum of compound **3p**.

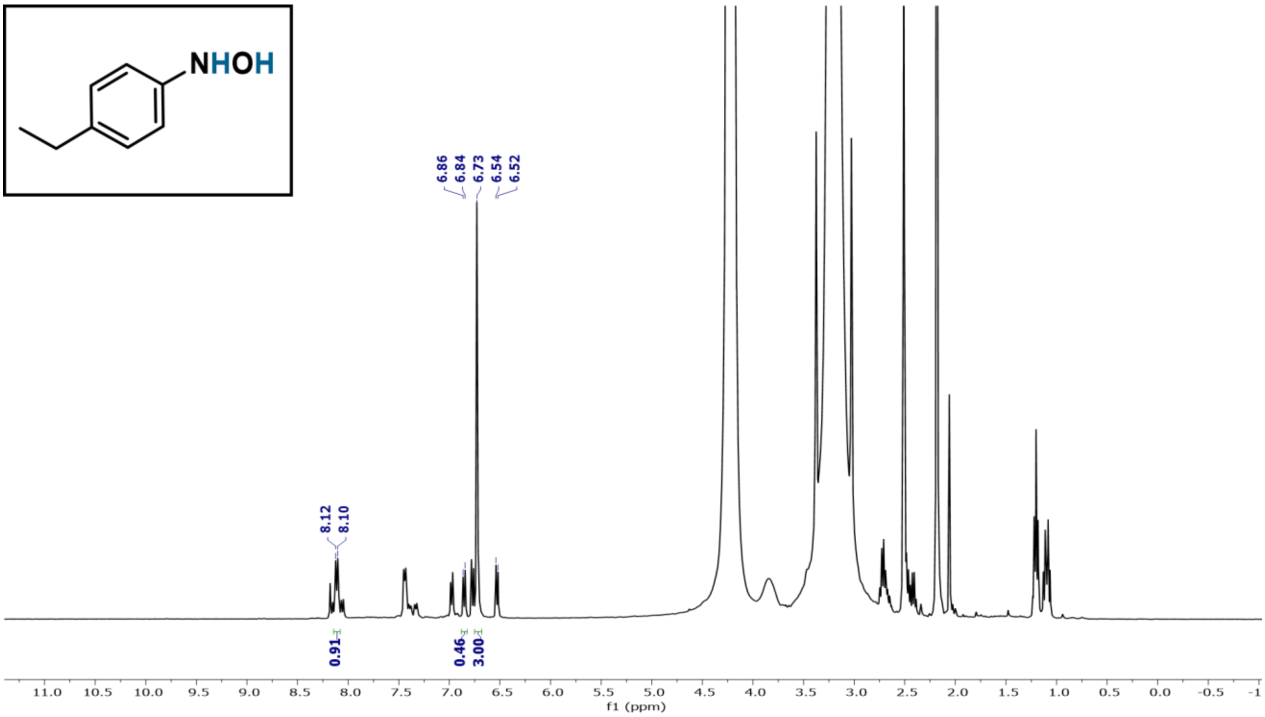

Figure S121 – ^{1}H NMR (400 MHz, DMSO-d_6, 296 K) crude spectrum of compound **3q**.

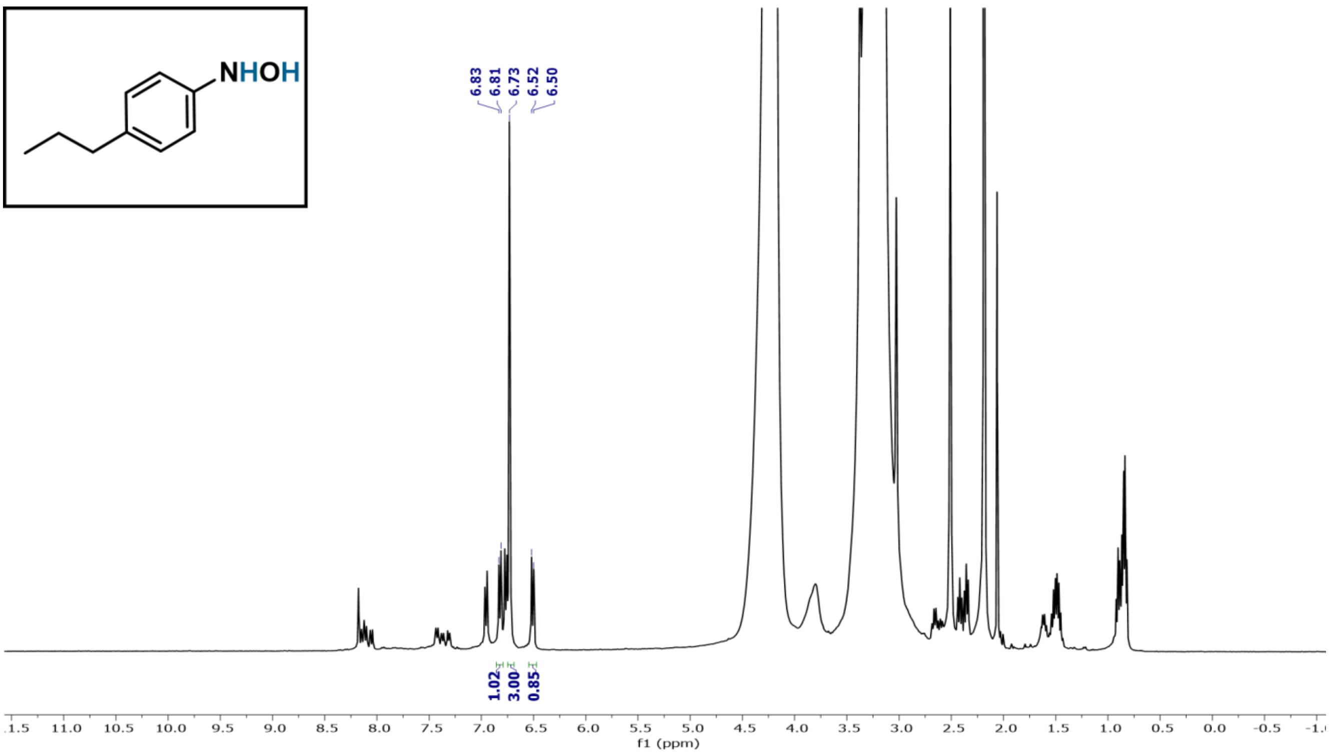

Figure S122 – ^{1}H NMR (400 MHz, DMSO-d_6, 296 K) crude spectrum of compound **3r**.

^{1}H and ^{13}C Spectra

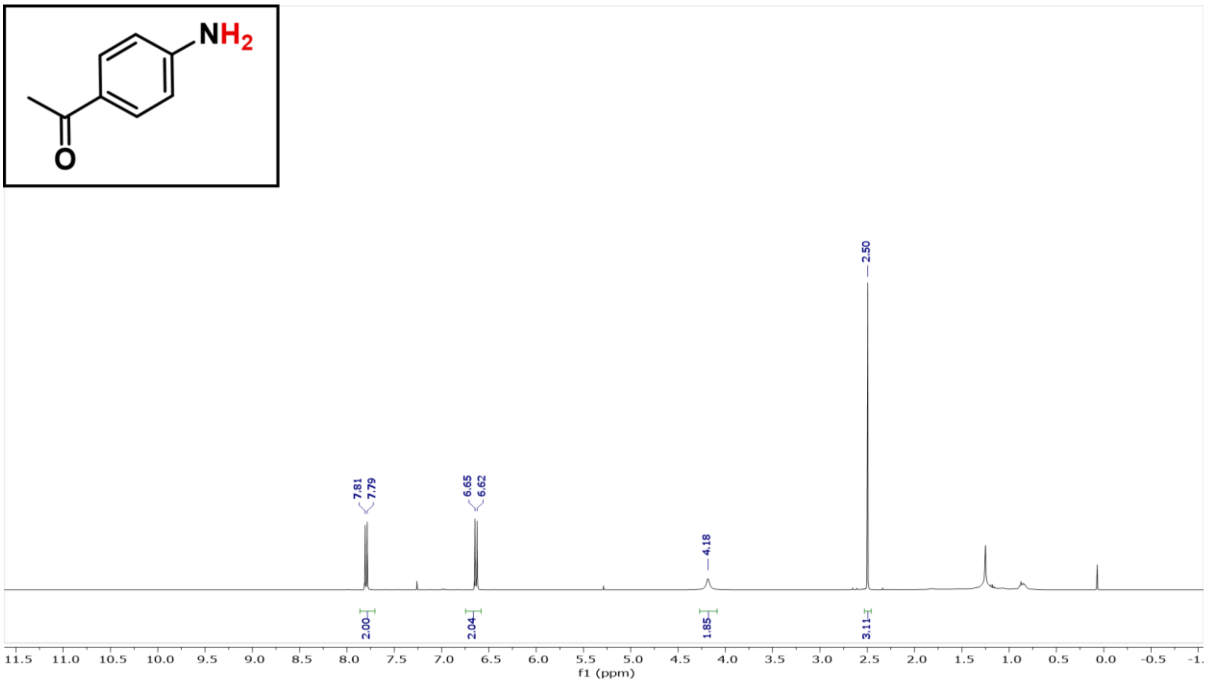

Figure S123 – ^{1}H NMR (400 MHz, $CDCl_3$, 296 K) spectrum of compound **2a**.

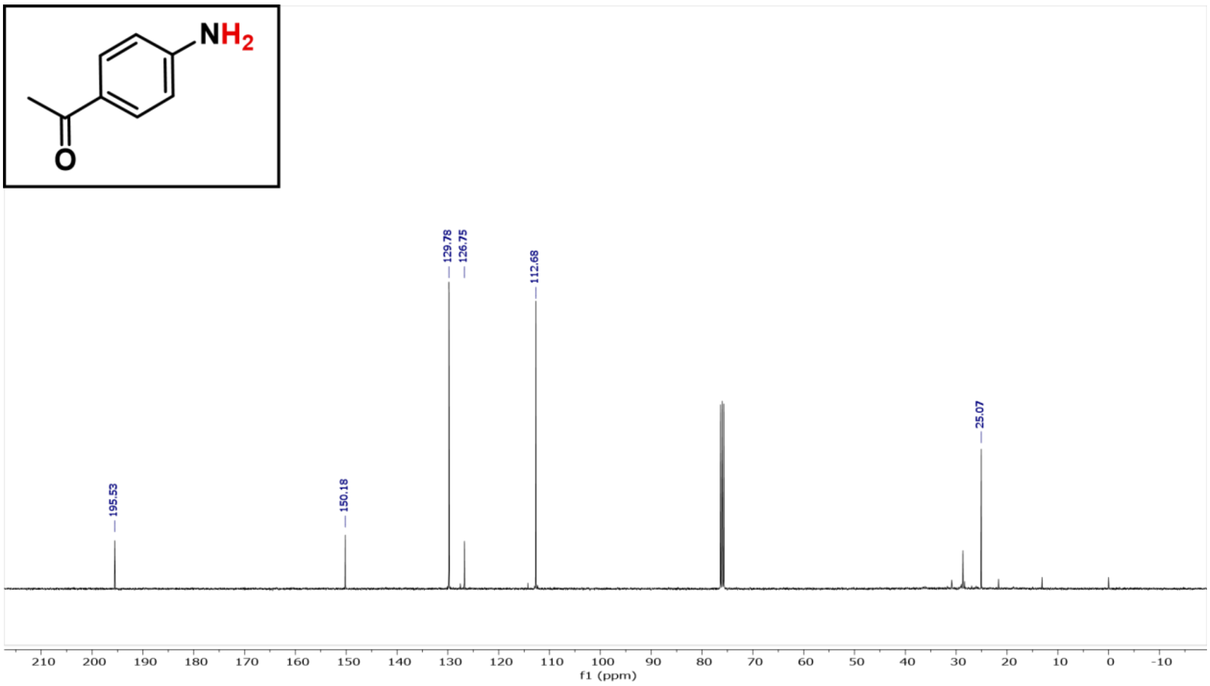

Figure S124 – ^{13}C{^{1}H} NMR (101 MHz, $CDCl_3$, 296 K) spectrum of compound **2a**.

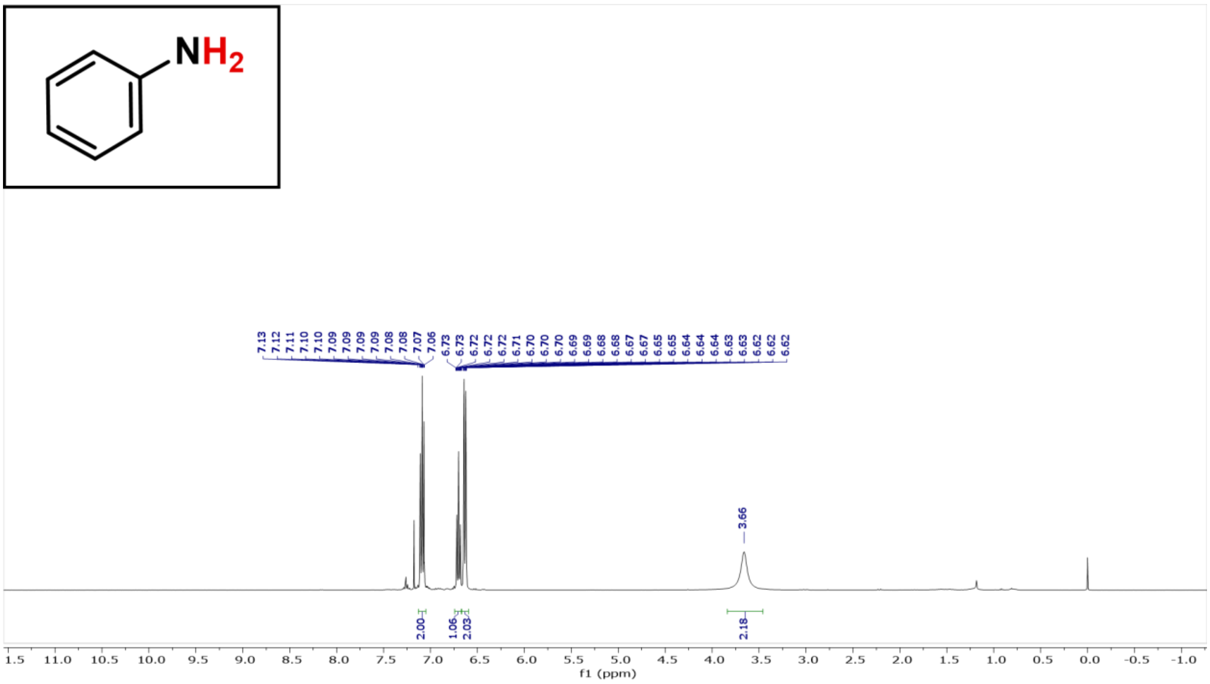

Figure S125 – ^{1}H NMR (400 MHz, CDCl$_3$, 296 K) spectrum of compound **2b**.

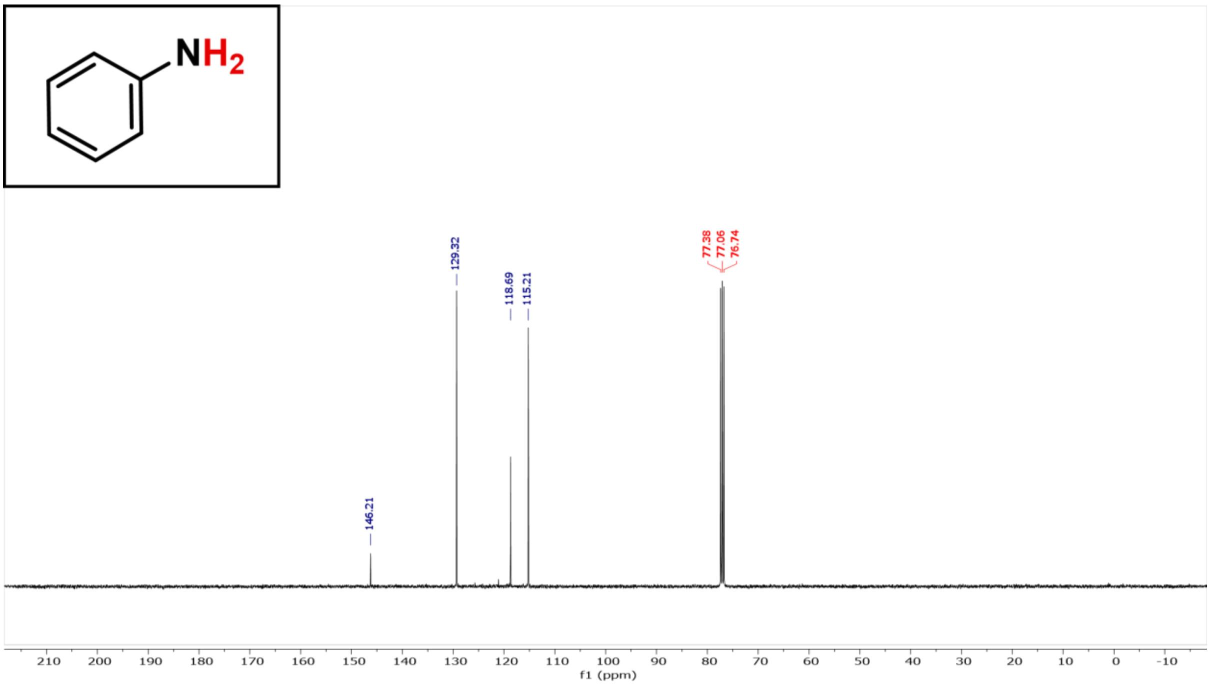

Figure S126 – ^{13}C{^{1}H} NMR (101 MHz, CDCl$_3$, 296 K) spectrum of compound **2b**.

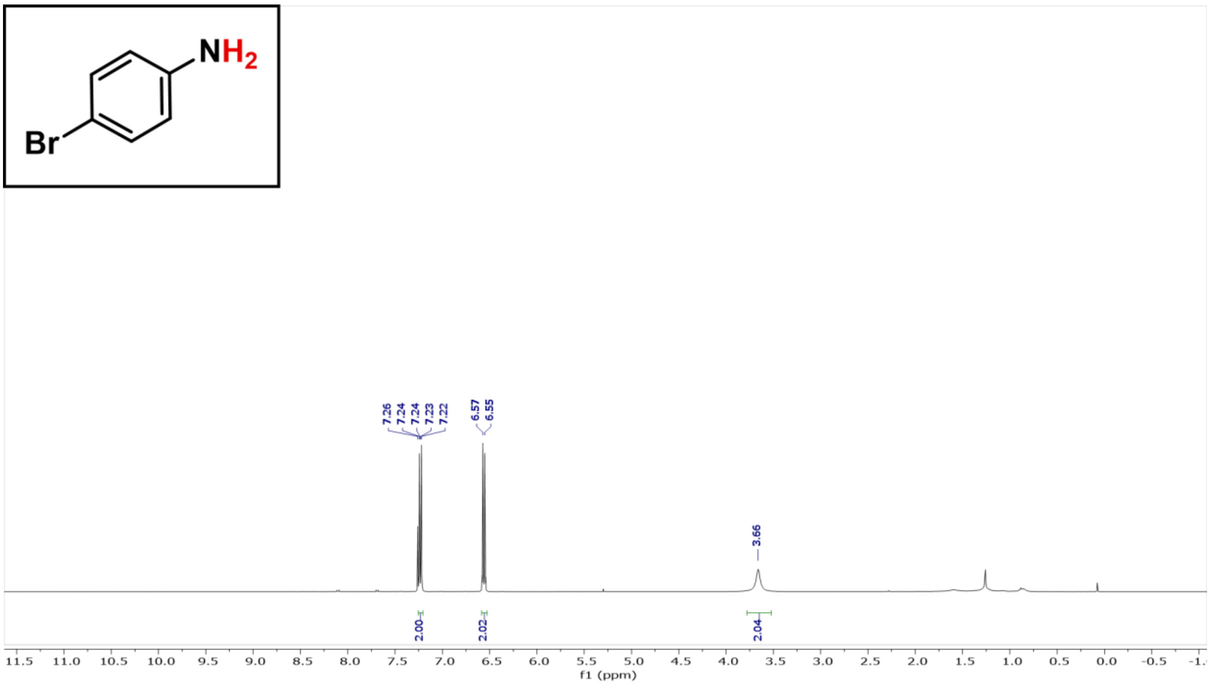

Figure S127 – ^{1}H NMR (400 MHz, CDCl$_3$, 296 K) spectrum of compound **2c**.

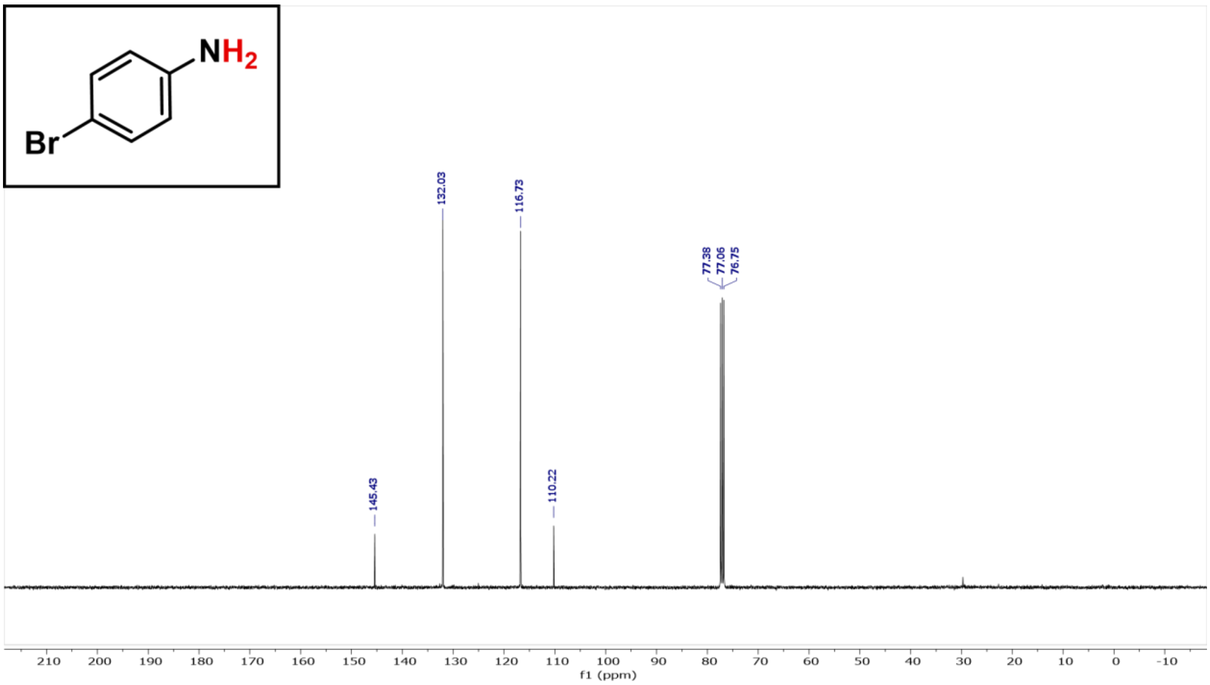

Figure S128 – ^{13}C{^{1}H} NMR (101 MHz, CDCl$_3$, 296 K) spectrum of compound **2c**.

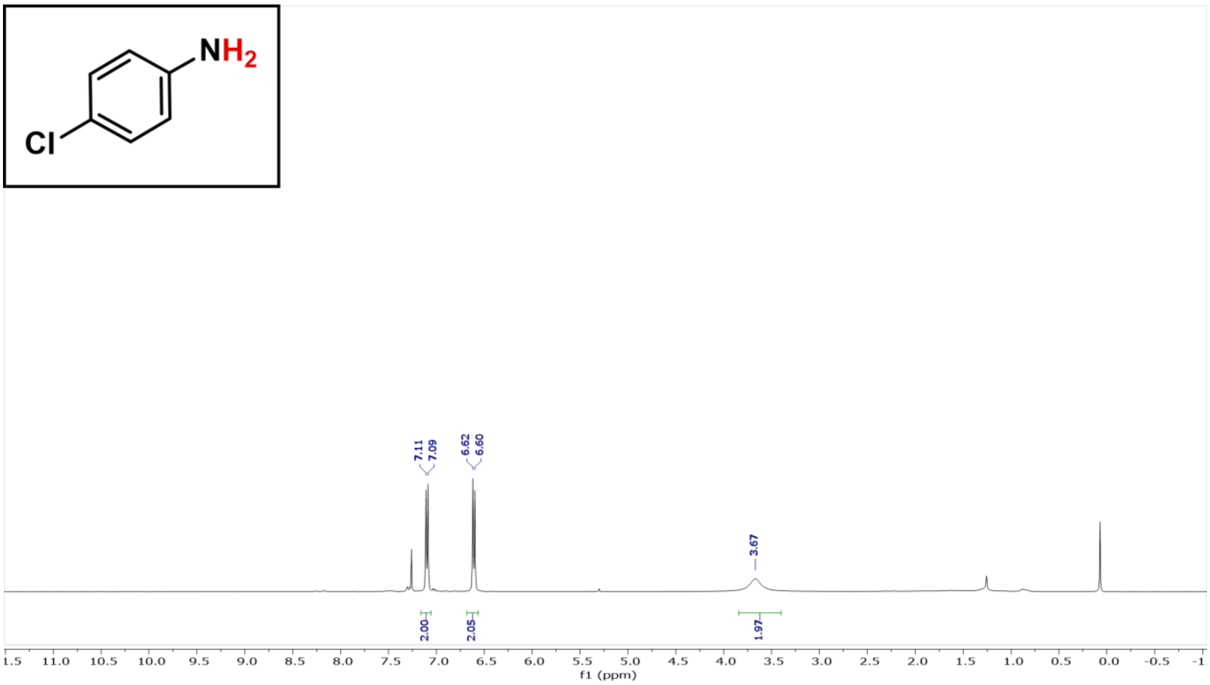

Figure S129 – ^{1}H NMR (400 MHz, $CDCl_3$, 296 K) spectrum of compound **2d**.

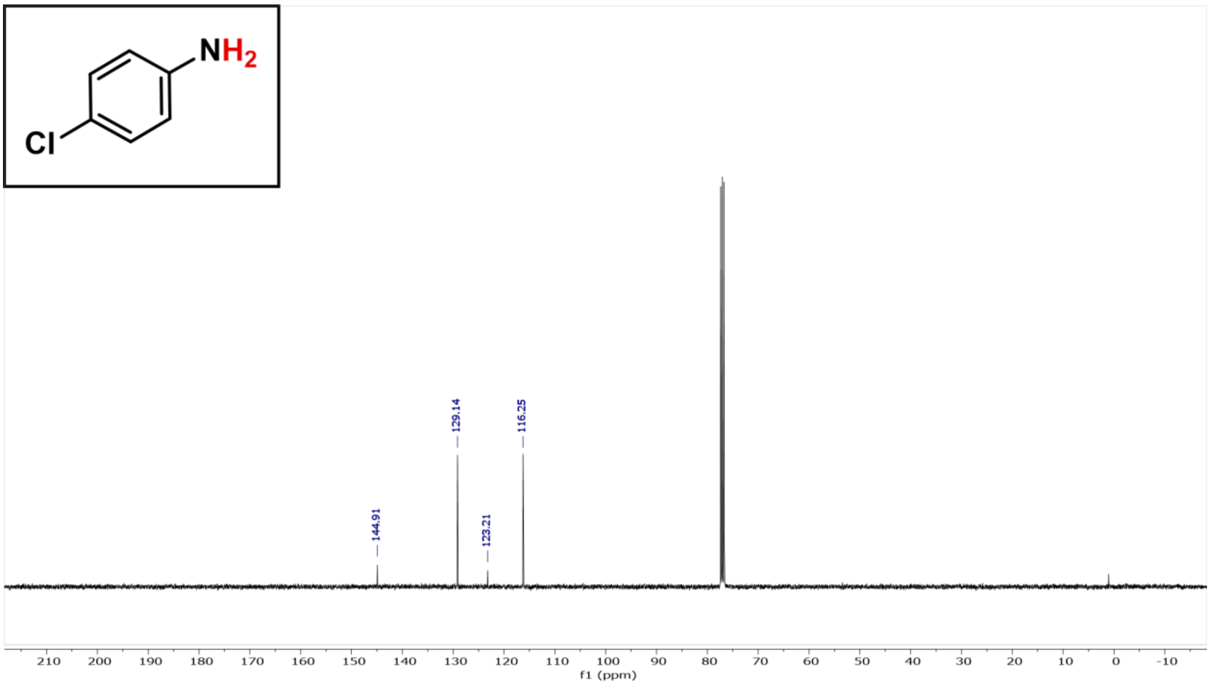

Figure S130 – $^{13}C\{^{1}H\}$ NMR (101 MHz, $CDCl_3$, 296 K) spectrum of compound **2d**.

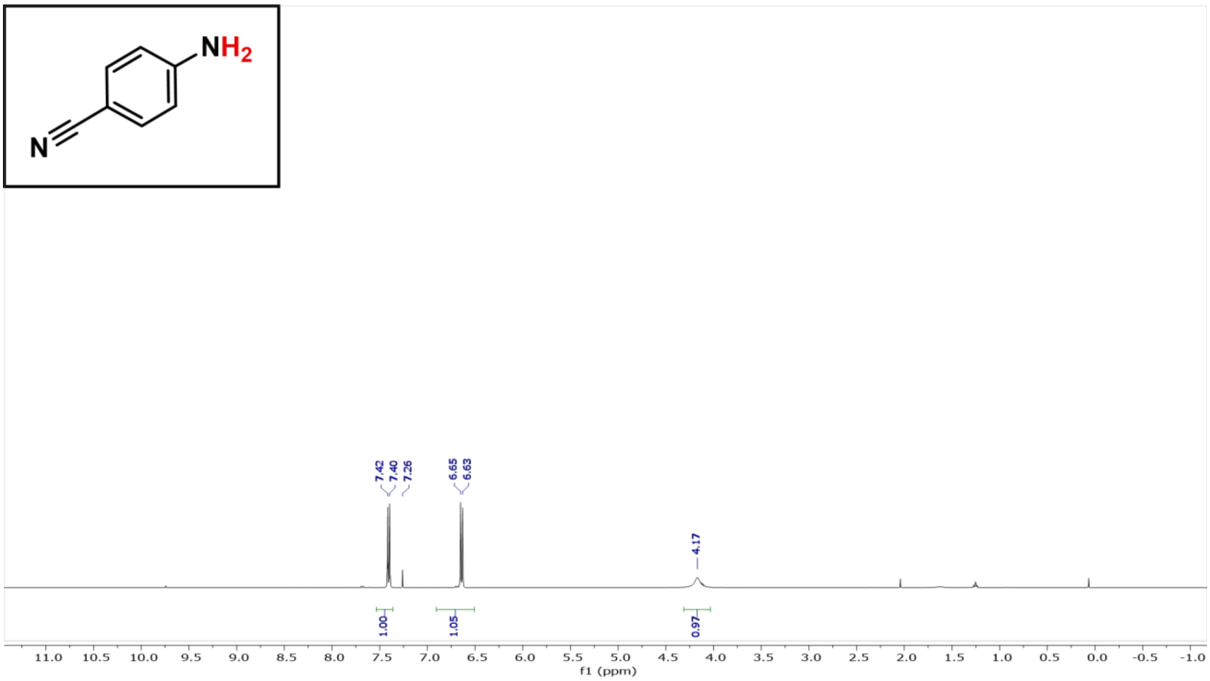

Figure S131 – ^{1}H NMR (400 MHz, $CDCl_3$, 296 K) spectrum of compound **2e**.

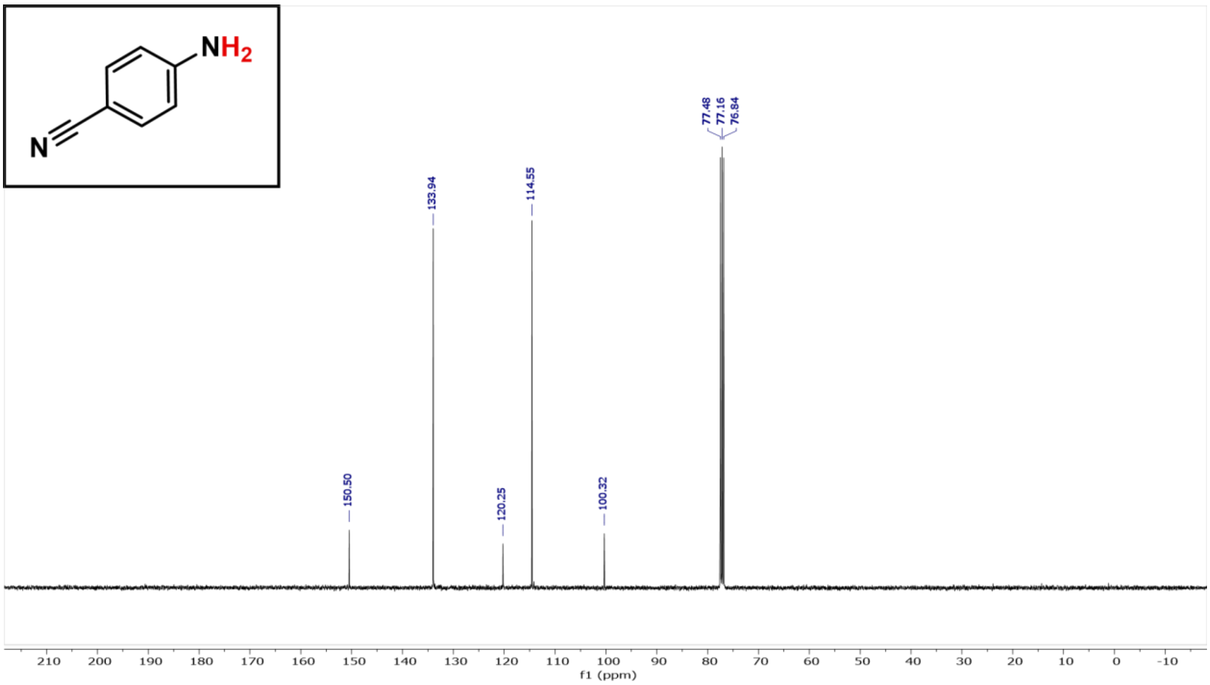

Figure S132 – ^{13}C{1H} NMR (101 MHz, CDCl3, 296 K) spectrum of compound **2e**.

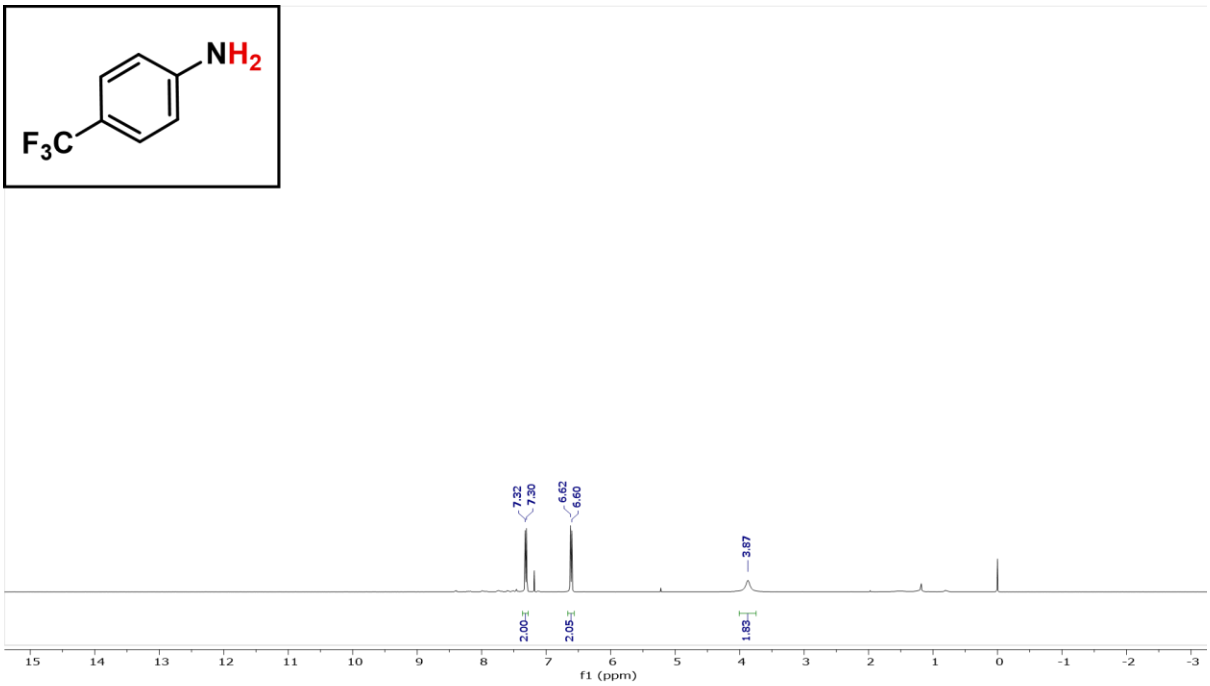

Figure S133 – ^{1}H NMR (400 MHz, CDCl$_3$, 296 K) spectrum of compound **2f**.

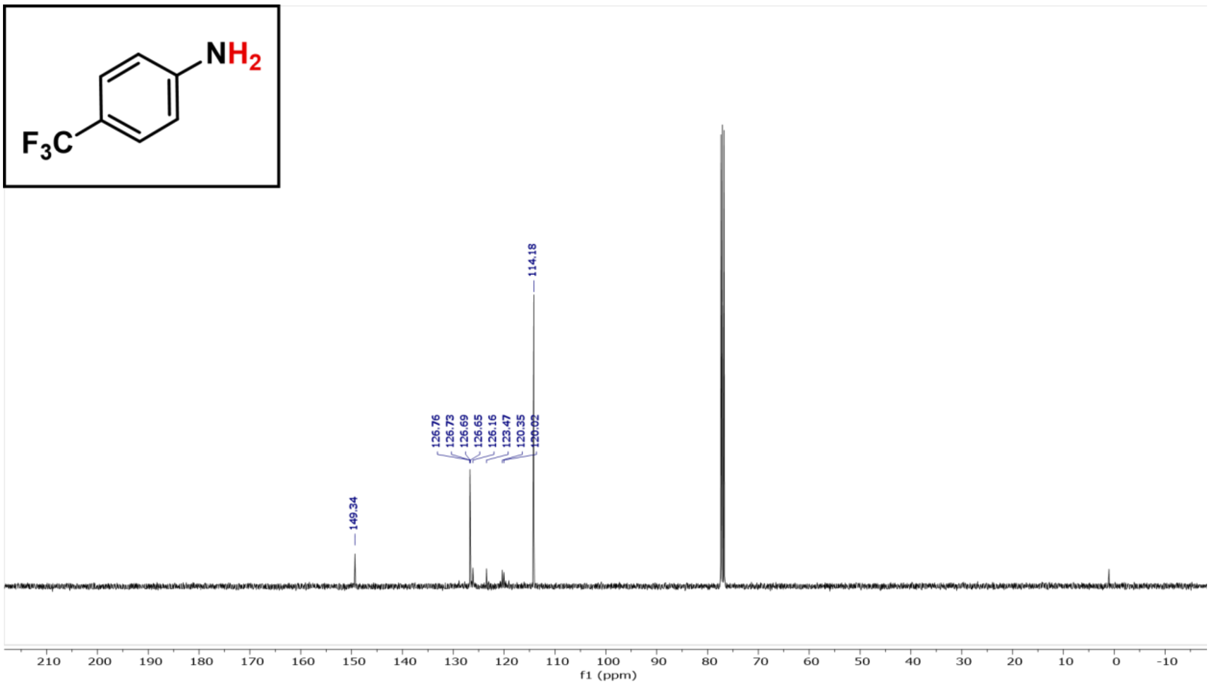

Figure S134 – ^{13}C{^{1}H} NMR (101 MHz, CDCl$_3$, 296 K) spectrum of compound **2f**.

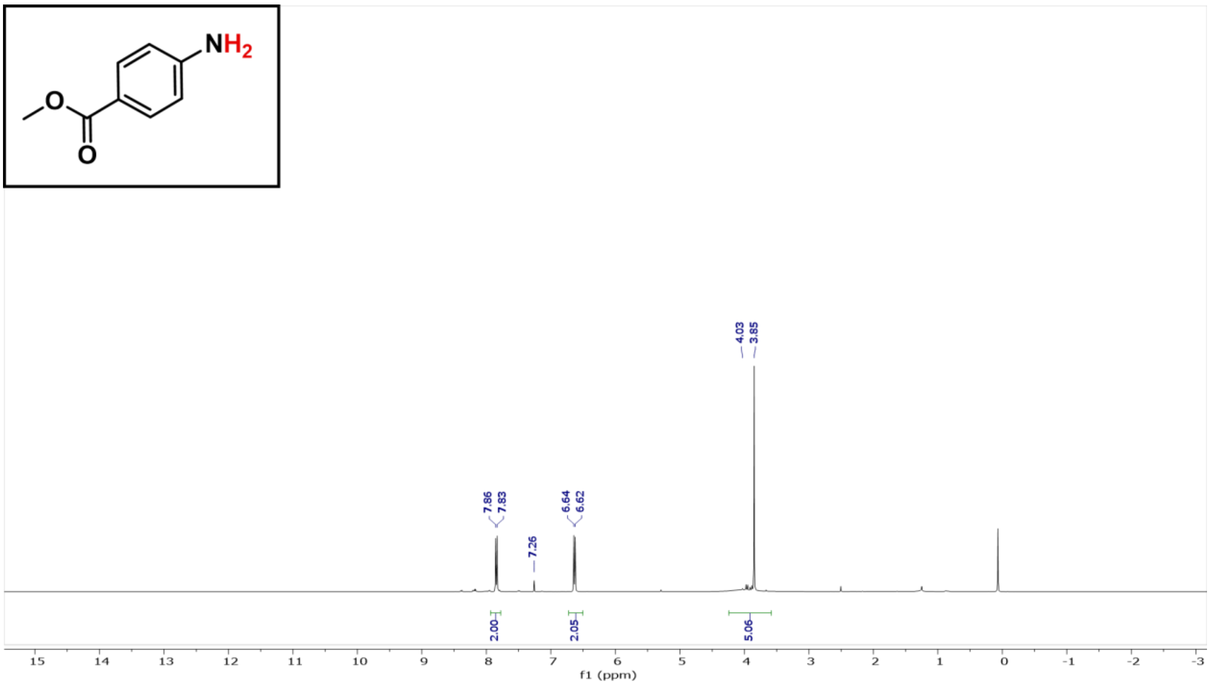

Figure S135 – ^{1}H NMR (400 MHz, CDCl$_3$, 296 K) spectrum of compound **2g**.

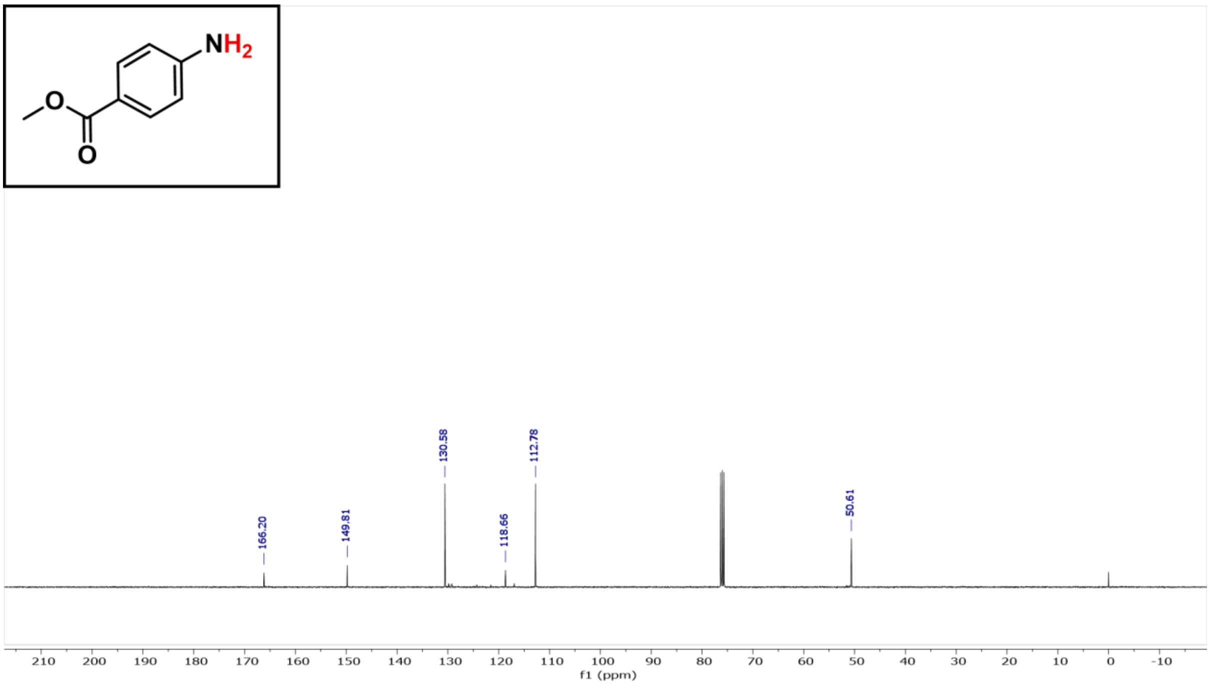

Figure S136 – ^{13}C{^{1}H} NMR (101 MHz, CDCl$_3$, 296 K) spectrum of compound **2g**.

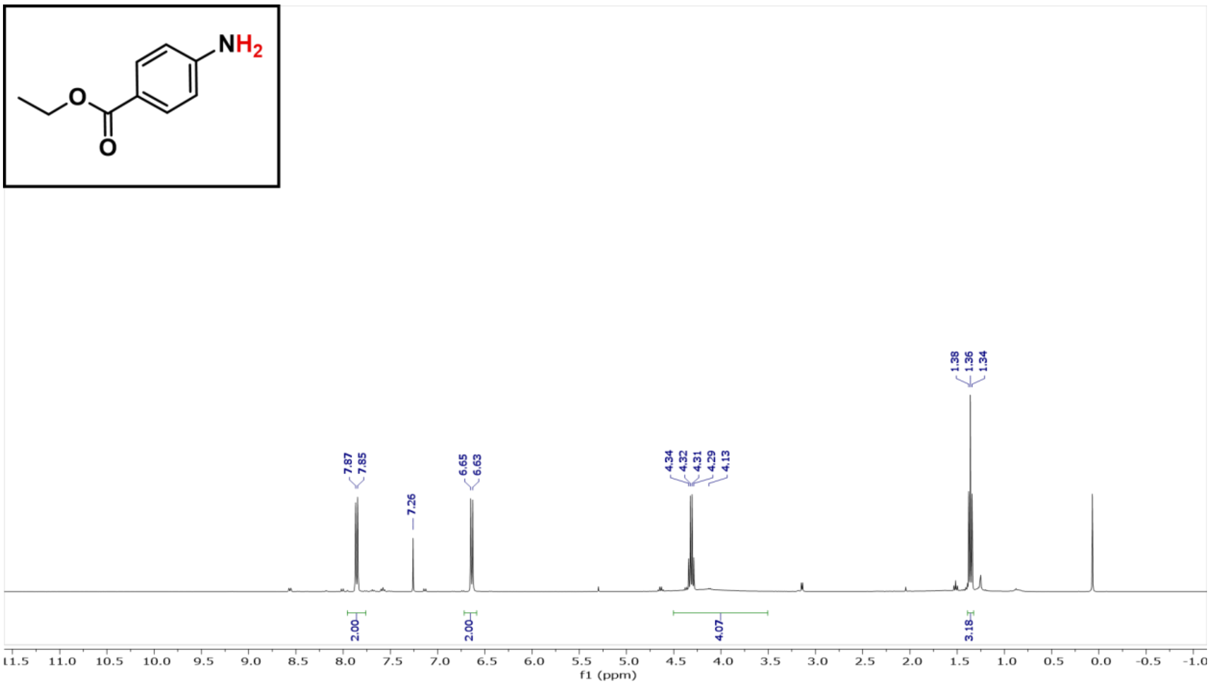

Figure S137 – ^{1}H NMR (400 MHz, CDCl₃, 296 K) spectrum of compound **2h**.

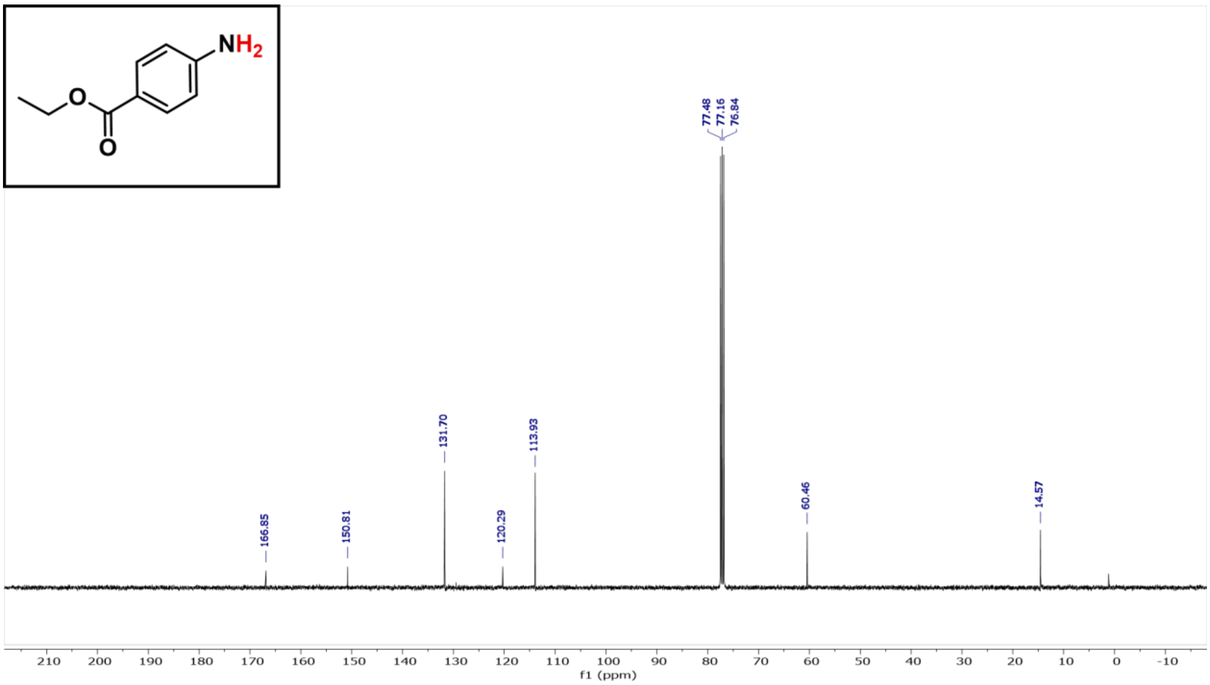

Figure S138 – ^{13}C{^{1}H} NMR (101 MHz, CDCl3, 296 K) spectrum of compound **2h**.

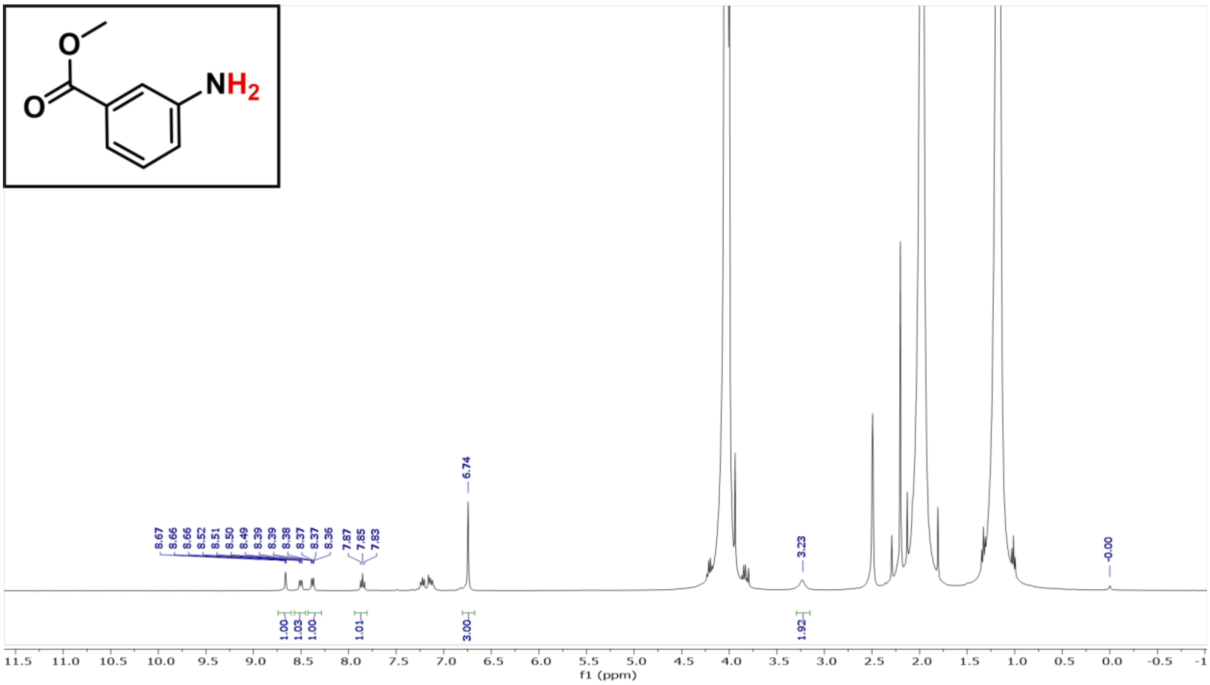

Figure S139 – ^{1}H NMR (400 MHz, $CDCl_3$, 296 K) crude spectrum of compound **2i**.

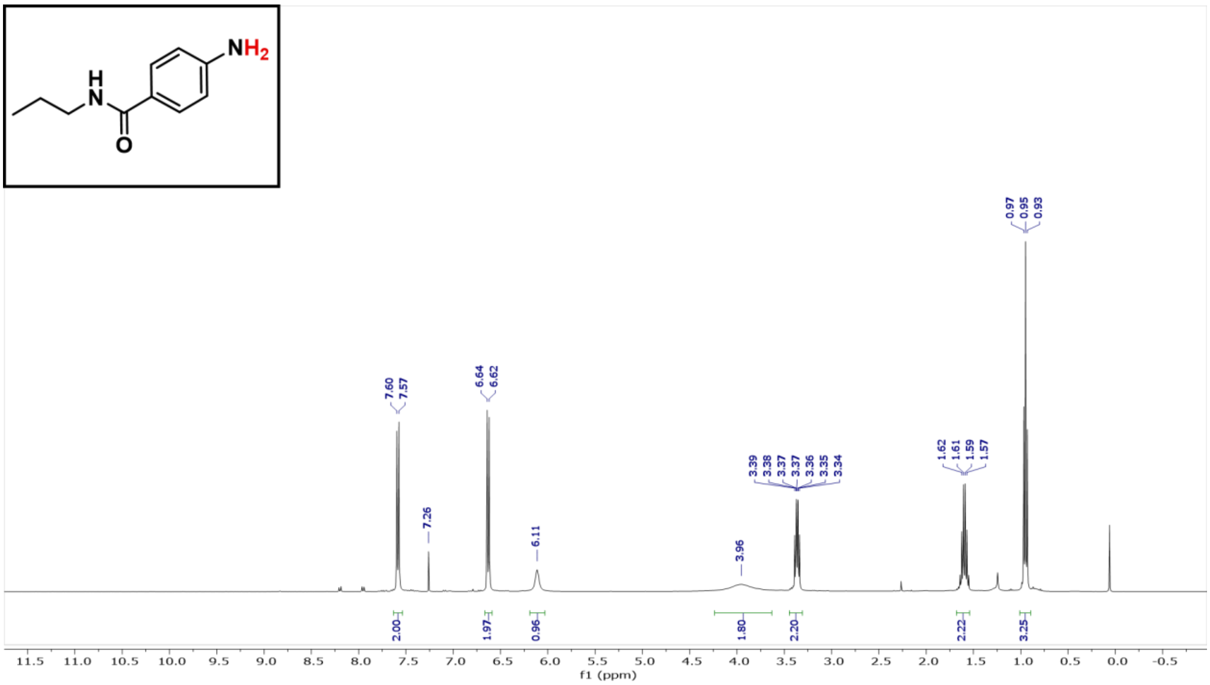

Figure S140 – ^{1}H NMR (400 MHz, $CDCl_3$, 296 K) spectrum of compound **2j**.

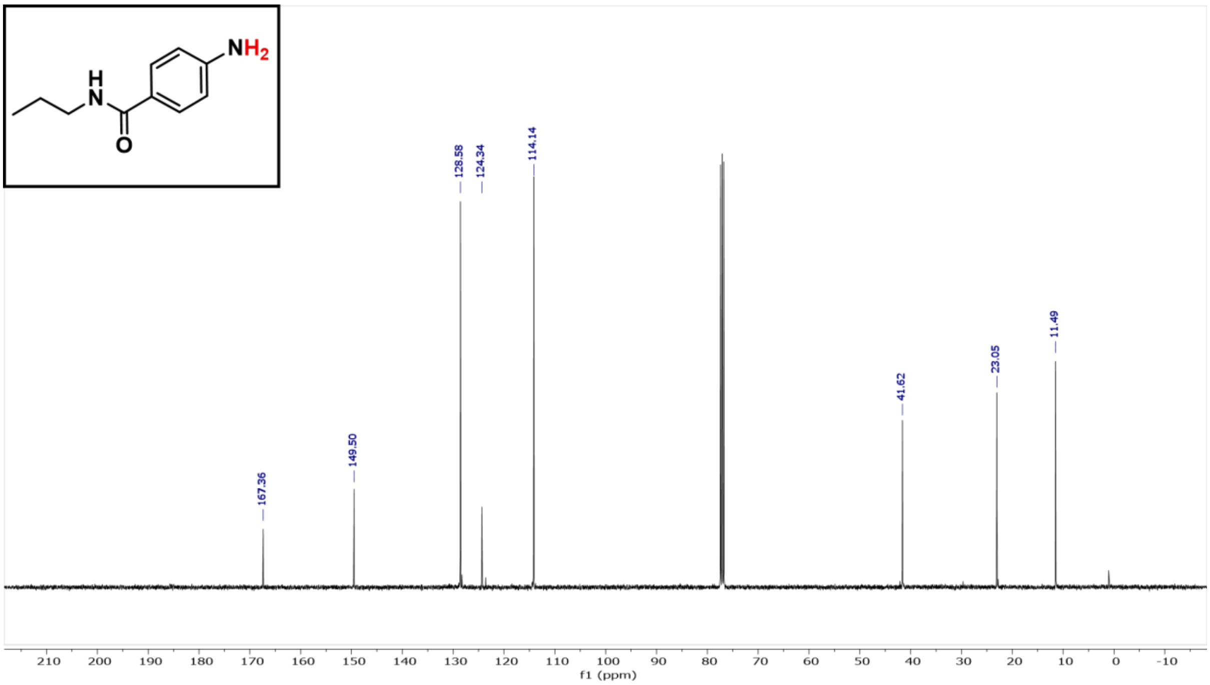

Figure S141 – $^{13}C\{^1H\}$ NMR (101 MHz, $CDCl_3$, 296 K) spectrum of compound **2j**.

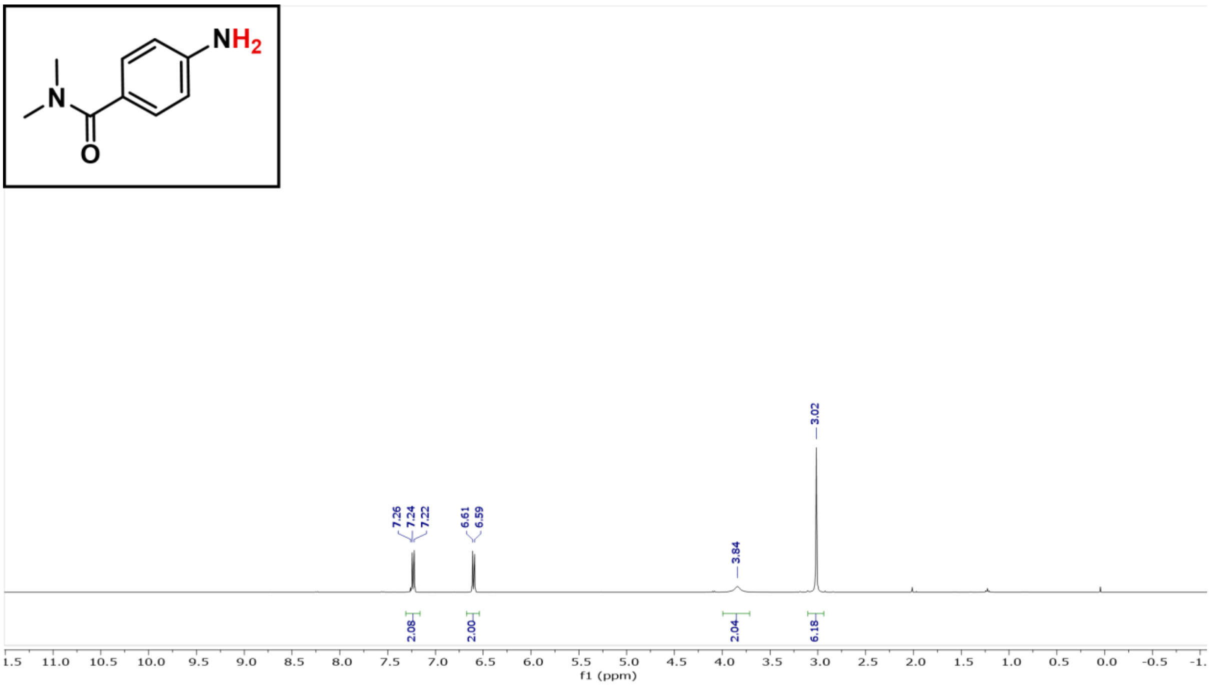

Figure S142 – ^1H NMR (400 MHz, $CDCl_3$, 296 K) spectrum of compound **2k**.

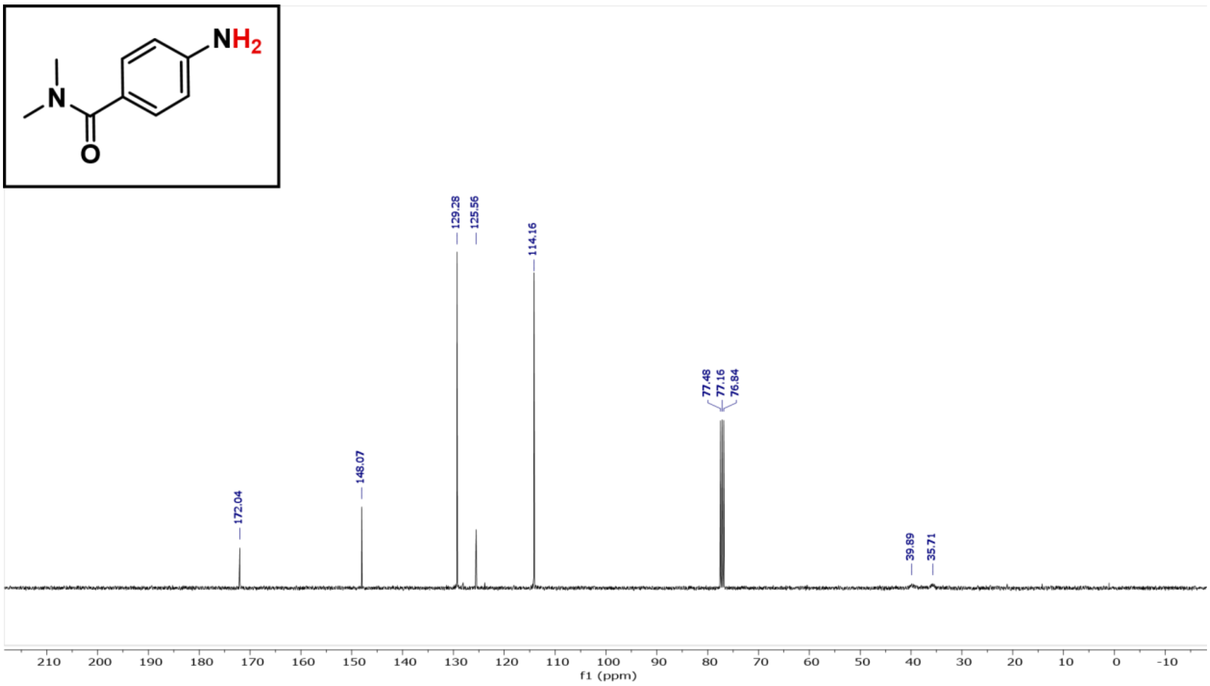

Figure S143 – ^{13}C{^{1}H} NMR (101 MHz, CDCl$_3$, 296 K) spectrum of compound **2k**.

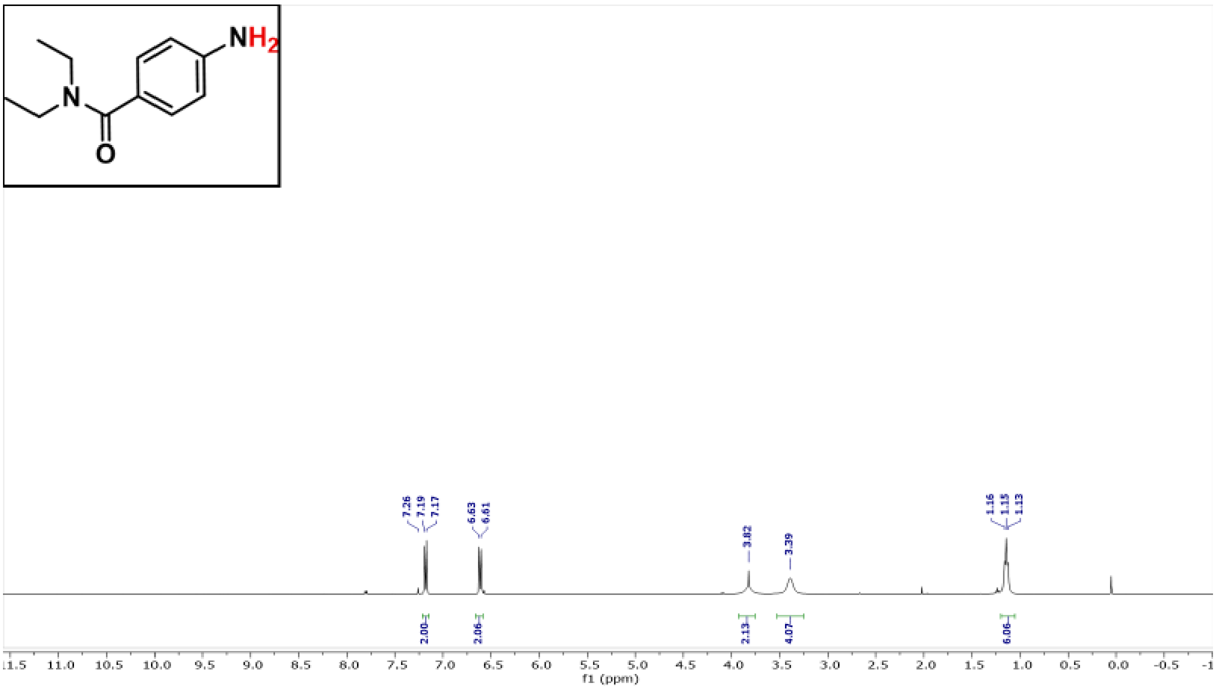

Figure S144 – ^{1}H NMR (400 MHz, CDCl$_3$, 296 K) spectrum of compound **2l**.

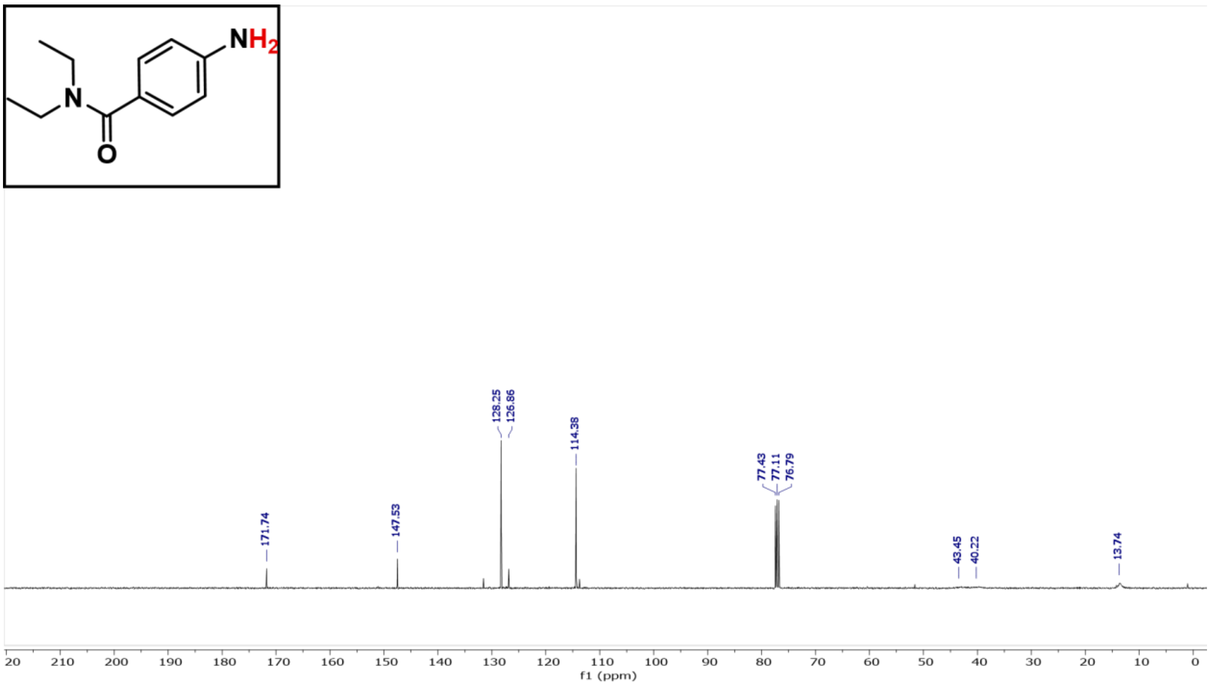

Figure S145 – $^{13}C\{^{1}H\}$ NMR (101 MHz, $CDCl_3$, 296 K) spectrum of compound **2l**.

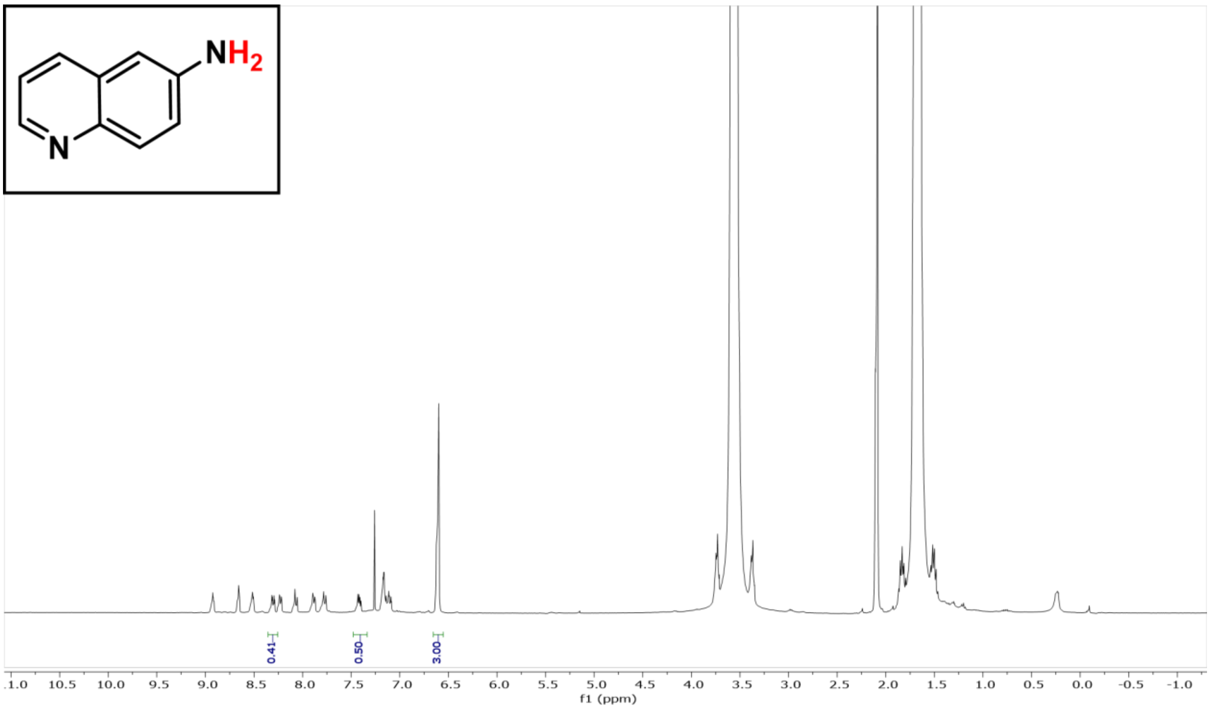

Figure S146 – ^{1}H NMR (400 MHz, DMSO-d_6, 296 K) crude spectrum of compound **2m**.

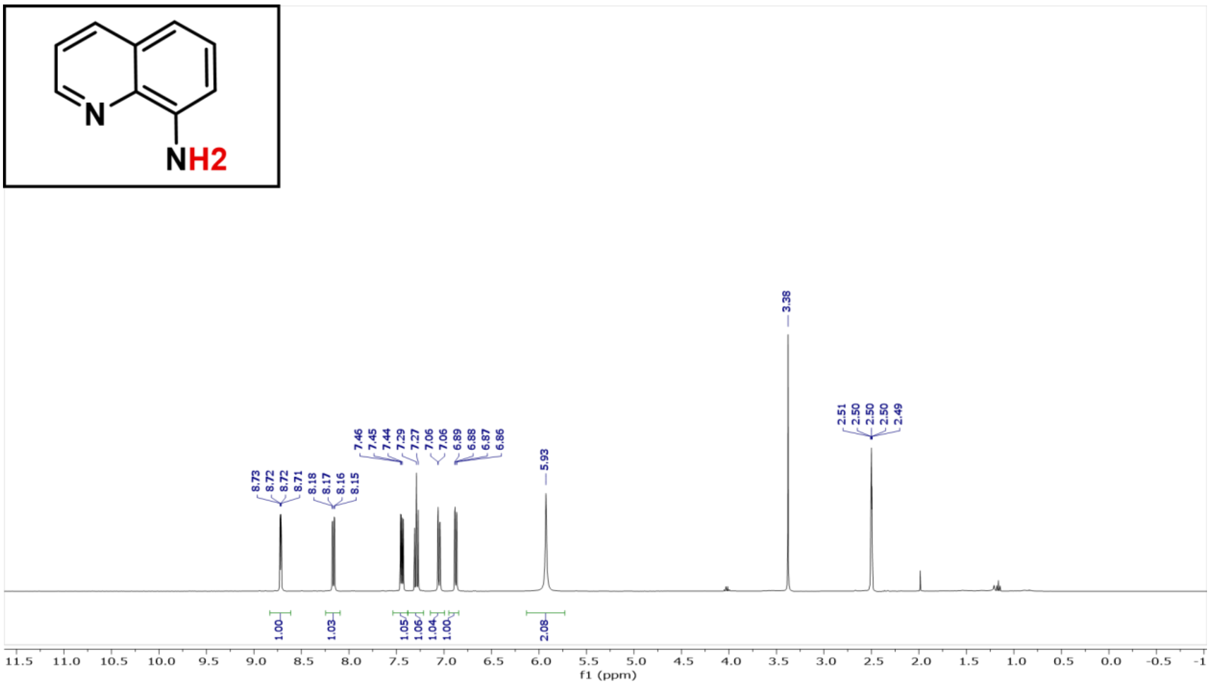

Figure S147 – ^{1}H NMR (400 MHz, DMSO-d_6, 296 K) spectrum of compound **2n**.

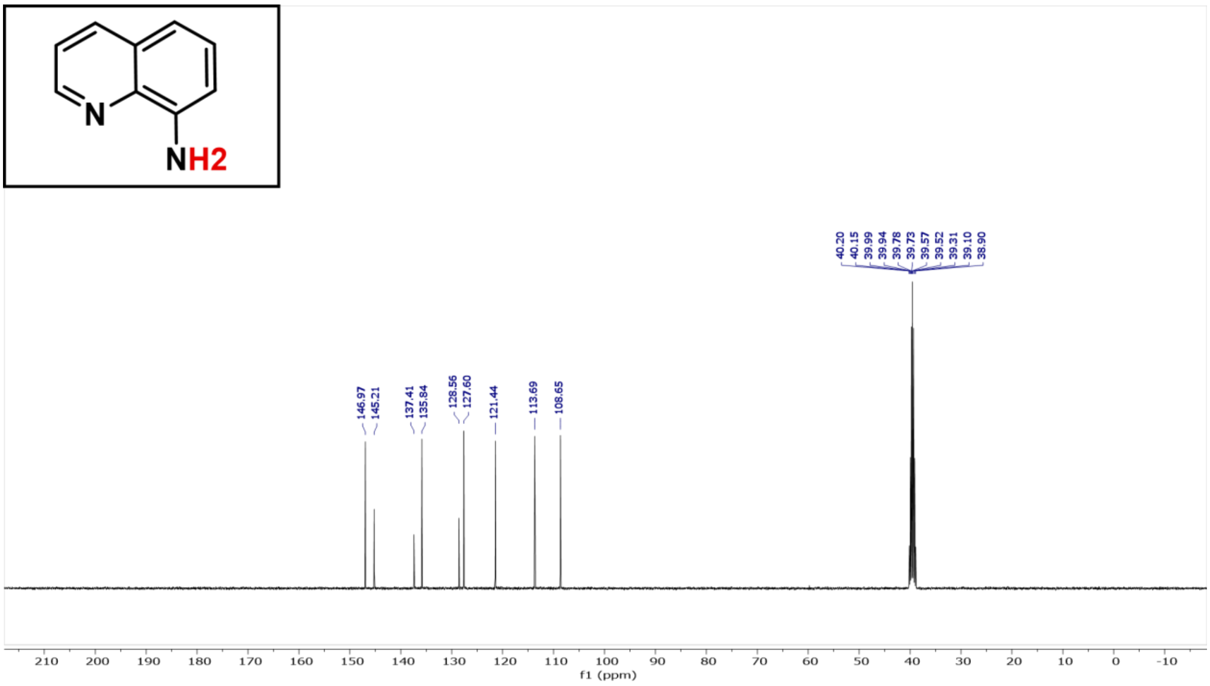

Figure S148 – ^{13}C{^{1}H} NMR (101 MHz, DMSO-d_6, 296 K) spectrum of compound **2n**.

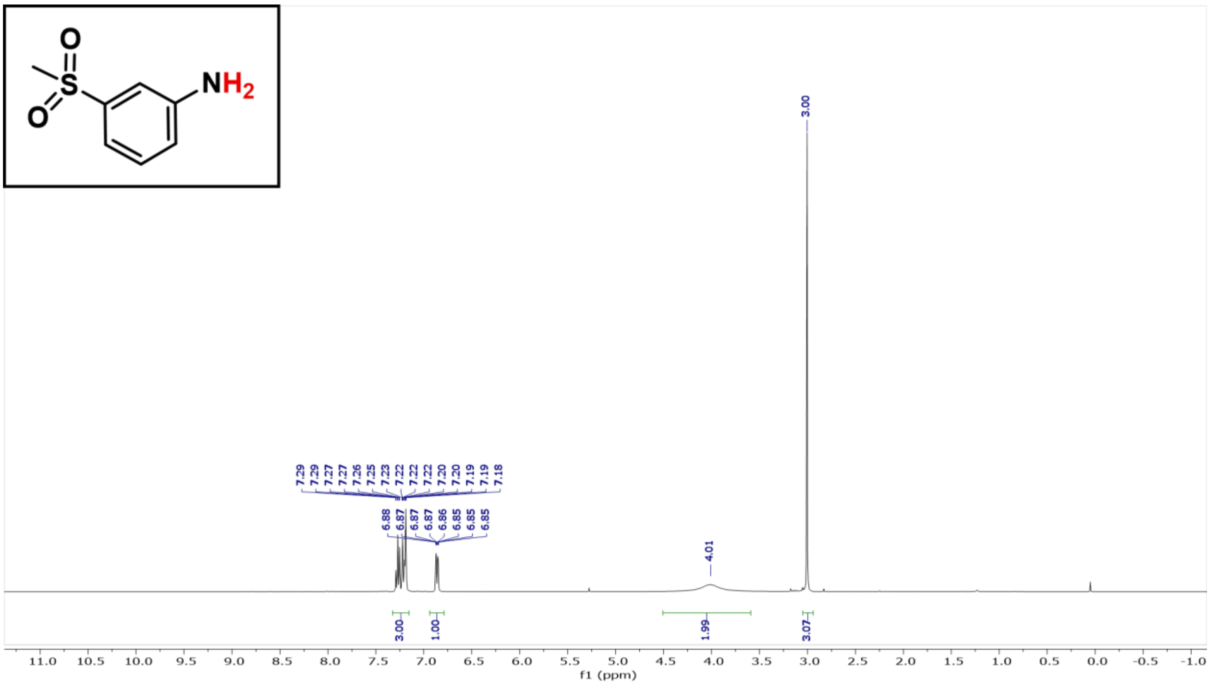

Figure S149 – ^{1}H NMR (400 MHz, CDCl$_3$, 296 K) spectrum of compound **2o**.

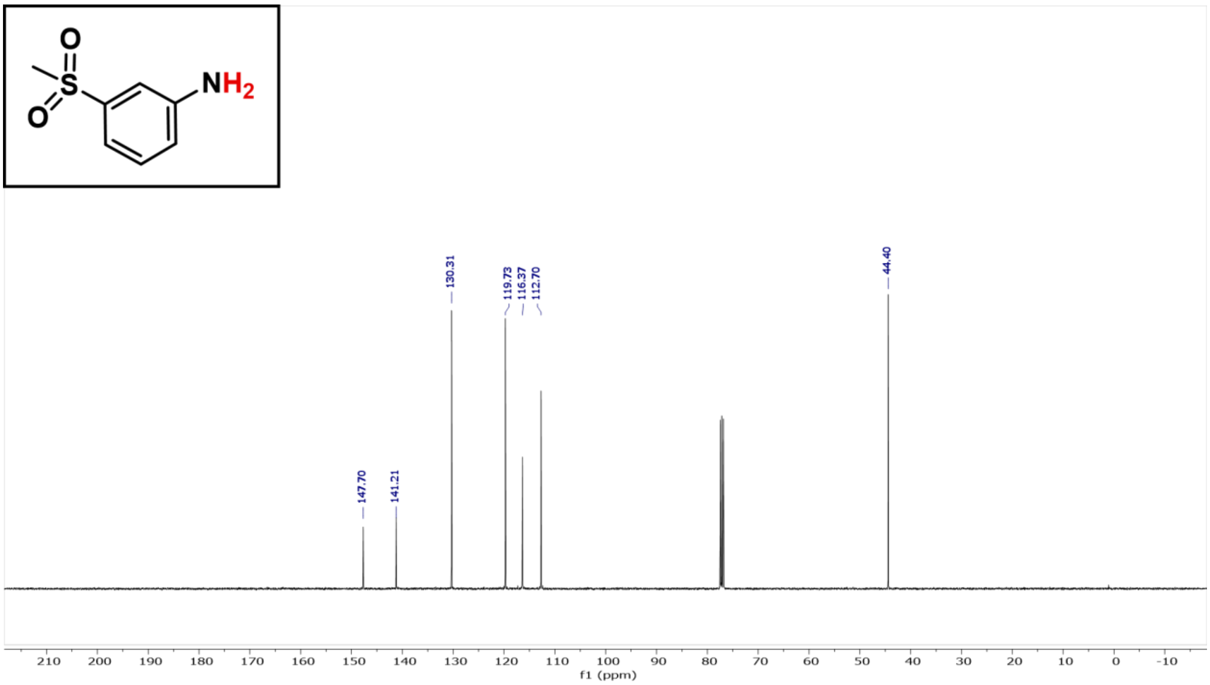

Figure S150 – ^{13}C{^{1}H} NMR (101 MHz, CDCl$_3$, 296 K) spectrum of compound **2o**.

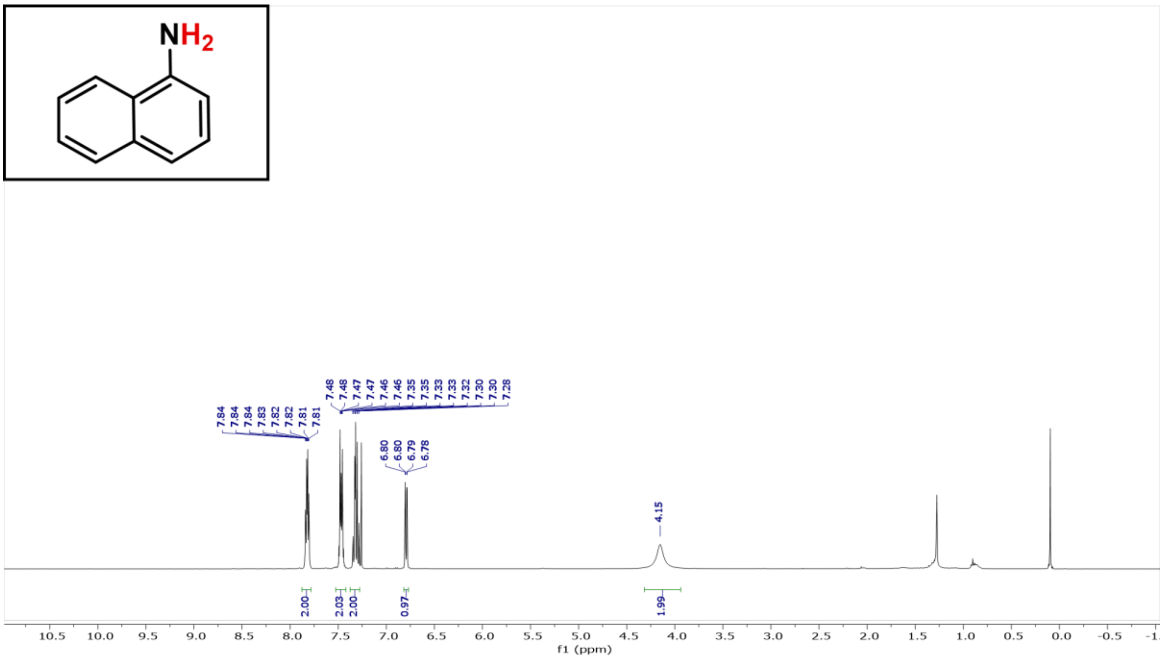

Figure S151 – ^{1}H NMR (400 MHz, $CDCl_3$, 296 K) spectrum of compound **2p**

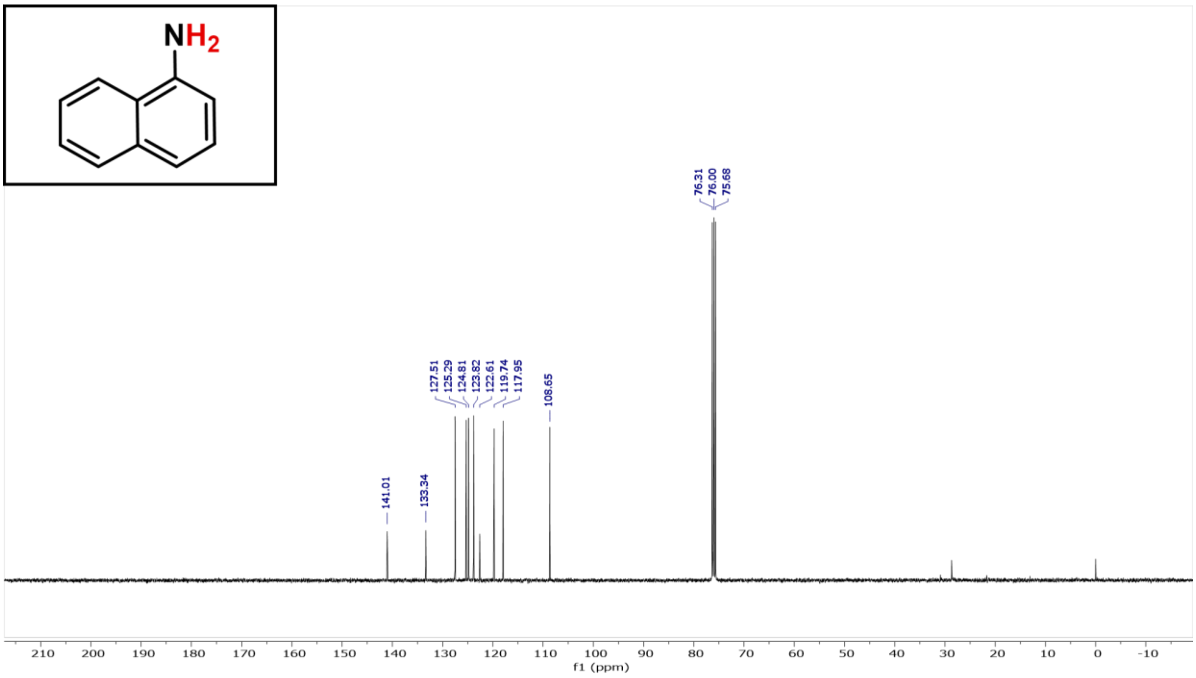

Figure S152 – $^{13}C\{^{1}H\}$ NMR (101 MHz, $CDCl_3$, 296 K) spectrum of compound **2p**. .

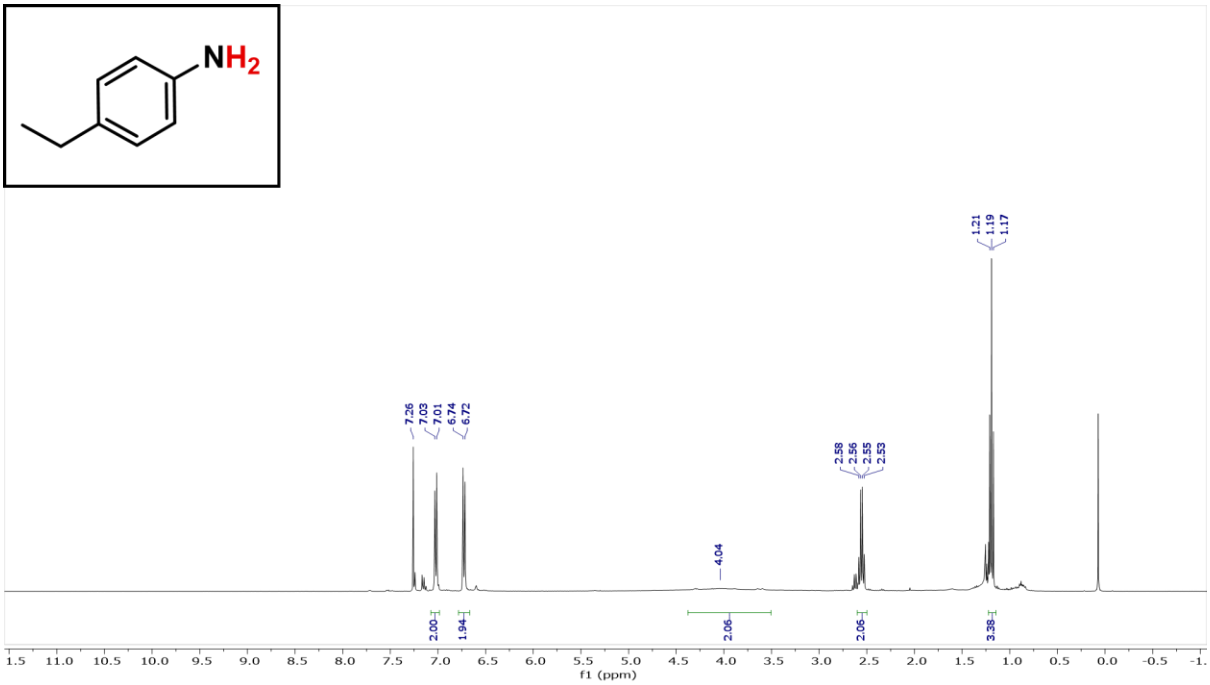

Figure S153 – ^{1}H NMR (400 MHz, CDCl$_3$, 296 K) spectrum of compound **2q**.

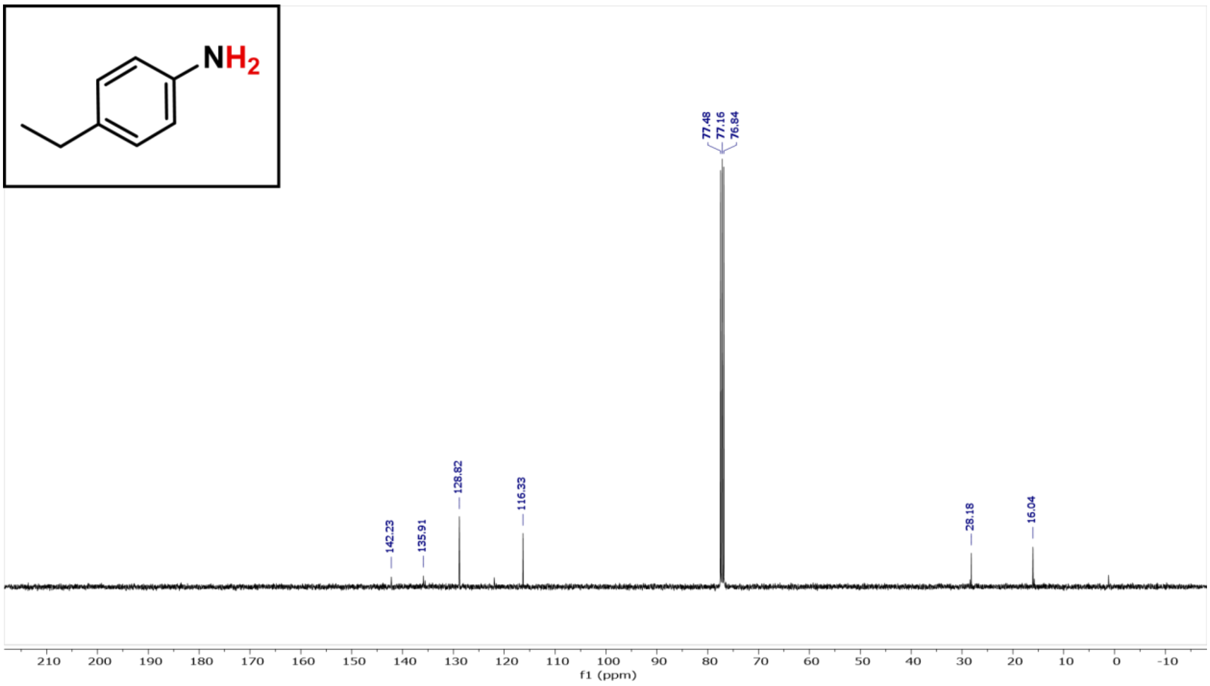

Figure S154 – ^{13}C{^{1}H} NMR (101 MHz, CDCl$_3$, 296 K) spectrum of compound **2q**.

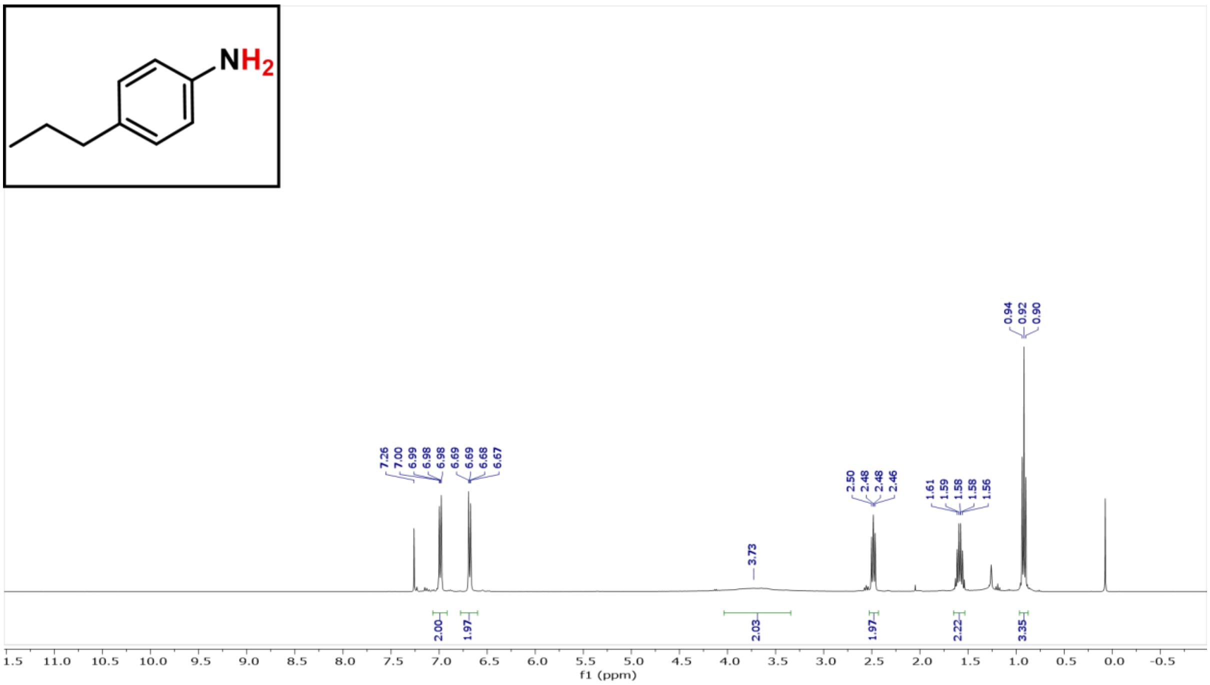

Figure S155 – ^{1}H NMR (400 MHz, CDCl$_3$, 296 K) spectrum of compound **2r**.

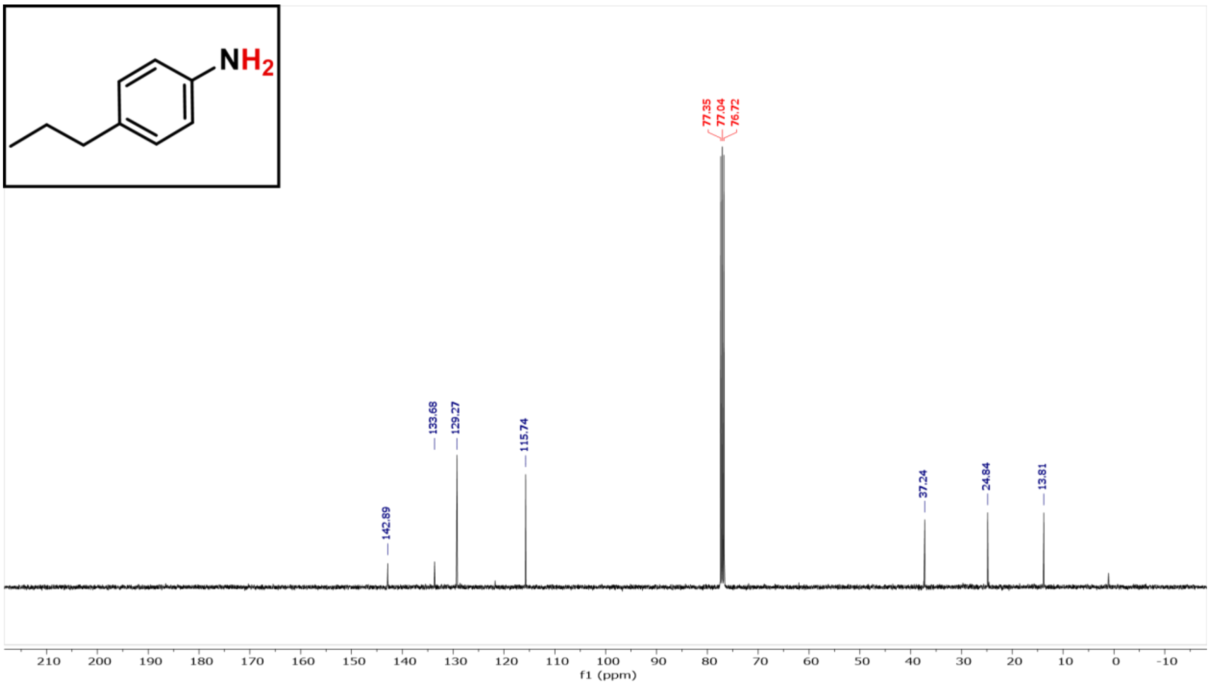

Figure S156 – ^{13}C{^{1}H} NMR (101 MHz, CDCl$_3$, 296 K) spectrum of compound **2r**.

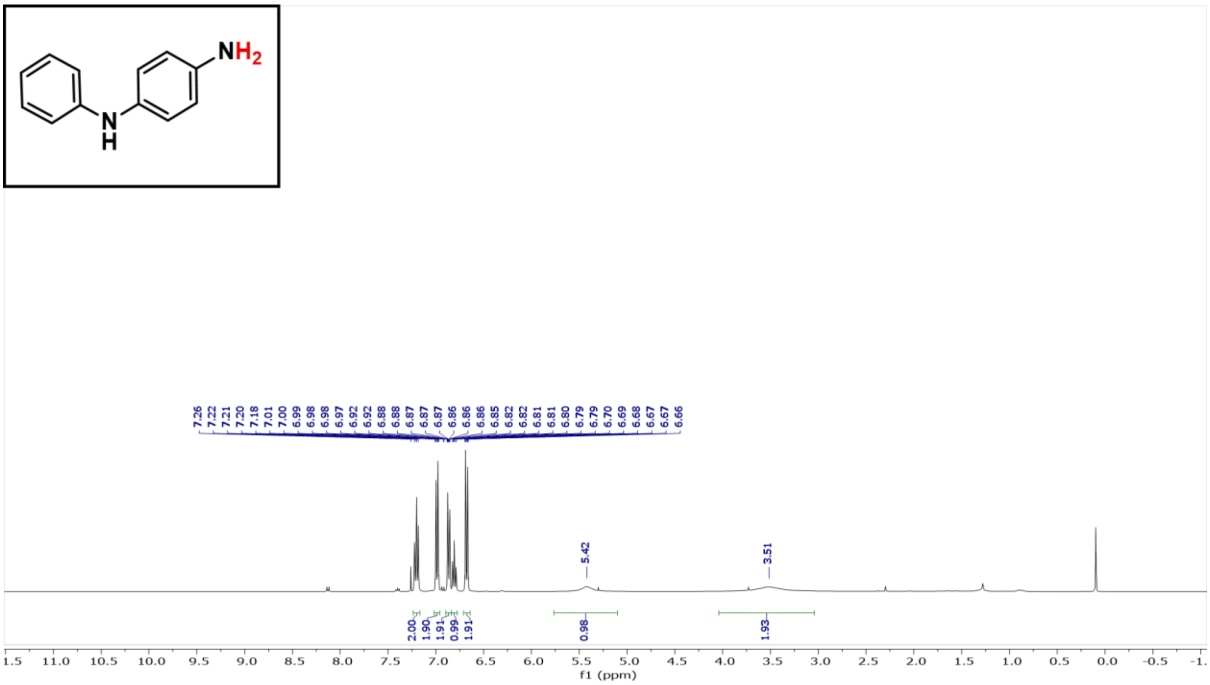

Figure S157 – ^{1}H NMR (400 MHz, CDCl$_3$, 296 K) spectrum of compound **2s**

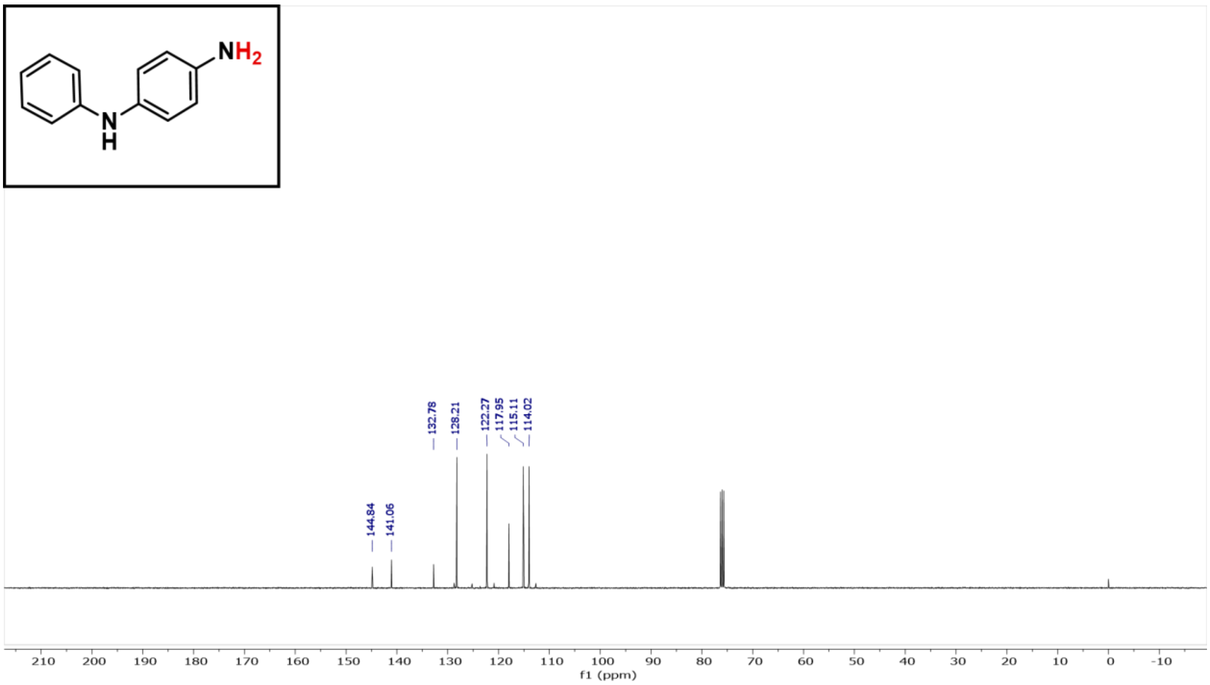

Figure S158 – ^{13}C{^{1}H} NMR (101 MHz, CDCl3, 296 K) spectrum of compound **2s**. .

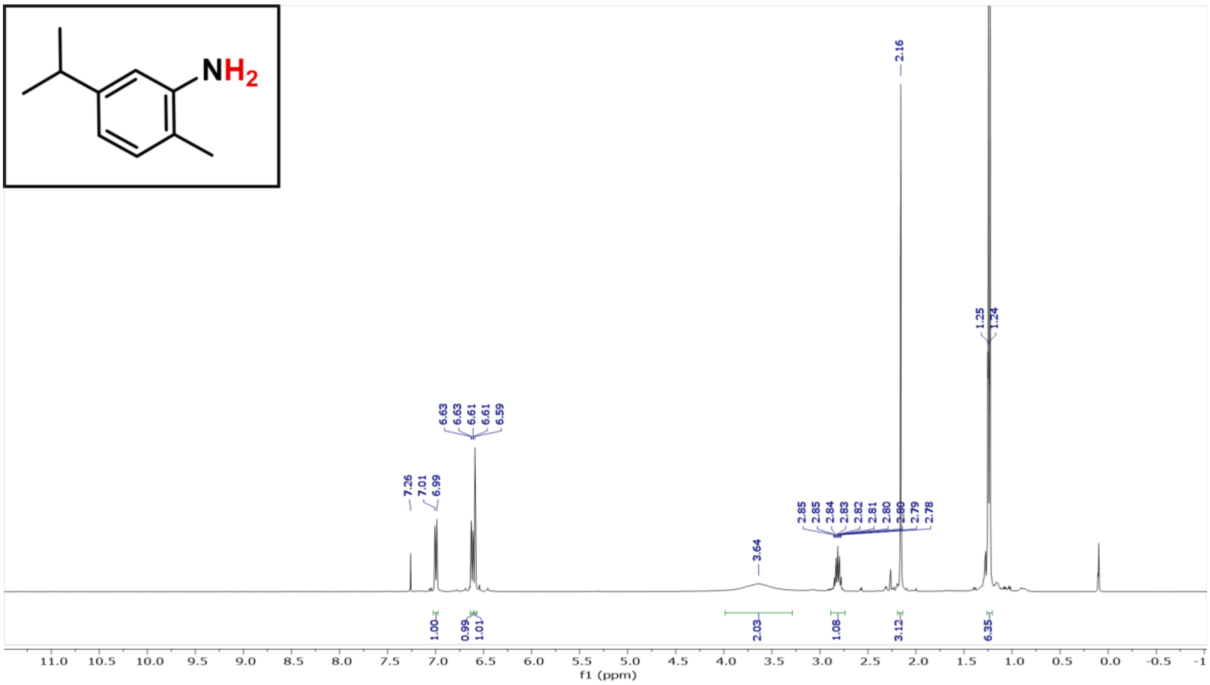

Figure S159 – ^{1}H NMR (400 MHz, CDCl$_3$, 296 K) spectrum of compound **2t**.

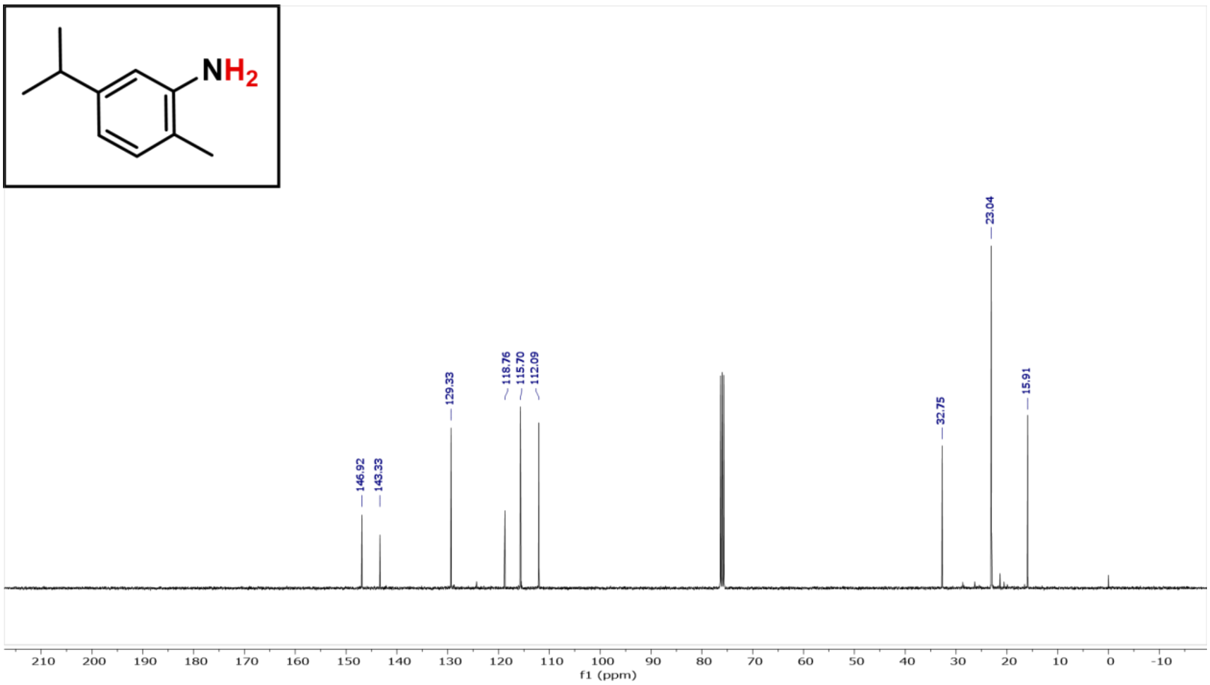

Figure S160 – ^{13}C{^{1}H} NMR (101 MHz, CDCl$_3$, 296 K) spectrum of compound **2t**.

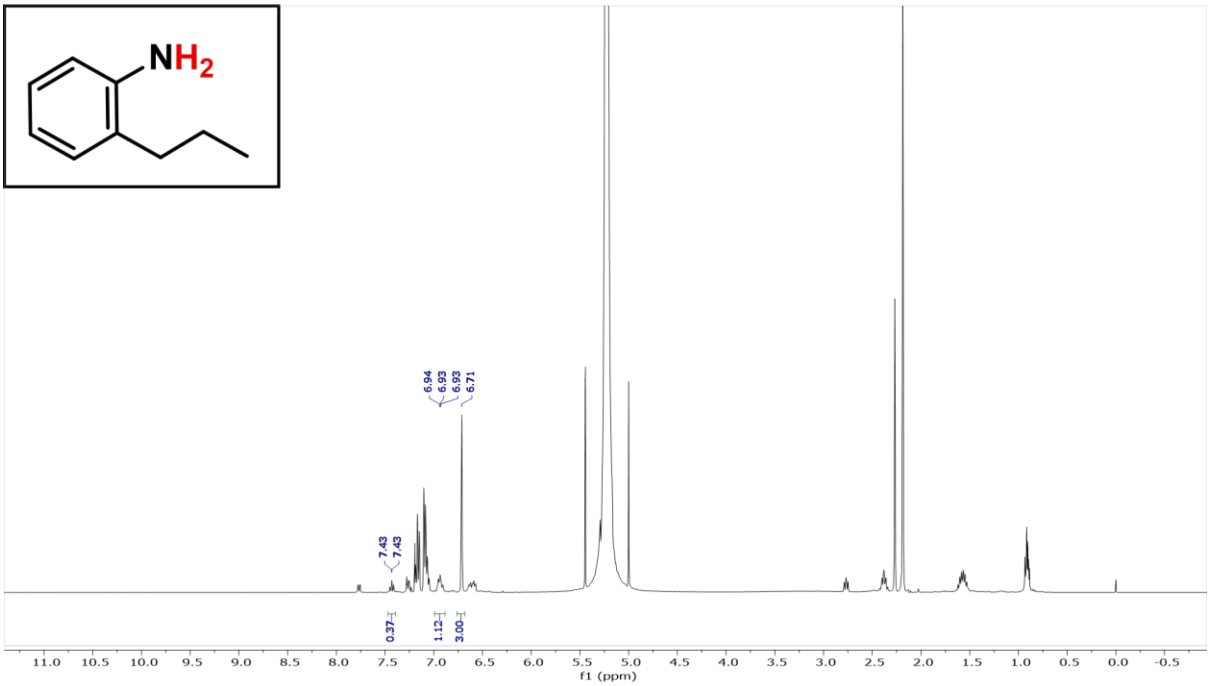

Figure S161 – ^{1}H NMR (400 MHz, CDCl$_3$, 296 K) crude spectrum of compound **2u**

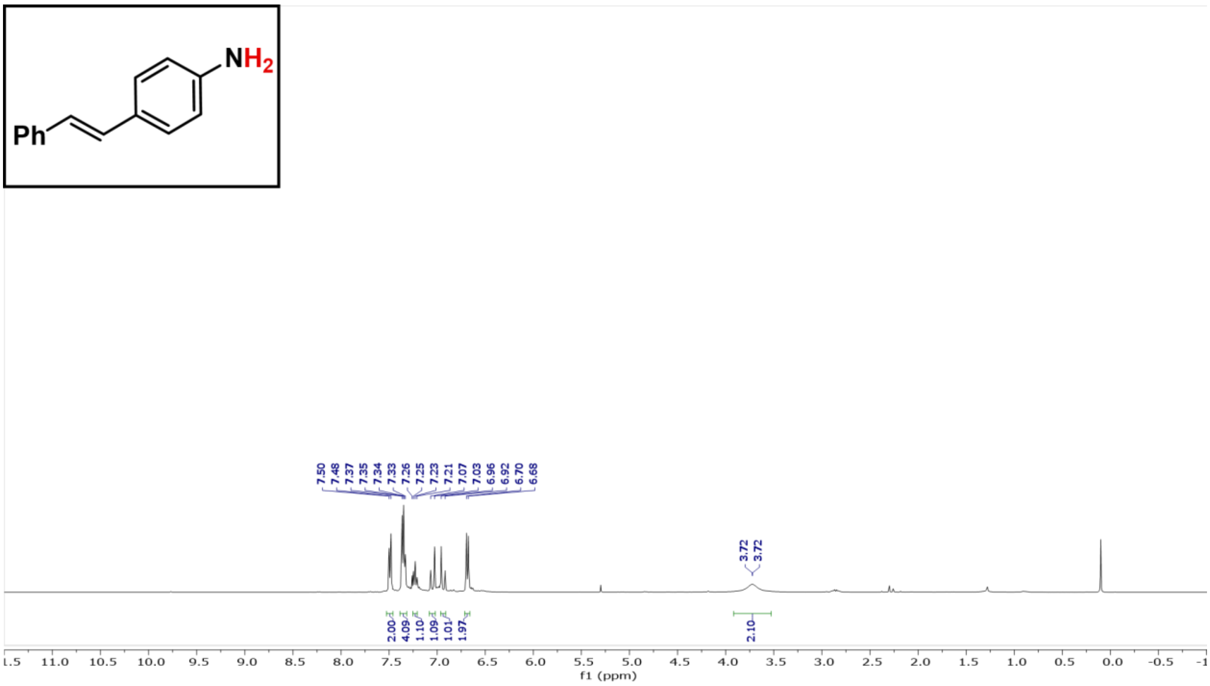

Figure S162 – ^{1}H NMR (400 MHz, CDCl$_3$, 296 K) spectrum of compound **2v**.

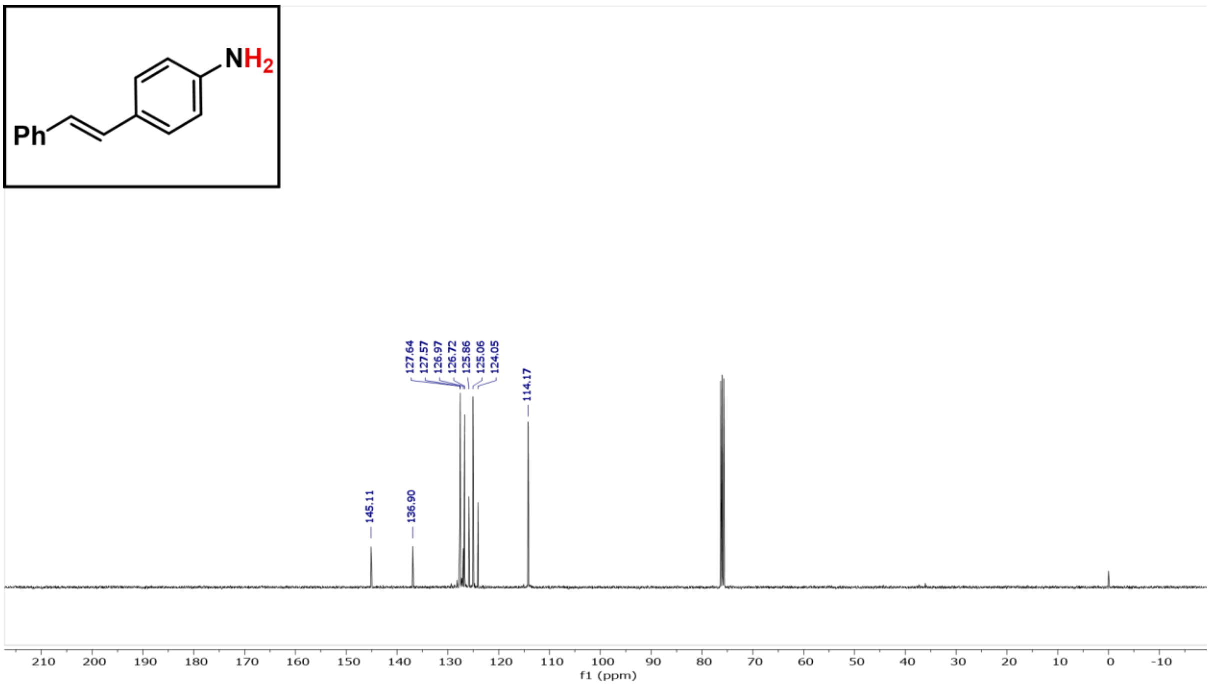

Figure S163 – $^{13}C\{^{1}H\}$ NMR (101 MHz, $CDCl_3$, 296 K) spectrum of compound **2v**.

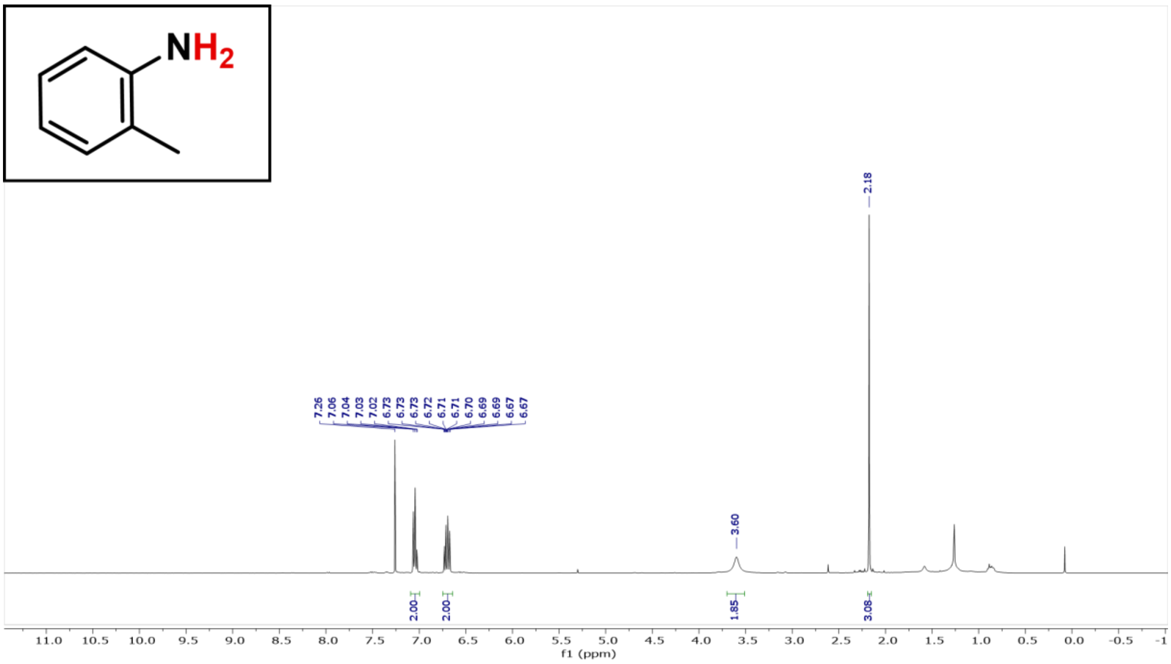

Figure S164 – ^{1}H NMR (400 MHz, $CDCl_3$, 296 K) spectrum of compound **2w**.

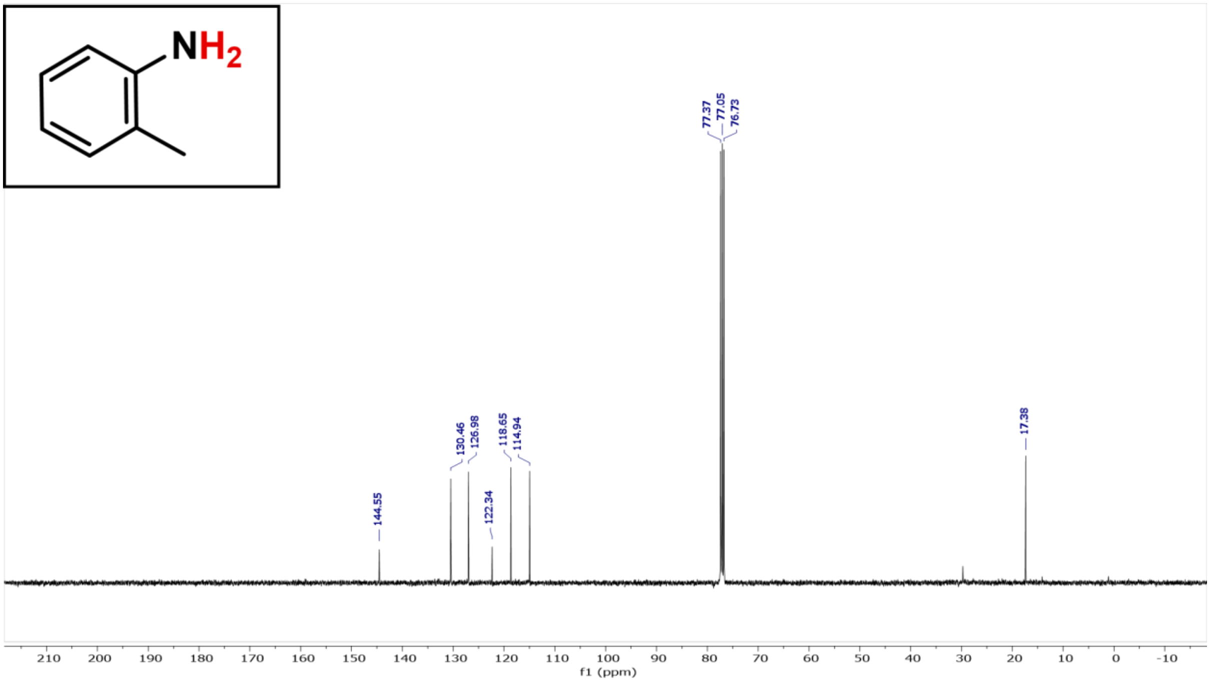

Figure S165 – $^{13}C\{^{1}H\}$ NMR (101 MHz, $CDCl_3$, 296 K) spectrum of compound **2w**.

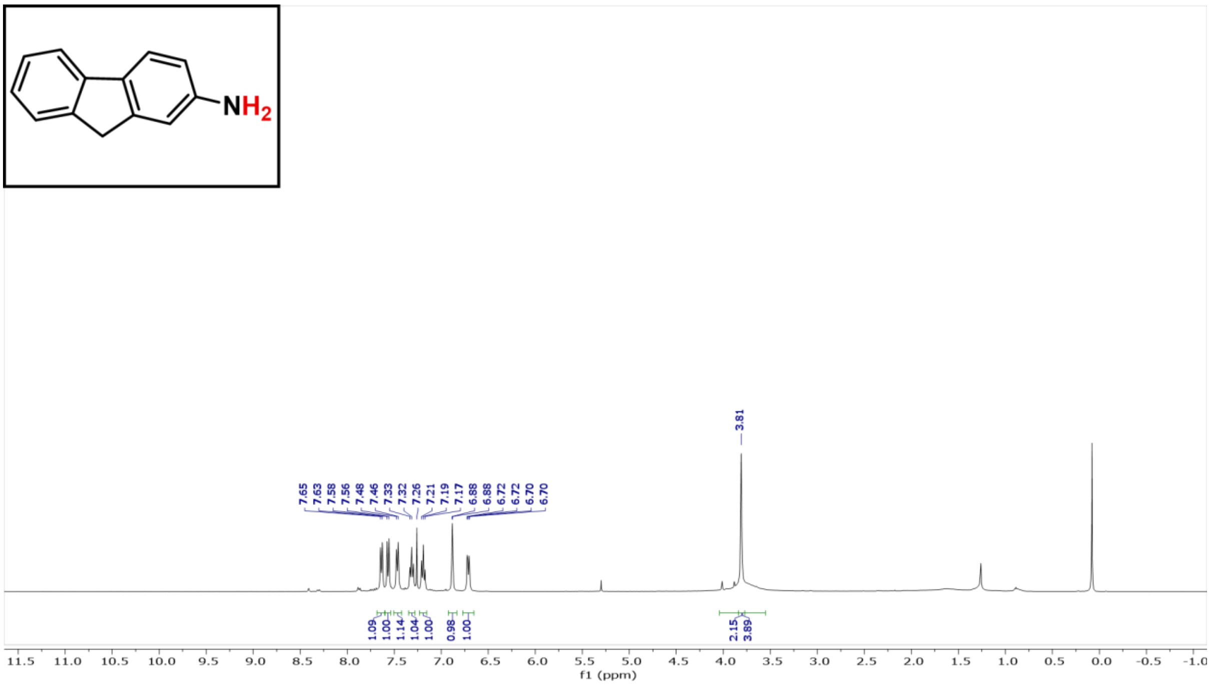

Figure S166 – ^{1}H NMR (400 MHz, $CDCl_3$, 296 K) spectrum of compound **2x**.

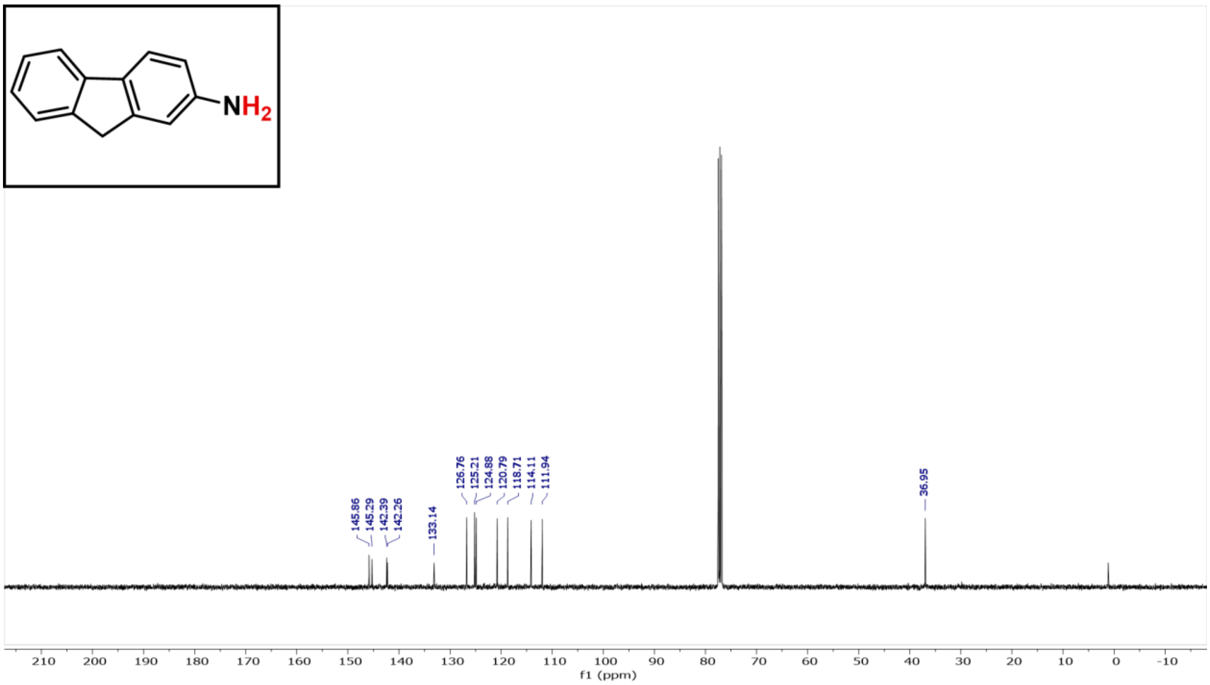

Figure S167 – $^{13}C\{^{1}H\}$ NMR (101 MHz, $CDCl_3$, 296 K) spectrum of compound **2x**.

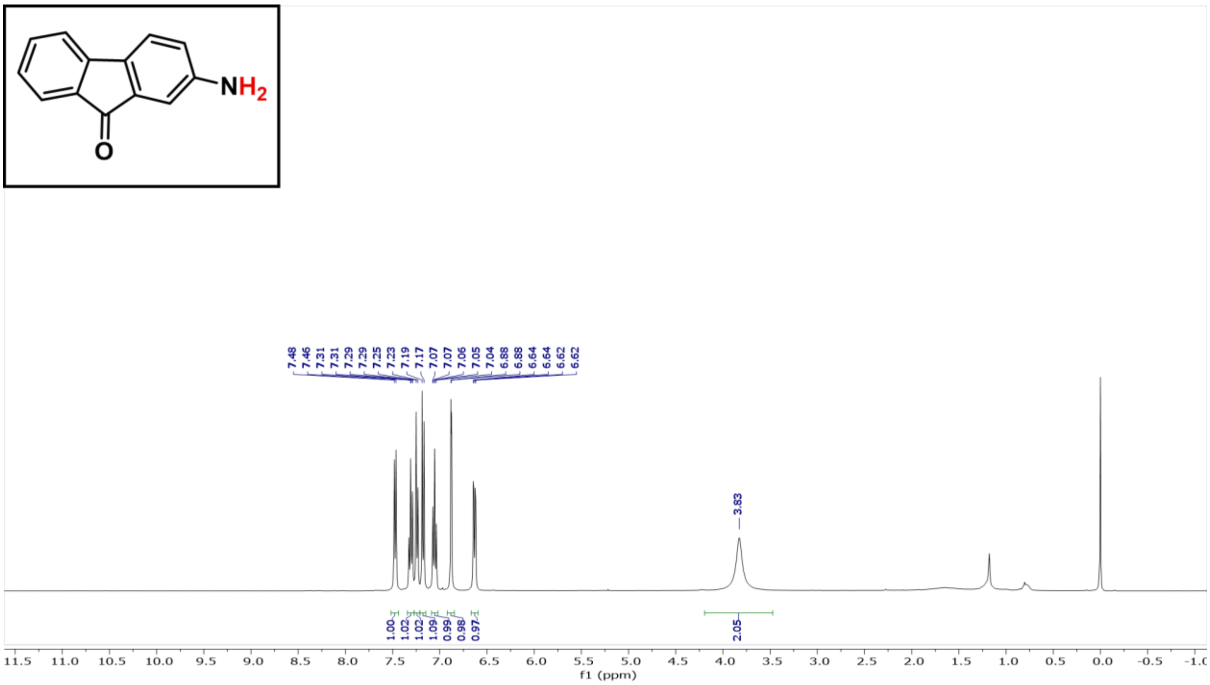

Figure S168 – ^{1}H NMR (400 MHz, $CDCl_3$, 296 K) spectrum of compound **2y**.

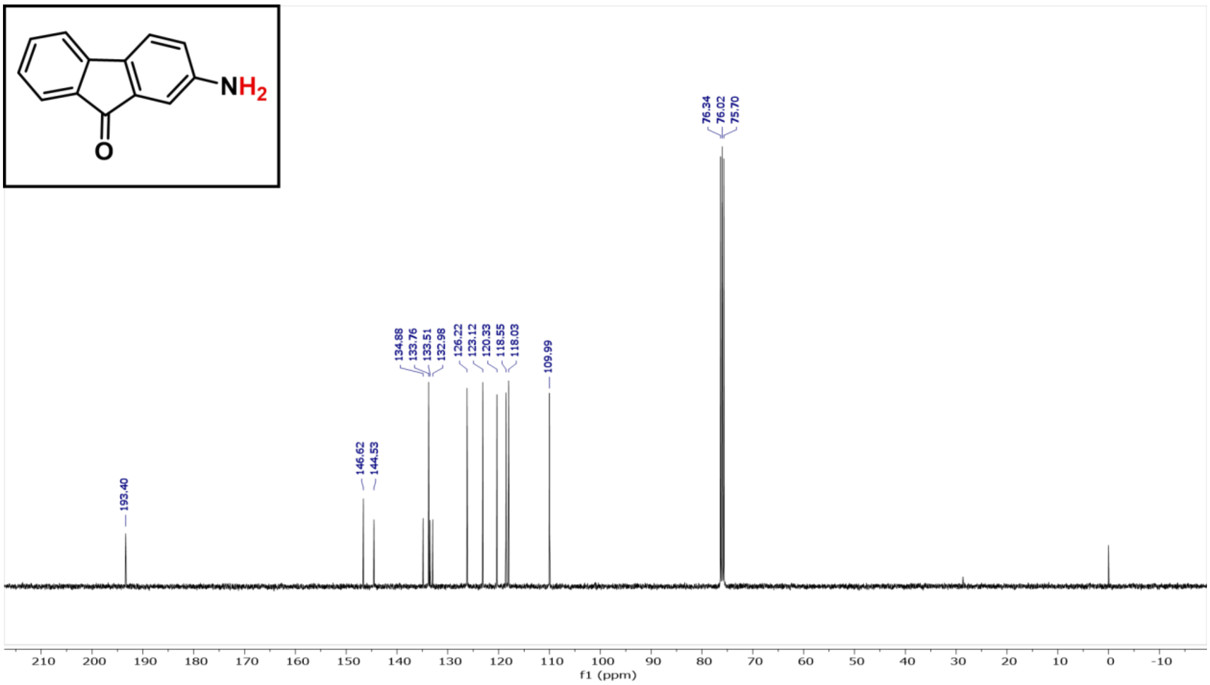

Figure S169 – $^{13}C\{^{1}H\}$ NMR (101 MHz, $CDCl_3$, 296 K) spectrum of compound **2y**.

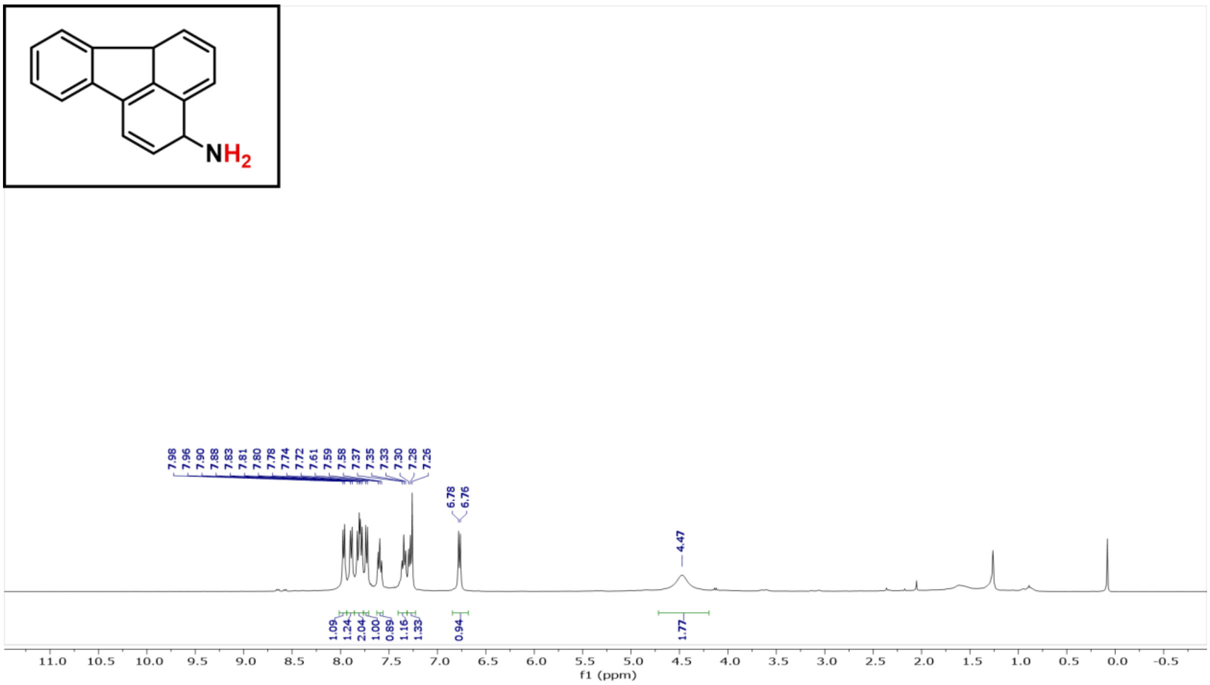

Figure S170 – ^{1}H NMR (400 MHz, $CDCl_3$, 296 K) spectrum of compound **2z**.

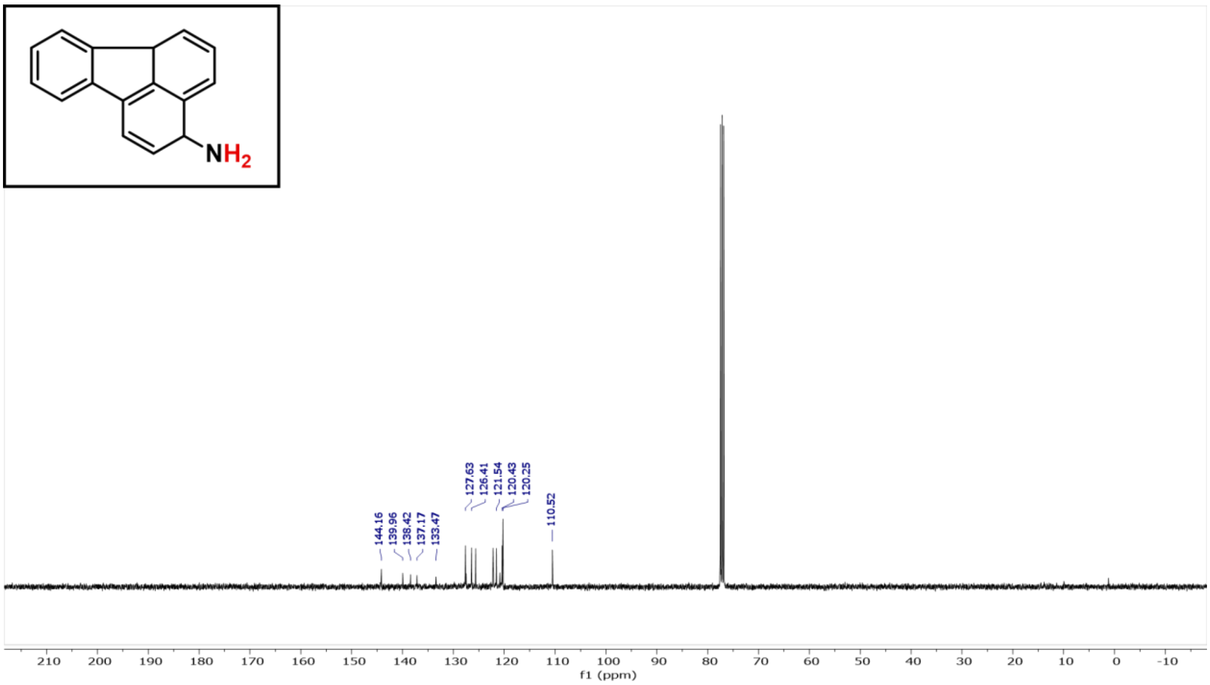

Figure S171 – ^{13}C{^{1}H} NMR (101 MHz, CDCl$_3$, 296 K) spectrum of compound **2z**.

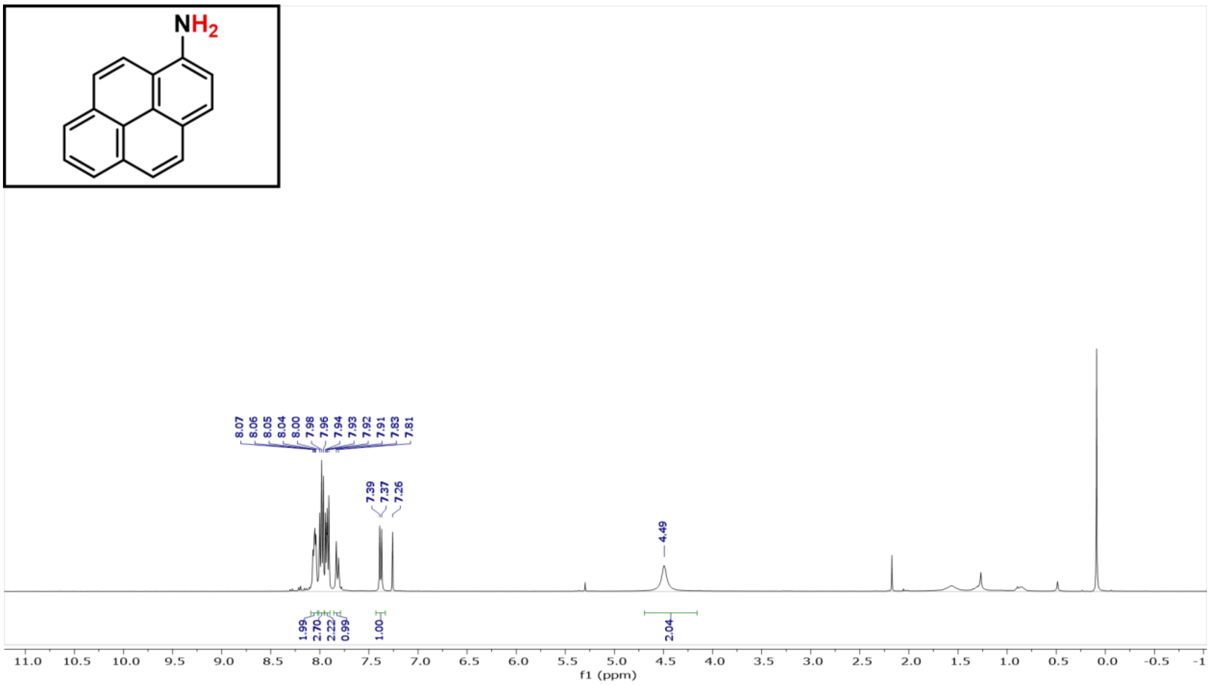

Figure S172 – ^{1}H NMR (400 MHz, CDCl$_3$, 296 K) spectrum of compound **2aa**.

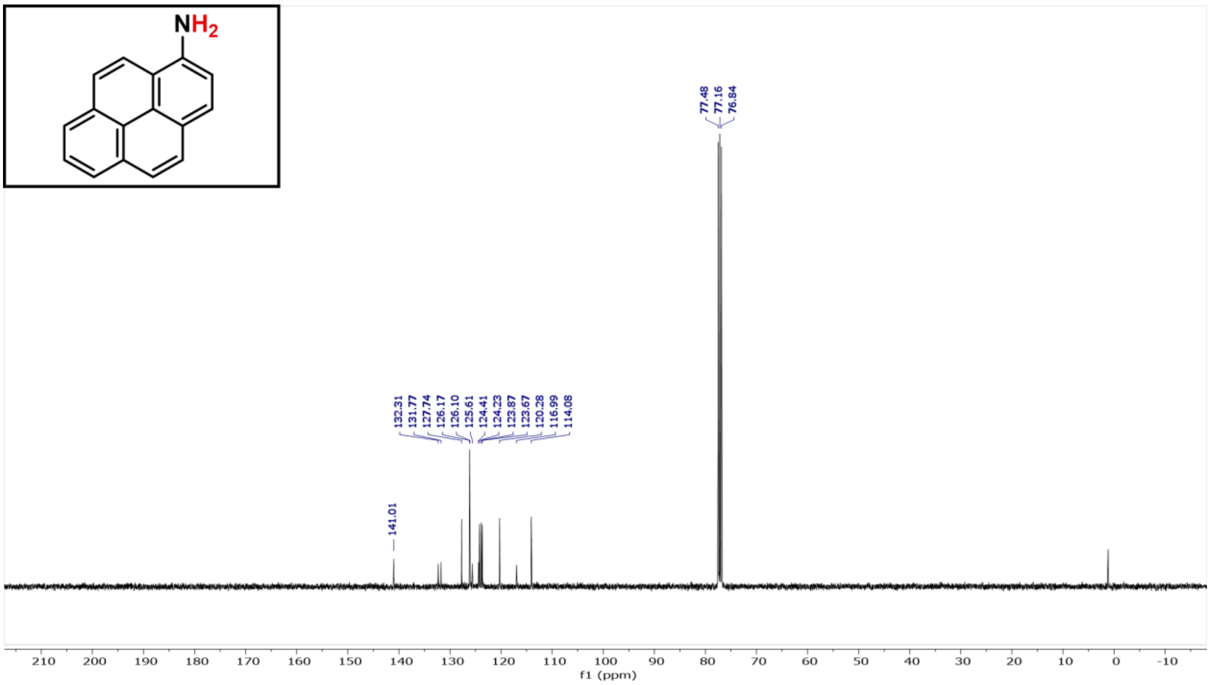

Figure S173 – $^{13}C\{^{1}H\}$ NMR (101 MHz, $CDCl_3$, 296 K) spectrum of compound **2aa**.

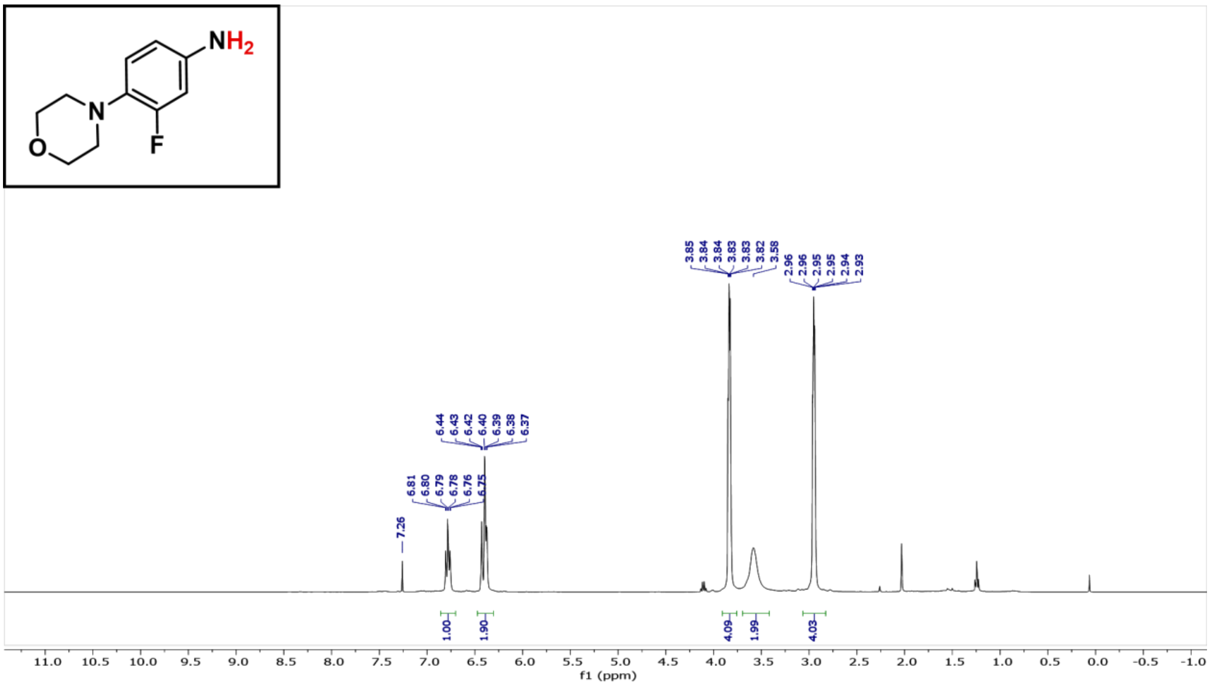

Figure S174 – ^{1}H NMR (400 MHz, $CDCl_3$, 296 K) spectrum of compound **2ab**.

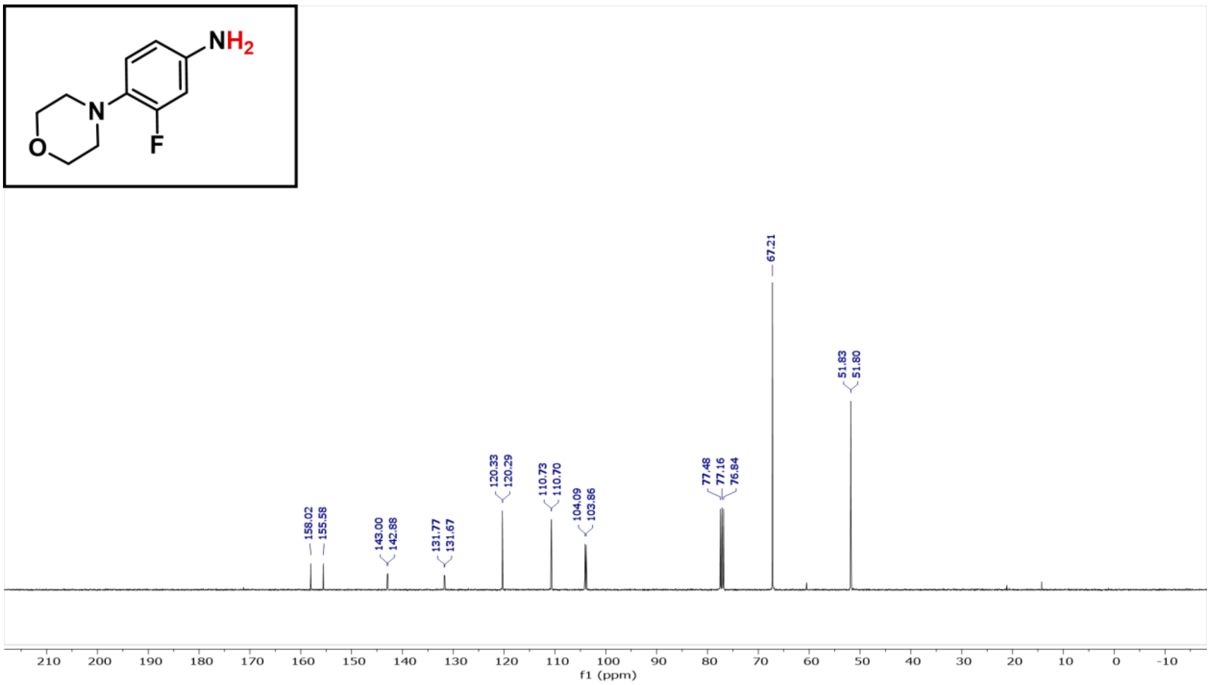

Figure S175 – ^{13}C{^{1}H} NMR (101 MHz, $CDCl_3$, 296 K) spectrum of compound **2ab**.

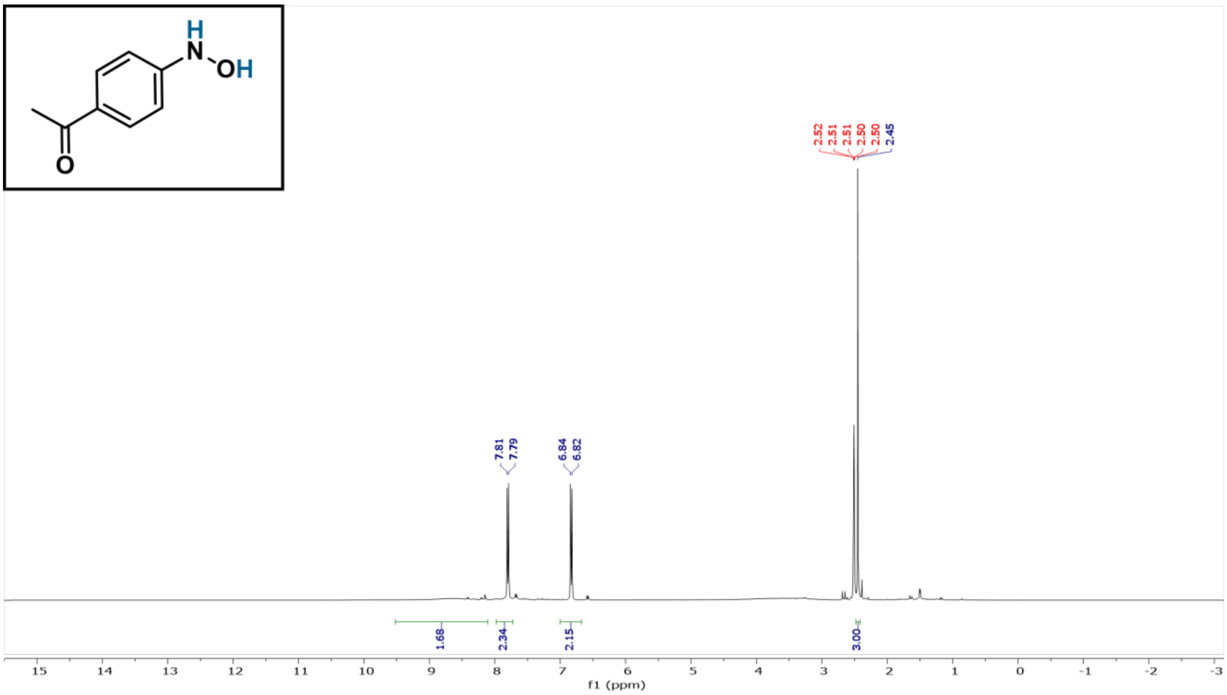

Figure S176 – ^{1}H NMR (400 MHz, DMSO-d_6, 296 K) spectrum of compound **3a**.

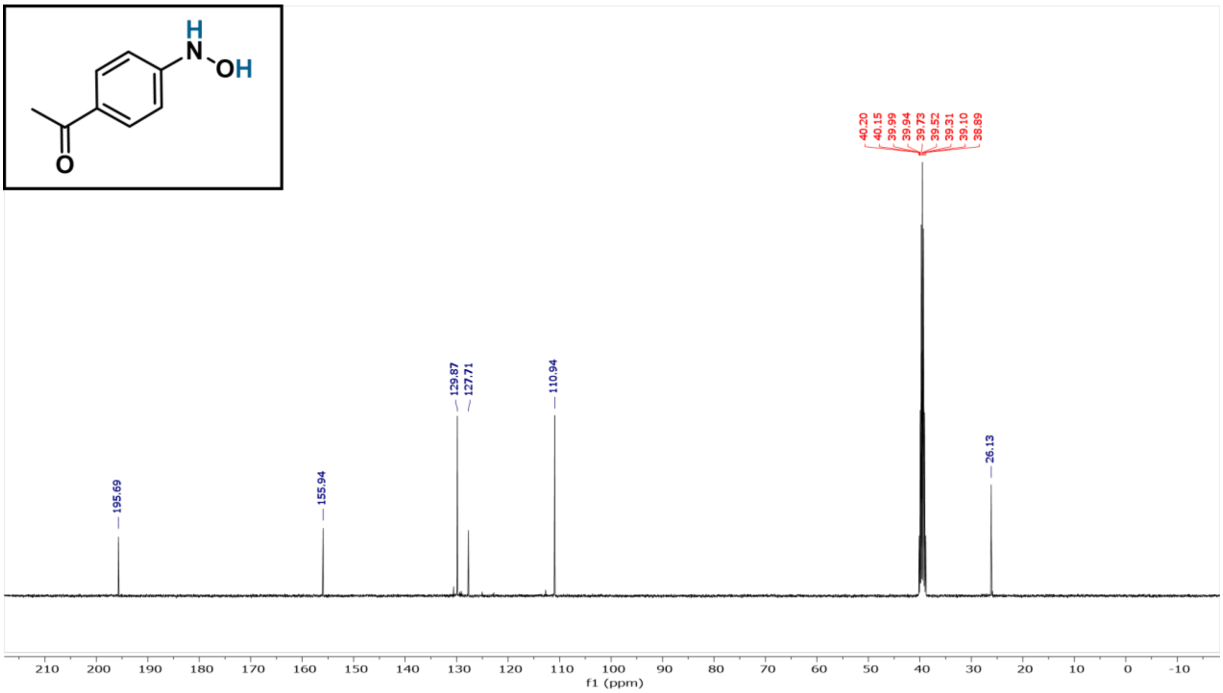

Figure S177 – ^{13}C{^{1}H} NMR (101 MHz, DMSO-d_6, 296 K) spectrum of compound **3a**.

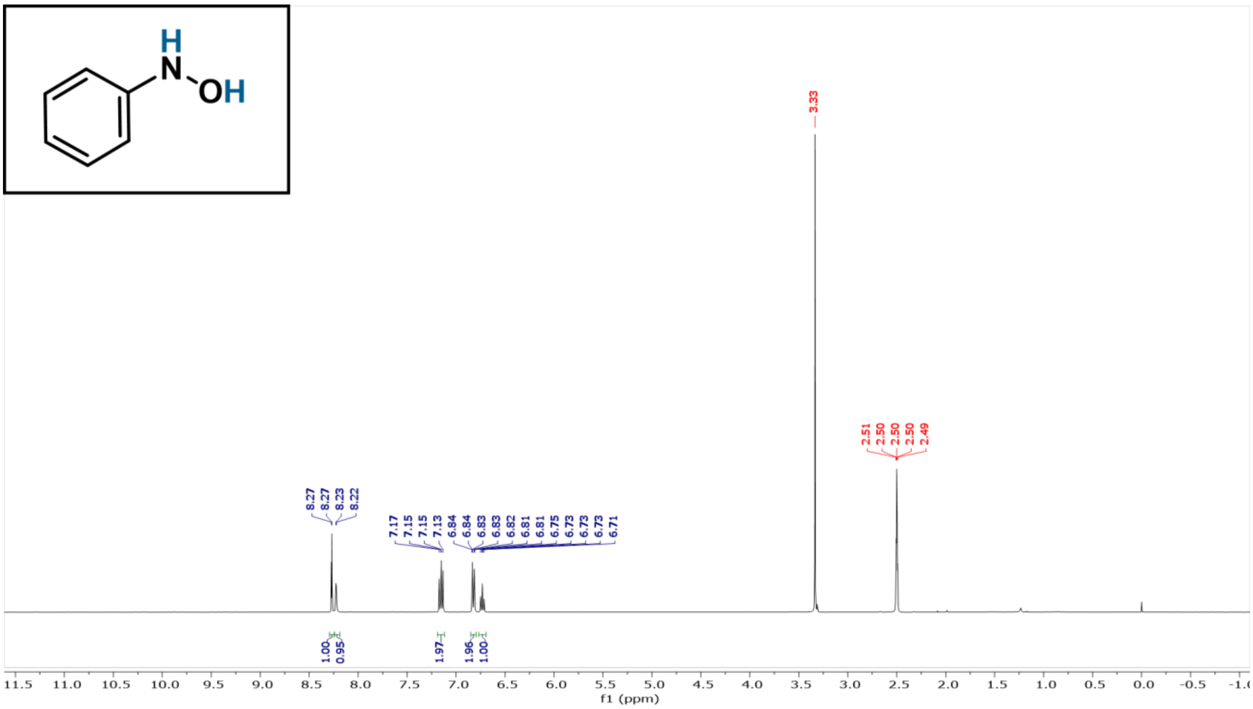

Figure S178 – ^{1}H NMR (400 MHz, DMSO-d_6, 296 K) spectrum of compound **3b**.

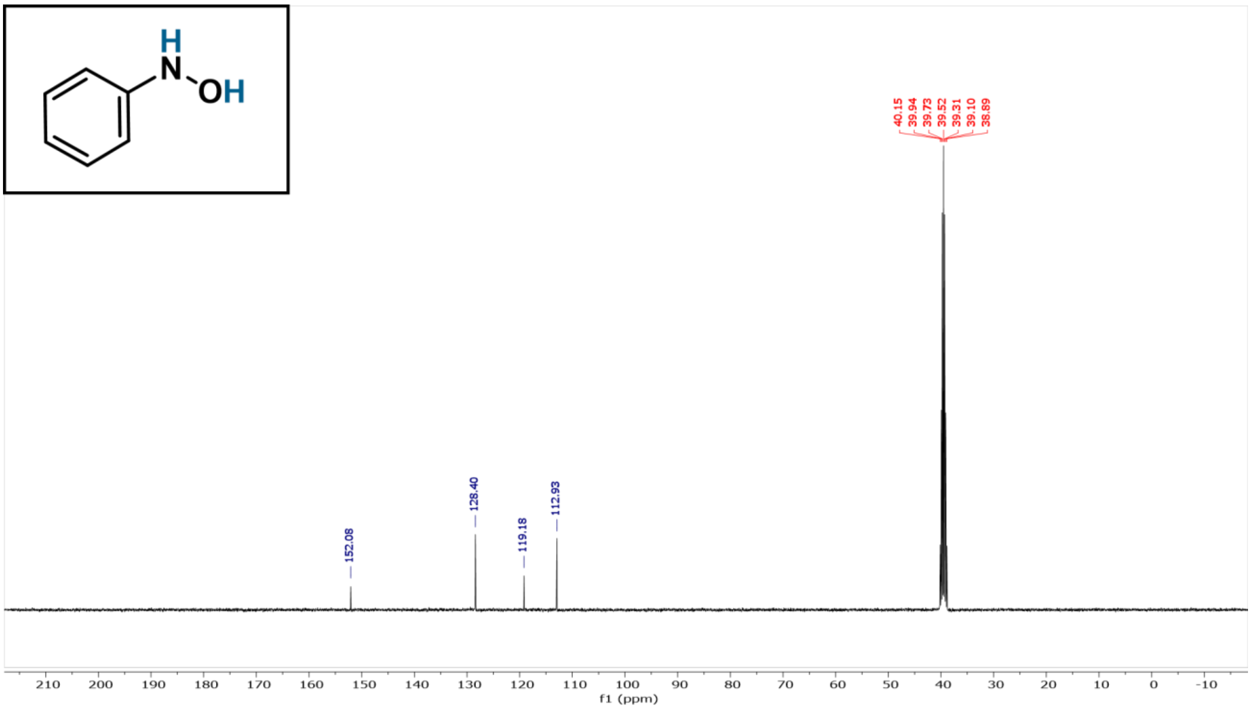

Figure S179 – ^{13}C{^{1}H} NMR (101 MHz, DMSO-d_6, 296 K) spectrum of compound **3b**.

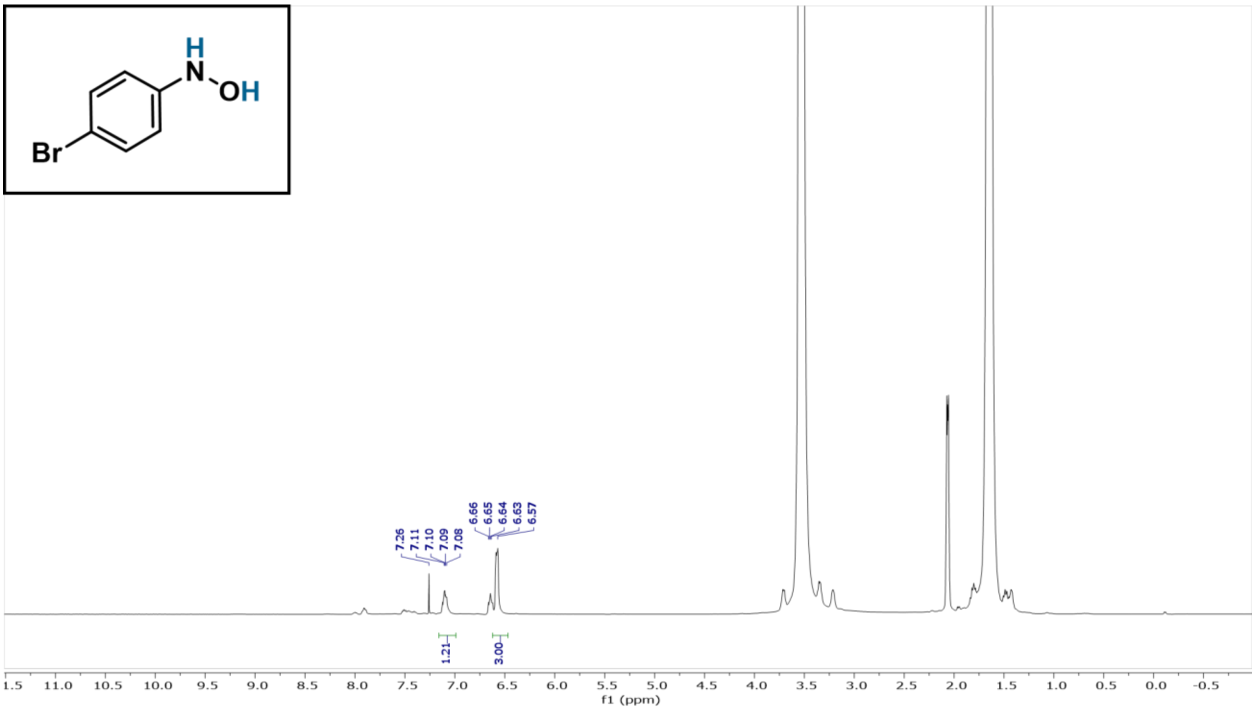

Figure S180 – **^{1}H NMR (400 MHz, DMSO-d_6, 296 K)** crude spectrum of compound **3c.**

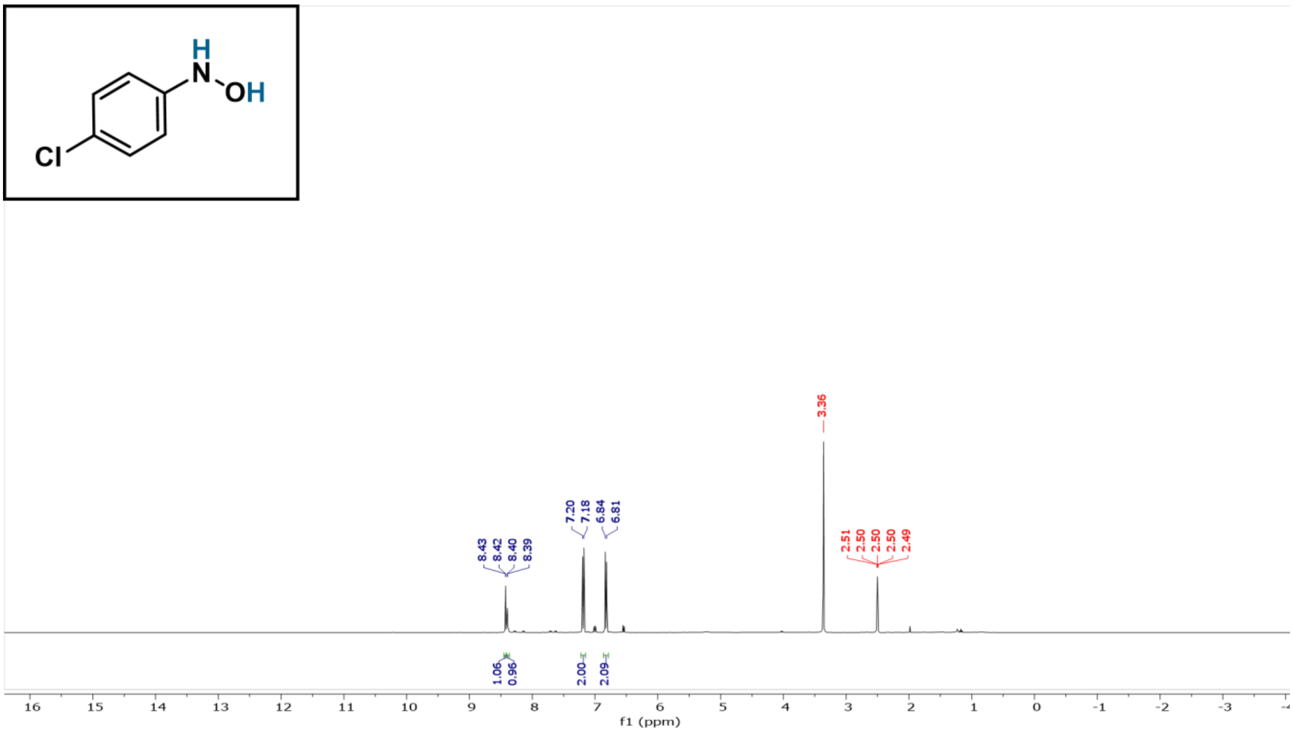

Figure S181 – ^{1}H NMR (400 MHz, DMSO-d_6, 296 K) spectrum of compound **3d**.

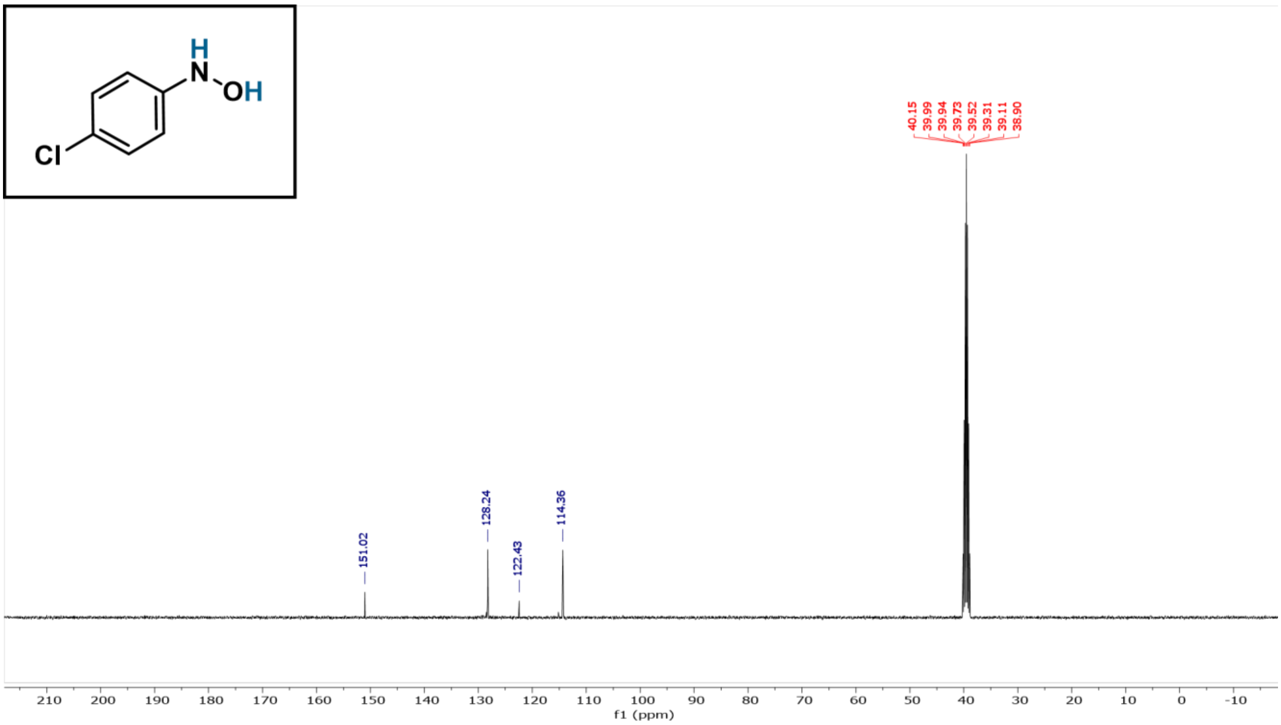

Figure S182 – $^{13}C\{^{1}H\}$ NMR (101 MHz, DMSO-d_6, 296 K) spectrum of compound **3d**.

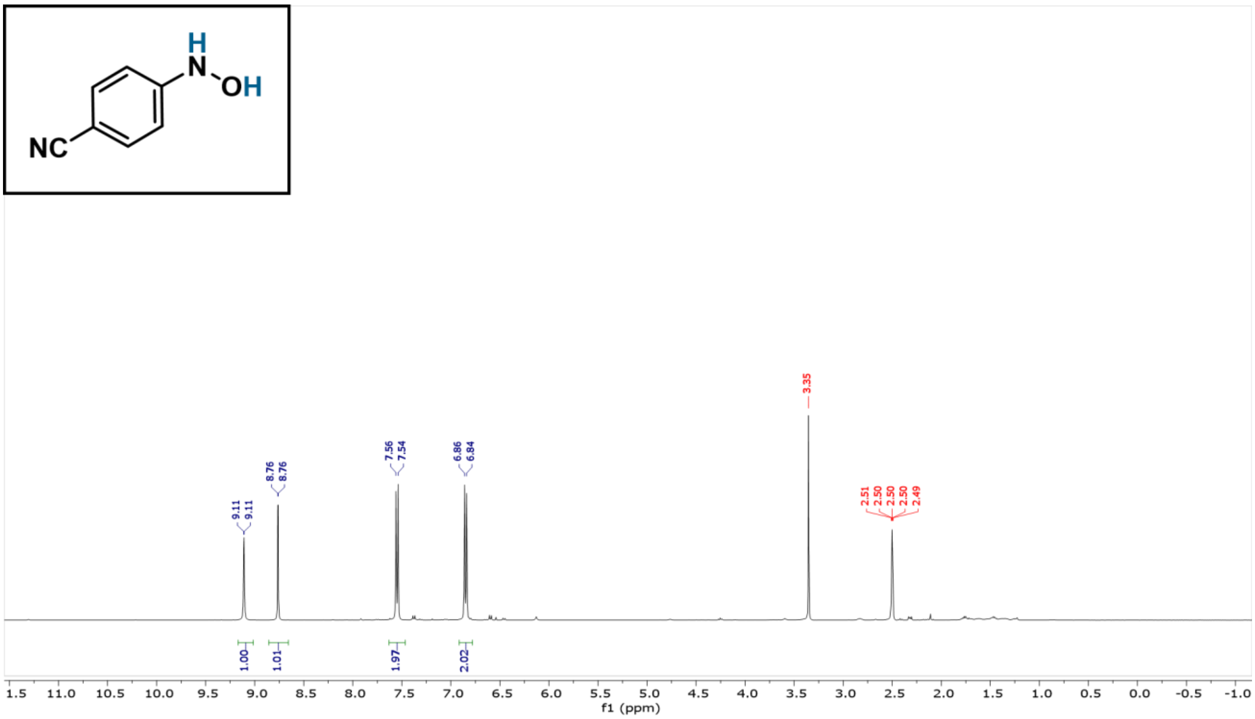

Figure S183 – ^{1}H NMR (400 MHz, DMSO-d_6, 296 K) spectrum of compound **3e**.

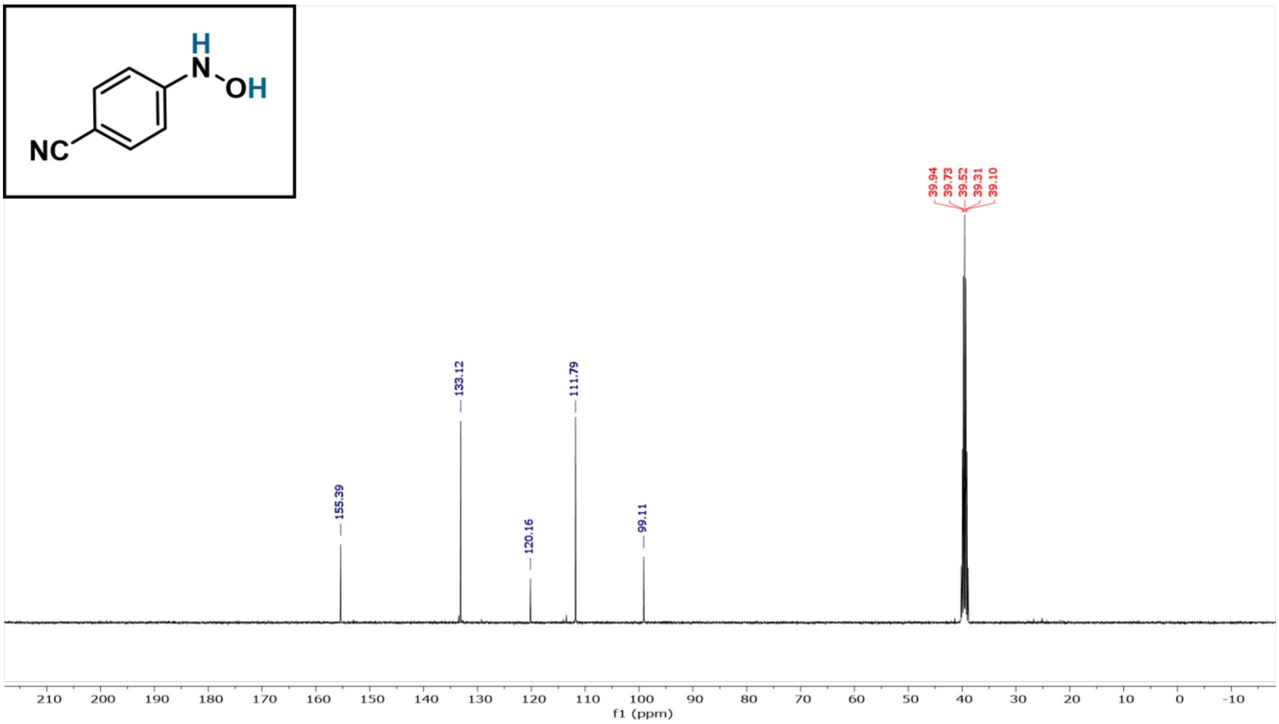

Figure S184 – $^{13}C\{^{1}H\}$ NMR (101 MHz, DMSO-d_6, 296 K) spectrum of compound **3e**.

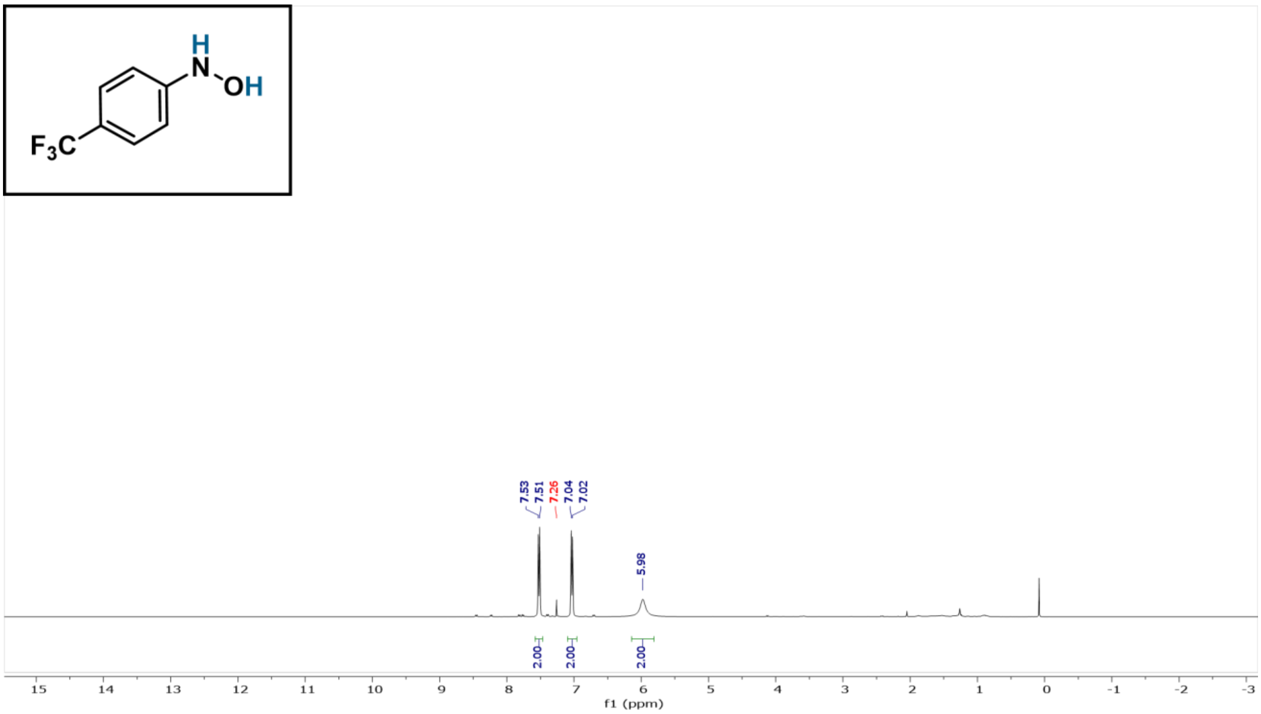

Figure S185 – ^{1}H NMR (400 MHz, $CDCl_3$, 296 K) spectrum of compound **3f**.

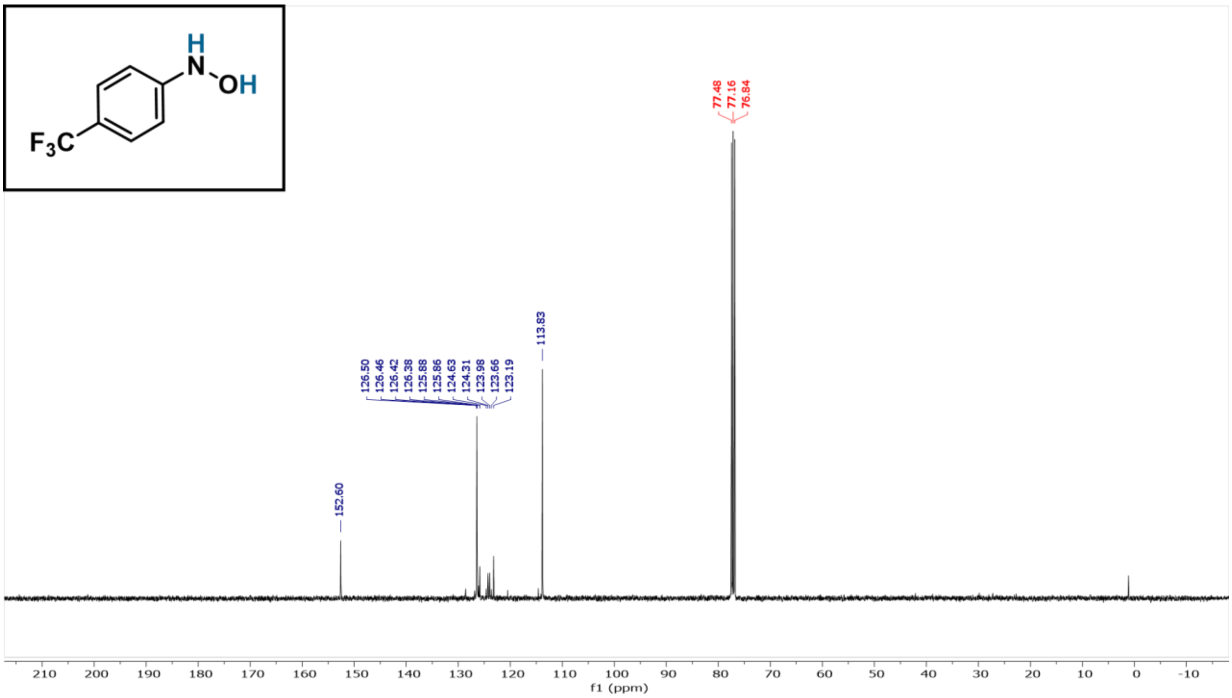

Figure S186 – $^{13}C\{^1H\}$ NMR (101 MHz, $CDCl_3$, 296 K) spectrum of compound **3f**.

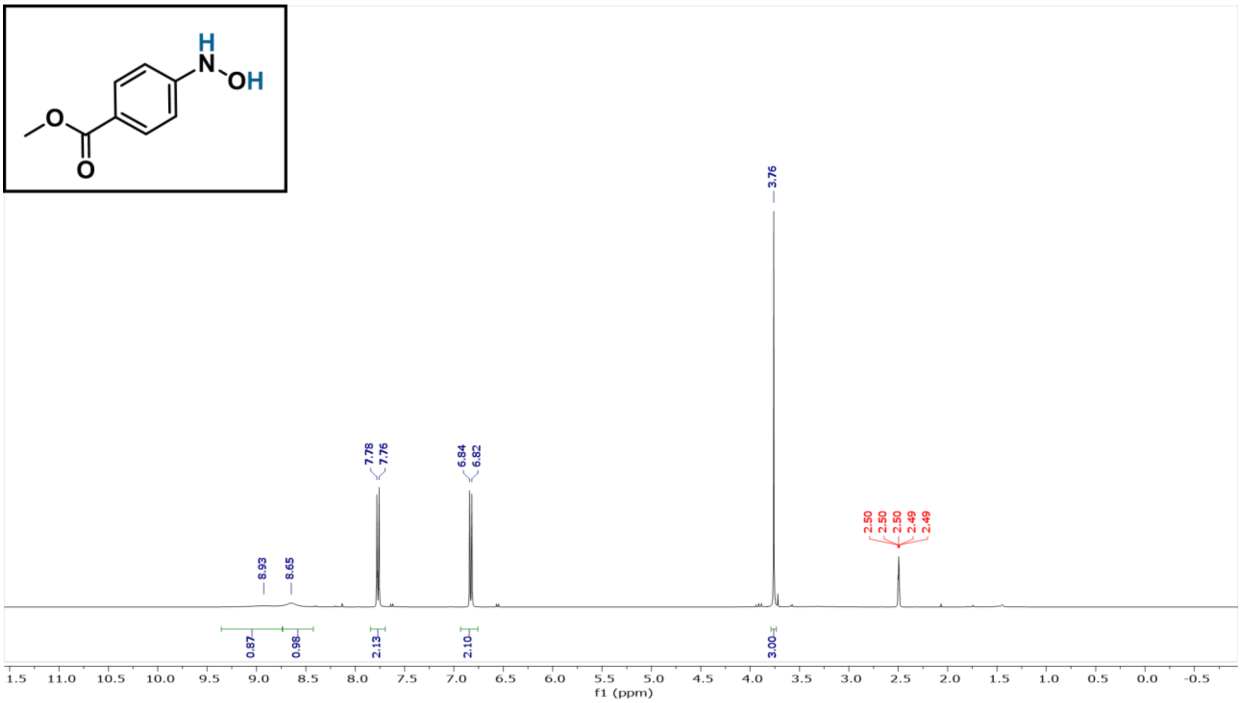

Figure S187 – ^1H NMR (400 MHz, DMSO-d_6, 296 K) spectrum of compound **3g**.

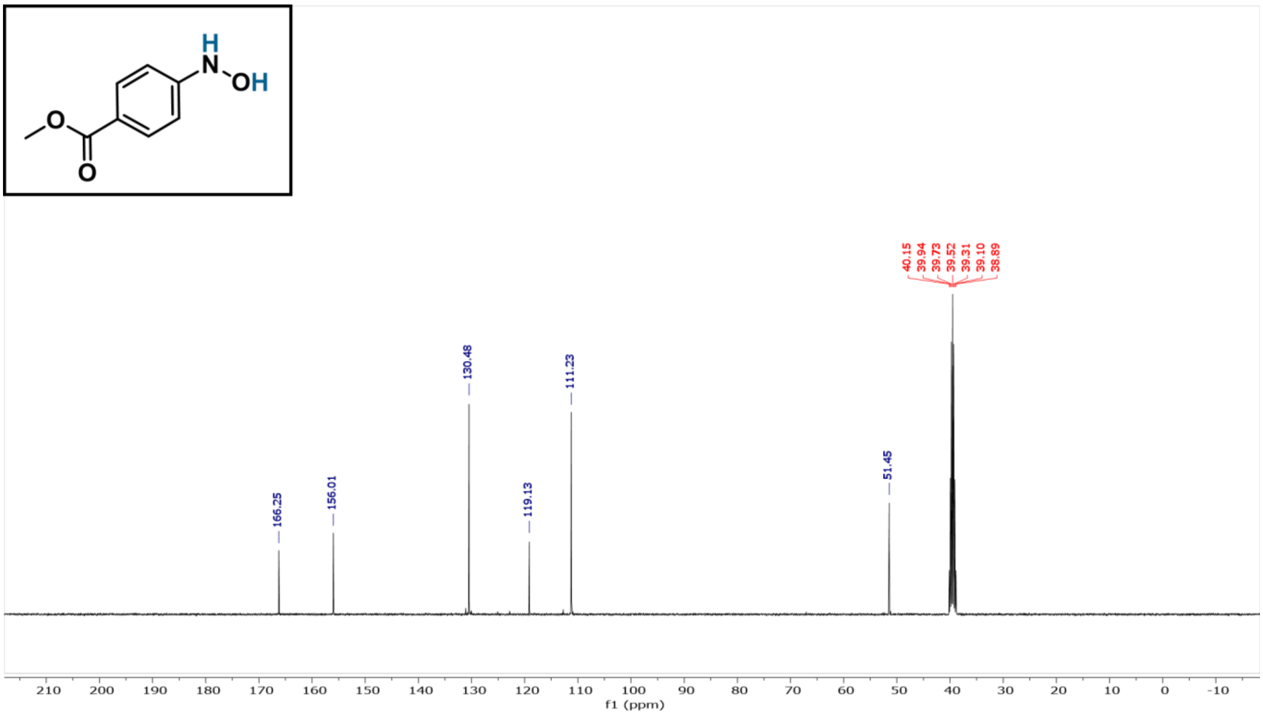

Figure S188 – $^{13}C\{^{1}H\}$ NMR (101 MHz, DMSO-d_6, 296 K) spectrum of compound **3g**.

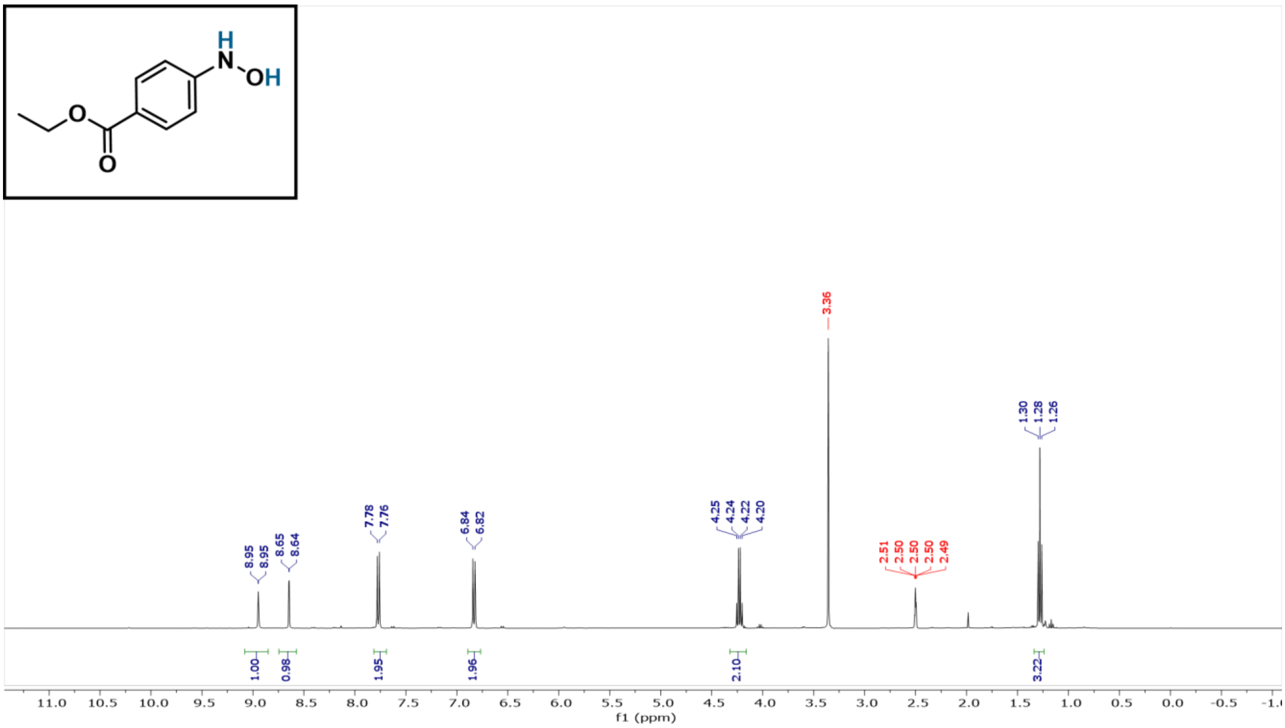

Figure S189 – ^{1}H NMR (400 MHz, DMSO-d_6, 296 K) spectrum of compound **3h**.

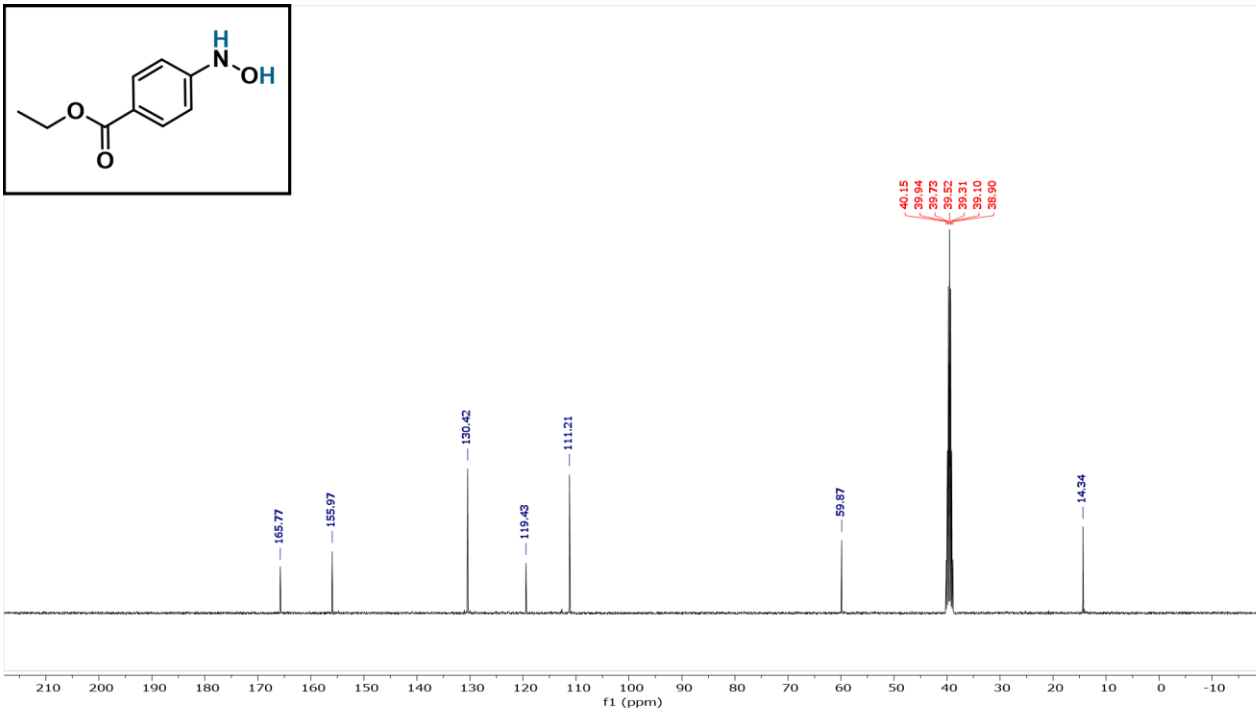

Figure S190 – $^{13}C\{^{1}H\}$ NMR (101 MHz, DMSO-d_6, 296 K) spectrum of compound **3h**.

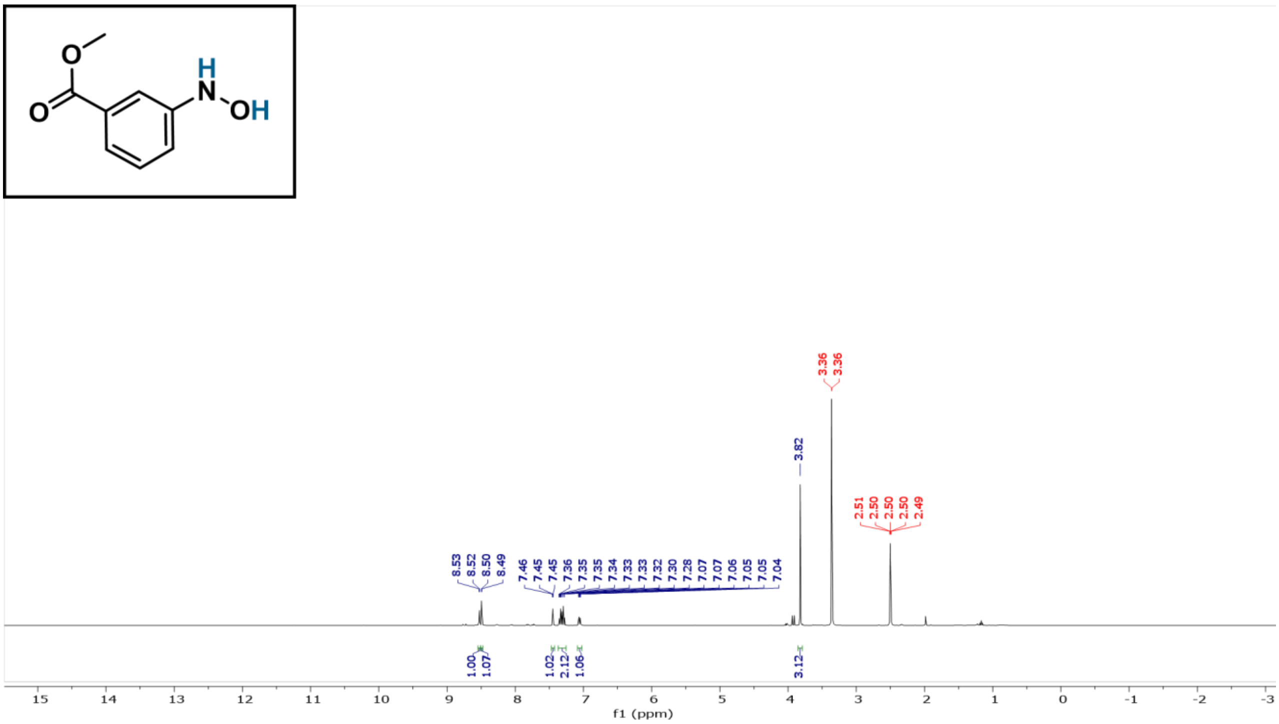

Figure S191 – ^{1}H NMR (400 MHz, DMSO-d_6, 296 K) spectrum of compound **3i**.

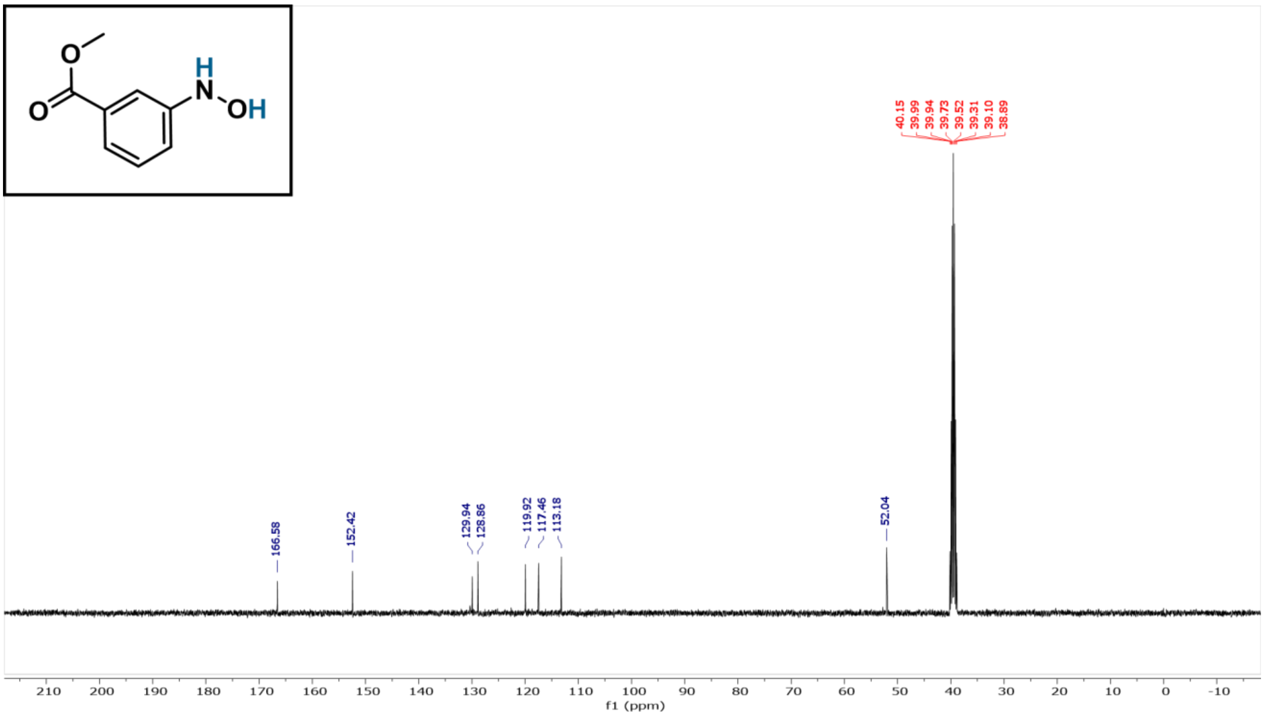

Figure S192 – $^{13}C\{^{1}H\}$ NMR (101 MHz, DMSO-d_6, 296 K) spectrum of compound **3i**.

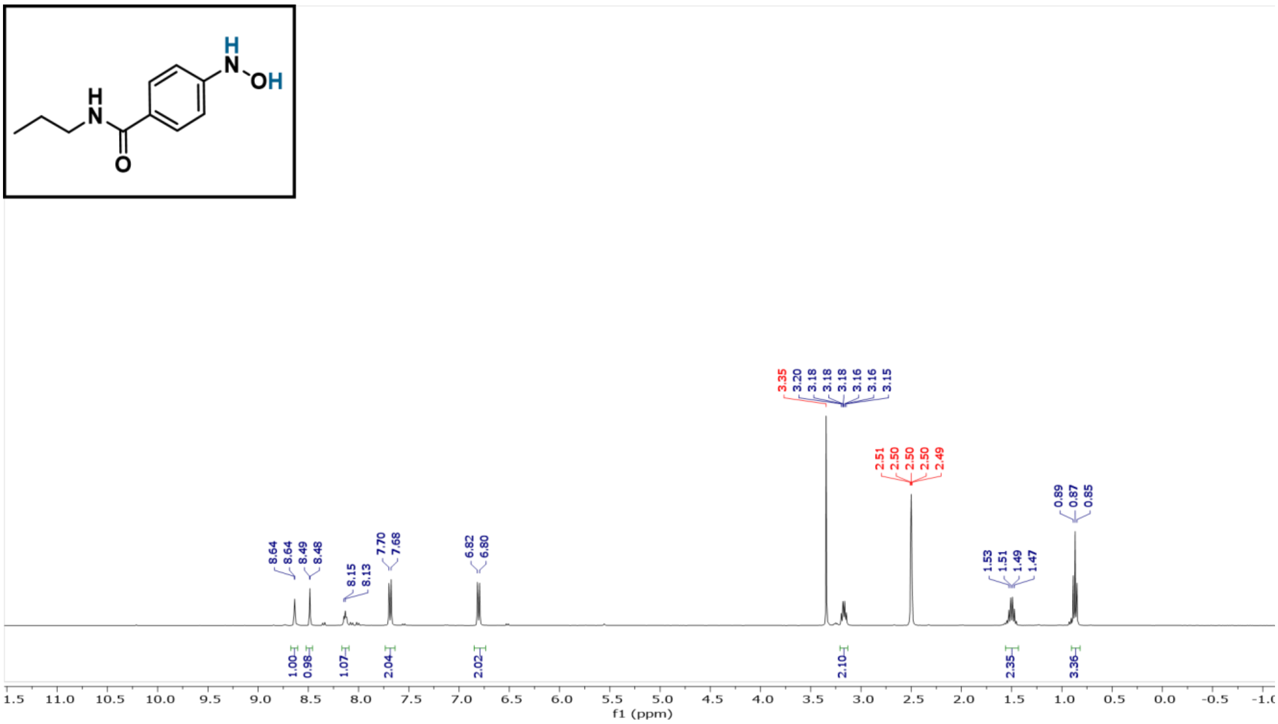

Figure S193 – ^{1}H NMR (400 MHz, DMSO-d_6, 296 K) spectrum of compound **3j**.

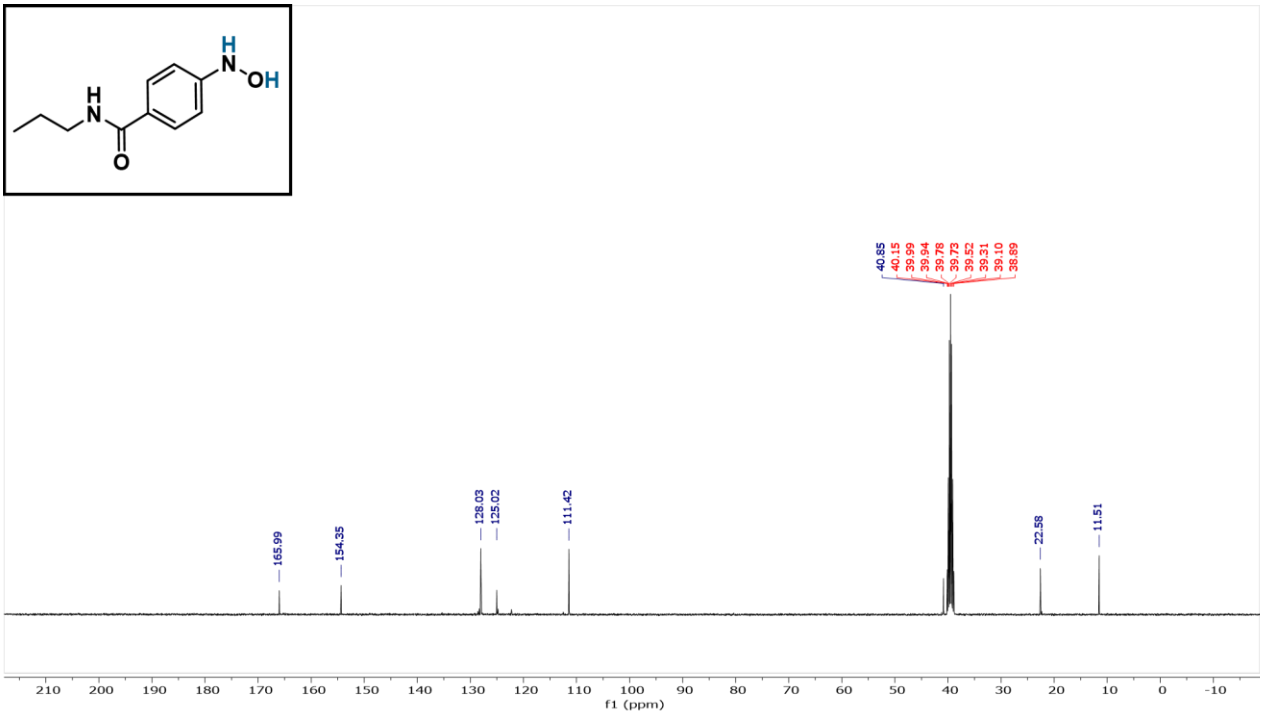

Figure S194 – $^{13}C\{^{1}H\}$ NMR (101 MHz, DMSO-d_6, 296 K) spectrum of compound **3j**.

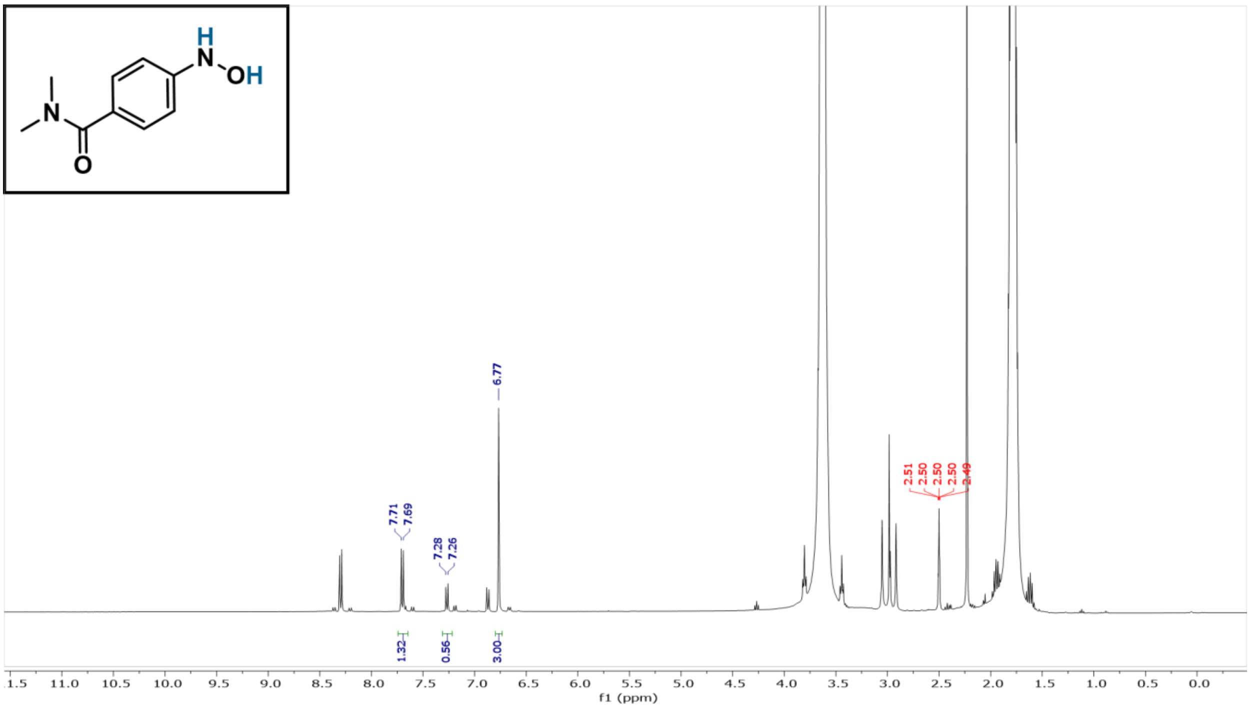

Figure S195 – ^{1}H NMR (400 MHz, DMSO-d_6, 296 K) crude spectrum of compound **3k**.

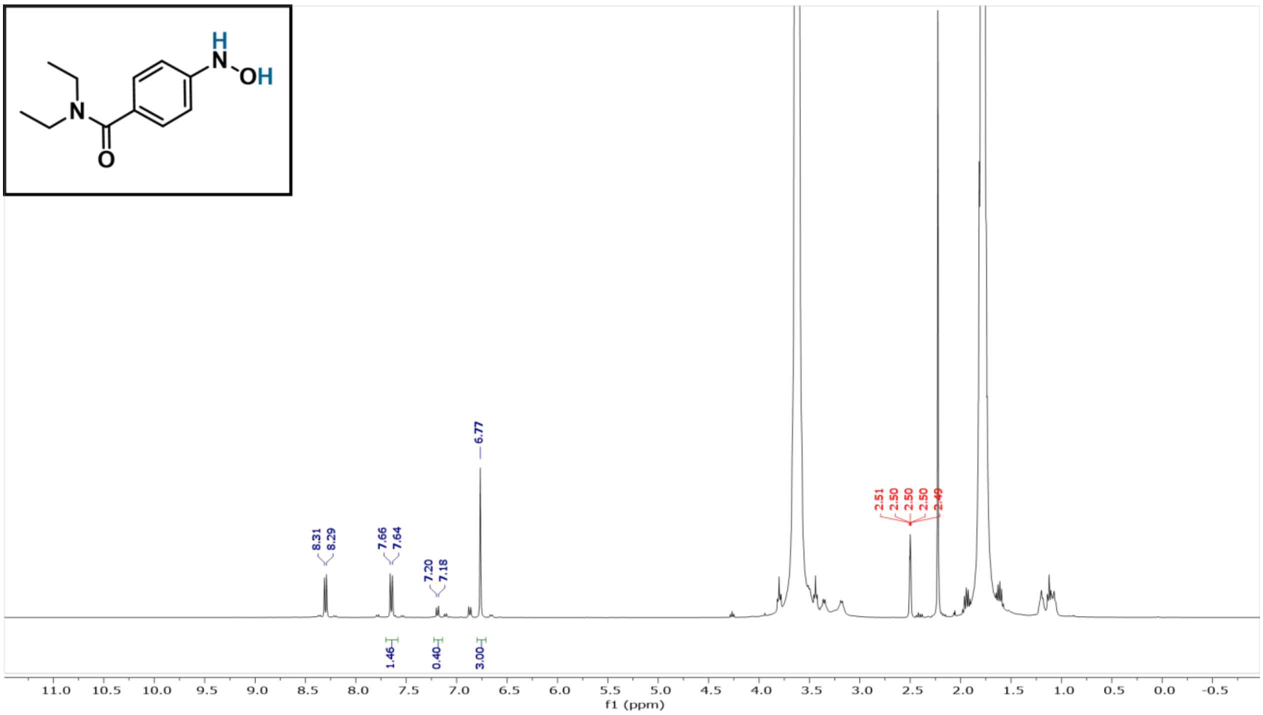

Figure S196 – ¹H NMR (400 MHz, DMSO-*d*₆, 296 K) crude spectrum of compound **3l**.

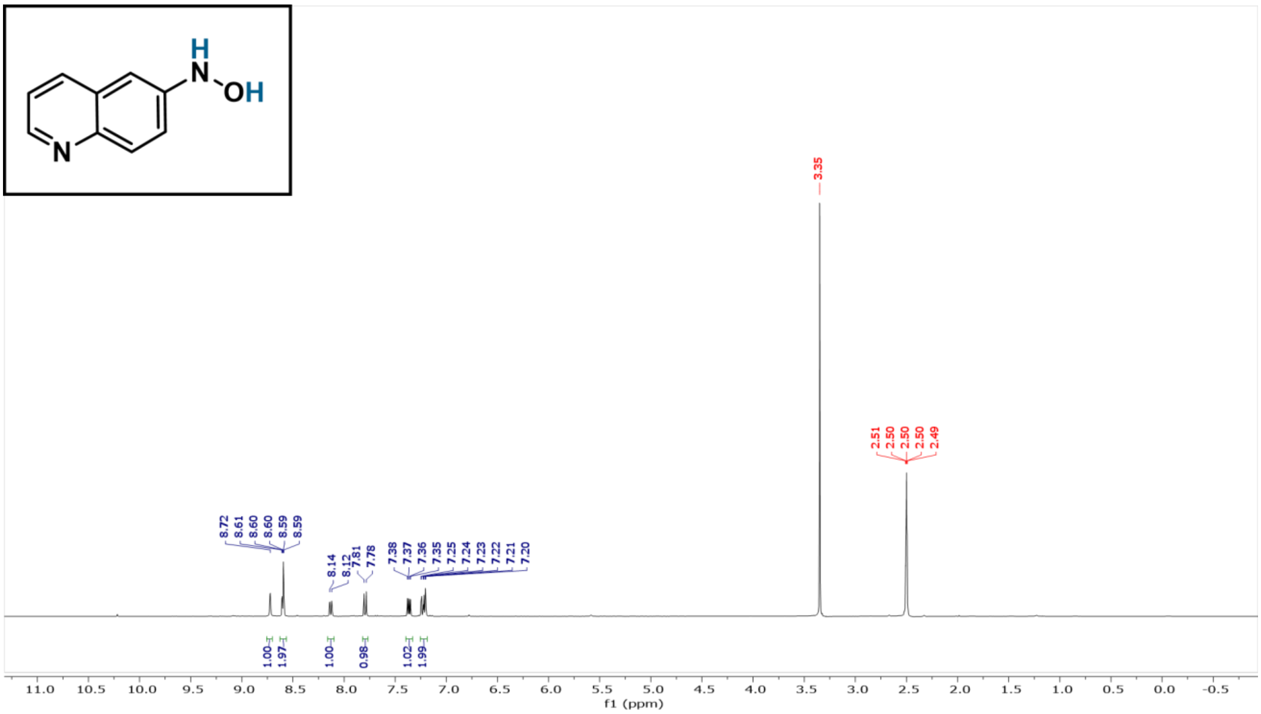

Figure S197 – ¹H NMR (400 MHz, DMSO-*d*₆, 296 K) spectrum of compound **3m**.

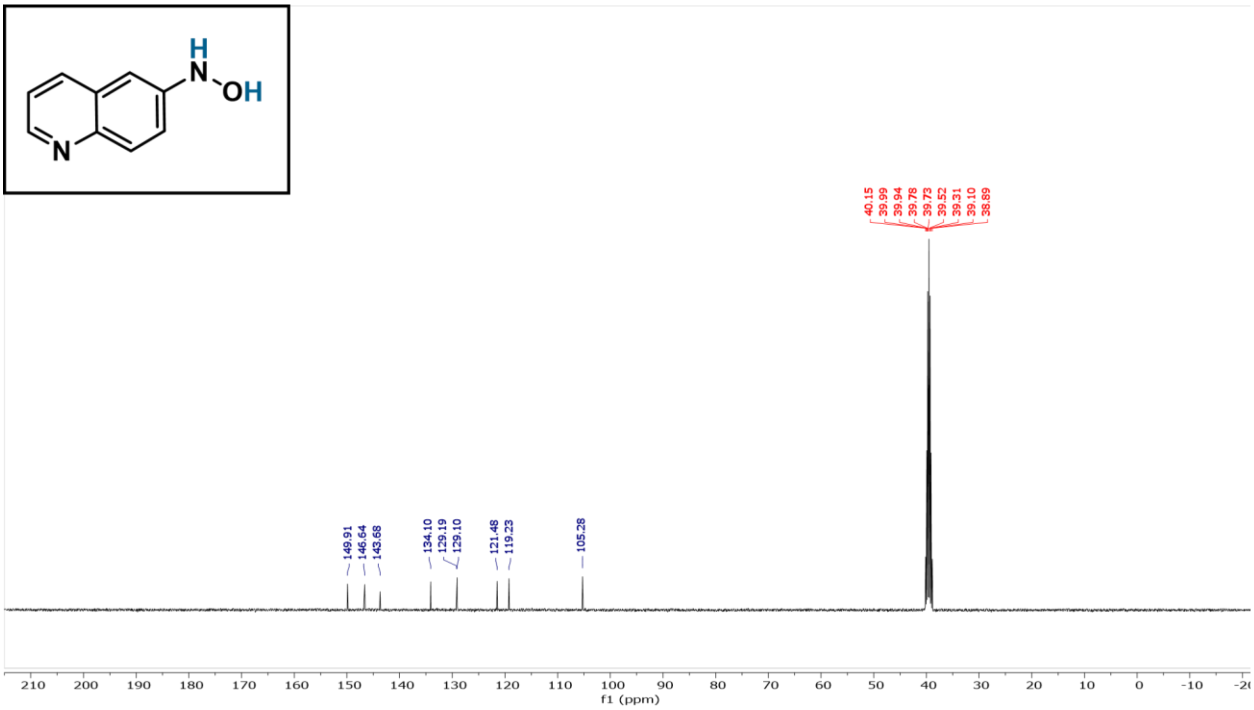

Figure S198 – $^{13}C\{^1H\}$ NMR (101 MHz, DMSO-*d*$_6$, 296 K) spectrum of compound **3m**.

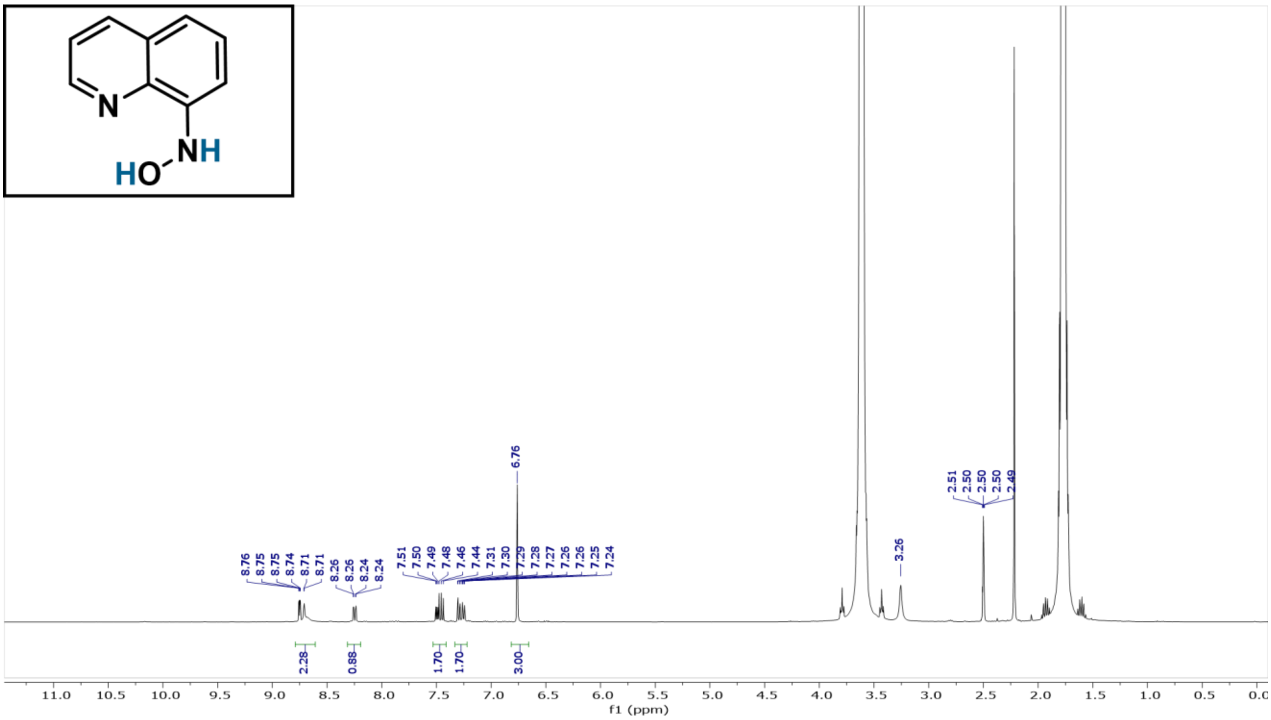

Figure S199 – ^1H NMR (400 MHz, DMSO-*d*$_6$, 296 K) crude spectrum of compound **3n**.

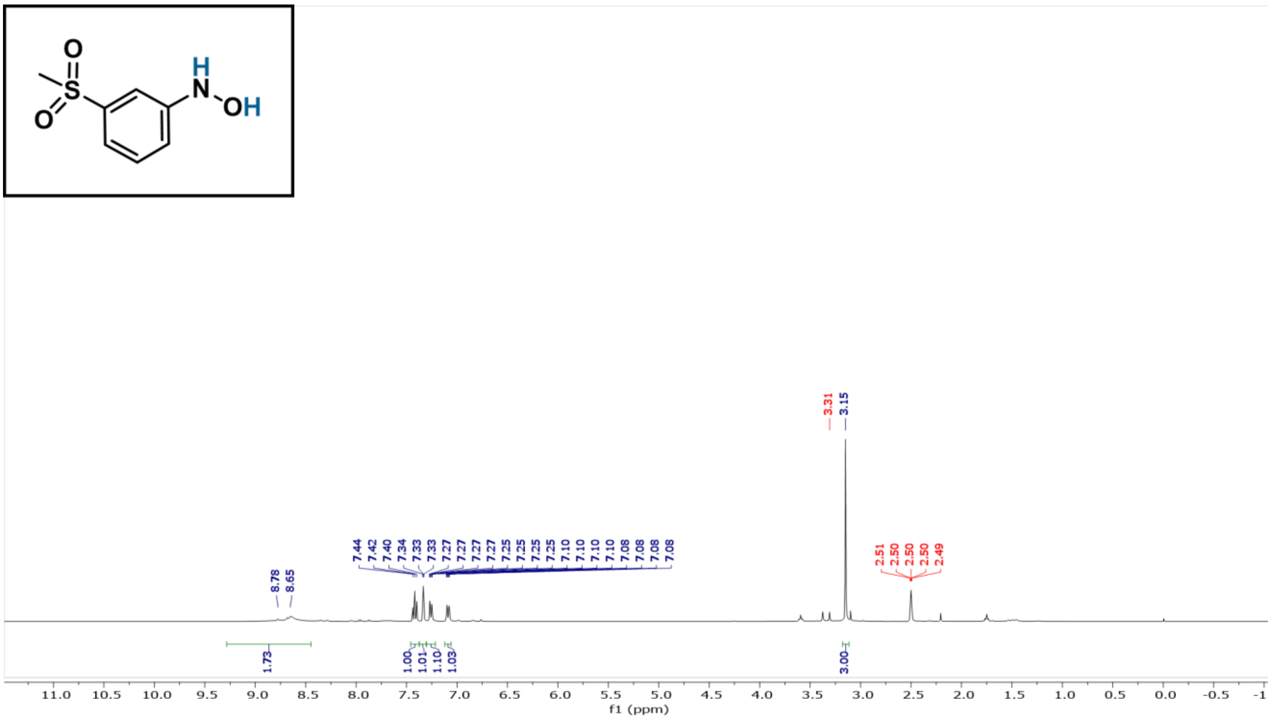

Figure S200 – ^{1}H NMR (400 MHz, DMSO-d_6, 296 K) spectrum of compound **3o**.

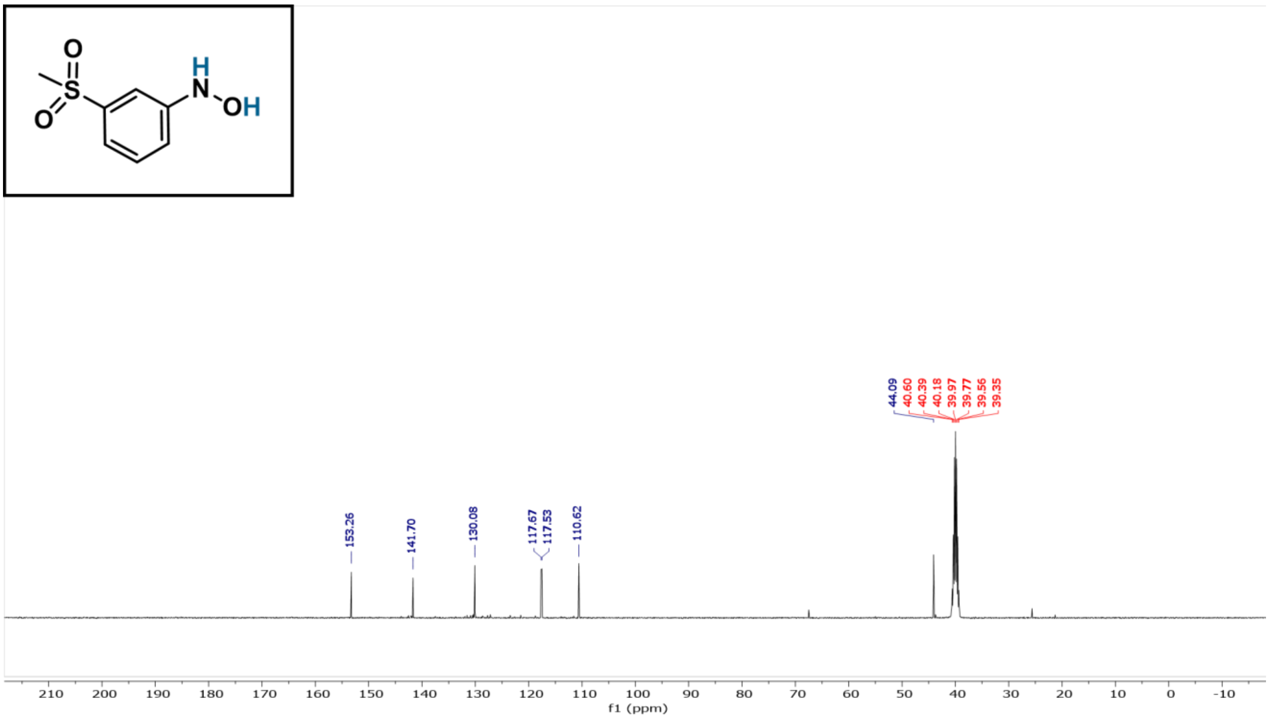

Figure S201 – ^{13}C{^{1}H} NMR (101 MHz, DMSO-d_6, 296 K) spectrum of compound **3o**.

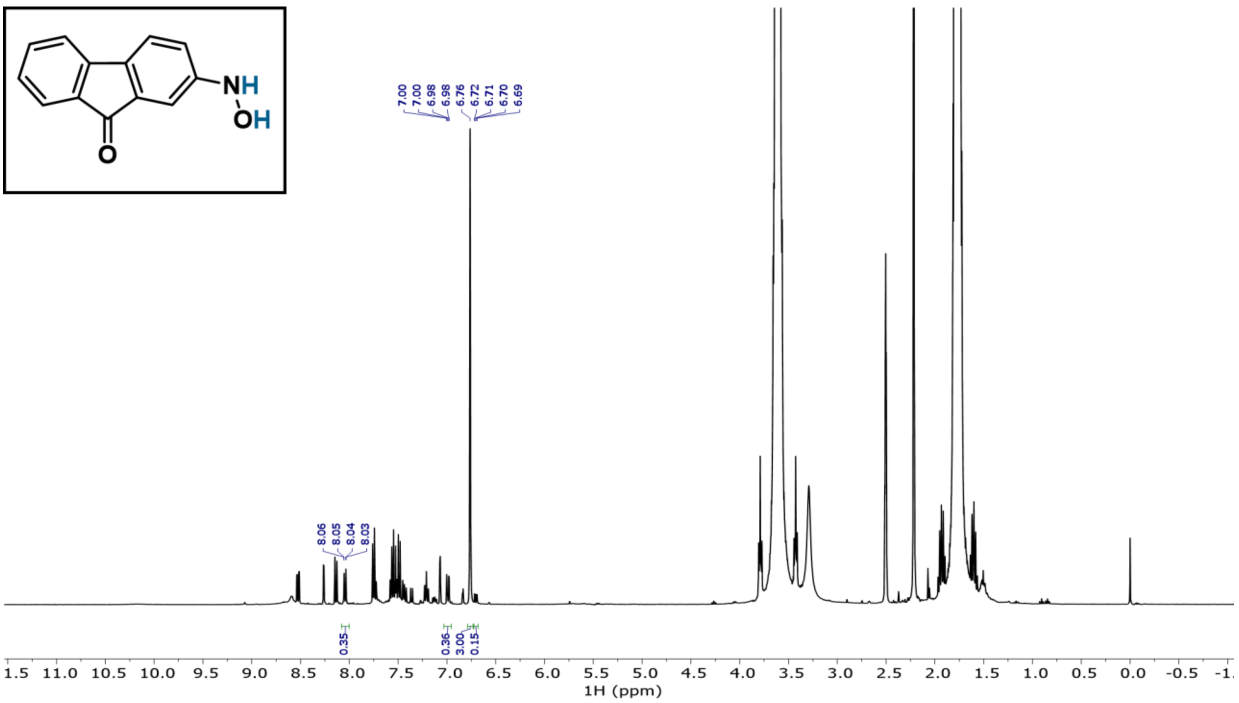

Figure S202 – ^{1}H NMR (400 MHz, CDCl$_3$, 296 K) crude spectrum of compound **3y**.

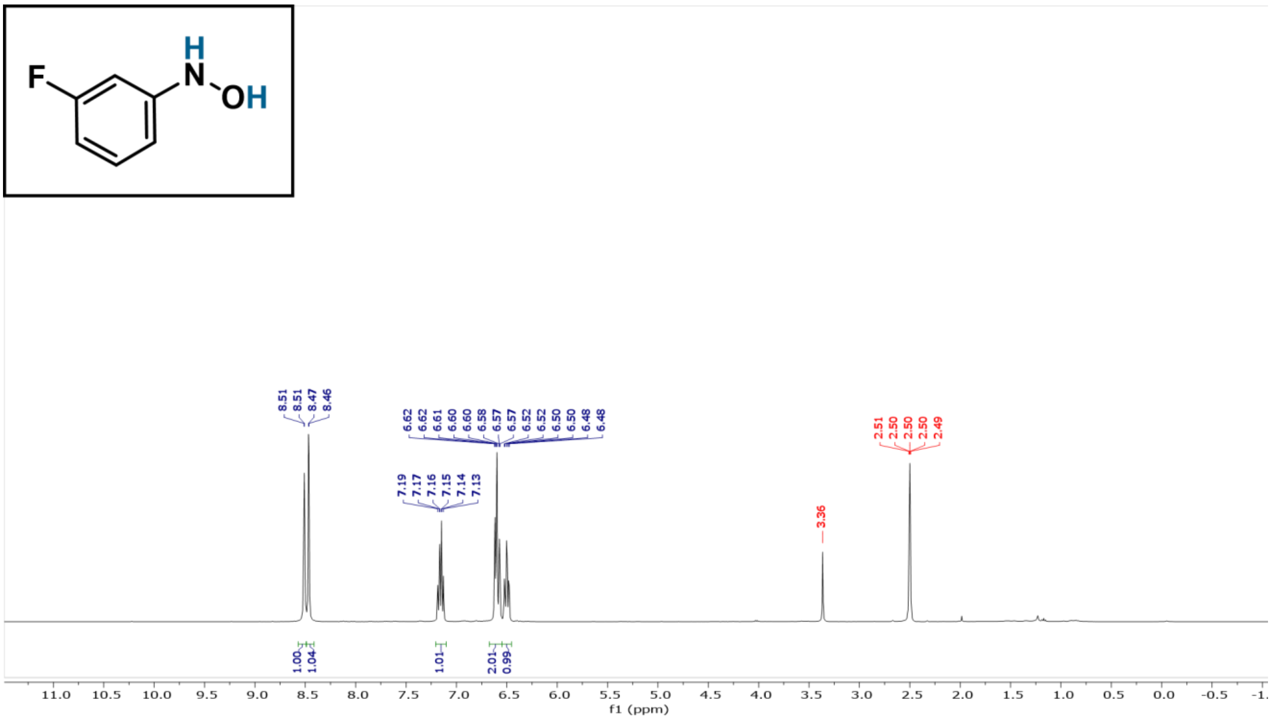

Figure S203 – ^{1}H NMR (400 MHz, DMSO-*d*$_6$, 296 K) spectrum of compound **3ac**.

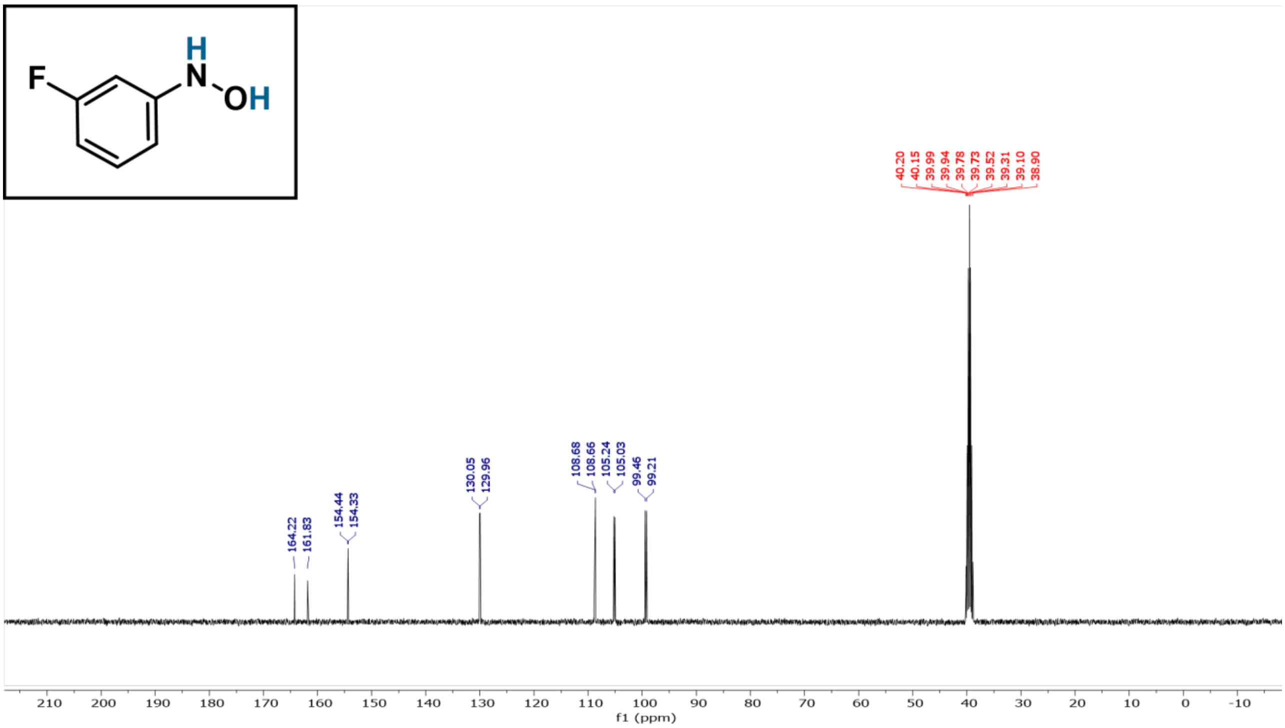

Figure S204 – $^{13}C\{^1H\}$ NMR (101 MHz, DMSO-d_6, 296 K) spectrum of compound **3ac**.

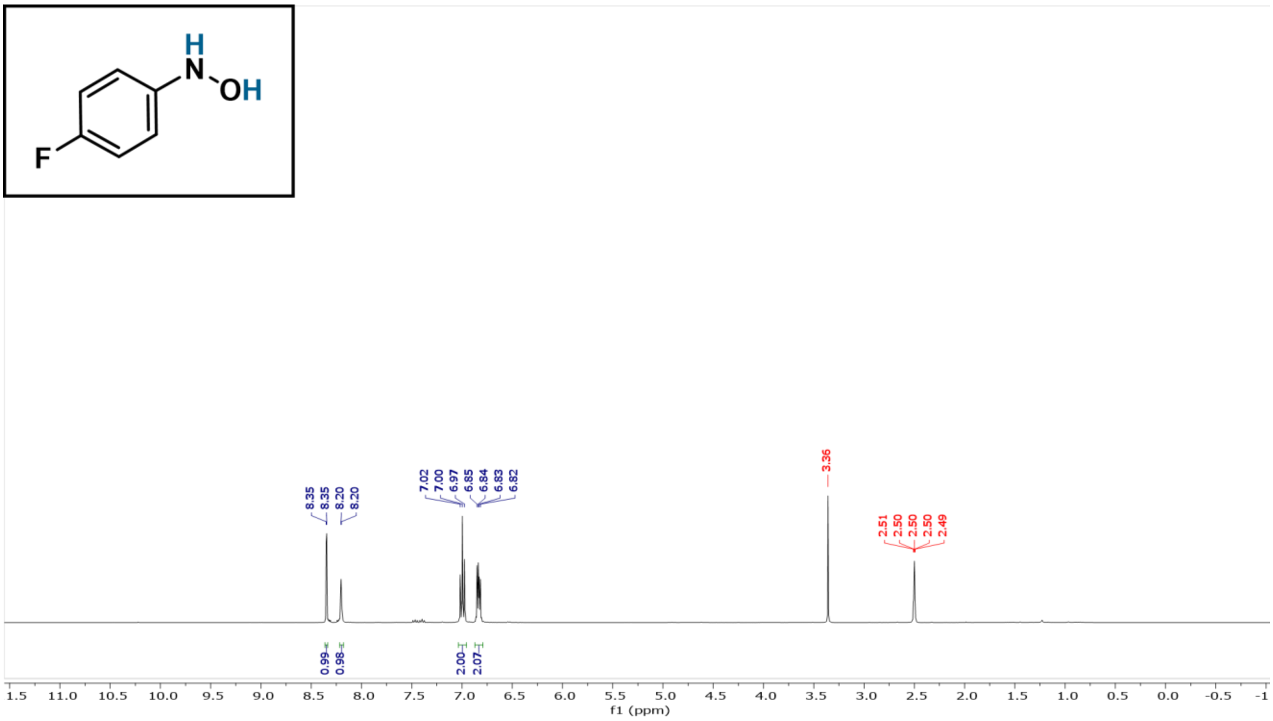

Figure S205 – ^{1}H NMR (400 MHz, DMSO-d_6, 296 K) spectrum of compound **3ad**.

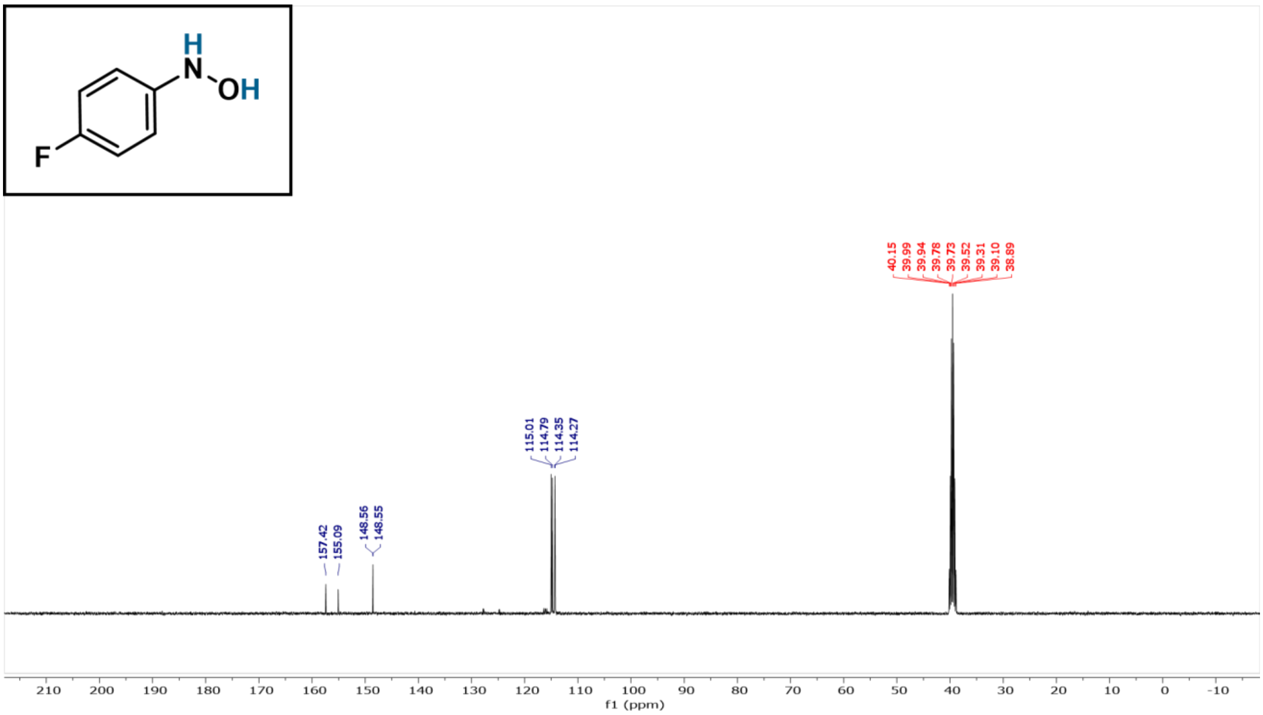

Figure S206 – $^{13}C\{^{1}H\}$ NMR (101 MHz, DMSO-d_6, 296 K) spectrum of compound **3ad**.

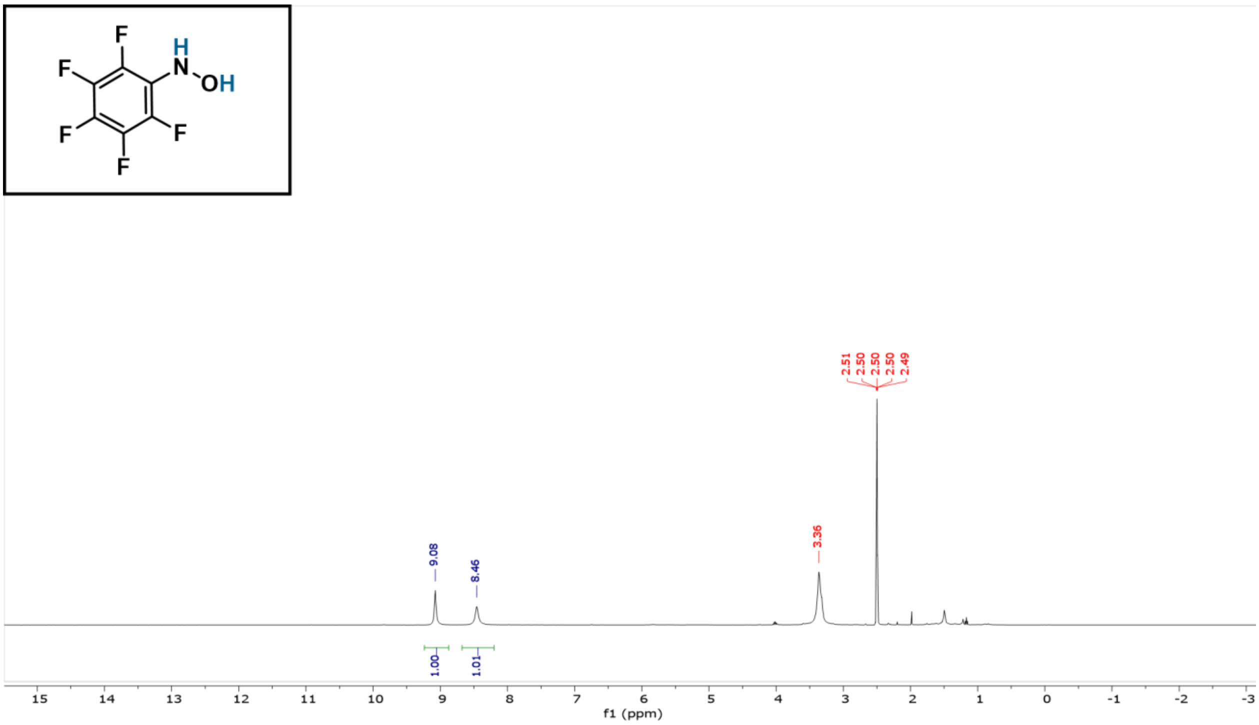

Figure S207 – ^{1}H NMR (400 MHz, DMSO-d_6, 296 K) spectrum of compound **3ae**.

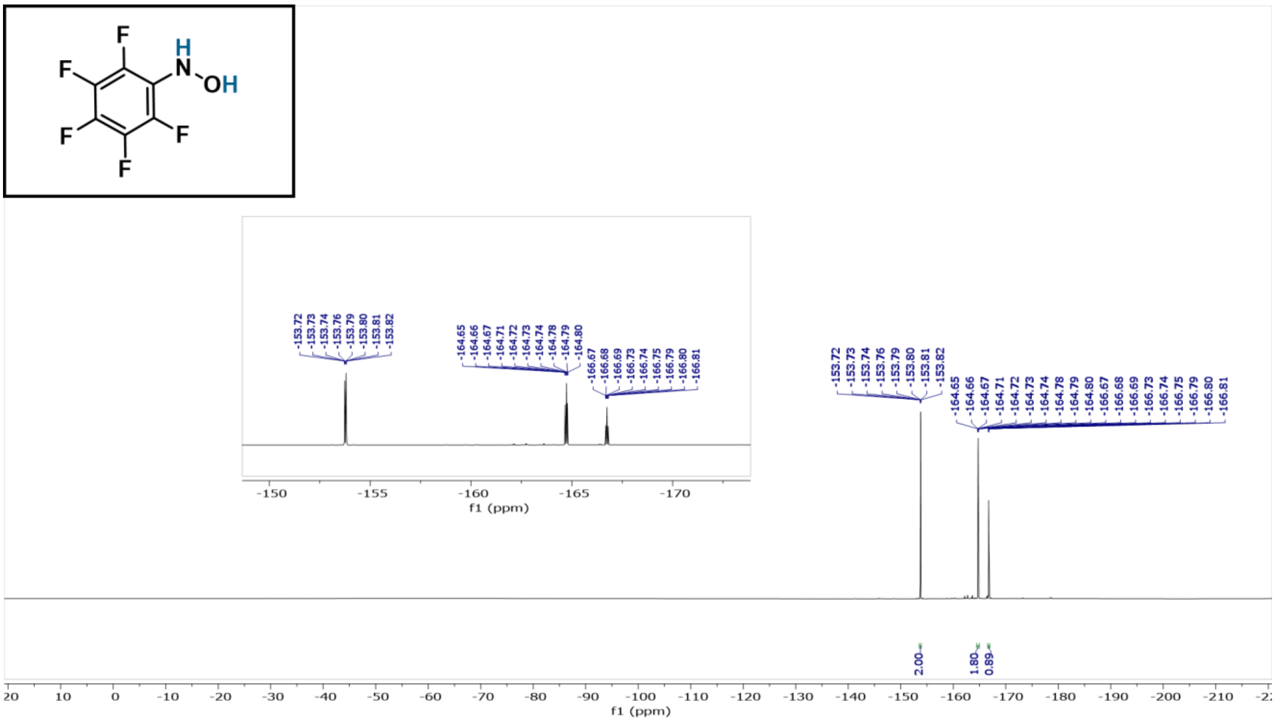

Figure S208 – ^{19}F NMR (376 MHz, DMSO-d_6, 296 K) spectrum of compound **3ae**.

Quantum Chemical Calculations

General Procedure

Calculations were carried out with the ORCA program package[32] without any truncations of the ligand. The Def2-TZVP basis set was applied for Rh, P, N, and B in combination with the Def2-SVP basis set for all other atoms.[33] Implicit solvation models of either toluene (ε = 2.4) or THF (ε = 7.25) using the CPCM model were applied,[34] while the relaxed surface scans were conducted without a solvation model. Geometry optimizations, frequency calculations, and relaxed surface scans were performed using the BP86 functional,[35] and the RI approximation was used to speed up the calculations with the auxiliary basis set def2/J.[36] Harmonic frequency calculations provided zero-point energies, thermal corrections, and entropic corrections. The final single-point energies of the optimized structures were calculated on the B3LYP-D3BJ level of theory[37] using the RIJCOSX approximation[38] with the auxiliary basis set def2/J[36] and Grimme's atom-pairwise dispersion correction with Becke-Johnson damping (D3BJ).[39] The transition state optimization for **TS1** was performed with geometric constraints for the cartesian coordinates of the atoms Rh1 and N60, and the subsequent frequency calculation confirmed the stationary point as a transition state by identifying a single imaginary frequency (Table S11). A relaxed surface scan by scanning the O1-B2 bond distance indicated the connectivity of **TS1** to **I1** and **I2** (Figure S210).

Key bond lengths and angles compared to the molecular structure of **($1{\cdot}CH_3CN$)** obtained by XRD (Table S10) show that the structure reproduces the geometrical features within the expected uncertainty. The relative Gibbs free energies were additionally calculated with implicit solvation of THF, revealing only minor differences to toluene solvation (Table S12).

Key Bond Lengths and Angles of Selected Structures

Table S10: Optimized structure of [**23_Rh_ACN_B_ACN**]$^{2+}$ (left) and key bond lengths and angles of the optimized structure of **1·CH$_3$CN** (BP86/Def2-TZVP (Rh, N, P, B), Def2-SVP, CPCM (toluene)) compared to the molecular structure determined by XRD (right). H atoms are omitted for clarity.

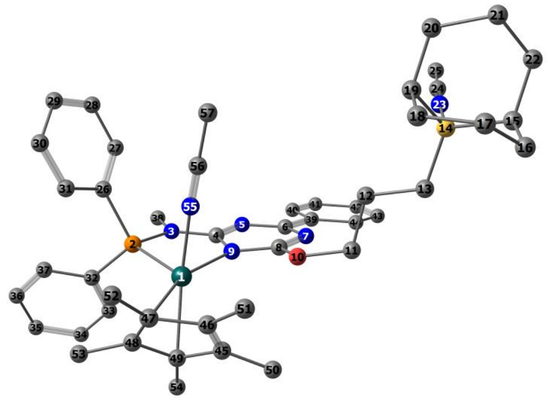

	DFT	XRD
Rh1-N9	2.14 Å	2.12 Å
Rh1-P2	2.31 Å	2.29 Å
Rh1-Cp*	1.87 Å	1.83 Å
Rh1-N55	2.06 Å	2.08 Å
B14-N23	1.56 Å	1.62 Å
P2-Rh1-N9	79.6°	78.7°

Table S11: Key bond lengths, angles, and imaginary frequency ν of **TS1** (BP86/Def2-TZVP (Rh, N, P, B), Def2-SVP, CPCM (toluene)). Selected H atoms are omitted for clarity.

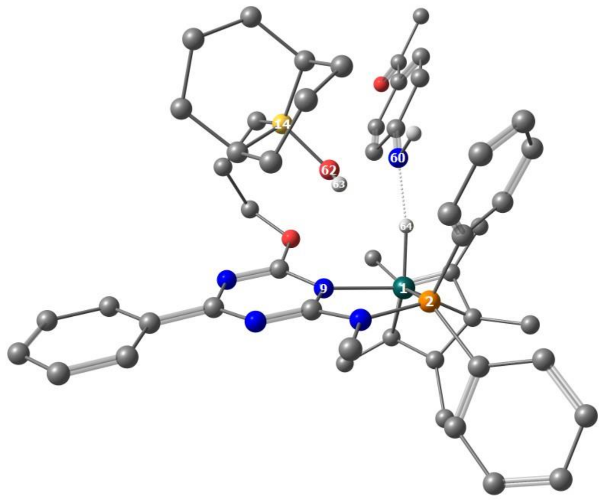

Rh1-P2	2.19 Å
Rh1-N9	2.09 Å
Rh1-H64	1.57 Å
Rh1-Cp*	1.92 Å
N60-H64	1.68 Å
N60-O62	1.76 Å
O62-H63	0.98 Å
O62-B14	1.54 Å
Rh1-H64-N60	171.3°
H64-N60-O62	89.6°

ν = -359.0 cm^{-1}

Relaxed Surface Scans

- Interaction of Hydroxylamine and **I1** Without Borane

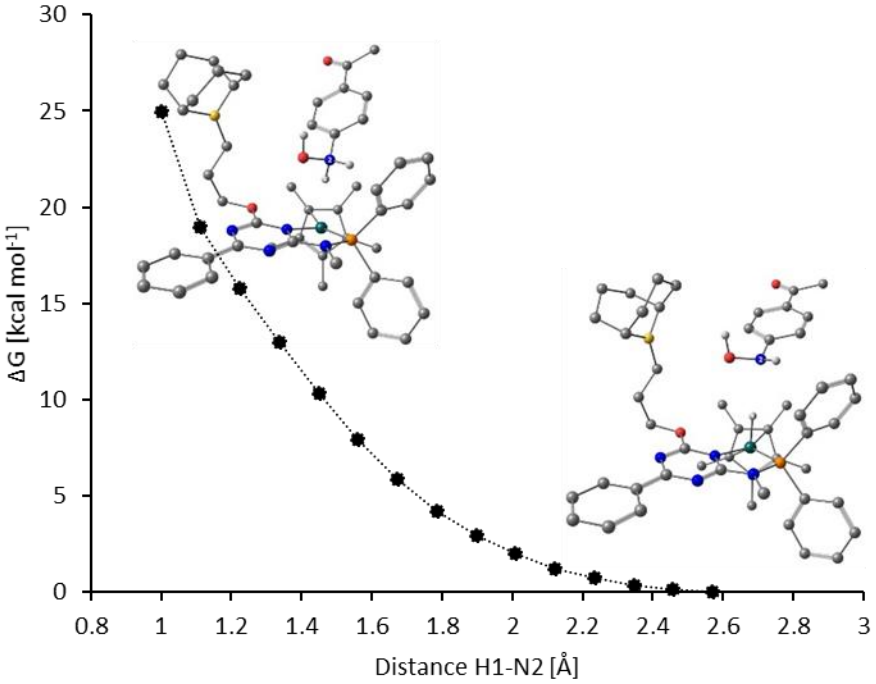

Figure S209: Relaxed surface scan varying the H1-N2 distance between **I1** and hydroxylamine from 2.6 Å to 1.0 Å. The N-O distance changes from 1.41 to 1.43 Å.

- Concerted N–O Bond Cleavage and Hydride Transfer *via* **TS1** by the Interaction of Hydroxylamine and **I1**

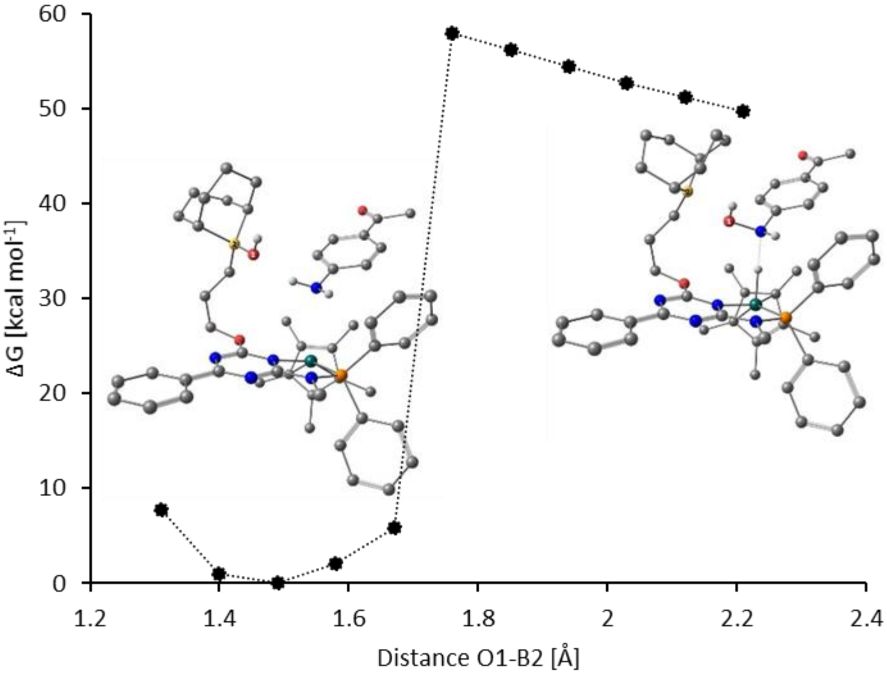

Figure S210: Relaxed surface scan varying the O1-B2 distance between **I1** and hydroxylamine from 2.2 Å to 1.3 Å.

- H_2 Cleavage Facilitated by **I2** Yielding **I1-H$_2$O**

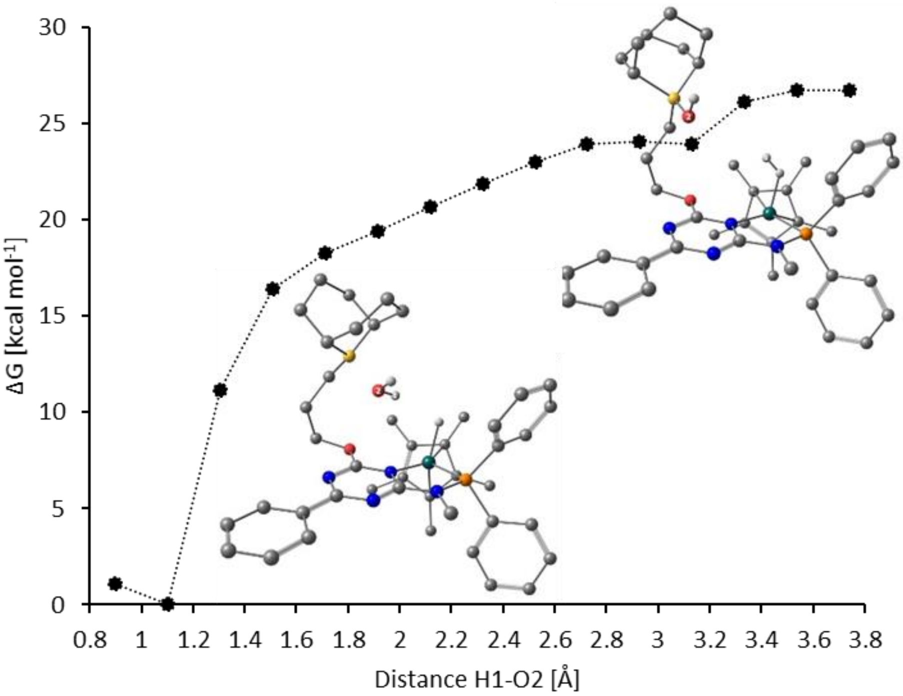

Figure S211: Relaxed surface scan varying the H1-O2 distance between **I2** and H_2 from 3.7 Å and 0.9 Å.

1. Gibbs Free Energies with Implicit Solvation of Toluene and THF

Table S12: Relative Gibbs free energies of selected intermediates calculated with implicit solvation of toluene and THF.

	**ΔG (Toluene)**	**ΔG (THF)**
[**23**_RhH_B]$^+$ (**I1**)	0.0 kcal mol^{-1}	0.0 kcal mol^{-1}
[**23**_RhH_B_ACN]$^+$	1.5 kcal mol^{-1}	3.7 kcal mol^{-1}
[**23**_RhH_B_THF]$^+$	-1.2 kcal mol^{-1}	-1.5 kcal mol^{-1}
[**23**_RhH_B_**3a**]$^+$ (coordination *via* NHOH/N)	6.6 kcal mol^{-1}	8.1 kcal mol^{-1}
[**23**_RhH_B_**3a**]$^+$ (coordination *via* NHOH/O)	11.0 kcal mol^{-1}	11.9 kcal mol^{-1}

2. Electronic Energies and Corrections

Table S13: Solvent-corrected electronic energies ($E_{el/sol}$, toluene), non-thermal ZPE corrections (E_{ZPE}), thermal energy correction (E_{therm}), enthalpy correction (H_{therm}), and entropy correction (TS). All energies are given in Hartree.

**Structure**	**$E_{el/sol}$ (toluene)**	**E_{ZPE}**	**E_{therm}**	**H_{therm}**	**-TS**
[**23**_RhH_B]$^{+}$ (**I1**)	-2439.858643	0.85876931	0.05269546	0.00094421	0.13229
**TS1**	-2954.70738	1.01809119	0.06170127	0.00094421	0.146068
[**23**_Rh_BOH]$^{+}$ (**I2**)	-2514.969839	0.86596555	0.05350305	0.00094421	0.131854
[**23**_Rh_ACN_B_ACN]$^{2+}$	-2704.411769	0.94450721	0.06094986	0.00094421	0.147382
[**23**_RhH_B_ACN]$^{+}$	-2572.532064	0.90579909	0.05659118	0.00094421	0.139444
[**23**_RhH_B_THF]$^{+}$	-2672.037118	0.97528395	0.05701336	0.00094421	0.139601
[**23**_RhH_B_Et_2O]$^{+}$	-2673.237326	0.99453001	0.05991008	0.00094421	0.145437
[**23**_RhH_B_H_2O]$^{+}$	-2516.214624	0.88484096	0.05427072	0.00094421	0.134363
[**23**_RhH_B_CH_2Cl_2]$^{+}$	-3399.134604	0.88775139	0.05862709	0.00094421	0.145633
[**23**_RhH_B_EtOH]$^{+}$	-2594.727829	0.93994229	0.05715528	0.00094421	0.1403226
[**23**_RhH_B_MeOH]$^{+}$	-2555.461064	0.91242716	0.05482346	0.00094421	0.1349070
[**23**_RhH_B_**3a**]$^{+}$ (coordination *via* NHOH/N)	-2954.729463	1.016189	0.06364604	0.00094421	0.153155
[**23**_RhH_B_**3a**]$^{+}$ (coordination *via* NHOH/O)	-2954.72742	1.01827599	0.06225004	0.00094421	0.148837
[**23**_RhH_B_**2a**]$^{+}$ (coordination *via* NH_2)	-2879.657544	1.01262584	0.06249732	0.00094421	0.151846
[**23**_RhH_B_**3e**]$^{+}$ (coordination *via* NHOH)	-2894.463685	0.97888474	0.0618584	0.00094421	0.149582
[**23**_RhH_B_(*N*-(4-propylphenyl)-hydroxylamine)]$^{+}$	-2920.054009	1.0622799	0.06439736	0.00094421	0.154538
[**23**_RhH_B_**3e**]$^{+}$ (coordination *via* nitrile)	-2894.470717	0.97772146	0.06231192	0.00094421	0.151666
[**23**_RhH_B_**3a**]$^{+}$ (coordination *via* ketone)	-2954.737935	1.01488004	0.06411893	0.00094421	0.154227

N-(4-propylphenyl)-hydroxylamine	-480.1704622	0.1993165	0.01151662	0.00094421	0.048969
H_2O	-76.32697552	0.02058749	0.00283582	0.00094421	0.021476
H_2	-1.167497707	0.00974129	0.00236045	0.00094421	0.014853
ACN	-132.6516256	0.0436453	0.00365764	0.00094421	0.02867
THF	-232.1487655	0.11277311	0.00416556	0.00094421	0.032103
CH_2Cl_2	-959.255157	0.02823369	0.00363963	0.00094421	0.030839
Et_2O	-233.3530317	0.13182525	0.00694012	0.00094421	0.037827
EtOH	-154.8383882	0.07718057	0.00436404	0.00094421	0.03076071
MeOH	-115.5723156	0.04947279	0.00333801	0.00094421	0.02705121
**2a**	-439.7783721	0.1495305	0.00956409	0.00094421	0.044505
**3a**	-514.8519229	0.15360428	0.01062532	0.00094421	0.047095
**3e**	-454.588678	0.11635693	0.0088191	0.00094421	0.043229

Table S14: Solvent-corrected electronic energies ($E_{el/sol}$, THF), non-thermal ZPE corrections (E_{ZPE}), thermal energy correction (E_{therm}), enthalpy correction (H_{therm}), and entropy correction (TS). All energies are given in Hartree.

**Structure**	**$E_{el/sol}$ (THF)**	**E_{ZPE}**	**E_{therm}**	**H_{therm}**	**-TS**
[**23**_RhH_B]$^{+}$ (**I1**)	-2439.875715	0.85801972	0.05274326	0.00094421	0.13238092
[**23**_Rh_ACN_B_ACN]$^{2+}$	-2704.469664	0.94393404	0.06009166	0.00094421	0.14605393
[**23**_RhH_BACN]$^{+}$	-2572.548617	0.90484009	0.05575149	0.00094421	0.1378419
[**23**_RhH_BTHF]$^{+}$	-2672.055068	0.97468044	0.05783348	0.00094421	0.14152075
[**23**_RhH_B_**3a**]$^{+}$ (coordination *via* NHOH/N)	-2954.751637	1.01568519	0.06266796	0.00094421	0.15019387
[**23**_RhH_B_**3a**]$^{+}$ (coordination *via* NHOH/O)	-2954.751156	1.01807636	0.06318704	0.00094421	0.15144693
ACN	-132.655017	0.04360222	0.00364968	0.00094421	0.0276161
H_2O	-76.32973433	0.02054058	0.0028359	0.00094421	0.02147822
H_2	-1.167597499	0.00973422	0.00236045	0.00094421	0.01485395
**2a**	-439.7831162	0.14939262	0.00958685	0.00094421	0.04455603
**3a**	-514.8569659	0.15341984	0.010659	0.00094421	0.04714794
THF	-232.1500598	0.1126837	0.0041714	0.00094421	0.0321127

3. XYZ Coordinates of the Optimized Structures | Toluene

- **[23_RhH_B]$^{+}$ (I1)**

Rh	7.695808000	5.901767000	12.583521000
P	7.121618000	4.878847000	10.686989000
N	8.111815000	3.453250000	10.695774000
C	8.972008000	3.265963000	11.742739000
N	9.721687000	2.157095000	11.746133000
C	10.607215000	2.037241000	12.751892000
N	10.809293000	2.994694000	13.681643000
C	10.019998000	4.064376000	13.615564000
N	9.008620000	4.231725000	12.719323000
O	10.182756000	5.069037000	14.468041000
C	11.276727000	5.010531000	15.437526000
H	11.375621000	3.970437000	15.804259000
H	10.923984000	5.652364000	16.268567000
C	12.586872000	5.533492000	14.858192000
H	12.900274000	4.878147000	14.018281000
H	12.408496000	6.540976000	14.424314000
C	13.695785000	5.602925000	15.921686000
H	13.861456000	4.592788000	16.373270000
H	13.362009000	6.213467000	16.795843000
B	15.125043000	6.119433000	15.499595000
C	16.281089000	6.325816000	16.555706000
H	15.981293000	6.023197000	17.584084000
C	16.570943000	7.860714000	16.593657000
H	17.420736000	8.065293000	17.283906000
H	15.688121000	8.361868000	17.053166000
C	16.848478000	8.508974000	15.219974000
H	16.799360000	9.614762000	15.324113000
H	17.889624000	8.293787000	14.908488000
C	15.872365000	8.065188000	14.108939000
H	16.245754000	8.414494000	13.119378000
H	14.898365000	8.585432000	14.259601000
C	15.588682000	6.527949000	14.047839000
H	14.804929000	6.372646000	13.274526000
C	16.815548000	5.652318000	13.649238000
H	16.447987000	4.613419000	13.485260000
H	17.211266000	5.986188000	12.662959000
C	17.964104000	5.614232000	14.680765000
H	18.575754000	6.533841000	14.591316000
H	18.654384000	4.782324000	14.421155000
C	17.494190000	5.440744000	16.140864000
H	18.348960000	5.621479000	16.831727000
H	17.204781000	4.376434000	16.299055000
C	7.548576000	5.730398000	9.129858000
C	8.752266000	5.445650000	8.448945000
H	9.419222000	4.647996000	8.807250000
C	9.114825000	6.189940000	7.313630000
H	10.053399000	5.955761000	6.788219000
C	8.287040000	7.227590000	6.851973000
H	8.573754000	7.808234000	5.961849000
C	7.094147000	7.526092000	7.534355000
H	6.444651000	8.342332000	7.182680000
C	6.726805000	6.786119000	8.669738000
H	5.795904000	7.039357000	9.199561000
C	5.454623000	4.161892000	10.441139000
C	4.920726000	3.434066000	11.530429000
H	5.491278000	3.367110000	12.470155000
C	3.677999000	2.793515000	11.415859000
H	3.275177000	2.223360000	12.267010000
C	2.948414000	2.883165000	10.215461000
H	1.970061000	2.386259000	10.126926000
C	3.473160000	3.603016000	9.128898000
H	2.910199000	3.668251000	8.185182000
C	4.724069000	4.236480000	9.234767000
H	5.129050000	4.777652000	8.367894000
C	7.982946000	2.398045000	9.677663000
H	8.965198000	2.180438000	9.218311000
H	7.285274000	2.744055000	8.895293000
H	7.588691000	1.467251000	10.129018000
C	11.417635000	0.800355000	12.830812000
C	11.247947000	-0.228524000	11.873196000
H	10.509140000	-0.096468000	11.070298000
C	12.014275000	-1.399079000	11.949857000
H	11.876130000	-2.193711000	11.200725000
C	12.959062000	-1.559019000	12.980821000
H	13.560831000	-2.479251000	13.039093000
C	13.134136000	-0.541250000	13.936917000
H	13.872423000	-0.664136000	14.744090000
C	12.369914000	0.631243000	13.864450000
H	12.502634000	1.430195000	14.607228000
C	7.232568000	6.501605000	14.817874000
C	7.836132000	7.598259000	14.092680000
C	6.957157000	7.917154000	12.978458000
C	5.814291000	7.013369000	13.047330000
C	5.988577000	6.136069000	14.185093000
C	7.707945000	5.923510000	16.115965000
H	7.026892000	6.252767000	16.931068000
H	8.726932000	6.259507000	16.375066000
H	7.691593000	4.815754000	16.113054000
C	9.038015000	8.391278000	14.516801000
H	8.745291000	9.182989000	15.240990000
H	9.520759000	8.891483000	13.655728000

H	9.797871000	7.755995000	15.008837000
C	7.074595000	9.109573000	12.072338000
H	6.529233000	9.976671000	12.505192000
H	6.642442000	8.911061000	11.073059000
H	8.128827000	9.414565000	11.930382000
C	4.567094000	7.133274000	12.222694000
H	3.845103000	7.796763000	12.747373000
H	4.067234000	6.158651000	12.067438000
H	4.764769000	7.585866000	11.232823000
C	4.975098000	5.184004000	14.752153000
H	4.384980000	5.679635000	15.555133000
H	5.450554000	4.291736000	15.205146000
H	4.255076000	4.838456000	13.986424000
H	8.854024000	6.454545000	11.669405000

- **TS1**

Rh	7.699953000	6.548562000	12.100039000
P	7.192394000	4.613482000	11.213927000
N	8.356952000	3.536414000	11.926563000
C	9.390661000	4.115567000	12.623220000
N	10.323679000	3.322620000	13.149729000
C	11.359565000	3.947059000	13.750878000
N	11.541988000	5.283335000	13.702538000
C	10.598267000	5.994647000	13.095282000
N	9.404253000	5.486692000	12.687060000
O	10.793369000	7.276657000	12.826548000
C	12.160701000	7.804891000	12.926565000
H	12.482180000	7.703934000	13.982244000
H	12.013249000	8.877584000	12.695721000
C	13.190826000	7.174382000	11.991189000
H	14.115869000	7.747974000	12.226188000
H	13.398115000	6.142203000	12.338253000
C	12.940936000	7.197953000	10.464307000
H	13.943026000	7.238967000	9.990665000
H	12.481669000	8.166448000	10.176007000
B	12.153961000	5.956356000	9.768780000
C	12.538759000	4.419749000	10.336390000
H	12.413765000	4.374508000	11.436290000
C	14.047126000	4.255182000	10.044632000
H	14.371873000	3.257513000	10.420589000
H	14.595659000	4.993749000	10.665663000
C	14.479306000	4.402730000	8.572698000
H	15.583445000	4.513515000	8.534502000
H	14.272335000	3.458600000	8.030762000
C	13.827512000	5.586995000	7.822301000
H	13.967075000	5.438208000	6.728067000
H	14.382022000	6.517866000	8.064977000
C	12.335912000	5.820375000	8.145959000
H	12.054933000	6.790705000	7.679067000
C	11.407696000	4.734593000	7.558083000
H	10.339694000	5.041872000	7.677271000
H	11.546691000	4.660572000	6.456363000
C	11.572994000	3.333486000	8.176522000
H	12.489666000	2.860514000	7.776313000
H	10.741451000	2.677810000	7.842800000
C	11.636034000	3.337266000	9.715706000
H	11.944046000	2.332110000	10.081084000
H	10.593778000	3.445792000	10.117895000
C	7.360958000	4.451531000	9.405297000
C	7.922829000	3.334499000	8.754064000
H	8.298129000	2.480361000	9.331260000
C	8.015104000	3.309645000	7.352266000
H	8.459247000	2.437034000	6.851200000
C	7.541999000	4.390989000	6.591320000
H	7.618187000	4.366300000	5.494121000
C	6.972061000	5.504908000	7.234562000
H	6.600766000	6.355645000	6.643536000
C	6.888102000	5.537569000	8.634209000
H	6.485890000	6.423019000	9.149239000
C	5.637215000	3.761644000	11.581015000
C	5.425118000	3.290386000	12.895631000
H	6.217095000	3.407244000	13.650911000
C	4.215030000	2.668657000	13.231609000
H	4.058282000	2.291641000	14.253308000
C	3.200786000	2.533557000	12.265840000
H	2.249098000	2.050262000	12.532694000
C	3.405006000	3.009564000	10.960002000
H	2.615002000	2.898831000	10.202334000
C	4.622608000	3.616309000	10.612053000
H	4.786809000	3.970228000	9.584113000
C	8.298365000	2.070870000	11.863687000
H	9.181237000	1.667276000	11.331222000
H	7.370993000	1.773523000	11.344958000
H	8.292105000	1.642200000	12.883640000
C	12.375845000	3.129406000	14.432866000
C	12.235759000	1.723409000	14.497726000
H	11.354470000	1.255916000	14.037565000
C	13.211063000	0.950043000	15.137586000
H	13.099134000	-0.143358000	15.187306000
C	14.334560000	1.569171000	15.715898000
H	15.100888000	0.958771000	16.217286000
C	14.480980000	2.966954000	15.652546000
H	15.361044000	3.449441000	16.103186000
C	13.508630000	3.745696000	15.014560000
H	13.609242000	4.837888000	14.951779000
C	7.212782000	7.733528000	14.052777000
C	7.629965000	8.661124000	12.998313000
C	6.698531000	8.535147000	11.910951000

C	5.733285000	7.513565000	12.282273000
C	6.015060000	7.083722000	13.652538000
C	7.924423000	7.582151000	15.361398000
H	7.647599000	8.402940000	16.057698000
H	9.023488000	7.627965000	15.231046000
H	7.675465000	6.625753000	15.859361000
C	8.655081000	9.734193000	13.202699000
H	8.335267000	10.411483000	14.023232000
H	8.786960000	10.353081000	12.298516000
H	9.639466000	9.316776000	13.487683000
C	6.616434000	9.393873000	10.684023000
H	5.884826000	10.216676000	10.833898000
H	6.281863000	8.815374000	9.800871000
H	7.592426000	9.846154000	10.431937000
C	4.489693000	7.170294000	11.519102000
H	3.654050000	7.832463000	11.832599000
H	4.169047000	6.126785000	11.698102000
H	4.624016000	7.303868000	10.429029000
C	5.141673000	6.201681000	14.487138000
H	4.392477000	6.809188000	15.040380000
H	5.720820000	5.632806000	15.240504000
H	4.579929000	5.475291000	13.871454000
C	10.497415000	10.936407000	8.688521000
C	10.410010000	10.425900000	10.006436000
C	10.257272000	10.070109000	7.597036000
C	10.077898000	9.096142000	10.238202000
C	9.961136000	8.722215000	7.814848000
C	9.848287000	8.230162000	9.140129000
H	10.609536000	11.127845000	10.830336000
H	10.323884000	10.443433000	6.565223000
H	10.008813000	8.669450000	11.245240000
H	9.802858000	8.038604000	6.966303000
C	10.848487000	12.395795000	8.528976000
O	11.032975000	13.087472000	9.524916000
N	9.416017000	6.942184000	9.385717000
H	9.377712000	6.393787000	8.513786000
O	10.677980000	6.008658000	10.193742000
H	10.261750000	5.128108000	10.054526000
H	8.424775000	6.738750000	10.723977000
C	10.958920000	12.965474000	7.128089000
H	10.002134000	12.854445000	6.576648000
H	11.220433000	14.036310000	7.194676000
H	11.737146000	12.432860000	6.542551000

- **[23_Rh_BOH]⁺ (I2)**

Rh	7.136653000	5.803771000	13.019595000
P	7.083078000	4.395639000	11.339138000
N	8.438225000	3.356902000	11.648595000
C	9.099313000	3.524431000	12.837330000
N	10.099506000	2.699096000	13.139780000
C	10.724065000	2.907053000	14.316868000
N	10.395634000	3.907983000	15.161831000
C	9.409228000	4.710706000	14.786355000
N	8.679454000	4.576720000	13.640112000
O	9.071659000	5.745699000	15.550758000
C	9.982158000	6.053231000	16.653312000
H	10.195554000	5.116081000	17.200900000
H	9.391498000	6.724579000	17.303781000
C	11.271998000	6.696774000	16.127341000
H	11.384513000	6.382653000	15.069127000
H	11.163491000	7.802425000	16.098305000
C	12.544584000	6.288415000	16.890679000
H	12.577185000	5.180051000	16.919924000
H	12.465874000	6.612265000	17.955471000
B	13.877375000	6.911464000	16.227516000
C	15.353747000	5.983158000	16.667052000
H	15.222008000	6.139102000	17.761476000
C	16.576672000	6.725274000	16.162395000
H	17.491038000	6.201405000	16.529437000
H	16.584387000	7.734968000	16.618455000
C	16.669226000	6.853686000	14.629354000
H	17.466464000	7.579546000	14.368046000
H	16.986809000	5.890429000	14.184387000
C	15.333472000	7.315433000	14.009669000
H	15.391576000	7.182635000	12.904135000
H	15.208628000	8.401950000	14.191021000
C	14.094279000	6.607590000	14.543760000
H	13.192899000	7.174852000	14.209248000
C	13.913179000	5.130524000	14.217161000
H	12.908387000	4.803935000	14.559639000
H	13.915762000	4.993168000	13.110388000
C	14.974785000	4.199947000	14.827912000
H	15.924679000	4.286503000	14.266423000
H	14.654176000	3.144346000	14.719969000
C	15.208903000	4.511908000	16.318992000
H	16.129150000	3.976238000	16.652304000
H	14.379254000	4.080873000	16.915615000
C	7.455661000	5.087489000	9.706321000
C	8.772638000	5.538487000	9.463953000
H	9.558970000	5.348241000	10.210520000
C	9.073683000	6.231800000	8.284425000
H	10.103358000	6.571044000	8.096448000
C	8.060070000	6.501224000	7.346134000
H	8.296469000	7.051877000	6.423432000
C	6.747076000	6.064765000	7.587009000
H	5.953210000	6.271001000	6.853616000
C	6.442026000	5.356811000	8.761574000
H	5.413474000	5.012755000	8.940812000

C	5.678442000	3.269718000	11.115202000
C	4.789634000	3.131881000	12.203527000
H	4.964831000	3.739107000	13.106043000
C	3.714301000	2.234938000	12.127421000
H	3.022505000	2.132941000	12.976882000
C	3.523421000	1.468287000	10.964708000
H	2.678843000	0.765698000	10.901975000
C	4.410116000	1.595584000	9.880532000
H	4.260924000	0.991627000	8.972974000
C	5.486442000	2.492547000	9.951116000
H	6.172693000	2.591380000	9.097588000
C	8.782518000	2.251173000	10.752070000
H	9.831970000	1.958530000	10.927997000
H	8.662599000	2.586750000	9.705065000
H	8.129232000	1.374175000	10.932823000
C	11.826984000	2.013215000	14.705758000
C	12.366379000	1.091081000	13.779018000
H	11.944773000	1.048521000	12.765441000
C	13.426559000	0.256976000	14.153784000
H	13.849508000	-0.451956000	13.426488000
C	13.948706000	0.321049000	15.458935000
H	14.778038000	-0.340073000	15.752331000
C	13.409267000	1.228281000	16.389176000
H	13.813972000	1.275086000	17.411056000
C	12.360550000	2.075887000	16.014682000
H	11.932644000	2.794647000	16.726632000
C	6.447444000	7.352028000	14.585233000
C	7.330364000	7.989998000	13.638351000
C	6.747156000	7.856500000	12.316557000
C	5.499747000	7.121217000	12.458879000
C	5.309309000	6.825352000	13.863552000
C	6.572163000	7.353101000	16.076150000
H	5.779983000	7.991589000	16.522653000
H	7.547025000	7.755217000	16.403221000
H	6.460395000	6.337961000	16.504278000
C	8.600368000	8.713290000	13.953650000
H	8.419136000	9.805931000	14.050695000
H	9.356017000	8.575641000	13.155631000
H	9.041328000	8.356235000	14.901201000
C	7.279672000	8.448296000	11.051440000
H	6.908969000	9.488500000	10.918549000
H	6.964744000	7.867484000	10.163643000
H	8.385861000	8.487667000	11.053012000
C	4.492849000	6.882073000	11.374362000
H	3.723237000	7.682794000	11.380034000
H	3.971403000	5.913768000	11.500032000
H	4.967355000	6.887665000	10.376060000
C	4.106601000	6.190673000	14.482965000
H	3.414633000	6.969688000	14.874527000
H	4.378465000	5.539108000	15.336304000
H	3.535995000	5.588703000	13.750738000
O	14.111967000	8.286099000	16.480006000
H	13.518444000	8.652909000	17.158328000

- **[23_Rh_ACN_B_ACN]$^{2+}$**

Rh	7.629375000	6.076281000	12.531994000
P	7.146605000	4.709669000	10.729625000
N	7.819203000	3.197731000	11.217844000
C	8.556557000	3.147384000	12.374678000
N	9.105870000	1.981647000	12.719541000
C	9.872190000	1.985904000	13.830882000
N	10.184264000	3.110611000	14.510865000
C	9.603170000	4.235037000	14.105764000
N	8.679749000	4.301658000	13.101617000
O	9.887052000	5.393055000	14.676448000
C	10.932000000	5.452286000	15.716188000
H	10.849089000	4.548833000	16.349733000
H	10.647080000	6.341096000	16.311747000
C	12.323440000	5.615269000	15.117682000
H	12.571299000	4.708256000	14.523301000
H	12.298411000	6.466817000	14.403413000
C	13.389967000	5.868716000	16.197834000
H	13.376035000	5.028516000	16.930487000
H	13.093435000	6.766861000	16.782185000
B	14.927251000	6.094249000	15.651491000
C	15.988214000	6.618688000	16.798590000
H	15.956158000	5.958113000	17.696724000
C	15.530640000	8.026501000	17.262116000
H	16.253133000	8.435736000	18.005277000
H	14.573456000	7.914349000	17.817871000
C	15.335363000	9.066877000	16.133941000
H	14.759236000	9.931290000	16.531439000
H	16.319492000	9.488538000	15.847091000
C	14.627541000	8.526358000	14.869271000
H	14.736748000	9.276325000	14.050913000
H	13.533649000	8.464922000	15.070841000
C	15.107101000	7.132210000	14.388516000
H	14.451661000	6.841902000	13.531365000
C	16.563503000	7.108769000	13.859513000
H	16.735573000	6.120362000	13.374614000
H	16.694525000	7.858377000	13.044385000
C	17.661903000	7.336301000	14.924556000
H	17.756827000	8.421929000	15.127760000
H	18.645826000	7.039935000	14.498962000
C	17.438961000	6.583986000	16.257149000
H	18.145649000	6.987355000	17.018259000
H	17.736499000	5.518834000	16.123163000
N	15.403755000	4.677221000	15.199921000

C	15.787833000	3.620801000	14.912555000
C	16.284170000	2.303846000	14.563881000
H	15.484003000	1.548308000	14.695543000
H	17.139051000	2.038378000	15.218503000
H	16.623558000	2.290712000	13.508545000
C	7.996790000	5.208790000	9.200020000
C	9.006550000	4.420037000	8.607371000
H	9.301445000	3.459290000	9.050966000
C	9.649744000	4.865415000	7.439375000
H	10.431772000	4.241701000	6.980069000
C	9.294017000	6.094365000	6.857820000
H	9.795595000	6.435592000	5.939396000
C	8.299725000	6.891301000	7.454493000
H	8.023576000	7.858816000	7.008356000
C	7.659172000	6.457531000	8.625491000
H	6.896575000	7.098420000	9.094741000
C	5.453068000	4.238305000	10.260361000
C	4.719431000	3.422714000	11.156662000
H	5.177488000	3.082129000	12.097542000
C	3.414059000	3.021445000	10.837056000
H	2.855693000	2.376686000	11.532656000
C	2.823870000	3.436394000	9.628396000
H	1.799026000	3.121286000	9.379712000
C	3.549084000	4.241850000	8.734196000
H	3.097104000	4.556226000	7.781275000
C	4.860860000	4.640657000	9.041881000
H	5.420192000	5.251035000	8.319269000
C	7.613700000	1.949020000	10.455651000
H	8.587953000	1.498629000	10.189475000
H	7.050099000	2.179483000	9.535277000
H	7.040499000	1.219824000	11.058975000
C	10.415572000	0.701315000	14.314491000
C	10.149565000	-0.499514000	13.611209000
H	9.542523000	-0.463543000	12.695897000
C	10.656507000	-1.718015000	14.080166000
H	10.446170000	-2.647324000	13.529382000
C	11.431912000	-1.754860000	15.254613000
H	11.826737000	-2.714414000	15.622758000
C	11.700846000	-0.566387000	15.960030000
H	12.303092000	-0.596463000	16.880772000
C	11.198878000	0.655633000	15.494471000
H	11.402058000	1.587152000	16.041120000
C	6.908028000	6.774184000	14.608039000
C	7.515954000	7.874901000	13.930044000
C	6.776349000	8.095351000	12.676318000
C	5.683586000	7.153666000	12.629211000
C	5.817608000	6.263588000	13.776088000
C	7.218498000	6.268324000	15.977708000
H	6.422039000	6.623014000	16.668762000
H	8.184951000	6.640920000	16.358825000
H	7.212071000	5.162296000	16.028824000
C	8.629217000	8.738808000	14.429284000
H	8.207165000	9.648156000	14.910090000
H	9.284463000	9.080564000	13.605601000
H	9.250757000	8.219106000	15.180736000
C	6.995870000	9.243945000	11.742510000
H	6.480772000	10.145456000	12.140766000
H	6.583318000	9.043999000	10.736216000
H	8.068140000	9.497408000	11.641520000
C	4.531270000	7.195814000	11.679169000
H	3.753119000	7.862740000	12.111752000
H	4.068052000	6.204359000	11.529480000
H	4.809050000	7.612958000	10.693237000
C	4.821170000	5.243349000	14.233050000
H	4.130476000	5.710528000	14.969103000
H	5.306592000	4.387080000	14.739818000
H	4.204223000	4.860241000	13.400629000
N	9.401564000	6.658981000	11.666452000
C	10.417799000	7.026675000	11.243015000
C	11.691033000	7.484442000	10.720698000
H	12.486419000	7.334672000	11.478861000
H	11.631436000	8.562390000	10.469553000
H	11.949994000	6.915671000	9.805479000

- **$[23\_RhH\_B\_ACN]^{+}$**

Rh	7.826320000	5.763924000	12.383697000
P	7.185258000	4.210077000	10.908587000
N	7.944113000	2.767426000	11.521486000
C	8.778229000	2.878316000	12.601977000
N	9.435832000	1.779515000	12.996649000
C	10.239124000	1.912580000	14.065785000
N	10.449782000	3.083774000	14.698134000
C	9.777920000	4.138789000	14.237693000
N	8.878498000	4.103805000	13.214370000
O	9.956373000	5.330047000	14.789145000
C	10.977699000	5.487733000	15.833392000
H	10.958250000	4.595237000	16.487519000
H	10.624339000	6.363553000	16.411092000
C	12.362534000	5.738412000	15.247950000
H	12.665893000	4.849950000	14.651385000
H	12.293575000	6.587871000	14.534750000
C	13.408299000	6.043390000	16.334864000
H	13.426451000	5.206196000	17.071720000
H	13.071399000	6.931596000	16.912704000
B	14.936489000	6.329818000	15.795552000
C	15.998768000	6.807190000	16.961184000
H	15.985888000	6.097466000	17.821887000
C	15.523433000	8.179381000	17.505551000

H	16.244596000	8.558465000	18.266629000
H	14.571120000	8.021342000	18.058667000
C	15.305415000	9.276967000	16.437363000
H	14.722189000	10.111080000	16.886487000
H	16.282414000	9.725763000	16.166730000
C	14.593356000	8.796677000	15.151051000
H	14.683906000	9.590950000	14.373509000
H	13.502856000	8.708615000	15.357217000
C	15.087668000	7.439020000	14.589582000
H	14.427810000	7.188018000	13.726056000
C	16.539229000	7.466678000	14.047549000
H	16.720663000	6.510384000	13.505019000
H	16.651346000	8.262845000	13.274851000
C	17.645402000	7.650030000	15.113283000
H	17.727620000	8.724385000	15.374792000
H	18.629733000	7.390321000	14.664358000
C	17.444701000	6.824203000	16.406053000
H	18.154532000	7.195607000	17.181270000
H	17.754589000	5.771869000	16.210672000
C	7.712970000	4.306549000	9.153375000
C	7.202338000	3.422666000	8.170875000
H	6.430218000	2.683834000	8.428920000
C	7.667967000	3.486603000	6.848311000
H	7.264315000	2.791851000	6.095855000
C	8.641042000	4.434806000	6.484925000
H	9.000986000	4.484653000	5.445652000
C	9.147722000	5.321363000	7.450243000
H	9.904865000	6.070351000	7.171965000
C	8.686929000	5.257464000	8.775400000
H	9.084283000	5.949873000	9.533616000
C	5.426083000	3.721528000	10.840667000
C	4.894703000	2.911315000	11.871171000
H	5.553931000	2.522697000	12.662644000
C	3.528548000	2.589859000	11.886962000
H	3.129748000	1.945770000	12.685695000
C	2.672265000	3.089754000	10.888822000
H	1.600836000	2.837548000	10.903694000
C	3.188987000	3.910862000	9.871916000
H	2.524597000	4.304873000	9.087596000
C	4.558910000	4.224918000	9.843286000
H	4.951456000	4.856525000	9.032821000
C	7.809074000	1.463471000	10.854977000
H	8.655344000	1.276757000	10.164624000
H	6.861806000	1.445637000	10.288617000
H	7.785489000	0.660751000	11.613181000
C	10.941713000	0.708593000	14.571351000
C	10.806464000	-0.533402000	13.906385000
H	10.179248000	-0.593128000	13.005905000
C	11.466587000	-1.671062000	14.390661000
H	11.356092000	-2.632505000	13.866066000
C	12.266678000	-1.586287000	15.545372000
H	12.781179000	-2.482044000	15.926599000
C	12.405275000	-0.355489000	16.214012000
H	13.025178000	-0.287621000	17.121285000
C	11.749923000	0.785911000	15.730952000
H	11.850128000	1.749342000	16.250317000
C	6.888808000	6.864927000	14.213313000
C	7.986496000	7.657666000	13.657684000
C	7.646822000	7.978821000	12.294743000
C	6.362762000	7.369337000	11.998604000
C	5.875987000	6.714263000	13.214712000
C	6.798313000	6.392436000	15.633453000
H	6.398200000	7.203475000	16.280610000
H	7.789826000	6.108779000	16.032970000
H	6.119206000	5.524267000	15.735228000
C	9.112378000	8.279123000	14.428991000
H	8.877031000	9.345365000	14.640886000
H	10.067846000	8.258723000	13.870164000
H	9.271255000	7.779595000	15.400607000
C	8.400406000	8.922377000	11.402080000
H	8.045123000	9.962520000	11.571838000
H	8.246675000	8.694035000	10.330235000
H	9.487365000	8.905391000	11.608280000
C	5.553748000	7.628200000	10.761803000
H	4.992517000	8.581446000	10.877500000
H	4.808401000	6.832215000	10.577048000
H	6.190524000	7.727162000	9.861889000
C	4.508102000	6.130218000	13.406175000
H	3.788757000	6.925264000	13.703108000
H	4.494794000	5.361178000	14.202261000
H	4.119162000	5.666989000	12.479489000
H	9.114275000	5.840436000	11.483004000
C	15.782456000	3.920119000	14.859064000
N	15.424275000	4.951710000	15.250775000
C	16.244622000	2.634957000	14.371661000
H	15.479675000	1.857364000	14.568286000
H	17.185210000	2.351510000	14.885764000
H	16.433328000	2.686618000	13.280408000

- **[23_RhH_B_THF]$^+$**

Rh	7.650446000	5.789978000	12.443330000
P	7.064361000	4.317859000	10.859706000
N	7.579835000	2.801384000	11.560573000
C	8.408006000	2.852303000	12.649390000
N	8.952369000	1.707440000	13.082867000
C	9.785768000	1.800758000	14.133985000
N	10.152915000	2.972769000	14.692020000
C	9.575241000	4.069885000	14.205273000

N	8.622437000	4.077128000	13.231056000
O	9.906165000	5.264390000	14.673554000
C	10.981148000	5.377563000	15.666826000
H	10.945284000	4.496893000	16.335994000
H	10.706819000	6.281524000	16.245635000
C	12.348047000	5.545545000	15.011575000
H	12.558763000	4.639180000	14.402806000
H	12.292020000	6.399210000	14.301645000
C	13.458583000	5.787274000	16.054006000
H	13.576291000	4.867932000	16.675229000
H	13.083973000	6.546885000	16.776508000
B	14.915223000	6.318345000	15.536442000
C	15.939445000	6.762318000	16.721819000
H	16.031307000	5.981221000	17.514324000
C	15.323233000	8.007638000	17.424095000
H	16.021373000	8.393673000	18.202692000
H	14.418946000	7.678041000	17.982102000
C	14.931433000	9.175021000	16.484803000
H	14.246154000	9.863008000	17.027869000
H	15.829402000	9.786623000	16.264154000
C	14.267757000	8.747797000	15.153675000
H	14.247875000	9.624841000	14.465589000
H	13.199765000	8.501215000	15.348063000
C	14.925084000	7.526199000	14.449506000
H	14.288163000	7.308833000	13.558744000
C	16.352351000	7.819761000	13.915458000
H	16.663896000	6.973099000	13.263502000
H	16.338132000	8.710683000	13.244650000
C	17.436981000	8.024876000	14.996488000
H	17.376545000	9.059069000	15.391316000
H	18.441047000	7.960210000	14.521459000
C	17.365258000	7.023611000	16.170498000
H	18.037303000	7.378324000	16.986268000
H	17.801205000	6.055102000	15.835000000
C	7.847272000	4.354769000	9.197676000
C	7.474367000	3.438703000	8.182517000
H	6.665578000	2.713530000	8.350183000
C	8.124027000	3.455026000	6.938647000
H	7.826707000	2.734970000	6.160839000
C	9.144118000	4.389545000	6.684097000
H	9.647984000	4.402329000	5.705292000
C	9.512408000	5.309869000	7.679540000
H	10.303883000	6.049773000	7.485063000
C	8.869290000	5.291299000	8.928355000
H	9.159224000	6.009428000	9.711035000
C	5.298710000	4.002064000	10.525187000
C	4.525460000	3.316347000	11.491374000
H	5.004568000	2.922394000	12.400965000
C	3.150201000	3.122622000	11.289288000
H	2.560581000	2.573802000	12.039623000
C	2.527698000	3.626735000	10.132030000
H	1.448933000	3.473430000	9.975198000
C	3.286161000	4.325497000	9.177451000
H	2.803911000	4.724396000	8.271882000
C	4.665723000	4.513819000	9.369557000
H	5.248581000	5.059002000	8.612764000
C	7.332419000	1.498523000	10.924371000
H	8.200994000	1.174415000	10.317823000
H	6.440134000	1.578478000	10.279894000
H	7.140385000	0.737820000	11.702234000
C	10.348309000	0.551062000	14.698132000
C	10.036211000	-0.701775000	14.117910000
H	9.377883000	-0.735214000	13.238548000
C	10.562765000	-1.882522000	14.658728000
H	10.315203000	-2.852256000	14.200376000
C	11.404965000	-1.829944000	15.785152000
H	11.815918000	-2.758954000	16.209917000
C	11.719825000	-0.588633000	16.368841000
H	12.375456000	-0.545300000	17.252080000
C	11.197330000	0.595112000	15.830048000
H	11.436114000	1.567670000	16.282783000
C	6.965500000	6.900060000	14.368944000
C	7.874623000	7.729989000	13.598859000
C	7.265923000	7.948816000	12.299901000
C	5.979942000	7.266679000	12.293695000
C	5.792693000	6.620287000	13.579362000
C	7.137282000	6.498114000	15.802762000
H	6.526638000	7.166298000	16.448703000
H	8.187323000	6.578079000	16.134090000
H	6.786477000	5.463634000	15.987045000
C	9.101033000	8.422521000	14.114411000
H	8.826537000	9.397105000	14.574890000
H	9.826735000	8.633371000	13.305657000
H	9.618588000	7.826317000	14.887113000
C	7.758566000	8.895343000	11.242769000
H	7.348862000	9.913686000	11.421101000
H	7.441103000	8.584757000	10.228975000
H	8.862181000	8.975659000	11.246707000
C	4.930739000	7.416618000	11.234351000
H	4.337215000	8.334535000	11.439810000
H	4.227010000	6.564185000	11.220218000
H	5.367793000	7.527316000	10.223736000
C	4.542980000	5.945821000	14.065432000
H	3.875068000	6.676893000	14.573019000
H	4.763824000	5.144287000	14.797173000
H	3.965933000	5.497673000	13.233923000
C	15.319468000	4.557900000	13.406480000
O	15.647889000	4.974894000	14.779799000
C	15.295151000	3.032957000	13.456343000
C	16.049599000	3.812833000	15.581917000

C	16.325934000	2.720313000	14.554336000
H	16.119580000	4.947304000	12.743929000
H	14.289451000	2.667105000	13.751361000
H	15.210665000	3.555175000	16.260552000
H	16.928444000	4.111135000	16.182747000
H	16.210760000	1.708051000	14.988058000
H	17.358747000	2.809264000	14.158485000
H	15.549312000	2.584032000	12.476507000
H	14.359297000	5.026713000	13.129011000
H	8.986879000	5.922035000	11.621008000

- **[23_RhH_B_CH_2Cl_2]$^+$**

Rh	7.773724000	5.783515000	12.407136000
P	7.150409000	4.263961000	10.887616000
N	8.028835000	2.847616000	11.384653000
C	8.883556000	2.947629000	12.449852000
N	9.608460000	1.866925000	12.765564000
C	10.447252000	1.989929000	13.809060000
N	10.615976000	3.139230000	14.494649000
C	9.864411000	4.171968000	14.120986000
N	8.934592000	4.142467000	13.125889000
O	9.982825000	5.339976000	14.741577000
C	10.999985000	5.497988000	15.782718000
H	11.133257000	4.532679000	16.306588000
H	10.551372000	6.228633000	16.484299000
C	12.314724000	6.020351000	15.212981000
H	12.714083000	5.284546000	14.483147000
H	12.108914000	6.952411000	14.643917000
C	13.350852000	6.286210000	16.318828000
H	13.613314000	5.329974000	16.834691000
H	12.898946000	6.897546000	17.137944000
B	14.724750000	6.957336000	15.931697000
C	15.734269000	7.465148000	17.034791000
H	15.458791000	7.128795000	18.059340000
C	15.556446000	9.021460000	17.005080000
H	16.255364000	9.490696000	17.734178000
H	14.533747000	9.254191000	17.380916000
C	15.733914000	9.673542000	15.616003000
H	15.358009000	10.719122000	15.657060000
H	16.812169000	9.763836000	15.378495000
C	15.010322000	8.925190000	14.475018000
H	15.336578000	9.331458000	13.490527000
H	13.919427000	9.144086000	14.540314000
C	15.192322000	7.367917000	14.482444000
H	14.540386000	6.963832000	13.677470000
C	16.642966000	6.880897000	14.205614000
H	16.604653000	5.775046000	14.084123000
H	16.997478000	7.276950000	13.226525000
C	17.677484000	7.220265000	15.300543000
H	18.001140000	8.275137000	15.197420000
H	18.596781000	6.619448000	15.127127000
C	17.177422000	6.971184000	16.739771000
H	17.892472000	7.422964000	17.464563000
H	17.204526000	5.875548000	16.942209000
C	7.573463000	4.490426000	9.115756000
C	7.031740000	3.659741000	8.103778000
H	6.294935000	2.882068000	8.351477000
C	7.419983000	3.826109000	6.765218000
H	6.992710000	3.171375000	5.990270000
C	8.346034000	4.824818000	6.415164000
H	8.645578000	4.954694000	5.363738000
C	8.882965000	5.659458000	7.410049000
H	9.603694000	6.447385000	7.142877000
C	8.498921000	5.493796000	8.750609000
H	8.918635000	6.147091000	9.531094000
C	5.423342000	3.666427000	10.884229000
C	5.007555000	2.759701000	11.887072000
H	5.736902000	2.365551000	12.611206000
C	3.667166000	2.349699000	11.960863000
H	3.359048000	1.632413000	12.737032000
C	2.721767000	2.853122000	11.048701000
H	1.671001000	2.530440000	11.108376000
C	3.123085000	3.768191000	10.060290000
H	2.388588000	4.165702000	9.343150000
C	4.466321000	4.173729000	9.974625000
H	4.767414000	4.880548000	9.187508000
C	7.957994000	1.572928000	10.653744000
H	8.858473000	1.424467000	10.026195000
H	7.060745000	1.576642000	10.011870000
H	7.881572000	0.733124000	11.367856000
C	11.232502000	0.805079000	14.224331000
C	11.055888000	-0.438233000	13.570476000
H	10.334677000	-0.511689000	12.744492000
C	11.791319000	-1.560093000	13.975324000
H	11.646460000	-2.523551000	13.462921000
C	12.712544000	-1.456593000	15.034896000
H	13.288676000	-2.339781000	15.352040000
C	12.897051000	-0.223351000	15.686545000
H	13.617774000	-0.132028000	16.513077000
C	12.162972000	0.900973000	15.285944000
H	12.312163000	1.867083000	15.786658000
C	6.826630000	6.764713000	14.302946000
C	7.883659000	7.623180000	13.765050000
C	7.508979000	7.990832000	12.423898000
C	6.245882000	7.342901000	12.119923000
C	5.803831000	6.619175000	13.315022000
C	6.781149000	6.230081000	15.703294000
H	6.393970000	7.008211000	16.396972000

H	7.786300000	5.938524000	16.061222000
H	6.113362000	5.351373000	15.786846000
C	8.996744000	8.254028000	14.547699000
H	8.729860000	9.304343000	14.798347000
H	9.947070000	8.283234000	13.980488000
H	9.180016000	7.726476000	15.500184000
C	8.210769000	8.999350000	11.560601000
H	7.815040000	10.016562000	11.774258000
H	8.052604000	8.805354000	10.482668000
H	9.300215000	9.019465000	11.753146000
C	5.407693000	7.620448000	10.906610000
H	4.824224000	8.554687000	11.060649000
H	4.680615000	6.810305000	10.711234000
H	6.025287000	7.761921000	9.999050000
C	4.462130000	5.975183000	13.498918000
H	3.709790000	6.736737000	13.801550000
H	4.480715000	5.199241000	14.288014000
H	4.093402000	5.504940000	12.567276000
H	9.027620000	5.938905000	11.470412000
Cl	15.565295000	2.922428000	15.010413000
C	16.129799000	2.594012000	16.680662000
H	16.601835000	1.596860000	16.690578000
H	16.840636000	3.388454000	16.965206000
Cl	14.798865000	2.588153000	17.886901000

- **[23_RhH_B_Et$_2$O]$^+$**

Rh	7.980254000	5.676676000	12.357077000
P	7.252686000	4.136578000	10.908051000
N	7.937373000	2.665688000	11.541159000
C	8.777357000	2.748434000	12.619669000
N	9.362874000	1.619690000	13.041415000
C	10.174142000	1.725763000	14.107424000
N	10.470008000	2.898346000	14.703111000
C	9.865291000	3.982239000	14.218862000
N	8.954254000	3.978852000	13.204811000
O	10.126217000	5.174645000	14.735375000
C	11.162911000	5.300989000	15.767821000
H	11.155961000	4.390122000	16.395622000
H	10.822837000	6.160813000	16.377311000
C	12.536542000	5.569539000	15.163031000
H	12.817872000	4.706508000	14.521559000
H	12.453956000	6.449323000	14.488833000
C	13.604821000	5.826687000	16.243301000
H	13.736348000	4.905881000	16.857729000
H	13.191219000	6.570396000	16.961826000
B	15.051649000	6.394789000	15.760696000
C	16.066240000	6.824339000	16.955582000
H	16.155504000	6.041651000	17.746923000
C	15.428753000	8.054156000	17.671655000
H	16.116057000	8.430514000	18.464170000
H	14.521802000	7.704552000	18.212498000
C	15.035146000	9.232888000	16.749597000
H	14.342611000	9.908221000	17.299119000
H	15.929742000	9.852899000	16.539960000
C	14.380482000	8.815244000	15.412755000
H	14.348347000	9.699582000	14.734917000
H	13.316785000	8.547429000	15.600959000
C	15.064303000	7.616272000	14.691676000
H	14.434754000	7.413315000	13.791996000
C	16.491119000	7.946132000	14.178907000
H	16.817513000	7.127684000	13.500874000
H	16.467817000	8.859156000	13.539094000
C	17.568846000	8.129116000	15.271745000
H	17.503611000	9.153951000	15.689403000
H	18.575347000	8.077996000	14.800464000
C	17.495104000	7.102816000	16.424055000
H	18.156034000	7.447462000	17.252904000
H	17.937617000	6.142828000	16.076317000
C	7.775726000	4.178280000	9.149496000
C	7.222787000	3.299613000	8.185377000
H	6.420356000	2.599842000	8.459573000
C	7.685045000	3.318671000	6.860243000
H	7.248426000	2.628128000	6.122461000
C	8.696610000	4.216929000	6.475613000
H	9.053881000	4.231742000	5.434330000
C	9.245053000	5.098609000	7.422330000
H	10.032404000	5.809056000	7.127292000
C	8.787968000	5.079084000	8.750205000
H	9.218284000	5.767603000	9.493926000
C	5.470387000	3.738175000	10.854651000
C	4.903115000	2.974962000	11.901926000
H	5.545451000	2.568493000	12.698242000
C	3.522664000	2.722973000	11.928261000
H	3.095208000	2.115035000	12.740379000
C	2.688512000	3.246553000	10.923542000
H	1.605819000	3.048990000	10.946543000
C	3.241740000	4.021143000	9.889514000
H	2.594831000	4.432969000	9.099737000
C	4.625609000	4.265419000	9.850517000
H	5.045506000	4.861267000	9.026802000
C	7.727141000	1.360105000	10.897390000
H	8.552391000	1.121388000	10.197649000
H	6.771756000	1.381216000	10.344980000
H	7.675615000	0.571300000	11.668561000
C	10.782206000	0.489175000	14.653236000
C	10.567671000	-0.756941000	14.017151000
H	9.953846000	-0.794627000	13.106329000
C	11.134274000	-1.926188000	14.542405000

H	10.963494000	-2.890504000	14.039730000
C	11.917888000	-1.869198000	15.710107000
H	12.359241000	-2.789446000	16.123268000
C	12.134867000	-0.634681000	16.349948000
H	12.743903000	-0.587835000	17.265683000
C	11.574102000	0.538002000	15.825400000
H	11.736740000	1.505052000	16.321712000
C	7.118557000	6.843947000	14.180640000
C	8.243059000	7.578307000	13.599925000
C	7.899649000	7.895933000	12.236773000
C	6.584922000	7.342583000	11.966467000
C	6.086073000	6.725902000	13.197487000
C	7.026291000	6.395520000	15.608478000
H	6.668867000	7.231739000	16.248491000
H	8.009915000	6.075572000	16.000084000
H	6.312754000	5.558216000	15.731029000
C	9.406781000	8.157781000	14.347110000
H	9.215895000	9.232125000	14.562807000
H	10.348224000	8.101462000	13.767430000
H	9.568514000	7.652947000	15.315561000
C	8.682919000	8.791691000	11.320720000
H	8.382880000	9.849940000	11.485070000
H	8.498568000	8.560469000	10.254322000
H	9.771313000	8.722877000	11.507833000
C	5.771783000	7.620630000	10.736521000
H	5.254017000	8.598788000	10.846939000
H	4.990044000	6.855943000	10.571213000
H	6.400630000	7.680995000	9.827642000
C	4.695932000	6.207317000	13.415480000
H	4.020096000	7.036111000	13.722222000
H	4.660699000	5.441987000	14.214494000
H	4.269322000	5.760047000	12.497647000
H	9.264643000	5.685829000	11.446131000
O	15.810939000	5.002264000	14.956861000
C	16.283438000	3.889278000	15.772807000
H	17.332591000	3.676829000	15.478650000
H	16.298708000	4.295576000	16.799361000
C	15.584110000	4.703551000	13.551022000
H	14.791578000	3.929625000	13.472191000
H	15.176085000	5.640008000	13.132701000
C	16.848515000	4.277325000	12.818110000
H	16.615611000	4.173893000	11.738699000
H	17.651270000	5.034170000	12.922794000
H	17.237865000	3.299711000	13.165768000
C	15.429140000	2.630600000	15.699667000
H	15.470389000	2.134705000	14.709573000
H	15.818710000	1.905567000	16.443197000
H	14.369532000	2.835404000	15.949285000

- **[23_RhH_B_H_2O]$^+$**

Rh	7.931256000	5.747329000	12.357108000
P	7.339408000	4.161410000	10.893930000
N	8.112572000	2.741542000	11.538831000
C	8.922163000	2.879756000	12.634925000
N	9.588155000	1.797025000	13.057374000
C	10.365488000	1.955269000	14.142451000
N	10.539696000	3.137122000	14.766846000
C	9.861890000	4.174889000	14.278400000
N	8.989737000	4.114469000	13.233190000
O	10.005098000	5.375201000	14.822397000
C	10.979051000	5.553660000	15.906691000
H	10.953245000	4.658528000	16.557103000
H	10.581843000	6.419059000	16.472118000
C	12.382315000	5.835375000	15.382870000
H	12.721283000	4.969688000	14.774072000
H	12.334629000	6.706910000	14.694881000
C	13.379593000	6.111673000	16.523190000
H	13.365178000	5.251411000	17.235693000
H	13.006284000	6.972754000	17.121921000
B	14.910799000	6.411525000	16.082149000
C	15.934306000	6.836015000	17.268279000
H	15.831108000	6.171431000	18.160659000
C	15.516271000	8.261394000	17.729657000
H	16.210928000	8.633566000	18.517757000
H	14.523221000	8.183957000	18.225162000
C	15.428557000	9.317733000	16.601467000
H	14.869398000	10.202934000	16.976724000
H	16.444448000	9.700098000	16.374899000
C	14.765754000	8.821780000	15.293561000
H	14.957102000	9.567664000	14.487650000
H	13.661262000	8.813525000	15.433384000
C	15.192413000	7.400750000	14.830773000
H	14.563336000	7.150525000	13.944087000
C	16.672913000	7.311121000	14.370864000
H	16.840504000	6.319755000	13.872153000
H	16.874496000	8.041848000	13.553958000
C	17.728904000	7.494730000	15.484941000
H	17.845558000	8.576360000	15.695110000
H	18.722652000	7.169456000	15.105604000
C	17.413171000	6.750637000	16.803394000
H	18.095227000	7.126850000	17.600121000
H	17.692516000	5.675821000	16.685940000
C	7.891471000	4.240836000	9.145618000
C	7.402016000	3.341544000	8.166389000
H	6.631157000	2.600405000	8.421559000
C	7.887981000	3.392169000	6.850572000
H	7.500757000	2.685397000	6.100686000
C	8.860903000	4.341820000	6.490776000

H	9.237019000	4.380981000	5.456793000
C	9.347099000	5.243220000	7.452913000
H	10.104394000	5.993060000	7.177357000
C	8.865729000	5.192986000	8.771286000
H	9.247148000	5.896931000	9.527072000
C	5.589199000	3.643515000	10.808720000
C	5.058264000	2.836261000	11.841790000
H	5.713977000	2.466360000	12.645043000
C	3.697183000	2.493576000	11.845182000
H	3.299083000	1.852066000	12.646344000
C	2.845063000	2.968877000	10.831603000
H	1.777605000	2.699982000	10.836807000
C	3.360987000	3.786571000	9.811543000
H	2.699994000	4.161314000	9.015013000
C	4.726076000	4.121980000	9.795598000
H	5.118247000	4.750472000	8.982564000
C	8.014767000	1.427293000	10.885874000
H	8.881370000	1.246030000	10.219822000
H	7.082731000	1.387275000	10.295887000
H	7.984450000	0.633547000	11.653237000
C	11.077537000	0.769732000	14.675849000
C	10.961880000	-0.485753000	14.032529000
H	10.342415000	-0.569154000	13.128637000
C	11.631230000	-1.606365000	14.542614000
H	11.536292000	-2.578515000	14.035021000
C	12.422132000	-1.490522000	15.701002000
H	12.945584000	-2.372554000	16.101518000
C	12.542165000	-0.246075000	16.347188000
H	13.158068000	-0.153622000	17.254937000
C	11.876704000	0.877950000	15.839067000
H	11.964493000	1.852716000	16.338902000
C	6.940900000	6.862999000	14.149448000
C	8.039376000	7.661800000	13.603896000
C	7.723285000	7.957512000	12.229478000
C	6.454072000	7.325752000	11.916851000
C	5.951294000	6.682786000	13.132852000
C	6.826106000	6.412603000	15.575057000
H	6.402669000	7.229140000	16.200099000
H	7.811944000	6.147410000	16.000240000
H	6.155267000	5.538019000	15.676872000
C	9.141065000	8.309816000	14.388328000
H	8.882426000	9.372732000	14.589903000
H	10.104885000	8.304315000	13.843627000
H	9.295430000	7.819223000	15.365187000
C	8.482199000	8.897121000	11.337125000
H	8.108439000	9.934658000	11.481100000
H	8.355440000	8.648294000	10.266267000
H	9.564638000	8.899495000	11.566657000
C	5.667220000	7.553209000	10.659802000
H	5.088028000	8.498455000	10.749708000
H	4.939201000	6.742043000	10.472057000
H	6.320675000	7.649693000	9.771686000
C	4.587658000	6.083259000	13.306044000
H	3.851295000	6.873551000	13.572359000
H	4.567946000	5.329066000	14.116028000
H	4.224721000	5.597219000	12.380434000
O	15.405746000	4.858077000	15.506497000
H	9.234357000	5.825193000	11.478592000
H	16.155930000	4.978148000	14.881397000
H	15.763970000	4.310936000	16.237433000

- **[23_RhH_B_EtOH]$^+$**

Rh	7.921932000	5.731102000	12.399254000
P	7.275024000	4.190013000	10.912621000
N	8.063490000	2.748958000	11.490988000
C	8.916760000	2.855766000	12.556616000
N	9.599915000	1.761699000	12.919383000
C	10.427301000	1.891055000	13.970346000
N	10.634581000	3.056673000	14.615523000
C	9.930924000	4.105670000	14.190939000
N	9.009678000	4.072545000	13.187677000
O	10.098141000	5.290715000	14.761308000
C	11.149337000	5.453789000	15.773045000
H	11.192500000	4.539360000	16.394389000
H	10.786208000	6.294107000	16.395458000
C	12.497710000	5.783443000	15.142688000
H	12.796019000	4.934258000	14.486502000
H	12.366436000	6.662838000	14.475778000
C	13.585630000	6.073676000	16.193558000
H	13.686210000	5.191420000	16.869485000
H	13.219760000	6.886382000	16.859886000
B	15.044741000	6.518726000	15.621161000
C	16.168306000	6.888944000	16.730803000
H	16.237195000	6.120226000	17.538225000
C	15.701018000	8.199883000	17.427417000
H	16.463957000	8.535053000	18.167566000
H	14.792610000	7.967525000	18.026738000
C	15.380568000	9.377100000	16.474633000
H	14.810465000	10.151889000	17.033615000
H	16.324213000	9.877102000	16.177338000
C	14.592295000	8.988300000	15.201344000
H	14.612454000	9.846717000	14.490246000
H	13.520506000	8.851109000	15.470169000
C	15.084554000	7.691912000	14.498180000
H	14.371668000	7.500913000	13.658337000
C	16.495499000	7.824036000	13.863223000
H	16.676450000	6.932173000	13.221340000
H	16.526042000	8.693063000	13.164997000

C	17.664460000	7.947980000	14.868242000
H	17.731823000	8.995546000	15.224911000
H	18.623441000	7.767410000	14.333723000
C	17.575467000	7.000439000	16.087002000
H	18.325145000	7.323605000	16.846300000
H	17.897955000	5.982366000	15.772551000
C	7.771055000	4.322595000	9.150560000
C	7.260165000	3.444095000	8.163326000
H	6.506840000	2.686671000	8.422977000
C	7.700884000	3.537380000	6.834024000
H	7.297654000	2.846255000	6.078022000
C	8.648414000	4.510302000	6.468467000
H	8.988713000	4.583126000	5.423954000
C	9.154403000	5.392217000	7.438375000
H	9.891152000	6.160643000	7.158340000
C	8.718832000	5.298751000	8.770292000
H	9.115304000	5.987517000	9.532202000
C	5.521927000	3.678595000	10.865572000
C	5.017675000	2.846984000	11.892657000
H	5.694148000	2.456471000	12.668500000
C	3.656453000	2.506351000	11.924526000
H	3.278786000	1.845787000	12.720068000
C	2.778266000	3.007878000	10.946418000
H	1.710795000	2.740416000	10.973893000
C	3.267977000	3.849974000	9.933336000
H	2.586334000	4.245246000	9.164626000
C	4.632846000	4.183517000	9.888461000
H	5.004434000	4.831396000	9.080969000
C	7.934060000	1.451920000	10.809629000
H	8.778289000	1.279009000	10.113312000
H	6.984132000	1.434300000	10.247879000
H	7.919441000	0.639833000	11.558146000
C	11.160533000	0.691286000	14.439515000
C	11.048348000	-0.535675000	13.742867000
H	10.416552000	-0.586052000	12.845035000
C	11.738508000	-1.669858000	14.191812000
H	11.647259000	-2.619087000	13.641936000
C	12.544858000	-1.597153000	15.342998000
H	13.083608000	-2.490175000	15.696002000
C	12.659721000	-0.381962000	16.043595000
H	13.285222000	-0.323888000	16.947600000
C	11.975114000	0.756198000	15.595422000
H	12.056658000	1.707178000	16.140528000
C	6.989113000	6.792007000	14.255041000
C	8.074936000	7.604709000	13.704375000
C	7.722165000	7.945629000	12.349911000
C	6.442211000	7.327982000	12.052667000
C	5.969969000	6.649501000	13.262110000
C	6.916153000	6.294257000	15.667593000
H	6.526097000	7.093875000	16.334744000
H	7.912536000	6.002083000	16.048724000
H	6.237340000	5.425122000	15.762527000
C	9.199806000	8.224077000	14.479007000
H	8.959679000	9.286912000	14.702155000
H	10.154250000	8.214227000	13.918134000
H	9.362919000	7.715507000	15.445291000
C	8.460187000	8.910625000	11.467300000
H	8.093506000	9.943987000	11.653398000
H	8.303815000	8.695528000	10.393110000
H	9.548265000	8.903534000	11.668122000
C	5.622485000	7.597649000	10.825195000
H	5.056635000	8.546201000	10.955895000
H	4.880597000	6.799340000	10.636548000
H	6.252242000	7.710381000	9.921969000
C	4.608102000	6.051818000	13.453698000
H	3.883827000	6.838421000	13.760866000
H	4.604287000	5.275481000	14.242729000
H	4.218831000	5.594405000	12.524267000
H	9.195136000	5.831478000	11.480399000
O	15.631925000	5.155793000	14.784464000
C	15.736009000	3.837698000	15.403331000
H	14.716114000	3.428553000	15.560108000
H	16.187301000	4.032853000	16.393723000
C	16.594520000	2.916269000	14.556047000
H	17.614858000	3.326449000	14.421831000
H	16.679572000	1.929089000	15.053421000
H	16.145345000	2.747110000	13.555104000
H	15.204388000	5.083959000	13.905986000

- **[23_RhH_B_MeOH]$^+$**

Rh	7.897043000	5.725334000	12.398627000
P	7.255285000	4.175598000	10.919218000
N	8.016992000	2.732462000	11.525687000
C	8.855434000	2.841147000	12.603264000
N	9.516004000	1.742022000	12.990836000
C	10.326253000	1.872605000	14.055185000
N	10.539545000	3.042947000	14.688790000
C	9.861457000	4.097594000	14.237810000
N	8.956289000	4.064687000	13.219503000
O	10.037777000	5.287082000	14.795287000
C	11.079665000	5.447622000	15.818129000
H	11.103989000	4.537764000	16.447331000
H	10.718015000	6.297035000	16.429341000
C	12.438067000	5.751573000	15.197636000
H	12.733490000	4.902979000	14.543377000
H	12.330216000	6.636615000	14.534266000
C	13.524788000	6.013510000	16.256578000
H	13.592806000	5.130260000	16.936177000

H	13.184111000	6.838831000	16.920794000
B	14.994324000	6.405818000	15.684808000
C	16.131039000	6.770510000	16.787892000
H	16.179025000	6.014469000	17.607905000
C	15.700020000	8.108410000	17.454503000
H	16.464461000	8.434548000	18.197142000
H	14.777392000	7.916246000	18.045581000
C	15.428508000	9.274101000	16.472988000
H	14.876167000	10.077872000	17.008024000
H	16.390728000	9.739909000	16.179208000
C	14.641432000	8.883236000	15.199094000
H	14.700545000	9.722123000	14.467187000
H	13.563119000	8.793242000	15.460081000
C	15.077787000	7.548499000	14.532110000
H	14.360840000	7.358595000	13.698914000
C	16.494183000	7.602525000	13.897948000
H	16.655249000	6.679386000	13.280051000
H	16.557283000	8.423079000	13.146344000
C	17.663668000	7.739793000	14.898621000
H	17.745490000	8.798557000	15.214883000
H	18.621413000	7.522948000	14.376064000
C	17.545016000	6.837444000	16.149033000
H	18.297313000	7.168189000	16.901770000
H	17.863650000	5.804276000	15.872939000
C	7.779602000	4.279357000	9.163290000
C	7.266567000	3.399707000	8.178207000
H	6.494152000	2.660623000	8.434634000
C	7.730425000	3.467764000	6.855239000
H	7.325039000	2.776007000	6.100962000
C	8.704197000	4.416005000	6.493899000
H	9.062920000	4.469020000	5.454364000
C	9.213283000	5.298416000	7.461741000
H	9.971132000	6.047320000	7.185143000
C	8.754239000	5.230353000	8.787285000
H	9.153824000	5.919405000	9.547402000
C	5.496618000	3.685267000	10.852520000
C	4.968667000	2.868974000	11.879983000
H	5.630213000	2.477353000	12.668020000
C	3.603054000	2.545258000	11.896937000
H	3.206924000	1.896501000	12.693217000
C	2.743932000	3.048674000	10.903020000
H	1.672979000	2.794497000	10.918704000
C	3.257241000	3.875676000	9.889178000
H	2.590672000	4.272389000	9.108072000
C	4.626582000	4.192194000	9.859422000
H	5.016419000	4.828369000	9.051225000
C	7.883013000	1.429736000	10.856311000
H	8.735218000	1.240396000	10.174126000
H	6.941228000	1.416578000	10.280862000
H	7.848752000	0.625852000	11.612972000

C	11.031044000	0.667681000	14.553944000
C	10.896317000	-0.571296000	13.883028000
H	10.268854000	-0.627217000	12.982493000
C	11.558084000	-1.710398000	14.361224000
H	11.448952000	-2.669274000	13.831694000
C	12.358995000	-1.630169000	15.515721000
H	12.875675000	-2.526901000	15.891621000
C	12.497119000	-0.402565000	16.189944000
H	13.119642000	-0.338174000	17.095598000
C	11.840402000	0.740153000	15.712889000
H	11.941486000	1.701611000	16.235652000
C	6.955933000	6.815149000	14.232893000
C	8.053956000	7.612395000	13.684008000
C	7.717063000	7.941271000	12.322448000
C	6.434415000	7.331998000	12.019857000
C	5.945128000	6.670430000	13.231732000
C	6.864949000	6.333077000	15.649803000
H	6.468377000	7.140589000	16.303503000
H	7.856102000	6.043048000	16.045803000
H	6.183273000	5.466280000	15.745984000
C	9.177556000	8.229930000	14.461764000
H	8.940578000	9.294442000	14.680534000
H	10.133945000	8.214335000	13.904372000
H	9.335338000	7.723666000	15.430088000
C	8.472141000	8.889834000	11.436362000
H	8.115257000	9.928843000	11.609702000
H	8.321807000	8.666074000	10.363064000
H	9.558555000	8.873052000	11.645519000
C	5.627881000	7.596164000	10.782457000
H	5.066715000	8.549200000	10.900214000
H	4.882644000	6.801040000	10.593624000
H	6.266423000	7.697794000	9.884084000
C	4.576913000	6.085181000	13.416881000
H	3.856159000	6.879245000	13.712983000
H	4.561193000	5.314228000	14.211059000
H	4.191512000	5.623988000	12.487699000
H	9.181576000	5.806596000	11.493981000
O	15.463101000	4.993167000	14.854488000
C	15.823886000	3.779123000	15.554484000
H	16.000988000	2.978850000	14.811374000
H	14.963661000	3.511300000	16.191213000
H	16.158490000	5.235355000	14.203617000
H	16.723020000	3.932085000	16.183183000

- **[23_RhH_B_3a]$^+$ (Coordination *via* NHOH/N)**

Rh	7.587949000	5.763217000	12.522009000

P	6.884050000	4.173487000	11.113889000
N	7.626641000	2.736140000	11.761805000
C	8.491815000	2.868072000	12.814313000
N	9.134185000	1.770391000	13.236673000
C	9.971570000	1.925874000	14.275776000
N	10.234110000	3.118794000	14.847434000
C	9.572779000	4.169284000	14.363353000
N	8.637807000	4.114658000	13.374373000
O	9.804300000	5.379773000	14.854045000
C	10.845607000	5.542402000	15.874654000
H	10.800648000	4.682579000	16.569882000
H	10.540987000	6.458372000	16.416552000
C	12.234356000	5.706654000	15.266343000
H	12.446915000	4.797227000	14.655179000
H	12.214524000	6.555280000	14.549737000
C	13.313175000	5.938203000	16.342848000
H	13.280029000	5.101302000	17.079558000
H	13.018915000	6.833388000	16.931275000
B	14.858832000	6.153993000	15.834694000
C	15.892575000	6.611075000	16.998652000
H	15.837064000	5.943385000	17.887499000
C	15.421873000	8.017382000	17.480315000
H	16.145597000	8.423482000	18.224509000
H	14.469579000	7.889662000	18.040356000
C	15.209768000	9.071471000	16.365324000
H	14.606197000	9.913795000	16.770279000
H	16.185004000	9.524129000	16.096200000
C	14.535426000	8.537873000	15.079197000
H	14.641753000	9.303065000	14.275532000
H	13.440764000	8.441771000	15.254154000
C	15.084189000	7.166591000	14.581934000
H	14.479566000	6.895538000	13.683148000
C	16.563167000	7.264514000	14.114884000
H	16.809794000	6.354194000	13.530146000
H	16.672053000	8.105448000	13.390482000
C	17.622291000	7.434735000	15.231017000
H	17.704011000	8.505747000	15.505462000
H	18.622224000	7.169552000	14.821837000
C	17.360238000	6.607151000	16.508752000
H	18.036795000	6.975534000	17.314704000
H	17.669763000	5.553979000	16.334245000
C	7.377160000	4.197933000	9.346358000
C	6.838414000	3.282875000	8.408396000
H	6.065120000	2.561486000	8.708880000
C	7.276269000	3.293527000	7.074925000
H	6.850857000	2.575014000	6.357591000
C	8.248398000	4.219421000	6.655780000
H	8.585789000	4.227963000	5.607822000
C	8.782194000	5.137014000	7.576389000
H	9.537904000	5.869455000	7.253751000
C	8.349981000	5.125847000	8.912688000
H	8.768025000	5.842578000	9.636440000
C	5.114987000	3.718873000	11.097591000
C	4.587014000	2.956635000	12.165939000
H	5.252658000	2.582882000	12.959187000
C	3.215469000	2.663037000	12.216907000
H	2.818786000	2.055884000	13.045071000
C	2.351192000	3.143908000	11.216329000
H	1.275571000	2.913674000	11.258885000
C	2.865147000	3.917254000	10.161279000
H	2.194390000	4.295528000	9.374652000
C	4.240107000	4.202673000	10.097362000
H	4.630230000	4.796584000	9.257813000
C	7.444543000	1.412552000	11.146736000
H	8.256875000	1.190204000	10.426737000
H	6.473117000	1.390533000	10.622980000
H	7.443804000	0.636787000	11.932686000
C	10.654680000	0.726549000	14.815974000
C	10.490390000	-0.534084000	14.193748000
H	9.855764000	-0.611629000	13.299862000
C	11.133125000	-1.667234000	14.710552000
H	11.001557000	-2.643010000	14.218393000
C	11.943194000	-1.559661000	15.856377000
H	12.444289000	-2.451792000	16.262967000
C	12.109604000	-0.310642000	16.483369000
H	12.737088000	-0.225460000	17.383924000
C	11.472970000	0.826358000	15.966956000
H	11.594303000	1.803731000	16.454949000
C	6.745546000	6.936552000	14.349535000
C	7.817783000	7.699829000	13.711308000
C	7.411198000	7.970645000	12.354585000
C	6.109164000	7.362120000	12.148121000
C	5.682702000	6.752970000	13.408411000
C	6.717931000	6.519326000	15.789544000
H	6.301985000	7.339714000	16.414639000
H	7.731105000	6.293541000	16.170538000
H	6.078669000	5.629600000	15.948192000
C	8.985722000	8.339153000	14.401955000
H	8.761785000	9.409684000	14.604200000
H	9.907367000	8.305696000	13.789544000
H	9.200061000	7.860914000	15.373552000
C	8.121430000	8.874271000	11.388376000
H	7.780416000	9.922550000	11.535745000
H	7.910125000	8.605565000	10.335817000
H	9.217582000	8.857552000	11.538098000
C	5.240612000	7.585144000	10.945518000
H	4.708472000	8.556324000	11.049121000
H	4.468144000	6.801330000	10.835854000
H	5.828827000	7.630619000	10.008949000
C	4.324228000	6.185279000	13.693267000

H	3.631464000	6.991379000	14.022008000
H	4.353559000	5.428934000	14.501099000
H	3.874661000	5.711213000	12.800150000
C	17.562660000	3.329740000	11.954866000
C	16.218811000	3.739359000	11.801255000
C	18.108260000	3.264316000	13.256693000
C	15.445150000	4.082656000	12.917908000
C	17.349594000	3.613073000	14.379016000
C	16.021352000	4.039743000	14.200472000
H	15.757769000	3.784347000	10.803953000
H	19.151294000	2.930652000	13.364099000
H	14.396067000	4.391436000	12.792424000
H	17.776766000	3.554304000	15.389048000
C	18.446256000	2.943564000	10.793845000
O	19.599956000	2.570444000	10.995044000
N	15.195398000	4.433810000	15.334856000
H	14.226439000	4.170387000	15.102675000
O	15.546209000	3.714691000	16.517306000
H	15.494142000	2.763755000	16.268051000
H	8.856503000	5.804460000	11.589465000
C	17.878947000	3.030017000	9.388778000
H	18.657872000	2.728285000	8.665569000
H	16.996195000	2.366348000	9.276111000
H	17.541164000	4.062022000	9.159151000

- **[23_RhH_B_3a]$^+$ (Coordination *via* NHOH/O)**

Rh	7.950831000	6.619662000	12.092073000
P	7.258170000	4.695515000	11.276964000
N	8.357080000	3.550634000	11.967700000
C	9.348198000	4.024411000	12.778958000
N	10.214193000	3.153496000	13.309295000
C	11.194306000	3.680890000	14.068682000
N	11.362454000	5.004894000	14.255348000
C	10.486281000	5.810550000	13.658935000
N	9.390615000	5.382812000	12.982928000
O	10.659810000	7.123983000	13.692536000
C	11.904303000	7.601641000	14.299083000
H	12.010725000	7.118638000	15.289454000
H	11.729320000	8.684207000	14.442813000
C	13.113534000	7.301734000	13.409648000
H	12.910306000	6.350570000	12.882160000
H	13.201664000	8.079893000	12.623921000
C	14.410318000	7.177844000	14.225336000
H	14.163694000	6.588455000	15.138757000
H	14.683218000	8.188763000	14.601490000
B	15.708241000	6.517412000	13.516434000
C	16.307292000	7.137454000	12.152365000
H	16.296023000	8.251434000	12.160179000
C	15.361150000	6.687060000	11.001823000
H	15.785440000	7.005911000	10.022621000
H	14.408483000	7.245199000	11.104025000
C	15.046977000	5.171999000	10.933576000
H	14.143315000	5.021878000	10.303451000
H	15.861387000	4.653365000	10.390676000
C	14.831387000	4.472177000	12.295032000
H	14.881932000	3.369541000	12.148109000
H	13.799849000	4.669380000	12.655515000
C	15.821725000	4.905709000	13.407207000
H	15.471355000	4.384143000	14.330456000
C	17.275908000	4.439095000	13.134428000
H	17.882512000	4.578751000	14.059640000
H	17.297602000	3.341413000	12.949483000
C	17.990466000	5.158086000	11.968914000
H	17.653664000	4.724458000	11.007465000
H	19.079414000	4.941888000	12.017147000
C	17.772209000	6.684924000	11.927433000
H	18.155315000	7.079576000	10.959511000
H	18.437694000	7.174158000	12.685225000
C	7.395596000	4.459291000	9.487680000
C	8.498627000	3.784092000	8.926050000
H	9.253142000	3.318901000	9.576060000
C	8.646137000	3.721646000	7.531165000
H	9.507513000	3.190330000	7.099117000
C	7.702420000	4.336583000	6.690892000
H	7.821470000	4.285601000	5.598160000
C	6.609711000	5.024344000	7.248319000
H	5.873107000	5.515872000	6.595180000
C	6.457347000	5.091603000	8.640973000
H	5.608076000	5.644891000	9.067803000
C	5.656715000	4.008447000	11.783555000
C	5.410587000	4.003379000	13.175225000
H	6.171171000	4.409330000	13.859870000
C	4.208804000	3.486230000	13.675760000
H	4.023667000	3.480530000	14.760468000
C	3.239003000	2.981057000	12.788885000
H	2.290416000	2.583484000	13.180327000
C	3.484383000	2.977021000	11.405644000
H	2.732097000	2.571482000	10.712486000
C	4.693840000	3.482335000	10.899116000
H	4.886123000	3.456859000	9.817601000
C	8.178645000	2.102089000	11.808775000
H	9.110985000	1.636552000	11.439577000
H	7.368207000	1.925641000	11.080078000
H	7.904727000	1.637472000	12.775351000
C	12.172594000	2.771019000	14.698147000
C	12.092283000	1.375380000	14.486056000

H	11.281224000	0.977298000	13.860630000
C	13.040667000	0.519914000	15.061039000
H	12.975442000	-0.565042000	14.889547000
C	14.076939000	1.046743000	15.854172000
H	14.823583000	0.374062000	16.302932000
C	14.158067000	2.434054000	16.072488000
H	14.970257000	2.844465000	16.691254000
C	13.214141000	3.294984000	15.499173000
H	13.273085000	4.380853000	15.655804000
C	7.676636000	8.352880000	13.621491000
C	8.084208000	8.859271000	12.331299000
C	7.066378000	8.464111000	11.369837000
C	6.035549000	7.735585000	12.096226000
C	6.417933000	7.662121000	13.488314000
C	8.355605000	8.596864000	14.931990000
H	7.723313000	9.270242000	15.549734000
H	9.341628000	9.075628000	14.806906000
H	8.489960000	7.660799000	15.509005000
C	9.260806000	9.746125000	12.061954000
H	9.007748000	10.808020000	12.270542000
H	9.583854000	9.679375000	11.005979000
H	10.122784000	9.472587000	12.697554000
C	6.986594000	8.885009000	9.933280000
H	6.380374000	9.810738000	9.832173000
H	6.514721000	8.103125000	9.308332000
H	7.990697000	9.088961000	9.516603000
C	4.714574000	7.307426000	11.537195000
H	3.968173000	8.113217000	11.705110000
H	4.330784000	6.391007000	12.022861000
H	4.765973000	7.130465000	10.447138000
C	5.584969000	7.149481000	14.622609000
H	5.048812000	7.987424000	15.120096000
H	6.204399000	6.654368000	15.395892000
H	4.820624000	6.428377000	14.278425000
C	14.659499000	7.932070000	19.061464000
C	15.384489000	8.756053000	18.168812000
C	14.706998000	6.531640000	18.876786000
C	16.133354000	8.213619000	17.124366000
C	15.435425000	5.974727000	17.820890000
C	16.151796000	6.814128000	16.940800000
H	15.341711000	9.844120000	18.325705000
H	14.153973000	5.860039000	19.548878000
H	16.692714000	8.860233000	16.435183000
H	15.449053000	4.884848000	17.669010000
C	13.870411000	8.587884000	20.154922000
O	13.833435000	9.814935000	20.251288000
N	16.934106000	6.209282000	15.934424000
H	16.552159000	5.293028000	15.663482000
O	16.916250000	6.992635000	14.719279000
H	17.822771000	6.912169000	14.343178000
H	8.941466000	6.539182000	10.865714000
C	13.122496000	7.701104000	21.135070000
H	13.822233000	7.024341000	21.667938000
H	12.595342000	8.336913000	21.868690000
H	12.388275000	7.057569000	20.607535000

- **[23_RhH_B_2a]$^+$ (Coordination *via* NH_2)**

Rh	7.686460000	5.745038000	12.474843000
P	7.008993000	4.142217000	11.068197000
N	7.755123000	2.714505000	11.731609000
C	8.604326000	2.857794000	12.795843000
N	9.243569000	1.765519000	13.235786000
C	10.062213000	1.930905000	14.288520000
N	10.311738000	3.128853000	14.855419000
C	9.659380000	4.174540000	14.349371000
N	8.737661000	4.108934000	13.348394000
O	9.885020000	5.390640000	14.828141000
C	10.908526000	5.566266000	15.864787000
H	10.841981000	4.720371000	16.575596000
H	10.598485000	6.494704000	16.381829000
C	12.312059000	5.706679000	15.284120000
H	12.543074000	4.781277000	14.711019000
H	12.314863000	6.539839000	14.548800000
C	13.362005000	5.961523000	16.382462000
H	13.317008000	5.124481000	17.123851000
H	13.032902000	6.848985000	16.965985000
B	14.918305000	6.208906000	15.930218000
C	15.889364000	6.664150000	17.161898000
H	15.800442000	5.968228000	18.033108000
C	15.395253000	8.052283000	17.660311000
H	16.080401000	8.446342000	18.446357000
H	14.419185000	7.905616000	18.172764000
C	15.225681000	9.130723000	16.560686000
H	14.594176000	9.955869000	16.958195000
H	16.207794000	9.601361000	16.353665000
C	14.620120000	8.626630000	15.228303000
H	14.767046000	9.412716000	14.451601000
H	13.517442000	8.528619000	15.343645000
C	15.185384000	7.265861000	14.725660000
H	14.619253000	7.014292000	13.797625000
C	16.681903000	7.354056000	14.321391000
H	16.946075000	6.433316000	13.758324000
H	16.832696000	8.186687000	13.595129000
C	17.688262000	7.525366000	15.484151000
H	17.737076000	8.592140000	15.781146000
H	18.710971000	7.283671000	15.119254000

C	17.378570000	6.672051000	16.733591000
H	18.024615000	7.013956000	17.574868000
H	17.710883000	5.623046000	16.542603000
C	7.520159000	4.161641000	9.305546000
C	6.992998000	3.242776000	8.364751000
H	6.216877000	2.521826000	8.658975000
C	7.446264000	3.248711000	7.036390000
H	7.029633000	2.527187000	6.316932000
C	8.422896000	4.173381000	6.625095000
H	8.772665000	4.177971000	5.581178000
C	8.945491000	5.094674000	7.548454000
H	9.704828000	5.826235000	7.232307000
C	8.497442000	5.088444000	8.879510000
H	8.906652000	5.808472000	9.605025000
C	5.243540000	3.673723000	11.036641000
C	4.711998000	2.910264000	12.102339000
H	5.373457000	2.543422000	12.902258000
C	3.342304000	2.606513000	12.142347000
H	2.943132000	1.998603000	12.968762000
C	2.483048000	3.078118000	11.133084000
H	1.408876000	2.839816000	11.166986000
C	3.000268000	3.852516000	10.080431000
H	2.333529000	4.223672000	9.287030000
C	4.373537000	4.148365000	10.027719000
H	4.766178000	4.742954000	9.189813000
C	7.590025000	1.385470000	11.123508000
H	8.423071000	1.156524000	10.429839000
H	6.634597000	1.359112000	10.571463000
H	7.565393000	0.616143000	11.915485000
C	10.738608000	0.737227000	14.848911000
C	10.570635000	-0.531942000	14.245211000
H	9.937085000	-0.620297000	13.351594000
C	11.208017000	-1.659631000	14.780066000
H	11.073506000	-2.642198000	14.302417000
C	12.017380000	-1.537704000	15.924967000
H	12.515465000	-2.425270000	16.345008000
C	12.188065000	-0.280024000	16.533082000
H	12.817151000	-0.183215000	17.431266000
C	11.555750000	0.851163000	15.999255000
H	11.681500000	1.835881000	16.471006000
C	6.803678000	6.916118000	14.285896000
C	7.885239000	7.683821000	13.668491000
C	7.505102000	7.953351000	12.304399000
C	6.210874000	7.338278000	12.071270000
C	5.760752000	6.729254000	13.324183000
C	6.753561000	6.497690000	15.725043000
H	6.353291000	7.326688000	16.348926000
H	7.758381000	6.246871000	16.113127000
H	6.092207000	5.623085000	15.876351000
C	9.036346000	8.329177000	14.381498000
H	8.812770000	9.404309000	14.557974000
H	9.975530000	8.280126000	13.797422000
H	9.219829000	7.867289000	15.367336000
C	8.232596000	8.859888000	11.353799000
H	7.885390000	9.907008000	11.494849000
H	8.044513000	8.591630000	10.296738000
H	9.325404000	8.846366000	11.526514000
C	5.365830000	7.554210000	10.850716000
H	4.804766000	8.509134000	10.952769000
H	4.618169000	6.751135000	10.712038000
H	5.976158000	7.627094000	9.930222000
C	4.398500000	6.156763000	13.579774000
H	3.692071000	6.962883000	13.877875000
H	4.410357000	5.411518000	14.398231000
H	3.976360000	5.667759000	12.681342000
C	16.935966000	3.307623000	11.706916000
C	15.605129000	3.760617000	11.848396000
C	17.763558000	3.269283000	12.851942000
C	15.114165000	4.169585000	13.096509000
C	17.280394000	3.673067000	14.100200000
C	15.953423000	4.129295000	14.223599000
H	14.933370000	3.799795000	10.978523000
H	18.796226000	2.908069000	12.734115000
H	14.077918000	4.522959000	13.194440000
H	17.934523000	3.632920000	14.985646000
C	17.519535000	2.852115000	10.393297000
O	18.683182000	2.459210000	10.336877000
N	15.446589000	4.550944000	15.518882000
H	14.612085000	3.996923000	15.743590000
H	8.962465000	5.790343000	11.553915000
C	16.641308000	2.889578000	9.155271000
H	17.221541000	2.520422000	8.290415000
H	15.737741000	2.259123000	9.289686000
H	16.289791000	3.922148000	8.949275000
H	16.140523000	4.316059000	16.238354000

- **$[23\_RhH\_B\_3e]^+$ (Coordination *via* NHOH)**

Rh	7.592677000	5.763653000	12.539088000
P	6.902803000	4.177684000	11.119191000
N	7.644680000	2.739742000	11.767702000
C	8.502300000	2.870212000	12.826014000
N	9.145119000	1.772569000	13.248213000
C	9.976227000	1.926420000	14.292289000
N	10.233670000	3.118292000	14.868930000
C	9.571795000	4.168545000	14.384952000
N	8.641299000	4.115069000	13.392101000

O	9.799071000	5.378600000	14.879785000
C	10.843294000	5.542239000	15.896034000
H	10.801557000	4.683480000	16.592838000
H	10.541685000	6.459148000	16.437722000
C	12.229009000	5.705621000	15.280078000
H	12.439146000	4.793910000	14.671500000
H	12.204124000	6.551082000	14.560014000
C	13.314971000	5.942792000	16.347949000
H	13.292501000	5.106745000	17.086055000
H	13.021553000	6.836734000	16.939097000
B	14.852203000	6.170392000	15.827658000
C	15.900272000	6.628702000	16.974925000
H	15.852970000	5.968406000	17.869593000
C	15.436851000	8.040286000	17.450195000
H	16.168979000	8.450432000	18.183670000
H	14.490197000	7.917814000	18.020767000
C	15.214467000	9.085556000	16.329105000
H	14.616580000	9.931837000	16.733904000
H	16.187101000	9.534684000	16.045288000
C	14.524327000	8.542361000	15.055553000
H	14.621721000	9.300103000	14.244002000
H	13.431840000	8.448422000	15.244502000
C	15.066115000	7.165872000	14.562420000
H	14.450145000	6.889171000	13.673324000
C	16.539727000	7.257536000	14.077974000
H	16.778522000	6.342301000	13.497225000
H	16.641481000	8.091605000	13.345030000
C	17.611261000	7.434492000	15.180885000
H	17.696963000	8.507246000	15.446634000
H	18.606548000	7.165699000	14.762881000
C	17.361976000	6.616745000	16.467345000
H	18.049286000	6.988082000	17.262581000
H	17.665789000	5.560897000	16.297278000
C	7.410033000	4.209636000	9.356039000
C	6.877853000	3.299721000	8.409324000
H	6.099760000	2.579309000	8.699581000
C	7.329040000	3.314058000	7.080347000
H	6.908819000	2.599395000	6.356158000
C	8.308396000	4.238268000	6.674459000
H	8.656793000	4.249202000	5.630144000
C	8.835733000	5.150869000	7.603728000
H	9.597357000	5.881825000	7.291766000
C	8.389831000	5.136283000	8.935423000
H	8.802985000	5.849285000	9.665629000
C	5.135415000	3.717615000	11.089118000
C	4.602895000	2.948875000	12.150530000
H	5.264500000	2.573337000	12.946292000
C	3.231942000	2.651022000	12.191414000
H	2.831826000	2.038886000	13.014239000
C	2.372588000	3.133931000	11.187581000

H	1.297426000	2.900264000	11.222196000
C	2.890892000	3.913763000	10.139487000
H	2.224043000	4.293759000	9.350382000
C	4.265361000	4.203725000	10.085729000
H	4.658855000	4.802726000	9.251374000
C	7.470193000	1.416897000	11.148598000
H	8.296600000	1.191570000	10.445887000
H	6.510042000	1.398934000	10.604489000
H	7.449352000	0.640464000	11.933715000
C	10.658738000	0.726614000	14.832354000
C	10.503823000	-0.531353000	14.202393000
H	9.877251000	-0.606327000	13.302631000
C	11.145591000	-1.665130000	14.719065000
H	11.021574000	-2.638718000	14.220671000
C	11.945026000	-1.560973000	15.872629000
H	12.444987000	-2.453722000	16.279253000
C	12.101862000	-0.314660000	16.507484000
H	12.720374000	-0.232390000	17.414519000
C	11.466423000	0.823024000	15.991064000
H	11.579837000	1.798285000	16.485219000
C	6.721724000	6.922858000	14.363469000
C	7.804443000	7.690874000	13.748656000
C	7.420535000	7.973682000	12.388239000
C	6.122423000	7.366770000	12.154855000
C	5.674273000	6.748985000	13.404060000
C	6.674258000	6.492378000	15.799134000
H	6.276052000	7.316545000	16.430695000
H	7.679463000	6.236979000	16.183109000
H	6.011999000	5.617413000	15.944361000
C	8.961031000	8.325190000	14.462795000
H	8.745004000	9.400976000	14.644438000
H	9.898986000	8.272778000	13.876980000
H	9.142629000	7.857671000	15.446391000
C	8.148252000	8.885758000	11.443135000
H	7.806178000	9.932977000	11.595524000
H	7.954701000	8.628408000	10.384367000
H	9.241679000	8.865929000	11.611254000
C	5.274129000	7.597901000	10.939402000
H	4.721532000	8.556743000	11.050540000
H	4.519038000	6.802266000	10.798093000
H	5.881241000	7.672303000	10.016907000
C	4.310343000	6.180609000	13.659777000
H	3.609277000	6.987339000	13.968687000
H	4.321726000	5.427483000	14.470990000
H	3.882141000	5.702619000	12.758307000
C	17.491188000	3.312612000	11.926783000
C	16.142606000	3.723829000	11.785632000
C	18.073853000	3.245654000	13.215461000
C	15.396900000	4.069958000	12.916166000
C	17.326490000	3.598007000	14.345321000

C	15.996587000	4.025279000	14.189372000
H	15.683176000	3.760619000	10.787589000
H	19.116182000	2.914486000	13.328305000
H	14.346996000	4.381169000	12.808183000
H	17.771083000	3.540876000	15.347514000
N	15.195548000	4.422275000	15.336188000
H	14.220603000	4.157617000	15.132783000
O	15.579878000	3.725931000	16.518916000
H	15.499004000	2.769849000	16.298455000
H	8.865969000	5.812557000	11.614450000
C	18.255500000	2.948429000	10.766763000
N	18.872244000	2.652451000	9.820506000

- **[23_RhH_B_(*N*-(4-propylphenyl)-hydroxylamine)]$^+$**

Rh	7.575564000	5.769993000	12.549335000
P	6.872432000	4.194808000	11.124861000
N	7.610402000	2.750338000	11.761301000
C	8.475210000	2.871524000	12.815700000
N	9.116718000	1.769228000	13.226821000
C	9.954691000	1.914331000	14.267176000
N	10.217493000	3.101072000	14.850788000
C	9.556606000	4.156958000	14.377634000
N	8.621146000	4.112156000	13.388261000
O	9.787555000	5.361881000	14.880803000
C	10.839926000	5.517343000	15.892301000
H	10.802295000	4.652294000	16.581428000
H	10.540364000	6.429099000	16.443716000
C	12.221056000	5.687108000	15.269038000
H	12.429549000	4.779085000	14.654374000
H	12.189246000	6.536307000	14.553460000
C	13.312813000	5.922748000	16.331665000
H	13.291483000	5.084656000	17.067421000
H	13.022326000	6.816965000	16.922818000
B	14.853592000	6.135754000	15.794409000
C	15.897352000	6.615895000	16.944680000
H	15.870319000	5.944967000	17.832815000
C	15.413600000	8.012207000	17.438652000
H	16.142237000	8.427823000	18.173054000
H	14.471793000	7.869538000	18.012631000
C	15.169936000	9.066168000	16.330151000
H	14.560614000	9.899035000	16.746348000
H	16.134868000	9.533305000	16.048713000
C	14.484680000	8.527009000	15.051895000
H	14.570800000	9.296381000	14.249479000
H	13.393705000	8.418368000	15.242286000
C	15.041980000	7.164553000	14.543382000
H	14.427138000	6.887090000	13.653268000
C	16.510956000	7.283579000	14.050132000
H	16.759937000	6.377333000	13.460401000
H	16.594925000	8.127506000	13.325571000
C	17.586155000	7.468986000	15.148677000
H	17.657369000	8.541534000	15.420529000
H	18.583149000	7.217493000	14.723478000
C	17.357639000	6.639752000	16.432576000
H	18.038769000	7.023955000	17.227603000
H	17.685452000	5.592821000	16.255695000
C	7.371005000	4.234675000	9.359042000
C	6.832111000	3.330891000	8.410340000
H	6.054287000	2.610357000	8.701093000
C	7.276091000	3.351583000	7.079000000
H	6.850563000	2.641660000	6.353227000
C	8.254926000	4.276239000	6.672820000
H	8.597536000	4.292339000	5.626642000
C	8.789093000	5.182667000	7.604220000
H	9.550462000	5.913910000	7.292280000
C	8.350365000	5.161615000	8.938240000
H	8.768928000	5.869576000	9.670292000
C	5.102677000	3.743026000	11.099450000
C	4.571097000	2.971171000	12.159041000
H	5.234463000	2.588797000	12.950079000
C	3.199017000	2.679120000	12.204082000
H	2.799611000	2.064583000	13.025469000
C	2.337662000	3.170918000	11.206292000
H	1.261619000	2.941821000	11.244154000
C	2.855135000	3.953890000	10.160121000
H	2.186731000	4.340898000	9.375735000
C	4.230647000	4.238070000	10.102223000
H	4.623426000	4.839724000	9.269418000
C	7.427638000	1.431949000	11.135518000
H	8.248099000	1.208251000	10.425296000
H	6.463256000	1.419796000	10.598663000
H	7.410653000	0.650938000	11.916185000
C	10.637893000	0.709401000	14.794866000
C	10.464744000	-0.546817000	14.166187000
H	9.823029000	-0.616729000	13.276741000
C	11.107346000	-1.685272000	14.671351000
H	10.968546000	-2.657634000	14.174399000
C	11.926641000	-1.587291000	15.811484000
H	12.427808000	-2.483542000	16.208858000
C	12.102318000	-0.342594000	16.444450000
H	12.737668000	-0.264529000	17.340106000
C	11.465339000	0.799561000	15.939996000
H	11.594605000	1.773731000	16.432314000
C	6.719584000	6.923501000	14.384337000
C	7.803128000	7.689535000	13.768502000
C	7.414039000	7.981810000	12.411589000

C	6.111859000	7.382598000	12.181139000
C	5.666627000	6.760143000	13.429105000
C	6.677207000	6.485269000	15.817804000
H	6.287200000	7.308186000	16.456071000
H	7.682975000	6.221786000	16.194762000
H	6.010668000	5.613312000	15.961608000
C	8.966486000	8.313800000	14.480312000
H	8.756427000	9.389424000	14.669793000
H	9.901035000	8.260515000	13.889209000
H	9.151397000	7.839204000	15.459836000
C	8.142035000	8.895350000	11.468093000
H	7.807866000	9.943787000	11.629461000
H	7.940426000	8.646208000	10.408857000
H	9.236286000	8.867230000	11.629576000
C	5.258829000	7.624958000	10.971119000
H	4.714593000	8.587850000	11.088462000
H	4.496533000	6.835970000	10.831034000
H	5.861506000	7.697686000	10.045558000
C	4.301219000	6.196740000	13.688186000
H	3.605484000	7.004804000	14.005611000
H	4.313071000	5.438631000	14.494751000
H	3.866286000	5.726264000	12.785966000
C	17.555283000	3.357542000	11.879413000
C	16.199188000	3.731480000	11.747938000
C	18.106192000	3.326562000	13.179605000
C	15.424591000	4.072700000	12.866417000
C	17.348612000	3.673029000	14.309114000
C	16.010791000	4.063274000	14.143455000
H	15.736756000	3.753241000	10.748114000
H	19.158442000	3.028937000	13.314844000
H	14.368388000	4.359188000	12.743747000
H	17.792291000	3.640995000	15.313334000
N	15.183798000	4.457312000	15.285907000
H	14.219344000	4.181133000	15.048461000
O	15.537295000	3.716317000	16.459537000
H	15.521480000	2.772772000	16.179218000
H	8.845223000	5.817315000	11.619804000
C	18.372946000	2.943802000	10.674308000
H	19.434969000	3.231542000	10.831973000
H	18.023893000	3.504497000	9.780250000
C	18.302559000	1.428787000	10.376910000
H	18.642164000	0.869121000	11.276332000
H	17.238586000	1.140464000	10.226206000
C	19.134824000	1.016625000	9.158386000
H	20.207876000	1.265357000	9.298556000
H	19.065699000	-0.074469000	8.971813000
H	18.791915000	1.536599000	8.239024000

- **[23_RhH_B_3e]$^+$ (Coordination *via* Nitrile)**

Rh	7.650855000	5.701473000	12.551464000
P	6.915592000	4.151480000	11.117465000
N	7.611709000	2.686019000	11.751414000
C	8.469914000	2.779748000	12.815080000
N	9.070004000	1.657307000	13.233589000
C	9.901399000	1.777206000	14.283069000
N	10.201752000	2.954980000	14.864364000
C	9.582662000	4.032418000	14.382900000
N	8.650288000	4.014706000	13.388075000
O	9.848881000	5.229618000	14.881853000
C	10.906642000	5.367788000	15.893397000
H	10.868044000	4.492877000	16.569925000
H	10.606907000	6.271930000	16.457782000
C	12.283438000	5.547985000	15.264778000
H	12.547311000	4.625837000	14.701642000
H	12.221837000	6.368215000	14.517143000
C	13.361534000	5.869424000	16.314669000
H	13.398142000	5.045459000	17.065547000
H	13.041636000	6.769058000	16.884828000
B	14.876858000	6.139368000	15.727774000
C	15.964730000	6.640402000	16.864018000
H	15.976852000	5.939887000	17.732435000
C	15.495038000	8.015553000	17.405123000
H	16.231788000	8.405877000	18.145587000
H	14.556409000	7.858730000	17.981296000
C	15.246984000	9.101053000	16.331404000
H	14.671104000	9.937681000	16.785663000
H	16.215430000	9.550889000	16.033005000
C	14.506792000	8.604054000	15.067416000
H	14.576140000	9.390657000	14.279701000
H	13.421849000	8.514495000	15.300727000
C	14.993680000	7.242824000	14.509103000
H	14.315047000	6.980121000	13.663627000
C	16.431278000	7.271250000	13.931806000
H	16.603849000	6.309626000	13.396650000
H	16.521246000	8.059939000	13.148364000
C	17.562207000	7.471457000	14.968240000
H	17.646810000	8.549217000	15.215400000
H	18.536187000	7.209831000	14.498261000
C	17.396795000	6.659395000	16.274635000
H	18.122774000	7.043831000	17.028584000
H	17.707686000	5.607015000	16.082862000
C	7.421955000	4.186872000	9.353526000
C	6.865104000	3.300720000	8.398590000
H	6.069559000	2.597313000	8.683463000
C	7.313974000	3.317310000	7.068807000
H	6.874139000	2.621282000	6.338093000

C	8.315929000	4.220387000	6.670414000
H	8.662432000	4.233475000	5.625463000
C	8.868372000	5.109171000	7.608088000
H	9.648260000	5.823395000	7.302538000
C	8.424602000	5.092142000	8.940521000
H	8.857742000	5.786360000	9.677287000
C	5.134538000	3.745336000	11.081622000
C	4.579176000	2.982622000	12.135610000
H	5.229829000	2.580926000	12.927603000
C	3.200022000	2.724981000	12.174104000
H	2.781911000	2.117517000	12.991443000
C	2.355343000	3.242382000	11.175007000
H	1.273700000	3.040418000	11.207602000
C	2.896743000	4.016481000	10.134356000
H	2.241532000	4.423580000	9.348979000
C	4.279212000	4.266269000	10.083186000
H	4.690042000	4.861751000	9.254644000
C	7.399086000	1.375978000	11.118223000
H	8.216408000	1.136146000	10.409532000
H	6.436670000	1.389592000	10.577812000
H	7.360573000	0.591567000	11.894684000
C	10.529537000	0.548777000	14.826132000
C	10.268047000	-0.711340000	14.237078000
H	9.601217000	-0.766504000	13.365235000
C	10.851826000	-1.872404000	14.761778000
H	10.640734000	-2.848366000	14.298187000
C	11.702426000	-1.792197000	15.880333000
H	12.156555000	-2.706013000	16.294214000
C	11.969168000	-0.543003000	16.470910000
H	12.632613000	-0.477192000	17.346871000
C	11.388958000	0.621040000	15.948317000
H	11.591146000	1.599573000	16.405854000
C	6.819102000	6.866251000	14.389606000
C	7.922472000	7.609276000	13.780858000
C	7.544463000	7.918213000	12.424651000
C	6.228719000	7.352345000	12.187449000
C	5.764922000	6.734203000	13.430930000
C	6.764041000	6.420523000	15.820316000
H	6.403550000	7.251882000	16.464922000
H	7.761145000	6.120381000	16.192921000
H	6.068837000	5.570455000	15.959465000
C	9.098943000	8.200993000	14.498306000
H	8.913689000	9.279883000	14.695120000
H	10.032406000	8.130006000	13.907429000
H	9.272582000	7.715106000	15.474322000
C	8.298017000	8.818185000	11.488148000
H	7.992884000	9.874296000	11.656921000
H	8.090558000	8.582281000	10.427009000
H	9.390981000	8.758830000	11.650047000
C	5.385067000	7.621307000	10.976338000
H	4.866503000	8.598083000	11.095271000
H	4.602207000	6.853402000	10.832379000
H	5.991578000	7.680590000	10.052303000
C	4.385613000	6.202209000	13.683225000
H	3.709095000	7.024157000	14.006569000
H	4.377691000	5.437134000	14.483260000
H	3.940948000	5.750013000	12.776414000
C	16.263262000	2.494926000	14.348376000
C	17.134522000	1.724302000	15.173094000
C	15.918127000	2.001685000	13.059773000
C	17.639381000	0.510538000	14.723804000
C	16.421634000	0.785272000	12.605695000
C	17.294658000	0.022920000	13.429517000
H	17.406666000	2.094379000	16.172377000
H	15.249644000	2.589930000	12.414133000
H	18.312773000	-0.077040000	15.366541000
H	16.164502000	0.410742000	11.606264000
N	17.849723000	-1.148968000	12.986120000
H	18.257593000	-1.809489000	13.647957000
O	17.384210000	-1.736287000	11.815733000
H	16.499805000	-2.114576000	12.029736000
H	8.923659000	5.722039000	11.625138000
C	15.761705000	3.739342000	14.806544000
N	15.367414000	4.767987000	15.193079000

- **$[23\_RhH\_B\_3a]^+$ (Coordination *via* Ketone)**

Rh	7.786495000	5.869579000	12.621325000
P	7.092618000	4.870891000	10.750680000
N	7.898040000	3.333175000	10.804150000
C	8.762448000	3.094647000	11.838459000
N	9.367993000	1.901787000	11.890561000
C	10.256731000	1.729440000	12.886821000
N	10.621327000	2.709800000	13.737780000
C	9.973650000	3.868852000	13.623621000
N	8.945846000	4.097119000	12.757331000
O	10.297508000	4.899466000	14.387395000
C	11.453444000	4.803169000	15.289623000
H	11.485045000	3.783626000	15.719429000
H	11.211705000	5.526171000	16.093265000
C	12.756990000	5.182448000	14.595275000
H	13.001850000	4.414141000	13.830239000
H	12.592752000	6.133760000	14.044891000
C	13.917154000	5.349099000	15.595572000
H	14.031493000	4.405961000	16.179167000
H	13.615161000	6.110160000	16.349549000
B	15.356727000	5.813478000	14.960108000

C	16.510884000	6.214847000	16.053247000
H	16.641818000	5.443143000	16.848128000
C	16.039740000	7.495849000	16.799282000
H	16.824432000	7.826037000	17.519745000
H	15.162507000	7.223144000	17.427833000
C	15.651612000	8.694422000	15.902732000
H	15.092650000	9.438766000	16.512819000
H	16.569679000	9.224338000	15.577682000
C	14.815459000	8.320008000	14.657809000
H	14.778586000	9.199792000	13.972730000
H	13.760773000	8.148093000	14.973082000
C	15.306950000	7.057060000	13.903475000
H	14.569541000	6.863228000	13.088039000
C	16.684978000	7.235547000	13.212741000
H	16.850747000	6.351515000	12.557034000
H	16.671671000	8.114704000	12.525715000
C	17.896203000	7.366602000	14.165392000
H	17.952679000	8.403687000	14.553546000
H	18.835091000	7.227723000	13.584103000
C	17.885358000	6.379441000	15.355487000
H	18.662126000	6.694407000	16.091384000
H	18.218443000	5.379924000	14.992374000
C	7.614771000	5.623947000	9.170902000
C	8.695260000	5.107070000	8.423921000
H	9.209942000	4.192160000	8.750136000
C	9.133913000	5.769270000	7.264333000
H	9.977035000	5.355721000	6.689926000
C	8.503898000	6.951808000	6.841648000
H	8.849705000	7.467570000	5.932769000
C	7.435157000	7.479502000	7.588666000
H	6.942696000	8.410626000	7.268960000
C	6.995623000	6.824725000	8.749926000
H	6.169265000	7.258697000	9.333432000
C	5.351081000	4.355840000	10.515419000
C	4.768978000	3.611103000	11.568286000
H	5.358641000	3.393735000	12.472677000
C	3.452387000	3.139137000	11.460865000
H	3.012575000	2.552420000	12.281956000
C	2.696771000	3.418422000	10.306503000
H	1.661228000	3.053973000	10.224175000
C	3.268425000	4.157793000	9.257563000
H	2.684312000	4.371048000	8.349154000
C	4.592522000	4.621446000	9.354591000
H	5.031199000	5.180032000	8.515733000
C	7.608534000	2.250193000	9.850391000
H	8.542119000	1.883878000	9.384009000
H	6.937919000	2.639368000	9.064480000
H	7.112569000	1.402339000	10.361457000
C	10.878301000	0.393926000	13.049625000
C	10.553499000	-0.661507000	12.164185000
H	9.843529000	-0.475068000	11.346249000
C	11.130787000	-1.927642000	12.330723000
H	10.871600000	-2.742453000	11.637361000
C	12.037638000	-2.158171000	13.382053000
H	12.487859000	-3.154468000	13.513202000
C	12.366660000	-1.113997000	14.266368000
H	13.073748000	-1.292545000	15.090978000
C	11.793603000	0.154496000	14.102288000
H	12.044819000	0.974634000	14.789363000
C	7.379677000	6.433817000	14.863238000
C	8.098038000	7.491901000	14.180858000
C	7.261596000	7.945936000	13.082438000
C	6.034871000	7.160159000	13.109521000
C	6.108074000	6.228083000	14.218179000
C	7.800991000	5.742425000	16.124449000
H	7.193220000	6.125587000	16.973245000
H	8.863327000	5.920938000	16.365444000
H	7.630770000	4.648619000	16.076622000
C	9.367938000	8.146938000	14.638798000
H	9.141675000	8.989511000	15.328716000
H	9.946397000	8.559815000	13.790438000
H	10.022053000	7.440655000	15.181722000
C	7.512286000	9.158381000	12.231966000
H	7.098710000	10.063940000	12.727667000
H	7.030723000	9.076219000	11.239027000
H	8.593432000	9.330666000	12.071561000
C	4.810909000	7.425319000	12.283381000
H	4.148748000	8.135739000	12.825212000
H	4.225471000	6.505101000	12.096564000
H	5.057099000	7.886913000	11.308644000
C	4.994520000	5.365683000	14.738434000
H	4.429262000	5.899991000	15.534568000
H	5.371026000	4.424987000	15.186270000
H	4.268689000	5.103253000	13.945753000
C	16.572234000	2.543542000	13.103885000
C	16.410204000	3.052406000	11.784205000
C	17.066640000	1.215562000	13.248278000
C	16.727605000	2.290591000	10.665378000
C	17.386807000	0.440148000	12.139552000
C	17.228493000	0.970362000	10.827899000
H	16.021891000	4.073518000	11.658266000
H	17.203137000	0.779100000	14.248090000
H	16.591635000	2.687525000	9.651017000
H	17.768544000	-0.584327000	12.270706000
C	16.243843000	3.378953000	14.259237000
O	15.838212000	4.559149000	14.036863000
N	17.626251000	0.211375000	9.748402000
H	17.604696000	-0.804451000	9.862795000
O	17.120922000	0.579657000	8.491292000
H	17.915504000	0.858881000	7.988702000

H	8.962330000	6.337166000	11.680805000
C	16.400534000	2.836929000	15.655352000
H	15.839160000	1.887745000	15.768995000
H	17.468191000	2.605812000	15.852756000
H	16.050768000	3.563428000	16.405261000

- **ACN**

C	8.840318000	1.278341000	11.751961000
N	8.840354000	1.278379000	10.587483000
C	8.840246000	1.278357000	13.210366000
H	9.880479000	1.278361000	13.592261000
H	8.320216000	0.377523000	13.592413000
H	8.320216000	2.179199000	13.592390000

- **H_2O**

O	7.802683000	-5.727820000	-0.068987000
H	7.802683000	-4.969949000	0.546328000
H	7.802683000	-6.485690000	0.546328000

- **H_2**

H	5.125622000	-4.042562000	-2.032042000
H	5.125622000	-4.042562000	-2.799294000

- **2a**

C	4.035412000	-3.054175000	-3.383848000
C	2.890157000	-3.533198000	-2.688494000
C	5.078947000	-2.450309000	-2.632174000
C	2.803531000	-3.409883000	-1.303916000
C	4.978570000	-2.334439000	-1.243748000
C	3.840778000	-2.810111000	-0.545418000
H	2.072606000	-4.003715000	-3.258104000
H	5.972005000	-2.073803000	-3.155972000
H	1.919009000	-3.776631000	-0.760661000
H	5.806417000	-1.859629000	-0.695798000
C	3.678731000	-2.710890000	0.934354000
O	2.663533000	-3.136121000	1.494744000
C	4.796770000	-2.071292000	1.748726000
H	4.975611000	-1.023097000	1.430137000
H	4.514299000	-2.084295000	2.817246000
H	5.753417000	-2.618144000	1.615394000

N	4.148867000	-3.213413000	-4.749033000
H	4.845542000	-2.664993000	-5.243543000
H	3.310754000	-3.424588000	-5.281710000

- **3a**

C	4.130620000	-2.774949000	-3.342651000
C	2.879842000	-3.068396000	-2.746382000
C	5.239645000	-2.461687000	-2.515160000
C	2.750685000	-3.035945000	-1.357270000
C	5.090883000	-2.429455000	-1.126851000
C	3.843563000	-2.714346000	-0.516264000
H	2.030126000	-3.328999000	-3.391414000
H	6.216774000	-2.240231000	-2.973478000
H	1.785638000	-3.265223000	-0.879797000
H	5.968251000	-2.183384000	-0.510308000
N	4.283853000	-2.708310000	-4.729961000
H	5.217260000	-2.966672000	-5.060468000
C	3.626844000	-2.696894000	0.962996000
O	2.520383000	-2.953301000	1.445431000
C	4.802768000	-2.352641000	1.867338000
H	5.194282000	-1.338070000	1.644735000
H	4.468852000	-2.391628000	2.920219000
H	5.642497000	-3.063689000	1.721649000
O	3.327015000	-3.440469000	-5.475817000
H	2.840304000	-2.744477000	-5.963295000

- **THF**

O	6.126387000	4.755869000	10.241701000
C	6.232044000	5.918386000	11.065190000
C	5.543905000	3.750982000	11.067861000
C	6.546561000	5.415753000	12.503366000
C	6.264632000	3.890094000	12.421605000
H	4.443518000	3.922963000	11.189067000
H	5.270305000	6.488624000	11.057012000
H	5.900726000	5.919740000	13.250044000
H	7.018437000	6.570493000	10.634067000
H	7.598179000	5.616697000	12.788259000
H	5.659511000	3.512090000	13.269650000
H	7.214506000	3.318085000	12.402986000
H	5.687183000	2.769471000	10.572849000

- **CH_2Cl_2**

C	-1.393375000	0.576719000	-0.894657000

H	-0.874666000	-0.394413000	-0.819320000
H	-0.684262000	1.420446000	-0.838315000
Cl	-2.524904000	0.710200000	0.491218000
Cl	-2.211992000	0.645867000	-2.489585000

- **Et_2O**

O	-1.175629000	0.166579000	-1.588468000
C	-2.380075000	-0.147570000	-0.901242000
H	-2.568371000	0.568839000	-0.064187000
H	-2.301463000	-1.163552000	-0.436262000
C	0.003051000	0.065549000	-0.797566000
H	0.011219000	-0.906283000	-0.242539000
H	0.845060000	0.032590000	-1.521369000
C	-3.534390000	-0.103333000	-1.892634000
H	-3.374534000	-0.830395000	-2.714719000
H	-4.490060000	-0.349758000	-1.387564000
H	-3.627689000	0.906906000	-2.340643000
C	0.206613000	1.228416000	0.177464000
H	-0.580753000	1.263434000	0.957869000
H	1.182947000	1.127241000	0.695216000
H	0.200476000	2.196929000	-0.363577000

- **EtOH**

C	-2.359752000	-0.438275000	-0.582857000
H	-3.464353000	-0.543758000	-0.438668000
H	-2.049505000	0.465514000	-0.000629000
O	-2.024203000	-0.310980000	-1.961450000
H	-2.481154000	0.480570000	-2.301784000
C	-1.654242000	-1.666482000	-0.026036000
H	-1.889489000	-1.802163000	1.049041000
H	-1.969367000	-2.580145000	-0.570892000
H	-0.554355000	-1.567130000	-0.132809000

- **MeOH**

C	-2.347553000	-0.400336000	-0.593565000
H	-3.413220000	-0.368046000	-0.259067000
H	-1.908613000	-1.342026000	-0.204778000
H	-1.812207000	0.445261000	-0.096660000
O	-2.200303000	-0.409015000	-2.002701000
H	-2.588819000	0.419756000	-2.339493000

- ***N*-(4-propylphenyl)-hydroxylamine**

C	8.746110000	1.331318000	11.178333000
C	7.731916000	0.627229000	11.867571000
C	9.618811000	2.127853000	11.950232000
C	7.591289000	0.713553000	13.259183000
C	9.491623000	2.232307000	13.344768000
C	8.470311000	1.526077000	14.012568000
H	7.031992000	-0.007884000	11.299402000
H	10.423791000	2.689024000	11.446700000
H	6.789484000	0.155504000	13.769912000
H	10.183719000	2.852118000	13.930920000
N	8.242246000	1.678162000	15.401263000
H	7.903663000	0.809766000	15.826558000
O	9.409028000	2.061883000	16.129876000
H	9.164843000	2.941517000	16.481466000
C	8.918933000	1.192868000	9.678843000
H	7.921036000	1.095204000	9.196421000
H	9.371344000	2.123213000	9.270244000
C	9.792321000	-0.010870000	9.261157000
H	9.343424000	-0.941703000	9.673537000
H	10.789232000	0.082656000	9.746215000
C	9.959268000	-0.140289000	7.743178000
H	10.588485000	-1.013858000	7.475122000
H	8.978137000	-0.266674000	7.237843000
H	10.439071000	0.763204000	7.310273000

- **3e**

C	8.516391000	1.188226000	11.112491000
C	7.278737000	1.151903000	11.809008000
C	9.715404000	1.360479000	11.850277000
C	7.244524000	1.286836000	13.195671000
C	9.683959000	1.499535000	13.239287000
C	8.447251000	1.470149000	13.927799000
H	6.342925000	1.010053000	11.248677000
H	10.679776000	1.380653000	11.321371000
H	6.280411000	1.254954000	13.727040000
H	10.611772000	1.621468000	13.813047000
N	8.371981000	1.691756000	15.302391000
H	7.598626000	1.209742000	15.768098000
O	9.564707000	1.437882000	16.019363000
H	9.810847000	2.318649000	16.370347000
C	8.551947000	1.046515000	9.688802000
N	8.580438000	0.930823000	8.525118000

4. XYZ Coordinates of the Optimized Structures | THF

- **[23_RhH_B]$^{+}$ (I1)**

Rh	7.687680000	5.902197000	12.600755000
P	7.129584000	4.900351000	10.686558000
N	8.083582000	3.448900000	10.711110000
C	8.952142000	3.264209000	11.749504000
N	9.688933000	2.144896000	11.763236000
C	10.584452000	2.032634000	12.759774000
N	10.812458000	3.001607000	13.670567000
C	10.032596000	4.079200000	13.598088000
N	9.012033000	4.241627000	12.712685000
O	10.217352000	5.096460000	14.429377000
C	11.308235000	5.039065000	15.402137000
H	11.396653000	4.002651000	15.781426000
H	10.960595000	5.694900000	16.223984000
C	12.623117000	5.545400000	14.818484000
H	12.958097000	4.855656000	14.015399000
H	12.440475000	6.530090000	14.336639000
C	13.711762000	5.680079000	15.896817000
H	13.870571000	4.696811000	16.408358000
H	13.358281000	6.338327000	16.726697000
B	15.151682000	6.156058000	15.470287000
C	16.262085000	6.515820000	16.535644000
H	15.926429000	6.334207000	17.581372000
C	16.496694000	8.054024000	16.388991000
H	17.295045000	8.381847000	17.093330000
H	15.570007000	8.572549000	16.726913000
C	16.835338000	8.529613000	14.959691000
H	16.752676000	9.637751000	14.918333000
H	17.897642000	8.309791000	14.734089000
C	15.933358000	7.918440000	13.864917000
H	16.361054000	8.139231000	12.860171000
H	14.945315000	8.432585000	13.888441000
C	15.671927000	6.382244000	13.996978000
H	14.922012000	6.112413000	13.221656000
C	16.924456000	5.483104000	13.768418000
H	16.579961000	4.423765000	13.749091000
H	17.348394000	5.677123000	12.756510000
C	18.037228000	5.622036000	14.829434000
H	18.625976000	6.540419000	14.634096000
H	18.758509000	4.784349000	14.708631000
C	17.520831000	5.633769000	16.284809000
H	18.344060000	5.942603000	16.969121000
H	17.263356000	4.590875000	16.580030000
C	7.610035000	5.748408000	9.141737000
C	8.772091000	5.381348000	8.428733000
H	9.380513000	4.525277000	8.754171000
C	9.171126000	6.119748000	7.301013000
H	10.077669000	5.821988000	6.751815000
C	8.420576000	7.230028000	6.877705000
H	8.735679000	7.804963000	5.993290000
C	7.269757000	7.608977000	7.592497000
H	6.681737000	8.483030000	7.272809000
C	6.867857000	6.877701000	8.721770000
H	5.975561000	7.197856000	9.280789000
C	5.453393000	4.220858000	10.403227000
C	4.927031000	3.403144000	11.431047000
H	5.515072000	3.229255000	12.345847000
C	3.665846000	2.806022000	11.286626000
H	3.269505000	2.164938000	12.089007000
C	2.910237000	3.030533000	10.120205000
H	1.917477000	2.567572000	10.009276000
C	3.427375000	3.841599000	9.095963000
H	2.843880000	4.012986000	8.178239000
C	4.696866000	4.431828000	9.229974000
H	5.094318000	5.045976000	8.409728000
C	7.926792000	2.383478000	9.707016000
H	8.900061000	2.144928000	9.239057000
H	7.225723000	2.730904000	8.928376000
H	7.520789000	1.465950000	10.174958000
C	11.380212000	0.784794000	12.851243000
C	11.196787000	-0.252683000	11.905560000
H	10.460166000	-0.120885000	11.100425000
C	11.947362000	-1.433135000	11.995492000
H	11.798417000	-2.233589000	11.254472000
C	12.889506000	-1.595056000	13.028851000
H	13.478010000	-2.523211000	13.098182000
C	13.078449000	-0.568409000	13.973037000
H	13.814369000	-0.691779000	14.782486000
C	12.330630000	0.614296000	13.886404000
H	12.476434000	1.419949000	14.619700000
C	7.222666000	6.417712000	14.847402000
C	7.785987000	7.556058000	14.155697000
C	6.889713000	7.882891000	13.057187000
C	5.773807000	6.944426000	13.104865000
C	5.985135000	6.034386000	14.209413000
C	7.726779000	5.807385000	16.119492000
H	7.030789000	6.068652000	16.946491000
H	8.729257000	6.179933000	16.393008000
H	7.760776000	4.701108000	16.067902000
C	8.964437000	8.373678000	14.599106000
H	8.649855000	9.128017000	15.353460000
H	9.421129000	8.921643000	13.752876000

H	9.748861000	7.747599000	15.063670000
C	6.961916000	9.109944000	12.193875000
H	6.399750000	9.944488000	12.667559000
H	6.517410000	8.936662000	11.195384000
H	8.005484000	9.449350000	12.050017000
C	4.518336000	7.053720000	12.292241000
H	3.786204000	7.686343000	12.840600000
H	4.041175000	6.070830000	12.119518000
H	4.696527000	7.533911000	11.311756000
C	5.013320000	5.024443000	14.748281000
H	4.429476000	5.460696000	15.589292000
H	5.527268000	4.125831000	15.142840000
H	4.286632000	4.696674000	13.981216000
H	8.835797000	6.502925000	11.700493000

- **[23_Rh_ACN_B_ACN]$^{2+}$**

Rh	7.591270000	6.104693000	12.466957000
P	7.098944000	4.694541000	10.704572000
N	7.841686000	3.213611000	11.191479000
C	8.601726000	3.203695000	12.331645000
N	9.187229000	2.056149000	12.688467000
C	9.968987000	2.101193000	13.784933000
N	10.264618000	3.241310000	14.443490000
C	9.651190000	4.345834000	14.025045000
N	8.712134000	4.371996000	13.036840000
O	9.921254000	5.520296000	14.568352000
C	10.936123000	5.610203000	15.631429000
H	10.829497000	4.728325000	16.291805000
H	10.638750000	6.518562000	16.190095000
C	12.350203000	5.743416000	15.078715000
H	12.596390000	4.831941000	14.491188000
H	12.374334000	6.596948000	14.367026000
C	13.379849000	5.958311000	16.202722000
H	13.285894000	5.129020000	16.943144000
H	13.104063000	6.877831000	16.763697000
B	14.951934000	6.082018000	15.733727000
C	15.993982000	6.463211000	16.951816000
H	15.860911000	5.767460000	17.813994000
C	15.631938000	7.882582000	17.462040000
H	16.347660000	8.198211000	18.256898000
H	14.641328000	7.827892000	17.965342000
C	15.579276000	8.985520000	16.377430000
H	15.053328000	9.875293000	16.789358000
H	16.608613000	9.337594000	16.161846000
C	14.899718000	8.564924000	15.052344000
H	15.124817000	9.335398000	14.277693000
H	13.795178000	8.597184000	15.188474000
C	15.280344000	7.155386000	14.531557000
H	14.644983000	6.960202000	13.635276000
C	16.754544000	7.029918000	14.070454000
H	16.869771000	6.051472000	13.550387000
H	16.988007000	7.800175000	13.298065000
C	17.812781000	7.119238000	15.195549000
H	17.981453000	8.183303000	15.458468000
H	18.791196000	6.763342000	14.802676000
C	17.461976000	6.326583000	16.476272000
H	18.164868000	6.628596000	17.287643000
H	17.670271000	5.246987000	16.297727000
N	15.324300000	4.652190000	15.223833000
C	15.594367000	3.582606000	14.865288000
C	15.942927000	2.248579000	14.420878000
H	15.093442000	1.558657000	14.596183000
H	16.826156000	1.883380000	14.982775000
H	16.182950000	2.260292000	13.338631000
C	7.881755000	5.195596000	9.139704000
C	8.913808000	4.441968000	8.539870000
H	9.263491000	3.505271000	8.994872000
C	9.512464000	4.895372000	7.351443000
H	10.314634000	4.300393000	6.888607000
C	9.090641000	6.097377000	6.757719000
H	9.560305000	6.446338000	5.825264000
C	8.073920000	6.859432000	7.361989000
H	7.747106000	7.807533000	6.908341000
C	7.476700000	6.418335000	8.553232000
H	6.698342000	7.034568000	9.029493000
C	5.408673000	4.150981000	10.302842000
C	4.742648000	3.319272000	11.235841000
H	5.247103000	3.010806000	12.163902000
C	3.442637000	2.864163000	10.970573000
H	2.936540000	2.209144000	11.696013000
C	2.791347000	3.240922000	9.781078000
H	1.770238000	2.884499000	9.575815000
C	3.449837000	4.063501000	8.851283000
H	2.949769000	4.351325000	7.913999000
C	4.755862000	4.516216000	9.104119000
H	5.262132000	5.142018000	8.356196000
C	7.657712000	1.948830000	10.451123000
H	8.639321000	1.529049000	10.162885000
H	7.063115000	2.148450000	9.543157000
H	7.123650000	1.210636000	11.078835000
C	10.553916000	0.835465000	14.281223000
C	10.310658000	-0.382090000	13.600155000
H	9.690153000	-0.376600000	12.693026000
C	10.857457000	-1.580269000	14.078987000
H	10.664171000	-2.522355000	13.543459000
C	11.651043000	-1.579907000	15.241758000
H	12.078297000	-2.522813000	15.617065000
C	11.897137000	-0.373984000	15.925497000

H	12.514545000	-0.372536000	16.836811000
C	11.354823000	0.827637000	15.449373000
H	11.542206000	1.771888000	15.979728000
C	6.897018000	6.810700000	14.544193000
C	7.466419000	7.916420000	13.839942000
C	6.696555000	8.102758000	12.599517000
C	5.624181000	7.136710000	12.587307000
C	5.801317000	6.265327000	13.742393000
C	7.245906000	6.324552000	15.911215000
H	6.445249000	6.653695000	16.609930000
H	8.203295000	6.734334000	16.276739000
H	7.278083000	5.218965000	15.965855000
C	8.568174000	8.814420000	14.302726000
H	8.131440000	9.733639000	14.750353000
H	9.213627000	9.135571000	13.462889000
H	9.199635000	8.331855000	15.070128000
C	6.873780000	9.236910000	11.640206000
H	6.340334000	10.131305000	12.030233000
H	6.451398000	9.003263000	10.645322000
H	7.937948000	9.514497000	11.519317000
C	4.456909000	7.139990000	11.655120000
H	3.677686000	7.807093000	12.085326000
H	4.005429000	6.138997000	11.537470000
H	4.717175000	7.537186000	10.656284000
C	4.842159000	5.225135000	14.232074000
H	4.173517000	5.677483000	14.997014000
H	5.364619000	4.376049000	14.713237000
H	4.201824000	4.834927000	13.421000000
N	9.313585000	6.719631000	11.528815000
C	10.290233000	7.112694000	11.040857000
C	11.509270000	7.601664000	10.428179000
H	12.241990000	7.879214000	11.211970000
H	11.287114000	8.491625000	9.806108000
H	11.946823000	6.811795000	9.785596000

- **[23_RhH_B_ACN]$^+$**

Rh	7.711380000	5.772591000	12.555366000
P	7.308005000	4.294510000	10.920854000
N	7.867597000	2.798906000	11.639287000
C	8.653237000	2.890757000	12.754147000
N	9.252721000	1.778065000	13.202754000
C	10.052595000	1.920464000	14.273409000
N	10.340760000	3.110675000	14.838452000
C	9.711558000	4.173281000	14.336636000
N	8.778732000	4.122929000	13.345902000
O	9.968207000	5.386560000	14.802899000
C	11.023786000	5.565444000	15.806805000
H	11.009964000	4.701846000	16.498664000
H	10.708272000	6.473014000	16.357495000
C	12.390019000	5.761097000	15.158575000
H	12.642720000	4.847307000	14.576675000
H	12.311949000	6.591138000	14.423349000
C	13.491646000	6.065790000	16.188895000
H	13.542140000	5.229808000	16.926013000
H	13.187065000	6.956726000	16.780740000
B	14.991178000	6.342336000	15.570351000
C	16.128736000	6.758599000	16.686416000
H	16.154411000	6.015996000	17.519173000
C	15.713378000	8.116317000	17.310233000
H	16.484249000	8.454232000	18.042091000
H	14.790086000	7.953114000	17.908930000
C	15.454733000	9.258091000	16.298457000
H	14.913479000	10.083204000	16.812844000
H	16.423014000	9.701543000	15.989340000
C	14.660891000	8.840184000	15.038204000
H	14.725107000	9.662145000	14.286804000
H	13.582393000	8.765289000	15.302833000
C	15.093002000	7.494770000	14.399810000
H	14.378107000	7.289363000	13.568829000
C	16.510195000	7.517169000	13.773064000
H	16.642998000	6.576247000	13.191674000
H	16.592049000	8.338122000	13.022256000
C	17.679891000	7.644943000	14.777534000
H	17.795723000	8.707287000	15.074774000
H	18.631760000	7.387344000	14.261605000
C	17.539730000	6.771319000	16.046568000
H	18.303538000	7.097260000	16.791215000
H	17.812698000	5.722394000	15.789207000
C	8.190994000	4.381326000	9.311091000
C	7.914775000	3.465863000	8.265121000
H	7.120746000	2.712964000	8.370820000
C	8.644565000	3.516745000	7.067062000
H	8.420636000	2.797154000	6.264493000
C	9.650385000	4.484245000	6.889767000
H	10.217246000	4.523892000	5.946632000
C	9.924007000	5.403011000	7.917093000
H	10.703980000	6.168432000	7.783881000
C	9.199886000	5.350528000	9.119922000
H	9.416026000	6.068448000	9.926560000
C	5.586880000	3.888746000	10.478124000
C	4.799888000	3.133191000	11.378611000
H	5.247351000	2.737039000	12.303178000
C	3.450899000	2.872576000	11.091072000
H	2.851056000	2.270791000	11.791066000
C	2.868625000	3.377117000	9.913087000
H	1.810502000	3.171174000	9.689242000
C	3.640805000	4.143586000	9.023263000
H	3.189944000	4.543529000	8.102020000

C	4.994354000	4.400618000	9.301328000
H	5.587649000	5.000666000	8.595821000
C	7.724852000	1.489474000	10.984459000
H	8.645171000	1.212903000	10.433273000
H	6.873221000	1.528807000	10.284146000
H	7.519403000	0.715556000	11.745818000
C	10.674129000	0.703865000	14.852487000
C	10.434226000	-0.567279000	14.277605000
H	9.786508000	-0.642177000	13.392769000
C	11.017262000	-1.716082000	14.830438000
H	10.824483000	-2.699941000	14.375563000
C	11.846199000	-1.613182000	15.963492000
H	12.302484000	-2.516734000	16.397104000
C	12.089920000	-0.353242000	16.541760000
H	12.735823000	-0.269167000	17.429362000
C	11.509733000	0.798489000	15.991329000
H	11.695691000	1.784110000	16.440718000
C	6.904898000	6.777652000	14.487383000
C	7.737104000	7.703250000	13.743481000
C	7.124471000	7.884880000	12.439288000
C	5.904304000	7.090614000	12.410178000
C	5.769620000	6.402419000	13.678309000
C	7.101792000	6.360848000	15.913666000
H	6.401987000	6.930585000	16.563416000
H	8.128669000	6.560842000	16.266693000
H	6.878193000	5.286273000	16.063967000
C	8.889348000	8.499900000	14.280240000
H	8.526009000	9.470621000	14.683925000
H	9.634776000	8.727859000	13.494264000
H	9.407848000	7.975097000	15.103034000
C	7.535240000	8.893935000	11.405376000
H	7.028242000	9.864774000	11.598740000
H	7.257218000	8.573916000	10.382959000
H	8.626175000	9.078766000	11.423830000
C	4.854540000	7.170425000	11.344907000
H	4.190261000	8.035959000	11.561632000
H	4.218813000	6.266552000	11.312478000
H	5.287595000	7.332375000	10.339611000
C	4.586718000	5.603000000	14.142371000
H	3.839037000	6.262288000	14.637020000
H	4.878490000	4.830147000	14.879983000
H	4.073159000	5.097952000	13.301784000
H	9.069158000	6.052165000	11.803458000
C	15.703178000	3.947420000	14.490308000
N	15.414243000	4.974660000	14.944989000
C	16.070084000	2.667713000	13.918829000
H	15.301953000	1.909225000	14.170152000
H	17.048158000	2.339185000	14.324376000
H	16.146550000	2.753819000	12.816206000

- **[23_RhH_B_THF]$^+$**

Rh	7.623756000	5.792346000	12.461002000
P	7.087757000	4.332343000	10.849902000
N	7.585077000	2.810219000	11.557445000
C	8.401221000	2.860945000	12.653067000
N	8.940928000	1.716841000	13.098024000
C	9.772619000	1.817348000	14.148923000
N	10.149999000	2.991992000	14.694302000
C	9.574905000	4.087351000	14.197947000
N	8.614854000	4.086798000	13.232332000
O	9.917895000	5.286801000	14.643868000
C	10.987278000	5.411873000	15.640133000
H	10.946190000	4.541591000	16.322574000
H	10.713602000	6.325398000	16.203610000
C	12.357403000	5.564641000	14.987398000
H	12.563834000	4.654252000	14.383656000
H	12.310513000	6.415204000	14.272995000
C	13.467224000	5.801335000	16.032052000
H	13.575542000	4.882067000	16.655059000
H	13.096551000	6.566586000	16.750768000
B	14.930696000	6.312406000	15.513389000
C	15.961683000	6.741270000	16.701702000
H	16.047831000	5.952336000	17.487203000
C	15.359681000	7.986909000	17.414535000
H	16.063061000	8.361656000	18.194825000
H	14.452898000	7.662029000	17.971154000
C	14.976636000	9.165702000	16.485272000
H	14.296983000	9.854248000	17.035157000
H	15.880162000	9.771355000	16.269392000
C	14.308266000	8.755645000	15.150803000
H	14.298373000	9.639151000	14.470129000
H	13.237926000	8.519199000	15.342944000
C	14.951330000	7.532655000	14.437351000
H	14.310551000	7.326461000	13.547297000
C	16.380516000	7.813809000	13.902459000
H	16.681564000	6.967069000	13.246050000
H	16.376137000	8.709212000	13.236639000
C	17.468804000	8.001239000	14.983320000
H	17.417807000	9.033012000	15.386557000
H	18.471938000	7.930598000	14.506324000
C	17.389030000	6.991251000	16.149661000
H	18.067551000	7.332883000	16.966513000
H	17.812091000	6.020259000	15.805717000
C	7.919836000	4.374106000	9.211320000
C	7.579297000	3.457447000	8.185483000
H	6.768892000	2.728515000	8.327285000
C	8.265127000	3.477073000	6.960834000
H	7.992162000	2.756422000	6.174574000
C	9.289318000	4.415080000	6.736269000

H	9.821439000	4.430186000	5.772417000
C	9.625360000	5.336166000	7.742743000
H	10.419609000	6.079147000	7.572136000
C	8.945857000	5.314413000	8.972296000
H	9.210192000	6.034326000	9.762504000
C	5.333193000	4.022962000	10.465376000
C	4.531045000	3.331241000	11.403082000
H	4.982720000	2.921296000	12.319536000
C	3.159815000	3.151673000	11.163003000
H	2.546966000	2.599194000	11.891805000
C	2.571665000	3.674373000	9.995753000
H	1.495875000	3.532393000	9.809562000
C	3.360389000	4.377025000	9.068680000
H	2.905356000	4.790771000	8.155625000
C	4.735757000	4.552785000	9.299349000
H	5.341975000	5.103756000	8.565392000
C	7.338736000	1.507666000	10.920015000
H	8.219808000	1.170482000	10.339287000
H	6.466108000	1.594847000	10.250365000
H	7.113328000	0.753378000	11.695271000
C	10.325263000	0.568151000	14.728295000
C	9.981111000	-0.690524000	14.179313000
H	9.305112000	-0.731503000	13.313661000
C	10.497452000	-1.869988000	14.733662000
H	10.223600000	-2.843795000	14.299200000
C	11.362900000	-1.810546000	15.842302000
H	11.766939000	-2.738129000	16.276980000
C	11.710352000	-0.563365000	16.394474000
H	12.385517000	-0.513271000	17.262628000
C	11.196566000	0.618869000	15.842815000
H	11.462858000	1.594975000	16.272079000
C	6.998081000	6.901565000	14.404547000
C	7.807307000	7.761625000	13.567398000
C	7.108229000	7.915008000	12.301748000
C	5.856283000	7.173986000	12.396515000
C	5.792816000	6.537044000	13.692862000
C	7.267721000	6.541686000	15.834077000
H	6.568354000	7.104807000	16.489853000
H	8.297100000	6.794480000	16.143149000
H	7.094246000	5.465154000	16.031763000
C	9.035931000	8.513550000	13.986971000
H	8.752927000	9.486684000	14.445242000
H	9.697326000	8.732817000	13.126947000
H	9.625058000	7.953912000	14.736635000
C	7.477134000	8.864049000	11.197400000
H	7.009055000	9.857283000	11.374337000
H	7.126655000	8.503897000	10.211126000
H	8.571579000	9.017514000	11.138082000
C	4.733004000	7.261721000	11.410258000
H	4.120208000	8.159132000	11.648317000
H	4.062611000	6.383407000	11.453289000
H	5.092494000	7.377585000	10.370332000
C	4.624258000	5.796685000	14.275565000
H	3.939151000	6.497676000	14.802570000
H	4.948318000	5.039630000	15.016082000
H	4.030265000	5.281325000	13.496423000
C	15.304959000	4.572513000	13.371921000
O	15.643002000	4.976086000	14.750015000
C	15.245165000	3.049265000	13.415418000
C	16.025254000	3.798733000	15.543300000
C	16.272354000	2.707231000	14.507608000
H	16.116144000	4.944453000	12.713113000
H	14.232152000	2.705786000	13.711756000
H	15.184645000	3.554719000	16.224231000
H	16.914812000	4.073405000	16.139666000
H	16.133914000	1.696199000	14.937289000
H	17.305491000	2.774661000	14.108459000
H	15.486717000	2.601204000	12.432057000
H	14.356296000	5.064603000	13.096500000
H	8.964220000	5.976037000	11.651428000

- **[23_RhH_B_3a]$^{+}$ (Coordination *via* NHOH/N)**

Rh	7.554381000	5.778308000	12.538560000
P	6.932895000	4.184911000	11.092909000
N	7.636180000	2.748894000	11.794424000
C	8.472089000	2.895483000	12.864832000
N	9.092702000	1.802097000	13.332135000
C	9.915584000	1.981450000	14.378490000
N	10.201293000	3.189910000	14.904333000
C	9.561675000	4.233436000	14.376350000
N	8.617586000	4.152731000	13.399074000
O	9.824666000	5.460540000	14.805597000
C	10.859537000	5.648856000	15.827459000
H	10.792903000	4.821410000	16.559225000
H	10.567579000	6.593870000	16.325345000
C	12.255156000	5.759041000	15.221616000
H	12.446118000	4.827558000	14.638454000
H	12.259521000	6.587562000	14.481653000
C	13.336282000	5.991263000	16.296051000
H	13.285258000	5.166147000	17.045236000
H	13.055291000	6.900750000	16.869010000
B	14.885640000	6.163945000	15.782764000
C	15.931941000	6.618065000	16.940953000
H	15.869343000	5.958388000	17.835774000
C	15.486250000	8.034605000	17.414814000
H	16.219233000	8.435237000	18.153809000

H	14.533604000	7.925391000	17.977864000
C	15.285834000	9.085105000	16.293838000
H	14.693751000	9.937363000	16.695575000
H	16.266857000	9.523311000	16.020506000
C	14.601354000	8.552506000	15.012350000
H	14.718859000	9.311219000	14.203618000
H	13.506227000	8.474469000	15.189544000
C	15.126577000	7.169522000	14.524237000
H	14.515618000	6.901090000	13.629630000
C	16.605144000	7.241279000	14.052359000
H	16.836224000	6.322264000	13.475251000
H	16.725036000	8.074858000	13.320496000
C	17.669686000	7.403498000	15.164518000
H	17.766678000	8.474815000	15.433608000
H	18.665191000	7.121180000	14.755020000
C	17.397950000	6.586076000	16.446877000
H	18.084716000	6.946660000	17.248600000
H	17.687830000	5.527079000	16.274353000
C	7.537723000	4.190951000	9.358226000
C	7.057063000	3.269235000	8.395324000
H	6.258517000	2.557291000	8.648172000
C	7.587454000	3.260330000	7.095517000
H	7.205965000	2.536760000	6.358687000
C	8.596338000	4.171768000	6.734855000
H	9.007091000	4.163836000	5.713268000
C	9.073208000	5.095830000	7.680295000
H	9.857775000	5.817476000	7.405079000
C	8.547173000	5.105109000	8.982689000
H	8.920739000	5.828337000	9.724229000
C	5.170237000	3.723363000	10.976693000
C	4.582958000	2.982595000	12.029452000
H	5.200464000	2.635941000	12.872338000
C	3.213593000	2.675464000	12.000895000
H	2.770561000	2.086687000	12.818849000
C	2.411299000	3.117930000	10.932697000
H	1.337530000	2.875620000	10.911962000
C	2.984387000	3.869958000	9.892737000
H	2.361982000	4.221716000	9.055588000
C	4.356896000	4.172675000	9.910992000
H	4.790650000	4.759955000	9.088449000
C	7.465105000	1.411152000	11.206804000
H	8.349057000	1.123656000	10.604346000
H	6.568039000	1.412307000	10.564884000
H	7.322751000	0.666224000	12.010297000
C	10.560277000	0.787483000	14.978511000
C	10.330599000	-0.499751000	14.436148000
H	9.674808000	-0.603446000	13.560219000
C	10.933572000	-1.627895000	15.009653000
H	10.748659000	-2.624495000	14.579943000
C	11.771737000	-1.488135000	16.131924000

H	12.243148000	-2.375563000	16.582276000
C	12.005041000	-0.212056000	16.678199000
H	12.657485000	-0.099572000	17.557847000
C	11.405630000	0.919185000	16.106410000
H	11.582973000	1.917166000	16.531435000
C	6.869674000	6.998920000	14.403796000
C	7.757851000	7.792264000	13.569955000
C	7.130931000	7.918581000	12.268294000
C	5.860004000	7.212974000	12.317978000
C	5.698100000	6.645199000	13.645596000
C	7.058778000	6.700758000	15.860878000
H	6.369985000	7.337477000	16.457827000
H	8.088770000	6.911777000	16.198034000
H	6.816411000	5.647800000	16.106465000
C	8.971813000	8.546775000	14.024426000
H	8.679092000	9.554717000	14.392942000
H	9.696392000	8.695467000	13.200751000
H	9.494200000	8.032575000	14.850999000
C	7.591982000	8.804767000	11.146732000
H	7.144769000	9.817288000	11.254464000
H	7.286175000	8.412229000	10.158075000
H	8.692065000	8.924640000	11.144970000
C	4.795629000	7.294124000	11.266551000
H	4.218183000	8.233487000	11.413103000
H	4.079146000	6.454526000	11.327526000
H	5.216635000	7.324501000	10.243506000
C	4.467861000	5.974401000	14.182587000
H	3.749120000	6.730579000	14.569657000
H	4.707257000	5.291124000	15.020279000
H	3.941346000	5.389183000	13.403942000
C	17.519264000	3.299795000	11.910651000
C	16.180040000	3.728982000	11.764524000
C	18.071220000	3.226616000	13.210011000
C	15.418854000	4.086304000	12.885201000
C	17.323511000	3.586032000	14.336316000
C	16.001993000	4.035416000	14.164252000
H	15.712382000	3.778615000	10.770622000
H	19.108779000	2.875966000	13.315835000
H	14.372807000	4.407341000	12.766170000
H	17.752514000	3.513884000	15.344578000
C	18.384948000	2.901015000	10.743343000
O	19.542204000	2.523241000	10.931061000
N	15.188255000	4.445152000	15.303623000
H	14.216342000	4.182675000	15.081569000
O	15.545314000	3.732901000	16.487922000
H	15.422743000	2.781246000	16.266227000
H	8.848912000	5.887269000	11.646585000
C	17.800170000	2.978049000	9.346393000
H	18.564845000	2.662587000	8.613691000
H	16.908757000	2.322895000	9.254518000

H	17.466446000	4.010386000	9.112550000

- **[23_RhH_B_3a]⁺ (Coordination *via* NHOH/O)**

Rh	7.149336000	5.849095000	12.410293000
P	6.631478000	4.359112000	10.869436000
N	7.807969000	3.112514000	11.107952000
C	8.755125000	3.305083000	12.070576000
N	9.693157000	2.366348000	12.245987000
C	10.632544000	2.632862000	13.173491000
N	10.689958000	3.786341000	13.868592000
C	9.737913000	4.682989000	13.619108000
N	8.683848000	4.472134000	12.792423000
O	9.782457000	5.880764000	14.185719000
C	10.961455000	6.195617000	14.991027000
H	11.147498000	5.350196000	15.681275000
H	10.656604000	7.083055000	15.577230000
C	12.176896000	6.457355000	14.107854000
H	12.211224000	5.648389000	13.354513000
H	12.032829000	7.404987000	13.548876000
C	13.490131000	6.480545000	14.912864000
H	13.421989000	5.681933000	15.691009000
H	13.517101000	7.439088000	15.481582000
B	14.862357000	6.237123000	14.082883000
C	15.321545000	7.223063000	12.899710000
H	15.119956000	8.296502000	13.128169000
C	14.435111000	6.860542000	11.672005000
H	14.748797000	7.470782000	10.794532000
H	13.391218000	7.167269000	11.887582000
C	14.451235000	5.367611000	11.265871000
H	13.597940000	5.171935000	10.580140000
H	15.358189000	5.167515000	10.662312000
C	14.392823000	4.359811000	12.437417000
H	14.683922000	3.351838000	12.062519000
H	13.340242000	4.247193000	12.771123000
C	15.262092000	4.731682000	13.670670000
H	15.003542000	4.001007000	14.472892000
C	16.781114000	4.607392000	13.394656000
H	17.321802000	4.706120000	14.360458000
H	17.017384000	3.584716000	13.020388000
C	17.345238000	5.644339000	12.398263000
H	17.116848000	5.316157000	11.364669000
H	18.455444000	5.642329000	12.462477000
C	16.835802000	7.088752000	12.600268000
H	17.098247000	7.690243000	11.699548000
H	17.402048000	7.550327000	13.434964000
C	6.822300000	4.862351000	9.140092000
C	7.972030000	4.514124000	8.401504000
H	8.740598000	3.863822000	8.842511000
C	8.148147000	5.015725000	7.101555000
H	9.046220000	4.737397000	6.529528000
C	7.186284000	5.869708000	6.535097000
H	7.327911000	6.260559000	5.516075000
C	6.046162000	6.230908000	7.275455000
H	5.294377000	6.907181000	6.841390000
C	5.864872000	5.735079000	8.575074000
H	4.979037000	6.038287000	9.151821000
C	5.083283000	3.421324000	10.985109000
C	4.827912000	2.819455000	12.238092000
H	5.553521000	2.941448000	13.056923000
C	3.659211000	2.072184000	12.433922000
H	3.466801000	1.602730000	13.410425000
C	2.732231000	1.930333000	11.383619000
H	1.809220000	1.351464000	11.539741000
C	2.987970000	2.520874000	10.134576000
H	2.270172000	2.401250000	9.309050000
C	4.164725000	3.260960000	9.928503000
H	4.365642000	3.702356000	8.942702000
C	7.750397000	1.833457000	10.388411000
H	8.716185000	1.624856000	9.892241000
H	6.954070000	1.895496000	9.626454000
H	7.518521000	1.008035000	11.088300000
C	11.691151000	1.631156000	13.419809000
C	11.707714000	0.412736000	12.702450000
H	10.910940000	0.209488000	11.973459000
C	12.733773000	-0.517027000	12.917599000
H	12.742573000	-1.462603000	12.354578000
C	13.753304000	-0.241377000	13.848094000
H	14.560888000	-0.971252000	14.012245000
C	13.740412000	0.967885000	14.567989000
H	14.537186000	1.186811000	15.294780000
C	12.716237000	1.899607000	14.357279000
H	12.702758000	2.850330000	14.907199000
C	6.710371000	6.788516000	14.490481000
C	7.082417000	7.803181000	13.531908000
C	6.111300000	7.752837000	12.448926000
C	5.142655000	6.715702000	12.774590000
C	5.519687000	6.114238000	14.034185000
C	7.354529000	6.527127000	15.814967000
H	6.654116000	6.825033000	16.624569000
H	8.288875000	7.099868000	15.941422000
H	7.577118000	5.451609000	15.959646000
C	8.174893000	8.816019000	13.688680000
H	7.808471000	9.702523000	14.250227000
H	8.540533000	9.168822000	12.705787000
H	9.035276000	8.398401000	14.242848000
C	6.007448000	8.715595000	11.304462000
H	5.326096000	9.554125000	11.564681000

H	5.605517000	8.226279000	10.396997000
H	6.993669000	9.147350000	11.049665000
C	3.871979000	6.446487000	12.031217000
H	3.052697000	7.048493000	12.479634000
H	3.572978000	5.383469000	12.085204000
H	3.948862000	6.735565000	10.967003000
C	4.733553000	5.114152000	14.824300000
H	4.144412000	5.623590000	15.618321000
H	5.392459000	4.378876000	15.326447000
H	4.020104000	4.560082000	14.186973000
C	19.855645000	9.803240000	15.525577000
C	19.780488000	8.406158000	15.737773000
C	18.649598000	10.536536000	15.430527000
C	18.553333000	7.754264000	15.856427000
C	17.410893000	9.896293000	15.532534000
C	17.358103000	8.501242000	15.748397000
H	20.723147000	7.843383000	15.808433000
H	18.668969000	11.621870000	15.256528000
H	18.504705000	6.668863000	16.016542000
H	16.476223000	10.470036000	15.437345000
C	21.206830000	10.435673000	15.405800000
O	22.231112000	9.752775000	15.493757000
N	16.100197000	7.901161000	15.940829000
H	15.338249000	8.401950000	15.469392000
O	16.060934000	6.554155000	15.432239000
H	15.793771000	6.005097000	16.201930000
H	8.154867000	6.347350000	11.295215000
C	21.285327000	11.933349000	15.174111000
H	20.791757000	12.488515000	15.998540000
H	22.346089000	12.235837000	15.109810000
H	20.762645000	12.216216000	14.236868000

- **ACN**

C	8.840323000	1.278352000	11.754433000
N	8.840325000	1.278368000	10.589499000
C	8.840258000	1.278359000	13.211103000
H	9.881372000	1.278361000	13.590564000
H	8.319775000	0.376743000	13.590642000
H	8.319775000	2.179978000	13.590633000

- **H_2O**

O	7.802683000	-5.727820000	-0.070265000
H	7.802683000	-4.970694000	0.546967000
H	7.802683000	-6.484945000	0.546967000

- **H_2**

H	5.125622000	-4.042562000	-2.031947000
H	5.125622000	-4.042562000	-2.799389000

- **2a**

C	4.033155000	-3.050528000	-3.384391000
C	2.886639000	-3.531140000	-2.688349000
C	5.077208000	-2.446639000	-2.629926000
C	2.800985000	-3.409782000	-1.303939000
C	4.976884000	-2.333072000	-1.242048000
C	3.838530000	-2.810664000	-0.542854000
H	2.069221000	-3.999521000	-3.259724000
H	5.968958000	-2.068160000	-3.154307000
H	1.914853000	-3.778600000	-0.764534000
H	5.804813000	-1.858558000	-0.693932000
C	3.680751000	-2.712291000	0.934383000
O	2.665483000	-3.139508000	1.499933000
C	4.798761000	-2.072238000	1.746468000
H	4.976610000	-1.024235000	1.426841000
H	4.520317000	-2.084935000	2.816218000
H	5.755890000	-2.617333000	1.609819000
N	4.145230000	-3.204101000	-4.745440000
H	4.854898000	-2.674557000	-5.242742000
H	3.315770000	-3.436862000	-5.283295000

- **3a**

C	4.127970000	-2.780336000	-3.342325000
C	2.874563000	-3.065611000	-2.743558000
C	5.240233000	-2.473458000	-2.514183000
C	2.746997000	-3.030857000	-1.354786000
C	5.092106000	-2.438265000	-1.126499000
C	3.842644000	-2.714687000	-0.513441000
H	2.021770000	-3.318444000	-3.387849000
H	6.218441000	-2.261137000	-2.974205000
H	1.779134000	-3.253516000	-0.879684000
H	5.971700000	-2.197385000	-0.511096000
N	4.279082000	-2.718125000	-4.725214000
H	5.214702000	-2.953649000	-5.066745000
C	3.631053000	-2.692605000	0.963777000
O	2.523105000	-2.945227000	1.452215000
C	4.808963000	-2.349453000	1.863676000
H	5.205174000	-1.338210000	1.634640000
H	4.479362000	-2.382235000	2.918295000
H	5.645505000	-3.064240000	1.717951000
O	3.320064000	-3.439697000	-5.473665000

H	2.837519000	-2.741629000	-5.964053000

- **THF**

O	6.125511000	4.756023000	10.240235000
C	6.232581000	5.919666000	11.065848000
C	5.543341000	3.749716000	11.068774000
C	6.546294000	5.415804000	12.503034000
C	6.265146000	3.889965000	12.421057000
H	4.443648000	3.922911000	11.190175000
H	5.271141000	6.489595000	11.057184000
H	5.899038000	5.919257000	13.248659000
H	7.019966000	6.571071000	10.635359000
H	7.597743000	5.617162000	12.788205000
H	5.659646000	3.512042000	13.268737000
H	7.215185000	3.318114000	12.402176000
H	5.686653000	2.767920000	10.574213000

X-ray Crystallography and Refinement of Structures

Suitable single crystals of compounds **1·CH_3CN** (CCDC 2110200), **6** (CCDC 2117074), and **5·THF** (CCDC 2117073) were selected under a microscope in polarized light with an applied nitrogen cryo-stream at about -40 °C and covered with polyfluorinated polyether. The crystals were picked with nylon loops of suitable sizes and rapidly mounted in the nitrogen cold gas stream of the diffractometer at 100 K. A Bruker D8 Venture diffractometer with IμS3 Diamond source, INCOATEC Helios mirror optics (Mo-Kα radiation; λ=0.71073 Å), and Photon III detector was used for data collection. Final cell constants were obtained from least-squares fits of setting angles of several thousand strong reflections. Intensity data were corrected for absorption using intensities of redundant reflections using SADABS[40]. The structures were readily solved by Direct and Patterson methods and subsequent difference Fourier techniques. The Bruker APEX3[41] software package was used for the solution and refinement of the structures. All non-hydrogen atoms were anisotropically refined, and hydrogen atoms were placed at calculated positions and refined as riding atoms with isotropic displacement parameters.

1·CH_3CN crystallized in non-centrosymmetric space group Pn (No. 7). The crystal was found to be an inversion twin and data were refined accordingly. The asymmetric unit consists of two crystallographically independent complex molecules, four SbF_6-anions, and four molecules of dichloromethane. One SbF_6-anion and one dichloromethane molecule showed severe disorder, and split models were refined. The anion containing Sb50 was split into three positions giving an occupation ratio of about 0.44/0.48/0.08. The solvent molecule was split into two positions (ratio ~0.80/0.20). EADP, ISOR, and SAME restraints of ShelXL were used to model the disorder (192 restraints).

Compound **6** crystallized in space group P-1 (No. 2) with two independent complex molecules, four SbF_6-anions, and a well-defined dichloromethane molecule per asymmetric unit. More solvent was discovered, but the density was found to be diffuse and molecules severely disordered. The scattering contributions of these disordered dichloromethane molecules were removed using Platon/SQUEEZE.[42] A total density of 182 electrons in a solvent-accessible volume of 616 Å3 centered at ½, 1, 0.278 was recovered (~ 4 moles of CH_2Cl_2). Two SbF_6-anions (Sb2 and Sb4) and one phenyl ring of a ligand (C18-C23) were found to be disordered, and split models were refined using the above-mentioned restraints (413 restraints).

The refinement of **5·THF** revealed that the two diethyl ether molecules in the asymmetric unit were slightly disordered and that the molecule containing O81-C85 was not fully occupied. The occupation factor of this second solvent molecule was refined to a value of ~0.20. Displacement parameters of both ether molecules were restrained using ISOR, SIMU, and SAME instructions of ShelXL (121 restraints).

Crystallographic details of data collection and refinement of the structures are shown in **Table S15** to **Table S29**.

1. Molecular Structure of $1{\cdot}CH_3CN$ (CCDC 2110200)

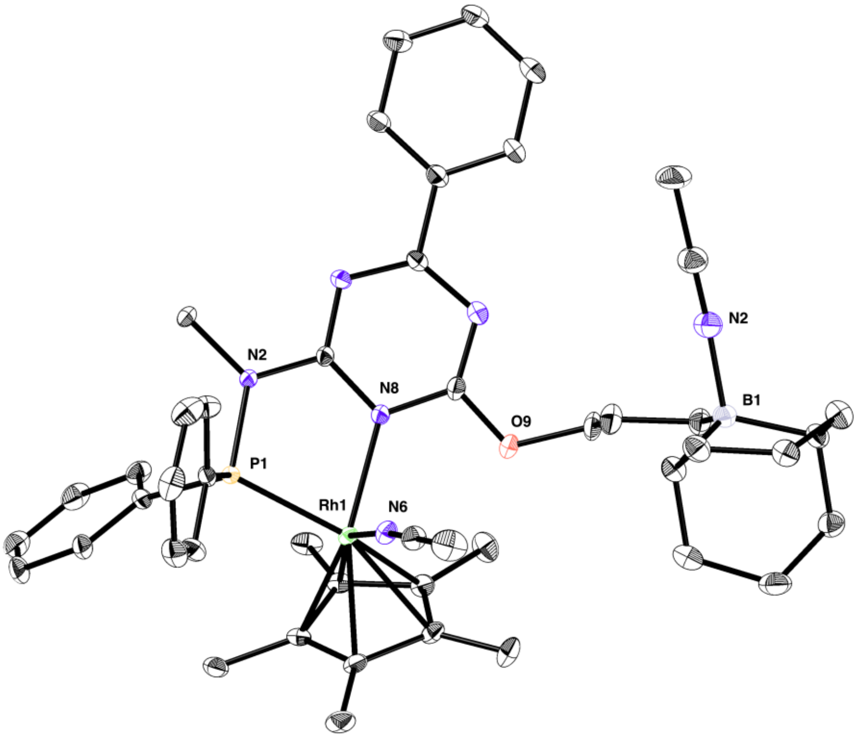

Molecular structure of complex **$1{\cdot}CH_3CN$**. Suitable single crystals were obtained by slow diffusion of a concentrated DCM solution containing **1** and **$1{\cdot}CH_3CN$** into *n*-pentane. The molecular structure is shown with thermal ellipsoids drawn at the 40% probability level. For clarity reasons, the hydrogen atoms and SbF_6 anions were omitted.

Table S15 – Crystal data and structure refinement for **1·CH$_3$CN** (CCDC 2110200).

Identification code	**1·CH$_3$CN**, CCDC 2110200.	
Empirical formula	$C_{49} H_{63} B Cl_4 F_{12} N_6 O P Rh Sb_2$	
Formula weight	1510.04	
Temperature	100(2) K	
Wavelength	0.71073 Å	
Crystal system	Monoclinic	
Space group	Pn ; No. 7	
Unit cell dimensions	a = 14.7354(6) Å	a= 90°.
	b = 17.6276(7) Å	b= 100.720(2)°.
	c = 23.1782(9) Å	g = 90°.
Volume	5915.5(4) Å^3	
Z	4	
Density (calculated)	1.696 Mg/m^3	
Absorption coefficient	1.469 mm^{-1}	
F(000)	3000	
Crystal size	0.263 x 0.243 x 0.095 mm^3	
Theta range for data collection	2.129 to 35.000°.	
Index ranges	-23<=h<=23, -28<=k<=28, -37<=l<=37	
Reflections collected	346240	
Independent reflections	51933 [R(int) = 0.0616]	
Completeness to theta = 25.242°	99.9 %	
Absorption correction	Semi-empirical from equivalents	
Max. and min. transmission	0.7479 and 0.6614	
Refinement method	Full-matrix least-squares on F^2	
Data / restraints / parameters	51933 / 192 / 1459	
Goodness-of-fit on F^2	1.040	
Final R indices [I>2sigma(I)]	R1 = 0.0332, wR2 = 0.0864	
R indices (all data)	R1 = 0.0364, wR2 = 0.0876	
Absolute structure parameter	0.488(9)	
Extinction coefficient	n/a	
Largest diff. peak and hole	2.901 and -1.142 e.Å^{-3}	

Table S16 – Atomic coordinates (x 10^4) and equivalent isotropic displacement parameters ($Å^2$x 10^3) for CCDC 2110200. U(eq) is defined as one-third of the trace of the orthogonalized U^{ij} tensor.

	x	y	z	U(eq)
Rh(1)	6900(1)	3374(1)	5553(1)	12(1)
P(1)	6368(1)	2619(1)	4759(1)	12(1)
N(2)	6701(2)	1739(1)	5012(1)	14(1)
C(3)	7352(2)	1688(2)	5509(1)	13(1)
N(4)	7731(2)	1018(1)	5658(1)	16(1)
C(5)	8402(2)	1012(2)	6126(1)	15(1)
N(6)	8728(2)	1629(2)	6443(1)	19(1)
C(7)	8299(2)	2270(2)	6267(1)	16(1)
N(8)	7574(2)	2337(1)	5819(1)	14(1)
O(9)	8542(2)	2920(1)	6538(1)	21(1)
C(10)	9389(2)	2950(2)	6980(2)	23(1)
C(11)	10203(3)	3111(2)	6675(2)	26(1)
C(12)	11049(3)	3370(2)	7110(2)	22(1)
B(13)	11950(3)	3569(2)	6820(2)	21(1)
C(14)	12779(2)	3999(2)	7259(2)	20(1)
C(15)	12425(3)	4785(2)	7399(2)	26(1)
C(16)	12018(3)	5282(2)	6864(2)	33(1)
C(17)	11410(3)	4851(2)	6361(2)	33(1)
C(18)	11767(3)	4062(2)	6217(2)	27(1)
C(19)	12665(3)	4111(2)	5948(2)	28(1)
C(20)	13513(3)	4389(2)	6380(2)	25(1)
C(21)	13655(3)	4035(2)	6992(2)	25(1)
N(22)	12314(3)	2753(2)	6651(2)	26(1)
C(23)	12517(3)	2158(2)	6531(2)	29(1)
C(24)	12779(4)	1398(3)	6380(2)	42(1)
C(25)	6911(2)	2792(2)	4136(1)	14(1)
C(26)	7485(2)	2258(2)	3950(2)	21(1)
C(27)	7891(3)	2427(2)	3463(2)	29(1)
C(28)	7737(3)	3112(3)	3180(2)	28(1)
C(29)	7182(3)	3653(2)	3380(2)	25(1)
C(30)	6773(2)	3500(2)	3863(2)	20(1)
C(31)	5141(2)	2519(2)	4497(1)	15(1)

C(32)	4642(2)	2109(2)	4857(2)	21(1)
C(33)	3686(3)	2083(2)	4707(2)	28(1)
C(34)	3229(3)	2444(2)	4212(2)	31(1)
C(35)	3714(3)	2843(2)	3853(2)	29(1)
C(36)	4681(2)	2880(2)	3995(2)	21(1)
C(37)	6399(2)	1031(2)	4693(2)	19(1)
C(38)	8841(2)	278(2)	6306(2)	18(1)
C(39)	8408(2)	-392(2)	6085(2)	20(1)
C(40)	8828(3)	-1087(2)	6251(2)	24(1)
C(41)	9679(3)	-1106(2)	6631(2)	27(1)
C(42)	10111(3)	-438(2)	6841(2)	34(1)
C(43)	9692(3)	256(2)	6685(2)	27(1)
C(51)	6624(2)	3788(2)	6426(2)	18(1)
C(52)	7023(2)	4379(2)	6148(1)	17(1)
C(53)	6416(2)	4527(2)	5590(1)	18(1)
C(54)	5634(2)	4039(2)	5543(1)	15(1)
C(55)	5800(2)	3534(2)	6040(2)	16(1)
C(56)	6944(3)	3482(2)	7031(2)	26(1)
C(57)	7877(3)	4813(2)	6385(2)	26(1)
C(58)	6537(3)	5156(2)	5179(2)	25(1)
C(59)	4771(2)	4106(2)	5089(2)	20(1)
C(60)	5155(3)	2954(2)	6208(2)	23(1)
N(61)	8073(2)	3675(2)	5227(1)	17(1)
C(62)	8723(2)	3916(2)	5094(2)	21(1)
C(63)	9554(3)	4226(3)	4930(3)	35(1)
Rh(2)	2707(1)	8330(1)	4387(1)	12(1)
P(101)	3354(1)	7690(1)	5224(1)	12(1)
N(102)	3028(2)	6782(1)	5059(1)	14(1)
C(103)	2391(2)	6643(2)	4562(1)	12(1)
N(104)	2080(2)	5936(1)	4468(1)	14(1)
C(105)	1403(2)	5848(2)	4002(1)	14(1)
N(106)	1024(2)	6410(1)	3646(1)	16(1)
C(107)	1390(2)	7088(2)	3767(1)	14(1)
N(108)	2106(2)	7242(1)	4209(1)	12(1)
O(109)	1070(2)	7686(1)	3452(1)	17(1)
C(110)	301(2)	7578(2)	2956(2)	19(1)
C(111)	-626(2)	7604(2)	3147(2)	20(1)

C(112)	-794(2)	8320(2)	3479(2)	21(1)
B(113)	-1852(3)	8479(2)	3560(2)	19(1)
C(114)	-2536(2)	8837(2)	2994(2)	22(1)
C(115)	-2165(3)	9624(2)	2873(2)	33(1)
C(116)	-1959(4)	10167(2)	3411(3)	41(1)
C(117)	-1542(3)	9788(2)	3998(2)	34(1)
C(118)	-1959(3)	9011(2)	4111(2)	23(1)
C(119)	-2961(3)	9048(2)	4200(2)	27(1)
C(120)	-3657(3)	9286(2)	3662(2)	27(1)
C(121)	-3546(3)	8875(2)	3097(2)	25(1)
N(122)	-2261(2)	7649(2)	3672(1)	21(1)
C(123)	-2496(3)	7046(2)	3735(2)	22(1)
C(124)	-2798(3)	6271(2)	3808(2)	29(1)
C(125)	4589(2)	7618(2)	5446(2)	16(1)
C(126)	5064(2)	7229(2)	5070(2)	21(1)
C(127)	6024(3)	7207(2)	5185(2)	26(1)
C(128)	6520(2)	7576(2)	5681(2)	27(1)
C(129)	6044(2)	7957(2)	6061(2)	26(1)
C(130)	5085(2)	7979(2)	5945(2)	20(1)
C(131)	2893(2)	7947(2)	5865(1)	16(1)
C(132)	3062(3)	8685(2)	6091(2)	23(1)
C(133)	2719(3)	8910(3)	6584(2)	31(1)
C(134)	2190(3)	8408(3)	6843(2)	36(1)
C(135)	1981(3)	7689(3)	6607(2)	33(1)
C(136)	2332(3)	7459(2)	6116(2)	24(1)
C(137)	3368(2)	6138(2)	5444(2)	20(1)
C(138)	1039(2)	5075(2)	3873(2)	17(1)
C(139)	238(3)	4971(2)	3454(2)	27(1)
C(140)	-111(3)	4241(2)	3335(2)	36(1)
C(141)	325(3)	3622(2)	3634(2)	31(1)
C(142)	1119(3)	3728(2)	4049(2)	26(1)
C(143)	1481(2)	4455(2)	4174(2)	21(1)
C(151)	2424(3)	9259(2)	3722(2)	21(1)
C(152)	3033(3)	9496(2)	4261(2)	21(1)
C(153)	3871(2)	9075(2)	4325(2)	19(1)
C(154)	3755(2)	8518(2)	3854(2)	17(1)
C(155)	2881(2)	8675(2)	3474(2)	19(1)

C(156)	1511(3)	9614(2)	3480(2)	31(1)
C(157)	2863(3)	10153(2)	4634(2)	31(1)
C(158)	4738(3)	9226(2)	4759(2)	24(1)
C(159)	4456(3)	7985(2)	3694(2)	24(1)
C(160)	2582(3)	8291(3)	2892(2)	27(1)
N(161)	1578(2)	8605(2)	4757(1)	16(1)
C(162)	1000(2)	8814(2)	4986(2)	19(1)
C(163)	293(3)	9062(3)	5304(2)	34(1)
Sb(20)	-513(1)	6627(1)	5194(1)	22(1)
F(201)	728(2)	6342(3)	5304(2)	56(1)
F(202)	-765(2)	6041(2)	4509(1)	42(1)
F(203)	-767(3)	5796(2)	5638(2)	54(1)
F(204)	-268(3)	7211(2)	5882(2)	48(1)
F(205)	-267(4)	7458(2)	4755(2)	67(1)
F(206)	-1761(3)	6900(3)	5088(2)	64(1)
Sb(30)	4234(1)	5497(1)	3529(1)	19(1)
F(301)	5016(2)	6304(2)	3406(2)	38(1)
F(302)	3232(2)	6171(2)	3373(1)	36(1)
F(303)	4081(3)	5256(2)	2731(1)	46(1)
F(304)	5268(2)	4858(2)	3701(1)	43(1)
F(305)	4402(2)	5760(2)	4324(1)	36(1)
F(306)	3473(3)	4697(2)	3659(2)	53(1)
Sb(40)	10410(1)	1589(1)	4947(1)	25(1)
F(401)	11657(3)	1791(3)	4987(2)	62(1)
F(402)	10223(4)	2573(2)	5178(2)	76(1)
F(403)	10173(3)	1889(3)	4166(2)	65(1)
F(404)	10609(3)	597(2)	4713(3)	69(1)
F(405)	10666(3)	1284(3)	5735(2)	70(1)
F(406)	9168(2)	1379(3)	4915(2)	59(1)
Sb(50)	5538(4)	401(3)	6458(3)	17(1)
F(501)	6021(11)	-208(8)	5917(6)	32(1)
F(502)	4376(5)	341(6)	5975(4)	56(2)
F(503)	5816(7)	1259(4)	6057(4)	52(2)
F(504)	6688(7)	445(7)	6945(4)	68(3)
F(505)	5190(10)	-474(6)	6818(5)	74(4)
F(506)	5033(11)	1006(9)	6981(6)	43(1)
Sb(51)	5576(4)	432(3)	6495(2)	17(1)

F(511)	6086(10)	-100(8)	5928(6)	32(1)
F(512)	4684(5)	828(5)	5897(3)	56(2)
F(513)	6356(6)	1262(3)	6454(4)	52(2)
F(514)	6484(7)	47(7)	7093(4)	68(3)
F(515)	4776(8)	-376(6)	6550(6)	74(4)
F(516)	5047(10)	968(9)	7047(6)	43(1)
Sb(52)	5278(4)	531(2)	6515(2)	17(1)
F(521)	5973(18)	-181(14)	6165(13)	32(1)
F(522)	4410(20)	560(20)	5822(10)	56(2)
F(523)	5960(30)	1317(13)	6246(18)	52(2)
F(524)	6170(20)	470(20)	7198(10)	68(3)
F(525)	4600(30)	-262(14)	6755(18)	74(4)
F(526)	4615(19)	1249(14)	6852(14)	43(1)
C(700)	5487(5)	-1528(4)	7846(3)	53(2)
Cl(71)	6582(1)	-1899(1)	7847(1)	69(1)
Cl(72)	4600(1)	-2111(1)	7468(1)	47(1)
C(800)	4116(3)	3584(2)	2059(2)	30(1)
Cl(81)	5183(1)	3300(1)	2473(1)	47(1)
Cl(82)	3309(1)	2840(1)	2010(1)	51(1)
C(900)	1113(4)	3754(3)	2129(3)	42(1)
Cl(91)	1952(1)	4263(1)	2608(1)	45(1)
Cl(92)	433(1)	4337(1)	1615(1)	76(1)
C(600)	9295(4)	-1261(3)	8002(2)	31(1)
Cl(61)	10495(1)	-1445(1)	8130(1)	33(1)
Cl(62)	9043(2)	-445(2)	8356(2)	78(1)
C(650)	8625(12)	-1168(9)	7991(8)	31(1)
Cl(66)	9503(4)	-643(3)	8434(2)	33(1)
Cl(67)	7856(6)	-560(6)	7550(5)	78(1)

Table S17 – Bond lengths [Å] and angles [°] for CCDC 2110200.

Rh(1)-N(61)	2.081(3)
Rh(1)-N(8)	2.116(3)
Rh(1)-C(55)	2.158(3)
Rh(1)-C(53)	2.162(3)
Rh(1)-C(54)	2.200(3)
Rh(1)-C(52)	2.232(3)
Rh(1)-C(51)	2.258(3)
Rh(1)-P(1)	2.2866(8)
P(1)-N(2)	1.699(3)
P(1)-C(25)	1.802(3)
P(1)-C(31)	1.806(3)
N(2)-C(3)	1.358(4)
N(2)-C(37)	1.477(4)
C(3)-N(4)	1.325(4)
C(3)-N(8)	1.358(4)
N(4)-C(5)	1.326(4)
C(5)-N(6)	1.349(4)
C(5)-C(38)	1.472(4)
N(6)-C(7)	1.322(4)
C(7)-O(9)	1.324(4)
C(7)-N(8)	1.350(4)
O(9)-C(10)	1.462(4)
C(10)-C(11)	1.528(6)
C(11)-C(12)	1.520(5)
C(12)-B(13)	1.633(6)
B(13)-N(22)	1.609(5)
B(13)-C(14)	1.625(5)
B(13)-C(18)	1.626(6)
C(14)-C(21)	1.532(5)
C(14)-C(15)	1.536(5)
C(15)-C(16)	1.546(6)
C(16)-C(17)	1.532(7)
C(17)-C(18)	1.545(6)
C(18)-C(19)	1.568(6)
C(19)-C(20)	1.528(6)

C(20)-C(21)	1.530(6)
N(22)-C(23)	1.139(5)
C(23)-C(24)	1.456(6)
C(25)-C(26)	1.388(4)
C(25)-C(30)	1.396(4)
C(26)-C(27)	1.404(5)
C(27)-C(28)	1.372(6)
C(28)-C(29)	1.390(6)
C(29)-C(30)	1.394(5)
C(31)-C(36)	1.386(5)
C(31)-C(32)	1.411(5)
C(32)-C(33)	1.387(5)
C(33)-C(34)	1.373(7)
C(34)-C(35)	1.385(7)
C(35)-C(36)	1.403(5)
C(38)-C(43)	1.391(5)
C(38)-C(39)	1.393(5)
C(39)-C(40)	1.393(5)
C(40)-C(41)	1.392(6)
C(41)-C(42)	1.383(6)
C(42)-C(43)	1.388(5)
C(51)-C(52)	1.409(4)
C(51)-C(55)	1.439(5)
C(51)-C(56)	1.495(5)
C(52)-C(53)	1.454(5)
C(52)-C(57)	1.488(5)
C(53)-C(54)	1.427(5)
C(53)-C(58)	1.493(5)
C(54)-C(55)	1.440(4)
C(54)-C(59)	1.496(5)
C(55)-C(60)	1.496(5)
N(61)-C(62)	1.141(4)
C(62)-C(63)	1.455(5)
Rh(2)-N(161)	2.066(3)
Rh(2)-N(108)	2.120(2)
Rh(2)-C(152)	2.142(3)
Rh(2)-C(154)	2.174(3)

Rh(2)-C(153)	2.186(3)
Rh(2)-C(151)	2.233(3)
Rh(2)-C(155)	2.261(3)
Rh(2)-P(101)	2.2937(8)
P(101)-N(102)	1.694(3)
P(101)-C(125)	1.802(3)
P(101)-C(131)	1.803(3)
N(102)-C(103)	1.365(4)
N(102)-C(137)	1.473(4)
C(103)-N(104)	1.332(3)
C(103)-N(108)	1.354(4)
N(104)-C(105)	1.337(4)
C(105)-N(106)	1.341(4)
C(105)-C(138)	1.474(4)
N(106)-C(107)	1.320(4)
C(107)-O(109)	1.319(4)
C(107)-N(108)	1.354(4)
O(109)-C(110)	1.470(4)
C(110)-C(111)	1.513(5)
C(111)-C(112)	1.522(5)
C(112)-B(113)	1.630(5)
B(113)-C(118)	1.616(5)
B(113)-N(122)	1.622(5)
B(113)-C(114)	1.624(5)
C(114)-C(115)	1.536(5)
C(114)-C(121)	1.551(5)
C(115)-C(116)	1.557(8)
C(116)-C(117)	1.536(8)
C(117)-C(118)	1.543(6)
C(118)-C(119)	1.531(6)
C(119)-C(120)	1.518(6)
C(120)-C(121)	1.533(6)
N(122)-C(123)	1.135(4)
C(123)-C(124)	1.457(5)
C(125)-C(126)	1.395(5)
C(125)-C(130)	1.400(5)
C(126)-C(127)	1.391(5)

C(127)-C(128)	1.403(6)
C(128)-C(129)	1.395(6)
C(129)-C(130)	1.389(5)
C(131)-C(136)	1.393(5)
C(131)-C(132)	1.408(5)
C(132)-C(133)	1.390(5)
C(133)-C(134)	1.388(7)
C(134)-C(135)	1.393(7)
C(135)-C(136)	1.394(5)
C(138)-C(143)	1.390(5)
C(138)-C(139)	1.394(5)
C(139)-C(140)	1.395(5)
C(140)-C(141)	1.385(7)
C(141)-C(142)	1.382(6)
C(142)-C(143)	1.398(5)
C(151)-C(155)	1.410(5)
C(151)-C(152)	1.457(5)
C(151)-C(156)	1.495(6)
C(152)-C(153)	1.424(5)
C(152)-C(157)	1.495(5)
C(153)-C(154)	1.454(5)
C(153)-C(158)	1.495(5)
C(154)-C(155)	1.444(5)
C(154)-C(159)	1.494(5)
C(155)-C(160)	1.501(5)
N(161)-C(162)	1.146(4)
C(162)-C(163)	1.451(5)
Sb(20)-F(205)	1.856(3)
Sb(20)-F(201)	1.868(3)
Sb(20)-F(203)	1.868(3)
Sb(20)-F(206)	1.871(3)
Sb(20)-F(202)	1.873(3)
Sb(20)-F(204)	1.874(3)
Sb(30)-F(306)	1.861(3)
Sb(30)-F(303)	1.870(3)
Sb(30)-F(305)	1.873(3)
Sb(30)-F(302)	1.876(3)

Sb(30)-F(304)	1.878(3)
Sb(30)-F(301)	1.885(3)
Sb(40)-F(402)	1.851(4)
Sb(40)-F(403)	1.854(4)
Sb(40)-F(406)	1.856(3)
Sb(40)-F(401)	1.857(4)
Sb(40)-F(404)	1.871(3)
Sb(40)-F(405)	1.873(4)
Sb(50)-F(504)	1.854(8)
Sb(50)-F(503)	1.859(7)
Sb(50)-F(502)	1.866(7)
Sb(50)-F(506)	1.870(7)
Sb(50)-F(505)	1.870(8)
Sb(50)-F(501)	1.886(7)
Sb(51)-F(512)	1.859(7)
Sb(51)-F(515)	1.867(7)
Sb(51)-F(514)	1.868(7)
Sb(51)-F(516)	1.874(7)
Sb(51)-F(513)	1.875(7)
Sb(51)-F(511)	1.880(6)
Sb(52)-F(526)	1.858(10)
Sb(52)-F(522)	1.860(10)
Sb(52)-F(525)	1.861(10)
Sb(52)-F(524)	1.867(10)
Sb(52)-F(523)	1.880(10)
Sb(52)-F(521)	1.894(10)
C(700)-Cl(71)	1.740(7)
C(700)-Cl(72)	1.762(7)
C(800)-Cl(81)	1.756(4)
C(800)-Cl(82)	1.759(5)
C(900)-Cl(92)	1.743(6)
C(900)-Cl(91)	1.748(5)
C(600)-Cl(62)	1.731(5)
C(600)-Cl(61)	1.769(6)
C(650)-Cl(67)	1.746(12)
C(650)-Cl(66)	1.757(11)

N(61)-Rh(1)-N(8)	87.13(11)
N(61)-Rh(1)-C(55)	155.73(11)
N(8)-Rh(1)-C(55)	108.52(11)
N(61)-Rh(1)-C(53)	94.37(12)
N(8)-Rh(1)-C(53)	160.24(12)
C(55)-Rh(1)-C(53)	64.61(12)
N(61)-Rh(1)-C(54)	128.35(11)
N(8)-Rh(1)-C(54)	144.52(11)
C(55)-Rh(1)-C(54)	38.58(12)
C(53)-Rh(1)-C(54)	38.17(12)
N(61)-Rh(1)-C(52)	92.50(11)
N(8)-Rh(1)-C(52)	121.70(11)
C(55)-Rh(1)-C(52)	63.62(12)
C(53)-Rh(1)-C(52)	38.60(13)
C(54)-Rh(1)-C(52)	63.57(12)
N(61)-Rh(1)-C(51)	122.86(12)
N(8)-Rh(1)-C(51)	99.85(11)
C(55)-Rh(1)-C(51)	37.94(13)
C(53)-Rh(1)-C(51)	62.91(12)
C(54)-Rh(1)-C(51)	62.83(12)
C(52)-Rh(1)-C(51)	36.57(11)
N(61)-Rh(1)-P(1)	92.33(8)
N(8)-Rh(1)-P(1)	78.73(7)
C(55)-Rh(1)-P(1)	108.55(9)
C(53)-Rh(1)-P(1)	120.82(9)
C(54)-Rh(1)-P(1)	97.93(9)
C(52)-Rh(1)-P(1)	159.22(9)
C(51)-Rh(1)-P(1)	144.78(9)
N(2)-P(1)-C(25)	106.74(14)
N(2)-P(1)-C(31)	103.53(14)
C(25)-P(1)-C(31)	108.20(14)
N(2)-P(1)-Rh(1)	102.70(9)
C(25)-P(1)-Rh(1)	114.14(10)
C(31)-P(1)-Rh(1)	120.01(11)
C(3)-N(2)-C(37)	118.0(2)
C(3)-N(2)-P(1)	117.8(2)
C(37)-N(2)-P(1)	123.9(2)

N(4)-C(3)-N(2)	118.5(3)
N(4)-C(3)-N(8)	124.4(3)
N(2)-C(3)-N(8)	117.1(3)
C(3)-N(4)-C(5)	115.7(3)
N(4)-C(5)-N(6)	125.1(3)
N(4)-C(5)-C(38)	117.3(3)
N(6)-C(5)-C(38)	117.5(3)
C(7)-N(6)-C(5)	114.8(3)
N(6)-C(7)-O(9)	121.5(3)
N(6)-C(7)-N(8)	125.3(3)
O(9)-C(7)-N(8)	113.2(3)
C(7)-N(8)-C(3)	114.3(3)
C(7)-N(8)-Rh(1)	124.2(2)
C(3)-N(8)-Rh(1)	121.3(2)
C(7)-O(9)-C(10)	118.9(3)
O(9)-C(10)-C(11)	108.9(3)
C(12)-C(11)-C(10)	111.5(3)
C(11)-C(12)-B(13)	114.9(3)
N(22)-B(13)-C(14)	108.9(3)
N(22)-B(13)-C(18)	106.2(3)
C(14)-B(13)-C(18)	105.9(3)
N(22)-B(13)-C(12)	104.0(3)
C(14)-B(13)-C(12)	114.7(3)
C(18)-B(13)-C(12)	116.8(3)
C(21)-C(14)-C(15)	113.2(3)
C(21)-C(14)-B(13)	111.0(3)
C(15)-C(14)-B(13)	107.9(3)
C(14)-C(15)-C(16)	115.9(3)
C(17)-C(16)-C(15)	114.5(3)
C(16)-C(17)-C(18)	115.9(3)
C(17)-C(18)-C(19)	112.5(4)
C(17)-C(18)-B(13)	107.7(3)
C(19)-C(18)-B(13)	110.8(3)
C(20)-C(19)-C(18)	113.8(3)
C(19)-C(20)-C(21)	115.6(3)
C(20)-C(21)-C(14)	114.3(3)
C(23)-N(22)-B(13)	175.7(4)

N(22)-C(23)-C(24)	179.8(7)
C(26)-C(25)-C(30)	120.7(3)
C(26)-C(25)-P(1)	121.5(2)
C(30)-C(25)-P(1)	117.7(2)
C(25)-C(26)-C(27)	118.9(3)
C(28)-C(27)-C(26)	120.8(3)
C(27)-C(28)-C(29)	120.0(3)
C(28)-C(29)-C(30)	120.3(3)
C(29)-C(30)-C(25)	119.2(3)
C(36)-C(31)-C(32)	120.3(3)
C(36)-C(31)-P(1)	122.7(3)
C(32)-C(31)-P(1)	116.7(2)
C(33)-C(32)-C(31)	118.9(4)
C(34)-C(33)-C(32)	120.9(4)
C(33)-C(34)-C(35)	120.5(3)
C(34)-C(35)-C(36)	119.9(4)
C(31)-C(36)-C(35)	119.5(4)
C(43)-C(38)-C(39)	120.4(3)
C(43)-C(38)-C(5)	119.9(3)
C(39)-C(38)-C(5)	119.7(3)
C(38)-C(39)-C(40)	119.6(3)
C(41)-C(40)-C(39)	119.9(4)
C(42)-C(41)-C(40)	120.1(3)
C(41)-C(42)-C(43)	120.4(4)
C(42)-C(43)-C(38)	119.6(4)
C(52)-C(51)-C(55)	108.8(3)
C(52)-C(51)-C(56)	127.6(3)
C(55)-C(51)-C(56)	123.6(3)
C(52)-C(51)-Rh(1)	70.71(18)
C(55)-C(51)-Rh(1)	67.26(17)
C(56)-C(51)-Rh(1)	130.2(3)
C(51)-C(52)-C(53)	107.4(3)
C(51)-C(52)-C(57)	127.5(3)
C(53)-C(52)-C(57)	125.0(3)
C(51)-C(52)-Rh(1)	72.72(18)
C(53)-C(52)-Rh(1)	68.08(16)
C(57)-C(52)-Rh(1)	127.0(2)

C(54)-C(53)-C(52)	108.3(3)
C(54)-C(53)-C(58)	126.2(3)
C(52)-C(53)-C(58)	125.0(3)
C(54)-C(53)-Rh(1)	72.36(16)
C(52)-C(53)-Rh(1)	73.32(17)
C(58)-C(53)-Rh(1)	126.5(2)
C(53)-C(54)-C(55)	107.3(3)
C(53)-C(54)-C(59)	125.0(3)
C(55)-C(54)-C(59)	127.4(3)
C(53)-C(54)-Rh(1)	69.47(17)
C(55)-C(54)-Rh(1)	69.15(17)
C(59)-C(54)-Rh(1)	131.1(2)
C(51)-C(55)-C(54)	107.7(3)
C(51)-C(55)-C(60)	123.7(3)
C(54)-C(55)-C(60)	127.5(3)
C(51)-C(55)-Rh(1)	74.79(18)
C(54)-C(55)-Rh(1)	72.27(17)
C(60)-C(55)-Rh(1)	128.4(2)
C(62)-N(61)-Rh(1)	171.5(3)
N(61)-C(62)-C(63)	179.4(5)
N(161)-Rh(2)-N(108)	87.35(10)
N(161)-Rh(2)-C(152)	92.89(12)
N(108)-Rh(2)-C(152)	159.61(13)
N(161)-Rh(2)-C(154)	155.74(12)
N(108)-Rh(2)-C(154)	110.02(11)
C(152)-Rh(2)-C(154)	64.86(13)
N(161)-Rh(2)-C(153)	126.07(12)
N(108)-Rh(2)-C(153)	146.58(11)
C(152)-Rh(2)-C(153)	38.39(14)
C(154)-Rh(2)-C(153)	38.96(12)
N(161)-Rh(2)-C(151)	93.05(12)
N(108)-Rh(2)-C(151)	120.79(12)
C(152)-Rh(2)-C(151)	38.82(14)
C(154)-Rh(2)-C(151)	63.75(13)
C(153)-Rh(2)-C(151)	64.07(13)
N(161)-Rh(2)-C(155)	124.47(12)
N(108)-Rh(2)-C(155)	100.23(11)

C(152)-Rh(2)-C(155)	63.08(13)
C(154)-Rh(2)-C(155)	37.95(13)
C(153)-Rh(2)-C(155)	63.27(13)
C(151)-Rh(2)-C(155)	36.57(13)
N(161)-Rh(2)-P(101)	90.25(8)
N(108)-Rh(2)-P(101)	79.14(7)
C(152)-Rh(2)-P(101)	121.24(11)
C(154)-Rh(2)-P(101)	109.14(10)
C(153)-Rh(2)-P(101)	98.22(10)
C(151)-Rh(2)-P(101)	159.91(10)
C(155)-Rh(2)-P(101)	145.28(10)
N(102)-P(101)-C(125)	103.11(14)
N(102)-P(101)-C(131)	106.79(15)
C(125)-P(101)-C(131)	107.30(15)
N(102)-P(101)-Rh(2)	102.63(10)
C(125)-P(101)-Rh(2)	121.04(12)
C(131)-P(101)-Rh(2)	114.35(10)
C(103)-N(102)-C(137)	118.5(2)
C(103)-N(102)-P(101)	118.7(2)
C(137)-N(102)-P(101)	122.7(2)
N(104)-C(103)-N(108)	124.9(3)
N(104)-C(103)-N(102)	117.8(3)
N(108)-C(103)-N(102)	117.2(2)
C(103)-N(104)-C(105)	115.0(3)
N(104)-C(105)-N(106)	125.0(3)
N(104)-C(105)-C(138)	117.3(3)
N(106)-C(105)-C(138)	117.7(3)
C(107)-N(106)-C(105)	115.6(3)
O(109)-C(107)-N(106)	121.1(3)
O(109)-C(107)-N(108)	113.9(3)
N(106)-C(107)-N(108)	124.9(3)
C(107)-N(108)-C(103)	114.2(2)
C(107)-N(108)-Rh(2)	124.5(2)
C(103)-N(108)-Rh(2)	121.11(19)
C(107)-O(109)-C(110)	118.3(2)
O(109)-C(110)-C(111)	111.9(3)
C(110)-C(111)-C(112)	114.5(3)

C(111)-C(112)-B(113)	116.8(3)
C(118)-B(113)-N(122)	107.7(3)
C(118)-B(113)-C(114)	106.0(3)
N(122)-B(113)-C(114)	106.7(3)
C(118)-B(113)-C(112)	115.2(3)
N(122)-B(113)-C(112)	104.8(3)
C(114)-B(113)-C(112)	115.9(3)
C(115)-C(114)-C(121)	111.9(3)
C(115)-C(114)-B(113)	108.1(3)
C(121)-C(114)-B(113)	111.2(3)
C(114)-C(115)-C(116)	115.6(4)
C(117)-C(116)-C(115)	115.4(3)
C(116)-C(117)-C(118)	115.5(4)
C(119)-C(118)-C(117)	114.2(3)
C(119)-C(118)-B(113)	111.7(3)
C(117)-C(118)-B(113)	106.3(3)
C(120)-C(119)-C(118)	115.0(3)
C(119)-C(120)-C(121)	114.1(3)
C(120)-C(121)-C(114)	114.2(3)
C(123)-N(122)-B(113)	175.0(3)
N(122)-C(123)-C(124)	179.4(4)
C(126)-C(125)-C(130)	119.6(3)
C(126)-C(125)-P(101)	116.9(2)
C(130)-C(125)-P(101)	123.3(3)
C(127)-C(126)-C(125)	120.3(3)
C(126)-C(127)-C(128)	120.0(3)
C(129)-C(128)-C(127)	119.6(3)
C(130)-C(129)-C(128)	120.3(3)
C(129)-C(130)-C(125)	120.2(3)
C(136)-C(131)-C(132)	119.5(3)
C(136)-C(131)-P(101)	122.2(3)
C(132)-C(131)-P(101)	118.1(3)
C(133)-C(132)-C(131)	120.3(4)
C(134)-C(133)-C(132)	119.5(4)
C(133)-C(134)-C(135)	120.7(4)
C(134)-C(135)-C(136)	119.8(4)
C(131)-C(136)-C(135)	120.0(4)

C(143)-C(138)-C(139)	120.2(3)
C(143)-C(138)-C(105)	120.4(3)
C(139)-C(138)-C(105)	119.4(3)
C(138)-C(139)-C(140)	119.5(4)
C(141)-C(140)-C(139)	120.5(4)
C(142)-C(141)-C(140)	119.7(3)
C(141)-C(142)-C(143)	120.6(4)
C(138)-C(143)-C(142)	119.5(3)
C(155)-C(151)-C(152)	107.1(3)
C(155)-C(151)-C(156)	128.0(4)
C(152)-C(151)-C(156)	124.8(3)
C(155)-C(151)-Rh(2)	72.80(19)
C(152)-C(151)-Rh(2)	67.22(18)
C(156)-C(151)-Rh(2)	127.2(3)
C(153)-C(152)-C(151)	109.0(3)
C(153)-C(152)-C(157)	125.4(4)
C(151)-C(152)-C(157)	125.0(4)
C(153)-C(152)-Rh(2)	72.48(17)
C(151)-C(152)-Rh(2)	73.96(18)
C(157)-C(152)-Rh(2)	126.8(3)
C(152)-C(153)-C(154)	107.1(3)
C(152)-C(153)-C(158)	126.4(3)
C(154)-C(153)-C(158)	126.3(3)
C(152)-C(153)-Rh(2)	69.13(18)
C(154)-C(153)-Rh(2)	70.08(17)
C(158)-C(153)-Rh(2)	130.2(2)
C(155)-C(154)-C(153)	107.2(3)
C(155)-C(154)-C(159)	123.2(3)
C(153)-C(154)-C(159)	128.3(3)
C(155)-C(154)-Rh(2)	74.28(18)
C(153)-C(154)-Rh(2)	70.96(18)
C(159)-C(154)-Rh(2)	129.9(2)
C(151)-C(155)-C(154)	109.3(3)
C(151)-C(155)-C(160)	127.8(4)
C(154)-C(155)-C(160)	122.8(3)
C(151)-C(155)-Rh(2)	70.63(19)
C(154)-C(155)-Rh(2)	67.77(18)

C(160)-C(155)-Rh(2)	130.9(2)
C(162)-N(161)-Rh(2)	173.6(3)
N(161)-C(162)-C(163)	177.1(4)
F(205)-Sb(20)-F(201)	90.0(2)
F(205)-Sb(20)-F(203)	179.6(2)
F(201)-Sb(20)-F(203)	90.3(2)
F(205)-Sb(20)-F(206)	90.7(2)
F(201)-Sb(20)-F(206)	179.2(2)
F(203)-Sb(20)-F(206)	89.0(2)
F(205)-Sb(20)-F(202)	89.90(17)
F(201)-Sb(20)-F(202)	90.09(17)
F(203)-Sb(20)-F(202)	90.31(16)
F(206)-Sb(20)-F(202)	89.68(17)
F(205)-Sb(20)-F(204)	90.41(17)
F(201)-Sb(20)-F(204)	90.16(18)
F(203)-Sb(20)-F(204)	89.38(17)
F(206)-Sb(20)-F(204)	90.06(18)
F(202)-Sb(20)-F(204)	179.60(17)
F(306)-Sb(30)-F(303)	91.28(18)
F(306)-Sb(30)-F(305)	90.08(16)
F(303)-Sb(30)-F(305)	178.64(16)
F(306)-Sb(30)-F(302)	92.01(17)
F(303)-Sb(30)-F(302)	90.51(15)
F(305)-Sb(30)-F(302)	89.31(14)
F(306)-Sb(30)-F(304)	90.00(18)
F(303)-Sb(30)-F(304)	90.93(14)
F(305)-Sb(30)-F(304)	89.20(13)
F(302)-Sb(30)-F(304)	177.50(16)
F(306)-Sb(30)-F(301)	179.21(17)
F(303)-Sb(30)-F(301)	89.27(16)
F(305)-Sb(30)-F(301)	89.37(15)
F(302)-Sb(30)-F(301)	88.56(13)
F(304)-Sb(30)-F(301)	89.42(15)
F(402)-Sb(40)-F(403)	90.2(2)
F(402)-Sb(40)-F(406)	90.0(2)
F(403)-Sb(40)-F(406)	90.7(2)
F(402)-Sb(40)-F(401)	90.3(2)

F(403)-Sb(40)-F(401)	90.0(2)
F(406)-Sb(40)-F(401)	179.3(2)
F(402)-Sb(40)-F(404)	179.5(2)
F(403)-Sb(40)-F(404)	89.7(2)
F(406)-Sb(40)-F(404)	90.5(2)
F(401)-Sb(40)-F(404)	89.2(2)
F(402)-Sb(40)-F(405)	90.0(3)
F(403)-Sb(40)-F(405)	179.3(2)
F(406)-Sb(40)-F(405)	90.0(2)
F(401)-Sb(40)-F(405)	89.3(2)
F(404)-Sb(40)-F(405)	90.0(3)
F(504)-Sb(50)-F(503)	90.5(5)
F(504)-Sb(50)-F(502)	178.9(6)
F(503)-Sb(50)-F(502)	90.6(5)
F(504)-Sb(50)-F(506)	90.6(6)
F(503)-Sb(50)-F(506)	90.6(6)
F(502)-Sb(50)-F(506)	89.3(6)
F(504)-Sb(50)-F(505)	93.3(6)
F(503)-Sb(50)-F(505)	176.0(6)
F(502)-Sb(50)-F(505)	85.6(6)
F(506)-Sb(50)-F(505)	90.3(6)
F(504)-Sb(50)-F(501)	90.8(6)
F(503)-Sb(50)-F(501)	89.2(6)
F(502)-Sb(50)-F(501)	89.3(6)
F(506)-Sb(50)-F(501)	178.6(7)
F(505)-Sb(50)-F(501)	89.8(6)
F(512)-Sb(51)-F(515)	88.3(5)
F(512)-Sb(51)-F(514)	179.1(6)
F(515)-Sb(51)-F(514)	92.7(6)
F(512)-Sb(51)-F(516)	89.7(6)
F(515)-Sb(51)-F(516)	89.4(6)
F(514)-Sb(51)-F(516)	90.4(6)
F(512)-Sb(51)-F(513)	91.4(5)
F(515)-Sb(51)-F(513)	178.1(5)
F(514)-Sb(51)-F(513)	87.7(5)
F(516)-Sb(51)-F(513)	88.8(5)
F(512)-Sb(51)-F(511)	89.2(5)

F(515)-Sb(51)-F(511)	90.4(5)
F(514)-Sb(51)-F(511)	90.8(5)
F(516)-Sb(51)-F(511)	178.9(7)
F(513)-Sb(51)-F(511)	91.4(5)
F(526)-Sb(52)-F(522)	90.6(9)
F(526)-Sb(52)-F(525)	91.9(9)
F(522)-Sb(52)-F(525)	87.6(9)
F(526)-Sb(52)-F(524)	91.5(9)
F(522)-Sb(52)-F(524)	177.8(11)
F(525)-Sb(52)-F(524)	92.0(10)
F(526)-Sb(52)-F(523)	89.5(9)
F(522)-Sb(52)-F(523)	91.0(9)
F(525)-Sb(52)-F(523)	178.1(11)
F(524)-Sb(52)-F(523)	89.4(9)
F(526)-Sb(52)-F(521)	178.6(10)
F(522)-Sb(52)-F(521)	89.5(9)
F(525)-Sb(52)-F(521)	89.6(9)
F(524)-Sb(52)-F(521)	88.4(9)
F(523)-Sb(52)-F(521)	89.1(9)
Cl(71)-C(700)-Cl(72)	112.6(4)
Cl(81)-C(800)-Cl(82)	110.4(2)
Cl(92)-C(900)-Cl(91)	112.3(3)
Cl(62)-C(600)-Cl(61)	111.7(3)
Cl(67)-C(650)-Cl(66)	110.2(9)

Table S18 – Anisotropic displacement parameters ($Å^2$x 10^3) for CCDC 2110200. The anisotropic displacement factor exponent takes the form: -2p^2[h^2 a*^{2}U^{11} + ... + 2 h k a* b* U^{12}].

	U^{11}	U^{22}	U^{33}	U^{23}	U^{13}	U^{12}
Rh(1)	14(1)	10(1)	12(1)	0(1)	3(1)	-2(1)
P(1)	12(1)	10(1)	12(1)	0(1)	1(1)	0(1)
N(2)	16(1)	10(1)	14(1)	0(1)	-2(1)	-1(1)
C(3)	12(1)	13(1)	14(1)	0(1)	0(1)	-2(1)
N(4)	14(1)	13(1)	18(1)	1(1)	1(1)	2(1)
C(5)	13(1)	16(1)	17(1)	3(1)	2(1)	2(1)
N(6)	16(1)	20(1)	18(1)	1(1)	0(1)	1(1)
C(7)	16(1)	17(1)	14(1)	-1(1)	1(1)	-4(1)
N(8)	14(1)	12(1)	14(1)	0(1)	0(1)	-1(1)
O(9)	20(1)	19(1)	20(1)	-6(1)	-3(1)	-2(1)
C(10)	16(1)	29(2)	21(1)	-9(1)	-3(1)	-2(1)
C(11)	24(2)	32(2)	21(2)	-6(1)	1(1)	-3(1)
C(12)	21(1)	25(1)	20(1)	-4(1)	1(1)	0(1)
B(13)	23(2)	19(1)	19(2)	-1(1)	3(1)	3(1)
C(14)	18(1)	23(1)	20(1)	0(1)	2(1)	3(1)
C(15)	25(2)	26(2)	28(2)	-5(1)	5(1)	2(1)
C(16)	35(2)	20(1)	44(2)	-1(2)	7(2)	7(1)
C(17)	32(2)	27(2)	37(2)	5(2)	0(2)	9(1)
C(18)	29(2)	25(2)	25(2)	1(1)	-3(1)	4(1)
C(19)	34(2)	26(2)	23(2)	6(1)	2(1)	2(1)
C(20)	26(2)	27(2)	22(2)	2(1)	7(1)	2(1)
C(21)	22(1)	32(2)	23(2)	2(1)	7(1)	3(1)
N(22)	32(2)	25(1)	21(1)	1(1)	7(1)	4(1)
C(23)	34(2)	28(2)	27(2)	0(1)	8(2)	5(1)
C(24)	56(3)	30(2)	41(3)	-8(2)	6(2)	20(2)
C(25)	12(1)	15(1)	13(1)	1(1)	0(1)	0(1)
C(26)	20(1)	19(1)	27(2)	0(1)	12(1)	2(1)
C(27)	29(2)	29(2)	34(2)	-9(2)	20(2)	-4(1)
C(28)	30(2)	37(2)	20(2)	-3(1)	10(1)	-12(2)
C(29)	28(2)	29(2)	19(1)	10(1)	4(1)	-3(1)
C(30)	20(1)	19(1)	20(1)	6(1)	3(1)	2(1)
C(31)	12(1)	15(1)	18(1)	0(1)	1(1)	1(1)

C(32)	17(1)	16(1)	30(2)	-3(1)	7(1)	-4(1)
C(33)	17(1)	24(2)	45(2)	-9(2)	11(1)	-5(1)
C(34)	15(1)	29(2)	49(3)	-16(2)	3(1)	0(1)
C(35)	15(1)	34(2)	34(2)	-8(2)	-8(1)	7(1)
C(36)	15(1)	23(1)	22(2)	-3(1)	-2(1)	1(1)
C(37)	24(1)	12(1)	18(1)	-3(1)	-3(1)	-2(1)
C(38)	16(1)	19(1)	19(1)	4(1)	1(1)	1(1)
C(39)	17(1)	17(1)	25(2)	4(1)	0(1)	2(1)
C(40)	24(2)	18(1)	31(2)	5(1)	6(1)	3(1)
C(41)	30(2)	22(1)	27(2)	5(1)	-1(1)	9(1)
C(42)	29(2)	28(2)	37(2)	4(2)	-11(2)	9(1)
C(43)	25(2)	24(2)	27(2)	1(1)	-9(1)	6(1)
C(51)	22(1)	17(1)	15(1)	1(1)	5(1)	0(1)
C(52)	24(1)	14(1)	14(1)	-3(1)	6(1)	-3(1)
C(53)	26(1)	11(1)	16(1)	-1(1)	5(1)	-1(1)
C(54)	19(1)	12(1)	16(1)	2(1)	6(1)	4(1)
C(55)	18(1)	15(1)	18(1)	2(1)	8(1)	2(1)
C(56)	29(2)	34(2)	16(1)	6(1)	5(1)	2(1)
C(57)	32(2)	26(2)	21(2)	-8(1)	3(1)	-9(1)
C(58)	37(2)	14(1)	25(2)	6(1)	8(1)	-3(1)
C(59)	21(1)	17(1)	22(1)	2(1)	3(1)	6(1)
C(60)	24(1)	18(1)	29(2)	6(1)	14(1)	2(1)
N(61)	19(1)	17(1)	16(1)	-1(1)	4(1)	-4(1)
C(62)	18(1)	20(1)	24(2)	0(1)	5(1)	-1(1)
C(63)	25(2)	33(2)	52(3)	10(2)	18(2)	-5(2)
Rh(2)	14(1)	9(1)	14(1)	-1(1)	3(1)	-1(1)
P(101)	12(1)	12(1)	13(1)	-3(1)	1(1)	-1(1)
N(102)	16(1)	11(1)	15(1)	1(1)	-1(1)	-2(1)
C(103)	14(1)	9(1)	13(1)	-1(1)	2(1)	-2(1)
N(104)	14(1)	11(1)	16(1)	-1(1)	1(1)	-2(1)
C(105)	14(1)	12(1)	16(1)	-2(1)	2(1)	-3(1)
N(106)	16(1)	13(1)	18(1)	-2(1)	0(1)	-1(1)
C(107)	12(1)	14(1)	14(1)	0(1)	1(1)	0(1)
N(108)	12(1)	10(1)	13(1)	-1(1)	1(1)	-1(1)
O(109)	16(1)	16(1)	17(1)	2(1)	-1(1)	1(1)
C(110)	16(1)	22(1)	17(1)	0(1)	-1(1)	5(1)
C(111)	14(1)	21(1)	23(1)	-3(1)	0(1)	0(1)

C(112)	17(1)	19(1)	26(2)	-2(1)	3(1)	-1(1)
B(113)	18(1)	16(1)	23(2)	-1(1)	6(1)	-2(1)
C(114)	20(1)	25(1)	23(2)	6(1)	6(1)	2(1)
C(115)	29(2)	30(2)	42(2)	18(2)	12(2)	2(1)
C(116)	38(2)	16(1)	75(4)	9(2)	24(2)	0(1)
C(117)	26(2)	22(2)	53(3)	-9(2)	7(2)	-6(1)
C(118)	24(1)	21(1)	24(2)	-3(1)	3(1)	-1(1)
C(119)	27(2)	26(2)	30(2)	-3(1)	9(1)	0(1)
C(120)	23(2)	22(1)	37(2)	0(1)	10(1)	4(1)
C(121)	21(1)	26(2)	28(2)	4(1)	2(1)	3(1)
N(122)	20(1)	20(1)	24(1)	2(1)	5(1)	2(1)
C(123)	23(1)	20(1)	25(2)	0(1)	7(1)	-2(1)
C(124)	32(2)	22(1)	34(2)	4(1)	6(2)	-8(1)
C(125)	13(1)	15(1)	19(1)	-3(1)	0(1)	0(1)
C(126)	17(1)	18(1)	27(2)	-6(1)	5(1)	0(1)
C(127)	18(1)	20(1)	41(2)	-3(1)	11(1)	3(1)
C(128)	13(1)	25(2)	41(2)	3(1)	0(1)	1(1)
C(129)	17(1)	29(2)	28(2)	-2(1)	-6(1)	-1(1)
C(130)	15(1)	21(1)	20(1)	-4(1)	-3(1)	-2(1)
C(131)	15(1)	22(1)	12(1)	-2(1)	0(1)	3(1)
C(132)	26(2)	24(1)	18(1)	-6(1)	2(1)	6(1)
C(133)	36(2)	36(2)	19(2)	-10(1)	3(1)	14(2)
C(134)	35(2)	56(3)	19(2)	-3(2)	9(2)	21(2)
C(135)	25(2)	51(2)	25(2)	2(2)	13(1)	7(2)
C(136)	20(1)	33(2)	20(1)	-2(1)	5(1)	0(1)
C(137)	23(1)	15(1)	19(1)	4(1)	-3(1)	-1(1)
C(138)	16(1)	13(1)	21(1)	-4(1)	2(1)	-3(1)
C(139)	25(2)	21(1)	31(2)	-3(1)	-6(1)	-7(1)
C(140)	33(2)	23(2)	45(2)	-7(2)	-12(2)	-10(1)
C(141)	33(2)	16(1)	42(2)	-9(1)	5(2)	-9(1)
C(142)	25(2)	12(1)	42(2)	-6(1)	11(1)	-2(1)
C(143)	19(1)	13(1)	29(2)	-3(1)	4(1)	-1(1)
C(151)	27(2)	14(1)	22(1)	4(1)	5(1)	-2(1)
C(152)	30(2)	10(1)	25(2)	-2(1)	10(1)	-2(1)
C(153)	23(1)	15(1)	21(1)	-2(1)	9(1)	-8(1)
C(154)	19(1)	15(1)	18(1)	-3(1)	7(1)	-6(1)
C(155)	25(1)	16(1)	17(1)	0(1)	7(1)	-6(1)

C(156)	32(2)	21(1)	38(2)	13(1)	5(2)	7(1)
C(157)	42(2)	12(1)	43(2)	-7(1)	15(2)	-2(1)
C(158)	24(2)	24(1)	26(2)	-9(1)	7(1)	-12(1)
C(159)	21(1)	26(2)	28(2)	-10(1)	12(1)	-2(1)
C(160)	29(2)	38(2)	15(1)	-4(1)	5(1)	-10(1)
N(161)	17(1)	15(1)	16(1)	0(1)	2(1)	2(1)
C(162)	17(1)	20(1)	20(1)	-2(1)	2(1)	3(1)
C(163)	24(2)	44(2)	39(2)	-12(2)	16(2)	5(2)
Sb(20)	23(1)	22(1)	23(1)	2(1)	6(1)	1(1)
F(201)	27(1)	85(3)	56(2)	-3(2)	7(1)	14(2)
F(202)	48(2)	43(2)	35(2)	-12(1)	9(1)	-2(1)
F(203)	82(3)	34(2)	54(2)	7(1)	35(2)	-11(2)
F(204)	63(2)	48(2)	34(2)	-11(1)	7(2)	-4(2)
F(205)	118(4)	39(2)	43(2)	15(2)	13(2)	-23(2)
F(206)	36(2)	98(3)	57(2)	-17(2)	4(2)	26(2)
Sb(30)	26(1)	15(1)	14(1)	0(1)	-2(1)	3(1)
F(301)	33(1)	28(1)	56(2)	6(1)	17(1)	-1(1)
F(302)	28(1)	39(1)	40(2)	9(1)	2(1)	14(1)
F(303)	74(2)	43(2)	16(1)	-4(1)	-3(1)	14(2)
F(304)	56(2)	40(2)	31(1)	1(1)	0(1)	32(1)
F(305)	51(2)	35(1)	19(1)	-8(1)	2(1)	6(1)
F(306)	68(2)	31(1)	56(2)	5(1)	1(2)	-22(2)
Sb(40)	26(1)	20(1)	31(1)	2(1)	13(1)	-1(1)
F(401)	37(2)	76(3)	77(3)	-8(2)	22(2)	-23(2)
F(402)	100(4)	36(2)	94(4)	-26(2)	25(3)	2(2)
F(403)	80(3)	81(3)	35(2)	16(2)	17(2)	7(2)
F(404)	55(2)	29(2)	124(4)	-18(2)	25(3)	4(2)
F(405)	58(2)	106(4)	49(2)	35(2)	15(2)	3(2)
F(406)	26(1)	77(3)	77(3)	0(2)	18(2)	-6(2)
Sb(50)	23(1)	16(1)	14(1)	-1(1)	3(1)	-4(1)
F(501)	30(2)	36(3)	31(2)	-14(2)	9(1)	2(2)
F(502)	41(3)	78(5)	42(3)	-18(3)	-10(2)	21(3)
F(503)	68(4)	21(2)	80(5)	5(3)	50(3)	-3(2)
F(504)	64(5)	103(8)	30(4)	-15(4)	-14(3)	34(5)
F(505)	105(9)	44(3)	91(9)	-25(5)	67(7)	-42(5)
F(506)	45(2)	47(2)	41(3)	-23(2)	18(2)	-8(2)
Sb(51)	23(1)	16(1)	14(1)	-1(1)	3(1)	-4(1)

F(511)	30(2)	36(3)	31(2)	-14(2)	9(1)	2(2)
F(512)	41(3)	78(5)	42(3)	-18(3)	-10(2)	21(3)
F(513)	68(4)	21(2)	80(5)	5(3)	50(3)	-3(2)
F(514)	64(5)	103(8)	30(4)	-15(4)	-14(3)	34(5)
F(515)	105(9)	44(3)	91(9)	-25(5)	67(7)	-42(5)
F(516)	45(2)	47(2)	41(3)	-23(2)	18(2)	-8(2)
Sb(52)	23(1)	16(1)	14(1)	-1(1)	3(1)	-4(1)
F(521)	30(2)	36(3)	31(2)	-14(2)	9(1)	2(2)
F(522)	41(3)	78(5)	42(3)	-18(3)	-10(2)	21(3)
F(523)	68(4)	21(2)	80(5)	5(3)	50(3)	-3(2)
F(524)	64(5)	103(8)	30(4)	-15(4)	-14(3)	34(5)
F(525)	105(9)	44(3)	91(9)	-25(5)	67(7)	-42(5)
F(526)	45(2)	47(2)	41(3)	-23(2)	18(2)	-8(2)
C(700)	63(4)	59(4)	38(3)	-3(3)	7(3)	6(3)
Cl(71)	46(1)	91(1)	61(1)	-7(1)	-12(1)	5(1)
Cl(72)	34(1)	74(1)	34(1)	22(1)	9(1)	9(1)
C(800)	34(2)	28(2)	26(2)	-3(1)	-2(2)	7(2)
Cl(81)	28(1)	82(1)	28(1)	2(1)	0(1)	13(1)
Cl(82)	39(1)	48(1)	62(1)	-22(1)	2(1)	-4(1)
C(900)	36(2)	39(2)	47(3)	-4(2)	-3(2)	-5(2)
Cl(91)	34(1)	64(1)	39(1)	-20(1)	9(1)	-12(1)
Cl(92)	53(1)	73(1)	86(1)	37(1)	-30(1)	-20(1)
C(600)	35(2)	31(2)	26(2)	-3(2)	7(2)	-2(2)
Cl(61)	39(1)	34(1)	24(1)	2(1)	2(1)	5(1)
Cl(62)	54(1)	72(1)	111(2)	-59(2)	26(1)	2(1)
C(650)	35(2)	31(2)	26(2)	-3(2)	7(2)	-2(2)
Cl(66)	39(1)	34(1)	24(1)	2(1)	2(1)	5(1)
Cl(67)	54(1)	72(1)	111(2)	-59(2)	26(1)	2(1)

Table S19 – Hydrogen coordinates (x 10^4) and isotropic displacement parameters ($Å^2$x 10 ^{3}) for CCDC 2110200.

	x	y	z	U(eq)
H(10A)	9485	2460	7192	28
H(10B)	9341	3355	7269	28
H(11A)	10356	2646	6474	31
H(11B)	10025	3509	6374	31
H(12A)	11221	2967	7407	27
H(12B)	10882	3827	7316	27
H(14)	12923	3703	7633	24
H(15A)	12942	5065	7641	32
H(15B)	11943	4714	7641	32
H(16A)	11647	5693	6996	40
H(16B)	12534	5522	6712	40
H(17A)	11338	5168	6003	40
H(17B)	10790	4788	6460	40
H(18)	11274	3806	5928	32
H(19A)	12799	3603	5803	33
H(19B)	12551	4458	5606	33
H(20A)	13464	4946	6420	30
H(20B)	14069	4286	6209	30
H(21A)	14128	4332	7258	30
H(21B)	13897	3514	6971	30
H(24A)	12294	1038	6431	64
H(24B)	13360	1254	6636	64
H(24C)	12861	1390	5970	64
H(26)	7602	1787	4148	25
H(27)	8277	2062	3327	35
H(28)	8009	3216	2848	34
H(29)	7081	4128	3186	30
H(30)	6404	3873	4006	24
H(32)	4955	1854	5198	25
H(33)	3344	1813	4949	34
H(34)	2574	2420	4115	38
H(35)	3393	3092	3512	35

H(36)	5018	3150	3750	25
H(37A)	6934	777	4584	28
H(37B)	5949	1151	4337	28
H(37C)	6113	696	4945	28
H(39)	7830	-376	5824	24
H(40)	8535	-1546	6105	29
H(41)	9963	-1580	6747	33
H(42)	10698	-455	7092	40
H(43)	9984	714	6836	32
H(56A)	6569	3699	7297	40
H(56B)	7594	3618	7167	40
H(56C)	6880	2929	7025	40
H(57A)	7722	5248	6611	40
H(57B)	8158	4994	6059	40
H(57C)	8315	4484	6639	40
H(58A)	6263	5622	5302	37
H(58B)	6230	5022	4780	37
H(58C)	7197	5238	5187	37
H(59A)	4410	4542	5181	30
H(59B)	4404	3642	5087	30
H(59C)	4934	4179	4702	30
H(60A)	4935	3123	6560	34
H(60B)	5480	2469	6288	34
H(60C)	4627	2889	5885	34
H(63A)	9923	4485	5269	53
H(63B)	9382	4589	4608	53
H(63C)	9918	3814	4803	53
H(11C)	372	7082	2768	23
H(11D)	324	7979	2660	23
H(11E)	-675	7160	3400	24
H(11F)	-1119	7559	2795	24
H(11G)	-585	8760	3272	25
H(11H)	-399	8297	3874	25
H(114)	-2519	8505	2647	27
H(11I)	-2621	9873	2563	40
H(11J)	-1590	9556	2717	40
H(11K)	-1528	10566	3328	50

H(11L)	-2542	10420	3456	50
H(11M)	-1620	10136	4320	41
H(11N)	-871	9722	4013	41
H(118)	-1576	8788	4472	28
H(11O)	-3139	8542	4327	33
H(11P)	-2998	9409	4522	33
H(12C)	-3598	9838	3604	32
H(12D)	-4287	9189	3737	32
H(12E)	-3928	9138	2759	31
H(12F)	-3786	8352	3108	31
H(12G)	-2802	6176	4224	44
H(12H)	-2374	5916	3670	44
H(12I)	-3422	6200	3579	44
H(126)	4730	6979	4733	25
H(127)	6345	6942	4927	31
H(128)	7176	7566	5759	33
H(129)	6377	8202	6400	31
H(130)	4765	8240	6204	23
H(132)	3413	9030	5907	27
H(133)	2846	9404	6742	37
H(134)	1967	8557	7185	43
H(135)	1601	7356	6779	39
H(136)	2188	6969	5953	29
H(13A)	3717	5791	5237	30
H(13B)	2843	5868	5552	30
H(13C)	3771	6327	5800	30
H(139)	-67	5395	3252	33
H(140)	-653	4168	3047	43
H(141)	80	3126	3554	37
H(142)	1422	3302	4251	31
H(143)	2024	4525	4461	25
H(15C)	1602	10045	3230	46
H(15D)	1222	9790	3804	46
H(15E)	1110	9238	3247	46
H(15F)	2968	10628	4436	47
H(15G)	3285	10125	5013	47
H(15H)	2223	10137	4696	47

H(15I)	5090	9629	4610	37
H(15J)	5111	8762	4818	37
H(15K)	4580	9384	5134	37
H(15L)	4688	8185	3354	36
H(15M)	4170	7488	3595	36
H(15N)	4970	7932	4027	36
H(16C)	2949	8484	2611	41
H(16D)	1927	8396	2744	41
H(16E)	2674	7742	2939	41
H(16F)	114	8637	5532	52
H(16G)	-248	9239	5025	52
H(16H)	534	9476	5570	52
H(70A)	5442	-1021	7659	64
H(70B)	5399	-1464	8256	64
H(80A)	4197	3732	1659	36
H(80B)	3883	4030	2245	36
H(90A)	1421	3369	1920	51
H(90B)	712	3483	2359	51
H(60D)	9066	-1205	7574	37
H(60E)	8971	-1696	8141	37
H(65A)	8902	-1522	7741	37
H(65B)	8289	-1472	8243	37

2. Molecular Structure of 6 (CCDC 2117074)

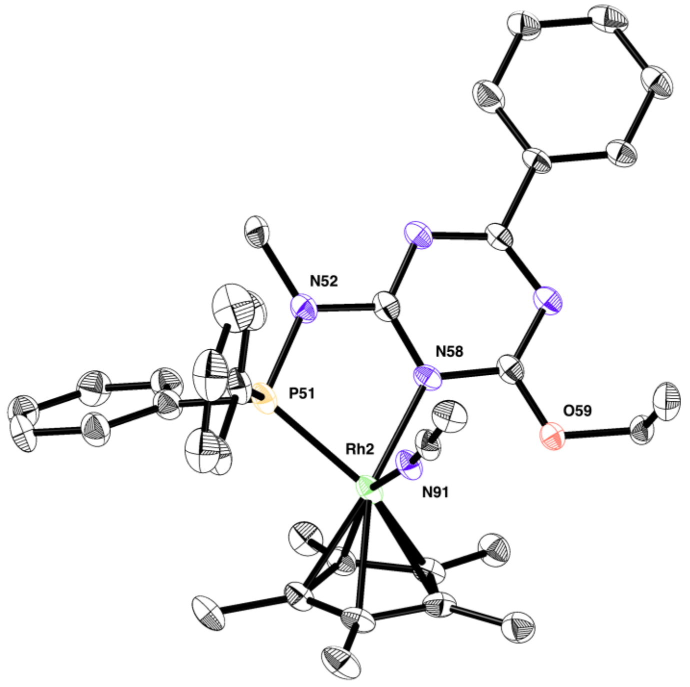

Molecular structure of complex **6**. Suitable single crystals were obtained by slow diffusion of a concentrated solution of **6** in DCM into *n*-pentane. The molecular structure is shown with thermal ellipsoids drawn at the 30% probability level. For clarity reasons, the hydrogen atoms and SbF_6 anions were omitted.

Table S20 – Crystal data and structure refinement for **6** (CCDC 2117074).

Identification code	**6**, CCDC 2117074	
Empirical formula	$C_{36.50} H_{42} Cl F_{12} N_5 O P Rh Sb_2$	
Formula weight	1207.58	
Temperature	100(2) K	
Wavelength	0.71073 Å	
Crystal system	Triclinic	
Space group	P-1 ; No.2	
Unit cell dimensions	a = 15.8221(14) Å	a= 103.364(4)°.
	b = 16.8828(14) Å	b= 90.994(4)°.
	c = 18.944(2) Å	g = 100.471(4)°.
Volume	4832.0(8) $Å^3$	
Z	4	
Density (calculated)	1.660 Mg/m^3	
Absorption coefficient	1.615 mm^{-1}	
F(000)	2364	
Crystal size	0.140 x 0.020 x 0.020 mm^3	
Theta range for data collection	1.850 to 26.683°.	
Index ranges	-19<=h<=19, -21<=k<=21, -23<=l<=23	
Reflections collected	196914	
Independent reflections	20021 [R(int) = 0.1044]	
Completeness to theta = 25.242°	100.0 %	
Absorption correction	Semi-empirical from equivalents	
Max. and min. transmission	0.9420 and 0.7809	
Refinement method	Full-matrix least-squares on F^2	
Data / restraints / parameters	20021 / 403 / 1127	
Goodness-of-fit on F^2	1.067	
Final R indices [I>2sigma(I)]	R1 = 0.0519, wR2 = 0.1119	
R indices (all data)	R1 = 0.0906, wR2 = 0.1335	
Extinction coefficient	n/a	
Largest diff. peak and hole	1.401 and -0.959 e.$Å^{-3}$	

Table S21 – Atomic coordinates (x 10^4) and equivalent isotropic displacement parameters (Å^{2}x 10^3) for CCDC 2117074. U(eq) is defined as one-third of the trace of the orthogonalized U^{ij} tensor.

	x	y	z	U(eq)
Rh(1)	4846(1)	5470(1)	2319(1)	34(1)
P(1)	4835(1)	6752(1)	2106(1)	36(1)
N(2)	4844(3)	7356(3)	2961(3)	35(1)
C(3)	5022(4)	7037(4)	3534(4)	32(1)
N(4)	5099(3)	7526(3)	4192(3)	34(1)
C(5)	5329(4)	7202(4)	4724(4)	34(2)
N(6)	5561(3)	6462(4)	4626(3)	36(1)
C(7)	5438(4)	6005(4)	3948(4)	36(2)
N(8)	5106(3)	6229(3)	3382(3)	33(1)
O(9)	5635(3)	5263(3)	3773(2)	39(1)
C(10)	6125(5)	4998(5)	4315(4)	47(2)
C(11)	7079(5)	5310(5)	4276(5)	56(2)
C(12)	3942(4)	6978(4)	1648(4)	41(2)
C(13)	3161(4)	7012(5)	1991(4)	43(2)
C(14)	2465(5)	7138(5)	1630(4)	48(2)
C(15)	2506(5)	7233(5)	929(5)	54(2)
C(16)	3270(5)	7206(5)	580(4)	56(2)
C(17)	3983(5)	7085(5)	941(4)	46(2)
C(18)	5772(4)	7180(7)	1685(5)	35(2)
C(19)	6301(6)	7935(7)	2004(5)	39(3)
C(20)	7029(6)	8218(6)	1666(7)	49(3)
C(21)	7227(5)	7745(7)	1008(5)	47(2)
C(22)	6698(7)	6990(7)	689(4)	47(3)
C(23)	5970(6)	6707(7)	1027(5)	44(3)
C(18')	5804(8)	7102(13)	1659(9)	35(2)
C(19')	6139(10)	7943(11)	1757(11)	39(3)
C(20')	6845(11)	8201(10)	1384(12)	49(3)
C(21')	7215(9)	7619(13)	912(10)	47(2)
C(22')	6880(12)	6778(12)	813(10)	47(3)
C(23')	6174(12)	6520(10)	1187(11)	44(3)
C(24)	4703(5)	8223(4)	3125(4)	45(2)
C(25)	5345(4)	7703(4)	5471(4)	35(2)

C(26)	4875(5)	8330(5)	5622(4)	50(2)
C(27)	4845(6)	8773(6)	6332(5)	67(3)
C(28)	5319(6)	8606(6)	6890(4)	60(2)
C(29)	5775(5)	7991(5)	6739(4)	51(2)
C(30)	5784(4)	7530(5)	6035(4)	46(2)
C(31)	4013(4)	4390(4)	2600(4)	38(2)
C(32)	3526(4)	4786(4)	2186(4)	36(2)
C(33)	3871(4)	4723(4)	1478(4)	39(2)
C(34)	4600(4)	4329(4)	1495(4)	44(2)
C(35)	4679(4)	4111(4)	2176(4)	43(2)
C(36)	3803(4)	4296(5)	3345(4)	45(2)
C(37)	2686(4)	5045(4)	2371(4)	38(2)
C(38)	3463(5)	4928(5)	842(4)	49(2)
C(39)	5136(5)	4083(5)	847(5)	57(2)
C(40)	5310(4)	3629(4)	2378(5)	51(2)
N(41)	6182(4)	5654(4)	2282(3)	46(2)
C(42)	6909(5)	5794(5)	2294(5)	50(2)
C(43)	7841(4)	6023(5)	2295(5)	64(2)
Rh(2)	-84(1)	1803(1)	3259(1)	32(1)
P(51)	-629(1)	813(1)	2231(1)	35(1)
N(52)	-391(3)	1306(3)	1555(3)	36(1)
C(53)	-54(4)	2144(4)	1745(4)	32(1)
N(54)	87(3)	2539(3)	1217(3)	32(1)
C(55)	393(4)	3354(4)	1438(3)	29(1)
N(56)	482(3)	3785(3)	2124(3)	31(1)
C(57)	305(4)	3341(4)	2619(4)	31(1)
N(58)	103(3)	2499(3)	2460(3)	29(1)
O(59)	321(3)	3701(3)	3322(2)	34(1)
C(60)	419(5)	4601(4)	3534(4)	39(2)
C(61)	-438(5)	4844(5)	3503(4)	50(2)
C(62)	-230(4)	-139(4)	1918(4)	40(2)
C(63)	571(5)	-99(5)	1619(4)	45(2)
C(64)	892(5)	-818(5)	1376(4)	52(2)
C(65)	423(5)	-1564(5)	1429(4)	52(2)
C(66)	-370(5)	-1616(5)	1728(5)	52(2)
C(67)	-698(5)	-904(4)	1975(4)	47(2)
C(68)	-1792(4)	531(4)	2227(4)	42(2)

C(69)	-2325(5)	678(5)	1697(5)	59(2)
C(70)	-3237(6)	490(6)	1768(6)	78(3)
C(71)	-3560(6)	188(6)	2320(6)	74(3)
C(72)	-3032(5)	56(5)	2852(5)	64(2)
C(73)	-2144(5)	225(4)	2800(4)	49(2)
C(74)	-529(5)	901(4)	774(4)	42(2)
C(75)	638(4)	3815(4)	871(3)	32(1)
C(76)	706(5)	3395(5)	163(4)	49(2)
C(77)	946(6)	3825(5)	-369(4)	55(2)
C(78)	1109(4)	4677(5)	-185(4)	48(2)
C(79)	1045(4)	5104(5)	520(4)	44(2)
C(80)	811(4)	4672(4)	1049(4)	35(2)
C(81)	-39(4)	1569(5)	4331(4)	39(2)
C(82)	407(4)	1020(4)	3874(4)	38(2)
C(83)	1109(4)	1504(4)	3600(4)	36(2)
C(84)	1127(4)	2368(4)	3957(4)	38(2)
C(85)	419(4)	2414(5)	4386(3)	38(2)
C(86)	-797(5)	1329(5)	4758(4)	54(2)
C(87)	253(5)	91(5)	3754(5)	53(2)
C(88)	1819(4)	1196(5)	3185(4)	44(2)
C(89)	1822(4)	3066(5)	3876(4)	45(2)
C(90)	187(5)	3171(5)	4865(4)	49(2)
N(91)	-1274(3)	2176(3)	3358(3)	34(1)
C(92)	-1921(4)	2359(4)	3346(4)	35(2)
C(93)	-2781(4)	2568(5)	3305(4)	45(2)
Sb(1)	2473(1)	6510(1)	4535(1)	36(1)
F(1)	1462(2)	6476(3)	3995(2)	49(1)
F(2)	1808(2)	6250(3)	5293(2)	47(1)
F(3)	2546(3)	7635(2)	4953(2)	56(1)
F(4)	3125(3)	6747(3)	3764(2)	51(1)
F(5)	2393(2)	5370(2)	4143(2)	45(1)
F(6)	3481(2)	6517(3)	5066(2)	50(1)
Sb(2)	7923(5)	8089(5)	4015(6)	44(1)
F(7)	7445(11)	8103(12)	4908(8)	66(2)
F(8)	6921(8)	7388(8)	3536(11)	62(3)
F(9)	7503(10)	9019(7)	3935(8)	90(3)
F(10)	8939(8)	8761(9)	4497(8)	88(4)

F(11)	8372(8)	7152(7)	4075(10)	71(3)
F(12)	8423(11)	8054(11)	3117(7)	62(3)
Sb(2')	7813(6)	8124(6)	4048(6)	44(1)
F(7')	7395(12)	8021(13)	4943(9)	66(2)
F(8')	7037(10)	7158(8)	3583(12)	62(3)
F(9')	7020(10)	8765(9)	3916(9)	90(3)
F(10')	8612(10)	9069(8)	4520(10)	88(4)
F(11')	8619(8)	7469(9)	4158(11)	71(3)
F(12')	8260(12)	8214(13)	3152(8)	62(3)
Sb(3)	-2147(1)	3532(1)	1558(1)	50(1)
F(13)	-2493(3)	4118(3)	2442(2)	52(1)
F(14)	-2750(3)	4121(4)	1065(3)	70(1)
F(15)	-3136(3)	2709(3)	1484(3)	72(2)
F(16)	-1549(2)	2965(3)	2072(2)	53(1)
F(17)	-1176(3)	4373(3)	1637(3)	64(1)
F(18)	-1794(3)	2963(4)	688(3)	80(2)
Sb(4)	2642(2)	2095(2)	1254(1)	40(1)
F(20)	2998(5)	1235(4)	1583(4)	72(2)
F(21)	1545(3)	1743(5)	1552(4)	73(2)
F(22)	2383(6)	1398(5)	341(3)	104(3)
F(23)	3757(4)	2452(5)	992(5)	91(2)
F(24)	2935(5)	2764(4)	2188(3)	82(2)
F(25)	2303(5)	2963(4)	952(5)	79(2)
Sb(4')	2550(5)	1996(5)	1116(4)	40(1)
F(20')	2583(11)	956(7)	1275(9)	72(2)
F(21')	1742(9)	2154(10)	1822(8)	73(2)
F(22')	1697(10)	1555(11)	383(7)	104(3)
F(23')	3398(9)	1859(11)	444(8)	91(2)
F(24')	3422(9)	2462(10)	1852(7)	82(2)
F(25')	2563(12)	3056(7)	967(11)	79(2)
C(101)	253(5)	7289(6)	3150(4)	59(2)
Cl(1)	135(1)	6527(1)	2308(1)	59(1)
Cl(2)	1169(2)	8079(1)	3181(1)	66(1)

Table S22 – Bond lengths [Å] and angles [°] for CCDC 2117074.

Rh(1)-N(41)	2.085(6)
Rh(1)-N(8)	2.109(6)
Rh(1)-C(34)	2.148(7)
Rh(1)-C(32)	2.175(6)
Rh(1)-C(33)	2.189(7)
Rh(1)-C(35)	2.213(7)
Rh(1)-C(31)	2.225(6)
Rh(1)-P(1)	2.2948(19)
P(1)-N(2)	1.701(6)
P(1)-C(12)	1.789(7)
P(1)-C(18)	1.809(5)
P(1)-C(18')	1.834(7)
N(2)-C(3)	1.366(8)
N(2)-C(24)	1.483(8)
C(3)-N(4)	1.315(8)
C(3)-N(8)	1.358(8)
N(4)-C(5)	1.327(8)
C(5)-N(6)	1.339(8)
C(5)-C(25)	1.469(9)
N(6)-C(7)	1.328(9)
C(7)-O(9)	1.315(8)
C(7)-N(8)	1.345(8)
O(9)-C(10)	1.472(8)
C(10)-C(11)	1.514(10)
C(12)-C(17)	1.394(10)
C(12)-C(13)	1.410(9)
C(13)-C(14)	1.360(10)
C(14)-C(15)	1.376(11)
C(15)-C(16)	1.390(11)
C(16)-C(17)	1.379(11)
C(18)-C(19)	1.3900
C(18)-C(23)	1.3900
C(19)-C(20)	1.3900
C(20)-C(21)	1.3900
C(21)-C(22)	1.3900

C(22)-C(23)	1.3900
C(18')-C(19')	1.3900
C(18')-C(23')	1.3900
C(19')-C(20')	1.3900
C(20')-C(21')	1.3900
C(21')-C(22')	1.3900
C(22')-C(23')	1.3900
C(25)-C(30)	1.379(9)
C(25)-C(26)	1.382(10)
C(26)-C(27)	1.386(11)
C(27)-C(28)	1.394(12)
C(28)-C(29)	1.350(11)
C(29)-C(30)	1.382(11)
C(31)-C(35)	1.419(9)
C(31)-C(32)	1.439(10)
C(31)-C(36)	1.492(9)
C(32)-C(33)	1.445(9)
C(32)-C(37)	1.495(8)
C(33)-C(34)	1.436(10)
C(33)-C(38)	1.495(10)
C(34)-C(35)	1.431(11)
C(34)-C(39)	1.526(9)
C(35)-C(40)	1.493(10)
N(41)-C(42)	1.129(9)
C(42)-C(43)	1.455(9)
Rh(2)-N(91)	2.089(5)
Rh(2)-N(58)	2.115(5)
Rh(2)-C(81)	2.159(6)
Rh(2)-C(83)	2.166(7)
Rh(2)-C(82)	2.182(7)
Rh(2)-C(85)	2.210(7)
Rh(2)-C(84)	2.236(7)
Rh(2)-P(51)	2.288(2)
P(51)-N(52)	1.694(5)
P(51)-C(62)	1.810(7)
P(51)-C(68)	1.814(7)
N(52)-C(53)	1.378(8)

N(52)-C(74)	1.473(9)
C(53)-N(54)	1.327(8)
C(53)-N(58)	1.347(8)
N(54)-C(55)	1.337(8)
C(55)-N(56)	1.325(8)
C(55)-C(75)	1.484(8)
N(56)-C(57)	1.334(8)
C(57)-O(59)	1.328(8)
C(57)-N(58)	1.358(8)
O(59)-C(60)	1.458(7)
C(60)-C(61)	1.490(10)
C(62)-C(63)	1.394(9)
C(62)-C(67)	1.395(10)
C(63)-C(64)	1.385(10)
C(64)-C(65)	1.367(11)
C(65)-C(66)	1.383(11)
C(66)-C(67)	1.382(10)
C(68)-C(73)	1.390(10)
C(68)-C(69)	1.394(11)
C(69)-C(70)	1.435(11)
C(70)-C(71)	1.337(14)
C(71)-C(72)	1.379(14)
C(72)-C(73)	1.392(10)
C(75)-C(76)	1.382(10)
C(75)-C(80)	1.382(9)
C(76)-C(77)	1.393(10)
C(77)-C(78)	1.374(11)
C(78)-C(79)	1.379(11)
C(79)-C(80)	1.387(9)
C(81)-C(82)	1.412(9)
C(81)-C(85)	1.457(10)
C(81)-C(86)	1.506(9)
C(82)-C(83)	1.434(9)
C(82)-C(87)	1.506(10)
C(83)-C(84)	1.455(10)
C(83)-C(88)	1.484(9)
C(84)-C(85)	1.398(9)

C(84)-C(89)	1.496(9)
C(85)-C(90)	1.497(10)
N(91)-C(92)	1.124(8)
C(92)-C(93)	1.472(9)
Sb(1)-F(3)	1.863(4)
Sb(1)-F(6)	1.867(3)
Sb(1)-F(1)	1.869(3)
Sb(1)-F(5)	1.876(3)
Sb(1)-F(4)	1.879(3)
Sb(1)-F(2)	1.880(3)
Sb(2)-F(9)	1.848(6)
Sb(2)-F(7)	1.863(6)
Sb(2)-F(8)	1.873(6)
Sb(2)-F(11)	1.873(6)
Sb(2)-F(10)	1.874(6)
Sb(2)-F(12)	1.880(6)
Sb(2')-F(9')	1.850(7)
Sb(2')-F(7')	1.864(6)
Sb(2')-F(11')	1.871(6)
Sb(2')-F(10')	1.874(6)
Sb(2')-F(8')	1.875(6)
Sb(2')-F(12')	1.878(6)
Sb(3)-F(18)	1.857(5)
Sb(3)-F(16)	1.868(4)
Sb(3)-F(17)	1.871(5)
Sb(3)-F(15)	1.873(5)
Sb(3)-F(14)	1.876(4)
Sb(3)-F(13)	1.883(4)
Sb(4)-F(22)	1.845(6)
Sb(4)-F(25)	1.852(5)
Sb(4)-F(24)	1.865(5)
Sb(4)-F(21)	1.871(5)
Sb(4)-F(23)	1.873(5)
Sb(4)-F(20)	1.874(5)
Sb(4')-F(22')	1.843(7)
Sb(4')-F(20')	1.858(7)
Sb(4')-F(24')	1.871(7)

Sb(4')-F(25')	1.871(7)
Sb(4')-F(23')	1.873(7)
Sb(4')-F(21')	1.875(7)
C(101)-Cl(2)	1.772(9)
C(101)-Cl(1)	1.786(9)

N(41)-Rh(1)-N(8)	84.6(2)
N(41)-Rh(1)-C(34)	95.3(2)
N(8)-Rh(1)-C(34)	156.4(3)
N(41)-Rh(1)-C(32)	157.5(2)
N(8)-Rh(1)-C(32)	110.2(2)
C(34)-Rh(1)-C(32)	64.2(2)
N(41)-Rh(1)-C(33)	128.2(2)
N(8)-Rh(1)-C(33)	147.2(2)
C(34)-Rh(1)-C(33)	38.6(3)
C(32)-Rh(1)-C(33)	38.7(2)
N(41)-Rh(1)-C(35)	94.5(2)
N(8)-Rh(1)-C(35)	118.1(3)
C(34)-Rh(1)-C(35)	38.3(3)
C(32)-Rh(1)-C(35)	63.8(2)
C(33)-Rh(1)-C(35)	64.4(3)
N(41)-Rh(1)-C(31)	125.7(2)
N(8)-Rh(1)-C(31)	97.7(2)
C(34)-Rh(1)-C(31)	63.2(2)
C(32)-Rh(1)-C(31)	38.1(2)
C(33)-Rh(1)-C(31)	64.0(3)
C(35)-Rh(1)-C(31)	37.3(2)
N(41)-Rh(1)-P(1)	91.06(18)
N(8)-Rh(1)-P(1)	79.16(15)
C(34)-Rh(1)-P(1)	124.4(2)
C(32)-Rh(1)-P(1)	107.94(19)
C(33)-Rh(1)-P(1)	99.01(19)
C(35)-Rh(1)-P(1)	162.2(2)
C(31)-Rh(1)-P(1)	142.93(17)
N(2)-P(1)-C(12)	102.8(3)
N(2)-P(1)-C(18)	107.7(4)
C(12)-P(1)-C(18)	104.8(4)

N(2)-P(1)-C(18')	110.6(7)
C(12)-P(1)-C(18')	107.1(6)
N(2)-P(1)-Rh(1)	102.35(19)
C(12)-P(1)-Rh(1)	122.7(2)
C(18)-P(1)-Rh(1)	115.0(4)
C(18')-P(1)-Rh(1)	110.6(7)
C(3)-N(2)-C(24)	117.5(5)
C(3)-N(2)-P(1)	118.3(5)
C(24)-N(2)-P(1)	124.2(4)
N(4)-C(3)-N(8)	124.6(6)
N(4)-C(3)-N(2)	118.3(6)
N(8)-C(3)-N(2)	117.1(6)
C(3)-N(4)-C(5)	115.9(6)
N(4)-C(5)-N(6)	124.6(6)
N(4)-C(5)-C(25)	117.3(6)
N(6)-C(5)-C(25)	118.1(6)
C(7)-N(6)-C(5)	114.7(6)
O(9)-C(7)-N(6)	121.3(6)
O(9)-C(7)-N(8)	113.4(6)
N(6)-C(7)-N(8)	125.4(6)
C(7)-N(8)-C(3)	113.4(6)
C(7)-N(8)-Rh(1)	125.0(5)
C(3)-N(8)-Rh(1)	121.2(4)
C(7)-O(9)-C(10)	118.6(6)
O(9)-C(10)-C(11)	109.7(6)
C(17)-C(12)-C(13)	118.6(7)
C(17)-C(12)-P(1)	121.5(6)
C(13)-C(12)-P(1)	119.9(5)
C(14)-C(13)-C(12)	120.1(7)
C(13)-C(14)-C(15)	121.1(7)
C(14)-C(15)-C(16)	119.9(7)
C(17)-C(16)-C(15)	119.7(7)
C(16)-C(17)-C(12)	120.6(7)
C(19)-C(18)-C(23)	120.0
C(19)-C(18)-P(1)	122.7(6)
C(23)-C(18)-P(1)	117.3(6)
C(20)-C(19)-C(18)	120.0

C(19)-C(20)-C(21)	120.0
C(20)-C(21)-C(22)	120.0
C(21)-C(22)-C(23)	120.0
C(22)-C(23)-C(18)	120.0
C(19')-C(18')-C(23')	120.0
C(19')-C(18')-P(1)	120.6(12)
C(23')-C(18')-P(1)	119.3(12)
C(18')-C(19')-C(20')	120.0
C(21')-C(20')-C(19')	120.0
C(22')-C(21')-C(20')	120.0
C(23')-C(22')-C(21')	120.0
C(22')-C(23')-C(18')	120.0
C(30)-C(25)-C(26)	119.1(7)
C(30)-C(25)-C(5)	121.1(6)
C(26)-C(25)-C(5)	119.7(6)
C(25)-C(26)-C(27)	120.0(7)
C(26)-C(27)-C(28)	119.8(8)
C(29)-C(28)-C(27)	119.8(8)
C(28)-C(29)-C(30)	120.6(7)
C(25)-C(30)-C(29)	120.5(7)
C(35)-C(31)-C(32)	108.5(6)
C(35)-C(31)-C(36)	128.4(6)
C(32)-C(31)-C(36)	123.1(6)
C(35)-C(31)-Rh(1)	70.9(4)
C(32)-C(31)-Rh(1)	69.1(3)
C(36)-C(31)-Rh(1)	126.6(5)
C(31)-C(32)-C(33)	108.4(6)
C(31)-C(32)-C(37)	126.4(6)
C(33)-C(32)-C(37)	123.8(6)
C(31)-C(32)-Rh(1)	72.8(4)
C(33)-C(32)-Rh(1)	71.2(4)
C(37)-C(32)-Rh(1)	132.5(5)
C(34)-C(33)-C(32)	105.8(6)
C(34)-C(33)-C(38)	128.2(6)
C(32)-C(33)-C(38)	125.5(6)
C(34)-C(33)-Rh(1)	69.1(4)
C(32)-C(33)-Rh(1)	70.2(4)

C(38)-C(33)-Rh(1)	131.2(5)
C(35)-C(34)-C(33)	109.9(6)
C(35)-C(34)-C(39)	125.1(7)
C(33)-C(34)-C(39)	124.6(7)
C(35)-C(34)-Rh(1)	73.3(4)
C(33)-C(34)-Rh(1)	72.2(4)
C(39)-C(34)-Rh(1)	127.0(5)
C(31)-C(35)-C(34)	107.2(6)
C(31)-C(35)-C(40)	126.7(7)
C(34)-C(35)-C(40)	126.0(6)
C(31)-C(35)-Rh(1)	71.8(4)
C(34)-C(35)-Rh(1)	68.4(4)
C(40)-C(35)-Rh(1)	128.0(5)
C(42)-N(41)-Rh(1)	176.0(6)
N(41)-C(42)-C(43)	176.2(9)
N(91)-Rh(2)-N(58)	84.99(19)
N(91)-Rh(2)-C(81)	94.6(2)
N(58)-Rh(2)-C(81)	157.2(2)
N(91)-Rh(2)-C(83)	158.0(2)
N(58)-Rh(2)-C(83)	111.7(2)
C(81)-Rh(2)-C(83)	64.4(2)
N(91)-Rh(2)-C(82)	126.2(2)
N(58)-Rh(2)-C(82)	148.8(2)
C(81)-Rh(2)-C(82)	37.9(3)
C(83)-Rh(2)-C(82)	38.5(2)
N(91)-Rh(2)-C(85)	95.9(2)
N(58)-Rh(2)-C(85)	118.3(2)
C(81)-Rh(2)-C(85)	39.0(3)
C(83)-Rh(2)-C(85)	64.0(2)
C(82)-Rh(2)-C(85)	63.9(3)
N(91)-Rh(2)-C(84)	127.6(2)
N(58)-Rh(2)-C(84)	99.2(2)
C(81)-Rh(2)-C(84)	63.2(2)
C(83)-Rh(2)-C(84)	38.6(2)
C(82)-Rh(2)-C(84)	63.4(3)
C(85)-Rh(2)-C(84)	36.7(2)
N(91)-Rh(2)-P(51)	89.67(16)

N(58)-Rh(2)-P(51)	78.89(15)
C(81)-Rh(2)-P(51)	123.9(2)
C(83)-Rh(2)-P(51)	107.04(19)
C(82)-Rh(2)-P(51)	99.3(2)
C(85)-Rh(2)-P(51)	162.22(19)
C(84)-Rh(2)-P(51)	142.63(18)
N(52)-P(51)-C(62)	102.6(3)
N(52)-P(51)-C(68)	108.0(3)
C(62)-P(51)-C(68)	107.0(3)
N(52)-P(51)-Rh(2)	103.4(2)
C(62)-P(51)-Rh(2)	123.7(2)
C(68)-P(51)-Rh(2)	110.9(2)
C(53)-N(52)-C(74)	117.5(5)
C(53)-N(52)-P(51)	118.1(4)
C(74)-N(52)-P(51)	124.4(4)
N(54)-C(53)-N(58)	125.0(6)
N(54)-C(53)-N(52)	118.1(6)
N(58)-C(53)-N(52)	116.9(5)
C(53)-N(54)-C(55)	115.1(6)
N(56)-C(55)-N(54)	125.2(5)
N(56)-C(55)-C(75)	117.3(6)
N(54)-C(55)-C(75)	117.5(6)
C(55)-N(56)-C(57)	115.5(5)
O(59)-C(57)-N(56)	121.5(6)
O(59)-C(57)-N(58)	114.4(5)
N(56)-C(57)-N(58)	124.1(6)
C(53)-N(58)-C(57)	114.1(5)
C(53)-N(58)-Rh(2)	122.1(4)
C(57)-N(58)-Rh(2)	123.5(4)
C(57)-O(59)-C(60)	118.1(5)
O(59)-C(60)-C(61)	110.0(5)
C(63)-C(62)-C(67)	119.6(7)
C(63)-C(62)-P(51)	118.6(5)
C(67)-C(62)-P(51)	121.8(5)
C(64)-C(63)-C(62)	119.8(7)
C(65)-C(64)-C(63)	120.0(7)
C(64)-C(65)-C(66)	121.1(7)

C(67)-C(66)-C(65)	119.6(7)
C(66)-C(67)-C(62)	119.9(7)
C(73)-C(68)-C(69)	120.3(7)
C(73)-C(68)-P(51)	118.3(6)
C(69)-C(68)-P(51)	121.2(6)
C(68)-C(69)-C(70)	117.3(8)
C(71)-C(70)-C(69)	121.2(10)
C(70)-C(71)-C(72)	121.5(8)
C(71)-C(72)-C(73)	119.0(8)
C(68)-C(73)-C(72)	120.7(8)
C(76)-C(75)-C(80)	119.2(6)
C(76)-C(75)-C(55)	120.5(6)
C(80)-C(75)-C(55)	120.3(6)
C(75)-C(76)-C(77)	120.7(7)
C(78)-C(77)-C(76)	119.3(7)
C(77)-C(78)-C(79)	120.5(7)
C(78)-C(79)-C(80)	119.9(7)
C(75)-C(80)-C(79)	120.3(7)
C(82)-C(81)-C(85)	108.2(6)
C(82)-C(81)-C(86)	126.3(7)
C(85)-C(81)-C(86)	125.1(6)
C(82)-C(81)-Rh(2)	71.9(4)
C(85)-C(81)-Rh(2)	72.4(4)
C(86)-C(81)-Rh(2)	126.6(5)
C(81)-C(82)-C(83)	108.3(6)
C(81)-C(82)-C(87)	126.8(6)
C(83)-C(82)-C(87)	124.6(6)
C(81)-C(82)-Rh(2)	70.2(4)
C(83)-C(82)-Rh(2)	70.1(4)
C(87)-C(82)-Rh(2)	130.7(5)
C(82)-C(83)-C(84)	107.0(6)
C(82)-C(83)-C(88)	127.0(6)
C(84)-C(83)-C(88)	124.4(6)
C(82)-C(83)-Rh(2)	71.4(4)
C(84)-C(83)-Rh(2)	73.3(4)
C(88)-C(83)-Rh(2)	132.1(5)
C(85)-C(84)-C(83)	108.7(6)

C(85)-C(84)-C(89)	127.7(7)
C(83)-C(84)-C(89)	123.6(6)
C(85)-C(84)-Rh(2)	70.7(4)
C(83)-C(84)-Rh(2)	68.1(4)
C(89)-C(84)-Rh(2)	128.5(5)
C(84)-C(85)-C(81)	107.6(6)
C(84)-C(85)-C(90)	127.7(7)
C(81)-C(85)-C(90)	124.6(6)
C(84)-C(85)-Rh(2)	72.7(4)
C(81)-C(85)-Rh(2)	68.7(4)
C(90)-C(85)-Rh(2)	127.2(5)
C(92)-N(91)-Rh(2)	173.9(6)
N(91)-C(92)-C(93)	177.6(8)
F(3)-Sb(1)-F(6)	90.24(19)
F(3)-Sb(1)-F(1)	91.05(19)
F(6)-Sb(1)-F(1)	178.71(19)
F(3)-Sb(1)-F(5)	178.22(18)
F(6)-Sb(1)-F(5)	89.11(18)
F(1)-Sb(1)-F(5)	89.61(18)
F(3)-Sb(1)-F(4)	91.39(19)
F(6)-Sb(1)-F(4)	90.08(17)
F(1)-Sb(1)-F(4)	90.01(17)
F(5)-Sb(1)-F(4)	90.27(17)
F(3)-Sb(1)-F(2)	89.74(18)
F(6)-Sb(1)-F(2)	90.51(17)
F(1)-Sb(1)-F(2)	89.38(17)
F(5)-Sb(1)-F(2)	88.61(17)
F(4)-Sb(1)-F(2)	178.73(19)
F(9)-Sb(2)-F(7)	91.6(5)
F(9)-Sb(2)-F(8)	91.4(5)
F(7)-Sb(2)-F(8)	90.2(4)
F(9)-Sb(2)-F(11)	178.3(5)
F(7)-Sb(2)-F(11)	90.0(5)
F(8)-Sb(2)-F(11)	89.1(4)
F(9)-Sb(2)-F(10)	90.4(5)
F(7)-Sb(2)-F(10)	89.6(5)
F(8)-Sb(2)-F(10)	178.2(5)

F(11)-Sb(2)-F(10)	89.1(5)
F(9)-Sb(2)-F(12)	89.9(5)
F(7)-Sb(2)-F(12)	178.5(5)
F(8)-Sb(2)-F(12)	90.1(4)
F(11)-Sb(2)-F(12)	88.5(4)
F(10)-Sb(2)-F(12)	90.0(4)
F(9')-Sb(2')-F(7')	91.5(5)
F(9')-Sb(2')-F(11')	178.6(6)
F(7')-Sb(2')-F(11')	89.8(5)
F(9')-Sb(2')-F(10')	91.2(5)
F(7')-Sb(2')-F(10')	89.7(5)
F(11')-Sb(2')-F(10')	89.3(5)
F(9')-Sb(2')-F(8')	90.5(5)
F(7')-Sb(2')-F(8')	90.0(5)
F(11')-Sb(2')-F(8')	89.0(5)
F(10')-Sb(2')-F(8')	178.2(6)
F(9')-Sb(2')-F(12')	90.0(5)
F(7')-Sb(2')-F(12')	178.4(6)
F(11')-Sb(2')-F(12')	88.7(5)
F(10')-Sb(2')-F(12')	90.0(5)
F(8')-Sb(2')-F(12')	90.2(5)
F(18)-Sb(3)-F(16)	90.4(2)
F(18)-Sb(3)-F(17)	90.1(2)
F(16)-Sb(3)-F(17)	90.82(19)
F(18)-Sb(3)-F(15)	90.7(3)
F(16)-Sb(3)-F(15)	90.2(2)
F(17)-Sb(3)-F(15)	178.7(2)
F(18)-Sb(3)-F(14)	91.0(2)
F(16)-Sb(3)-F(14)	178.5(2)
F(17)-Sb(3)-F(14)	88.8(2)
F(15)-Sb(3)-F(14)	90.2(2)
F(18)-Sb(3)-F(13)	179.3(2)
F(16)-Sb(3)-F(13)	89.53(19)
F(17)-Sb(3)-F(13)	89.2(2)
F(15)-Sb(3)-F(13)	89.9(2)
F(14)-Sb(3)-F(13)	89.0(2)
F(22)-Sb(4)-F(25)	91.8(4)

F(22)-Sb(4)-F(24)	177.2(4)
F(25)-Sb(4)-F(24)	90.8(4)
F(22)-Sb(4)-F(21)	91.9(4)
F(25)-Sb(4)-F(21)	92.3(3)
F(24)-Sb(4)-F(21)	88.8(3)
F(22)-Sb(4)-F(23)	89.9(4)
F(25)-Sb(4)-F(23)	88.8(3)
F(24)-Sb(4)-F(23)	89.4(4)
F(21)-Sb(4)-F(23)	177.9(4)
F(22)-Sb(4)-F(20)	89.6(3)
F(25)-Sb(4)-F(20)	178.5(4)
F(24)-Sb(4)-F(20)	87.8(3)
F(21)-Sb(4)-F(20)	87.9(3)
F(23)-Sb(4)-F(20)	90.9(3)
F(22')-Sb(4')-F(20')	91.9(6)
F(22')-Sb(4')-F(24')	178.8(7)
F(20')-Sb(4')-F(24')	89.2(6)
F(22')-Sb(4')-F(25')	90.1(6)
F(20')-Sb(4')-F(25')	177.7(7)
F(24')-Sb(4')-F(25')	88.8(6)
F(22')-Sb(4')-F(23')	90.6(6)
F(20')-Sb(4')-F(23')	89.7(6)
F(24')-Sb(4')-F(23')	89.0(6)
F(25')-Sb(4')-F(23')	89.1(6)
F(22')-Sb(4')-F(21')	92.0(6)
F(20')-Sb(4')-F(21')	90.3(5)
F(24')-Sb(4')-F(21')	88.4(6)
F(25')-Sb(4')-F(21')	90.9(6)
F(23')-Sb(4')-F(21')	177.3(7)
Cl(2)-C(101)-Cl(1)	111.2(4)

Table S23 – Anisotropic displacement parameters ($Å^2 x 10^3$) for CCDC 2117074. The anisotropic displacement factor exponent takes the form: $-2p^2[h^2 a^{*2}U^{11} + ... + 2 h k a^* b^* U^{12}]$.

	U^{11}	U^{22}	U^{33}	U^{23}	U^{13}	U^{12}
Rh(1)	27(1)	31(1)	45(1)	12(1)	12(1)	-1(1)
P(1)	36(1)	35(1)	37(1)	13(1)	7(1)	-1(1)
N(2)	41(3)	30(3)	34(3)	13(2)	7(2)	5(2)
C(3)	22(3)	39(4)	39(4)	16(3)	8(3)	5(3)
N(4)	32(3)	32(3)	42(3)	15(3)	9(2)	5(2)
C(5)	25(3)	40(4)	44(4)	24(3)	8(3)	1(3)
N(6)	28(3)	43(3)	45(3)	23(3)	13(2)	8(2)
C(7)	25(3)	38(4)	50(4)	19(3)	15(3)	4(3)
N(8)	21(2)	39(3)	44(3)	19(3)	8(2)	4(2)
O(9)	39(3)	39(3)	42(3)	14(2)	3(2)	13(2)
C(10)	45(4)	46(5)	62(5)	30(4)	11(4)	16(3)
C(11)	45(4)	67(6)	64(5)	32(4)	-4(4)	6(4)
C(12)	44(4)	35(4)	43(4)	15(3)	1(3)	0(3)
C(13)	44(4)	47(4)	41(4)	16(3)	11(3)	7(3)
C(14)	44(4)	57(5)	47(4)	17(4)	5(3)	13(4)
C(15)	45(4)	61(5)	61(5)	25(4)	4(4)	12(4)
C(16)	55(5)	68(6)	46(5)	24(4)	-2(4)	2(4)
C(17)	42(4)	55(5)	41(4)	15(4)	8(3)	4(3)
C(18)	36(3)	35(4)	35(4)	12(3)	4(3)	2(3)
C(19)	24(5)	43(4)	45(7)	7(5)	-5(5)	-4(4)
C(20)	33(5)	46(5)	56(8)	-1(5)	-1(5)	-6(4)
C(21)	42(4)	51(5)	44(5)	18(4)	4(3)	-11(3)
C(22)	55(6)	48(6)	37(5)	15(4)	14(4)	-1(5)
C(23)	51(6)	37(6)	37(6)	11(4)	3(4)	-9(4)
C(18')	36(3)	35(4)	35(4)	12(3)	4(3)	2(3)
C(19')	24(5)	43(4)	45(7)	7(5)	-5(5)	-4(4)
C(20')	33(5)	46(5)	56(8)	-1(5)	-1(5)	-6(4)
C(21')	42(4)	51(5)	44(5)	18(4)	4(3)	-11(3)
C(22')	55(6)	48(6)	37(5)	15(4)	14(4)	-1(5)
C(23')	51(6)	37(6)	37(6)	11(4)	3(4)	-9(4)
C(24)	69(5)	30(4)	39(4)	14(3)	10(4)	12(3)
C(25)	28(3)	42(4)	38(4)	20(3)	10(3)	2(3)

C(26)	63(5)	61(5)	34(4)	18(4)	5(3)	21(4)
C(27)	79(6)	80(7)	52(5)	16(5)	10(5)	36(5)
C(28)	68(6)	74(6)	39(4)	16(4)	13(4)	12(5)
C(29)	36(4)	71(6)	47(5)	25(4)	0(3)	2(4)
C(30)	34(4)	61(5)	48(4)	23(4)	7(3)	9(3)
C(31)	29(3)	28(4)	57(4)	15(3)	14(3)	-1(3)
C(32)	31(3)	30(4)	46(4)	12(3)	11(3)	-2(3)
C(33)	34(3)	30(4)	52(4)	8(3)	15(3)	-1(3)
C(34)	35(4)	35(4)	54(5)	8(3)	21(3)	-6(3)
C(35)	27(3)	36(4)	67(5)	15(4)	11(3)	5(3)
C(36)	33(4)	48(4)	64(5)	32(4)	19(3)	7(3)
C(37)	25(3)	41(4)	51(4)	15(3)	11(3)	3(3)
C(38)	44(4)	47(5)	49(4)	13(4)	4(3)	-9(3)
C(39)	45(4)	53(5)	65(5)	4(4)	28(4)	3(4)
C(40)	31(4)	34(4)	88(6)	15(4)	19(4)	2(3)
N(41)	39(4)	40(4)	56(4)	9(3)	17(3)	3(3)
C(42)	33(4)	43(4)	71(5)	12(4)	17(4)	2(3)
C(43)	26(4)	60(5)	94(7)	6(5)	20(4)	-4(3)
Rh(2)	32(1)	30(1)	40(1)	19(1)	13(1)	5(1)
P(51)	37(1)	27(1)	45(1)	17(1)	14(1)	4(1)
N(52)	39(3)	30(3)	39(3)	14(2)	7(2)	-1(2)
C(53)	23(3)	32(4)	42(4)	13(3)	7(3)	3(2)
N(54)	29(3)	33(3)	37(3)	16(2)	6(2)	5(2)
C(55)	20(3)	32(4)	38(4)	17(3)	5(2)	5(2)
N(56)	24(3)	34(3)	38(3)	16(2)	8(2)	3(2)
C(57)	24(3)	31(4)	41(4)	15(3)	6(3)	0(2)
N(58)	26(3)	34(3)	32(3)	16(2)	8(2)	6(2)
O(59)	39(2)	27(2)	34(2)	10(2)	3(2)	0(2)
C(60)	54(4)	26(4)	33(4)	6(3)	10(3)	-1(3)
C(61)	56(5)	33(4)	58(5)	5(3)	15(4)	6(3)
C(62)	43(4)	37(4)	42(4)	14(3)	8(3)	7(3)
C(63)	44(4)	46(4)	48(4)	18(4)	13(3)	5(3)
C(64)	51(5)	55(5)	51(5)	9(4)	11(4)	17(4)
C(65)	63(5)	46(5)	51(5)	7(4)	-1(4)	23(4)
C(66)	61(5)	28(4)	70(5)	19(4)	6(4)	9(3)
C(67)	51(4)	37(4)	52(5)	14(3)	9(4)	4(3)
C(68)	40(4)	27(4)	60(5)	14(3)	11(3)	2(3)

C(69)	50(5)	53(5)	75(6)	25(4)	13(4)	0(4)
C(70)	42(5)	78(7)	114(9)	26(6)	4(5)	8(5)
C(71)	44(5)	57(6)	111(8)	11(6)	28(5)	-4(4)
C(72)	49(5)	55(6)	82(7)	13(5)	27(5)	3(4)
C(73)	41(4)	40(4)	68(5)	19(4)	28(4)	5(3)
C(74)	51(4)	26(4)	45(4)	10(3)	3(3)	-5(3)
C(75)	29(3)	35(4)	36(4)	21(3)	5(3)	3(3)
C(76)	58(5)	43(4)	53(5)	23(4)	14(4)	9(4)
C(77)	83(6)	48(5)	39(4)	21(4)	24(4)	9(4)
C(78)	37(4)	69(6)	46(4)	36(4)	11(3)	-1(3)
C(79)	37(4)	42(4)	58(5)	26(4)	6(3)	1(3)
C(80)	30(3)	41(4)	38(4)	19(3)	5(3)	4(3)
C(81)	36(4)	51(4)	37(4)	25(3)	8(3)	10(3)
C(82)	40(4)	37(4)	43(4)	20(3)	8(3)	9(3)
C(83)	33(3)	38(4)	40(4)	17(3)	5(3)	3(3)
C(84)	38(4)	47(4)	38(4)	20(3)	6(3)	13(3)
C(85)	39(4)	51(4)	29(3)	14(3)	6(3)	14(3)
C(86)	52(5)	64(5)	60(5)	40(4)	26(4)	14(4)
C(87)	58(5)	43(5)	68(5)	35(4)	10(4)	10(4)
C(88)	34(4)	51(5)	53(4)	18(4)	12(3)	10(3)
C(89)	38(4)	44(4)	53(5)	17(4)	2(3)	2(3)
C(90)	57(5)	50(5)	42(4)	15(4)	10(4)	12(4)
N(91)	34(3)	29(3)	42(3)	17(2)	13(2)	3(2)
C(92)	36(4)	27(3)	43(4)	13(3)	13(3)	-2(3)
C(93)	35(4)	55(5)	47(4)	17(4)	11(3)	10(3)
Sb(1)	29(1)	40(1)	42(1)	17(1)	11(1)	7(1)
F(1)	36(2)	56(3)	60(3)	27(2)	3(2)	7(2)
F(2)	41(2)	58(3)	50(2)	24(2)	18(2)	14(2)
F(3)	61(3)	42(3)	65(3)	14(2)	10(2)	11(2)
F(4)	48(2)	57(3)	52(3)	25(2)	17(2)	3(2)
F(5)	42(2)	37(2)	60(3)	16(2)	15(2)	11(2)
F(6)	33(2)	63(3)	59(3)	28(2)	5(2)	5(2)
Sb(2)	35(2)	50(1)	49(1)	24(1)	8(1)	-3(1)
F(7)	60(3)	87(5)	49(3)	24(3)	15(2)	1(3)
F(8)	42(4)	79(7)	60(4)	15(5)	10(3)	-4(4)
F(9)	119(9)	78(7)	94(5)	43(5)	27(7)	43(6)
F(10)	79(7)	81(8)	85(4)	20(6)	9(6)	-34(5)

F(11)	53(6)	88(8)	84(5)	36(7)	10(5)	22(6)
F(12)	56(6)	81(6)	54(3)	35(3)	20(3)	2(4)
Sb(2')	35(2)	50(1)	49(1)	24(1)	8(1)	-3(1)
F(7')	60(3)	87(5)	49(3)	24(3)	15(2)	1(3)
F(8')	42(4)	79(7)	60(4)	15(5)	10(3)	-4(4)
F(9')	119(9)	78(7)	94(5)	43(5)	27(7)	43(6)
F(10')	79(7)	81(8)	85(4)	20(6)	9(6)	-34(5)
F(11')	53(6)	88(8)	84(5)	36(7)	10(5)	22(6)
F(12')	56(6)	81(6)	54(3)	35(3)	20(3)	2(4)
Sb(3)	33(1)	75(1)	51(1)	27(1)	8(1)	19(1)
F(13)	39(2)	65(3)	56(3)	21(2)	16(2)	10(2)
F(14)	51(3)	105(4)	71(3)	35(3)	2(2)	36(3)
F(15)	39(2)	68(3)	103(4)	5(3)	6(3)	11(2)
F(16)	37(2)	76(3)	61(3)	38(2)	13(2)	19(2)
F(17)	41(2)	95(4)	73(3)	55(3)	18(2)	13(2)
F(18)	66(3)	138(5)	48(3)	17(3)	6(2)	53(3)
Sb(4)	35(1)	39(1)	46(1)	18(1)	7(1)	-2(1)
F(20)	81(5)	65(4)	81(5)	28(4)	10(4)	28(4)
F(21)	39(3)	73(5)	117(5)	51(4)	11(3)	2(3)
F(22)	147(7)	86(5)	70(4)	29(4)	-3(5)	-16(5)
F(23)	65(4)	97(5)	113(6)	33(4)	39(4)	9(4)
F(24)	113(5)	65(4)	58(4)	19(3)	0(4)	-13(4)
F(25)	61(5)	73(4)	121(4)	68(3)	-3(4)	4(3)
Sb(4')	35(1)	39(1)	46(1)	18(1)	7(1)	-2(1)
F(20')	81(5)	65(4)	81(5)	28(4)	10(4)	28(4)
F(21')	39(3)	73(5)	117(5)	51(4)	11(3)	2(3)
F(22')	147(7)	86(5)	70(4)	29(4)	-3(5)	-16(5)
F(23')	65(4)	97(5)	113(6)	33(4)	39(4)	9(4)
F(24')	113(5)	65(4)	58(4)	19(3)	0(4)	-13(4)
F(25')	61(5)	73(4)	121(4)	68(3)	-3(4)	4(3)
C(101)	52(5)	81(6)	50(5)	22(4)	12(4)	22(4)
Cl(1)	46(1)	53(1)	80(2)	22(1)	17(1)	9(1)
Cl(2)	83(2)	56(1)	59(1)	13(1)	10(1)	14(1)

Table S24 – Hydrogen coordinates (x 10^4) and isotropic displacement parameters (Å^{2}x 10 ^{3}) for CCDC 2117074.

	x	y	z	U(eq)
H(10A)	6005	4386	4219	57
H(10B)	5946	5222	4808	57
H(11A)	7404	5117	4626	85
H(11B)	7198	5918	4393	85
H(11C)	7251	5098	3784	85
H(13)	3120	6947	2475	52
H(14)	1942	7161	1866	58
H(15)	2013	7316	683	65
H(16)	3301	7271	96	68
H(17)	4507	7074	705	55
H(19)	6166	8259	2454	47
H(20)	7391	8734	1884	59
H(21)	7725	7939	777	57
H(22)	6833	6667	240	56
H(23)	5608	6191	809	52
H(19')	5885	8341	2080	47
H(20')	7074	8776	1451	59
H(21')	7698	7795	656	57
H(22')	7133	6380	491	56
H(23')	5945	5945	1120	52
H(24A)	5211	8592	3401	68
H(24B)	4198	8265	3413	68
H(24C)	4608	8384	2670	68
H(26)	4571	8458	5238	61
H(27)	4503	9188	6439	81
H(28)	5320	8924	7374	72
H(29)	6092	7874	7120	61
H(30)	6096	7090	5938	55
H(36A)	3282	3871	3314	68
H(36B)	3705	4826	3642	68
H(36C)	4284	4129	3570	68
H(37A)	2217	4563	2223	58

H(37B)	2600	5469	2115	58
H(37C)	2692	5272	2896	58
H(38A)	2957	4498	644	73
H(38B)	3878	4956	466	73
H(38C)	3285	5465	998	73
H(39A)	4922	3500	600	85
H(39B)	5740	4155	1017	85
H(39C)	5090	4434	507	85
H(40A)	5191	3066	2065	77
H(40B)	5257	3602	2887	77
H(40C)	5895	3903	2314	77
H(43A)	8106	5919	2728	95
H(43B)	8002	6612	2300	95
H(43C)	8042	5691	1858	95
H(60A)	800	4853	3203	47
H(60B)	689	4810	4034	47
H(61A)	-380	5437	3726	75
H(61B)	-841	4525	3769	75
H(61C)	-655	4729	2995	75
H(63)	895	420	1581	54
H(64)	1439	-792	1173	62
H(65)	646	-2055	1257	63
H(66)	-688	-2139	1762	62
H(67)	-1240	-934	2184	56
H(69)	-2094	893	1306	70
H(70)	-3618	582	1415	94
H(71)	-4167	60	2347	88
H(72)	-3271	-147	3247	76
H(73)	-1775	130	3160	59
H(74A)	-905	1181	541	63
H(74B)	-800	318	713	63
H(74C)	26	937	550	63
H(76)	587	2805	37	59
H(77)	996	3532	-855	66
H(78)	1267	4974	-547	58
H(79)	1162	5693	644	53
H(80)	768	4967	1535	42

H(86A)	-592	1312	5244	81
H(86B)	-1121	781	4507	81
H(86C)	-1172	1739	4800	81
H(87A)	598	-58	4121	79
H(87B)	419	-154	3268	79
H(87C)	-359	-120	3796	79
H(88A)	2285	1179	3525	67
H(88B)	2036	1569	2875	67
H(88C)	1603	638	2883	67
H(89A)	2345	3060	4158	67
H(89B)	1633	3594	4055	67
H(89C)	1942	3000	3362	67
H(90A)	295	3170	5376	73
H(90B)	-424	3172	4773	73
H(90C)	537	3668	4761	73
H(93A)	-2725	3171	3397	67
H(93B)	-3131	2365	3672	67
H(93C)	-3060	2308	2821	67
H(10C)	-269	7539	3212	70
H(10D)	310	7020	3556	70

3. Molecular Structure of 5·THF (CCDC 2117073)

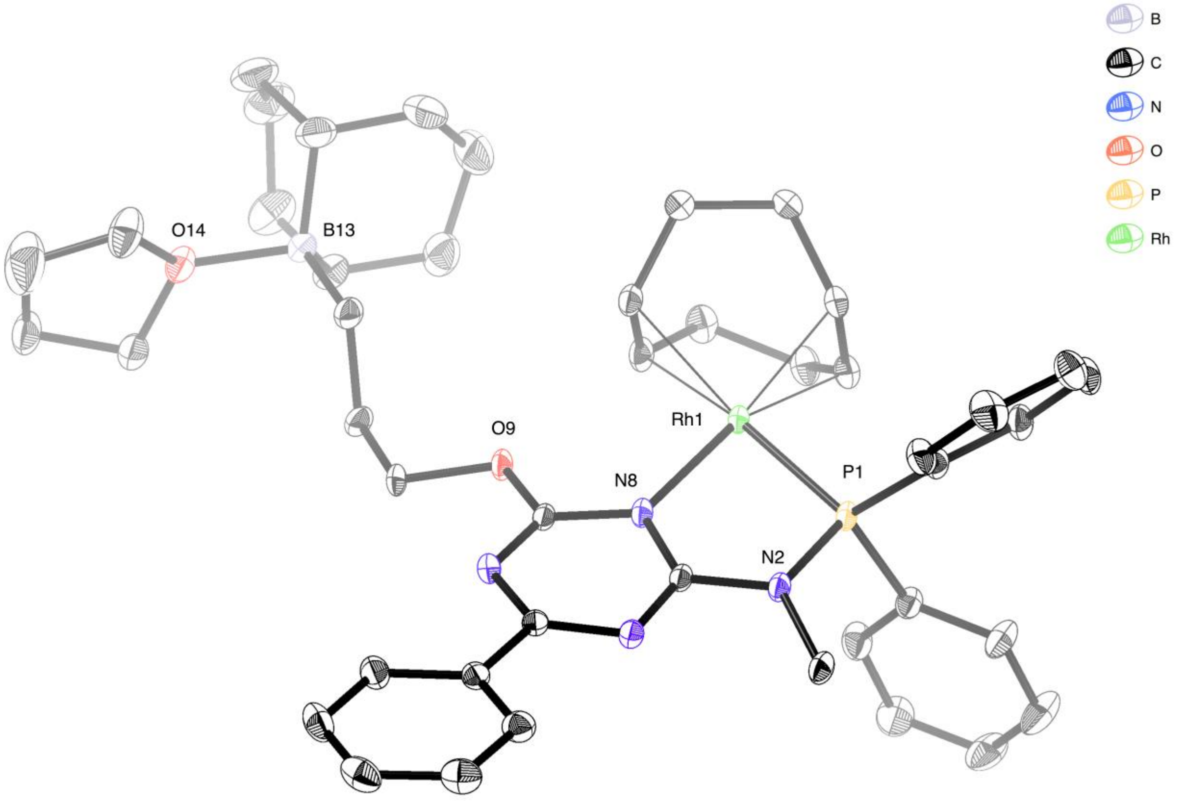

Molecular structure of complex **5·THF**. Suitable single crystals were obtained by slow diffusion of a concentrated solution of **5** in THF into an Et_2O/pentane mixture of solvents. The molecular structure is shown with thermal ellipsoids drawn at the 50% probability level. For clarity reasons, the hydrogen atoms and SbF_6 anion were omitted.

Table S25 – Crystal data and structure refinement for **5·THF** (CCDC 2117073).

Identification code	**5·THF**, CCDC 2117073	
Empirical formula	$C_{49.81} H_{70.02} B F_6 N_4 O_{3.20} P Rh Sb$	
Formula weight	1156.47	
Temperature	100(2) K	
Wavelength	0.71073 Å	
Crystal system	Triclinic	
Space group	P-1	
Unit cell dimensions	a = 10.9512(8) Å	a= 107.077(3)°.
	b = 14.5045(10) Å	b= 102.329(3)°.
	c = 17.8366(13) Å	g = 99.395(3)°.
Volume	2567.2(3) $Å^3$	
Z	2	
Density (calculated)	1.496 Mg/m^3	
Absorption coefficient	0.945 mm^{-1}	
F(000)	1185	
Crystal size	0.340 x 0.162 x 0.060 mm^3	
Theta range for data collection	2.010 to 30.547°.	
Index ranges	-15<=h<=15, -20<=k<=20, -25<=l<=25	
Reflections collected	161609	
Independent reflections	15704 [R(int) = 0.0566]	
Completeness to theta = 25.242°	99.9 %	
Absorption correction	Semi-empirical from equivalents	
Max. and min. transmission	0.9144 and 0.7711	
Refinement method	Full-matrix least-squares on F^2	
Data / restraints / parameters	15704 / 121 / 644	
Goodness-of-fit on F^2	1.200	
Final R indices [I>2sigma(I)]	R1 = 0.0458, wR2 = 0.0964	
R indices (all data)	R1 = 0.0533, wR2 = 0.0997	
Extinction coefficient	n/a	
Largest diff. peak and hole	1.532 and -1.658 e.$Å^{-3}$	

Table S26 – Atomic coordinates (x 10^4) and equivalent isotropic displacement parameters ($Å^2$x 10^3) for CCDC 2117073. U(eq) is defined as one-third of the trace of the orthogonalized U^{ij} tensor.

	x	y	z	U(eq)
Rh(1)	3953(1)	8350(1)	7650(1)	12(1)
P(1)	5119(1)	9414(1)	7246(1)	13(1)
N(2)	5244(2)	8697(2)	6331(1)	14(1)
C(3)	4584(2)	7720(2)	6018(2)	13(1)
N(4)	4716(2)	7185(2)	5306(1)	14(1)
C(5)	4068(2)	6229(2)	5018(2)	14(1)
N(6)	3232(2)	5836(2)	5348(1)	15(1)
C(7)	3133(2)	6428(2)	6045(2)	13(1)
N(8)	3841(2)	7376(1)	6453(1)	13(1)
O(9)	2286(2)	6086(1)	6381(1)	15(1)
C(10)	1425(3)	5100(2)	5874(2)	18(1)
C(11)	449(3)	4831(2)	6305(2)	20(1)
C(12)	961(3)	4486(2)	7016(2)	19(1)
B(13)	-65(3)	4117(2)	7465(2)	19(1)
O(14)	-885(2)	2943(1)	6856(1)	21(1)
C(15)	-224(3)	2138(2)	6721(2)	33(1)
C(16)	-1212(5)	1249(3)	6137(4)	67(1)
C(17)	-2364(3)	1604(2)	5790(2)	30(1)
C(18)	-1884(3)	2722(2)	6097(2)	26(1)
C(19)	567(3)	4016(2)	8330(2)	28(1)
C(20)	1275(4)	5074(3)	8924(2)	40(1)
C(21)	462(5)	5847(3)	8983(2)	46(1)
C(22)	-426(4)	5787(2)	8169(2)	34(1)
C(23)	-1155(3)	4720(2)	7598(2)	24(1)
C(24)	-2162(4)	4214(3)	7933(2)	35(1)
C(25)	-1610(4)	3942(3)	8683(2)	43(1)
C(26)	-446(4)	3483(3)	8640(2)	37(1)
C(27)	4421(2)	10372(2)	6995(2)	16(1)
C(28)	3086(3)	10122(2)	6654(2)	23(1)
C(29)	2490(3)	10771(2)	6357(2)	29(1)
C(30)	3218(3)	11680(2)	6418(2)	30(1)
C(31)	4539(3)	11948(2)	6772(2)	32(1)

C(32)	5146(3)	11293(2)	7055(2)	23(1)
C(33)	6767(2)	10014(2)	7839(2)	14(1)
C(34)	7036(3)	10854(2)	8537(2)	19(1)
C(35)	8298(3)	11279(2)	9028(2)	25(1)
C(36)	9291(3)	10865(2)	8827(2)	28(1)
C(37)	9031(3)	10035(2)	8137(2)	28(1)
C(38)	7773(3)	9602(2)	7647(2)	21(1)
C(39)	5861(3)	9121(2)	5809(2)	19(1)
C(40)	4271(2)	5563(2)	4272(2)	15(1)
C(41)	3507(3)	4588(2)	3915(2)	20(1)
C(42)	3686(3)	3958(2)	3213(2)	25(1)
C(43)	4604(3)	4291(2)	2869(2)	26(1)
C(44)	5381(3)	5255(2)	3230(2)	25(1)
C(45)	5220(3)	5892(2)	3933(2)	20(1)
C(51)	3243(3)	7158(2)	8158(2)	17(1)
C(52)	2229(2)	7568(2)	7956(2)	18(1)
C(53)	1793(3)	8319(2)	8569(2)	21(1)
C(54)	2390(3)	9400(2)	8662(2)	21(1)
C(55)	3757(3)	9571(2)	8601(2)	17(1)
C(56)	4722(2)	9160(2)	8934(2)	16(1)
C(57)	4568(3)	8537(2)	9478(2)	19(1)
C(58)	4129(3)	7415(2)	9003(2)	20(1)
Sb(60)	-523(1)	7859(1)	5795(1)	22(1)
F(61)	-2126(2)	7759(2)	6014(2)	68(1)
F(62)	-527(2)	6610(2)	5881(2)	48(1)
F(63)	314(3)	8486(2)	6900(1)	63(1)
F(64)	-541(4)	9093(2)	5708(2)	89(1)
F(65)	-1353(3)	7253(2)	4682(1)	47(1)
F(66)	1089(3)	7899(2)	5599(2)	66(1)
O(71)	7409(3)	7914(2)	10107(1)	35(1)
C(72)	7838(3)	7473(3)	9422(2)	31(1)
C(73)	7905(4)	8163(3)	8940(2)	40(1)
C(74)	7286(6)	7295(4)	10575(3)	56(1)
C(75)	6888(8)	7814(5)	11309(3)	88(2)
O(81)	4860(19)	5184(13)	9942(8)	62(4)
C(82)	5390(20)	5207(15)	10762(9)	50(4)
C(83)	4830(30)	5980(20)	11258(14)	83(8)

C(84)	5449(19)	4543(15)	9441(10)	52(4)
C(85)	4810(30)	4460(20)	8583(10)	73(7)

Table S27 – Bond lengths [Å] and angles [°] for CCDC 2117073.

Rh(1)-C(55)	2.139(2)
Rh(1)-C(56)	2.147(2)
Rh(1)-N(8)	2.151(2)
Rh(1)-P(1)	2.2220(6)
Rh(1)-C(51)	2.280(3)
Rh(1)-C(52)	2.293(2)
P(1)-N(2)	1.705(2)
P(1)-C(33)	1.807(3)
P(1)-C(27)	1.812(3)
N(2)-C(3)	1.368(3)
N(2)-C(39)	1.473(3)
C(3)-N(4)	1.333(3)
C(3)-N(8)	1.370(3)
N(4)-C(5)	1.340(3)
C(5)-N(6)	1.330(3)
C(5)-C(40)	1.482(3)
N(6)-C(7)	1.324(3)
C(7)-O(9)	1.314(3)
C(7)-N(8)	1.359(3)
O(9)-C(10)	1.479(3)
C(10)-C(11)	1.508(4)
C(11)-C(12)	1.527(4)
C(12)-B(13)	1.622(4)
B(13)-C(23)	1.608(4)
B(13)-C(19)	1.610(4)
B(13)-O(14)	1.698(4)
O(14)-C(18)	1.459(4)
O(14)-C(15)	1.459(4)
C(15)-C(16)	1.481(6)
C(16)-C(17)	1.518(6)
C(17)-C(18)	1.510(4)
C(19)-C(26)	1.539(5)
C(19)-C(20)	1.544(5)
C(20)-C(21)	1.535(6)
C(21)-C(22)	1.535(6)

C(22)-C(23)	1.547(4)
C(23)-C(24)	1.546(4)
C(24)-C(25)	1.535(5)
C(25)-C(26)	1.536(6)
C(27)-C(28)	1.398(4)
C(27)-C(32)	1.400(4)
C(28)-C(29)	1.392(4)
C(29)-C(30)	1.387(4)
C(30)-C(31)	1.388(5)
C(31)-C(32)	1.393(4)
C(33)-C(38)	1.394(4)
C(33)-C(34)	1.398(3)
C(34)-C(35)	1.395(4)
C(35)-C(36)	1.387(5)
C(36)-C(37)	1.384(4)
C(37)-C(38)	1.392(4)
C(40)-C(45)	1.396(4)
C(40)-C(41)	1.401(4)
C(41)-C(42)	1.391(4)
C(42)-C(43)	1.379(5)
C(43)-C(44)	1.393(4)
C(44)-C(45)	1.388(4)
C(51)-C(52)	1.374(4)
C(51)-C(58)	1.509(4)
C(52)-C(53)	1.518(4)
C(53)-C(54)	1.543(4)
C(54)-C(55)	1.510(4)
C(55)-C(56)	1.403(4)
C(56)-C(57)	1.524(4)
C(57)-C(58)	1.535(4)
Sb(60)-F(64)	1.844(3)
Sb(60)-F(63)	1.860(2)
Sb(60)-F(62)	1.862(2)
Sb(60)-F(66)	1.866(3)
Sb(60)-F(65)	1.866(2)
Sb(60)-F(61)	1.871(3)
O(71)-C(74)	1.403(5)

O(71)-C(72)	1.416(4)
C(72)-C(73)	1.501(5)
C(74)-C(75)	1.498(6)
O(81)-C(84)	1.425(11)
O(81)-C(82)	1.441(10)
C(82)-C(83)	1.515(11)
C(84)-C(85)	1.502(11)

C(55)-Rh(1)-C(56)	38.22(10)
C(55)-Rh(1)-N(8)	160.95(9)
C(56)-Rh(1)-N(8)	158.78(9)
C(55)-Rh(1)-P(1)	89.61(7)
C(56)-Rh(1)-P(1)	96.05(7)
N(8)-Rh(1)-P(1)	80.86(6)
C(55)-Rh(1)-C(51)	95.66(10)
C(56)-Rh(1)-C(51)	80.08(9)
N(8)-Rh(1)-C(51)	97.62(8)
P(1)-Rh(1)-C(51)	165.43(7)
C(55)-Rh(1)-C(52)	80.40(10)
C(56)-Rh(1)-C(52)	87.15(10)
N(8)-Rh(1)-C(52)	103.13(9)
P(1)-Rh(1)-C(52)	159.50(7)
C(51)-Rh(1)-C(52)	34.98(10)
N(2)-P(1)-C(33)	104.27(11)
N(2)-P(1)-C(27)	103.16(11)
C(33)-P(1)-C(27)	106.27(12)
N(2)-P(1)-Rh(1)	103.94(7)
C(33)-P(1)-Rh(1)	118.97(8)
C(27)-P(1)-Rh(1)	118.10(9)
C(3)-N(2)-C(39)	118.5(2)
C(3)-N(2)-P(1)	118.39(17)
C(39)-N(2)-P(1)	122.38(16)
N(4)-C(3)-N(2)	116.4(2)
N(4)-C(3)-N(8)	125.6(2)
N(2)-C(3)-N(8)	118.0(2)
C(3)-N(4)-C(5)	115.1(2)
N(6)-C(5)-N(4)	124.5(2)

N(6)-C(5)-C(40)	117.3(2)
N(4)-C(5)-C(40)	118.2(2)
C(7)-N(6)-C(5)	116.2(2)
O(9)-C(7)-N(6)	118.5(2)
O(9)-C(7)-N(8)	116.0(2)
N(6)-C(7)-N(8)	125.4(2)
C(7)-N(8)-C(3)	112.6(2)
C(7)-N(8)-Rh(1)	129.26(16)
C(3)-N(8)-Rh(1)	118.13(15)
C(7)-O(9)-C(10)	114.61(19)
O(9)-C(10)-C(11)	109.1(2)
C(10)-C(11)-C(12)	115.0(2)
C(11)-C(12)-B(13)	117.3(2)
C(23)-B(13)-C(19)	106.7(2)
C(23)-B(13)-C(12)	117.7(2)
C(19)-B(13)-C(12)	114.9(2)
C(23)-B(13)-O(14)	105.1(2)
C(19)-B(13)-O(14)	104.8(2)
C(12)-B(13)-O(14)	106.5(2)
C(18)-O(14)-C(15)	106.3(2)
C(18)-O(14)-B(13)	123.2(2)
C(15)-O(14)-B(13)	120.5(2)
O(14)-C(15)-C(16)	105.5(3)
C(15)-C(16)-C(17)	107.3(3)
C(18)-C(17)-C(16)	103.9(3)
O(14)-C(18)-C(17)	104.3(2)
C(26)-C(19)-C(20)	113.1(3)
C(26)-C(19)-B(13)	111.7(3)
C(20)-C(19)-B(13)	107.1(2)
C(21)-C(20)-C(19)	115.2(3)
C(20)-C(21)-C(22)	115.3(3)
C(21)-C(22)-C(23)	114.6(3)
C(24)-C(23)-C(22)	113.3(3)
C(24)-C(23)-B(13)	112.2(2)
C(22)-C(23)-B(13)	105.9(3)
C(25)-C(24)-C(23)	115.5(3)
C(24)-C(25)-C(26)	114.5(3)

C(25)-C(26)-C(19)	114.5(3)
C(28)-C(27)-C(32)	119.3(2)
C(28)-C(27)-P(1)	116.60(19)
C(32)-C(27)-P(1)	123.9(2)
C(29)-C(28)-C(27)	120.3(3)
C(30)-C(29)-C(28)	119.8(3)
C(29)-C(30)-C(31)	120.4(3)
C(30)-C(31)-C(32)	120.0(3)
C(31)-C(32)-C(27)	120.1(3)
C(38)-C(33)-C(34)	119.2(2)
C(38)-C(33)-P(1)	120.57(19)
C(34)-C(33)-P(1)	120.0(2)
C(35)-C(34)-C(33)	120.2(3)
C(36)-C(35)-C(34)	120.0(3)
C(37)-C(36)-C(35)	120.0(3)
C(36)-C(37)-C(38)	120.4(3)
C(37)-C(38)-C(33)	120.2(3)
C(45)-C(40)-C(41)	120.2(2)
C(45)-C(40)-C(5)	120.8(2)
C(41)-C(40)-C(5)	119.0(2)
C(42)-C(41)-C(40)	119.4(3)
C(43)-C(42)-C(41)	120.3(3)
C(42)-C(43)-C(44)	120.4(3)
C(45)-C(44)-C(43)	120.0(3)
C(44)-C(45)-C(40)	119.6(3)
C(52)-C(51)-C(58)	125.9(2)
C(52)-C(51)-Rh(1)	73.00(15)
C(58)-C(51)-Rh(1)	107.12(16)
C(51)-C(52)-C(53)	124.3(2)
C(51)-C(52)-Rh(1)	72.02(15)
C(53)-C(52)-Rh(1)	110.09(16)
C(52)-C(53)-C(54)	112.4(2)
C(55)-C(54)-C(53)	113.8(2)
C(56)-C(55)-C(54)	126.0(2)
C(56)-C(55)-Rh(1)	71.17(14)
C(54)-C(55)-Rh(1)	110.00(17)
C(55)-C(56)-C(57)	124.3(2)

C(55)-C(56)-Rh(1)	70.61(14)
C(57)-C(56)-Rh(1)	114.47(16)
C(56)-C(57)-C(58)	113.2(2)
C(51)-C(58)-C(57)	113.4(2)
F(64)-Sb(60)-F(63)	88.64(15)
F(64)-Sb(60)-F(62)	179.25(15)
F(63)-Sb(60)-F(62)	91.59(12)
F(64)-Sb(60)-F(66)	92.51(17)
F(63)-Sb(60)-F(66)	88.44(13)
F(62)-Sb(60)-F(66)	88.21(13)
F(64)-Sb(60)-F(65)	90.34(13)
F(63)-Sb(60)-F(65)	178.91(13)
F(62)-Sb(60)-F(65)	89.43(10)
F(66)-Sb(60)-F(65)	91.23(13)
F(64)-Sb(60)-F(61)	90.68(17)
F(63)-Sb(60)-F(61)	90.73(13)
F(62)-Sb(60)-F(61)	88.61(14)
F(66)-Sb(60)-F(61)	176.68(15)
F(65)-Sb(60)-F(61)	89.66(13)
C(74)-O(71)-C(72)	111.8(3)
O(71)-C(72)-C(73)	108.6(3)
O(71)-C(74)-C(75)	109.6(4)
C(84)-O(81)-C(82)	106.0(11)
O(81)-C(82)-C(83)	102.4(12)
O(81)-C(84)-C(85)	105.1(12)

Symmetry transformations used to generate equivalent atoms:

Table S28 – Anisotropic displacement parameters ($Å^2$x 10^3) for CCDC 2117073. The anisotropic displacement factor exponent takes the form: $-2p^2[h^2 a^{*2}U^{11} + ... + 2 h k a^* b^* U^{12}]$.

	U^{11}	U^{22}	U^{33}	U^{23}	U^{13}	U^{12}
Rh(1)	11(1)	8(1)	14(1)	3(1)	3(1)	0(1)
P(1)	12(1)	8(1)	16(1)	3(1)	2(1)	1(1)
N(2)	15(1)	10(1)	15(1)	4(1)	4(1)	-1(1)
C(3)	11(1)	10(1)	16(1)	5(1)	1(1)	1(1)
N(4)	14(1)	12(1)	15(1)	5(1)	3(1)	1(1)
C(5)	14(1)	12(1)	14(1)	5(1)	1(1)	3(1)
N(6)	14(1)	11(1)	15(1)	3(1)	2(1)	0(1)
C(7)	12(1)	9(1)	15(1)	5(1)	1(1)	1(1)
N(8)	11(1)	9(1)	16(1)	3(1)	2(1)	0(1)
O(9)	14(1)	10(1)	16(1)	3(1)	3(1)	-2(1)
C(10)	18(1)	11(1)	17(1)	3(1)	1(1)	-6(1)
C(11)	18(1)	18(1)	22(1)	11(1)	2(1)	-3(1)
C(12)	16(1)	18(1)	22(1)	9(1)	2(1)	0(1)
B(13)	21(1)	16(1)	19(1)	9(1)	4(1)	2(1)
O(14)	19(1)	16(1)	29(1)	9(1)	5(1)	4(1)
C(15)	27(2)	20(1)	54(2)	12(1)	11(1)	8(1)
C(16)	62(3)	26(2)	91(3)	14(2)	-12(2)	11(2)
C(17)	31(2)	19(1)	35(2)	7(1)	8(1)	-4(1)
C(18)	27(1)	19(1)	28(1)	10(1)	1(1)	-1(1)
C(19)	30(2)	33(2)	25(1)	17(1)	5(1)	6(1)
C(20)	47(2)	45(2)	20(2)	13(1)	0(1)	-1(2)
C(21)	71(3)	33(2)	25(2)	-1(1)	16(2)	1(2)
C(22)	48(2)	22(1)	33(2)	7(1)	20(2)	9(1)
C(23)	26(1)	22(1)	27(1)	12(1)	11(1)	7(1)
C(24)	35(2)	38(2)	47(2)	22(2)	24(2)	15(1)
C(25)	54(2)	47(2)	41(2)	23(2)	28(2)	11(2)
C(26)	45(2)	42(2)	33(2)	25(2)	14(2)	9(2)
C(27)	15(1)	13(1)	20(1)	6(1)	4(1)	3(1)
C(28)	15(1)	18(1)	30(1)	7(1)	-1(1)	2(1)
C(29)	19(1)	27(1)	38(2)	14(1)	0(1)	6(1)
C(30)	27(2)	29(2)	43(2)	23(1)	7(1)	12(1)
C(31)	25(2)	26(1)	55(2)	26(2)	12(1)	8(1)

C(32)	17(1)	18(1)	38(2)	17(1)	7(1)	4(1)
C(33)	13(1)	11(1)	18(1)	5(1)	2(1)	-1(1)
C(34)	18(1)	15(1)	20(1)	3(1)	6(1)	-2(1)
C(35)	26(1)	19(1)	20(1)	1(1)	2(1)	-7(1)
C(36)	18(1)	29(2)	29(2)	7(1)	-4(1)	-4(1)
C(37)	15(1)	32(2)	32(2)	8(1)	0(1)	6(1)
C(38)	16(1)	18(1)	23(1)	3(1)	1(1)	3(1)
C(39)	21(1)	14(1)	21(1)	6(1)	6(1)	-3(1)
C(40)	17(1)	14(1)	15(1)	5(1)	2(1)	5(1)
C(41)	20(1)	17(1)	19(1)	4(1)	2(1)	3(1)
C(42)	25(1)	18(1)	22(1)	-2(1)	-1(1)	6(1)
C(43)	29(2)	30(1)	18(1)	2(1)	5(1)	16(1)
C(44)	26(1)	30(2)	23(1)	10(1)	11(1)	12(1)
C(45)	22(1)	20(1)	19(1)	7(1)	6(1)	7(1)
C(51)	18(1)	12(1)	18(1)	4(1)	5(1)	-1(1)
C(52)	15(1)	14(1)	20(1)	4(1)	5(1)	-3(1)
C(53)	17(1)	21(1)	24(1)	4(1)	9(1)	2(1)
C(54)	17(1)	17(1)	24(1)	1(1)	5(1)	5(1)
C(55)	18(1)	13(1)	17(1)	1(1)	5(1)	2(1)
C(56)	15(1)	12(1)	16(1)	1(1)	2(1)	0(1)
C(57)	22(1)	18(1)	14(1)	4(1)	1(1)	2(1)
C(58)	25(1)	16(1)	17(1)	6(1)	3(1)	2(1)
Sb(60)	21(1)	15(1)	23(1)	1(1)	-2(1)	5(1)
F(61)	29(1)	74(2)	79(2)	-9(2)	15(1)	17(1)
F(62)	57(2)	34(1)	54(1)	25(1)	3(1)	15(1)
F(63)	57(2)	71(2)	30(1)	-16(1)	-13(1)	31(1)
F(64)	123(3)	19(1)	97(2)	14(1)	-13(2)	10(2)
F(65)	69(2)	32(1)	27(1)	5(1)	-11(1)	14(1)
F(66)	39(1)	70(2)	70(2)	1(1)	28(1)	-10(1)
O(71)	47(1)	38(1)	28(1)	14(1)	11(1)	26(1)
C(72)	30(2)	31(2)	28(2)	5(1)	4(1)	13(1)
C(73)	45(2)	39(2)	39(2)	13(2)	15(2)	13(2)
C(74)	87(3)	59(2)	51(2)	34(2)	34(2)	48(2)
C(75)	163(6)	95(4)	62(3)	52(3)	67(4)	90(4)
O(81)	58(6)	62(7)	61(6)	23(5)	16(5)	-2(5)
C(82)	47(7)	62(7)	51(6)	33(6)	19(6)	6(6)
C(83)	74(13)	92(13)	87(13)	37(11)	24(11)	20(11)

C(84)	39(7)	53(7)	66(7)	25(6)	18(6)	7(5)
C(85)	70(12)	63(11)	85(12)	30(10)	17(10)	8(10)

Table S29 – Hydrogen coordinates (x 10^4) and isotropic displacement parameters ($Å^2$x 10^3) for CCDC 2117073.

	x	y	z	U(eq)
H(10A)	1936	4595	5785	21
H(10B)	979	5118	5336	21
H(11A)	-278	4296	5901	23
H(11B)	106	5417	6512	23
H(12A)	1651	5041	7432	23
H(12B)	1364	3934	6813	23
H(15A)	120	2029	7240	40
H(15B)	497	2294	6491	40
H(16A)	-1468	785	6419	80
H(16B)	-868	899	5694	80
H(17A)	-2628	1335	5186	36
H(17B)	-3104	1402	5992	36
H(18A)	-1525	2947	5699	32
H(18B)	-2588	3049	6199	32
H(19)	1221	3614	8257	34
H(20A)	1596	5039	9475	48
H(20B)	2034	5308	8752	48
H(21A)	1051	6518	9235	56
H(21B)	-73	5770	9354	56
H(22A)	97	6099	7879	40
H(22B)	-1066	6179	8286	40
H(23)	-1608	4752	7060	28
H(24A)	-2730	4664	8076	42
H(24B)	-2704	3601	7492	42
H(25A)	-2298	3465	8752	52
H(25B)	-1351	4549	9173	52
H(26A)	-760	2780	8277	44
H(26B)	-26	3489	9192	44
H(28)	2584	9506	6625	27
H(29)	1586	10591	6114	34
H(30)	2810	12122	6215	36
H(31)	5030	12578	6823	38

H(32)	6052	11471	7288	28
H(34)	6356	11135	8677	23
H(35)	8477	11851	9500	30
H(36)	10150	11152	9164	34
H(37)	9714	9758	7997	34
H(38)	7599	9023	7181	25
H(39A)	6431	8715	5606	28
H(39B)	6364	9802	6128	28
H(39C)	5197	9129	5347	28
H(41)	2873	4359	4152	24
H(42)	3172	3296	2969	30
H(43)	4708	3861	2382	31
H(44)	6021	5476	2994	30
H(45)	5752	6548	4183	24
H(51)	3136	6462	7792	20
H(52)	1519	7118	7468	21
H(53A)	2037	8230	9106	25
H(53B)	842	8192	8392	25
H(54A)	1851	9583	8234	25
H(54B)	2374	9846	9198	25
H(55)	4092	10236	8565	21
H(56)	5611	9589	9083	19
H(57A)	3930	8737	9772	23
H(57B)	5401	8678	9891	23
H(58A)	4899	7151	8956	24
H(58B)	3681	7084	9315	24
H(72A)	7234	6827	9078	37
H(72B)	8699	7355	9605	37
H(73A)	8256	7888	8484	60
H(73B)	8464	8814	9294	60
H(73C)	7038	8238	8727	60
H(74A)	8119	7129	10749	67
H(74B)	6633	6668	10242	67
H(75A)	6014	7898	11135	132
H(75B)	7485	8467	11604	132
H(75C)	6904	7416	11669	132
H(82A)	6339	5408	10927	61

H(82B)	5105	4551	10817	61
H(83A)	5125	6059	11837	124
H(83B)	5107	6618	11184	124
H(83C)	3883	5768	11075	124
H(84A)	5307	3882	9506	62
H(84B)	6388	4829	9586	62
H(85A)	5171	4026	8203	110
H(85B)	3881	4180	8454	110
H(85C)	4956	5122	8533	110

References

[1] H. S. Jung, T. Yun, Y. Cho, H. B. Jeon, *Tetrahedron* **2016**, *72*, 5988-5993.

[2] J. L. Hauser, G. Amberchan, M. Tso, R. Manley, K. Bustillo, J. Cooper, J. H. Golden, B. Singaram, S. R. J. Oliver, *ACS Appl. Nano Mater.* **2019**, *2*, 1472-1483.

[3] V. Zubar, A. Dewanji, M. Rueping, *Org. Lett.* **2021**, *23*, 2742-2747.

[4] H. Goksu, N. Zengin, H. Burhan, K. Cellat, F. Sen, *Sci. Rep.* **2020**, *10*, 8043.

[5] P. Ji, K. Manna, Z. Lin, X. Feng, A. Urban, Y. Song, W. Lin, *J. Am. Chem. Soc.* **2017**, *139*, 7004-7011.

[6] Z. Qiu, L. Lv, J. Li, C. C. Li, C. J. Li, *Chem. Sci.* **2019**, *10*, 4775-4781.

[7] Y. Monguchi, T. Maejima, S. Mori, T. Maegawa, H. Sajiki, *Chem. - Eur. J.* **2010**, *16*, 7372-7375.

[8] Y. Sunada, H. Kawakami, T. Imaoka, Y. Motoyama, H. Nagashima, *Angew. Chem., Int. Ed.* **2009**, *48*, 9511-9514.

[9] M. R. Maddani, S. K. Moorthy, K. R. Prabhu, *Tetrahedron* **2010**, *66*, 329-333.

[10] J. Spencer, R. P. Rathnam, H. Patel, N. Anjum, *Tetrahedron* **2008**, *64*, 10195-10200.

[11] T. Yamada, Y. Kobayashi, N. Ito, T. Ichikawa, K. Park, K. Kunishima, S. Ueda, M. Mizuno, T. Adachi, Y. Sawama, Y. Monguchi, H. Sajiki, *ACS Omega* **2019**, *4*, 10243-10251.

[12] M. A. Khanfar, L. Quinti, H. Wang, S. H. Choi, A. G. Kazantsev, R. B. Silverman, *Eur. J. Med. Chem.* **2014**, *76*, 414-426.

[13] X. Jin, Y. Koizumi, K. Yamaguchi, K. Nozaki, N. Mizuno, *J. Am. Chem. Soc.* **2017**, *139*, 13821-13829.

[14] P. Tegeder, M. Freitag, K. M. Chepiga, S. Muratsugu, N. Moller, S. Lamping, M. Tada, F. Glorius, B. J. Ravoo, *Chem. - Eur. J.* **2018**, *24*, 18682-18688.

[15] T. Okazaki, T. Adachi, T. Kitagawa, *Bull. Chem. Soc. Jpn.* **2013**, *86*, 464-471.

[16] X. Hu, G. Zhang, L. Nie, T. Kong, A. Lei, *Nat. Commun.* **2019**, *10*, 5467.

[17] C. Y. Wang, C. F. Fu, Y. H. Liu, S. M. Peng, S. T. Liu, *Inorg. Chem.* **2007**, *46*, 5779-5786.

[18] S. Bhunia, S. V. Kumar, D. Ma, *J. Org. Chem.* **2017**, *82*, 12603-12612.

[19] K. N. Hojczyk, P. Feng, C. Zhan, M.-Y. Ngai, *Angew. Chem.* **2014**, *126*, 14787-14791.

[20] R. Shintani, G. C. Fu, *Angew. Chem., Int. Ed.* **2003**, *42*, 4082-4085.

[21] M. S. Jeletic, M. T. Mock, A. M. Appel, J. C. Linehan, *J. Am. Chem. Soc.* **2013**, *135*, 11533-11536.

[22] A. Rajapakse, R. Hillebrand, S. M. Lewis, Z. D. Parsons, C. L. Barnes, K. S. J. A. C. S. E. S. R. O. Gates, **2014**, *70*, 322-324.

[23] A. O. Miller, G. G. Furin, *J. Fluorine Chem.* **1987**, *36*, 247-272.

[24] D. R. Anton, R. H. Crabtree, *Organometallics* **1983**, *2*, 855-859.

[25] N. Yasukawa, Y. Miki, M. Kuwata, H. Sajiki, Y. Sawama, *ChemSusChem* **2020**, *13*, 5632-5637.

[26] G. W. Kramer, H. C. Brown, *J. Organomet. Chem.* **1974**, *73*, 1-15.

[27] B. Neumuller, F. Gahlmann, *Z. Anorg. Allg. Chem.* **1992**, *612*, 123-129.

[28] D. T. Yang, S. K. Mellerup, X. Wang, J. S. Lu, S. N. Wang, *Angew. Chem., Int. Ed.* **2015**, *54*, 5498-5501.

[29] E. Burkhardt, S. Pichlmair, *Vol. WO2009133045 (A1)* (Ed.: BASF), International, **2009**.

[30] a) G. Povie, G. Villa, L. Ford, D. Pozzi, C. H. Schiesser, P. Renaud, *Chem. Commun.* **2010**, *46*, 803-805; b) G. Povie, M. Marzorati, P. Bigler, P. Renaud, *J. Org. Chem.* **2013**, *78*, 1553-1558.

[31] G. Povie, M. Marzorati, P. Bigler, P. Renaud, *J. Org. Chem.* **2013**, *78*, 1553-1558.

[32] F. Neese, *Wiley Interdiscip. Rev. Comput. Mol. Sci.* **2012**, *2*, 73-78.

[33] a) A. Schäfer, H. Horn, R. Ahlrichs, *J. Chem. Phys.* **1992**, *97*, 2571-2577; b) A. Schäfer, C. Huber, R. Ahlrichs, *J. Chem. Phys.* **1994**, *100*, 5829-5835; c) F. Weigend, R. Ahlrichs, *Phys. Chem. Chem. Phys.* **2005**, *7*, 3297-3305.

[34] J. Tomasi, B. Mennucci, R. Cammi, *Chem. Rev.* **2005**, *105*, 2999-3093.

[35] J. P. Perdew, *Phys. Rev. B* **1986**, *33*, 8822-8824.

[36] a) K. Eichkorn, O. Treutler, H. Öhm, M. Häser, R. Ahlrichs, *Chem. Phys. Lett.* **1995**, *240*, 283-290; b) K. Eichkorn, O. Treutler, H. Öhm, M. Häser, R. Ahlrichs, *Chem. Phys. Lett.* **1995**, *242*, 652-660; c) K. Eichkorn, F. Weigend, O. Treutler, R. Ahlrichs, *Theor. Chem. Acc.* **1997**, *97*, 119-124.

[37] a) A. D. Becke, *Phys. Rev. A* **1988**, *38*, 3098-3100; b) C. Lee, W. Yang, R. G. Parr, *Phys. Rev. B* **1988**, *37*, 785-789; c) A. D. Becke, *J. Chem. Phys.* **1993**, *98*, 5648-5652.
[38] F. Neese, F. Wennmohs, A. Hansen, U. Becker, *Chem. Phys.* **2009**, *356*, 98-109.
[39] a) S. Grimme, J. Antony, S. Ehrlich, H. Krieg, *J. Chem. Phys.* **2010**, *132*, 154104; b) S. Grimme, S. Ehrlich, L. Goerigk, *J. Comput. Chem.* **2011**, *32*, 1456-1465.
[40] SADABS, Bruker–Siemens Area Detector Absorption and Other Correction, G.M. Sheldrick, University of Göttingen, Germany, 2012, Version 2012/1.
[41] ShelXTL MP Vers. 2013-2, Bruker AXS Inc., 1993-2013.
[42] Platon/Squeeze: A.L. Spek (2009) Acta Cryst. D2065, 2148-2155. Platon – A Multipurpose Crystallographic Tool, Utrecht University, Utrecht, The Netherlands, A.L. Spek (2011).
